# On-DNA C–H functionalization of electron-rich arenes for DNA-encoded libraries

**DOI:** 10.1038/s41557-025-01844-6

**Published:** 2025-06-16

**Authors:** Eduardo de Pedro Beato, Luca Torkowski, Philipp Hartmann, Lara Vogelsang, Karl-Josef Dietz, Tobias Ritter

**Affiliations:** 1 Max-Planck-Institut für Kohlenforschung, Mülheim an der Ruhr, Germany; 2 Institute of Organic Chemistry, RWTH Aachen University, Aachen, Germany; 3 Department of Biochemistry and Physiology of Plants, Faculty of Biology, Bielefeld University, Bielefeld, Germany

**Keywords:** Synthetic chemistry methodology, Combinatorial libraries

## Abstract

DNA-encoded libraries (DELs) are useful for hit discovery in the pharmaceutical industry. Although a large number of individually coded molecules are accessible through DELs, their structural diversity is limited because few transformations are benign and chemoselective enough to be applied in the presence of DNA in aqueous environments. In particular, C–H functionalization chemistry that could be ideally suited to increase structural diversity through late-stage functionalization is currently absent from DEL synthesis. Here we present a general C–H functionalization of electron-rich arenes on DNA. The development of a selenoxide reagent is key to achieving the regio- and chemoselective formation of arylselenonium salts in aqueous media. The introduction of arylselenonium salts offers a versatile linchpin on DNA conjugates, which gives access to a multitude of analogues through diverse subsequent reactions, including transition-metal-mediated and photochemical transformations for the formation of C–C, C–I and C–S bonds.

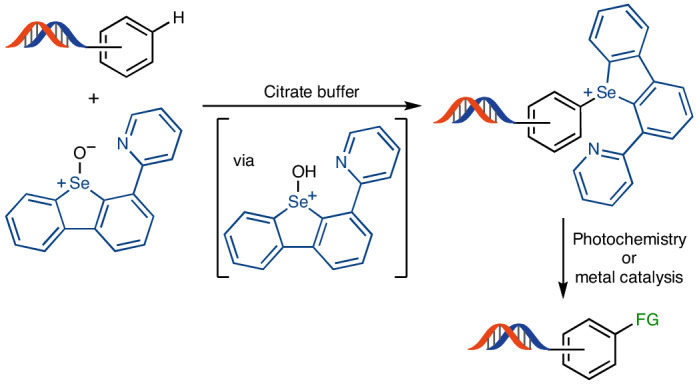

## Abstract (web summary)

C–H functionalization is a key reaction in organic synthesis. Now a selenoxide reagent has been developed for the formation of arylselenonium salts via C–H functionalization of DNA conjugates with high selectivity. The arylselenonium salts participate in various transformations to forge new C–C and C–X bonds.

## Main

In DNA-encoded libraries (DELs), the DNA sequence acts as a covalently attached molecular barcode for each individual molecule in the library and allows its unique identification^1^. The library can be screened in a single bioassay against a target protein, and potential hits can be identified through polymerase chain reaction (PCR) and sequencing^2^. The key advantage of DELs is the ability to screen up to billions of compounds at once with only a fraction of the investment required for high-throughput screening approaches^3^. However, because the crucial information to identify the molecules is contained in the DNA sequence, the chemical toolbox suitable for the construction of the library is restricted. The reaction chemistry must be executed with the molecule on DNA, while maintaining the functionality of the DNA present during synthesis and DNA sequence growth^4^. Compatibility with aqueous environment, pH-buffered solutions, high selectivity and reaction temperature within the limits of DNA stability have constrained the synthesis of libraries to a few reactions^5,6^. Accessibility of a wide range of building blocks, although not a must, has also influenced the selection of reactions to produce successful DELs, favouring compounds that are widely available with diverse substituents. As a consequence, most libraries rely on robust transformations, such as amide couplings, S_N_Ar reactions, reductive amination and metal-mediated cross-coupling reactions, that use broadly available building blocks—amines, carboxylic acids, aldehydes and aryl halides. Recently, efforts to explore a broader chemical space have spurred the development of new methodologies compatible with DNA-encoded conjugates, for example, Suzuki cross-coupling reactions^7^, Buchwald–Hartwig aminations^8–10^, a DNA-compatible synthesis of diazonium salts^11^, the formation of heterocycles directly on DNA^12–14^ and strain-promoted cycloadditions^15^. Other innovative methodologies, including photoredox^16–19^ and radical conjugated additions^20^, are also emerging in the field.

**Fig. 1:**
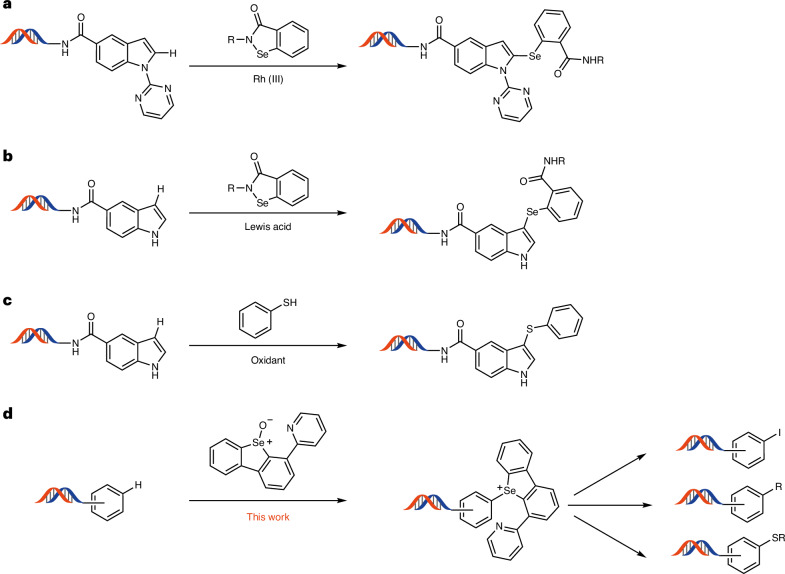
C–H functionalization on-DNA. **a**, Rhodium-mediated C2 functionalization limited to indoles with pyrimidine as directing group. **b**, Lewis-acid-mediated C3 functionalization of free N–H indoles. **c**, Oxidative thioester synthesis between electron-rich arenes and arylthiols. **d**, A general C–H functionalization of arenes by means of a synthetic linchpin, and the subsequent transformations, as a strategy to expand the accessible chemical space for DEL synthesis. Depicted DNA fragment: HP–AOP–NH–. See the Methods for more details.

Direct functionalization of C–H bonds on DNA conjugates has been scarcely explored and is limited to a few isolated examples for the introduction of a single functionality. Early examples relied on ruthenium^21^- or palladium^22^-mediated C–H activation of DNA-conjugated olefins in a Heck-type reaction. The group of Yu developed a C–H activation with cyclic carboxylic acids as directing group, for the construction of new C*sp*^2^–C*sp*^3^ bonds with DNA-conjugated aryl iodides^23^. However, none of these examples shows the functionalization of an arene’s C–H bond. In 2020, Hou and Yang reported the first C–H functionalization of conjugated arenes for library synthesis^24^. They used a rhodium mediator and a pyrimidine scaffold as a directing group to introduce benzoselenazolone fragments to the C2 position of indole derivatives (Fig. 1a). Later, the same group leveraged the nucleophilicity of indoles to achieve its functionalization on the C3 position (Fig. 1b)^25^. Both of these methodologies, although first of their kind, are limited to the use of indoles and benzoselenazolone as coupling partners.

**Fig. 2:**
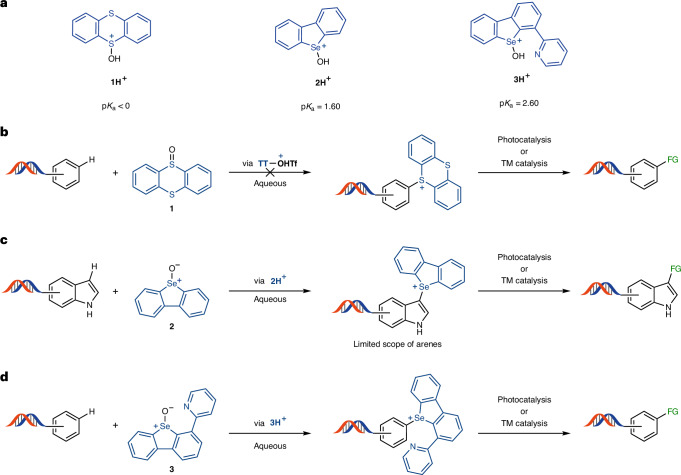
C–H functionalization in aqueous media. **a**, Different p*K*_a_ values of protonated sulfoxide and selenoxides species highlight the structural effects on oxide basicity and their activation. **b**, Thianthrenium salt formation is incompatible with aqueous media. **c**, C–H functionalization in aqueous media by activated selenoxide reagent **2H**. **d**, C–H functionalization in aqueous media by activated selenoxide reagent **3H**. TT, thianthrene; TM, transition metal; FG, functional group. Depicted DNA fragment: HP–AOP–NH–. See the Methods for more details.

Another study by Li’s group expanded the C–H functionalization to other electron-rich arenes, such as anilines^26^. The oxidative synthesis of thioethers was possible through direct coupling between the arenes and thiophenol derivatives (Fig. 1c). Li’s study is the most general C–H functionalization of DNA-conjugated arenes so far, yet the transformation is still limited to the formation of C–S and C–Se bonds with aryl thiols and selenides. Traditionally, the functionalization of arene derivatives relies on halogenation procedures, which can then participate in various bond formations^27^. However, halogenation procedures often use highly oxidative or acidic conditions that are detrimental for DNA stability^28^. In addition, high selectivity is crucial to prevent the formation of complex mixtures of constitutional isomers and false-positive hits during library screening. Therefore, halogens must be present in the corresponding building blocks before their installation on DNA. The dependency of library synthesis on the access to the corresponding building block substantially reduces the available chemical space of the resulting library. The direct, regioselective introduction of a synthetic linchpin through C–H functionalization, which could be further transformed into different functional groups, remains an unsolved challenge in the field of DELs (Fig. 1d). Thianthrenation of arenes is often a more selective alternative to halogenation^29^. The resulting arylthiantrenium salts can be used in a variety of subsequent transformations, from metal-catalysed C–C cross couplings to radical arylation reactions^30–34^. Although selective, the activation of *S*-thianthreneoxide requires anhydrous conditions, as well as highly reactive activating reagents, such as trifluoroacetic anhydride, incompatible with DNA stability (Fig. 2b). Recent discoveries in our laboratory^35^ support that the use of a selenium analogue to thianthrene provides the opportunity to perform selective C–H functionalization in the presence of water^36,37^.

We envisioned the C–H modification of electron-rich arenes through sufficiently basic selenoxides, so that they can be activated by acid sufficiently weak to be endured by DNA. The p*K*_a_ values of the corresponding protonated selenoxides, conjugated acids **1H** and **2H**, differ by as much as two orders of magnitude (Fig. 2a)^38,39^. Selenoxide-based reagent **2** not only exhibited compatibility with aqueous buffers but also demonstrated similar site selectivity to the thianthrenation methodology (Fig. 2c). Introduction of a pyridine moiety in **3** further increased the p*K*_a_ of the conjugated acid **3H** by another order of magnitude and allowed reactivity for C–H functionalization at pH 3.5. We first evaluated the reactivity of selenoxide **2** to functionalize a small set of DNA-conjugated arenes, and we observed full conversion for the most electron-rich heterocycles, such as indoles (Supplementary Information, pages 68–80). However, with less activated arenes, selenoxide **3** outperformed selenoxide **2**, presumably due to its higher basicity, increasing the scope of C–H functionalization (Fig. 2d). The different mode of activation of the selenoxide reagents simplifies the functionalization protocol compared with thianthrenation; no anhydride is necessary, and an aqueous medium in the pH range 2–4 is acidic enough for the reaction to take place. Moreover, selenoxide **3** is a bench-stable solid, soluble in water.

**Fig. 3:**
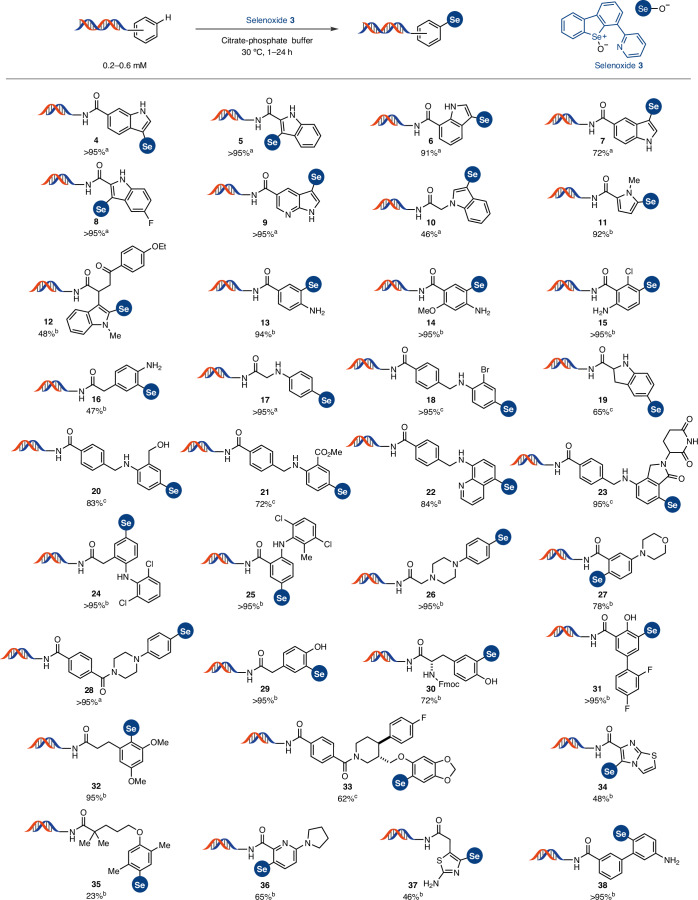
Substrate scope of selenonium salt formation. ^a^DNA conjugate (0.65 mM), selenoxide **3** (1.3 mM) in citrate-phosphate buffer (0.17 M, pH 3.5), 30 °C, 1 h. ^b^DNA conjugate (0.1–0.65 mM), selenoxide **3** (1.3–4 mM) in citrate-phosphate buffer (0.17–0.2 M, pH 3), 30 °C, 1–24 h. ^c^DNA conjugate (0.2–0.6 mM), selenoxide **3** (1.3–2 mM) in citrate-phosphate buffer (0.17–0.3 M, pH 2), 30 °C, 1 h. Depicted DNA fragment: HP–AOP–NH–. See the Supplementary Information for more details.

Next, we evaluated the scope of the linchpin installation with a wider range of DNA-conjugated arenes (Fig. 3). Electron-rich heteroarenes, such as indole (**4**–**10** and **12**) and pyrrole (**11**) derivatives, exhibited complete conversion in citrate-phosphate buffer as reaction medium within 1–16 h at 30 °C. Interestingly, unlike previously reported DEL reactions that often require a high excess of reagents, typically 40–100 equivalents^5,6^, our methodology achieved full conversion with only 2–10 molar equivalents of reagent in most cases. The functionalization of other electron-rich arenes, including primary (**13**–**16** and **38**), secondary (**17**–**25**) and tertiary anilines (**26**–**28** and **36**), proceeded smoothly, resulting in the formation of only one constitutional isomer detectable by high-performance liquid chromatography–mass spectrometry analysis. Benzylic alcohols (**20**) and anilines containing oxidation-sensitive groups are often found in medicinal chemistry, as are piperazines (**26** and **28**), morpholines (**27**) and benzylic amines (**18** and **20**–**23**). Our reaction conditions proved compatible with these scaffolds, with the exception of *N*-methylaniline (Supplementary Fig. 91), which underwent partial demethylation. Functional groups that can be further functionalized, such as bromide (**18**), chlorides (**15**, **24** and **25**), esters (**21**) and primary amines (**13**–**16** and **37**), also remained untouched. When exploring less electron-rich diarylanilines (**25**) and phenols (**29**–**31**), we found that an excess of 10–50 equivalents of selenoxide was required. Nevertheless, these substrates still exhibited yields of over 70%. Heterocyclic aniline **36** was successfully functionalized, which showcases the application of the C–H functionalization to substrates resulting from S_N_Ar, a reaction widely used in DEL synthesis. In addition, the fluorenylmethyloxycarbonyl (Fmoc) protecting group, commonly used in DEL libraries, proved to be compatible with our methodology (**30**). Electron-rich dimethoxyarenes (**32** and **33**) also underwent the desired transformation, whereas less activated anisole derivatives (Supplementary Fig. 91) remained unreactive. All tested benzene derivatives without at least one amino substituent or two alkoxides directly attached to the benzene ring failed to engage in the reaction, and no conversion was observed, with the only exception of dimethylanisole derivative **35**. Finally, we selected a representative set of arenes to perform the selenation procedure ‘off DNA’, to determine and characterize the constitutional isomer obtained upon C–H functionalization (Supplementary Information, pages 169–186). To use the C–H functionalization protocol in the production of DEL, DNA integrity is a requirement. No acidic or oxidative DNA degradation, typically by loss of nucleobases^40^, was observed under any of the reaction conditions used for the scope shown here. When the DNA conjugates were exposed to longer reaction times: pH 3.0, 96 h, and pH 2.0, 24 h, partial degradation was detected (Supplementary Information, pages 148–163). Quantitative (q)PCR has become a key tool to assess potential DNA damage in DEL methodology development^41^. We synthesized a DNA conjugate containing 87 base pairs and exposed it to the C–H functionalization conditions (Supplementary Information, pages 163–169). After the reaction, we observed a good correlation of the qPCR efficacy between the sample exposed to acid, and the control, which indicates that the DNA conjugates maintain their structural integrity and replication capabilities.

**Fig. 4:**
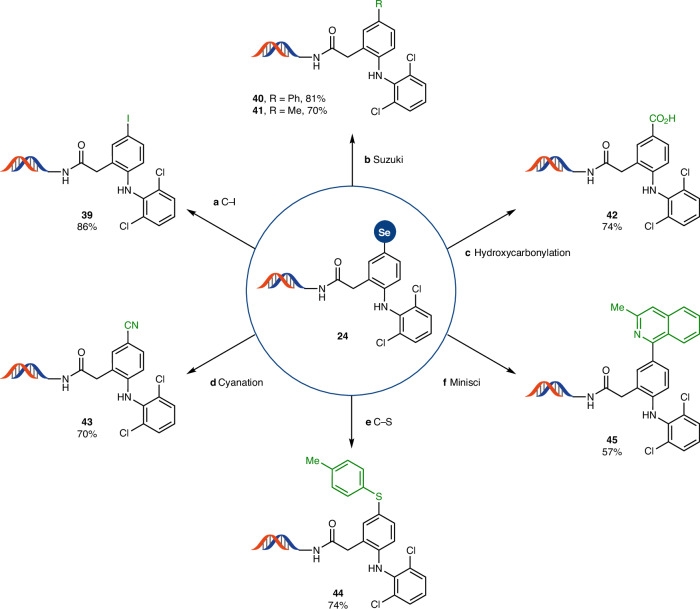
Further transformations of linchpin selenonium salt. **a**, Photochemical iodination of selenonium salt: DNA conjugate (0.3 mM), NaI (1.67 M) in NaP_i_ buffer (0.30 M, pH 6.0), Ar, 25 °C, 450-nm light-emitting diode (LED), 16 h. **b**, Suzuki cross-coupling: DNA conjugate (0.2 mM), APhos Pd G3 (4 mM), phenylboronic acid or methylboroxine (100 mM) in NaP_i_ buffer (0.25 M, pH 8.0):DMA (3:2), 95 °C, 15 min. **c**, Pd-mediated hydroxycarbonylation: DNA conjugate (0.17 mM), XantPhos Pd G3 (1.7 mM), trichlorophenyl formate (170 mM) in NaP_i_ buffer (0.17 M, pH 8.0):DMA (1:2), 80 °C, 30 min. **d**, Pd-mediated cyanation: DNA conjugate (0.2 mM), XantPhos Pd G3 (4 mM), potassium ferrocyanide (80 mM) in NaP_i_ buffer (0.2 M, pH 8.0):NMP (2:1), 95 °C, 15 min. **e**, Photochemical C–S coupling: DNA conjugate (0.6 mM), 4-methylbenzenethiol (300 mM), K_2_CO_3_ (300 mM) in DMSO:water (2:1), Ar, 25 °C, 456-nm LED, 15 min. **f**, Minisci-type arylation: DNA conjugate (0.3 mM), 1,2,2,6,6-pentamethylpiperidine (16 mM), 3-methylisoquinoline (660 mM) in DMSO:water (9:1), Ar, 25 °C, 450-nm LED, 15 min. Ar, argon; APhos, (4-(*N*,*N*-dimethylamino)phenyl)di-*tert*-butyl phosphine; XantPhos, 4,5-bis(diphenylphosphino)-9,9-dimethylxanthene. Depicted DNA fragment: HP–AOP–NH–. See the Methods for more details.

Subsequently, we assessed the versatility of the arylselenonium group as a linchpin for transition-metal-catalysed cross-coupling and radical reactions (Fig. 4). The robustness and wide availability of boronic acids makes the Suzuki cross-coupling one of the most widely used reactions in medicinal chemistry and hit identification^42^. Several metal-catalysed reactions such as Suzuki^7^ and Sonogashira^43^ couplings have been reported for DEL technology; however, these methods require a preinstalled halide or (pseudo)halide on the building block before DNA conjugation. When the arylselenonium salts were subjected to reaction conditions in the presence of a palladium catalyst and a boronic acid, we successfully achieved Suzuki cross-coupling to form C–C bonds with yields higher than 70% (**40** and **41**). Overall, the present study shows a general case of a full on-DNA two-step sequence for arene C–H bond functionalization leading to a cross-coupling product. Hydroxycarbonylation (**42**) via the use of trichlorophenyl formate as a CO surrogate^44^, and cyanation (**43**) via innocuous potassium ferrocyanide as a cyanide source, allows one to introduce a reactive functional group that could be further modified in the next step of a library synthesis^45–47^. Photoredox catalysis is a rapidly expanding field in organic and medicinal chemistry^48–50^ and often occurs at high dilution and mild temperature^51^, ideal for DEL reaction requisites. Indeed, selenonium salt **24** readily engaged in photoredox-mediated C–S bond formation (**44**) as well as Minisci-type reactions (**45**) under blue light irradiation in 15 min. Iodoarenes have been extensively exploited for the construction of DELs owing to their versatility in engaging in numerous transformations. However, to the best of our knowledge, there are no direct chemoselective monoiodination procedures for DNA-conjugated molecules. In fact, the only reported iodination of pyrroles produces mixtures of mono- and di-iodinated derivatives^52^. Conversion of the selenonium salt to the corresponding iodoarene **39** opens access to a variety of protocols already reported for DEL, such as C–N (refs. ^8–10^) or C–C (refs. ^7,17,18,43,53^) couplings without the need for further reaction development.

**Fig. 5:**
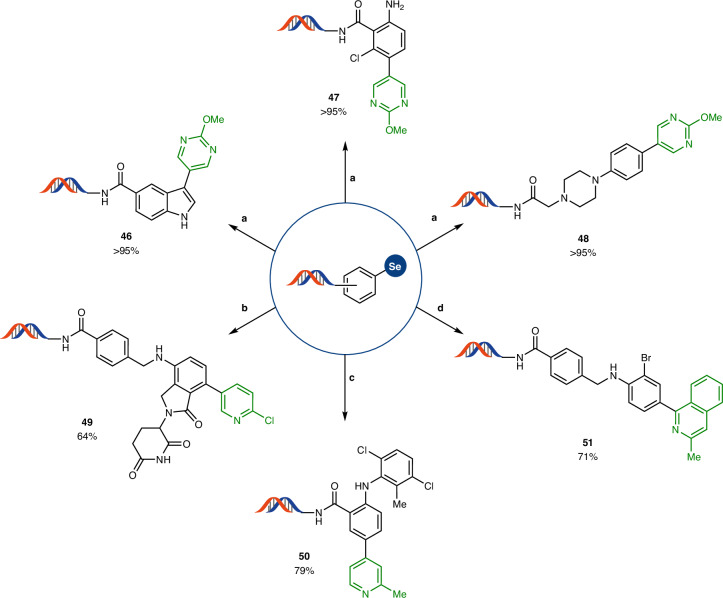
DNA-conjugated selenonium salts diversification via Suzuki cross-coupling. DNA conjugate (0.2 mM), APhos Pd G3 (4 mM), boronic acid (100 mM) in NaP_i_ buffer (0.25 M, pH 8.0):DMA (3:2), 95 °C, 15 min. **a**, 2-Methoxypyrimidine-5-boronic acid. **b**, 6-Chloro-3-pyridineboronic acid. **c**, 2-Methylpyridine-4-boronic acid, 80 °C, 10 min. **d**, DNA conjugate (0.3 mM), 1,2,2,6,6-pentamethylpiperidine (16 mM), 3-methylisoquinoline (660 mM) in DMSO:water (9:1), Ar, 25 °C, 450-nm LED, 15 min. Ar, argon; APhos, (4-(*N*,*N*-dimethylamino)phenyl)di-*tert*-butyl phosphine. Depicted DNA fragment: HP–AOP–NH–. See the Methods for more details.

To ensure the applicability of C–H functionalization in DEL synthesis, the following transformations need to be compatible with a range of different selenonium salts bearing different substitution patterns and electronic properties. Indole derivative (**46**), primary amine (**47**), tertiary aniline (**48**) and secondary anilines (**49** and **50**) all yielded the Suzuki cross-coupling products in more than 60% yield with boronic acids bearing different heterocycles (Fig. 5). We additionally tested the capability of the selenonium linchpin to be selectively activated by blue light irradiation in the presence of the labile group Br and observed C–Se bond scission exclusively (**51**). However, under palladium-mediated conditions, the same selenonium salt **18** undergoes double functionalization of both the bromide and selenonium groups.

## Conclusions

In summary, C–H functionalization of DNA conjugates was made possible by the development of a family of selenoxide reagents. Selenoxides allowed us to synthesize a range of arylselenonium salts of different electron-rich arenes, while maintaining their structural integrity and replication capabilities as confirmed by qPCR. Furthermore, the versatility of the resultant selenonium linchpin is showcased by a variety of transformations with common building blocks for DEL synthesis. Our present study represents a general chemoselective on-DNA C–H functionalization that allows the introduction of more than one functionality, including C–C, C–S and C–I bond formation.

## Methods

### DNA sequence of DNA headpiece (HP–NH_2_)

DNA headpiece HP–NH_2_ (5′-/5Phos/GAGTCA/iSp9/iUniAmM/iSp9/TGACTCCC-3′) was purchased from LGC, Biosearch Technologies.

### Reaction of DNA headpiece (HP–NH_2_) with linker Fmoc-AOP

At 20–25 °C, 100 µl of HP–NH_2_ (1.00 mM, 100 nmol, 1.00 equiv.) in borate buffer (pH 9.4, concentration (*c*) = 250 mM) was added to a 1.5-ml Eppendorf tube. Next, 10 µl of a Fmoc-15-amino-4,7,10,13-tetraoxapentadecanoic acid (Fmoc-AOP) stock solution (400 mM, 4.0 µmol, 40 equiv.) in dimethyl acetamide (DMA) was added. The mixture was vortexed for 5 s. Then, 10 µl of a 4-(4,6-dimethoxy-1,3,5-triazin-2-yl)-4-methyl-morpholinium chloride stock solution (400 mM, 4.0 µmol, 40 equiv.) in water was added. The mixture was vortexed for 5 s again, transferred into a Thermocycler at 25 °C and incubated at 25 °C for 16 h at 600 rpm. After 16 h, 12 µl of a 5 M solution of NaCl in water (10% volume of the reaction mixture) and 400 µl of ethanol at −20 °C were added to precipitate the DNA conjugate. The Eppendorf tube was placed in a freezer (−20 °C) for at least 1 h, and then it was centrifuged at 4 °C and 10,000*g* for at least 30 min. The supernatant was removed, and the pellet was redissolved in 50 µl of water to obtain an aqueous solution of HP–AOP–NHFmoc (2.00 mM, 100 nmol, 1.00 equiv.).

At 20–25 °C, to the 50 µl of HP–AOP–NHFmoc (2.00 mM, 100 nmol, 1.00 equiv.) in water was added 50 µl of a solution of piperidine in DMF (50% v/v). The mixture was vortexed for 5 s and left standing at 20–25 °C for 2 h. After 2 h, 10 µl of a 5 M solution of NaCl in water and 300 µl of ethanol at −20 °C were added to precipitate the DNA conjugate. The Eppendorf tube was placed in the freezer (−20 °C) for at least 1 h, and then it was centrifuged at 4 °C and 10,000*g* for at least 30 min. The supernatant was removed, and the pellet was redissolved in 20 µl of water. The remaining pellet was then dried under a flow of nitrogen, redissolved with 50 µl water and stored in the freezer at −20 °C to obtain HP–AOP–NH_2_ as a 2.00 mM aqueous solution.

### General procedure for Se modification of DNA conjugates

At 20–25 °C, DNA-conjugated arene (0.40–1.0 mM, final concentration 0.20–0.65 mM) in citrate-phosphate buffer (pH 2.0–3.5, *c* = 0.3–1 M, final concentration 0.16–0.30 M) was added to a 1.5-ml Eppendorf tube. Then, a stock solution of selenoxide **3** (*c* = 4.0 mM, 2–10 equiv., final concentration 1.3–2.0 mM) in water was added (see the Supplementary Information for more details). The mixture was vortexed for 5 s, transferred into a Thermocycler preheated at 30 °C and incubated at 30 °C for 1–24 h at 600 rpm.

### General procedure for palladium-mediated reactions of DNA conjugates

At 20–25 °C, DNA-conjugated selenonium salt (0.33 mM, final concentration 0.20 mM) in sodium-phosphate buffer (NaP_i_) (pH 8.0, *c* = 330 mM) was added to a 1.5-ml Eppendorf tube. Next, a stock solution of the Pd precatalyst (5–20 mM, final concentration 1.7–4 mM) in DMA or *N*-methyl-2-pyrrolidone (NMP) was added to the Eppendorf tube under air. The mixture was vortexed for 5 s. Lastly, a stock solution of the coupling partner (500 mM, final concentration 100–170 mM) in DMA or NMP was added. The mixture was vortexed for 5 s, transferred into a Thermocycler preheated at 80–95 °C and incubated at 80–95 °C for 15–30 min at 600 rpm.

### General procedure for photochemical reactions of DNA conjugates

At 20–25 °C, DNA-conjugated selenonium salt (2.0 mM, final concentration 0.33–0.66 mM) in water was added to a 1.5-ml Eppendorf tube. Next, a stock solution of base (0.10–2.0 M, final concentration 16–300 mM) in dimethyl sulfoxide (DMSO) or water was added to the Eppendorf tube under air. The mixture was vortexed for 5 s. Lastly, a stock solution of the coupling partner (0.50–1.0 M, final concentration 0.33–0.66 M) in DMSO was added. Argon was fluxed over the reaction for 30 s. The mixture was vortexed for 5 s, transferred into a Penn PhD Photoreactor M2 and irradiated at 450 nm for 15 min at 25 °C.

## Online content

Any methods, additional references, Nature Portfolio reporting summaries, source data, extended data, supplementary information, acknowledgements, peer review information; details of author contributions and competing interests; and statements of data and code availability are available at 10.1038/s41557-025-01844-6.

## Supplementary information

Supplementary Information Supplementary Figs. 1–147, discussion, detailed methods, liquid chromatography–mass spectrometry traces and characterization data.

## Data Availability

The data supporting the findings of this study are available within the Article and its Supplementary Information. Should any raw data files be needed in another format, they are available from the corresponding author upon reasonable request.
