# Supplementary material for: On-DNA C–H functionalization of electron-rich arenes for DNA-encoded libraries
Source: Nat Chem. 2025 Jun 16;17(9):1340–7. doi: 10.1038/s41557-025-01844-6 (PMC12411225; doi:10.1038/s41557-025-01844-6)
Supplement: Supplementary file 1 — Supplementary Figs. 1–147, discussion, detailed methods, liquid chromatography–mass spectrometry traces and characterization data. [file 41557_2025_1844_MOESM1_ESM.pdf]

nature chemistry

Supplementary information

https://doi.org/10.1038/s41557-025-01844-6

On-DNA C–H functionalization of electron-rich arenes for DNA-encoded libraries

In the format provided by the authors and unedited

TABLE OF CONTENTS

TABLE OF CONTENTS 1

MATERIALS AND METHODS...7

Starting Materials 7
Solvents..8
Chromatography..8
NMR Spectroscopy 8
Mass spectrometry...8
Liquid chromatography–mass spectrometry (LC–MS)..8
Gel electrophoresis of DNA conjugates 9
Quantitative polymerase chain reaction (qPCR)...9
Miscellaneous..9

EXPERIMENTAL DATA 10

Synthesis of selenoxide reagent 10

Preparation of selenide **S1** 10
Preparation of selenoxide reagent **2** 10
Preparation of selenide boronic acid **S2** 11
Preparation of selenoxide reagent **3** 12

Functionalization of DNA conjugates 13

DNA conjugate HP–AOP–NH_2 13

General procedures for the preparation of DNA-conjugated substrates.................15

General procedure for synthesis of DNA-conjugated arenes with HATU 15
General procedure for synthesis of DNA-conjugated arenes with COMU 15
General procedure for synthesis of DNA-conjugated arenes with EDC·HCl[4] 16
General procedure for synthesis of DNA-conjugated arenes with DMT-MM 16
General procedure for synthesis of DNA-conjugated arenes by reverse amidation 17
General procedure for synthesis of DNA-conjugated arenes by reductive amination ..17
General procedure for synthesis of DNA-conjugated arenes by S_NAr.....................18
General procedure for synthesis of DNA-conjugated arenes by Suzuki coupling 18

Preparation of DNA-conjugated substrates 19

DNA-conjugated arene **S5** 19
DNA-conjugated arene **S6** 20
DNA-conjugated arene **S7** 21
DNA-conjugated arene **S8** 22
DNA-conjugated arene **S9** 23
DNA-conjugated arene **S10** 24

DNA-conjugated arene **S11** 25
DNA-conjugated arene **S12** 26
DNA-conjugated arene **S13** 28
DNA-conjugated arene **S14** 29
DNA-conjugated arene **S15** 30
DNA-conjugated arene **S16** 31
DNA-conjugated arene **S17** 32
DNA-conjugated arene **S18** 33
DNA-conjugated arene **S19** 35
DNA-conjugated arene **S20** 36
DNA-conjugated arene **S21** 37
DNA-conjugated arene **S22** 38
DNA-conjugated arene **S23** 39
DNA-conjugated arene **S24** 40
DNA-conjugated arene **S25** 41
DNA-conjugated arene **S26** 42
DNA-conjugated arene **S27** 43
DNA-conjugated arene **S28** 45
DNA-conjugated arene **S29** 46
DNA-conjugated arene **S30** 47
DNA-conjugated arene **S31** 48
DNA-conjugated arene **S32** 49
DNA-conjugated arene **S33** 50
DNA-conjugated arene **S34** 51
DNA-conjugated arene **S35** 52
DNA-conjugated arene **S36** 53
DNA-conjugated arene **S37** 54
DNA-conjugated arene **S38** 55
DNA-conjugated arene **S39** 56
DNA-conjugated arene **S40** 57
DNA-conjugated arene **S41** 58
DNA-conjugated arene **S42** 59
DNA-conjugated arene **S43** 60
DNA-conjugated arene **S44** 61
DNA-conjugated arene **S45** 62

DNA-conjugated C−H functionalization with selenoxide reagent 64

General procedure for C−H functionalization of DNA-conjugated arenes at pH 3.5 64

General procedure for C−H functionalization of DNA-conjugated arenes at pH 3.0 64

General procedure for C−H functionalization of DNA conjugates with excess of Selenoxide 65

General procedure for C−H functionalization of less activated DNA conjugates 65

General procedure for C−H functionalization of DNA-conjugated arenes at pH 2.0 66

Stability of DNA-conjugated selenonium salts 66

DNA-conjugated C−H functionalization with selenoxide reagent **2** 67

Synthesis of DNA-conjugated selenonium salt **S46** 67

Synthesis of DNA-conjugated selenonium salt **S47** 68

Synthesis of DNA-conjugated selenonium salt **S48** 69

Synthesis of DNA-conjugated selenonium salt **S49** 70

Synthesis of DNA-conjugated selenonium salt **S50** 71

Synthesis of DNA-conjugated selenonium salt **S51** 72

Synthesis of DNA-conjugated selenonium salt **S52** 73

Synthesis of DNA-conjugated selenonium salt **S53** 74

Synthesis of DNA-conjugated selenonium salt **S54** 75

Synthesis of DNA-conjugated selenonium salt **S55** 76

Synthesis of DNA-conjugated selenonium salt **S56** 77

Synthesis of DNA-conjugated selenonium salt **S57** 78

Synthesis of DNA-conjugated selenonium salt **S58** 79

DNA-conjugated C−H functionalization with selenoxide reagent **3** 80

Synthesis of DNA-conjugated selenonium salt **4** 80

Synthesis of DNA-conjugated selenonium salt **5** 81

Synthesis of DNA-conjugated selenonium salt **6** 82

Synthesis of DNA-conjugated selenonium salt **7** 83

Synthesis of DNA-conjugated selenonium salt **8** 84

Synthesis of DNA-conjugated selenonium salt **9** 85

Synthesis of DNA-conjugated selenonium salt **10** 86

Synthesis of DNA-conjugated selenonium salt **11** 87

Synthesis of DNA-conjugated selenonium salt **12** 88

Synthesis of DNA-conjugated selenonium salt **13** 89

Synthesis of DNA-conjugated selenonium salt **14** 90

Synthesis of DNA-conjugated selenonium salt **15** 91

Synthesis of DNA-conjugated selenonium salt **16** 92

Synthesis of DNA-conjugated selenonium salt **17** 93

Synthesis of DNA-conjugated selenonium salt **18** 94

Synthesis of DNA-conjugated selenonium salt **19** ... 95

Synthesis of DNA-conjugated selenonium salt **20** ... 96

Synthesis of DNA-conjugated selenonium salt **21** ... 97

Synthesis of DNA-conjugated selenonium salt **22** ... 98

Synthesis of DNA-conjugated selenonium salt **23** ... 99

Synthesis of DNA-conjugated selenonium salt **24** ... 100

Synthesis of DNA-conjugated selenonium salt **25** ... 101

Synthesis of DNA-conjugated selenonium salt **26** ... 102

Synthesis of DNA-conjugated selenonium salt **27** ... 103

Synthesis of DNA-conjugated selenonium salt **28** ... 104

Synthesis of DNA-conjugated selenonium salt **29** ... 105

Synthesis of DNA-conjugated selenonium salt **30** ... 106

Synthesis of DNA-conjugated selenonium salt **31** ... 107

Synthesis of DNA-conjugated selenonium salt **32** ... 108

Synthesis of DNA-conjugated selenonium salt **33** ... 109

Synthesis of DNA-conjugated selenonium salt **34** ... 110

Synthesis of DNA-conjugated selenonium salt **35** ... 111

Synthesis of DNA-conjugated selenonium salt **36** ... 112

Synthesis of DNA-conjugated selenonium salt **37** ... 113

Synthesis of DNA-conjugated selenonium salt **38** ... 114

Failed DNA-conjugated substrates for C−H functionalization ... 115

DNA-conjugated functionalization of selenonium salts ... 115

Iodination of DNA-conjugated selenonium salt **24** ... 115

Suzuki coupling of DNA-conjugated selenonium salt **24** with phenylboronic acid ... 116

Suzuki coupling of DNA-conjugated selenonium salt **24** with trimethylboroxine ... 117

Hydroxycarbonylation of DNA-conjugated selenonium salt **24** ... 118

Cyanation of DNA-conjugated selenonium salt **24** ... 120

C–S coupling of DNA-conjugated selenonium salt **24** ... 121

Minisci reaction of DNA-conjugated selenonium salt **24** ... 122

Suzuki coupling of DNA-conjugated selenonium salt **24** with 2-methoxypyrimidine-5-boronic acid ... 123

Suzuki coupling of DNA-conjugated selenonium salt **7** with 2-methoxypyrimidine-5-boronic acid ... 124

Suzuki coupling of DNA-conjugated selenonium salt **15** with 2-methoxypyrimidine-5-boronic acid ... 125

Suzuki coupling of DNA-conjugated selenonium salt **28** with 2-methoxypyrimidine-5-boronic acid ... 126

Suzuki coupling of DNA-conjugated selenonium salt **23** with 6-chloro-3-pyridineboronic acid pinacol ester ... 127

Suzuki coupling of DNA-conjugated selenonium salt **25** with 2-methylpyridine-4-boronic acid ... 128

Minisci reaction of DNA-conjugated selenonium salt **18** with 3-methylisoquinoline.............................129

Proof-of-concept mock libraries 131

Mock library 1 – Cycle 1 131

Cycle 1: Amide coupling... 131

Cycle 1: C–H functionalization of mock library 1, cycle 1 pool.. 132

Mock library 1 – Cycle 2 134

Cycle 2: Suzuki coupling of pool of DNA-conjugated selenonium salts 1 with 2-methoxypyrimidine-5-boronic acid.. 135

Cycle 2: Suzuki coupling of pool of DNA-conjugated selenonium salts 1 with 6-chloro-3-pyridineboronic acid pinacol ester 136

Mock library 2 – Cycle 1 & 2 138

Cycle 1: Amide coupling... 138

Cycle 2: S_NAr 138

Cycle 2: C–H functionalization of mock library 2, cycle 2 pool.. 139

Mock library 2 – Cycle 3 141

Cycle 3: Suzuki coupling of pool of DNA-conjugated selenonium salts 2 with 2-methoxypyrimidine-5-boronic acid.. 141

Cycle 3: Suzuki coupling of pool of DNA-conjugated selenonium salts 2 with (4-(methylsulfonamido)phenyl)boronic acid 143

Cycle 3: Suzuki coupling of pool of DNA-conjugated selenonium salts 2 with phenylboronic acid 144

Cycle 3: Suzuki coupling of pool of DNA-conjugated selenonium salts 2 with 6-chloro-3-pyridineboronic acid pinacol ester 146

DNA stability tests.. 147

DNA stability under acidic conditions 147

C−H functionalization of DNA-conjugated arenes at pH 3.5... 147

C−H functionalization of DNA-conjugated arenes at pH 3.5 and 10 equiv. of Selenoxide 3................. 149

C−H functionalization of DNA-conjugated arenes at pH 3.. 151

C−H functionalization of DNA-conjugated arenes at pH 3.0 and 10 equiv. of Selenoxide 3................. 153

C−H functionalization of DNA-conjugated arenes at pH 3.0 and 50 equiv. of Selenoxide 3................. 155

C−H functionalization of DNA-conjugated arenes at pH 2.. 157

C−H functionalization of DNA-conjugated arenes at pH 2 and 10 equiv. of Selenoxide 3................... 160

DNA ligation of DNA-conjugated selenonium salt **24**...162

DNA ligation of DNA-conjugate **S25** 164

C−H functionalization of DNA-conjugate **S60** 165

qPCR procedure...166

“Off-DNA” preparation of selenonium salts... 168

Preparation of selenonium salt **S61** 168
Preparation of selenonium salt **S62** 169
Preparation of selenonium salt **S63** 170
Preparation of selenonium salt **S64** 170
Preparation of selenonium salt **S65** 171
Preparation of selenonium salt **S66** 172
Preparation of selenonium salt **S67** 172
Preparation of selenonium salt **S68** 173
Preparation of selenonium salt **S69** 174
Preparation of selenonium salt **S70** 175
Preparation of selenonium salt **S71** 175

"Off-DNA" functionalization of selenonium salts 176

Preparation of **S72**....................176
Preparation of selenonium salt **S73** 177
"Off-DNA" Iodination of selenonium salt (**S74**)....................178
"Off-DNA" Palladium mediated coupling of selenonium salt (**S75**) 178
"Off-DNA" C-S coupling of selenonium salt (**S76**)....................179

Synthesis of DNA-conjugated selenonium salts by amide coupling.................... 180

Synthesis of DNA-conjugated selenonium salt **11** by amide coupling....................180
Synthesis of DNA-conjugated selenonium salt **15** by amide coupling....................182
Synthesis of DNA-conjugated selenonium salt **25** by amide coupling....................184

NMR DATA....................186

NMR-Characterization of "off DNA" selenonium salts.................... 186

NMR-Characterization of "off DNA" following transformations.................... 246

REFERENCES 252

MATERIALS AND METHODS

Starting Materials

All substrates were used as received from the commercial suppliers:

Material	Vendor	Purity
N,N-Dimethylacetamide (DMA)	Iris Biotech	>99%
Fmoc-L-Tyr-OH	Iris Biotech	99%
Magnesium sulfate	Fisher Scientific	99%
Piperidine	Iris Biotech	>99%
Sodium chloride	Chemsolute	>99%
Triethylamine (TEA) puriss. p.a., ≥99.5% (GC)	Sigma-Aldrich	>99.5%
Hexafluoroisopropanol (HFIP)	Fluorochem	>99.5%
N-[(Dimethylamino)-1*H*-1,2,3-triazolo-[4,5-*b*]pyridin-1-ylmethylene]-*N*-methylmethanaminium hexafluorophosphate *N*-oxide (HATU)	Sigma-Aldrich	97%
N,N-Diisopropylethylamine (DIPEA)	Sigma-Aldrich	>99%
4-(4,6-Dimethoxy-1,3,5-triazin-2-yl)-4-methylmorpholinium chloride (DMT-MM)	Sigma-Aldrich	97%
Dimethyl sulfoxide (DMSO)	Iris Biotech	99%
N-Methyl-2-pyrrolidone (NMP)	Iris Biotech	99%
(1-Cyano-2-ethoxy-2-oxoethylidenaminooxy)dimethylamino-morpholino-carbenium hexafluorophosphate (COMU)	Iris Biotech	97%
Ethidium bromide	Sigma-Aldrich	n/a
Agarose	Bio-Rad	>99.5%
Citric acid	Sigma-Aldrich	>99.5%
APhos Pd G3	Sigma-Aldrich	97%
XanthPhos Pd G3	Sigma-Aldrich	97%
Sodium bicarbonate	Acros	99%
Sodium carbonate	Acros	99%
Sodium diethyldithiocarbamate trihydrate (30.5–32.5 wt.%, Na as Na_2SO_4)	Sigma-Aldrich	n/a
Sodium sulfate (anhydrous)	VWR	98.5–101.0%
Tetrafluoroboric acid diethyl ether complex (51–57 wt.% HBF_4)	Sigma-Aldrich	n/a
TPPTS (Triphenylphosphine-3,3′,3″-trisulfonic acid trisodium salt)	Sigma-Aldrich	≥95%
Palladium acetate	In-house	98%
Fmoc-15-amino-4,7,10,13-tetraoxapentadecanoic acid (Fmoc-AOP)	BroadPharm	99%
Sodium tetrafluoroborate	ThermoScientific	>97%
1-Hydroxy-7-azabenzotriazole (HOAt)	ThermoScientific	98%
1-Ethyl-3-(3-dimethylaminopropyl)carbodiimide hydrochloride (EDC·HCl)	Sigma-Aldrich	n/a
N-Methylmorpholine (NMM)	Sigma-Aldrich	99%

DNA headpiece HP–NH_2 (5’- /5Phos/GAGTCA/iSp9/iUniAmM/iSp9/TGACTCCC-3’) was purchased from LGC, Biosearch Technologies.

DNA for ligation including Primer–Tag1–Tag2–Tag3–Tag4–ClosingPrimer (5’-/5Phos/AAATCGATGTGTTC CGCAAGAAGCCTGGTAAGCGGAGAAAGGTCGTTACGATGCCCGGTCTACNNNNNNNNNNNNNCTGAT GGCGCGAGGGAGGC/GTAGACCGGGCATCGTAACGACCTTTCTCCGCTTACCAGGCTTCTTGCGGA ACACATCGATTTGG-3’) was purchased from Integrated DNA Technologies, Inc.

T4 ligase and 10X ligation buffer were purchased from New England BioLabs Inc.

Solvents

Water used to prepare buffers and as solvent was of ultra-high quality (UHQ) grade (18.2 MΩ·cm^{-1}). Methanol (>99%) was purchased from Sigma-Aldrich, dichloromethane (>99%) and acetonitrile (>99%) were purchased from Fisher Scientific.

Chromatography

Thin layer chromatography (TLC) was performed using EMD TLC plates pre-coated with 250 µm thickness silica gel 60 F254 plates and visualized by fluorescence quenching under UV light and $KMnO_4$ stain. Flash column chromatography was performed using silica gel (40–63 µm particle size) purchased from Geduran®.

NMR Spectroscopy

Chemical shifts are reported in ppm with the solvent residual peak as the internal standard. For ^1H NMR: $CDCl_3$, δ 7.26; D_2O, δ 4.79; CD_3OD, δ 3.31; $(CD_3)_2SO$, δ 2.50; CD_3CN, δ 1.94. For ^{13}C NMR: $CDCl_3$, δ 77.2; CD_3OD, δ 41.0; $(CD_3)_2SO$, δ 39.5; CD_3CN, δ 1.3[1]. ^{19}F NMR spectra were referenced using a unified chemical shift scale based on the ^1H resonance of tetramethylsilane (1% v/v solution in the respective solvent). Data are reported as follows: s = singlet, d = doublet, t = triplet, q = quartet, quint = quintet, m = multiplet, br = broad; coupling constants in Hz.

NMR spectra of small molecules were recorded on the following instruments:

1. Bruker Avance III 500 spectrometer equipped with a BBFO probe head, operating at 500 MHz, 471 MHz, and 126 MHz, for ^1H, ^{19}F, and ^{13}C acquisitions, respectively.
2. Bruker Avance III 600 spectrometer equipped with a triple-channel "TCI" cryogenic probehead (Bruker GmbH, Rheinstetten) operating at 600 MHz, 565 MHz, 151 MHz, and 115 MHz for ^1H, ^{19}F, ^{13}C, and ^{77}Se acquisitions, respectively. All experiments used standard Bruker pulse sequences with standard parameter sets found in libraries of Topspin 3.6.

Mass spectrometry

High resolution Mass Spectrometry (HRMS) experiments for small molecules were performed on a Thermo Scientific™ Q Exactive Plus or a Thermo Scientific™ Q Exactive GC Orbitrap device.

Liquid chromatography–mass spectrometry (LC–MS)

Analytical LC–MS measurements of DNA conjugates were performed on an Agilent 1290 Infinity II system utilizing an AdvanceBio Oligonucleotide column, 50 x 4.6 mm, 2.7 µm, at 50 °C, and a flow rate = 0.4 mL · min^{-1}. The following HPLC methods were used for all DNA measurements with this setup.

- HPLC Method A: linear gradient from 10:90 v/v (MeOH : 100 mM HFIP, 10 mM TEA in H_2O) to 50:50 v/v (MeOH : 100 mM HFIP, 10 mM TEA in H_2O) over 4 min, followed by isocratic run for 5 minutes with 50:50 v/v (MeOH : 100 mM HFIP, 10 mM TEA in H_2O) followed by a gradient from 50:50 v/v (MeOH : 100 mM HFIP, 10 mM TEA in H_2O) to 10:90 v/v (MeOH : 100 mM HFIP, 10 mM TEA in H_2O) over 1 minute.
- HPLC Method B: linear gradient from 10:90 v/v (MeOH : 100 mM HFIP, 10 mM TEA in H_2O) to

50:50 v/v (MeOH : 100 mM HFIP, 10 mM TEA in H_2O) over 4 min, followed by isocratic run for 1 minute with 50:50 v/v (MeOH : 100 mM HFIP, 10 mM TEA in H_2O) followed by a gradient from 50:50 v/v (MeOH : 100 mM HFIP, 10 mM TEA in H_2O) to 10:90 v/v (MeOH : 100 mM HFIP, 10 mM TEA in H_2O) over 1 minute.

Unless otherwise stated, conversions of the DNA conjugates were calculated by LC–MS by integration of the diode array detection (DAD) UV absorbance at 260 nm, assuming complete DNA recovery and similar UV absorbance for all DNA conjugates[2]. All signals in the total ion current (TIC) chromatogram that originate from DNA conjugates (molecular weight > 1000 g/mol) were considered. Typically observable signals in TIC chromatogram include, $[M-H_4]^{4-}$, $[M-H_5]^{5-}$, $[M-H_6]^{6-}$, $[M-H_7]^{7-}$, $[M-H_8]^{8-}$, and $[M-H_9]^{9-}$. In the cases were a small molecule impurity (molecular weight < 1000 g/mol) overlaps with DNA conjugates, conversion was calculated by integration of TIC chromatogram. Impurities present prior to the analyzed reaction were not considered.

Gel electrophoresis of DNA conjugates

Gel electrophoresis was carried out as instructed in the manual of Bio-Rad utilizing the Bio-Rad Laboratories ChemiDocMP system[3]. Image Lab Version 6.1.0 build 7 software from Bio-Rad Laboratories Inc. was used for analysis and processing of gel electrophoresis data.

Reagents used in the DNA Gel electrophoresis workflow:

Bio-Rad Laboratories 10x TBE Buffer (Tris/boric acid/EDTA).

Bio-Rad Laboratories Certified low range Ultra Agarose.

Bio-Rad Laboratories EZ Load 20 base pairs (bp) Molecular Ruler.

Bio-Rad Laboratories Nucleic Acid sample loading buffer, 5x.

CarlRoth SYBR® Green DNA dye, 11x conc., for electrophoresis, ready-to-use.

Devices used in the DNA Gel electrophoresis workflow:

Bio-Rad Laboratories ChemiDocMP.

Bio-Rad Laboratories Mini-Sub® Cell GT.

Bio-Rad Laboratories PowerPac Basic considered.

Quantitative polymerase chain reaction (qPCR)

Quantitive PCR was performed on a qPCR cycler MyiQTM Optics Module-Thermocycler from Bio-Rad Laboratories. PCR-Mastermix was prepared using KAPA SYBR® FASTqPCR Master Mix.

Primers for qPCR:

- Forward primer (5' 565 Cla Primer): 5'-TGA CTC CCA AAT CGA TGT G -3'
- Reverse primer (3' 454 Short Primer): 5'-GCC TCC CTC GCG CCA -3'

Miscellaneous

DNA-conjugated reactions were conducted in an Eppendorf ThermoMixer® C. DNA desalting and rebuffering was performed with AMICON® filter units from Sigma Aldrich. DNA concentrations were determined by A_{260} absorption using a Thermo Scientific™ NanoDrop™ One^{C}.

EXPERIMENTAL DATA

Synthesis of selenoxide reagent

Preparation of selenide S1

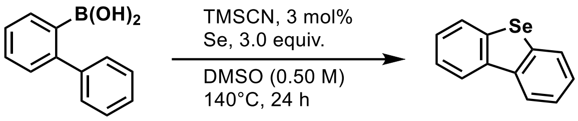

S1, 74%

Under ambient atmosphere, an oven-dried pressure vessel (50 mL) equipped with a Teflon-coated magnetic stirring bar was charged with 2-biphenylboronic acid (1.98 g, 10.0 mmol, 1.00 equiv.), selenium powder (2.37 g, 30.0 mmol, 3.00 equiv.), TMSCN (30 mg, 38 µL, 0.30 mmol, 3.0 mol%), and DMSO (20 mL, c = 0.50 M). The reaction vessel was closed by a Teflon nozzle head and heated at 140 °C for 24 h. After cooling to 25 °C, the resulting mixture was diluted with 10 mL of diethyl ether, filtered through a pad of celite (approx. 15 g), eluting with additional diethyl ether (3 × ca. 30 mL). The filtrate was combined and washed with brine (3 × ca. 100 mL). The organic layer was dried over anhydrous Na_2SO_4, filtered, and the solvent evaporated under reduced pressure. The residue was purified by chromatography on silica gel eluting with hexanes to afford the desired compound as a colorless solid (1.70 g, 7.40 mmol, 74%).

R*f* = 0.30 (silica gel, hexanes).

NMR Spectroscopy:

> **^{1}H NMR** (600 MHz, $CDCl_3$, 298 K, δ): 8.14 (ddd, *J* = 7.9, 1.4, 0.6 Hz, 2H), 7.89 (ddd, *J* = 7.9, 1.2, 0.6 Hz, 2H), 7.47 (ddd, *J* = 7.9, 7.2, 1.1 Hz, 2H), 7.40 (ddd, *J* = 7.9, 7.2, 1.3 Hz, 2H).
>
> **^{13}C NMR** (126 MHz, $CDCl_3$, 298 K, δ): ^{13}C NMR (151 MHz, $CDCl_3$) δ 139.4, 138.4, 127.0, 126.2, 125.0, 123.0.
>
> **^{77}Se NMR** (95 MHz, $CDCl_3$, 298 K, δ): 450.5.

HRMS ESI (m/z) calculated for $C_{12}H_8Se$ [M+H$^+$] 231.9786; found 231.9783; deviation: +1.3 ppm.

Preparation of selenoxide reagent 2

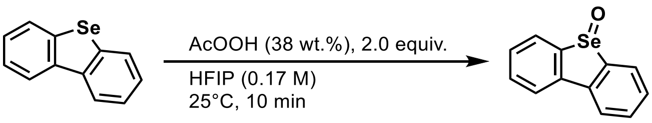

S1 **2**, 86%

Under ambient atmosphere, a 20 mL vial equipped with a teflon-coated magnetic stirring bar was charged with selenide **S1** (1.40 g, 6.06 mmol, 1.00 equiv.) and HFIP (30 mL, c = 0.20 M). The mixture was stirred at 25 °C for 2 min. Then, peracetic acid (38 wt.%, 2.56 g, 2.26 mL, 12 mmol, 2.0 equiv.) was diluted by HFIP (6.0 mL, final concentration 0.17 M) and added to the mixture at 25 °C over 5 minutes, resulting in a color change of the solution to deep blue, and finally to pale yellow. The mixture was stirred for another 10 min at 25 °C and subsequently transferred to a separatory funnel, washed with saturated aqueous Na_2CO_3 solution (40 mL) and extracted by chloroform (4 × ca. 40 mL). The organic layers were combined, dried over anhydrous Na_2SO_4, filtered, and the solvent evaporated under reduced pressure. The residue was purified by column chromatography on silica gel eluting with DCM/MeOH (10:1, v/v) to

afford the product as colorless solid (1.28 g, 5.20 mmol, 86%).

R*f* = 0.30 (silica gel, DCM/MeOH = 10/1).

NMR Spectroscopy:

^{1}H NMR (600 MHz, DMSO, 298 K, δ): 8.12 (ddd, *J* = 7.8, 1.2, 0.5 Hz, 1H), 8.04 (ddd, *J* = 7.6, 1.2, 0.5 Hz, 1H), 7.67 (td, *J* = 7.6, 1.2 Hz, 1H), 7.53 (td, *J* = 7.5, 1.2 Hz, 1H).

^{13}C NMR (151 MHz, DMSO, 298 K, δ): 146.9, 140.4, 132.0, 129.6, 129.0, 123.4.

^{77}Se NMR (115 MHz, DMSO, 298 K, δ): 939.2.

HRMS ESI (m/z) calculated for $C_{12}H_8OSeNa$ [M+Na]$^+$ 270.9633; found 270.9630; deviation: +1.1 ppm.

Preparation of selenide S3

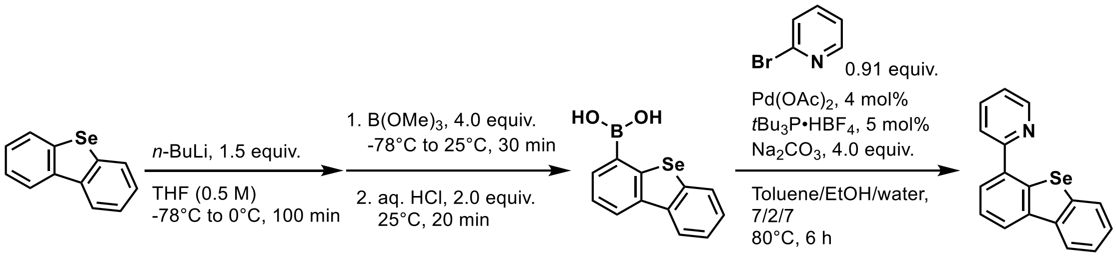

S1 **S2** **S3**, 58% *over 3 steps*

Under argon atmosphere, an 250 mL oven-dried two-neck round-bottom flask equipped with a teflon-coated magnetic stirring bar was charged with selenide **S1** (1.10 g, 4.76 mmol, 1.00 equiv.) and absolute THF (9.5 mL, c = 0.50 M). The mixture was stirred at 25 °C until all solids dissolved. Then, the flask was cooled down to –78 °C, followed by the addition of *n*-BuLi (2.5 M in pentane, 2.9 mL, 7.2 mmol, 1.5 equiv.). The resulting pale yellow solution was stirred at –78 °C for 10 min and was allowed to warm to 0 °C and stirred at 0 °C for 1.5 h, leading to the color change of the solution to bright orange. At this point, the flask was cooled down to –78 °C again, followed by the addition of $B(OMe)_3$ (2.0 g, 2.2 mL, 19 mmol, 4.0 equiv.). After the addition, the reaction was allowed to warm to 25 °C and stirred at 25 °C for 30 min. Subsequently, 20 mL of hydrochloric acid (2.0 M, 4.8 mmol, 2.0 equiv.) was added to work up the reaction, and the mixture was stirred at 25 °C for 20 min. Then, the flask was kept at ambient temperature (20–25 °C) by a cold-water bath, and 12 mL of an aqueous solution of NaOH (2 M) was slowly introduced (ca. over 30 s) to the flask. The mixture was stirred at 25 °C for 5 min and transferred to a separatory funnel. The aqueous layer was washed by Et_2O (ca. 30 mL) and the organic layer was extracted by NaOH (2 M, 2 × ca. 12 mL). The aqueous layers were combined and acidified to pH 1 by concentrated hydrochloric acid (37 wt.%) at 0 °C (detected by general pH test paper). The resulting suspension was filtrated, and the residue was washed by cold water (3 × ca. 5 mL). The collected residue was dried in vacuo to afford the desired compound as a colorless solid **S2** (953 mg, 3.47 mmol, 73%). The resulting solid was used for the next step without further purification.

Solvent preparation: A flask (100 mL) was charged with toluene (10 mL), deionized water (10 mL), and EtOH (3 mL) under ambient atomosphere. An argon flow was gently passed through the solution via a needle (Φ 0.80 × 120 mm) for 5 min. Then, the mixture was immediately used for the reaction.

Reaction set-up: Under argon atmosphere, a two-neck round-bottom flask (100 mL) equipped with a reflux condenser and Teflon-coated magnetic stirring bar was charged with the boronic acid **S2** (914 mg,

3.32 mmol, 1.00 equiv.), the coupling partner 2-bromo-pyridine (477 mg, 288 µL, 3.02 mmol, 0.91 equiv.), the catalyst $Pd(OAc)_2$ (29 mg, 0.13 mmol, 4.0 mol%), the ligand $tBu_3P{\cdot}HBF_4$ (46 mg, 0.16 mmol, 5.0 mol%), the base Na_2CO_3 (1.41 g, 13.3 mmol, 4.00 equiv.), and the mixture of degassed solvents (27 mL, c = 0.12 M). Then, the mixture was refluxed under argon atmosphere at 80 °C for 6 h. After cooling to 25 °C, the resulting mixture was transferred to a separatory funnel and washed by brine (ca. 50 mL) and the aqueous layer was extracted by EtOAc (3 × ca. 20 mL). The organic layers were combined, dried over anhydrous Na_2SO_4, filtered, and concentrated under reduced pressure. The residue was purified by the chromatography on silica gel eluting with hexanes/EtOAc (gradient from 50:1 to 20:1 v/v) to afford the desired compound **S3** as a beige solid (784 mg, 2.54 mmol, 77%).

Overall yield 4 steps: 56%

NMR Spectroscopy:

^{1}H NMR (600 MHz, $CDCl_3$, 298 K, δ): 8.87 (ddd, *J* = 4.8, 1.8, 1.0 Hz, 1H), 8.25 (dd, *J* = 7.7, 1.1 Hz, 1H), 8.22 – 8.18 (m, 1H), 8.08 (ddd, *J* = 7.6, 4.2, 1.1 Hz, 2H), 7.98 (ddd, *J* = 7.6, 1.3, 0.6 Hz, 1H), 7.84 (ddd, *J* = 8.2, 7.4, 1.8 Hz, 1H), 7.62 (t, *J* = 7.7 Hz, 1H), 7.47 (ddd, *J* = 7.9, 7.1, 1.3 Hz, 1H), 7.42 (td, *J* = 7.4, 1.3 Hz, 1H), 7.30 (ddd, *J* = 7.4, 4.8, 1.0 Hz, 1H).

^{13}C NMR (151 MHz, $CDCl_3$, 298 K, δ): 155.1, 147.8, 143.8, 140.3, 137.6, 137.5, 136.8, 134.5, 126.9, 125.6, 125.2, 124.6, 124.2, 123.5, 122.6, 122.2, 120.1.

^{77}Se NMR (115 MHz, $CDCl_3$, 298 K, δ): 490.7.

HRMS EI (m/z) calculated for $C_{17}H_{11}NSe$ [M^+] 309.0056; found 309.0051; deviation: +1.6 ppm.

Preparation of selenoxide reagent 3

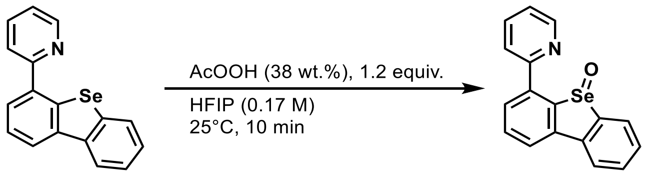

S3 **3**, 93%

Under ambient atmosphere, a 20 mL scintillation vial equipped with a teflon-coated magnetic stirring bar was charged with the selenide **S3** (648 mg, 2.10 mmol, 1.00 equiv.) and the solvent HFIP (10.5 mL, c = 0.17 M). The mixture was stirred at 25 °C for 2 min. Then, peracetic acid (38 wt.%, 505 mg, 447 µL, 2.5 mmol, 1.2 equiv.) was diluted by HFIP (1.7 mL, final concentration 0.17 M) and added to the mixture at 25 °C, resulting in the color change of the solution to deep blue, and finally to bright yellow. The mixture was stirred for another 5 min at 25 °C and subsequently transferred to a separatory funnel, washed with saturated aqueous Na_2CO_3 solution (ca. 20 mL) and extracted by chloroform (4 × ca. 20 mL). The organic layers were combined, dried over anhydrous Na_2SO_4, filtered, and the solvent evaporated under reduced pressure. The residue was purified by the column chromatography on silica gel eluting with DCM/MeOH (gradient from 50:1 to 20:1 v/v) to afford the desired compound as a colorless solid (603 mg, 1.95 mmol, 93%).

R_f = 0.28 (silica gel, DCM/MeOH = 10/1).

NMR Spectroscopy:

^{1}H NMR (600 MHz, DMSO, 298 K, δ): 8.79 (ddd, *J* = 4.7, 1.8, 0.9 Hz, 1H), 8.31 – 8.26 (m, 1H), 8.21

(ddd, J = 10.5, 7.9, 1.0 Hz, 2H), 8.14 (ddd, J = 7.9, 1.1, 0.5 Hz, 1H), 8.04 (ddd, J = 8.0, 7.5, 1.8 Hz, 1H), 7.96 (ddd, J = 7.5, 1.2, 0.6 Hz, 1H), 7.82 (t, J = 7.7 Hz, 1H), 7.68 – 7.62 (m, 1H), 7.58 – 7.49 (m, 2H).

^{13}C NMR (151 MHz, DMSO, 298 K, δ): 151.8, 148.4, 148.1, 142.9, 142.6, 139.0, 138.2, 137.5, 133.0, 131.3, 129.7, 128.3, 126.7, 124.1, 123.9, 123.2, 120.9.

^{77}Se NMR (151 MHz, DMSO, 298 K, δ): 953.5.

HRMS ESI (m/z) calculated for $C_{17}H_{11}NOSeNa$ [M+Na$^+$] 347.9898; found 347.9895; deviation: +0.8 ppm.

Functionalization of DNA conjugates

DNA conjugate HP–AOP–NH$_2$

The linker-elongated-headpiece **HP–AOP–NH$_2$** was prepared following the following procedure:

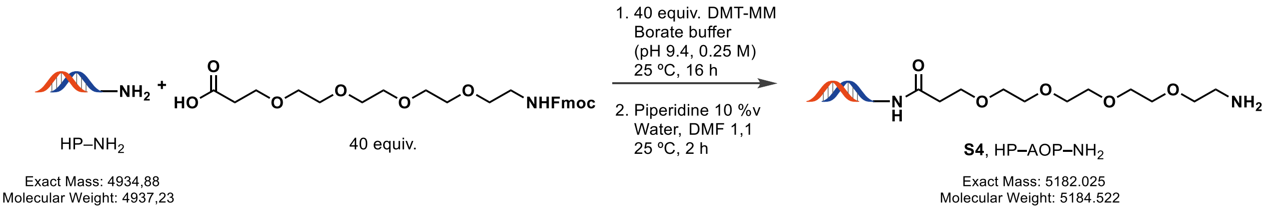

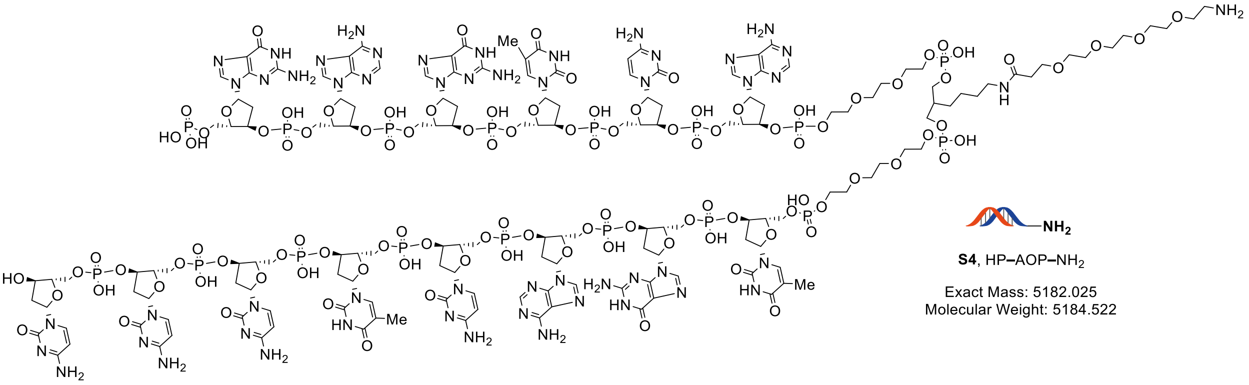

At 20–25 °C, 100 µL of HP–NH$_2$ (1.00 mM, 100 nmol, 1.00 equiv.) in borate buffer (pH 9.4, c = 250 mM) was added to a 1.5 mL Eppendorf tube. Next, 10 µL of a Fmoc-15-amino-4,7,10,13-tetraoxapentadecanoic acid (Fmoc-AOP) stock solution (400 mM, 4.0 µmol, 40 equiv.) in DMA was added. The mixture was vortexed for 5 seconds. Then, a 10 µL of a DMT-MM stock solution (400 mM, 4.0 µmol, 40 equiv.) in water was added. The mixture was vortexed for 5 seconds again, transferred into a Thermocycler at 25 °C, and incubated at 25 °C for 16 hours at 600 rpm. After 16 hours, an aliquot of 1 µL of the reaction mixture was diluted to 40 µL with water for LC–MS analysis.

Next, 12 µL of a 5 M solution of NaCl in water (10% volume of the reaction mixture) and 400 µL of ethanol at −20 °C were added to precipitate the DNA conjugate. The Eppendorf tube was placed in a freezer (−20 °C) for at least 1 hour, and then it was centrifuged at 4 °C and 10000 x g for at least 30 minutes. The supernatant was removed, the pellet was redissolved in 50 µL of water to obtain an aqueous solution of HP–AOP–NHFmoc (2.00 mM, 100 nmol, 1.00 equiv.).

At 20–25 °C, over the 50 µL of HP–AOP–NHFmoc (2.00 mM, 100 nmol, 1.00 equiv.) in water were added 50 µL of a solution of piperidine in DMF (50% v.). The mixture was vortexed for 5 seconds, and left standing at 20–25 °C for 2 hours.

After 2 hours, 10 µL of a 5 M solution of NaCl in water and 300 µL of ethanol at −20 °C were added to precipitate the DNA conjugate. The Eppendorf tube was placed in the freezer (−20 °C) for at least 1 hour, and then it was centrifuged at 4 °C and 10000 x g for at least 30 minutes. The supernatant was removed, the pellet was redissolved in 20 µL of water. The remaining pellet was then dried under a flow of nitrogen, redissolved with water and stored in the freezer at −20 °C.

An unknown impurity, detected in the synthesis of DNA conjugate **HP–AOP–NH_2 S4** by LC–MS analysis with a retention time of (2.981 min, and 3.246 min) and a molecular weight of (4978, and 5076) respectively, was present depending on the batch of commercial HP–NH_2. The impurity remained unreactive in all subsequent reactions performed during this study and was excluded from the conversion and yield calculations for the following transformations.

Two unknown impurities were detected during the synthesis of DNA conjugate **HP–AOP–NH_2 S4** by LC–MS analysis, with retention times of 2.981 min and 3.246 min, and molecular weights of 4978 and 5076, respectively. Both impurities varied depending on the batch of commercial HP–NH_2 used. They remained unreactive in all subsequent reactions throughout the study and were excluded from conversion and yield calculations for the transformations that followed.

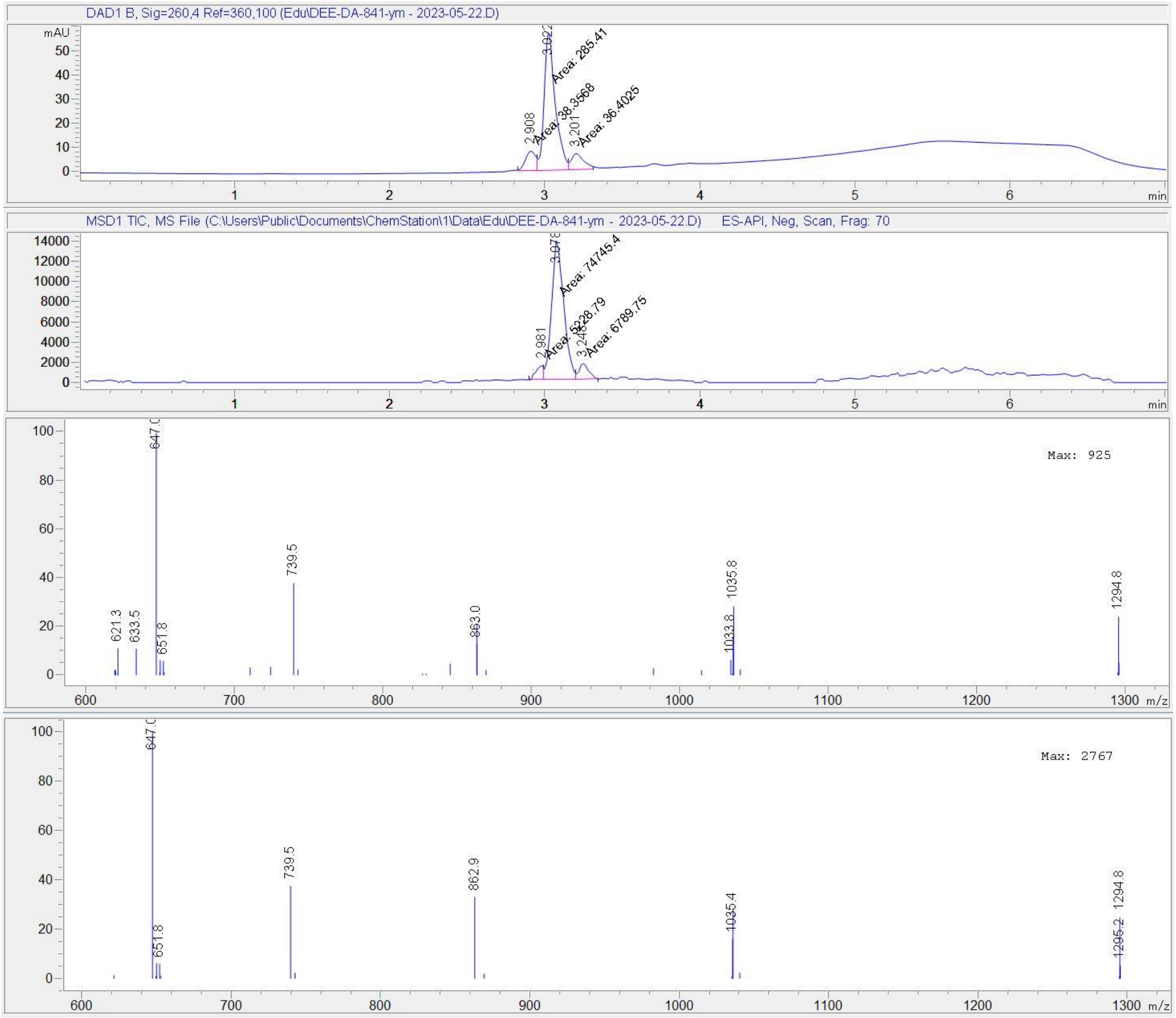

Figure S1. Analytical HPLC trace of **S4** with HPLC Method B. (Up) DAD chromatogram at 260 nm. (Middle up) TIC chromatogram. (Middle down) Ionization of all the chromatogram. (Below) Ionization of peak at 3.078 min containing reaction product **S4**.

General procedures for the preparation of DNA-conjugated substrates

General procedure for synthesis of DNA-conjugated arenes with HATU

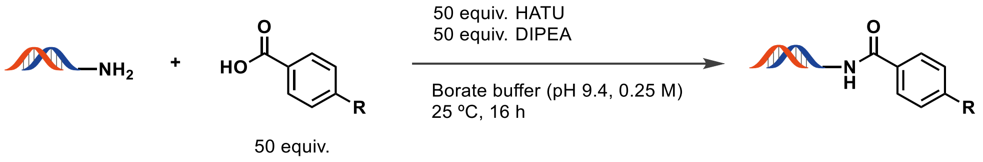

At 20–25 °C, 2.0 µL of a carboxylic acid stock solution (500 mM, 1.0 µmol, 50 equiv.) in DMA was mixed with 2.0 µL of a HATU stock solution in DMA (500 mM, 1.0 µmol, 50 equiv.) in a 1.5 mL Eppendorf tube. The mixture was vortexed for 5 seconds. Next, 2.0 µL of a DIPEA stock solution (500 mM, 1.0 µmol, 50 equiv.) in DMA was added. The mixture was vortexed for 5 seconds again, and left standing at 20–25 °C for 20 min. In another 1.5 mL Eppendorf tube, 20 µL of HP–AOP–NH_2 (1.0 mM, 20 nmol, 1.0 equiv.) in borate buffer (pH 9.4, c = 250 mM) was added, and the premix of acid, HATU and DIPEA was added over the solution. The mixture was vortexed for 5 seconds, transferred into a Thermocycler at 25 °C, and incubated at 25 °C for 16 hours at 600 rpm. After 16 hours, an aliquot of 1 µL of the reaction mixture was diluted to 40 µL with water for LC–MS analysis.

Next, 2.6 µL of a 5 M solution of NaCl in water and 80 µL of ethanol at −20 °C were added to precipitate the DNA conjugate. The Eppendorf tube was placed in the freezer (−20 °C) for at least 1 hour, and then it was centrifuged at 4 °C and 10000 x g for at least 30 minutes. The supernatant was removed, the pellet was redissolved in 26 µL of water, and the procedure was repeated again. The remaining pellet was then dried under a flow of nitrogen, redissolved with 10 µL of water and stored in the freezer at −20 °C.

General procedure for synthesis of DNA-conjugated arenes with COMU

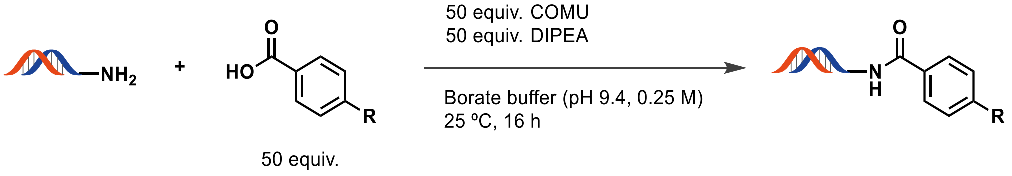

At 20–25 °C, 2.0 µL of a carboxylic acid stock solution (500 mM, 1.0 µmol, 50 equiv.) in DMA was mixed with 2.0 µL of a COMU stock solution in DMA (500 mM, 1.0 µmol, 50 equiv.) in a 1.5 mL Eppendorf tube. The mixture was vortexed for 5 seconds. Next, 2.0 µL of a DIPEA stock solution (500 mM, 1.0 µmol, 50 equiv.) in DMA was added. The mixture was vortexed for 5 seconds again, and left standing at 20–25 °C for 20 min. In another 1.5 mL Eppendorf tube, 20 µL of HP–AOP–NH_2 (1.0 mM, 20 nmol, 1.0 equiv.) in borate buffer (pH 9.4, c = 250 mM) was added, and the premix of acid, COMU and DIPEA was added over the solution. The mixture was vortexed for 5 seconds, transferred into a Thermocycler at 25 °C, and incubated at 25 °C for 16 hours at 600 rpm. After 16 hours, an aliquot of 1 µL of the reaction mixture was diluted to 40 µL with water for LC–MS analysis.

Next, 2.6 µL of a 5 M solution of NaCl in water and 80 µL of ethanol at −20 °C were added to precipitate the DNA conjugate. The Eppendorf tube was placed in the freezer (−20 °C) for at least 1 hour, and then it was centrifuged at 4 °C and 10000 x g for at least 30 minutes. The supernatant was removed, the pellet was redissolved in 26 µL of water, and the procedure was repeated again. The remaining pellet was then dried under a flow of nitrogen, redissolved with 10 µL of water and stored in the freezer at −20 °C.

General procedure for synthesis of DNA-conjugated arenes with EDC·HCl[4]

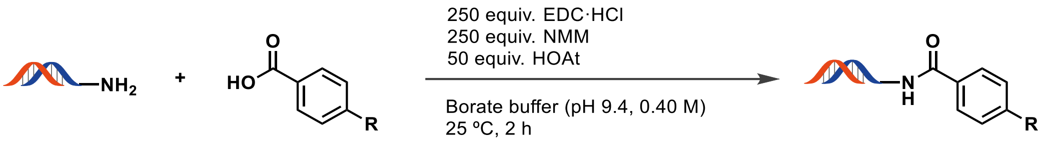

500 equiv.

At 20–25 °C, 20 µL of a carboxylic acid stock solution (500 mM, 10 µmol, 500 equiv.) in DMSO was mixed with 10 µL of a HOAt stock solution in DMSO (100 mM, 1.0 µmol, 50 equiv.), and 10 µL of an EDC·HCl stock solution in DMSO (500 mM, 5.0 µmol, 250 equiv.) in a 1.5 mL Eppendorf tube. The mixture was vortexed for 5 seconds. Next, 10 µL of a NMM stock solution (500 mM, 5.0 µmol, 250 equiv.) in DMSO was added. The mixture was vortexed for 5 seconds again, and left standing at 20–25 °C for 15 min. In another 1.5 mL Eppendorf tube, 60 µL of HP–AOP–NH_2 (0.33 mM, 20 nmol, 1.0 equiv.) in borate buffer (pH 9.4, c = 400 mM) was added, and the premix of acid, HOAt, EDC·HCl and NMM was added over the solution. The mixture was vortexed for 5 seconds, transferred into a Thermocycler at 25 °C, and incubated at 25 °C for 2 hours at 600 rpm. After 2 hours, an aliquot of 1 µL of the reaction mixture was diluted to 40 µL with water for LC–MS analysis.

Next, 10 µL of a 5 M solution of NaCl in water and 360 µL of ethanol at −20 °C were added to precipitate the DNA conjugate. The Eppendorf tube was placed in the freezer (−20 °C) for at least 1 hour, and then it was centrifuged at 4 °C and 10000 x g for at least 30 minutes. The supernatant was removed, the pellet was redissolved in 100 µL of water, and the procedure was repeated again. The remaining pellet was then dried under a flow of nitrogen, redissolved with 300 µL of water and the solution of DNA conjugate was then desalted.

DNA desalting and rebuffering was performed by charging the solution in an AMICON® filter unit from Sigma Aldrich (3 kD) in 300 µL of water, centrifuged at 4 °C and 10000 x g for at least 30 minutes, until the volume decreased to < 10 µL. Another 300 µL of water were added and the process was repeated all over again for at least 3 times. The remaining solution concentration was determined by A_{260} absorption using a Thermo Scientific™ NanoDrop™ One^{C}, concentration of the solution was adjusted to 2.0 mM and stored in the freezer at −20 °C.

Performing a desalting step via AMICON® filter after amidation with EDC·HCl method was crucial before the C−H functionalization step.

General procedure for synthesis of DNA-conjugated arenes with DMT-MM

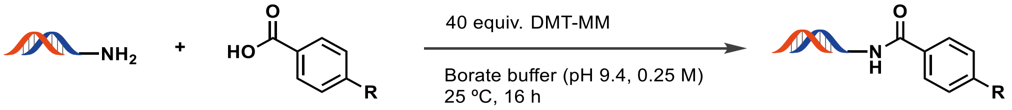

40 equiv.

At 20–25 °C, 20 µL of HP–AOP–NH_2 (1.0 mM, 20 nmol, 1.0 equiv.) in borate buffer (pH 9.4, c = 250 mM) was added to a 1.5 mL Eppendorf tube. Next, 2.0 µL of a carboxylic acid stock solution (400 mM, 0.80 µmol, 40 equiv.) in DMA was added. The mixture was vortexed for 5 seconds. Then, 2.0 µL of a DMT-MM stock solution (400 mM, 0.80 µmol, 40 equiv.) in water was added. The mixture was vortexed for 5 seconds again, transferred into a Thermocycler at 25 °C, and incubated at 25 °C for 16 hours at 600 rpm. After 16 hours, an aliquot of 1 µL of the reaction mixture was diluted to 40 µL with water for LC–MS

analysis.

Next, 2.4 µL of a 5 M solution of NaCl in water and 80 µL of ethanol at −20 °C were added to precipitate the DNA conjugate. The Eppendorf tube was placed in the freezer (−20 °C) for at least 1 hour, and then it was centrifuged at 4 °C and 10000 x g for at least 30 minutes. The supernatant was removed, the pellet was redissolved in 24 µL of water, and the procedure was repeated again. The remaining pellet was then dried under a flow of nitrogen, redissolved with 10 µL of water and stored in the freezer at −20 °C.

General procedure for synthesis of DNA-conjugated arenes by reverse amidation

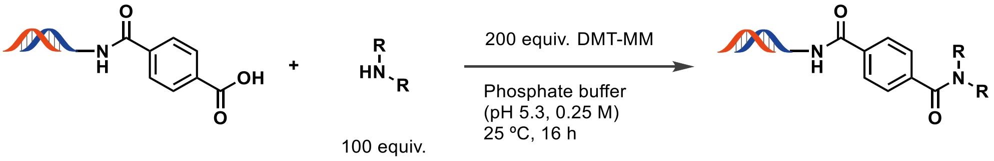

At 20–25 °C, 20 µL of HP–AOP–NHCOPhCO$_2$H (1.0 mM, 20 nmol, 1.0 equiv.) in phosphate buffer (pH 5.3, c = 250 mM) was added to a 1.5 mL Eppendorf tube. Next, 5.0 µL of an amine stock solution (400 mM, 2.0 µmol, 100 equiv.) in DMA was added. The mixture was vortexed for 5 seconds. Then, 5.0 µL of a DMT-MM stock solution (400 mM, 2.0 µmol, 100 equiv.) in water was added. The mixture was vortexed for 5 seconds again, transferred into a Thermocycler at 25 °C, and incubated at 25 °C for 2 hours at 600 rpm. After 2h, a second addition of 5.0 µL of a DMT-MM stock solution (400 mM, 2.0 µmol, 100 equiv.) in water was done. The mixture was vortexed for 5 seconds, transferred into a Thermocycler at 25 °C, and incubated at 25 °C for 14 more hours at 600 rpm. After 16 hours, an aliquot of 1 µL of the reaction mixture was diluted to 40 µL with water for LC–MS analysis.

Next, 3.5 µL of a 5 M solution of NaCl in water and 100 µL of ethanol at −20 °C were added to precipitate the DNA conjugate. The Eppendorf tube was placed in the freezer (−20 °C) for at least 1 hour, and then it was centrifuged at 4 °C and 10000 x g for at least 30 minutes. The supernatant was removed, the pellet was redissolved in 35 µL of water, and the procedure was repeated again. The remaining pellet was then dried under a flow of nitrogen, redissolved with 10 µL of water and stored in the freezer at −20 °C.

General procedure for synthesis of DNA-conjugated arenes by reductive amination

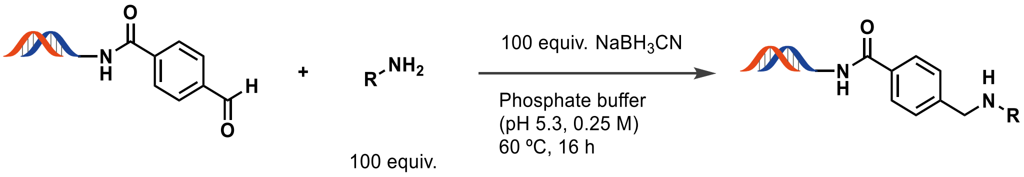

At 20–25 °C, 20 µL of HP–AOP–NHCOPhCOH (1.0 mM, 20 nmol, 1.0 equiv.) in phosphate buffer (pH 5.3, c = 250 mM) was added to a 1.5 mL Eppendorf tube. Next, 10 µL of an amine stock solution (200 mM, 2.0 µmol, 100 equiv.) in DMA was added. The mixture was vortexed for 5 seconds. Then, 10 µL of a sodium cyanoborohydride stock solution (200 mM, 2.0 µmol, 100 equiv.) in MeCN was added. The mixture was vortexed for 5 seconds again, transferred into a pre-heated Thermocycler at 60 °C, and incubated at 60 °C for 16 hours at 1000 rpm. After 16 hours, an aliquot of 1 µL of the reaction mixture was diluted to 40 µL with water for LC–MS analysis.

Next, 4 µL of a 5 M solution of NaCl in water and 130 µL of ethanol at −20 °C were added to precipitate the DNA conjugate. The Eppendorf tube was placed in the freezer (−20 °C) for at least 1 hour, and then it

was centrifuged at 4 °C and 10000 x g for at least 30 minutes. The supernatant was removed, the pellet was redissolved in 35 µL of water, and the procedure was repeated again. The remaining pellet was then dried under a flow of nitrogen, redissolved with 10 µL of water and stored in the freezer at −20 °C.

General procedure for synthesis of DNA-conjugated arenes by S_NAr

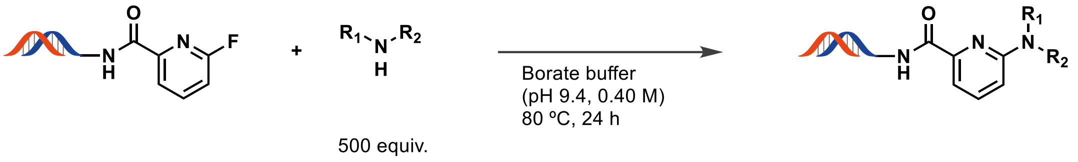

At 20–25 °C, 80 µL of HP–AOP–NHCOPyF (0.67 mM, 40 nmol, 1.0 equiv.) in borate buffer (pH 9.4, c = 400 mM) was added to a 1.5 mL Eppendorf tube. Next, 40 µL of amine stock solution (500 mM, 20 µmol, 500 equiv.) in DMSO was added. The mixture was vortexed for 5 seconds again, transferred into a pre-heated Thermocycler at 80 °C, and incubated at 80 °C for 16 hours at 1000 rpm. After 24 hours, an aliquot of 1 µL of the reaction mixture was diluted to 40 µL with water for LC–MS analysis.

Next, 12 µL of a 5 M solution of NaCl in water and 400 µL of ethanol at −20 °C were added to precipitate the DNA conjugate. The Eppendorf tube was placed in the freezer (−20 °C) for at least 1 hour, and then it was centrifuged at 4 °C and 10000 x g for at least 30 minutes. The supernatant was removed, the pellet was redissolved in 120 µL of water, and the procedure was repeated again. The remaining pellet was then dried under a flow of nitrogen, redissolved with water to 2.0 mM concentration and stored in the freezer at −20 °C.

General procedure for synthesis of DNA-conjugated arenes by Suzuki coupling

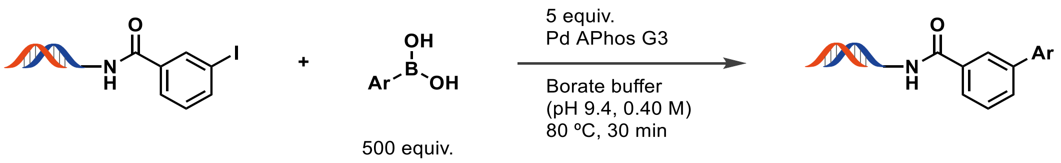

At 20–25 °C, 25 µL of HP–AOP–NHCOPhI (1.0 mM, 25 nmol, 1.0 equiv.) in water was added to a 1.5 mL Eppendorf tube. Next, 50 µL of Borate buffer (pH 9.4, 500 mM) was added. 6.2 µL of an APhos Pd G3 stock solution (20 mM, 125 nmol, 5.0 equiv.) in DMA was added over the solution of **S33**. The mixture was vortexed for 5 seconds. Lastly, 25 µL of a boronic acid stock solution (500 mM, 12.5 µmol, 500 equiv.) in DMA was added. The mixture was vortexed for 5 seconds, transferred into a Thermocycler pre-heated at 80 °C, and incubated at 80 °C for 30 min. at 600 rpm.

After 15 minutes, 10 µL of a 100 mM solution of sodium diethyldithiocarbamate trihydrate in water were added to remove the palladium salts from the solution. The reaction mixture was centrifuged, and the supernatant was diluted to 200 µL with water. Over the sample, 20 µL of a 5 M solution of NaCl in water and 600 µL of ethanol at −20 °C were added to precipitate the DNA conjugate. The Eppendorf tube was placed in the freezer (−20 °C) for at least 1 hour, and then it was centrifuged at 4 °C and 10000 x g for at least 30 minutes. The remaining pellet was then dried under a flow of nitrogen, redissolved with 12.5 µL of water to 2.0 mM concentration and stored in the freezer at −20 °C.

Preparation of DNA-conjugated substrates

DNA-conjugated arene S5

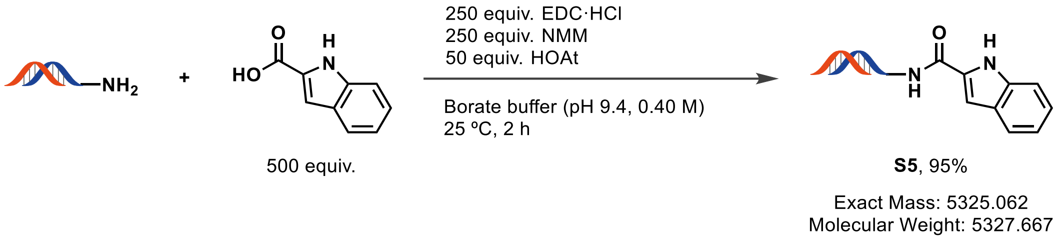

At 20–25 °C, 20 µL of a indole-2-carboxylic stock solution (500 mM, 10 µmol, 500 equiv.) in DMSO was mixed with 10 µL of a HOAt stock solution in DMSO (100 mM, 1.0 µmol, 50 equiv.), and 10 µL of an EDC·HCl stock solution in DMSO (500 mM, 5.0 µmol, 250 equiv.) in a 1.5 mL Eppendorf tube. The mixture was vortexed for 5 seconds. Next, 10 µL of a NMM stock solution (500 mM, 5.0 µmol, 250 equiv.) in DMSO was added. The mixture was vortexed for 5 seconds again, and left standing at 20–25 °C for 15 min. In another 1.5 mL Eppendorf tube, 60 µL of HP–AOP–NH_2 (0.33 mM, 20 nmol, 1.0 equiv.) in borate buffer (pH 9.4, c = 400 mM) was added, and the premix of acid, HOAt, EDC·HCl and NMM was added over the solution. The mixture was vortexed for 5 seconds, transferred into a Thermocycler at 25 °C, and incubated at 25 °C for 2 hours at 600 rpm. After 2 hours, an aliquot of 1 µL of the reaction mixture was diluted to 40 µL with water for LC–MS analysis.

Next, 10 µL of a 5 M solution of NaCl in water and 360 µL of ethanol at −20 °C were added to precipitate the DNA conjugate. The Eppendorf tube was placed in the freezer (−20 °C) for at least 1 hour, and then it was centrifuged at 4 °C and 10000 x g for at least 30 minutes. The supernatant was removed, the pellet was redissolved in 300 µL of water, and the procedure was repeated again. The remaining pellet was then dried under a flow of nitrogen, redissolved with 300 µL of water and the solution of DNA conjugate was then desalted. DNA desalting and rebuffering was performed by charging the solution in an AMICON® filter unit from Sigma Aldrich (3 kD) in 300 µL of water, centrifuged at 4 °C and 10000 x g for at least 30 minutes, until the volume decreased to < 10 µL. Another 300 µL of water were added and the process was repeated all over again for at least 3 times. The remaining solution concentration was determined by A_{260} absorption using a Thermo Scientific™ NanoDrop™ One^{C}, concentration of the solution was adjusted to 2.0 mM and stored in the freezer at −20 °C.

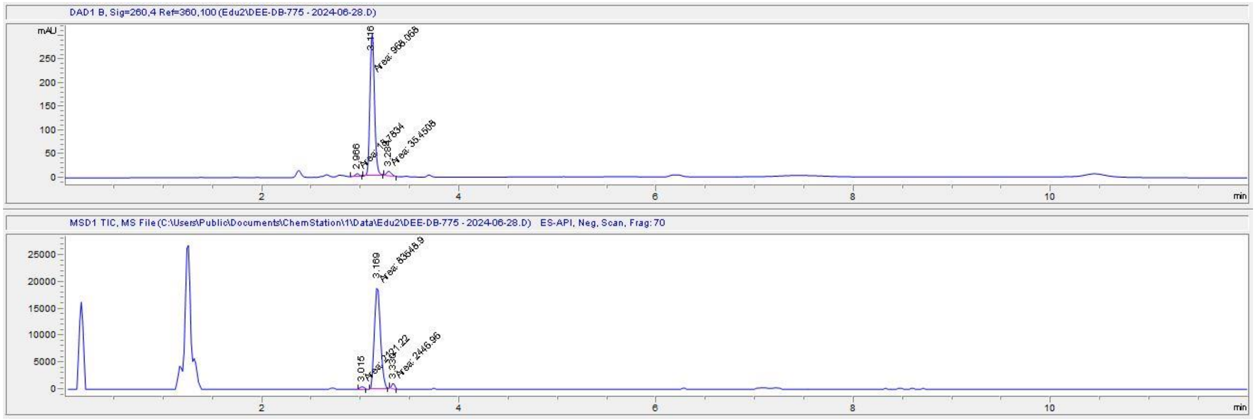

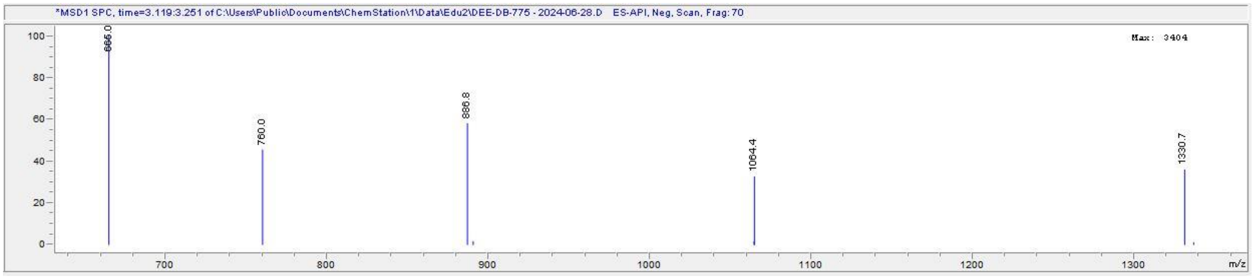

Figure S2. Analytical HPLC trace of **S5** with HPLC Method A. (Up) DAD chromatogram at 260 nm. (Middle) TIC chromatogram. (Below) Ionization of peak at 3.169 min containing reaction product.

DNA-conjugated arene S6

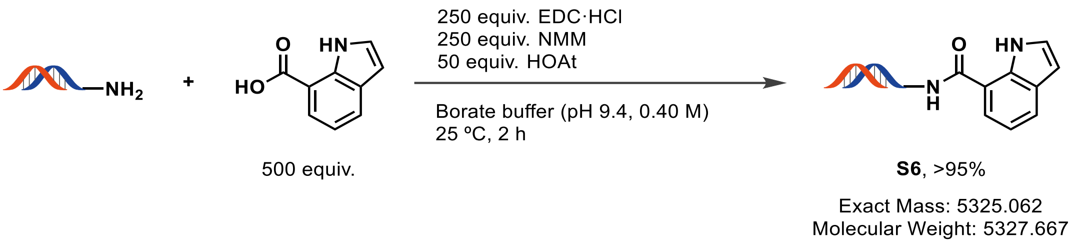

At 20–25 °C, 20 µL of a indole-7-carboxylic stock solution (500 mM, 10 µmol, 500 equiv.) in DMSO was mixed with 10 µL of a HOAt stock solution in DMSO (100 mM, 1.0 µmol, 50 equiv.), and 10 µL of an EDC·HCl stock solution in DMSO (500 mM, 5.0 µmol, 250 equiv.) in a 1.5 mL Eppendorf tube. The mixture was vortexed for 5 seconds. Next, 10 µL of a NMM stock solution (500 mM, 5.0 µmol, 250 equiv.) in DMSO was added. The mixture was vortexed for 5 seconds again, and left standing at 20–25 °C for 15 min. In another 1.5 mL Eppendorf tube, 60 µL of HP–AOP–NH_2 (0.33 mM, 20 nmol, 1.0 equiv.) in borate buffer (pH 9.4, c = 400 mM) was added, and the premix of acid, HOAt, EDC·HCl and NMM was added over the solution. The mixture was vortexed for 5 seconds, transferred into a Thermocycler at 25 °C, and incubated at 25 °C for 2 hours at 600 rpm. After 2 hours, an aliquot of 1 µL of the reaction mixture was diluted to 40 µL with water for LC–MS analysis.

Next, 10 µL of a 5 M solution of NaCl in water and 360 µL of ethanol at −20 °C were added to precipitate the DNA conjugate. The Eppendorf tube was placed in the freezer (−20 °C) for at least 1 hour, and then it was centrifuged at 4 °C and 10000 x g for at least 30 minutes. The supernatant was removed, the pellet was redissolved in 300 µL of water, and the procedure was repeated again. The remaining pellet was then dried under a flow of nitrogen, redissolved with 300 µL of water and the solution of DNA conjugate was then desalted. DNA desalting and rebuffering was performed by charging the solution in an AMICON® filter unit from Sigma Aldrich (3 kD) in 300 µL of water, centrifuged at 4 °C and 10000 x g for at least 30 minutes, until the volume decreased to < 10 µL. Another 300 µL of water were added and the process was repeated all over again for at least 3 times. The remaining solution concentration was determined by A_{260} absorption using a Thermo Scientific™ NanoDrop™ One^{C}, concentration of the solution was adjusted to 2.0 mM and stored in the freezer at −20 °C.

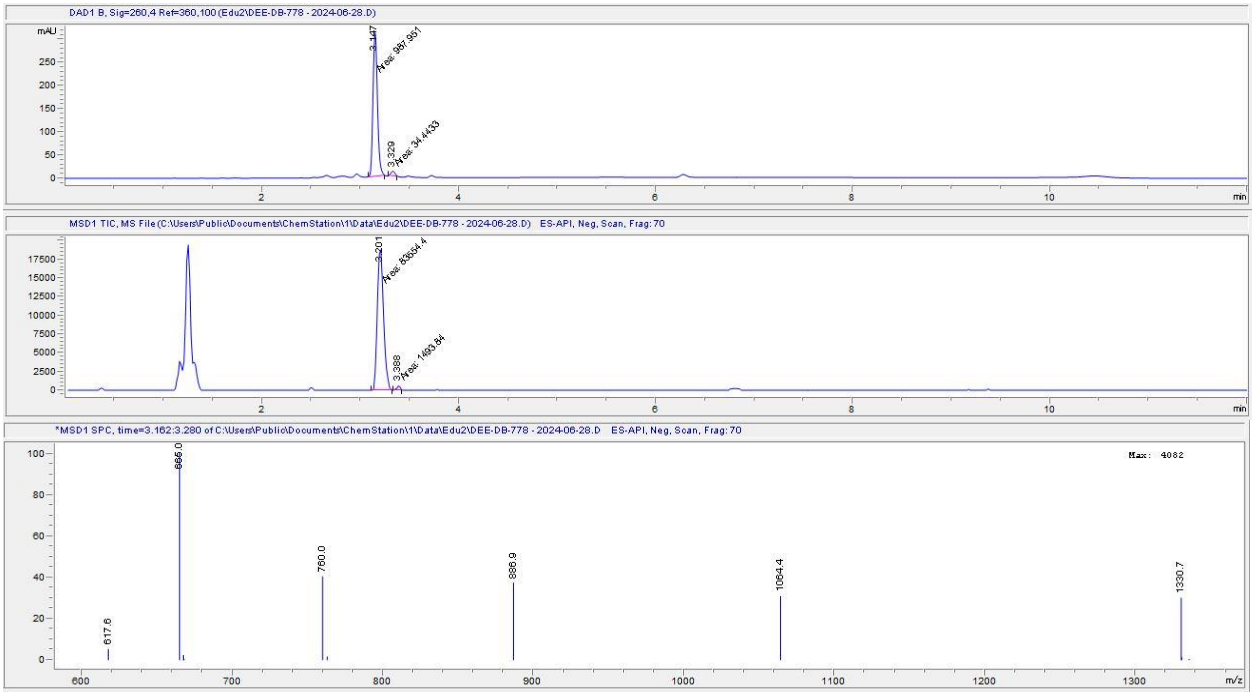

Figure S3. Analytical HPLC trace of **S6** with HPLC Method A. (Up) DAD chromatogram at 260 nm. (Middle) TIC chromatogram. (Below) Ionization of peak at 3.201 min containing reaction product.

DNA-conjugated arene S7

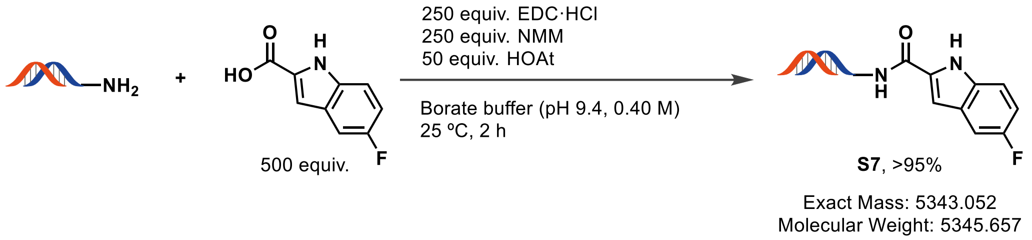

At 20–25 °C, 20 µL of a 5-fluoroindole-2-carboxylic stock solution (500 mM, 10 µmol, 500 equiv.) in DMSO was mixed with 10 µL of a HOAt stock solution in DMSO (100 mM, 1.0 µmol, 50 equiv.), and 10 µL of an EDC·HCl stock solution in DMSO (500 mM, 5.0 µmol, 250 equiv.) in a 1.5 mL Eppendorf tube. The mixture was vortexed for 5 seconds. Next, 10 µL of a NMM stock solution (500 mM, 5.0 µmol, 250 equiv.) in DMSO was added. The mixture was vortexed for 5 seconds again, and left standing at 20–25 °C for 15 min. In another 1.5 mL Eppendorf tube, 60 µL of HP–AOP–NH_2 (0.33 mM, 20 nmol, 1.0 equiv.) in borate buffer (pH 9.4, c = 400 mM) was added, and the premix of acid, HOAt, EDC·HCl and NMM was added over the solution. The mixture was vortexed for 5 seconds, transferred into a Thermocycler at 25 °C, and incubated at 25 °C for 2 hours at 600 rpm. After 2 hours, an aliquot of 1 µL of the reaction mixture was diluted to 40 µL with water for LC–MS analysis.

Next, 10 µL of a 5 M solution of NaCl in water and 360 µL of ethanol at −20 °C were added to precipitate the DNA conjugate. The Eppendorf tube was placed in the freezer (−20 °C) for at least 1 hour, and then it was centrifuged at 4 °C and 10000 x g for at least 30 minutes. The supernatant was removed, the pellet was redissolved in 300 µL of water, and the procedure was repeated again. The remaining pellet was then dried under a flow of nitrogen, redissolved with 300 µL of water and the solution of DNA conjugate was then desalted. DNA desalting and rebuffering was performed by charging the solution in an AMICON® filter unit from Sigma Aldrich (3 kD) in 300 µL of water, centrifuged at 4 °C and 10000 x g for at

least 30 minutes, until the volume decreased to < 10 µL. Another 300 µL of water were added and the process was repeated all over again for at least 3 times. The remaining solution concentration was determined by A_{260} absorption using a Thermo Scientific™ NanoDrop™ One^{C}, concentration of the solution was adjusted to 2.0 mM and stored in the freezer at −20 °C.

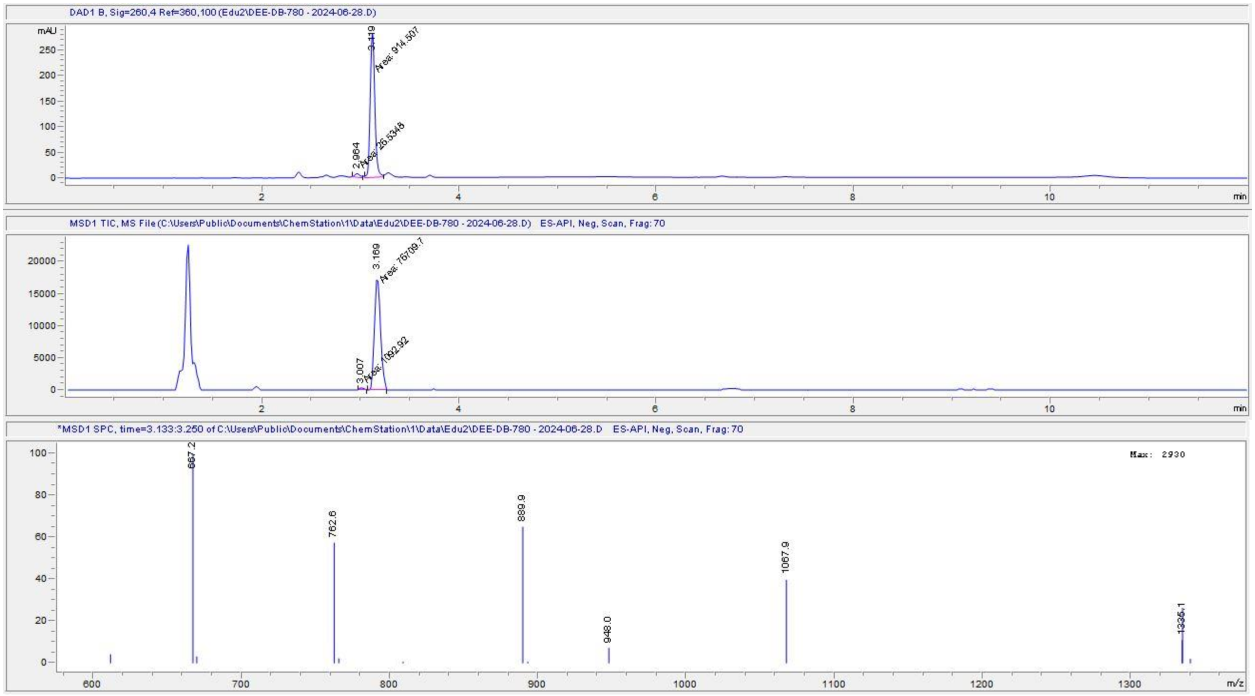

Figure S4. Analytical HPLC trace of **S7** with HPLC Method A. (Up) DAD chromatogram at 260 nm. (Middle) TIC chromatogram. (Below) Ionization of peak at 3.169 min containing reaction product.

DNA-conjugated arene S8

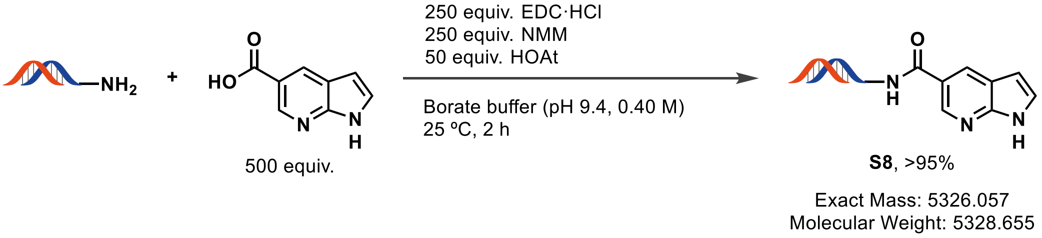

At 20–25 °C, 20 µL of a 1*H*-pyrrolo[2,3-*b*]pyridine-5-carboxylic stock solution (500 mM, 10 µmol, 500 equiv.) in DMSO was mixed with 10 µL of a HOAt stock solution in DMSO (100 mM, 1.0 µmol, 50 equiv.), and 10 µL of an EDC·HCl stock solution in DMSO (500 mM, 5.0 µmol, 250 equiv.) in a 1.5 mL Eppendorf tube. The mixture was vortexed for 5 seconds. Next, 10 µL of a NMM stock solution (500 mM, 5.0 µmol, 250 equiv.) in DMSO was added. The mixture was vortexed for 5 seconds again, and left standing at 20–25 °C for 15 min. In another 1.5 mL Eppendorf tube, 60 µL of HP–AOP–NH_2 (0.33 mM, 20 nmol, 1.0 equiv.) in borate buffer (pH 9.4, c = 400 mM) was added, and the premix of acid, HOAt, EDC·HCl and NMM was added over the solution. The mixture was vortexed for 5 seconds, transferred into a Thermocycler at 25 °C, and incubated at 25 °C for 2 hours at 600 rpm. After 2 hours, an aliquot of 1 µL of the reaction mixture was diluted to 40 µL with water for LC–MS analysis.

Next, 10 µL of a 5 M solution of NaCl in water and 360 µL of ethanol at −20 °C were added to precipitate the DNA conjugate. The Eppendorf tube was placed in the freezer (−20 °C) for at least 1 hour, and then it was centrifuged at 4 °C and 10000 x g for at least 30 minutes. The supernatant was removed, the pellet

was redissolved in 300 µL of water, and the procedure was repeated again. The remaining pellet was then dried under a flow of nitrogen, redissolved with 300 µL of water and the solution of DNA conjugate was then desalted. DNA desalting and rebuffering was performed by charging the solution in an AMICON® filter unit from Sigma Aldrich (3 kD) in 300 µL of water, centrifuged at 4 °C and 10000 x g for at least 30 minutes, until the volume decreased to < 10 µL. Another 300 µL of water were added and the process was repeated all over again for at least 3 times. The remaining solution concentration was determined by A_{260} absorption using a Thermo Scientific™ NanoDrop™ One^{C}, concentration of the solution was adjusted to 2.0 mM and stored in the freezer at −20 °C.

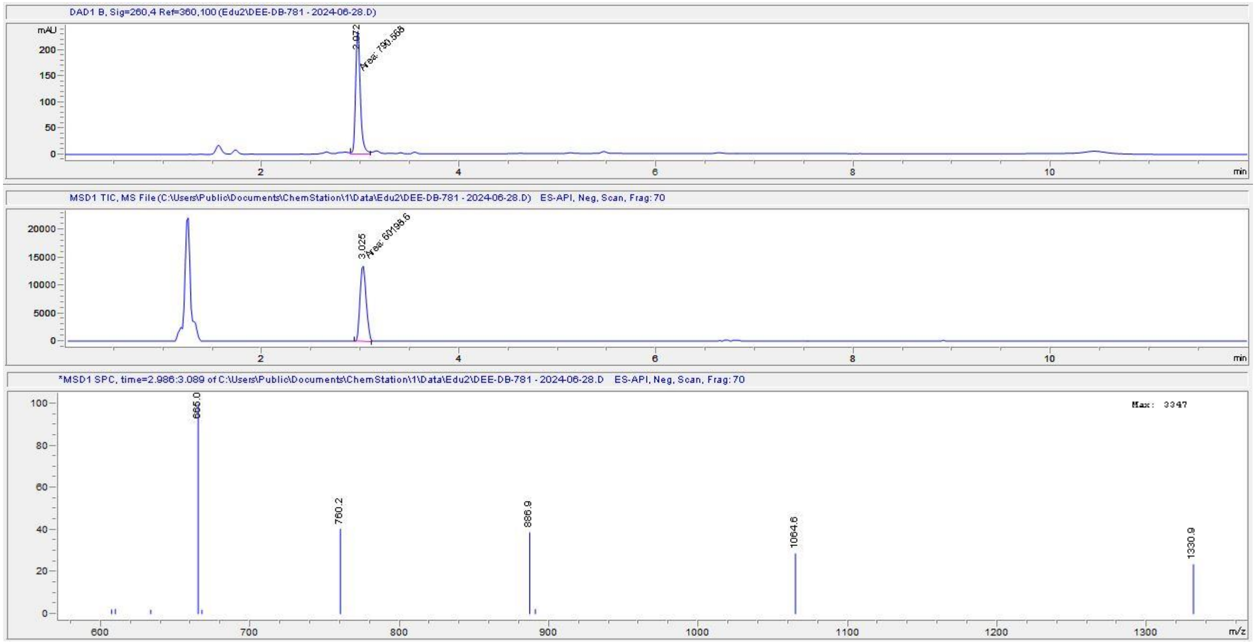

Figure S5. Analytical HPLC trace of **S8** with HPLC Method B. (Up) DAD chromatogram at 260 nm. (Middle) TIC chromatogram. (Below) Ionization of peak at 3.025 min containing reaction product.

DNA-conjugated arene S9

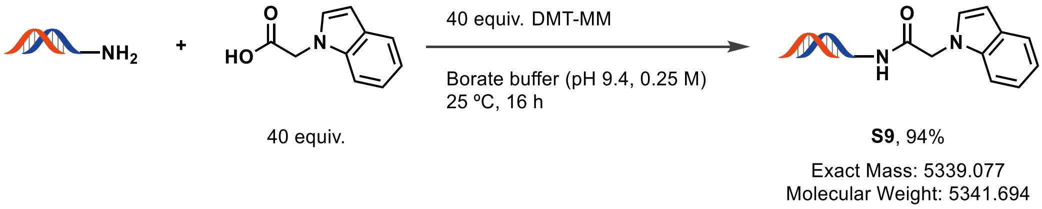

At 20–25 °C, 20 µL of HP–AOP–NH_2 (1.0 mM, 20 nmol, 1.0 equiv.) in borate buffer (pH 9.4, c = 250 mM) was added to a 1.5 mL Eppendorf tube. Next, 2.0 µL of an 2-(1*H*-indol-1-yl)acetic acid stock solution (400 mM, 0.80 µmol, 40 equiv.) in DMA was added. The mixture was vortexed for 5 seconds. Then, 2.0 µL of a DMT-MM stock solution (400 mM, 0.80 µmol, 40 equiv.) in water was added. The mixture was vortexed for 5 seconds again, transferred into a Thermocycler at 25 °C, and incubated at 25 °C for 16 hours at 600 rpm. After 16 hours, an aliquot of 1 µL of the reaction mixture was diluted to 40 µL with water for LC–MS analysis.

Next, 2.4 µL of a 5 M solution of NaCl in water and 80 µL of ethanol at −20 °C were added to precipitate the DNA conjugate. The Eppendorf tube was placed in the freezer (−20 °C) for at least 1 hour, and then it was centrifuged at 4 °C and 10000 x g for at least 30 minutes. The supernatant was removed, the pellet was redissolved in 24 µL of water, and the procedure was repeated again. The remaining pellet was then

dried under a flow of nitrogen, redissolved with 10 µL of water and stored in the freezer at −20 °C.

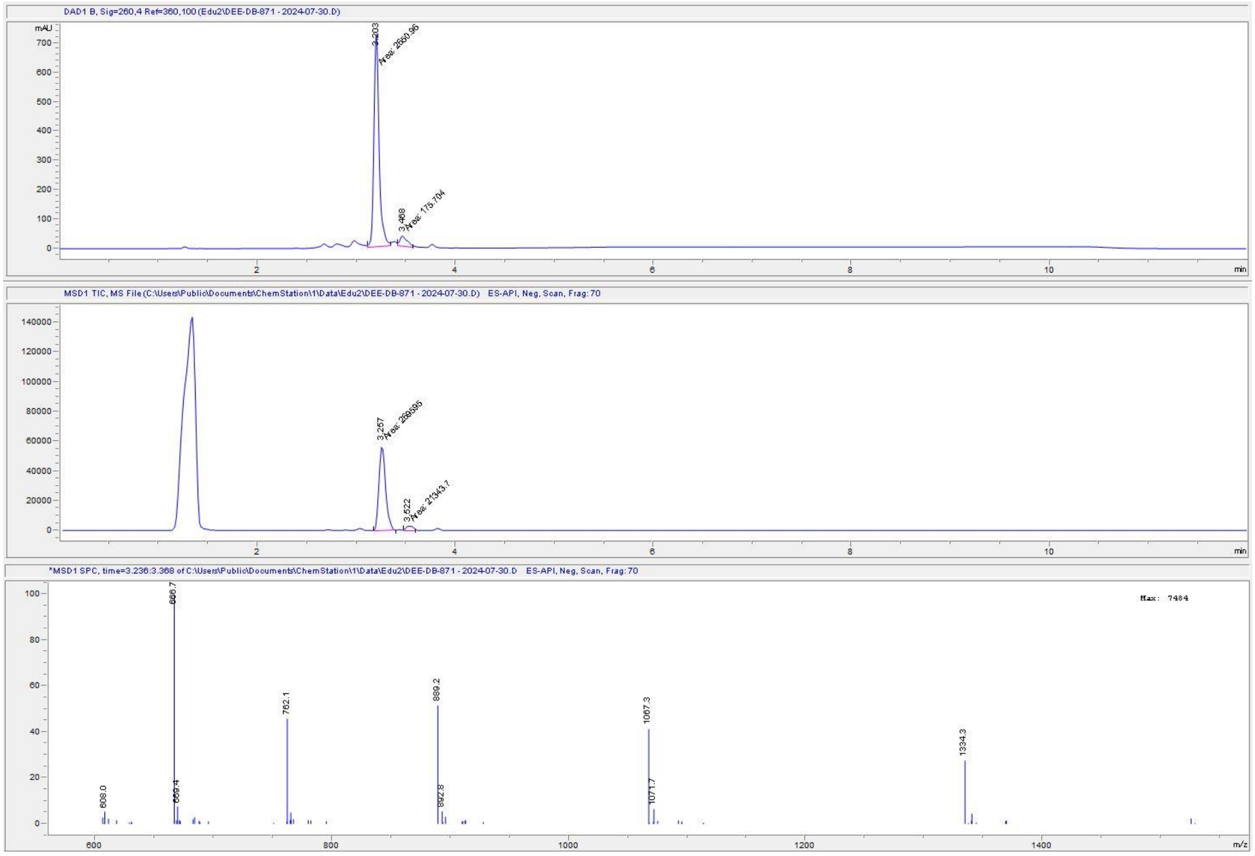

Figure S6. Analytical HPLC trace of **S9** with HPLC Method A. (Up) DAD chromatogram at 260 nm. (Middle) TIC chromatogram. (Below) Ionization of peak at 3.257 min containing reaction product.

DNA-conjugated arene S10

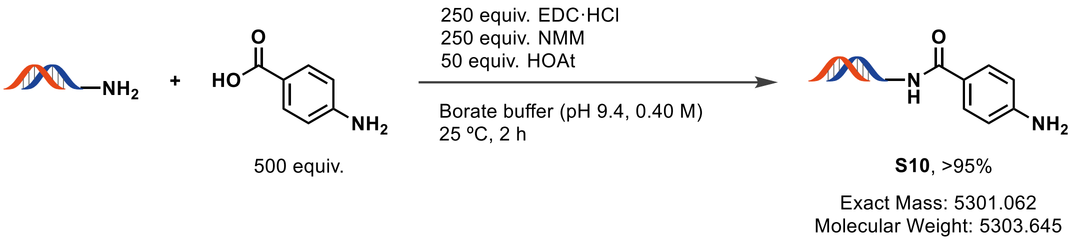

At 20–25 °C, 20 µL of a 4-aminobenzoic stock solution (500 mM, 10 µmol, 500 equiv.) in DMSO was mixed with 10 µL of a HOAt stock solution in DMSO (100 mM, 1.0 µmol, 50 equiv.), and 10 µL of an EDC·HCl stock solution in DMSO (500 mM, 5.0 µmol, 250 equiv.) in a 1.5 mL Eppendorf tube. The mixture was vortexed for 5 seconds. Next, 10 µL of a NMM stock solution (500 mM, 5.0 µmol, 250 equiv.) in DMSO was added. The mixture was vortexed for 5 seconds again, and left standing at 20–25 °C for 15 min. In another 1.5 mL Eppendorf tube, 60 µL of HP–AOP–NH_2 (0.33 mM, 20 nmol, 1.0 equiv.) in borate buffer (pH 9.4, c = 400 mM) was added, and the premix of acid, HOAt, EDC·HCl and NMM was added over the solution. The mixture was vortexed for 5 seconds, transferred into a Thermocycler at 25 °C, and incubated at 25 °C for 2 hours at 600 rpm. After 2 hours, an aliquot of 1 µL of the reaction mixture was diluted to 40 µL with water for LC–MS analysis.

Next, 10 µL of a 5 M solution of NaCl in water and 360 µL of ethanol at −20 °C were added to precipitate the DNA conjugate. The Eppendorf tube was placed in the freezer (−20 °C) for at least 1 hour, and then it

was centrifuged at 4 °C and 10000 x g for at least 30 minutes. The supernatant was removed, the pellet was redissolved in 300 µL of water, and the procedure was repeated again. The remaining pellet was then dried under a flow of nitrogen, redissolved with 300 µL of water and the solution of DNA conjugate was then desalted. DNA desalting and rebuffering was performed by charging the solution in an AMICON® filter unit from Sigma Aldrich (3 kD) in 300 µL of water, centrifuged at 4 °C and 10000 x g for at least 30 minutes, until the volume decreased to < 10 µL. Another 300 µL of water were added and the process was repeated all over again for at least 3 times. The remaining solution concentration was determined by A_{260} absorption using a Thermo Scientific™ NanoDrop™ One^{C}, concentration of the solution was adjusted to 2.0 mM and stored in the freezer at −20 °C.

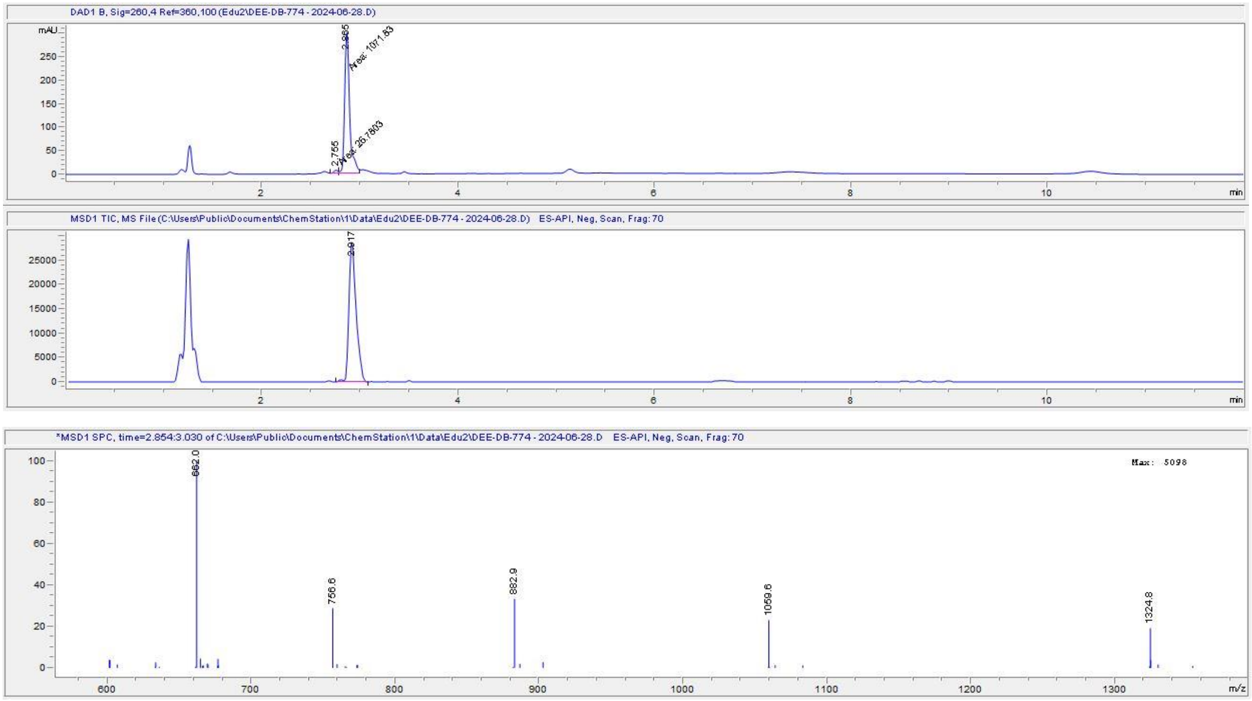

Figure S7. Analytical HPLC trace of **S10** with HPLC Method A. (Up) DAD chromatogram at 260 nm. (Middle) TIC chromatogram. (Below) Ionization of peak at 2.917 min containing reaction product.

DNA-conjugated arene S11

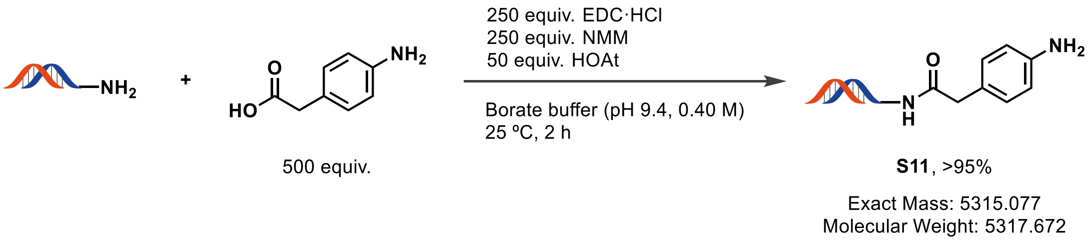

At 20–25 °C, 20 µL of a 2-(4-aminophenyl)acetic stock solution (500 mM, 10 µmol, 500 equiv.) in DMSO was mixed with 10 µL of a HOAt stock solution in DMSO (100 mM, 1.0 µmol, 50 equiv.), and 10 µL of an EDC·HCl stock solution in DMSO (500 mM, 5.0 µmol, 250 equiv.) in a 1.5 mL Eppendorf tube. The mixture was vortexed for 5 seconds. Next, 10 µL of a NMM stock solution (500 mM, 5.0 µmol, 250 equiv.) in DMSO was added. The mixture was vortexed for 5 seconds again, and left standing at 20–25 °C for 15 min. In another 1.5 mL Eppendorf tube, 60 µL of HP–AOP–NH_2 (0.33 mM, 20 nmol, 1.0 equiv.) in borate buffer (pH 9.4, c = 400 mM) was added, and the premix of acid, HOAt, EDC·HCl and NMM was added over the solution. The mixture was vortexed for 5 seconds, transferred into a Thermocycler at 25 °C, and

incubated at 25 °C for 2 hours at 600 rpm. After 2 hours, an aliquot of 1 µL of the reaction mixture was diluted to 40 µL with water for LC–MS analysis.

Next, 10 µL of a 5 M solution of NaCl in water and 360 µL of ethanol at −20 °C were added to precipitate the DNA conjugate. The Eppendorf tube was placed in the freezer (−20 °C) for at least 1 hour, and then it was centrifuged at 4 °C and 10000 x g for at least 30 minutes. The supernatant was removed, the pellet was redissolved in 300 µL of water, and the procedure was repeated again. The remaining pellet was then dried under a flow of nitrogen, redissolved with 300 µL of water and the solution of DNA conjugate was then desalted. DNA desalting and rebuffering was performed by charging the solution in an AMICON® filter unit from Sigma Aldrich (3 kD) in 300 µL of water, centrifuged at 4 °C and 10000 x g for at least 30 minutes, until the volume decreased to < 10 µL. Another 300 µL of water were added and the process was repeated all over again for at least 3 times. The remaining solution concentration was determined by A_{260} absorption using a Thermo Scientific™ NanoDrop™ One^{C}, concentration of the solution was adjusted to 2.0 mM and stored in the freezer at −20 °C.

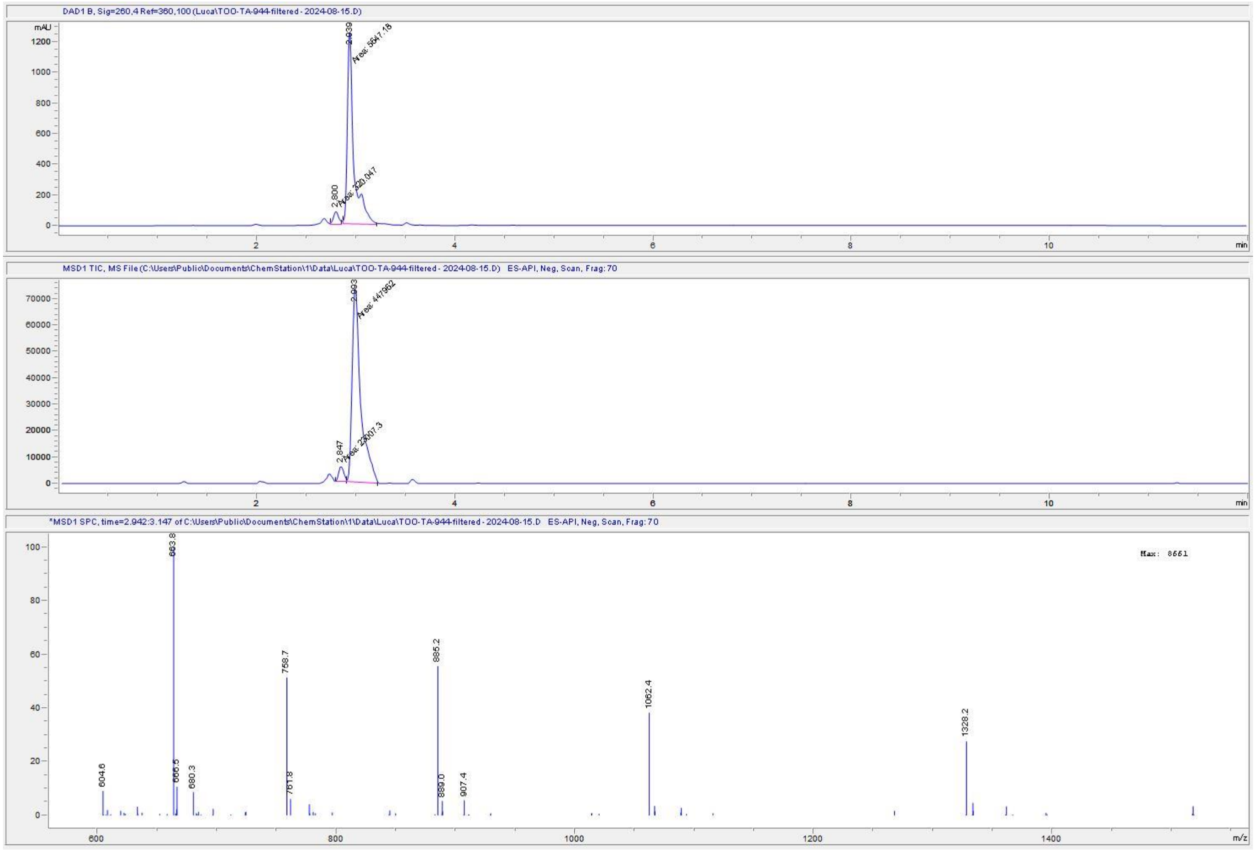

Figure S8. Analytical HPLC trace of **S11** with HPLC Method A. (Up) DAD chromatogram at 260 nm. (Middle) TIC chromatogram. (Below) Ionization of peak at 2.993 min containing reaction product.

DNA-conjugated arene S12

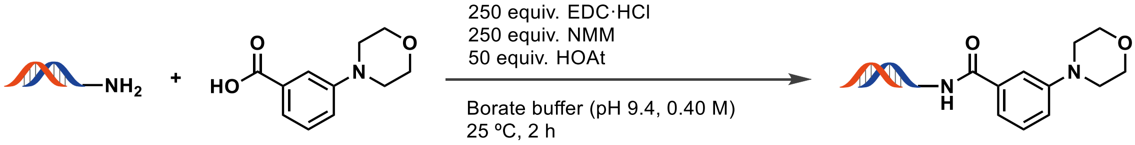

500 equiv.

S12, >95%

Exact Mass: 5371.104
Molecular Weight: 5373.736

At 20–25 °C, 20 µL of a 3-morpholinobenzoic stock solution (500 mM, 10 µmol, 500 equiv.) in DMSO was mixed with 10 µL of a HOAt stock solution in DMSO (100 mM, 1.0 µmol, 50 equiv.), and 10 µL of an EDC·HCl stock solution in DMSO (500 mM, 5.0 µmol, 250 equiv.) in a 1.5 mL Eppendorf tube. The mixture was vortexed for 5 seconds. Next, 10 µL of a NMM stock solution (500 mM, 5.0 µmol, 250 equiv.) in DMSO was added. The mixture was vortexed for 5 seconds again, and left standing at 20–25 °C for 15 min. In another 1.5 mL Eppendorf tube, 60 µL of HP–AOP–NH_2 (0.33 mM, 20 nmol, 1.0 equiv.) in borate buffer (pH 9.4, c = 400 mM) was added, and the premix of acid, HOAt, EDC·HCl and NMM was added over the solution. The mixture was vortexed for 5 seconds, transferred into a Thermocycler at 25 °C, and incubated at 25 °C for 2 hours at 600 rpm. After 2 hours, an aliquot of 1 µL of the reaction mixture was diluted to 40 µL with water for LC–MS analysis.

Next, 10 µL of a 5 M solution of NaCl in water and 360 µL of ethanol at −20 °C were added to precipitate the DNA conjugate. The Eppendorf tube was placed in the freezer (−20 °C) for at least 1 hour, and then it was centrifuged at 4 °C and 10000 x g for at least 30 minutes. The supernatant was removed, the pellet was redissolved in 300 µL of water, and the procedure was repeated again. The remaining pellet was then dried under a flow of nitrogen, redissolved with 300 µL of water and the solution of DNA conjugate was then desalted. DNA desalting and rebuffering was performed by charging the solution in an AMICON® filter unit from Sigma Aldrich (3 kD) in 300 µL of water, centrifuged at 4 °C and 10000 x g for at least 30 minutes, until the volume decreased to < 10 µL. Another 300 µL of water were added and the process was repeated all over again for at least 3 times. The remaining solution concentration was determined by A_{260} absorption using a Thermo Scientific™ NanoDrop™ One^{C}, concentration of the solution was adjusted to 2.0 mM and stored in the freezer at −20 °C.

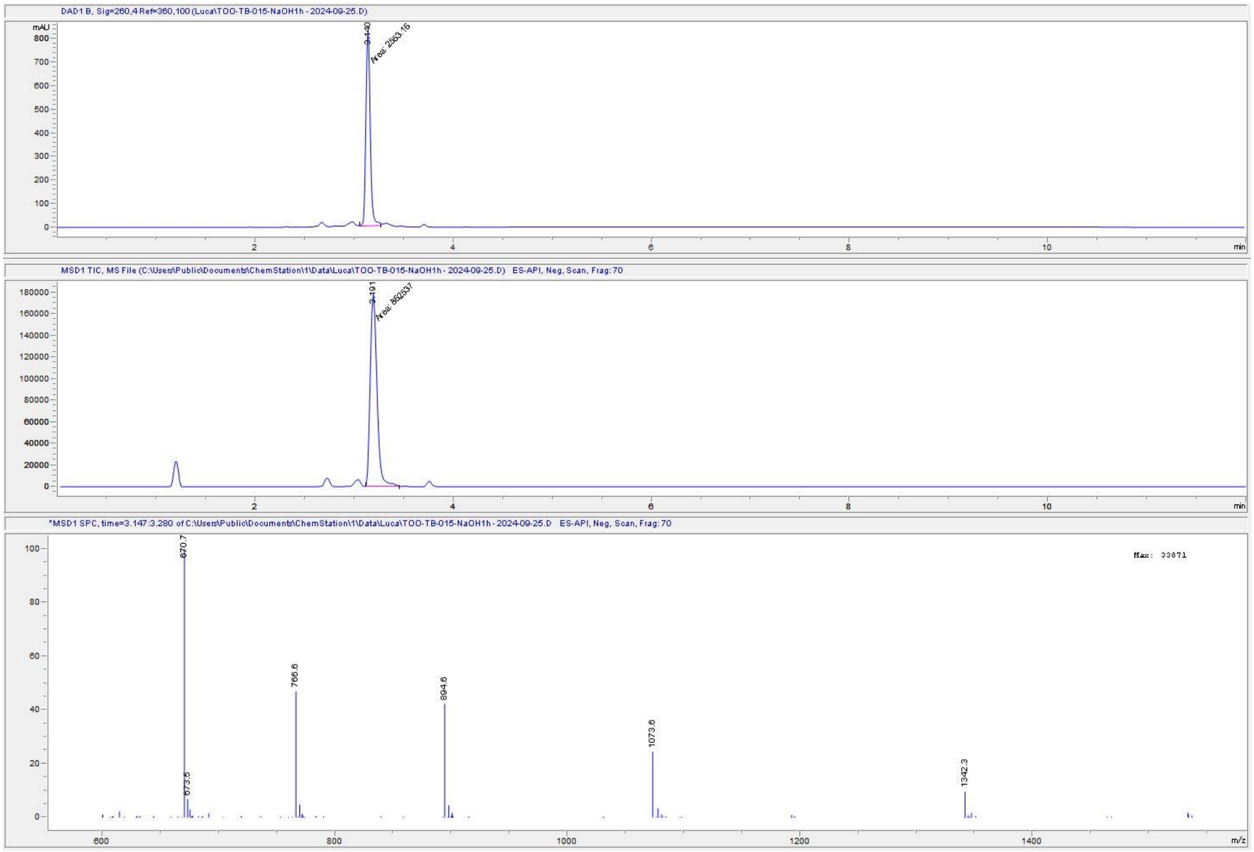

Figure S9. Analytical HPLC trace of **S12** with HPLC Method A. (Up) DAD chromatogram at 260 nm. (Middle) TIC chromatogram. (Below) Ionization of peak at 3.191 min containing reaction product.

DNA-conjugated arene S13

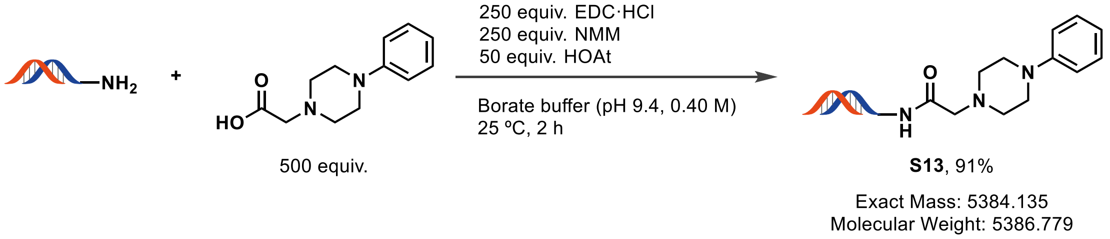

At 20–25 °C, 20 µL of a 2-(4-phenylpiperazin-1-yl)acetic acid stock solution (500 mM, 10 µmol, 500 equiv.) in DMSO was mixed with 10 µL of a HOAt stock solution in DMSO (100 mM, 1.0 µmol, 50 equiv.), and 10 µL of an EDC·HCl stock solution in DMSO (500 mM, 5.0 µmol, 250 equiv.) in a 1.5 mL Eppendorf tube. The mixture was vortexed for 5 seconds. Next, 10 µL of a NMM stock solution (500 mM, 5.0 µmol, 250 equiv.) in DMSO was added. The mixture was vortexed for 5 seconds again, and left standing at 20–25 °C for 15 min. In another 1.5 mL Eppendorf tube, 60 µL of HP–AOP–NH_2 (0.33 mM, 20 nmol, 1.0 equiv.) in borate buffer (pH 9.4, c = 400 mM) was added, and the premix of acid, HOAt, EDC·HCl and NMM was added over the solution. The mixture was vortexed for 5 seconds, transferred into a Thermocycler at 25 °C, and incubated at 25 °C for 2 hours at 600 rpm. After 2 hours, an aliquot of 1 µL of the reaction mixture was diluted to 40 µL with water for LC–MS analysis.

Next, 10 µL of a 5 M solution of NaCl in water and 360 µL of ethanol at −20 °C were added to precipitate the DNA conjugate. The Eppendorf tube was placed in the freezer (−20 °C) for at least 1 hour, and then it was centrifuged at 4 °C and 10000 x g for at least 30 minutes. The supernatant was removed, the pellet was redissolved in 300 µL of water, and the procedure was repeated again. The remaining pellet was then dried under a flow of nitrogen, redissolved with 300 µL of water and the solution of DNA conjugate was then desalted. DNA desalting and rebuffering was performed by charging the solution in an AMICON® filter unit from Sigma Aldrich (3 kD) in 300 µL of water, centrifuged at 4 °C and 10000 x g for at least 30 minutes, until the volume decreased to < 10 µL. Another 300 µL of water were added and the process was repeated all over again for at least 3 times. The remaining solution concentration was determined by A_{260} absorption using a Thermo Scientific™ NanoDrop™ One^C, concentration of the solution was adjusted to 2.0 mM and stored in the freezer at −20 °C.

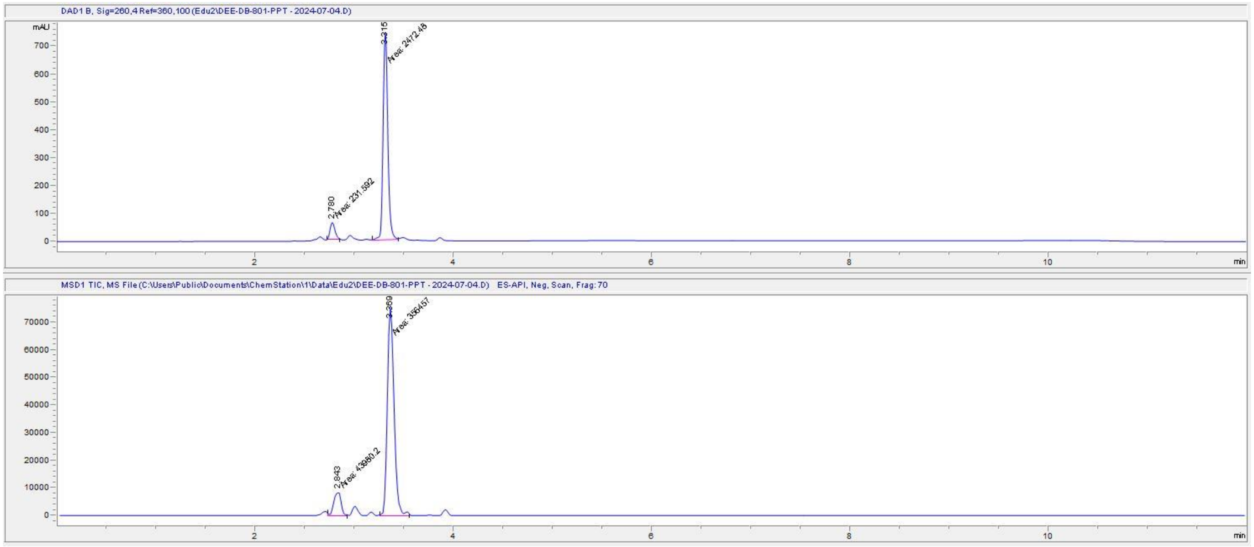

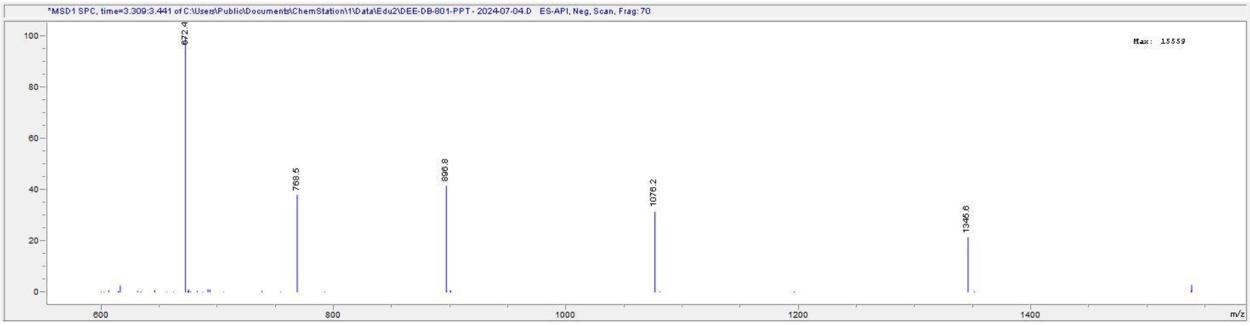

Figure S10. Analytical HPLC trace of **S13** with HPLC Method A. (Up) DAD chromatogram at 260 nm. (Middle) TIC chromatogram. (Below) Ionization of peak at 3.369 min containing reaction product.

DNA-conjugated arene S14

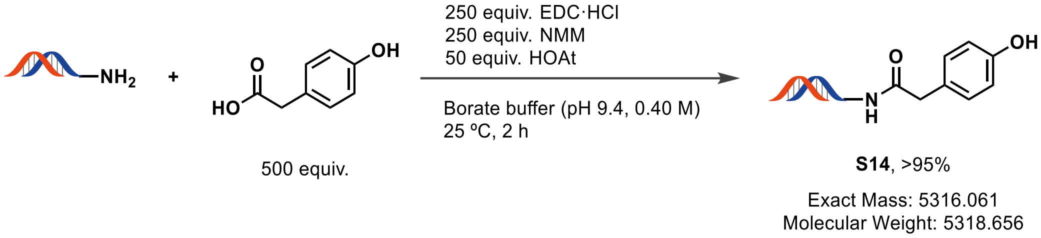

At 20–25 °C, 20 µL of a 2-(4-hydroxyphenyl)acetic acid stock solution (500 mM, 10 µmol, 500 equiv.) in DMSO was mixed with 10 µL of a HOAt stock solution in DMSO (100 mM, 1.0 µmol, 50 equiv.), and 10 µL of an EDC·HCl stock solution in DMSO (500 mM, 5.0 µmol, 250 equiv.) in a 1.5 mL Eppendorf tube. The mixture was vortexed for 5 seconds. Next, 10 µL of a NMM stock solution (500 mM, 5.0 µmol, 250 equiv.) in DMSO was added. The mixture was vortexed for 5 seconds again, and left standing at 20–25 °C for 15 min. In another 1.5 mL Eppendorf tube, 60 µL of HP–AOP–NH_2 (0.33 mM, 20 nmol, 1.0 equiv.) in borate buffer (pH 9.4, c = 400 mM) was added, and the premix of acid, HOAt, EDC·HCl and NMM was added over the solution. The mixture was vortexed for 5 seconds, transferred into a Thermocycler at 25 °C, and incubated at 25 °C for 2 hours at 600 rpm. After 2 hours, an aliquot of 1 µL of the reaction mixture was diluted to 40 µL with water for LC–MS analysis.

Next, 10 µL of a 5 M solution of NaCl in water and 360 µL of ethanol at −20 °C were added to precipitate the DNA conjugate. The Eppendorf tube was placed in the freezer (−20 °C) for at least 1 hour, and then it was centrifuged at 4 °C and 10000 x g for at least 30 minutes. The supernatant was removed, the pellet was redissolved in 300 µL of water, and the procedure was repeated again. The remaining pellet was then dried under a flow of nitrogen, redissolved with 300 µL of water and the solution of DNA conjugate was then desalted. DNA desalting and rebuffering was performed by charging the solution in an AMICON® filter unit from Sigma Aldrich (3 kD) in 300 µL of water, centrifuged at 4 °C and 10000 x g for at least 30 minutes, until the volume decreased to < 10 µL. Another 300 µL of water were added and the process was repeated all over again for at least 3 times. The remaining solution concentration was determined by A_{260} absorption using a Thermo Scientific™ NanoDrop™ One^C, concentration of the solution was adjusted to 2.0 mM and stored in the freezer at −20 °C.

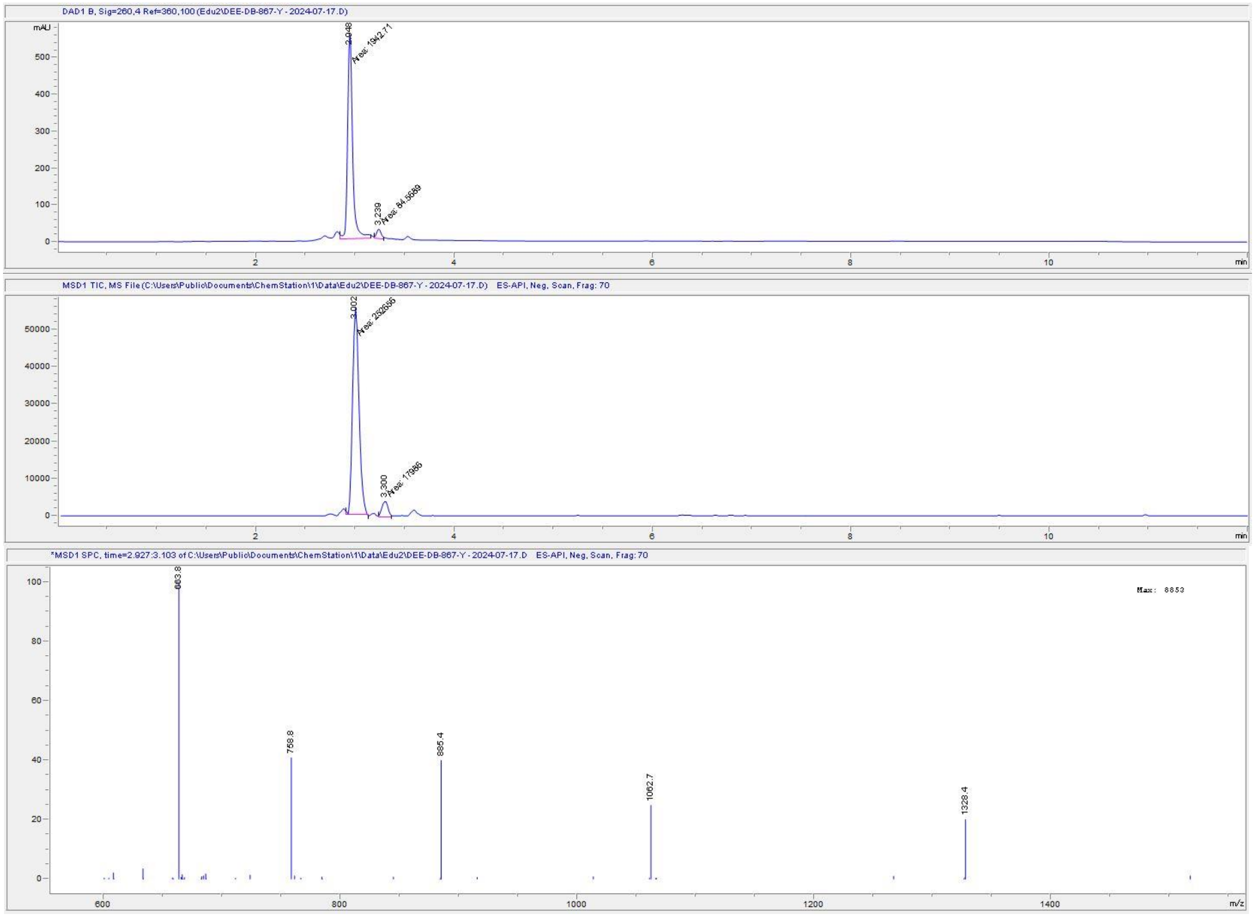

Figure S11. Analytical HPLC trace of **S14** with HPLC Method A. (Up) DAD chromatogram at 260 nm. (Middle) TIC chromatogram. (Below) Ionization of peak at 3.002 min containing reaction product.

DNA-conjugated arene S15

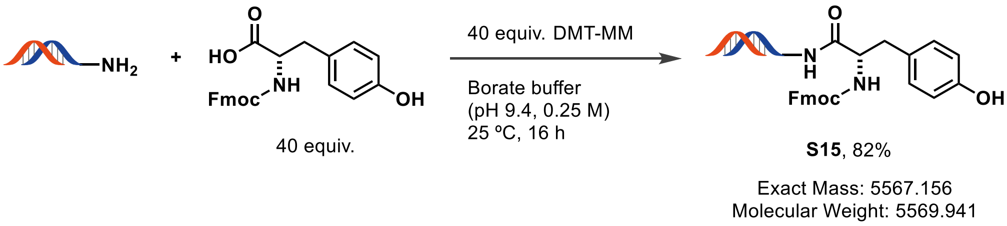

At 20–25 °C, 20 µL of HP–AOP–NH_2 (1.0 mM, 20 nmol, 1.0 equiv.) in borate buffer (pH 9.4, c = 250 mM) was added to a 1.5 mL Eppendorf tube. Next, 2.0 µL of an Fmoc-Tyr-OH stock solution (400 mM, 0.80 µmol, 40 equiv.) in DMA was added. The mixture was vortexed for 5 seconds. Then, 2.0 µL of a DMT-MM stock solution (400 mM, 0.80 µmol, 40 equiv.) in water was added. The mixture was vortexed for 5 seconds again, transferred into a Thermocycler at 25 °C, and incubated at 25 °C for 16 hours at 600 rpm. After 16 hours, an aliquot of 1 µL of the reaction mixture was diluted to 40 µL with water for LC–MS analysis.

Next, 2.4 µL of a 5 M solution of NaCl in water and 80 µL of ethanol at −20 °C were added to precipitate the DNA conjugate. The Eppendorf tube was placed in the freezer (−20 °C) for at least 1 hour, and then it was centrifuged at 4 °C and 10000 x g for at least 30 minutes. The supernatant was removed, the pellet was redissolved in 24 µL of water, and the procedure was repeated again. The remaining pellet was then dried under a flow of nitrogen, redissolved with 10 µL of water and stored in the freezer at −20 °C.

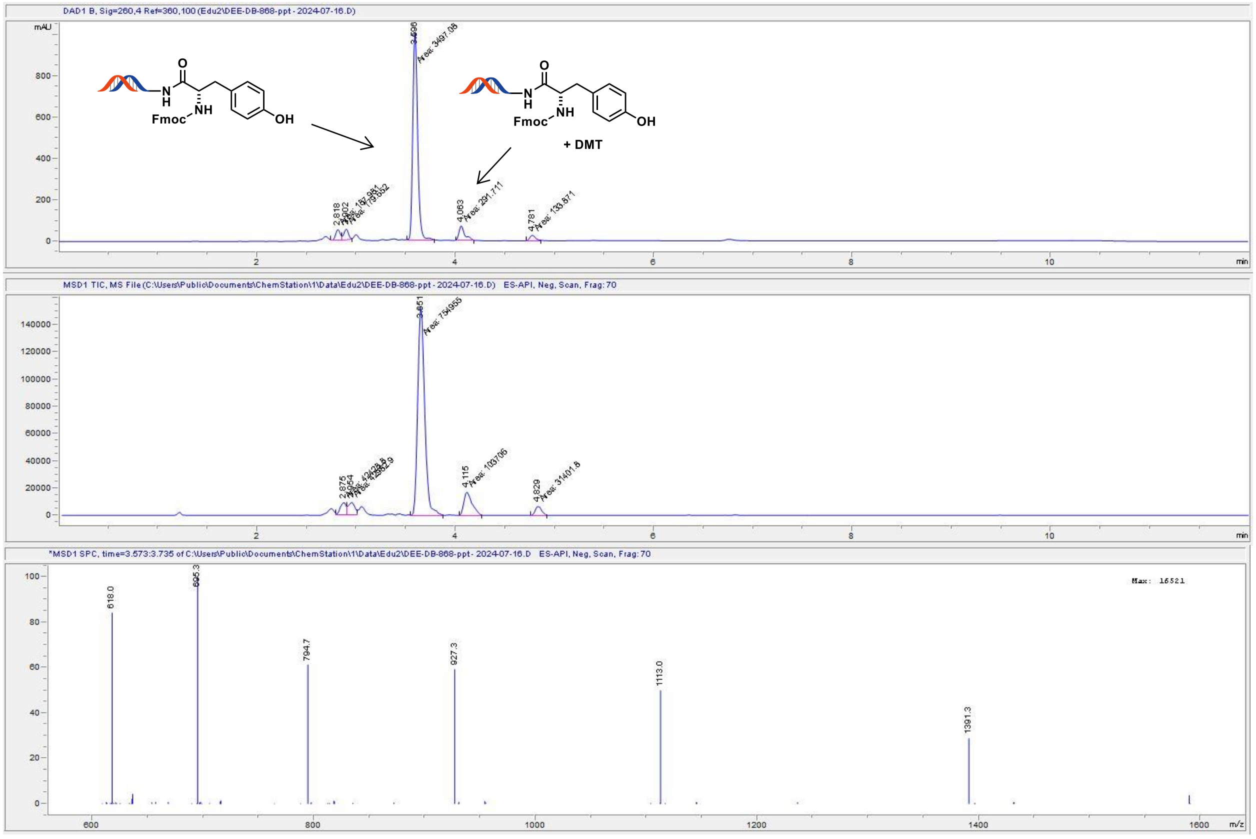

Figure S12. Analytical HPLC trace of **S15** with HPLC Method A. (Up) DAD chromatogram at 260 nm. (Middle) TIC chromatogram. (Below) Ionization of peak at 3.651 min containing reaction product.

DNA-conjugated arene S16

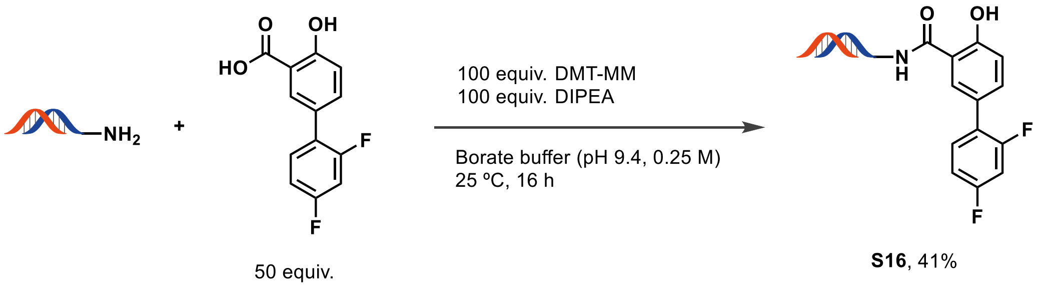

S16, 41%

Exact Mass: 5414.058
Molecular Weight: 5416.708

At 20–25 °C, 2.0 µL of a diflunisal stock solution (500 mM, 1.0 µmol, 50 equiv.) in DMA was mixed with 4.0 µL of a DMT-MM stock solution in DMA (500 mM, 2.0 µmol, 100 equiv.) in a 1.5 mL Eppendorf tube. The mixture was vortexed for 5 seconds. Next, 4.0 µL of a DIPEA stock solution (500 mM, 2.0 µmol, 100 equiv.) in DMA was added. The mixture was vortexed for 5 seconds again, and left standing at 20–25 °C for 20 min. In another 1.5 mL Eppendorf tube, 20 µL of HP–AOP–NH_2 (1.0 mM, 20 nmol, 1.0 equiv.) in borate buffer (pH 9.4, c = 250 mM) was added, and the premix of acid, DMT-MM and DIPEA was added over the solution. The mixture was vortexed for 5 seconds, transferred into a Thermocycler at 25 °C, and incubated at 25 °C for 16 hours at 600 rpm. After 16 hours, an aliquot of 1 µL of the reaction mixture was diluted to 40 µL with water for LC–MS analysis.

Next, 2.6 µL of a 5 M solution of NaCl in water and 80 µL of ethanol at −20 °C were added to precipitate the DNA conjugate. The Eppendorf tube was placed in the freezer (−20 °C) for at least 1 hour, and then it was centrifuged at 4 °C and 10000 x g for at least 30 minutes. The supernatant was removed, the pellet

was redissolved in 26 µL of water, and the procedure was repeated again. The remaining pellet was then dried under a flow of nitrogen, redissolved with 10 µL of water and stored in the freezer at −20 °C.

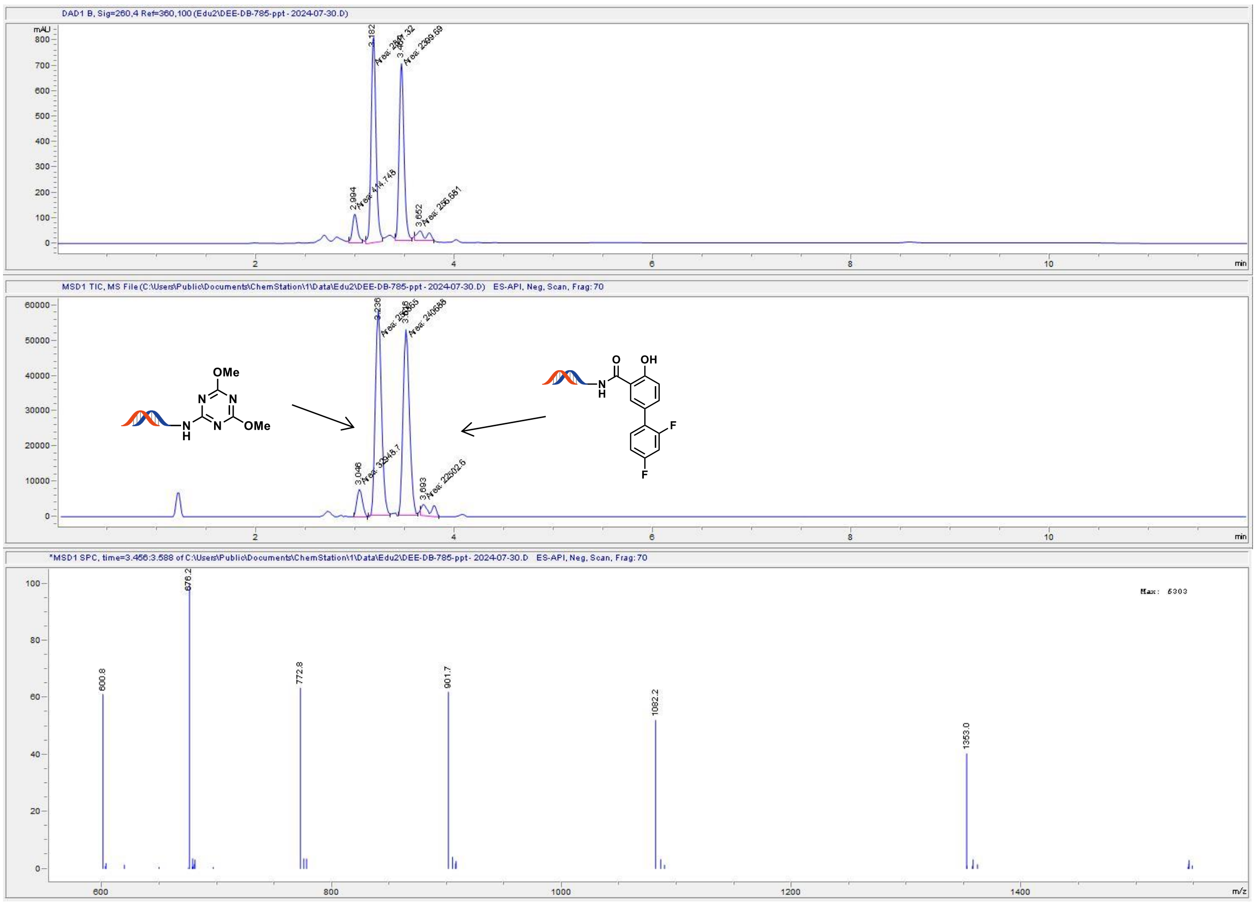

Figure S13. Analytical HPLC trace of **S16** with HPLC Method A. (Up) DAD chromatogram at 260 nm. (Middle) TIC chromatogram. (Below) Ionization of peak at 3.516 min containing reaction product.

DNA-conjugated arene S17

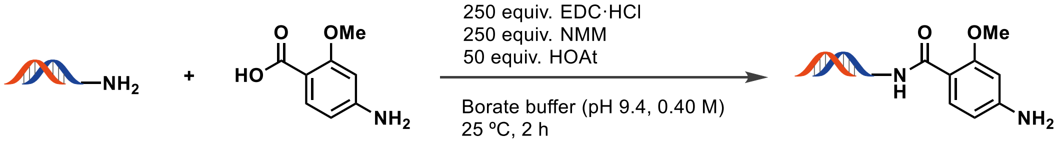

500 equiv.

S17, >95%

Exact Mass: 5331.072
Molecular Weight: 5333.671

At 20–25 °C, 20 µL of a 4-amino-2-methoxybenzoic acid stock solution (500 mM, 10 µmol, 500 equiv.) in DMSO was mixed with 10 µL of a HOAt stock solution in DMSO (100 mM, 1.0 µmol, 50 equiv.), and 10 µL of an EDC·HCl stock solution in DMSO (500 mM, 5.0 µmol, 250 equiv.) in a 1.5 mL Eppendorf tube. The mixture was vortexed for 5 seconds. Next, 10 µL of a NMM stock solution (500 mM, 5.0 µmol, 250 equiv.) in DMSO was added. The mixture was vortexed for 5 seconds again, and left standing at 20–25 °C for 15 min. In another 1.5 mL Eppendorf tube, 60 µL of HP–AOP–NH_2 (0.33 mM, 20 nmol, 1.0 equiv.) in borate buffer (pH 9.4, c = 400 mM) was added, and the premix of acid, HOAt, EDC·HCl and NMM was added over the solution. The mixture was vortexed for 5 seconds, transferred into a Thermocycler at 25 °C, and incubated at 25 °C for 2 hours at 600 rpm. After 2 hours, an aliquot of 1 µL of the reaction mixture was diluted to 40 µL with water for LC–MS analysis.

Next, 10 µL of a 5 M solution of NaCl in water and 360 µL of ethanol at −20 °C were added to precipitate the DNA conjugate. The Eppendorf tube was placed in the freezer (−20 °C) for at least 1 hour, and then it was centrifuged at 4 °C and 10000 x g for at least 30 minutes. The supernatant was removed, the pellet was redissolved in 300 µL of water, and the procedure was repeated again. The remaining pellet was then dried under a flow of nitrogen, redissolved with 300 µL of water and the solution of DNA conjugate was then desalted. DNA desalting and rebuffering was performed by charging the solution in an AMICON® filter unit from Sigma Aldrich (3 kD) in 300 µL of water, centrifuged at 4 °C and 10000 x g for at least 30 minutes, until the volume decreased to < 10 µL. Another 300 µL of water were added and the process was repeated all over again for at least 3 times. The remaining solution concentration was determined by A_{260} absorption using a Thermo Scientific™ NanoDrop™ One^{C}, concentration of the solution was adjusted to 2.0 mM and stored in the freezer at −20 °C.

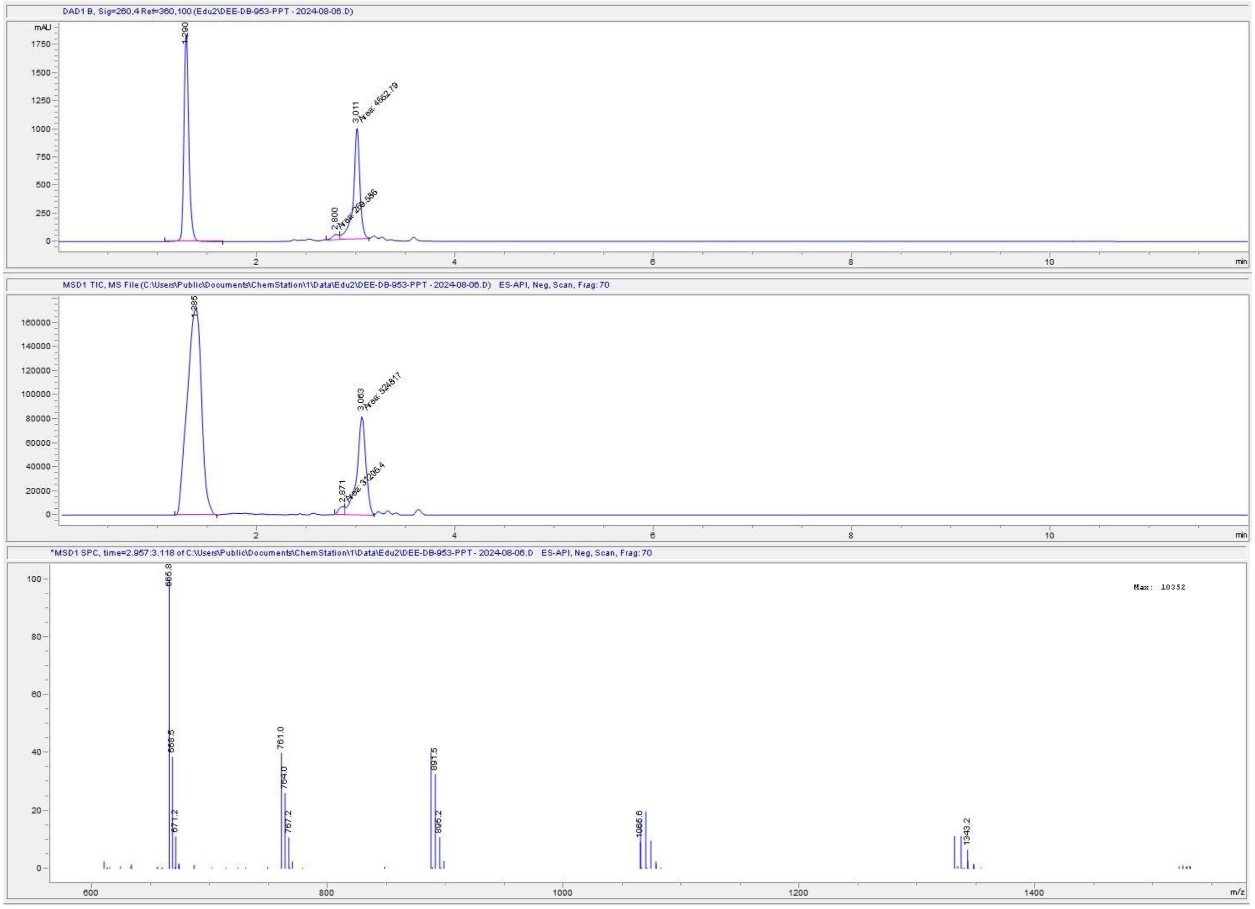

Figure S14. Analytical HPLC trace of **S17** with HPLC Method A. (Up) DAD chromatogram at 260 nm. (Middle) TIC chromatogram. (Below) Ionization of peak at 3.063 min containing reaction product.

DNA-conjugated arene S18

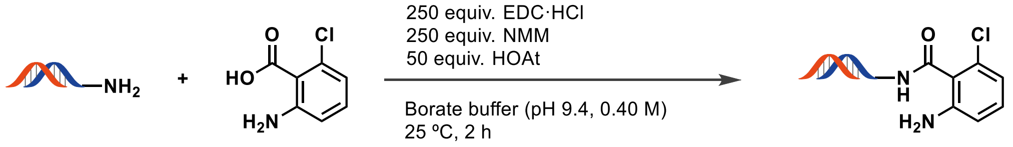

500 equiv.

S18, >95%

Exact Mass: 5335.023
Molecular Weight: 5338.087

At 20–25 °C, 20 µL of a 2-amino-6-chlorobenzoic stock solution (500 mM, 10 µmol, 500 equiv.) in DMSO was mixed with 10 µL of a HOAt stock solution in DMSO (100 mM, 1.0 µmol, 50 equiv.), and 10 µL of an

EDC·HCl stock solution in DMSO (500 mM, 5.0 µmol, 250 equiv.) in a 1.5 mL Eppendorf tube. The mixture was vortexed for 5 seconds. Next, 10 µL of a NMM stock solution (500 mM, 5.0 µmol, 250 equiv.) in DMSO was added. The mixture was vortexed for 5 seconds again, and left standing at 20–25 °C for 15 min. In another 1.5 mL Eppendorf tube, 60 µL of HP–AOP–NH_2 (0.33 mM, 20 nmol, 1.0 equiv.) in borate buffer (pH 9.4, c = 400 mM) was added, and the premix of acid, HOAt, EDC·HCl and NMM was added over the solution. The mixture was vortexed for 5 seconds, transferred into a Thermocycler at 25 °C, and incubated at 25 °C for 2 hours at 600 rpm. After 2 hours, an aliquot of 1 µL of the reaction mixture was diluted to 40 µL with water for LC–MS analysis.

Next, 10 µL of a 5 M solution of NaCl in water and 360 µL of ethanol at −20 °C were added to precipitate the DNA conjugate. The Eppendorf tube was placed in the freezer (−20 °C) for at least 1 hour, and then it was centrifuged at 4 °C and 10000 x g for at least 30 minutes. The remaining pellet was then dried under a flow of nitrogen, redissolved with 300 µL of water and the solution of DNA conjugate was then desalted. DNA desalting and rebuffering was performed by charging the solution in an AMICON® filter unit from Sigma Aldrich (3 kD) in 300 µL of water, centrifuged at 4 °C and 10000 x g for at least 30 minutes, until the volume decreased to < 10 µL. Another 300 µL of water were added and the process was repeated all over again for at least 3 times. The remaining solution concentration was determined by A_{260} absorption using a Thermo Scientific™ NanoDrop™ One^{C}, concentration of the solution was adjusted to 2.0 mM and stored in the freezer at −20 °C.

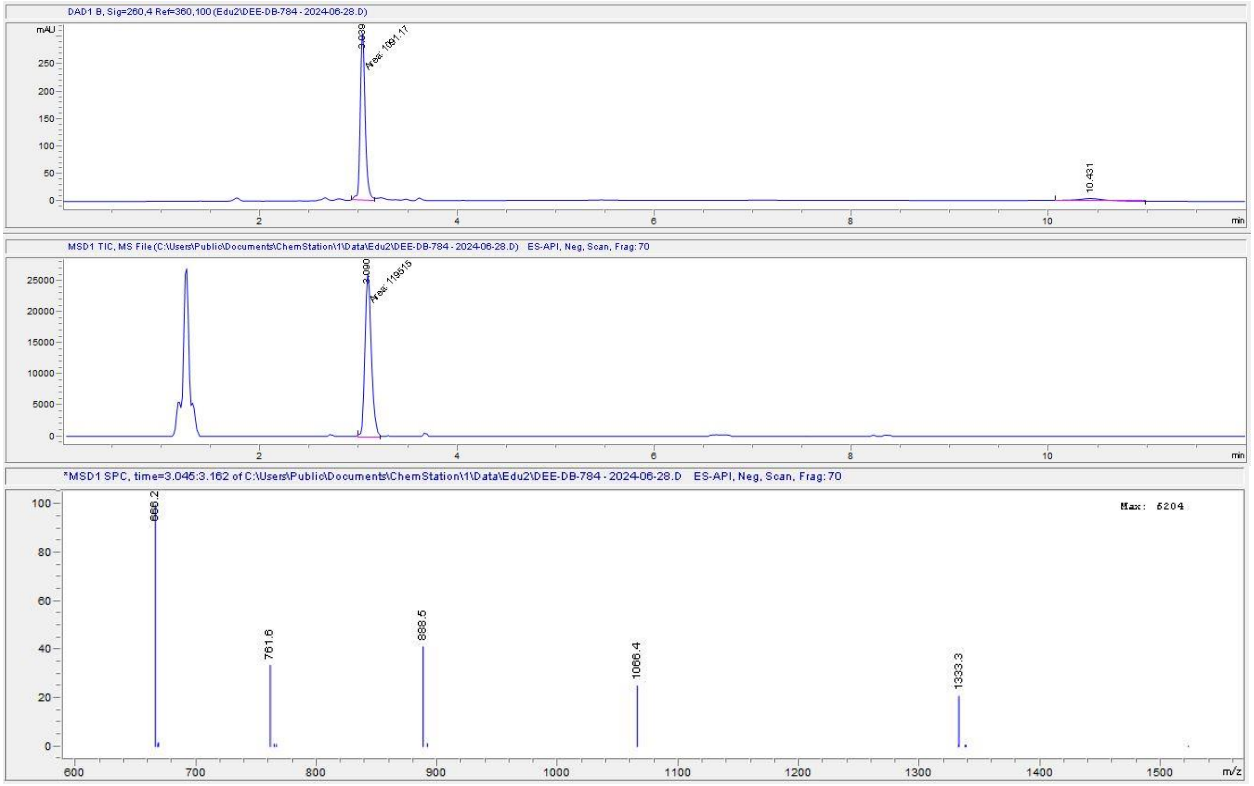

Figure S15. Analytical HPLC trace of **S18** with HPLC Method A. (Up) DAD chromatogram at 260 nm. (Middle) TIC chromatogram. (Below) Ionization of peak at 3.090 min containing reaction product.

DNA-conjugated arene S19

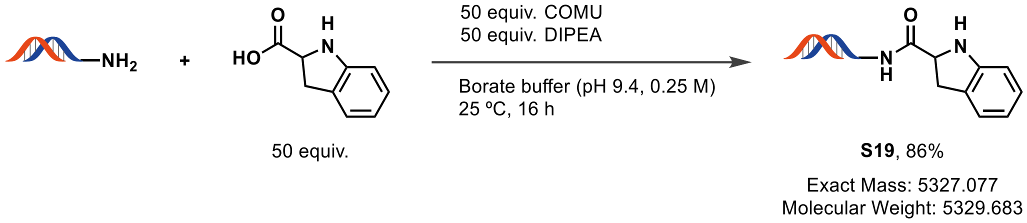

At 20–25 °C, 2.0 µL of an indoline-2-carboxylic acid stock solution (500 mM, 1.0 µmol, 50 equiv.) in DMA was mixed with 2.0 µL of a COMU stock solution in DMA (500 mM, 1.0 µmol, 50 equiv.) in a 1.5 mL Eppendorf tube. The mixture was vortexed for 5 seconds. Next, 2.0 µL of a DIPEA stock solution (500 mM, 1.0 µmol, 50 equiv.) in DMA was added. The mixture was vortexed for 5 seconds again, and left standing at 20–25 °C for 20 min. In another 1.5 mL Eppendorf tube, 20 µL of HP–AOP–NH_2 (1.0 mM, 20 nmol, 1.0 equiv.) in borate buffer (pH 9.4, c = 250 mM) was added, and the premix of acid, COMU and DIPEA was added over the solution. The mixture was vortexed for 5 seconds, transferred into a Thermocycler at 25 °C, and incubated at 25 °C for 16 hours at 600 rpm. After 16 hours, an aliquot of 1 µL of the reaction mixture was diluted to 40 µL with water for LC–MS analysis.

Next, 2.6 µL of a 5 M solution of NaCl in water and 80 µL of ethanol at −20 °C were added to precipitate the DNA conjugate. The Eppendorf tube was placed in the freezer (−20 °C) for at least 1 hour, and then it was centrifuged at 4 °C and 10000 x g for at least 30 minutes. The supernatant was removed, the pellet was redissolved in 26 µL of water, and the procedure was repeated again. The remaining pellet was then dried under a flow of nitrogen, redissolved with 10 µL of water and stored in the freezer at −20 °C.

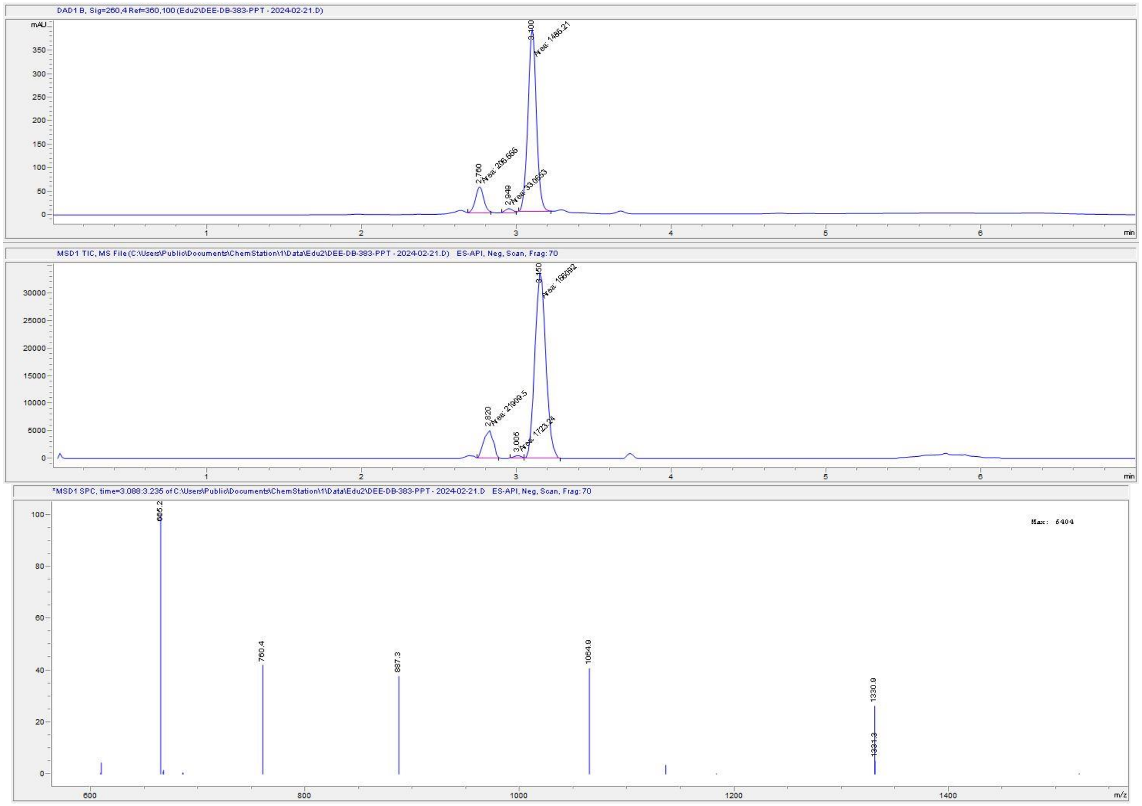

Figure S16. Analytical HPLC trace of **S19** with HPLC Method B. (Up) DAD chromatogram at 260 nm. (Middle) TIC chromatogram. (Below) Ionization of peak at 3.150 min containing reaction product.

DNA-conjugated arene S20

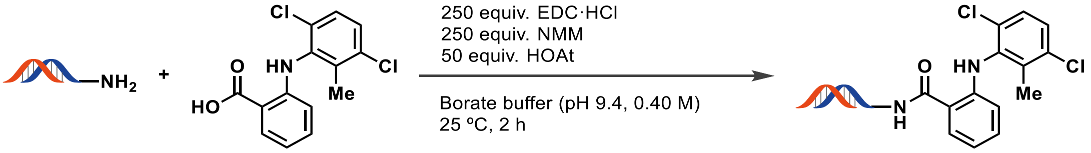

S20, >95%

Exact Mass: 5459.031
Molecular Weight: 5462.654

At 20–25 °C, 20 µL of a meclofenamate sodium salt stock solution (500 mM, 10 µmol, 500 equiv.) in DMSO was mixed with 10 µL of a HOAt stock solution in DMSO (100 mM, 1.0 µmol, 50 equiv.), and 10 µL of an EDC·HCl stock solution in DMSO (500 mM, 5.0 µmol, 250 equiv.) in a 1.5 mL Eppendorf tube. The mixture was vortexed for 5 seconds. Next, 10 µL of a NMM stock solution (500 mM, 5.0 µmol, 250 equiv.) in DMSO was added. The mixture was vortexed for 5 seconds again, and left standing at 20–25 °C for 15 min. In another 1.5 mL Eppendorf tube, 60 µL of HP–AOP–NH_2 (0.33 mM, 20 nmol, 1.0 equiv.) in borate buffer (pH 9.4, c = 400 mM) was added, and the premix of acid, HOAt, EDC·HCl and NMM was added over the solution. The mixture was vortexed for 5 seconds, transferred into a Thermocycler at 25 °C, and incubated at 25 °C for 2 hours at 600 rpm. After 2 hours, an aliquot of 1 µL of the reaction mixture was diluted to 40 µL with water for LC–MS analysis.

Next, 10 µL of a 5 M solution of NaCl in water and 360 µL of ethanol at −20 °C were added to precipitate the DNA conjugate. The Eppendorf tube was placed in the freezer (−20 °C) for at least 1 hour, and then it was centrifuged at 4 °C and 10000 x g for at least 30 minutes. The supernatant was removed, the pellet was redissolved in 300 µL of water, and the procedure was repeated again. The remaining pellet was then dried under a flow of nitrogen, redissolved with 300 µL of water and the solution of DNA conjugate was then desalted. DNA desalting and rebuffering was performed by charging the solution in an AMICON® filter unit from Sigma Aldrich (3 kD) in 300 µL of water, centrifuged at 4 °C and 10000 x g for at least 30 minutes, until the volume decreased to < 10 µL. Another 300 µL of water were added and the process was repeated all over again for at least 3 times. The remaining solution concentration was determined by A_{260} absorption using a Thermo Scientific™ NanoDrop™ One^{C}, concentration of the solution was adjusted to 2.0 mM and stored in the freezer at −20 °C.

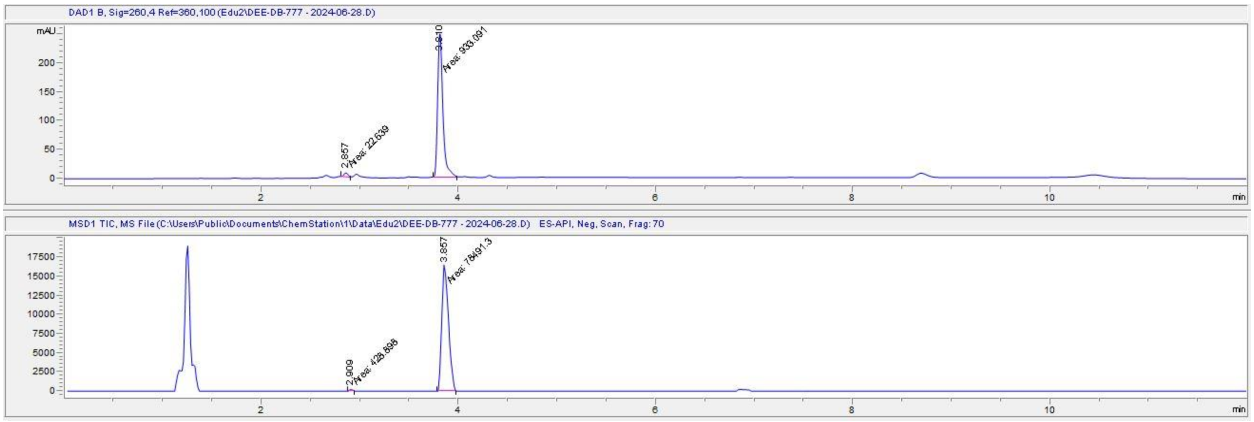

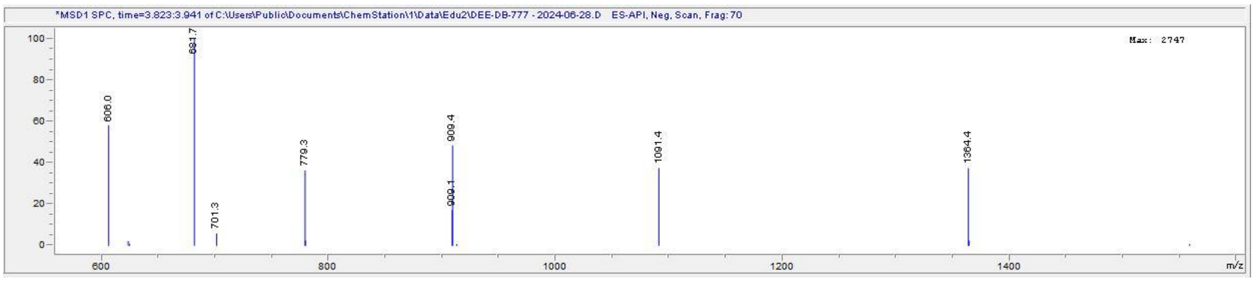

Figure S17. Analytical HPLC trace of **S20** with HPLC Method A. (Up) DAD chromatogram at 260 nm. (Middle) TIC chromatogram. (Below) Ionization of peak at 3.857 min containing reaction product.

DNA-conjugated arene S21

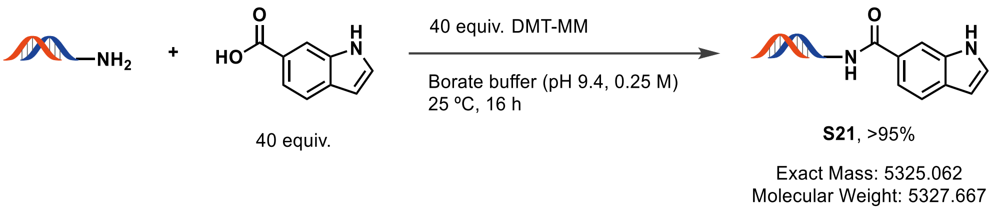

At 20–25 °C, 20 µL of HP–AOP–NH_2 (1.0 mM, 20 nmol, 1.0 equiv.) in borate buffer (pH 9.4, c = 250 mM) was added to a 1.5 mL Eppendorf tube. Next, 2.0 µL of an indole-6-carboxylic acid stock solution (400 mM, 0.80 µmol, 40 equiv.) in DMA was added. The mixture was vortexed for 5 seconds. Then, 2.0 µL of a DMT-MM stock solution (400 mM, 0.80 µmol, 40 equiv.) in water was added. The mixture was vortexed for 5 seconds again, transferred into a Thermocycler at 25 °C, and incubated at 25 °C for 16 hours at 600 rpm. After 16 hours, an aliquot of 1 µL of the reaction mixture was diluted to 40 µL with water for LC–MS analysis.

Next, 2.4 µL of a 5 M solution of NaCl in water and 80 µL of ethanol at −20 °C were added to precipitate the DNA conjugate. The Eppendorf tube was placed in the freezer (−20 °C) for at least 1 hour, and then it was centrifuged at 4 °C and 10000 x g for at least 30 minutes. The supernatant was removed, the pellet was redissolved in 24 µL of water, and the procedure was repeated again. The remaining pellet was then dried under a flow of nitrogen, redissolved with 10 µL of water and stored in the freezer at −20 °C.

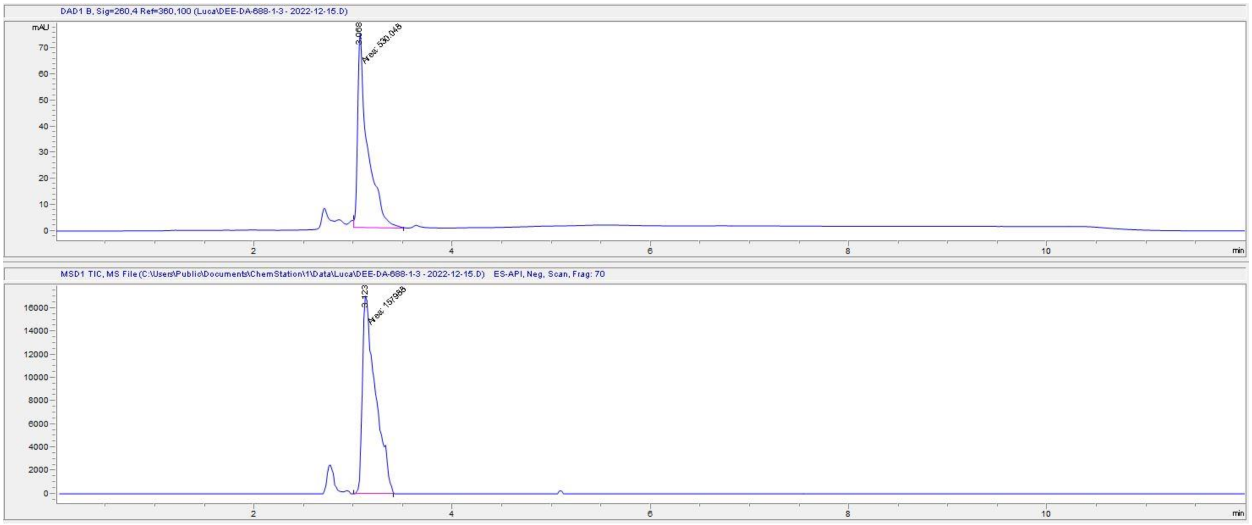

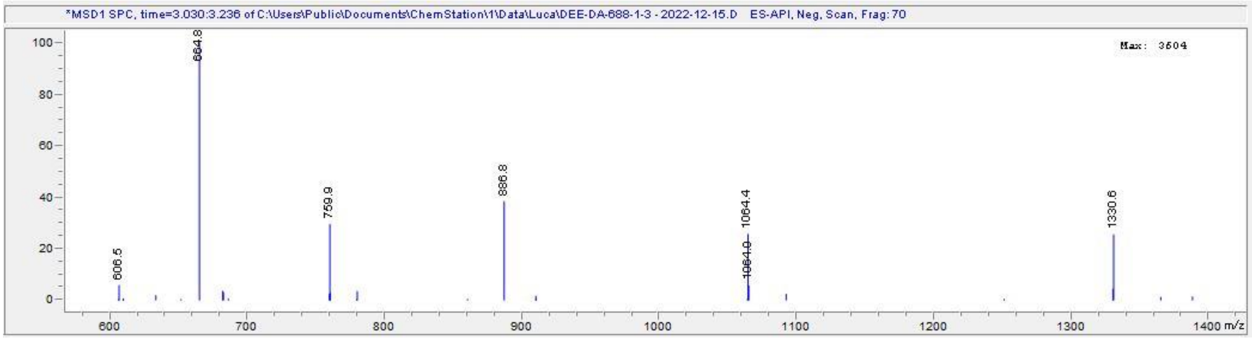

Figure S18. Analytical HPLC trace of **S21** with HPLC Method A. (Up) DAD chromatogram at 260 nm. (Middle) TIC chromatogram. (Below) Ionization of peak at 3.123 min containing reaction product.

DNA-conjugated arene S22

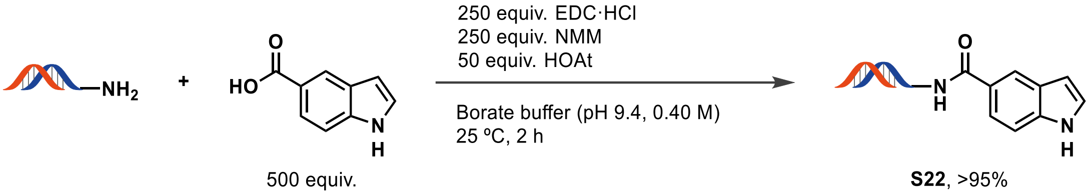

S22, >95%

Exact Mass: 5325.062
Molecular Weight: 5327.667

At 20–25 °C, 20 µL of a indole-5-carboxylic acid stock solution (500 mM, 10 µmol, 500 equiv.) in DMSO was mixed with 10 µL of a HOAt stock solution in DMSO (100 mM, 1.0 µmol, 50 equiv.), and 10 µL of an EDC·HCl stock solution in DMSO (500 mM, 5.0 µmol, 250 equiv.) in a 1.5 mL Eppendorf tube. The mixture was vortexed for 5 seconds. Next, 10 µL of a NMM stock solution (500 mM, 5.0 µmol, 250 equiv.) in DMSO was added. The mixture was vortexed for 5 seconds again, and left standing at 20–25 °C for 15 min. In another 1.5 mL Eppendorf tube, 60 µL of HP–AOP–NH_2 (0.33 mM, 20 nmol, 1.0 equiv.) in borate buffer (pH 9.4, c = 400 mM) was added, and the premix of acid, HOAt, EDC·HCl and NMM was added over the solution. The mixture was vortexed for 5 seconds, transferred into a Thermocycler at 25 °C, and incubated at 25 °C for 2 hours at 600 rpm. After 2 hours, an aliquot of 1 µL of the reaction mixture was diluted to 40 µL with water for LC–MS analysis.

Next, 10 µL of a 5 M solution of NaCl in water and 360 µL of ethanol at −20 °C were added to precipitate the DNA conjugate. The Eppendorf tube was placed in the freezer (−20 °C) for at least 1 hour, and then it was centrifuged at 4 °C and 10000 x g for at least 30 minutes. The supernatant was removed, the pellet was redissolved in 300 µL of water, and the procedure was repeated again. The remaining pellet was then dried under a flow of nitrogen, redissolved with 300 µL of water and the solution of DNA conjugate was then desalted. DNA desalting and rebuffering was performed by charging the solution in an AMICON® filter unit from Sigma Aldrich (3 kD) in 300 µL of water, centrifuged at 4 °C and 10000 x g for at least 30 minutes, until the volume decreased to < 10 µL. Another 300 µL of water were added and the process was repeated all over again for at least 3 times. The remaining solution concentration was determined by A_{260} absorption using a Thermo Scientific™ NanoDrop™ One^{C}, concentration of the solution was adjusted to 2.0 mM and stored in the freezer at −20 °C.

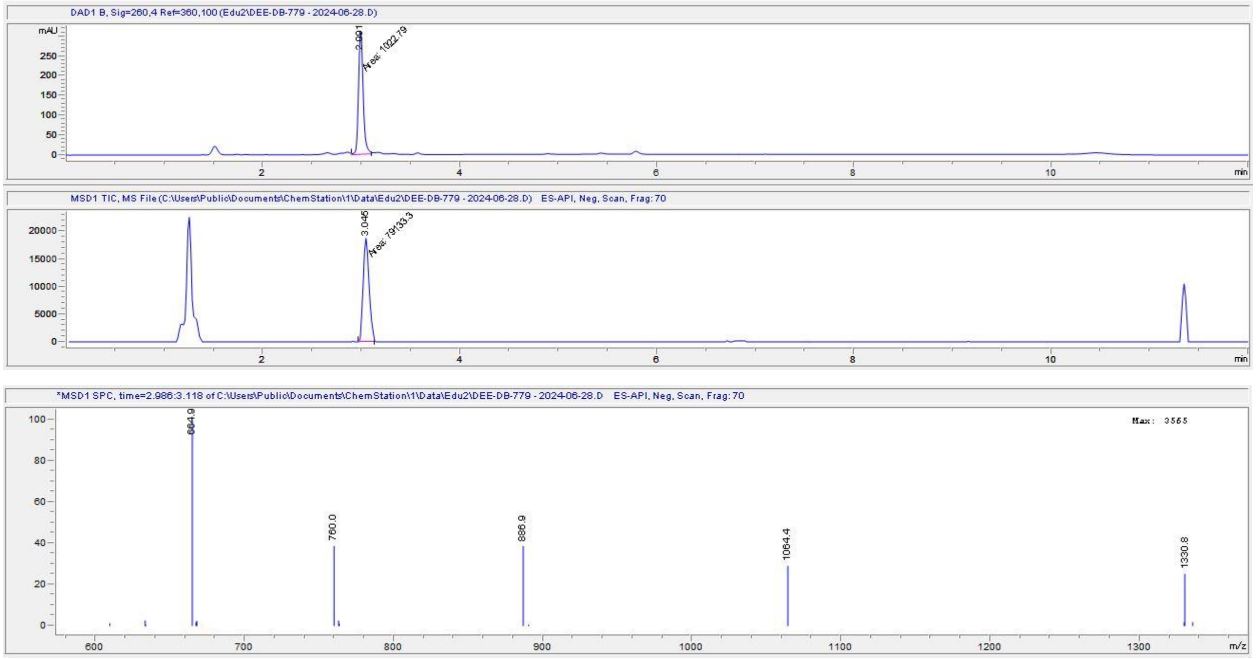

Figure S19. Analytical HPLC trace of **S22** with HPLC Method A. (Up) DAD chromatogram at 260 nm. (Middle) TIC chromatogram. (Below) Ionization of peak at 3.045 min containing reaction product.

DNA-conjugated arene S23

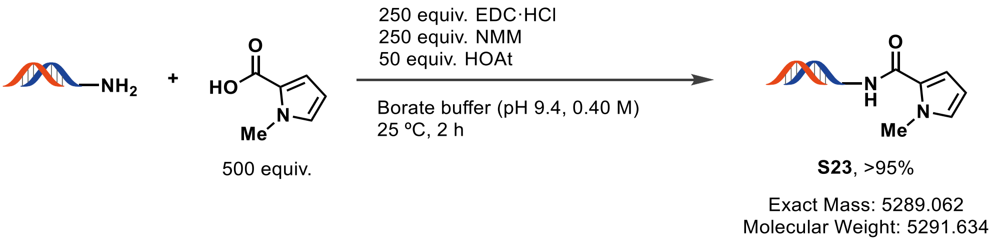

At 20–25 °C, 20 µL of a *N*-methyl-2-pyrrolecarboxylic stock solution (500 mM, 10 µmol, 500 equiv.) in DMSO was mixed with 10 µL of a HOAt stock solution in DMSO (100 mM, 1.0 µmol, 50 equiv.), and 10 µL of an EDC·HCl stock solution in DMSO (500 mM, 5.0 µmol, 250 equiv.) in a 1.5 mL Eppendorf tube. The mixture was vortexed for 5 seconds. Next, 10 µL of a NMM stock solution (500 mM, 5.0 µmol, 250 equiv.) in DMSO was added. The mixture was vortexed for 5 seconds again, and left standing at 20–25 °C for 15 min. In another 1.5 mL Eppendorf tube, 60 µL of HP–AOP–NH_2 (0.33 mM, 20 nmol, 1.0 equiv.) in borate buffer (pH 9.4, c = 400 mM) was added, and the premix of acid, HOAt, EDC·HCl and NMM was added over the solution. The mixture was vortexed for 5 seconds, transferred into a Thermocycler at 25 °C, and incubated at 25 °C for 2 hours at 600 rpm. After 2 hours, an aliquot of 1 µL of the reaction mixture was diluted to 40 µL with water for LC–MS analysis.

Next, 10 µL of a 5 M solution of NaCl in water and 360 µL of ethanol at −20 °C were added to precipitate the DNA conjugate. The Eppendorf tube was placed in the freezer (−20 °C) for at least 1 hour, and then it was centrifuged at 4 °C and 10000 x g for at least 30 minutes. The supernatant was removed, the pellet was redissolved in 300 µL of water, and the procedure was repeated again. The remaining pellet was then dried under a flow of nitrogen, redissolved with 300 µL of water and the solution of DNA conjugate was then desalted. DNA desalting and rebuffering was performed by charging the solution in an AMICON® filter unit from Sigma Aldrich (3 kD) in 300 µL of water, centrifuged at 4 °C and 10000 x g for at least 30 minutes, until the volume decreased to < 10 µL. Another 300 µL of water were added and the

process was repeated all over again for at least 3 times. The remaining solution concentration was determined by A_{260} absorption using a Thermo Scientific™ NanoDrop™ One^{C}, concentration of the solution was adjusted to 2.0 mM and stored in the freezer at −20 °C.

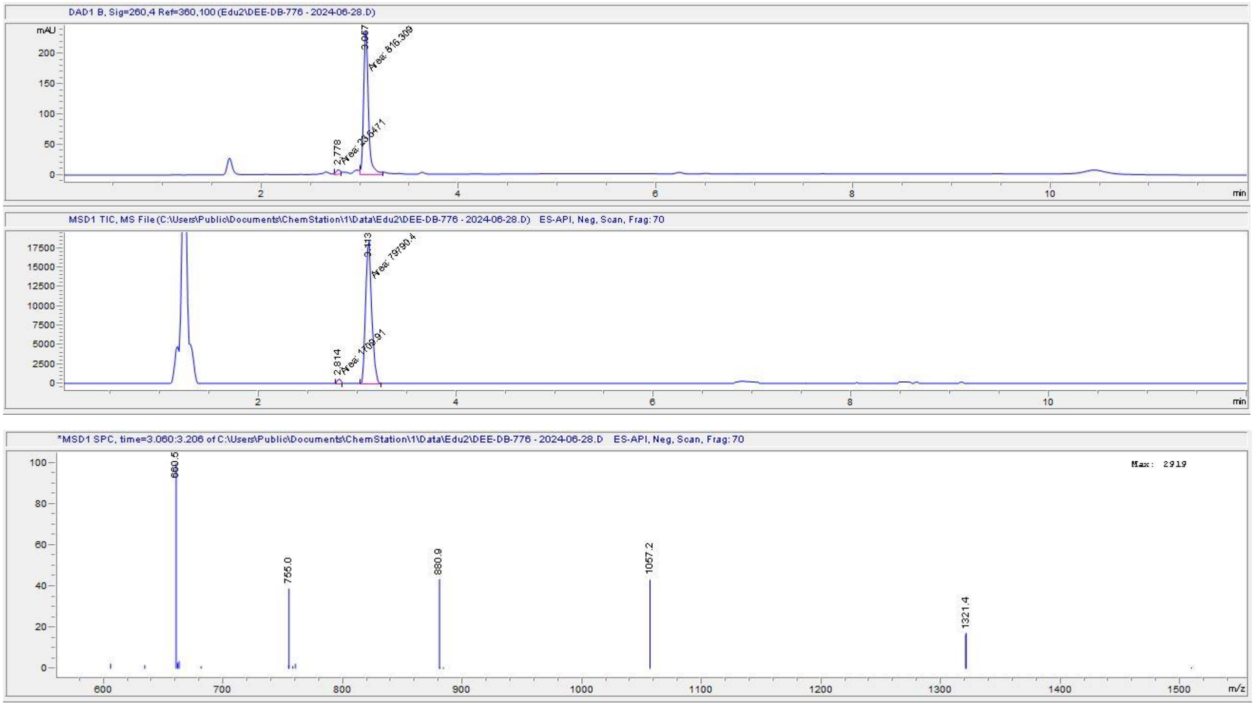

Figure S20. Analytical HPLC trace of **S23** with HPLC Method A. (Up) DAD chromatogram at 260 nm. (Middle) TIC chromatogram. (Below) Ionization of peak at 3.113 min containing reaction product.

DNA-conjugated arene S24

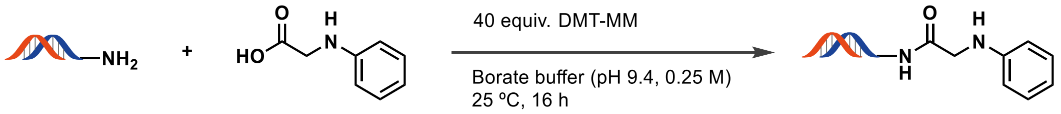

40 equiv.

S24, >95%

Exact Mass: 5315.077
Molecular Weight: 5317.672

At 20–25 °C, 20 µL of HP–AOP–NH_2 (1.0 mM, 20 nmol, 1.0 equiv.) in borate buffer (pH 9.4, c = 250 mM) was added to a 1.5 mL Eppendorf tube. Next, 2.0 µL of a *N*-phenylglycine stock solution (400 mM, 0.80 µmol, 40 equiv.) in DMA was added. The mixture was vortexed for 5 seconds. Then, 2.0 µL of a DMT-MM stock solution (400 mM, 0.80 µmol, 40 equiv.) in water was added. The mixture was vortexed for 5 seconds again, transferred into a Thermocycler at 25 °C, and incubated at 25 °C for 16 hours at 600 rpm. After 16 hours, an aliquot of 1 µL of the reaction mixture was diluted to 40 µL with water for LC–MS analysis.

Next, 2.4 µL of a 5 M solution of NaCl in water and 80 µL of ethanol at −20 °C were added to precipitate the DNA conjugate. The Eppendorf tube was placed in the freezer (−20 °C) for at least 1 hour, and then it was centrifuged at 4 °C and 10000 x g for at least 30 minutes. The supernatant was removed, the pellet was redissolved in 24 µL of water, and the procedure was repeated again. The remaining pellet was then dried under a flow of nitrogen, redissolved with 10 µL of water and stored in the freezer at −20 °C.

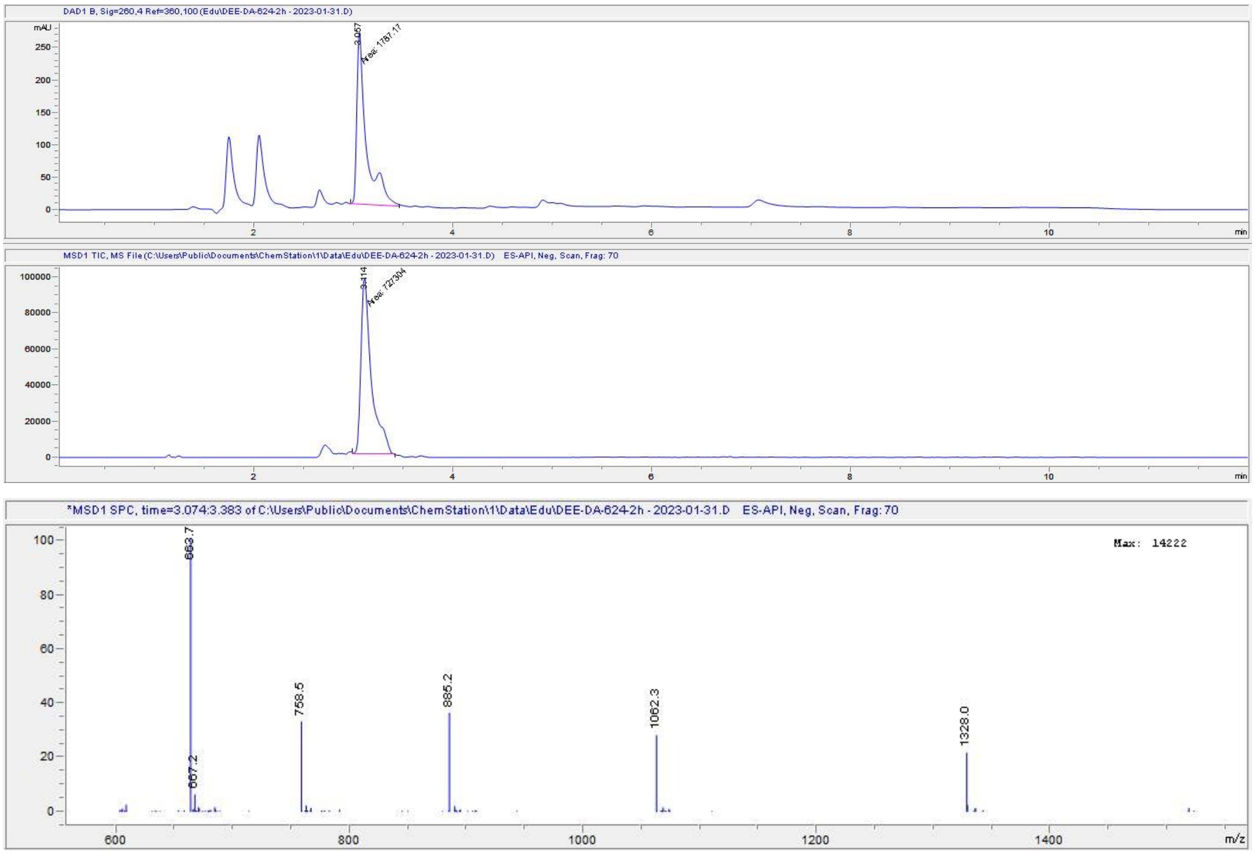

Figure S21. Analytical HPLC trace of **S24** with HPLC Method A. (Up) DAD chromatogram at 260 nm. (Middle) TIC chromatogram. (Below) Ionization of peak at 3.114 min containing reaction product.

DNA-conjugated arene S25

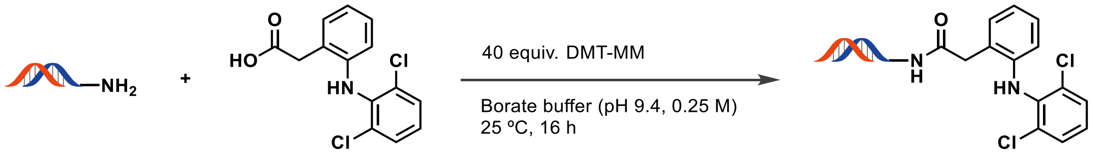

40 equiv.

S25, >95%

Exact Mass: 5459.031
Molecular Weight: 5462.654

At 20–25 °C, 400 µL of HP–AOP–NH_2 (1.0 mM, 400 nmol, 1.0 equiv.) in borate buffer (pH 9.4, c = 250 mM) was added to a 1.5 mL Eppendorf tube. Next, 40 µL of a diclofenac stock solution (400 mM, 16 µmol, 40 equiv.) in DMA was added. The mixture was vortexed for 5 seconds. Then, 40 µL of a DMT-MM stock solution (400 mM, 16 µmol, 40 equiv.) in water was added. The mixture was vortexed for 5 seconds again, transferred into a Thermocycler at 25 °C, and incubated at 25 °C for 16 hours at 600 rpm. After 16 hours, an aliquot of 1 µL of the reaction mixture was diluted to 40 µL with water for LC–MS analysis.

After 16 hours, 48 µL of a 5 M solution of NaCl in water and 1.6 mL of ethanol at −20 °C were added to precipitate the DNA conjugate. The Eppendorf tube was placed in the freezer (−20 °C) for at least 1 hour, and then it was centrifuged at 4 °C and 10000 x g for at least 30 minutes. The supernatant was removed, the pellet was redissolved in 480 µL of water, and the procedure was repeated again. The remaining pellet was then dried under a flow of nitrogen, redissolved with 200 µL of water and stored in the freezer at −20 °C.

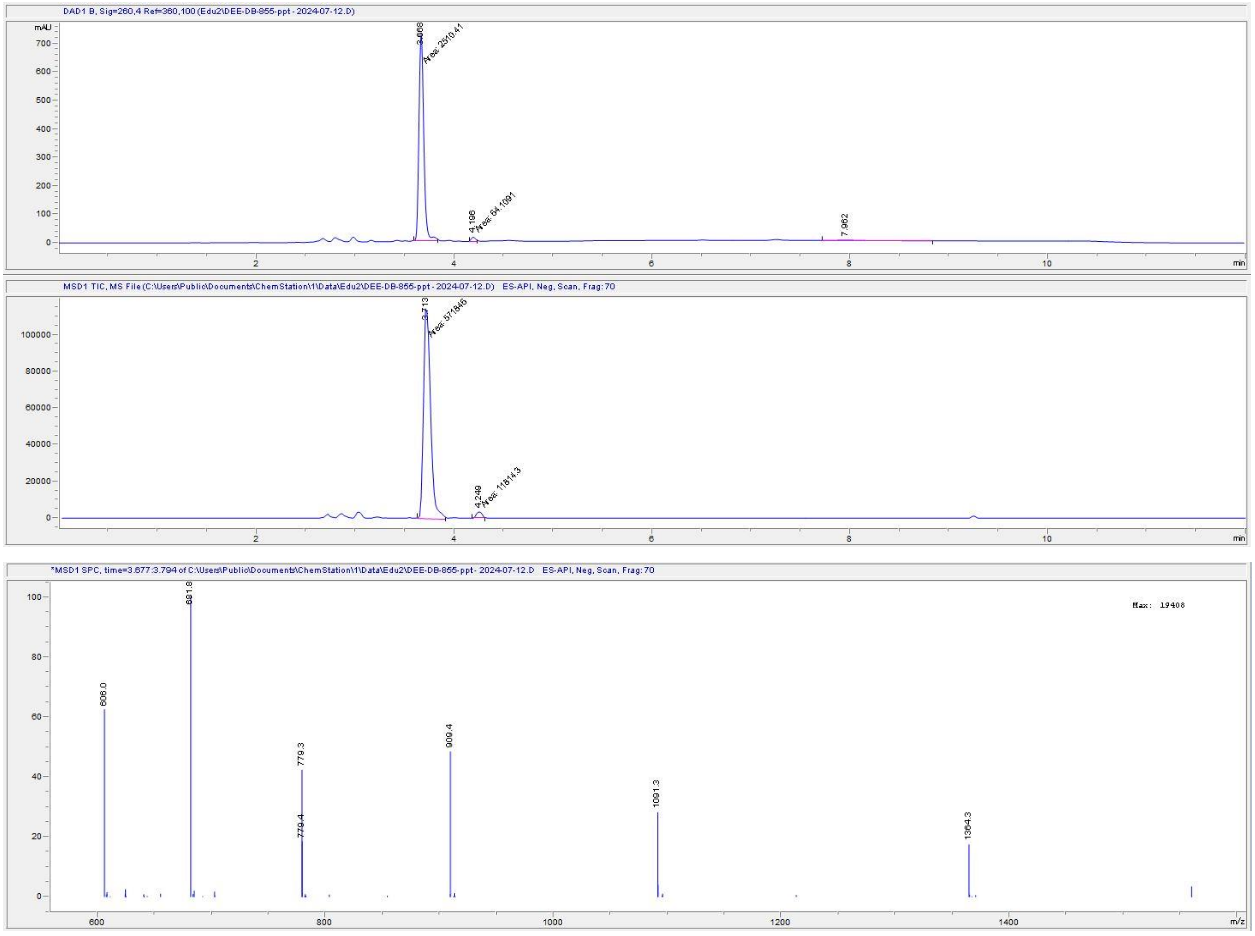

Figure S22. Analytical HPLC trace of **S25** with HPLC Method A. (Up) DAD chromatogram at 260 nm. (Middle) TIC chromatogram. (Below) Ionization of peak at 3.713 min containing reaction product.

DNA-conjugated arene S26

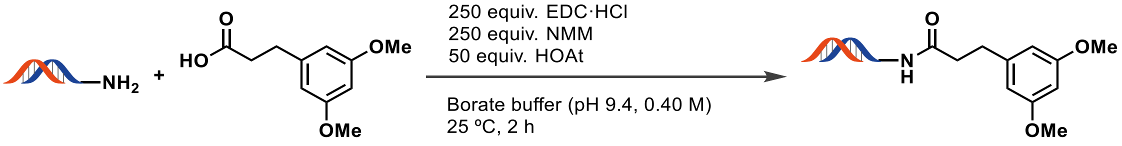

500 equiv.

S26, >95%

Exact Mass: 5374.103
Molecular Weight: 5376.736

At 20–25 °C, 20 µL of a 3-(3,5-dimethoxyphenyl)propanoic stock solution (500 mM, 10 µmol, 500 equiv.) in DMSO was mixed with 10 µL of a HOAt stock solution in DMSO (100 mM, 1.0 µmol, 50 equiv.), and 10 µL of an EDC·HCl stock solution in DMSO (500 mM, 5.0 µmol, 250 equiv.) in a 1.5 mL Eppendorf tube. The mixture was vortexed for 5 seconds. Next, 10 µL of a NMM stock solution (500 mM, 5.0 µmol, 250 equiv.) in DMSO was added. The mixture was vortexed for 5 seconds again, and left standing at 20–25 °C for 15 min. In another 1.5 mL Eppendorf tube, 60 µL of HP–AOP–NH_2 (0.33 mM, 20 nmol, 1.0 equiv.) in borate buffer (pH 9.4, c = 400 mM) was added, and the premix of acid, HOAt, EDC·HCl and NMM was added over the solution. The mixture was vortexed for 5 seconds, transferred into a Thermocycler at 25 °C, and incubated at 25 °C for 2 hours at 600 rpm. After 2 hours, an aliquot of 1 µL of the reaction mixture was diluted to 40 µL with water for LC–MS analysis.

Next, 10 µL of a 5 M solution of NaCl in water and 360 µL of ethanol at −20 °C were added to precipitate the DNA conjugate. The Eppendorf tube was placed in the freezer (−20 °C) for at least 1 hour, and then it

was centrifuged at 4 °C and 10000 x g for at least 30 minutes. The supernatant was removed, the pellet was redissolved in 300 µL of water, and the procedure was repeated again. The remaining pellet was then dried under a flow of nitrogen, redissolved with 300 µL of water and the solution of DNA conjugate was then desalted. DNA desalting and rebuffering was performed by charging the solution in an AMICON® filter unit from Sigma Aldrich (3 kD) in 300 µL of water, centrifuged at 4 °C and 10000 x g for at least 30 minutes, until the volume decreased to < 10 µL. Another 300 µL of water were added and the process was repeated all over again for at least 3 times. The remaining solution concentration was determined by A_{260} absorption using a Thermo Scientific™ NanoDrop™ One^{C}, concentration of the solution was adjusted to 2.0 mM and stored in the freezer at −20 °C.

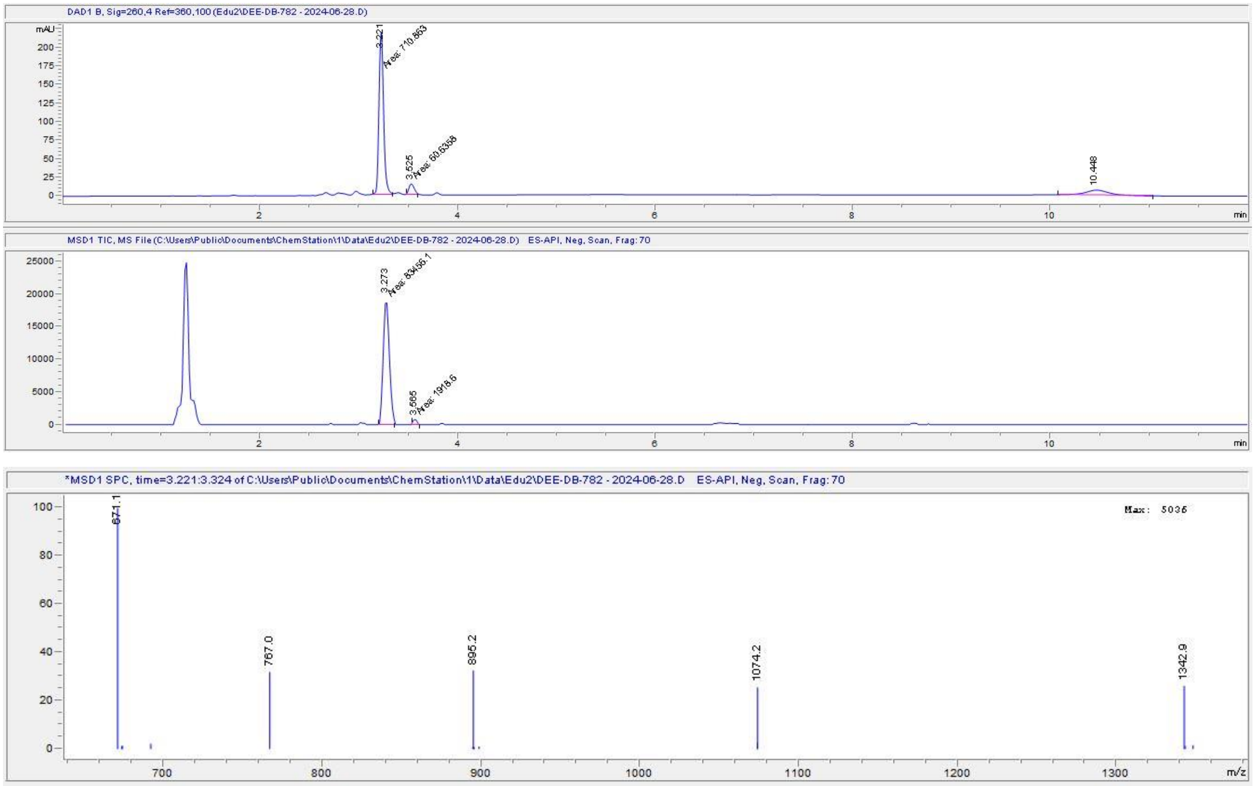

Figure S23. Analytical HPLC trace of **S26** with HPLC Method A. (Up) DAD chromatogram at 260 nm. (Middle) TIC chromatogram. (Below) Ionization of peak at 3.273 min containing reaction product.

DNA-conjugated arene S27

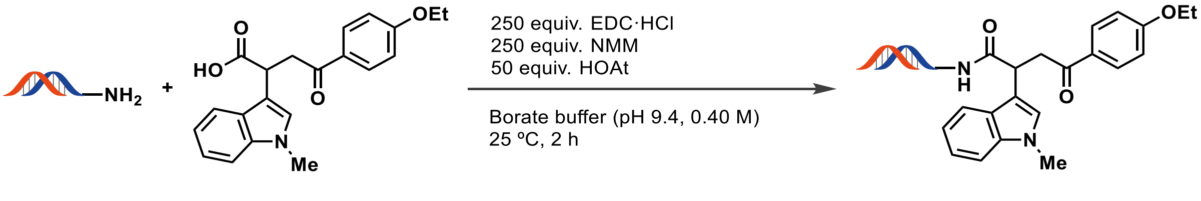

S27, >95%

Exact Mass: 5515.161
Molecular Weight: 5517.909

At 20–25 °C, 20 µL of a 4-(4-Ethoxyphenyl)-2-indol-3-yl-4-oxobutanoic acid stock solution (500 mM, 10 µmol, 500 equiv.) in DMSO was mixed with 10 µL of a HOAt stock solution in DMSO (100 mM, 1.0 µmol, 50 equiv.), and 10 µL of an EDC·HCl stock solution in DMSO (500 mM, 5.0 µmol, 250 equiv.) in a 1.5 mL Eppendorf tube. The mixture was vortexed for 5 seconds. Next, 10 µL of a NMM stock solution (500 mM, 5.0 µmol, 250 equiv.) in DMSO was added. The mixture was vortexed for 5 seconds again, and left

standing at 20–25 °C for 15 min. In another 1.5 mL Eppendorf tube, 60 µL of HP–AOP–NH_2 (0.33 mM, 20 nmol, 1.0 equiv.) in borate buffer (pH 9.4, c = 400 mM) was added, and the premix of acid, HOAt, EDC·HCl and NMM was added over the solution. The mixture was vortexed for 5 seconds, transferred into a Thermocycler at 25 °C, and incubated at 25 °C for 2 hours at 600 rpm. After 2 hours, an aliquot of 1 µL of the reaction mixture was diluted to 40 µL with water for LC–MS analysis.

Next, 10 µL of a 5 M solution of NaCl in water and 360 µL of ethanol at −20 °C were added to precipitate the DNA conjugate. The Eppendorf tube was placed in the freezer (−20 °C) for at least 1 hour, and then it was centrifuged at 4 °C and 10000 x g for at least 30 minutes. The supernatant was removed, the pellet was redissolved in 300 µL of water, and the procedure was repeated again. The remaining pellet was then dried under a flow of nitrogen, redissolved with 300 µL of water and the solution of DNA conjugate was then desalted. DNA desalting and rebuffering was performed by charging the solution in an AMICON® filter unit from Sigma Aldrich (3 kD) in 300 µL of water, centrifuged at 4 °C and 10000 x g for at least 30 minutes, until the volume decreased to < 10 µL. Another 300 µL of water were added and the process was repeated all over again for at least 3 times. The remaining solution concentration was determined by A_{260} absorption using a Thermo Scientific™ NanoDrop™ One^{C}, concentration of the solution was adjusted to 2.0 mM and stored in the freezer at −20 °C.

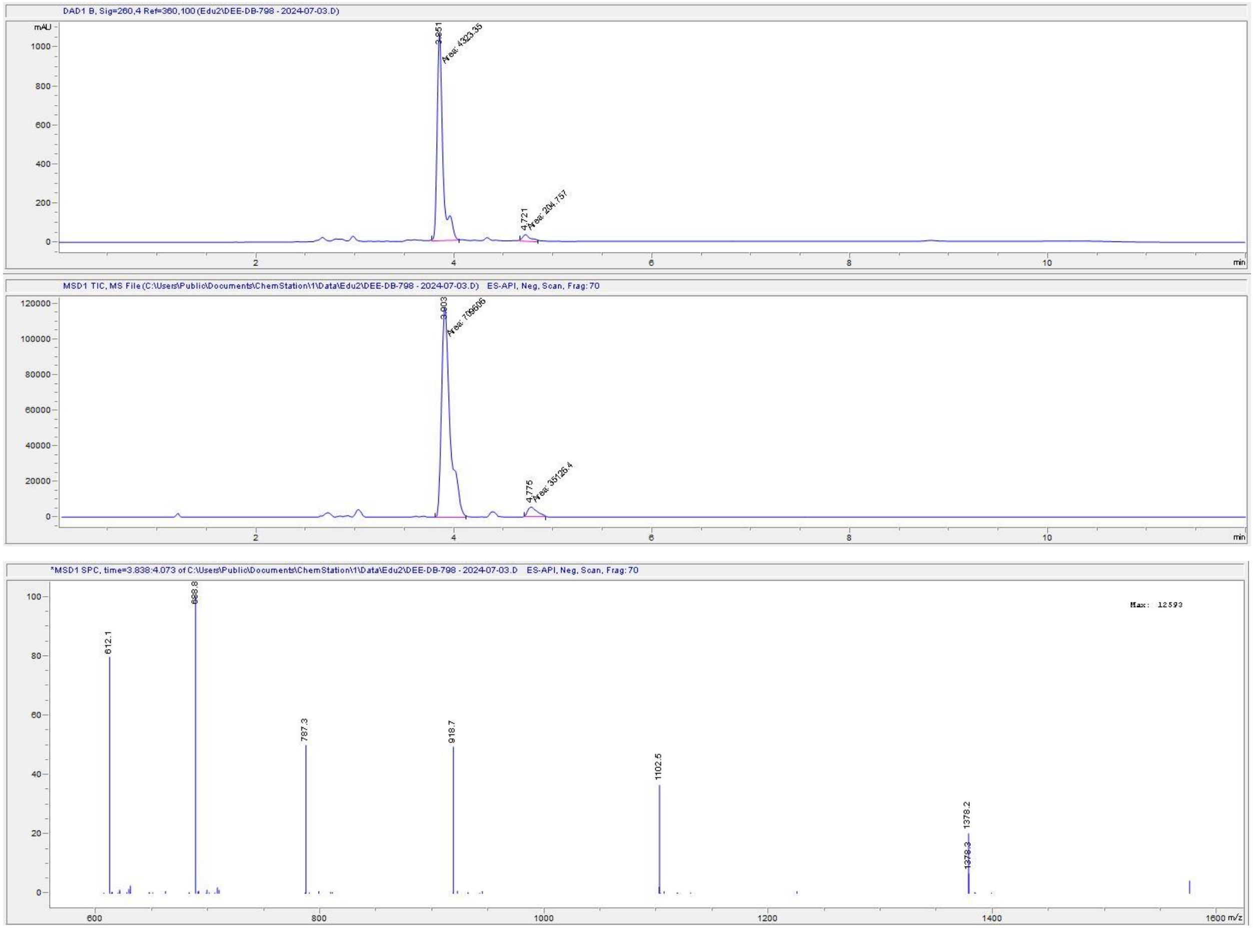

Figure S24. Analytical HPLC trace of **S27** with HPLC Method A. (Up) DAD chromatogram at 260 nm. (Middle) TIC chromatogram. (Below) Ionization of peak at 3.903 min containing reaction product.

DNA-conjugated arene S28

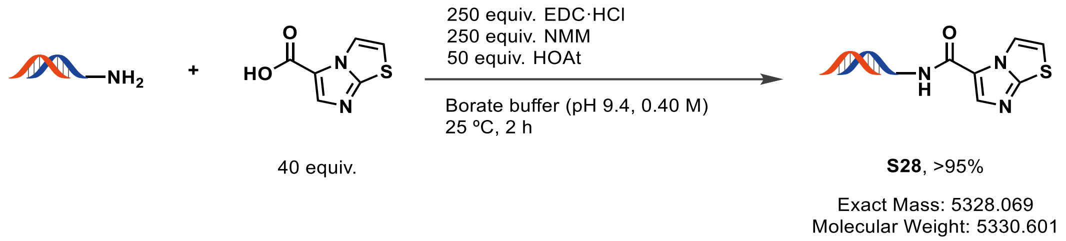

At 20–25 °C, 20 µL of a a imidazo[2,1-*b*]thiazole-5-carboxylic acid stock solution (500 mM, 10 µmol, 500 equiv.) in DMSO was mixed with 10 µL of a HOAt stock solution in DMSO (100 mM, 1.0 µmol, 50 equiv.), and 10 µL of an EDC·HCl stock solution in DMSO (500 mM, 5.0 µmol, 250 equiv.) in a 1.5 mL Eppendorf tube. The mixture was vortexed for 5 seconds. Next, 10 µL of a NMM stock solution (500 mM, 5.0 µmol, 250 equiv.) in DMSO was added. The mixture was vortexed for 5 seconds again, and left standing at 20–25 °C for 15 min. In another 1.5 mL Eppendorf tube, 60 µL of HP–AOP–NH_2 (0.33 mM, 20 nmol, 1.0 equiv.) in borate buffer (pH 9.4, c = 400 mM) was added, and the premix of acid, HOAt, EDC·HCl and NMM was added over the solution. The mixture was vortexed for 5 seconds, transferred into a Thermocycler at 25 °C, and incubated at 25 °C for 2 hours at 600 rpm. After 2 hours, an aliquot of 1 µL of the reaction mixture was diluted to 40 µL with water for LC–MS analysis.

Next, 10 µL of a 5 M solution of NaCl in water and 360 µL of ethanol at −20 °C were added to precipitate the DNA conjugate. The Eppendorf tube was placed in the freezer (−20 °C) for at least 1 hour, and then it was centrifuged at 4 °C and 10000 x g for at least 30 minutes. The supernatant was removed, the pellet was redissolved in 300 µL of water, and the procedure was repeated again. The remaining pellet was then dried under a flow of nitrogen, redissolved with 300 µL of water and the solution of DNA conjugate was then desalted. DNA desalting and rebuffering was performed by charging the solution in an AMICON® filter unit from Sigma Aldrich (3 kD) in 300 µL of water, centrifuged at 4 °C and 10000 x g for at least 30 minutes, until the volume decreased to < 10 µL. Another 300 µL of water were added and the process was repeated all over again for at least 3 times. The remaining solution concentration was determined by A_{260} absorption using a Thermo Scientific™ NanoDrop™ One^C, concentration of the solution was adjusted to 2.0 mM and stored in the freezer at −20 °C.

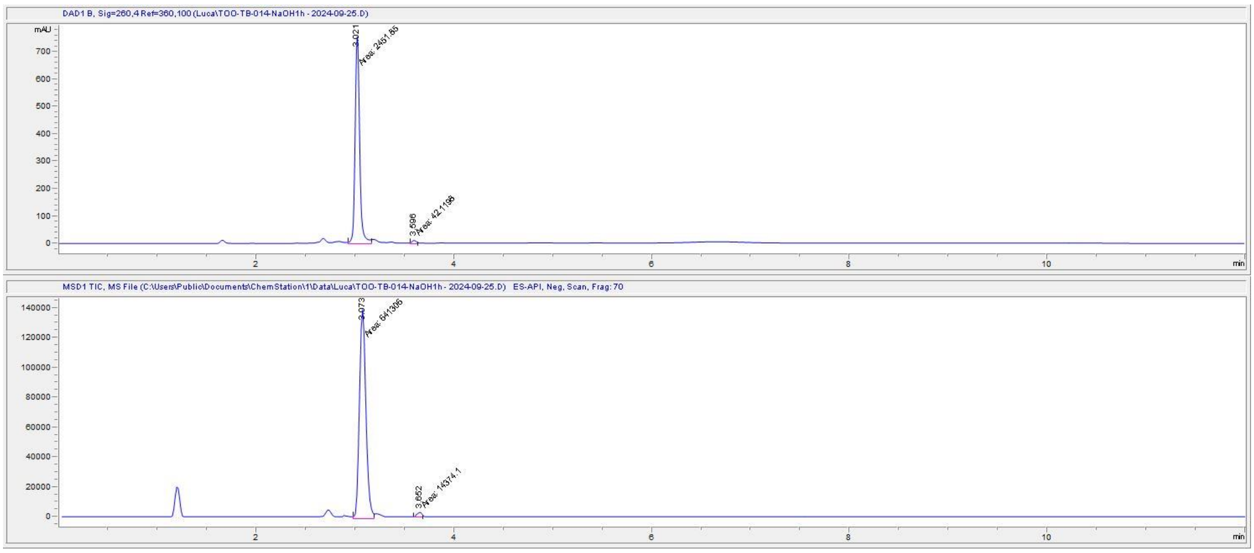

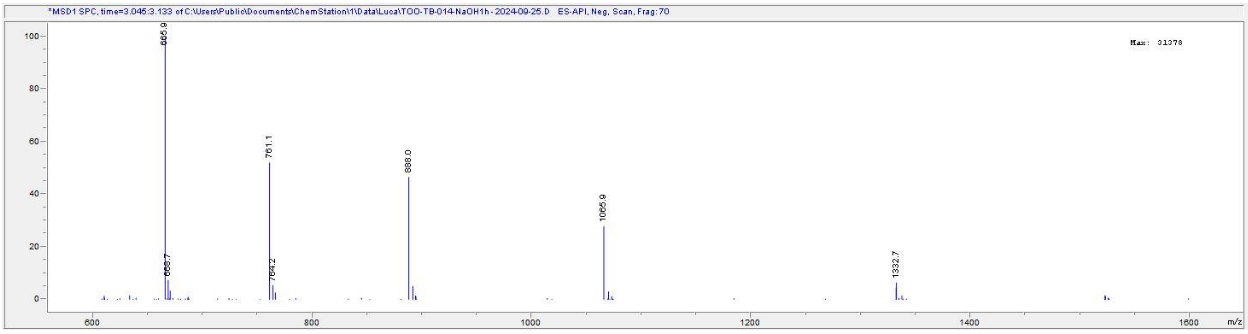

Figure S25. Analytical HPLC trace of **S28** with HPLC Method A. (Up) DAD chromatogram at 260 nm. (Middle) TIC chromatogram. (Below) Ionization of peak at 3.073 min containing reaction product.

DNA-conjugated arene S29

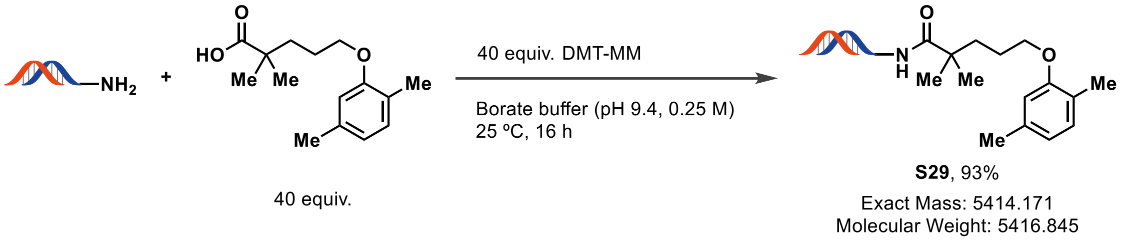

S29, 93%

At 20–25 °C, 20 µL of HP–AOP–NH_2 (1.0 mM, 20 nmol, 1.0 equiv.) in borate buffer (pH 9.4, c = 250 mM) was added to a 1.5 mL Eppendorf tube. Next, 2.0 µL of a gemfibrozil stock solution (400 mM, 0.80 µmol, 40 equiv.) in DMA was added. The mixture was vortexed for 5 seconds. Then, 2.0 µL of a DMT-MM stock solution (400 mM, 0.80 µmol, 40 equiv.) in water was added. The mixture was vortexed for 5 seconds again, transferred into a Thermocycler at 25 °C, and incubated at 25 °C for 16 hours at 600 rpm. After 16 hours, an aliquot of 1 µL of the reaction mixture was diluted to 40 µL with water for LC–MS analysis.

Next, 2.4 µL of a 5 M solution of NaCl in water and 80 µL of ethanol at −20 °C were added to precipitate the DNA conjugate. The Eppendorf tube was placed in the freezer (−20 °C) for at least 1 hour, and then it was centrifuged at 4 °C and 10000 x g for at least 30 minutes. The supernatant was removed, the pellet was redissolved in 24 µL of water, and the procedure was repeated again. The remaining pellet was then dried under a flow of nitrogen, redissolved with 10 µL of water and stored in the freezer at −20 °C.

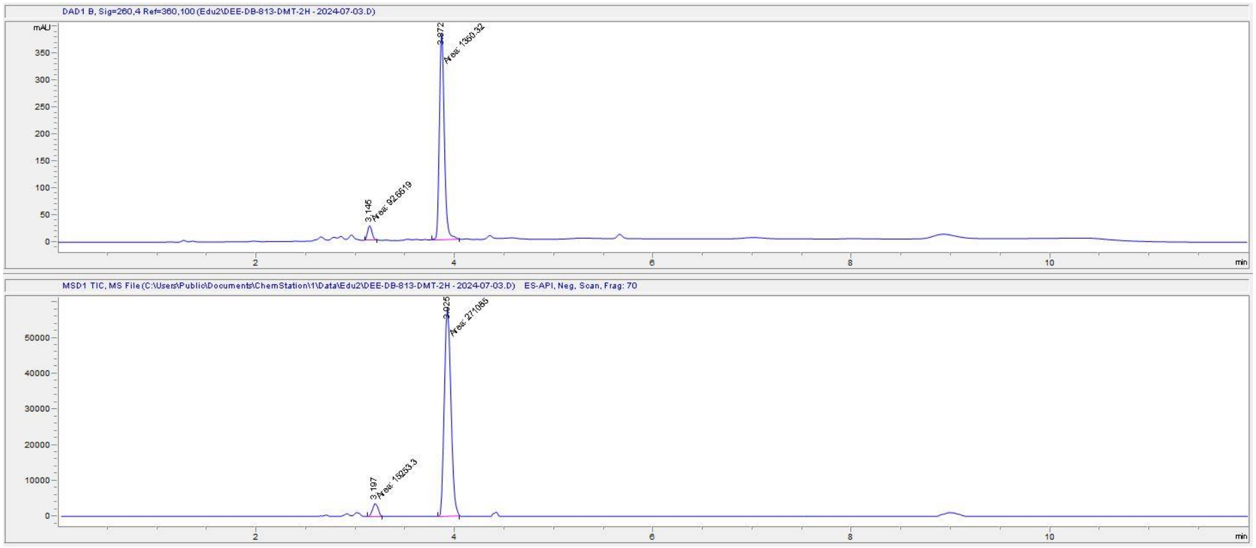

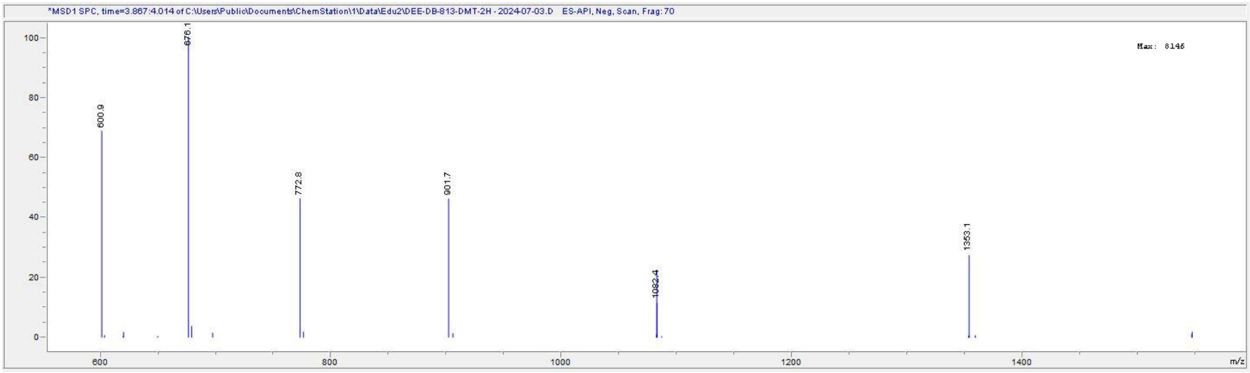

Figure S26. Analytical HPLC trace of **S29** with HPLC Method A. (Up) DAD chromatogram at 260 nm. (Middle) TIC chromatogram. (Below) Ionization of peak at 3.925 min containing reaction product.

DNA-conjugated arene S30

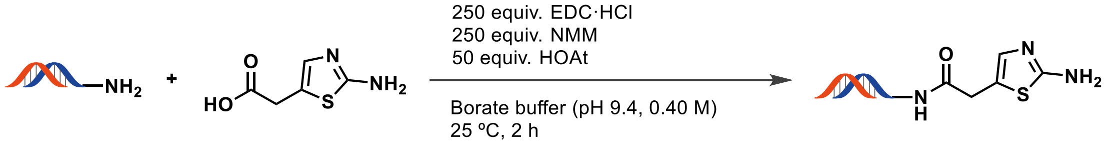

500 equiv.

S30, >95%

Exact Mass: 5322.029
Molecular Weight: 5324.468

At 20–25 °C, 20 µL of a 2-(2-aminothiazol-5-yl)acetic acid stock solution (500 mM, 10 µmol, 500 equiv.) in DMSO was mixed with 10 µL of a HOAt stock solution in DMSO (100 mM, 1.0 µmol, 50 equiv.), and 10 µL of an EDC·HCl stock solution in DMSO (500 mM, 5.0 µmol, 250 equiv.) in a 1.5 mL Eppendorf tube. The mixture was vortexed for 5 seconds. Next, 10 µL of a NMM stock solution (500 mM, 5.0 µmol, 250 equiv.) in DMSO was added. The mixture was vortexed for 5 seconds again, and left standing at 20–25 °C for 15 min. In another 1.5 mL Eppendorf tube, 60 µL of HP–AOP–NH_2 (0.33 mM, 20 nmol, 1.0 equiv.) in borate buffer (pH 9.4, c = 400 mM) was added, and the premix of acid, HOAt, EDC·HCl and NMM was added over the solution. The mixture was vortexed for 5 seconds, transferred into a Thermocycler at 25 °C, and incubated at 25 °C for 2 hours at 600 rpm. After 2 hours, an aliquot of 1 µL of the reaction mixture was diluted to 40 µL with water for LC–MS analysis.

Next, 10 µL of a 5 M solution of NaCl in water and 360 µL of ethanol at −20 °C were added to precipitate the DNA conjugate. The Eppendorf tube was placed in the freezer (−20 °C) for at least 1 hour, and then it was centrifuged at 4 °C and 10000 x g for at least 30 minutes. The supernatant was removed, the pellet was redissolved in 300 µL of water, and the procedure was repeated again. The remaining pellet was then dried under a flow of nitrogen, redissolved with 300 µL of water and the solution of DNA conjugate was then desalted. DNA desalting and rebuffering was performed by charging the solution in an AMICON® filter unit from Sigma Aldrich (3 kD) in 300 µL of water, centrifuged at 4 °C and 10000 x g for at least 30 minutes, until the volume decreased to < 10 µL. Another 300 µL of water were added and the process was repeated all over again for at least 3 times. The remaining solution concentration was determined by A_{260} absorption using a Thermo Scientific™ NanoDrop™ One^{C}, concentration of the solution was adjusted to 2.0 mM and stored in the freezer at −20 °C.

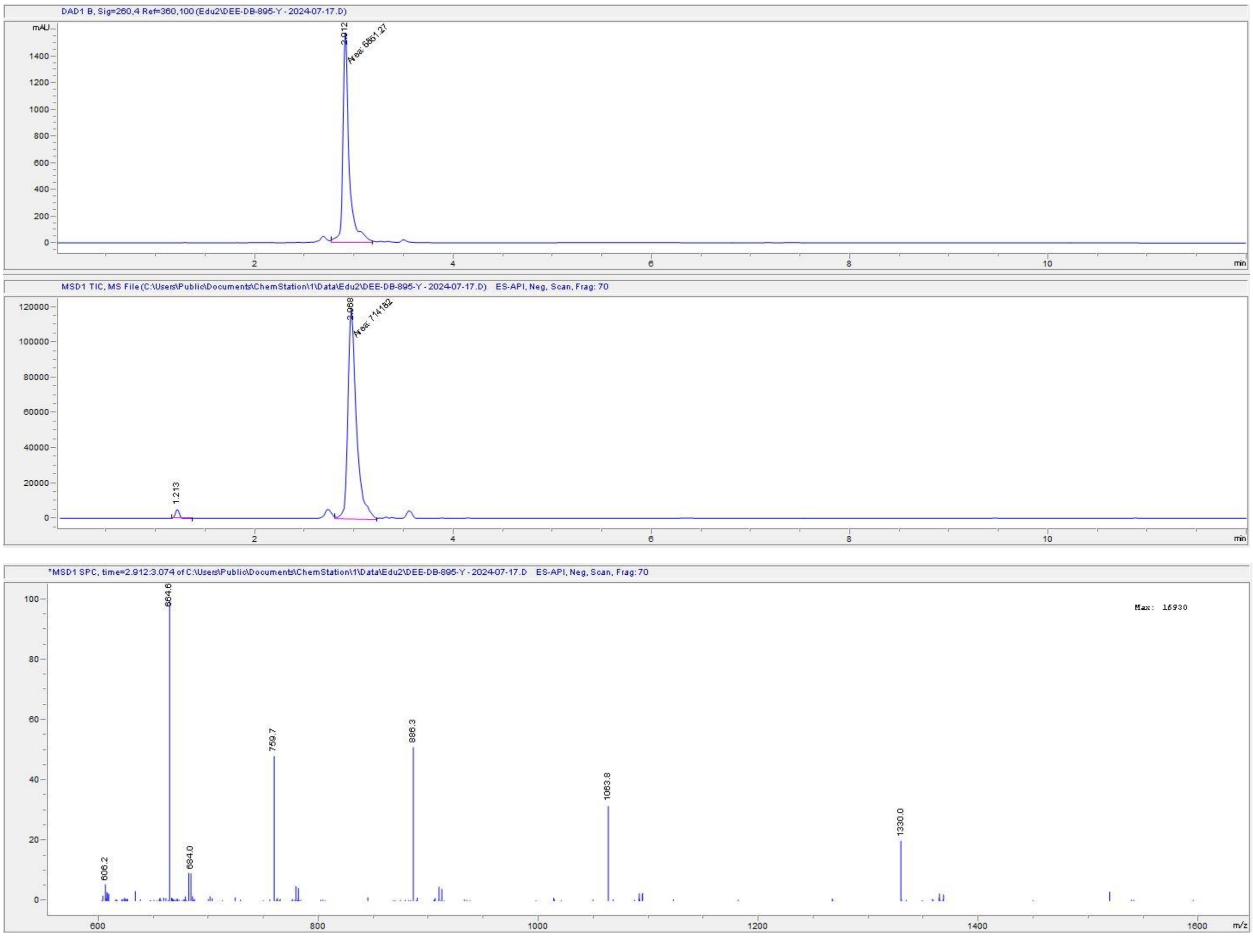

Figure S27. Analytical HPLC trace of **S30** with HPLC Method A. (Up) DAD chromatogram at 260 nm. (Middle) TIC chromatogram. (Below) Ionization of peak at 2.968 min containing reaction product.

DNA-conjugated arene S31

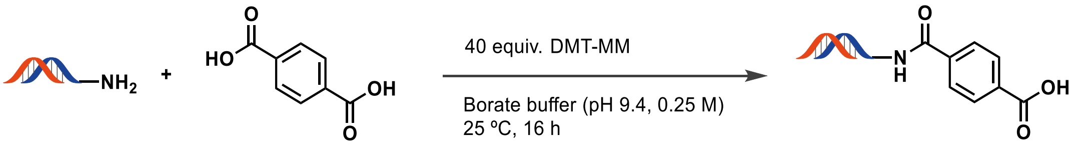

At 20–25 °C, 20 µL of HP–AOP–NH_2 (1.0 mM, 20 nmol, 1.0 equiv.) in borate buffer (pH 9.4, c = 250 mM) was added to a 1.5 mL Eppendorf tube. Next, 2.0 µL of a terephthalic acid stock solution (400 mM, 0.80 µmol, 40 equiv.) in DMA was added. The mixture was vortexed for 5 seconds. Then, 2.0 µL of a DMT-MM stock solution (400 mM, 0.80 µmol, 40 equiv.) in water was added. The mixture was vortexed for 5 seconds again, transferred into a Thermocycler at 25 °C, and incubated at 25 °C for 16 hours at 600 rpm. After 16 hours, an aliquot of 1 µL of the reaction mixture was diluted to 40 µL with water for LC–MS analysis.

Next, 2.4 µL of a 5 M solution of NaCl in water and 80 µL of ethanol at −20 °C were added to precipitate the DNA conjugate. The Eppendorf tube was placed in the freezer (−20 °C) for at least 1 hour, and then it was centrifuged at 4 °C and 10000 x g for at least 30 minutes. The supernatant was removed, the pellet was redissolved in 24 µL of water, and the procedure was repeated again. The remaining pellet was then

dried under a flow of nitrogen, redissolved with 10 µL of water and stored in the freezer at −20 °C.

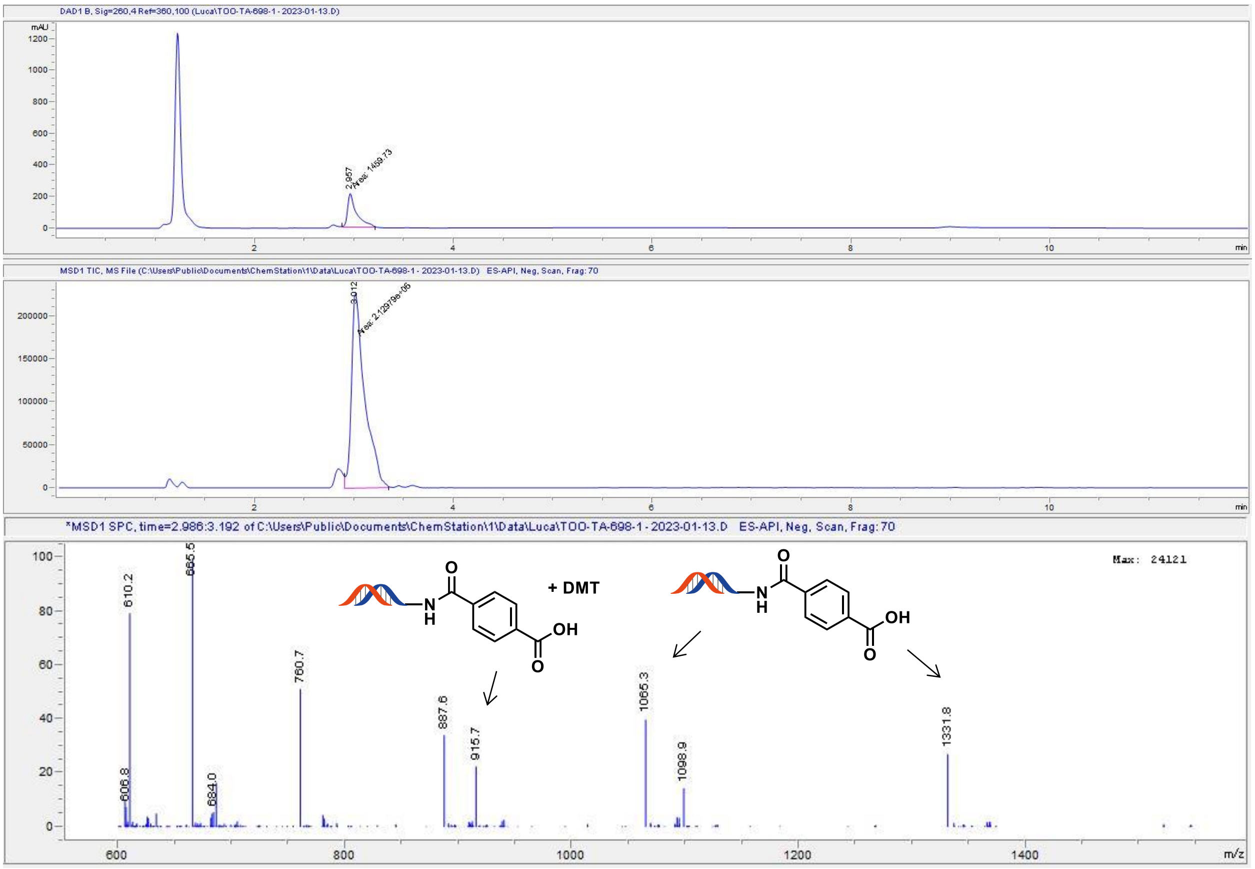

Figure S28. Analytical HPLC trace of **S28** with HPLC Method A. (Up) DAD chromatogram at 260 nm. (Middle) TIC chromatogram. (Below) Ionization of peak at 3.012 min containing reaction product.

DNA-conjugated arene S32

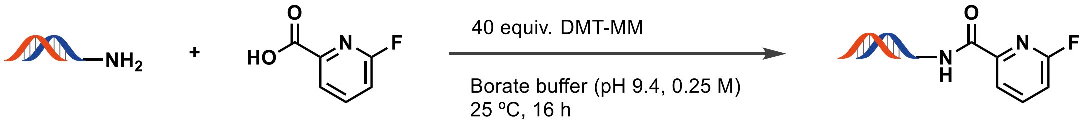

40 equiv.

S32, >95%

Exact Mass: 5305.037
Molecular Weight: 5307.608

At 20–25 °C, 20 µL of HP–AOP–NH_2 (1.0 mM, 20 nmol, 1.0 equiv.) in borate buffer (pH 9.4, c = 250 mM) was added to a 1.5 mL Eppendorf tube. Next, 2.0 µL of a 6-fluoropicolinic acid stock solution (400 mM, 0.80 µmol, 40 equiv.) in DMA was added. The mixture was vortexed for 5 seconds. Then, 2.0 µL of a DMT-MM stock solution (400 mM, 0.80 µmol, 40 equiv.) in water was added. The mixture was vortexed for 5 seconds again, transferred into a Thermocycler at 25 °C, and incubated at 25 °C for 16 hours at 600 rpm. After 16 hours, an aliquot of 1 µL of the reaction mixture was diluted to 40 µL with water for LC–MS analysis.

Next, 2.4 µL of a 5 M solution of NaCl in water and 80 µL of ethanol at −20 °C were added to precipitate the DNA conjugate. The Eppendorf tube was placed in the freezer (−20 °C) for at least 1 hour, and then it was centrifuged at 4 °C and 10000 x g for at least 30 minutes. The supernatant was removed, the pellet was redissolved in 24 µL of water, and the procedure was repeated again. The remaining pellet was then dried under a flow of nitrogen, redissolved with 10 µL of water and stored in the freezer at −20 °C.

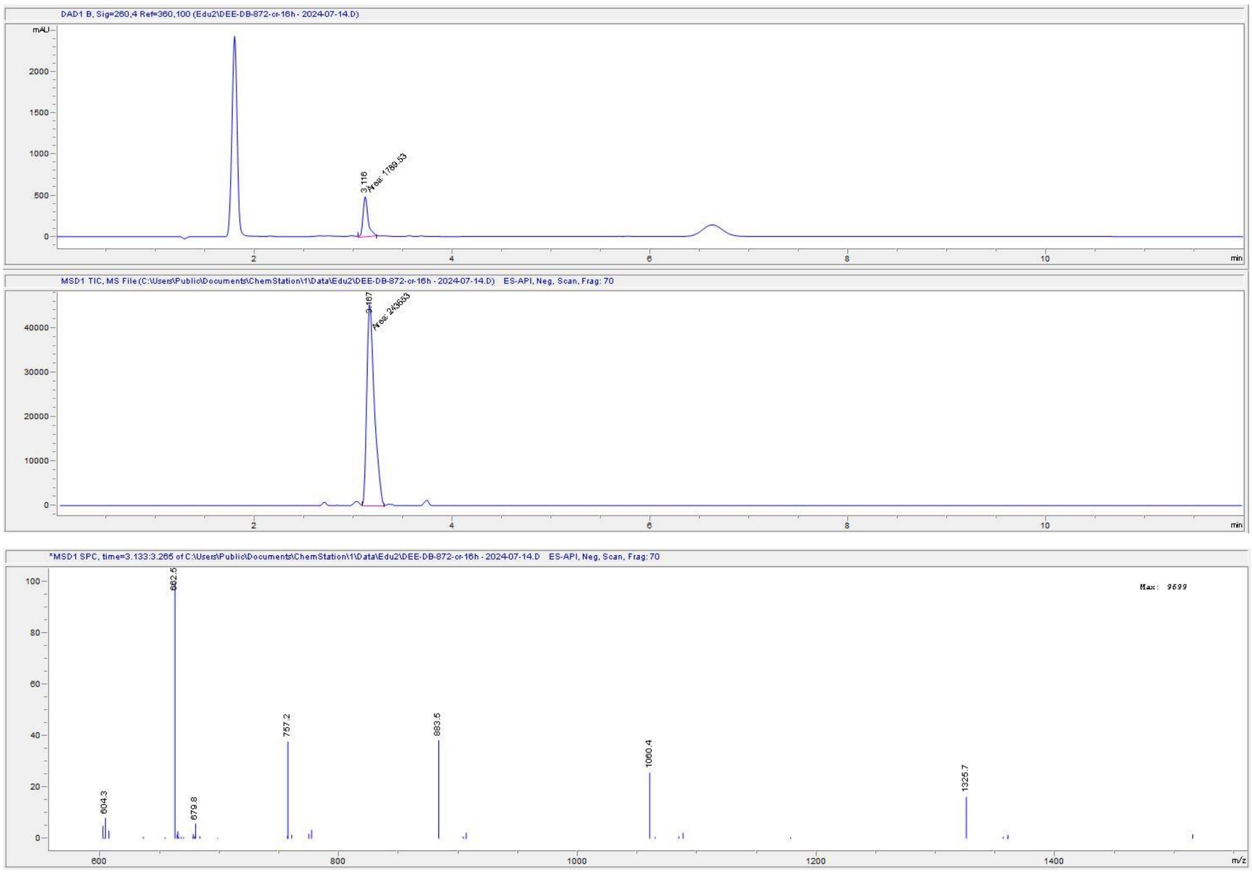

Figure S29. Analytical HPLC trace of **S32** with HPLC Method A. (Up) DAD chromatogram at 260 nm. (Middle) TIC chromatogram. (Below) Ionization of peak at 3.167 min containing reaction product.

DNA-conjugated arene S33

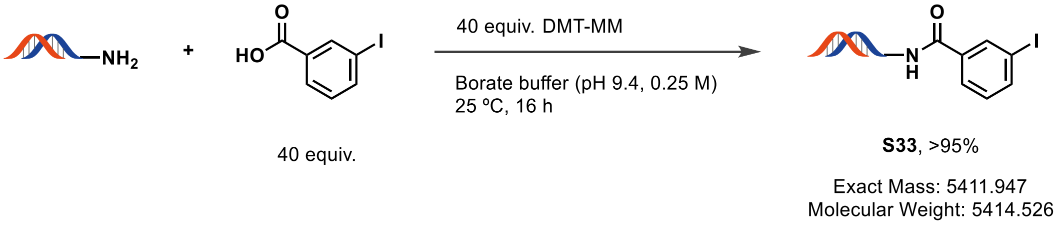

At 20–25 °C, 20 µL of HP–AOP–NH_2 (1.0 mM, 20 nmol, 1.0 equiv.) in borate buffer (pH 9.4, c = 250 mM) was added to a 1.5 mL Eppendorf tube. Next, 2.0 µL of a 3-iodobenzoic acid stock solution (400 mM, 0.80 µmol, 40 equiv.) in DMA was added. The mixture was vortexed for 5 seconds. Then, 2.0 µL of a DMT-MM stock solution (400 mM, 0.80 µmol, 40 equiv.) in water was added. The mixture was vortexed for 5 seconds again, transferred into a Thermocycler at 25 °C, and incubated at 25 °C for 16 hours at 600 rpm. After 16 hours, an aliquot of 1 µL of the reaction mixture was diluted to 40 µL with water for LC–MS analysis.

Next, 2.4 µL of a 5 M solution of NaCl in water and 80 µL of ethanol at −20 °C were added to precipitate the DNA conjugate. The Eppendorf tube was placed in the freezer (−20 °C) for at least 1 hour, and then it was centrifuged at 4 °C and 10000 x g for at least 30 minutes. The supernatant was removed, the pellet was redissolved in 24 µL of water, and the procedure was repeated again. The remaining pellet was then dried under a flow of nitrogen, redissolved with 10 µL of water and stored in the freezer at −20 °C.

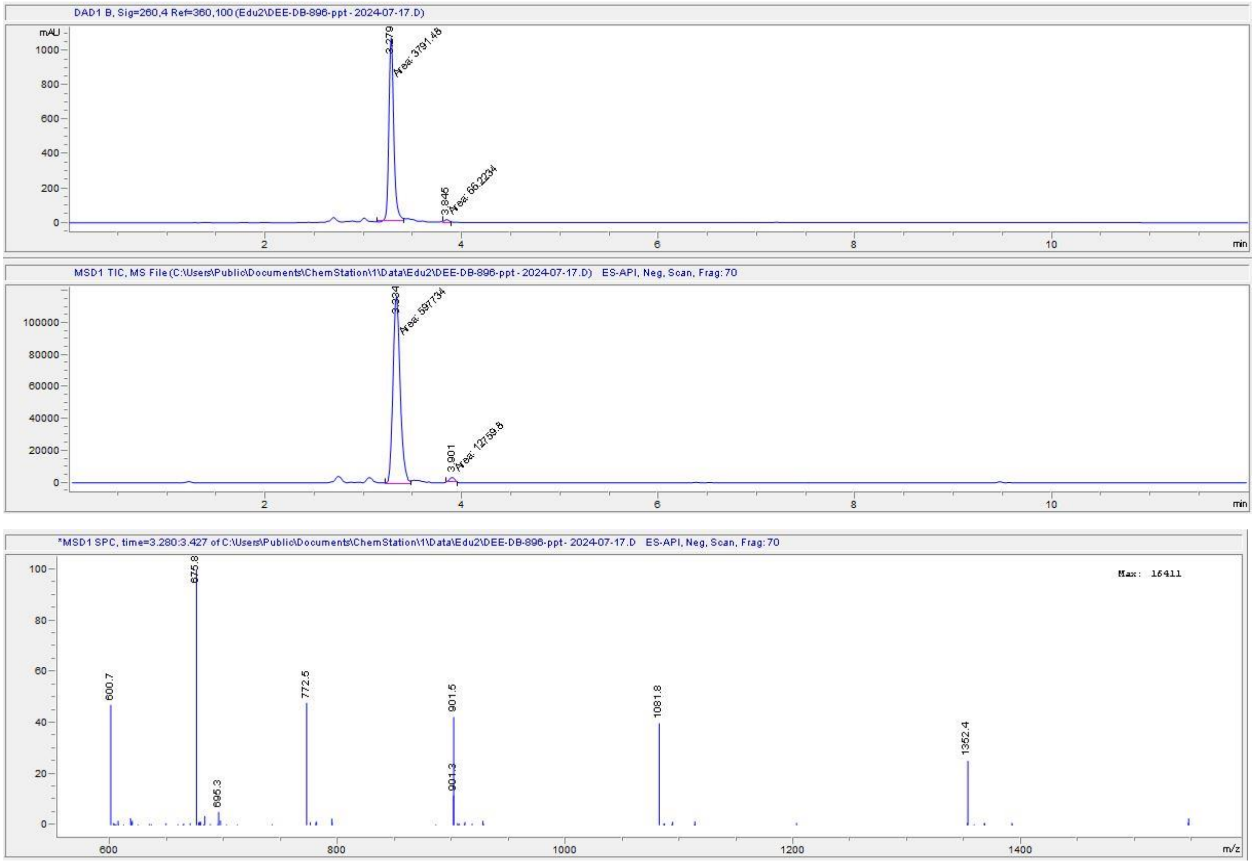

Figure S30. Analytical HPLC trace of **S33** with HPLC Method A. (Up) DAD chromatogram at 260 nm. (Middle) TIC chromatogram. (Below) Ionization of peak at 3.334 min containing reaction product.

DNA-conjugated arene S34

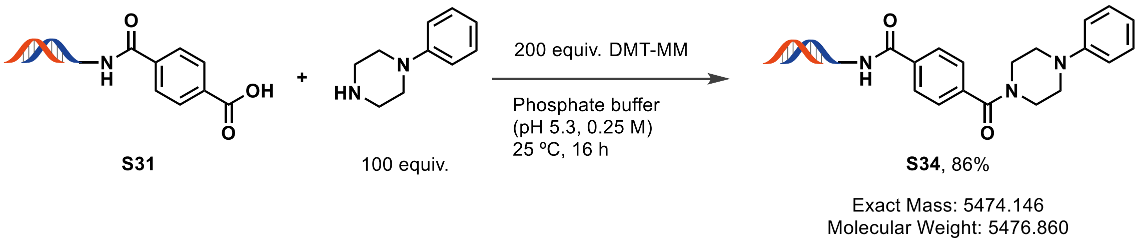

S31 100 equiv. **S34**, 86%

Exact Mass: 5474.146
Molecular Weight: 5476.860

At 20–25 °C, 20 µL of HP–AOP–NHCOPhCO$_2$H **S31** (1.0 mM, 20 nmol, 1.0 equiv.) in phosphate buffer (pH 5.3, c = 250 mM) was added to a 1.5 mL Eppendorf tube. Next, 5.0 µL of a 1-phenylpiperazine stock solution (400 mM, 2.0 µmol, 100 equiv.) in DMA was added. The mixture was vortexed for 5 seconds. Then, 5.0 µL of a DMT-MM stock solution (400 mM, 2.0 µmol, 100 equiv.) in water was added. The mixture was vortexed for 5 seconds again, transferred into a Thermocycler at 25 °C, and incubated at 25 °C for 2 hours at 600 rpm. After 2h, a second addition of 5.0 µL of a DMT-MM stock solution (400 mM, 2.0 µmol, 100 equiv.) in water was done. The mixture was vortexed for 5 seconds, transferred into a Thermocycler at 25 °C, and incubated at 25 °C for 14 more hours at 600 rpm. After 16 hours, an aliquot of 1 µL of the reaction mixture was diluted to 40 µL with water for LC–MS analysis.

Next, 3.5 µL of a 5 M solution of NaCl in water and 100 µL of ethanol at −20 °C were added to precipitate the DNA conjugate. The Eppendorf tube was placed in the freezer (−20 °C) for at least 1 hour, and then it was centrifuged at 4 °C and 10000 x g for at least 30 minutes. The supernatant was removed, the pellet was redissolved in 35 µL of water, and the procedure was repeated again. The remaining pellet was then

dried under a flow of nitrogen, redissolved with 10 µL of water and stored in the freezer at −20 °C.

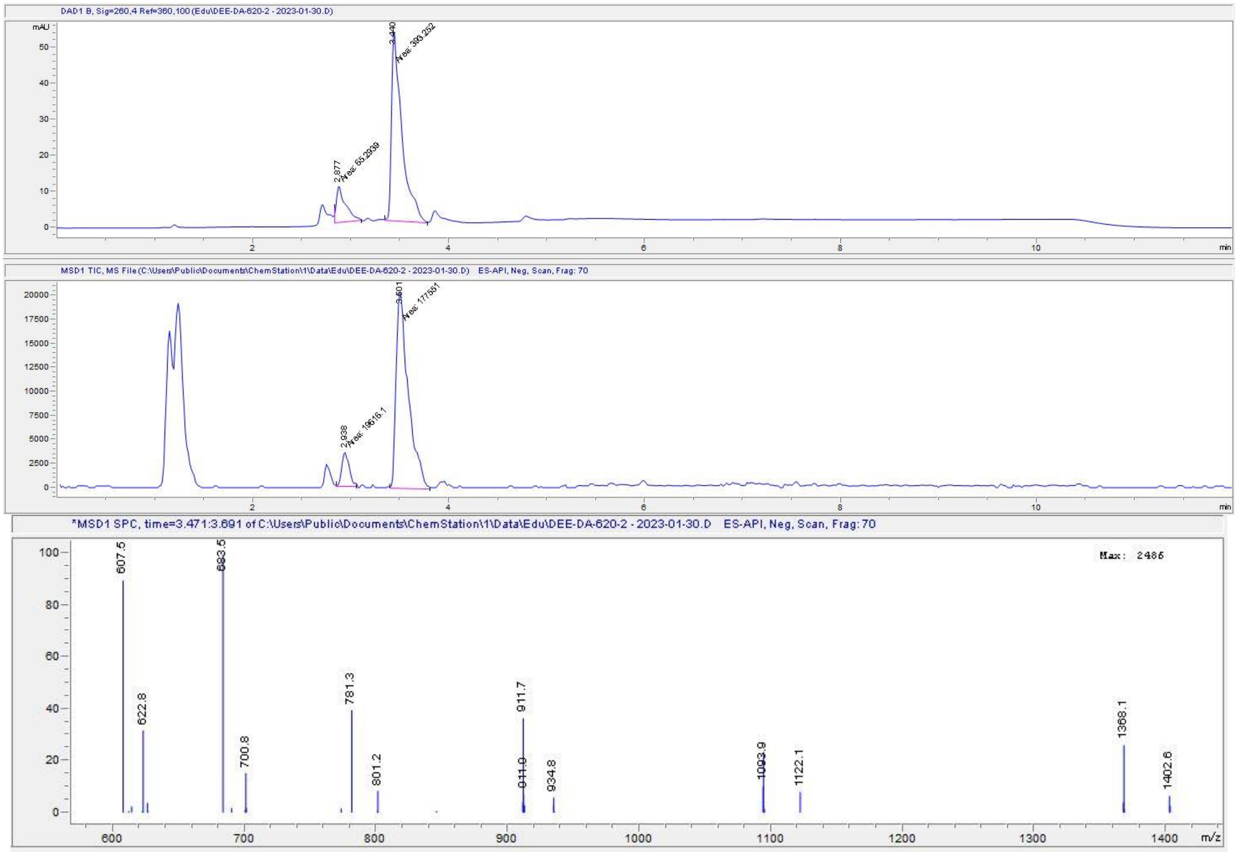

Figure S31. Analytical HPLC trace of **S34** with HPLC Method A. (Up) DAD chromatogram at 260 nm. (Middle) TIC chromatogram. (Below) Ionization of peak at 3.501 min containing reaction product.

DNA-conjugated arene S35

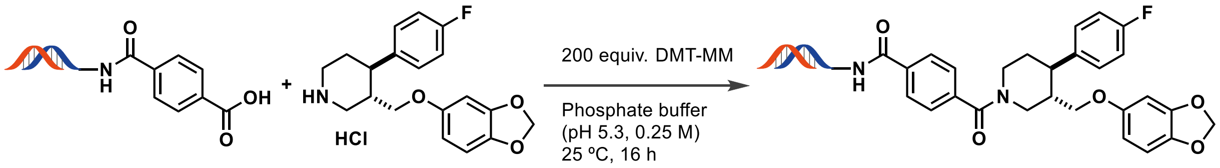

S31 **S35**, 32%

At 20–25 °C, 20 µL of HP–AOP–NHCOPhCO$_2$H **S31** (1.0 mM, 20 nmol, 1.0 equiv.) in phosphate buffer (pH 5.3, c = 250 mM) was added to a 1.5 mL Eppendorf tube. Next, 5.0 µL of a (3*S*,4*R*)-3-[(1,3-Benzodioxol-5-yloxy)methyl]-4-(4-fluorophenyl)piperidine hydrochloride stock solution (400 mM, 2.0 µmol, 100 equiv.) in DMA was added. The mixture was vortexed for 5 seconds. Then, 5.0 µL of a DMT-MM stock solution (400 mM, 2.0 µmol, 100 equiv.) in water was added. The mixture was vortexed for 5 seconds again, transferred into a Thermocycler at 25 °C, and incubated at 25 °C for 2 hours at 600 rpm. After 2h, a second addition of 5.0 µL of a DMT-MM stock solution (400 mM, 2.0 µmol, 100 equiv.) in water was done. The mixture was vortexed for 5 seconds, transferred into a Thermocycler at 25 °C, and incubated at 25 °C for 14 more hours at 600 rpm. After 16 hours, an aliquot of 1 µL of the reaction mixture was diluted to 40 µL with water for LC–MS analysis.

Next, 3.5 µL of a 5 M solution of NaCl in water and 100 µL of ethanol at −20 °C were added to precipitate the DNA conjugate. The Eppendorf tube was placed in the freezer (−20 °C) for at least 1 hour, and then it

was centrifuged at 4 °C and 10000 x g for at least 30 minutes. The supernatant was removed, the pellet was redissolved in 35 µL of water, and the procedure was repeated again. The remaining pellet was then dried under a flow of nitrogen, redissolved with 10 µL of water and stored in the freezer at −20 °C.

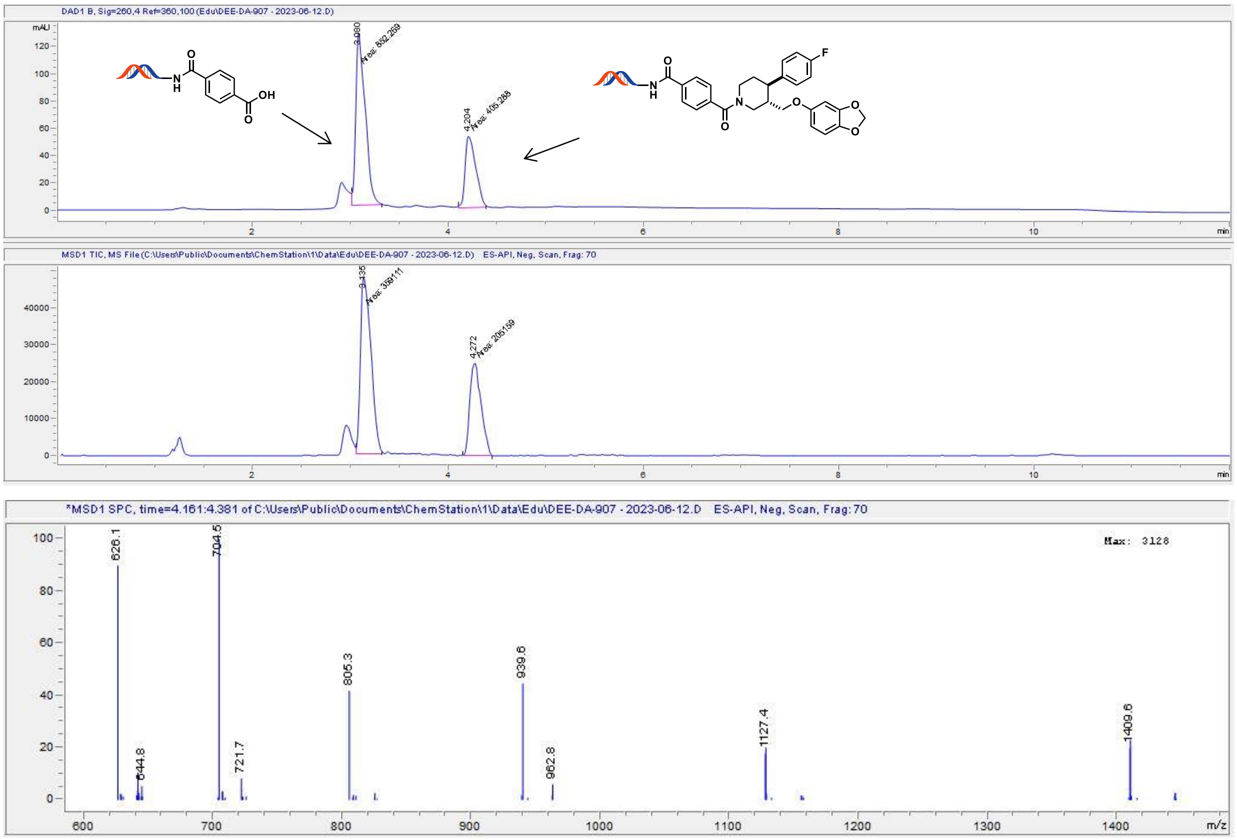

Figure S32. Analytical HPLC trace of **S35** with HPLC Method A. (Up) DAD chromatogram at 260 nm. (Middle) TIC chromatogram. (Below) Ionization of peak at 4.272 min containing reaction product.

DNA-conjugated arene S36

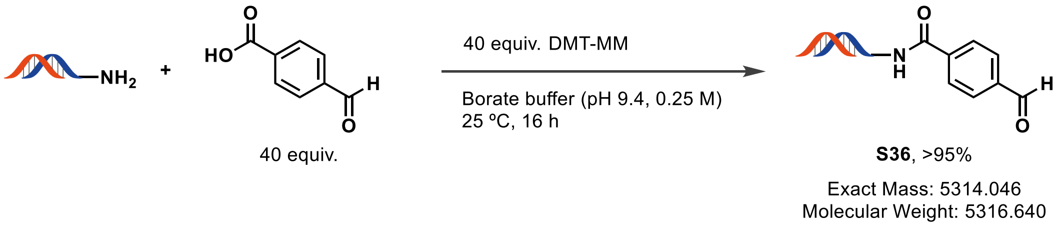

At 20–25 °C, 20 µL of HP–AOP–NH2 (1.0 mM, 20 nmol, 1.0 equiv.) in borate buffer (pH 9.4, c = 250 mM) was added to a 1.5 mL Eppendorf tube. Next, 2.0 µL of a 4-formylbenzoic acid stock solution (400 mM, 0.80 µmol, 40 equiv.) in DMA was added. The mixture was vortexed for 5 seconds. Then, 2.0 µL of a DMT-MM stock solution (400 mM, 0.80 µmol, 40 equiv.) in water was added. The mixture was vortexed for 5 seconds again, transferred into a Thermocycler at 25 °C, and incubated at 25 °C for 16 hours at 600 rpm. After 16 hours, an aliquot of 1 µL of the reaction mixture was diluted to 40 µL with water for LC–MS analysis.

Next, 2.4 µL of a 5 M solution of NaCl in water and 80 µL of ethanol at −20 °C were added to precipitate the DNA conjugate. The Eppendorf tube was placed in the freezer (−20 °C) for at least 1 hour, and then it was centrifuged at 4 °C and 10000 x g for at least 30 minutes. The supernatant was removed, the pellet was redissolved in 24 µL of water, and the procedure was repeated again. The remaining pellet was then

dried under a flow of nitrogen, redissolved with 10 µL of water and stored in the freezer at −20 °C.

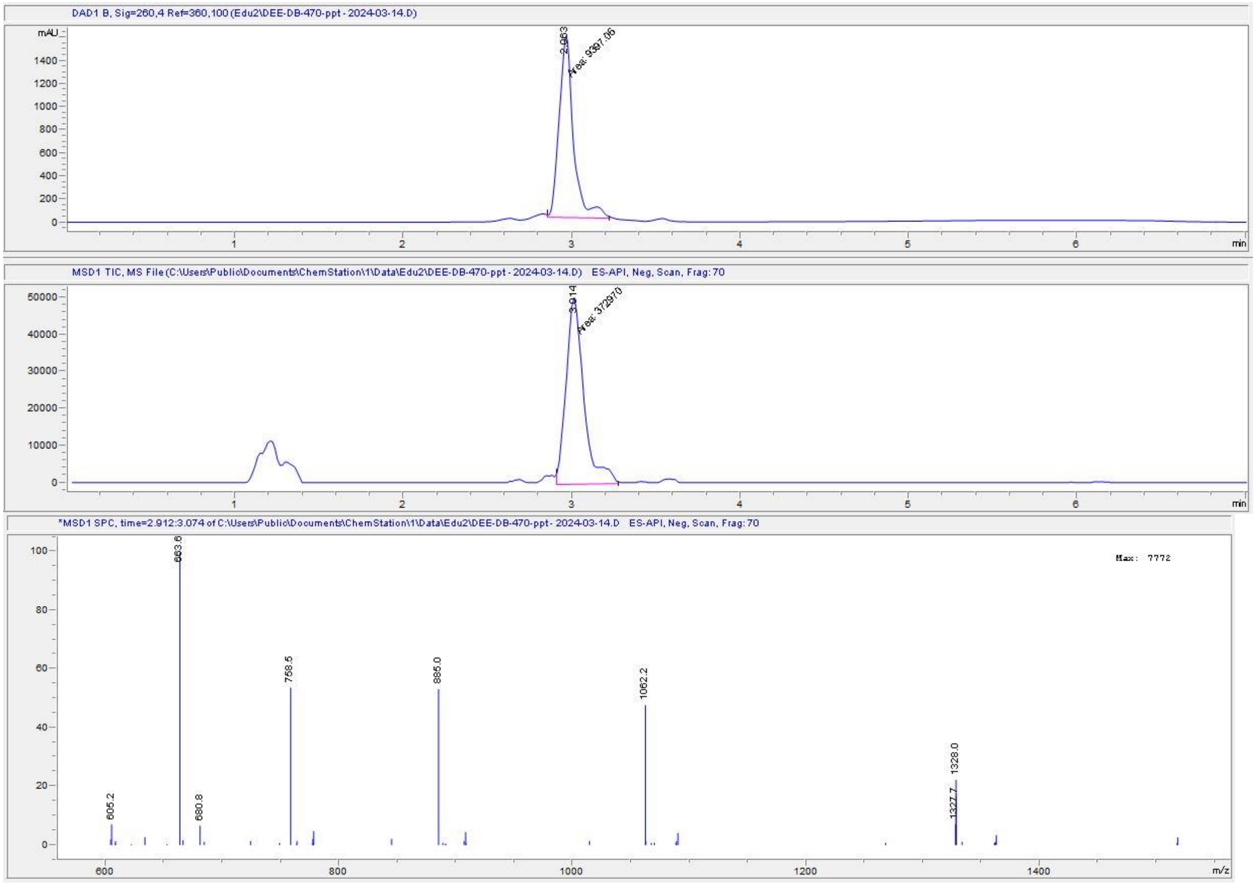

Figure S33. Analytical HPLC trace of **S36** with HPLC Method B. (Up) DAD chromatogram at 260 nm. (Middle) TIC chromatogram. (Below) Ionization of peak at 3.014 min containing reaction product.

DNA-conjugated arene S37

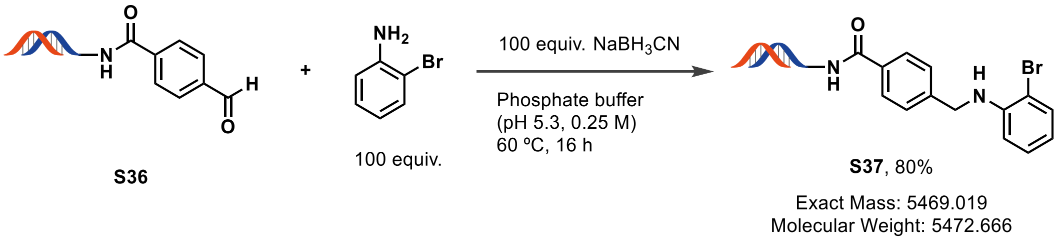

S36

100 equiv.

S37, 80%

Exact Mass: 5469.019
Molecular Weight: 5472.666

At 20–25 °C, 20 µL of HP–AOP–NHCOPhCOH **S36** (1.0 mM, 20 nmol, 1.0 equiv.) in phosphate buffer (pH 5.3, c = 250 mM) was added to a 1.5 mL Eppendorf tube. Next, 10 µL of a 2-bromoaniline stock solution (200 mM, 2.0 µmol, 100 equiv.) in DMA was added. The mixture was vortexed for 5 seconds. Then, 10 µL of a sodium cyanoborohydride stock solution (200 mM, 2.0 µmol, 100 equiv.) in MeCN was added. The mixture was vortexed for 5 seconds again, transferred into a pre-heated Thermocycler at 60 °C, and incubated at 60 °C for 16 hours at 1000 rpm. After 16 hours, an aliquot of 1 µL of the reaction mixture was diluted to 40 µL with water for LC–MS analysis.

Next, 4 µL of a 5 M solution of NaCl in water and 130 µL of ethanol at −20 °C were added to precipitate the DNA conjugate. The Eppendorf tube was placed in the freezer (−20 °C) for at least 1 hour, and then it was centrifuged at 4 °C and 10000 x g for at least 30 minutes. The supernatant was removed, the pellet was redissolved in 35 µL of water, and the procedure was repeated again. The remaining pellet was then dried under a flow of nitrogen, redissolved with 10 µL of water and stored in the freezer at −20 °C.

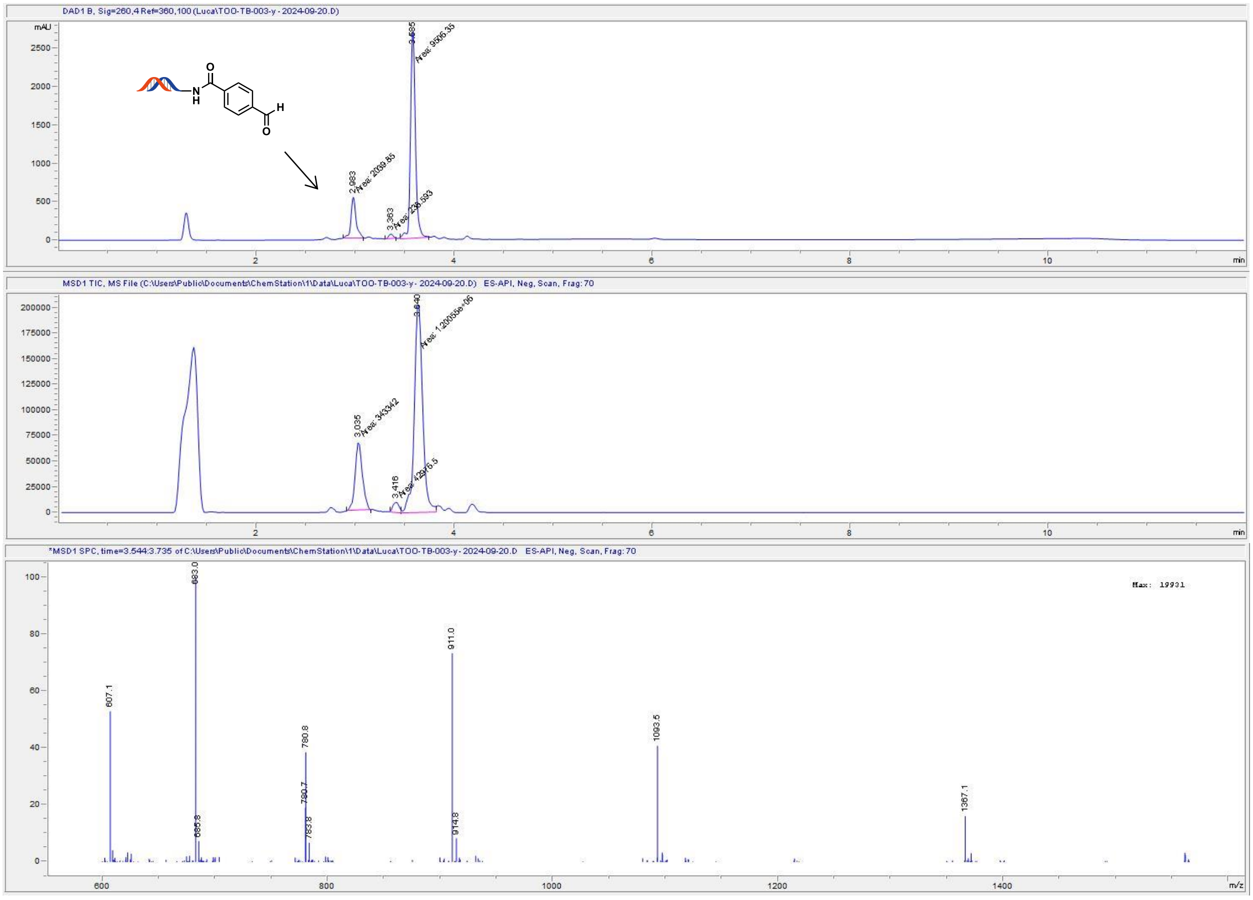

Figure S34. Analytical HPLC trace of **S37** with HPLC Method A. (Up) DAD chromatogram at 260 nm. (Middle) TIC chromatogram. (Below) Ionization of peak at 3.640 min containing reaction product.

DNA-conjugated arene S38

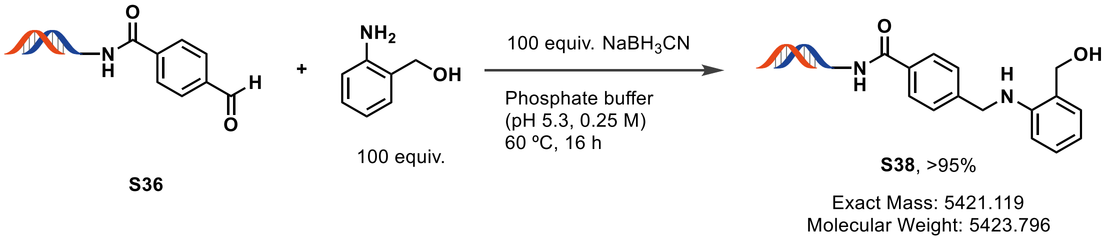

At 20–25 °C, 20 µL of HP–AOP–NHCOPhCOH **S36** (1.0 mM, 20 nmol, 1.0 equiv.) in phosphate buffer (pH 5.3, c = 250 mM) was added to a 1.5 mL Eppendorf tube. Next, 10 µL of a (2-aminophenyl)methanol stock solution (200 mM, 2.0 µmol, 100 equiv.) in DMA was added. The mixture was vortexed for 5 seconds. Then, 10 µL of a sodium cyanoborohydride stock solution (200 mM, 2.0 µmol, 100 equiv.) in MeCN was added. The mixture was vortexed for 5 seconds again, transferred into a pre-heated Thermocycler at 60 °C, and incubated at 60 °C for 16 hours at 1000 rpm. After 16 hours, an aliquot of 1 µL of the reaction mixture was diluted to 40 µL with water for LC–MS analysis.

Next, 4 µL of a 5 M solution of NaCl in water and 130 µL of ethanol at −20 °C were added to precipitate the DNA conjugate. The Eppendorf tube was placed in the freezer (−20 °C) for at least 1 hour, and then it was centrifuged at 4 °C and 10000 x g for at least 30 minutes. The supernatant was removed, the pellet was redissolved in 35 µL of water, and the procedure was repeated again. The remaining pellet was then dried under a flow of nitrogen, redissolved with 10 µL of water and stored in the freezer at −20 °C.

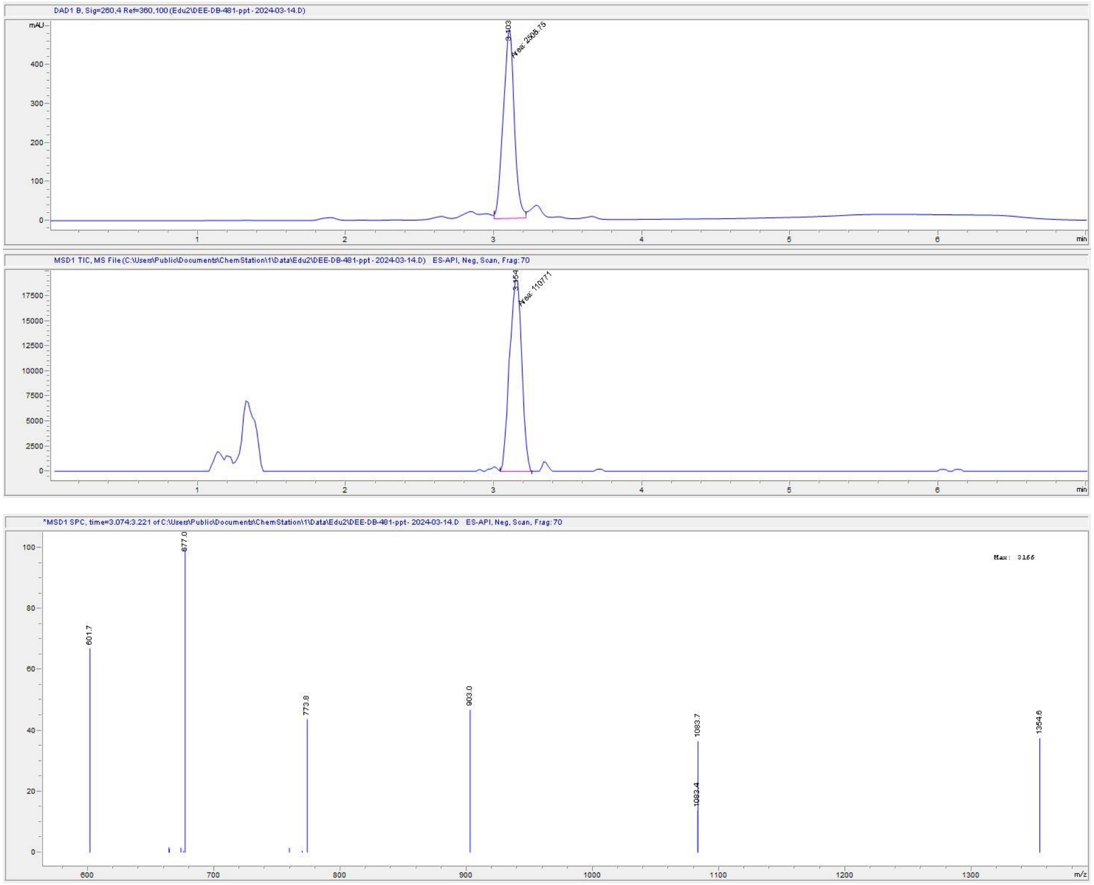

Figure S35. Analytical HPLC trace of **S38** with HPLC Method B. (Up) DAD chromatogram at 260 nm. (Middle) TIC chromatogram. (Below) Ionization of peak at 3.154 min containing reaction product.

DNA-conjugated arene S39

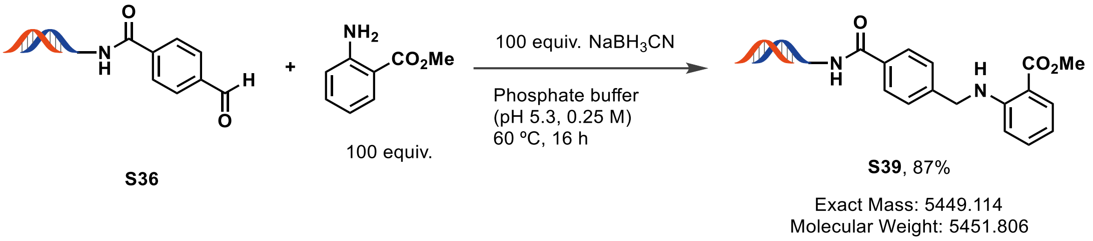

At 20–25 °C, 20 µL of HP–AOP–NHCOPhCOH **S36** (1.0 mM, 20 nmol, 1.0 equiv.) in phosphate buffer (pH 5.3, c = 250 mM) was added to a 1.5 mL Eppendorf tube. Next, 10 µL of a methyl 2-aminobenzoate stock solution (200 mM, 2.0 µmol, 100 equiv.) in DMA was added. The mixture was vortexed for 5 seconds. Then, 10 µL of a sodium cyanoborohydride stock solution (200 mM, 2.0 µmol, 100 equiv.) in MeCN was added. The mixture was vortexed for 5 seconds again, transferred into a pre-heated Thermocycler at 60 °C, and incubated at 60 °C for 16 hours at 1000 rpm. After 16 hours, an aliquot of 1 µL of the reaction mixture was diluted to 40 µL with water for LC–MS analysis.

Next, 4 µL of a 5 M solution of NaCl in water and 130 µL of ethanol at −20 °C were added to precipitate the DNA conjugate. The Eppendorf tube was placed in the freezer (−20 °C) for at least 1 hour, and then it was centrifuged at 4 °C and 10000 x g for at least 30 minutes. The supernatant was removed, the pellet was redissolved in 35 µL of water, and the procedure was repeated again. The remaining pellet was then dried under a flow of nitrogen, redissolved with 10 µL of water and stored in the freezer at −20 °C.

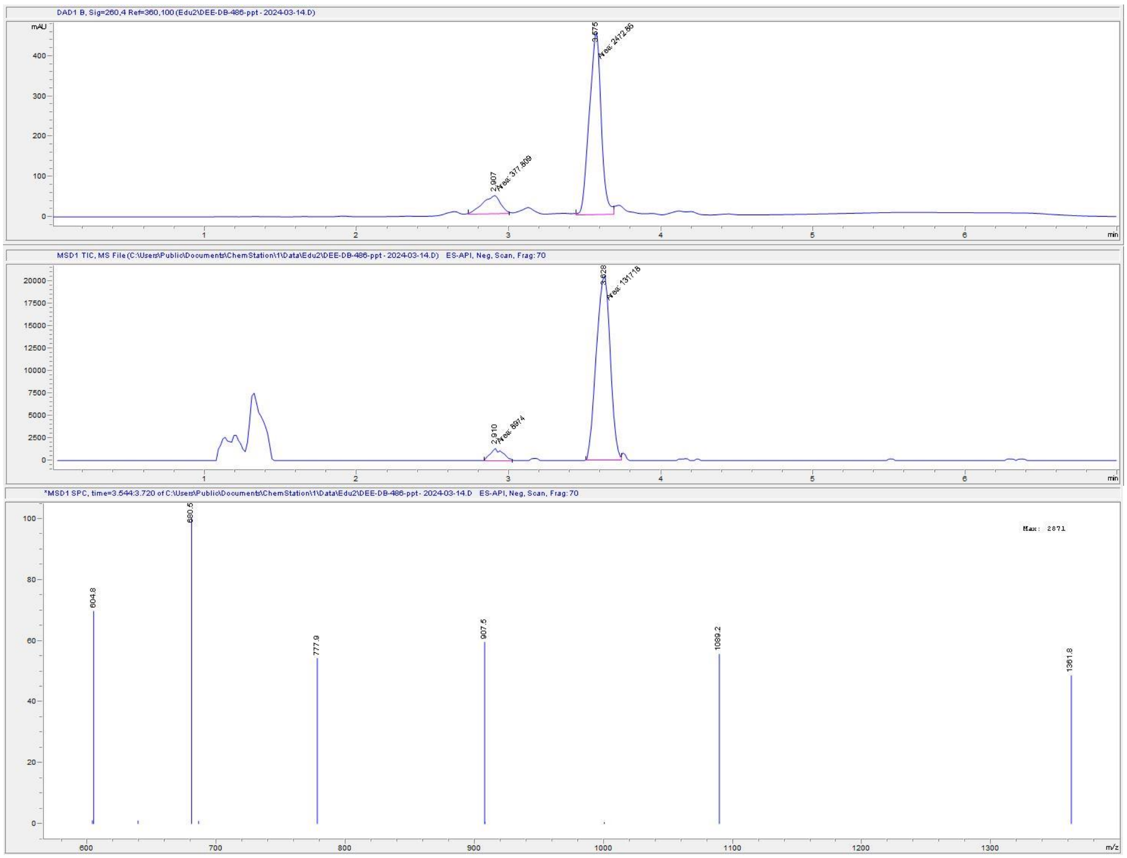

Figure S36. Analytical HPLC trace of **S39** with HPLC Method B. (Up) DAD chromatogram at 260 nm. (Middle) TIC chromatogram. (Below) Ionization of peak at 3.628 min containing reaction product.

DNA-conjugated arene S40

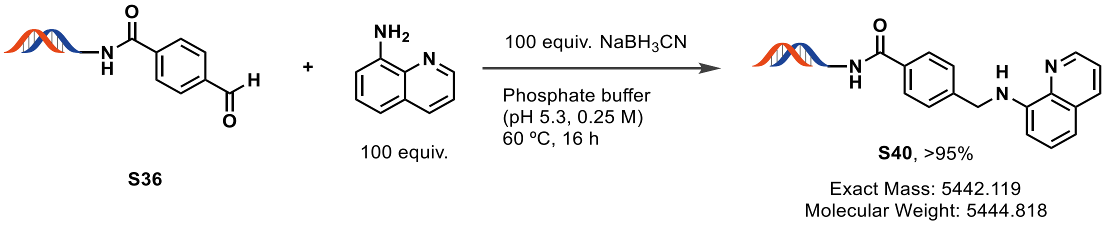

At 20–25 °C, 20 µL of HP–AOP–NHCOPhCOH **S36** (1.0 mM, 20 nmol, 1.0 equiv.) in phosphate buffer (pH 5.3, c = 250 mM) was added to a 1.5 mL Eppendorf tube. Next, 10 µL of an 8-aminoquinoline stock solution (200 mM, 2.0 µmol, 100 equiv.) in DMA was added. The mixture was vortexed for 5 seconds. Then, 10 µL of a sodium cyanoborohydride stock solution (200 mM, 2.0 µmol, 100 equiv.) in MeCN was added. The mixture was vortexed for 5 seconds again, transferred into a pre-heated Thermocycler at 60 °C, and incubated at 60 °C for 16 hours at 1000 rpm. After 16 hours, an aliquot of 1 µL of the reaction mixture was diluted to 40 µL with water for LC–MS analysis.

Next, 4 µL of a 5 M solution of NaCl in water and 130 µL of ethanol at −20 °C were added to precipitate the DNA conjugate. The Eppendorf tube was placed in the freezer (−20 °C) for at least 1 hour, and then it was centrifuged at 4 °C and 10000 x g for at least 30 minutes. The supernatant was removed, the pellet was redissolved in 35 µL of water, and the procedure was repeated again. The remaining pellet was then dried under a flow of nitrogen, redissolved with 10 µL of water and stored in the freezer at −20 °C.

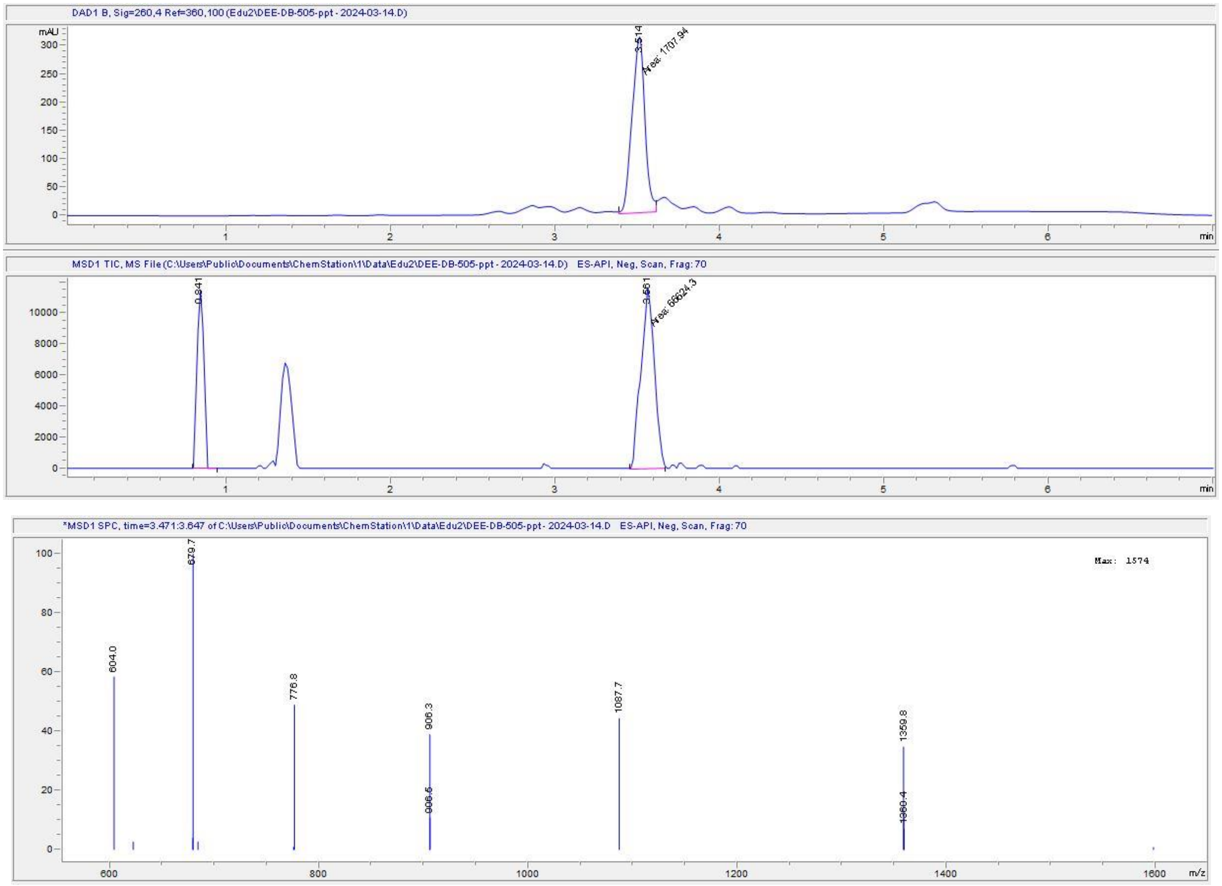

Figure S37. Analytical HPLC trace of **S40** with HPLC Method B. (Up) DAD chromatogram at 260 nm. (Middle) TIC chromatogram. (Below) Ionization of peak at 3.561 min containing reaction product.

DNA-conjugated arene S41

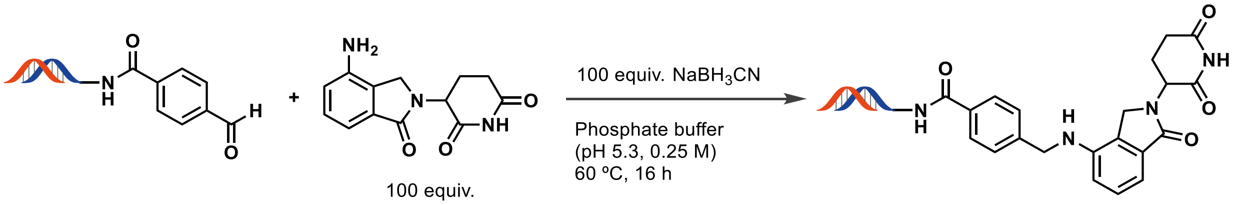

S36

S41, 80%

Exact Mass: 5557.146
Molecular Weight: 5559.906

At 20–25 °C, 20 µL of HP–AOP–NHCOPhCOH **S36** (1.0 mM, 20 nmol, 1.0 equiv.) in phosphate buffer (pH 5.3, c = 250 mM) was added to a 1.5 mL Eppendorf tube. Next, 10 µL of a lenalidomide stock solution (200 mM, 2.0 µmol, 100 equiv.) in DMA was added. The mixture was vortexed for 5 seconds. Then, 10 µL of a sodium cyanoborohydride stock solution (200 mM, 2.0 µmol, 100 equiv.) in MeCN was added. The mixture was vortexed for 5 seconds again, transferred into a pre-heated Thermocycler at 60 °C, and incubated at 60 °C for 16 hours at 1000 rpm. After 16 hours, an aliquot of 1 µL of the reaction mixture was diluted to 40 µL with water for LC–MS analysis.

Next, 4 µL of a 5 M solution of NaCl in water and 130 µL of ethanol at −20 °C were added to precipitate the DNA conjugate. The Eppendorf tube was placed in the freezer (−20 °C) for at least 1 hour, and then it was centrifuged at 4 °C and 10000 x g for at least 30 minutes. The supernatant was removed, the pellet was redissolved in 35 µL of water, and the procedure was repeated again. The remaining pellet was then dried under a flow of nitrogen, redissolved with 10 µL of water and stored in the freezer at −20 °C.

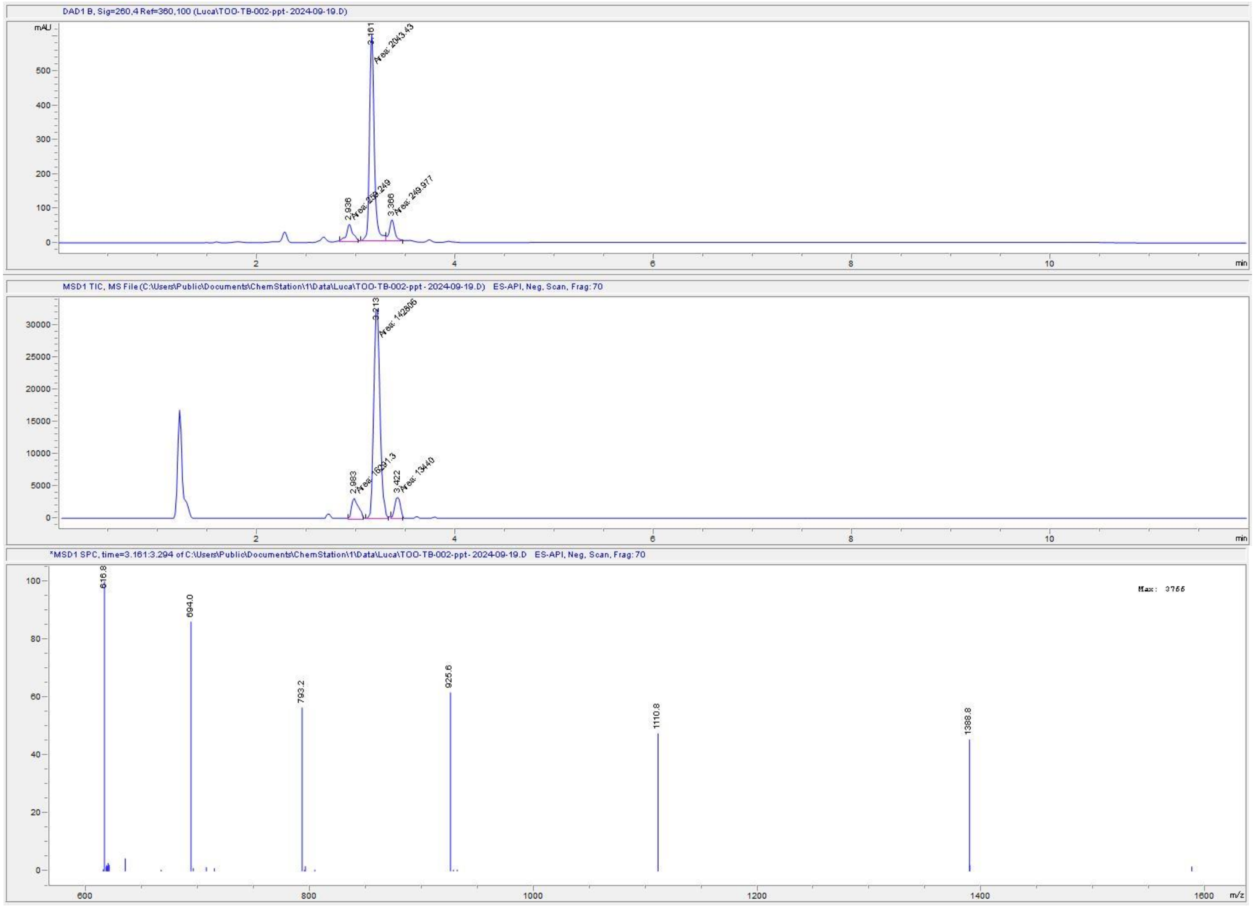

Figure S38. Analytical HPLC trace of **S41** with HPLC Method A. (Up) DAD chromatogram at 260 nm. (Middle) TIC chromatogram. (Below) Ionization of peak at 3.213 min containing reaction product.

DNA-conjugated arene S42

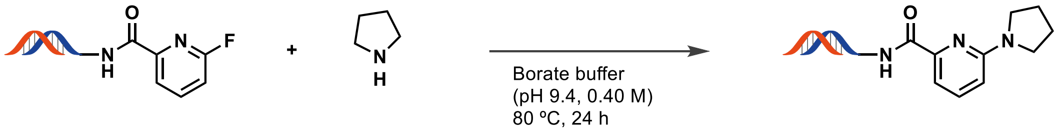

S32 500 equiv. **S42**, >95%

Exact Mass: 5356.104
Molecular Weight: 5358.725

At 20–25 °C, 80 µL of **S32** (0.67 mM, 40 nmol, 1.0 equiv.) in borate buffer (pH 9.4, c = 400 mM) was added to a 1.5 mL Eppendorf tube. Next, 40 µL of a pyrrolidine stock solution (500 mM, 20.0 µmol, 500 equiv.) in DMSO was added. The mixture was vortexed for 5 seconds again, transferred into a pre-heated Thermocycler at 80 °C, and incubated at 80 °C for 16 hours at 1000 rpm. After 24 hours, an aliquot of 1 µL of the reaction mixture was diluted to 40 µL with water for LC–MS analysis.

Next, 12 µL of a 5 M solution of NaCl in water and 400 µL of ethanol at −20 °C were added to precipitate the DNA conjugate. The Eppendorf tube was placed in the freezer (−20 °C) for at least 1 hour, and then it was centrifuged at 4 °C and 10000 x g for at least 30 minutes. The supernatant was removed, the pellet was redissolved in 120 µL of water, and the procedure was repeated again. The remaining pellet was then dried under a flow of nitrogen, redissolved with 20 µL of water and stored in the freezer at −20 °C.

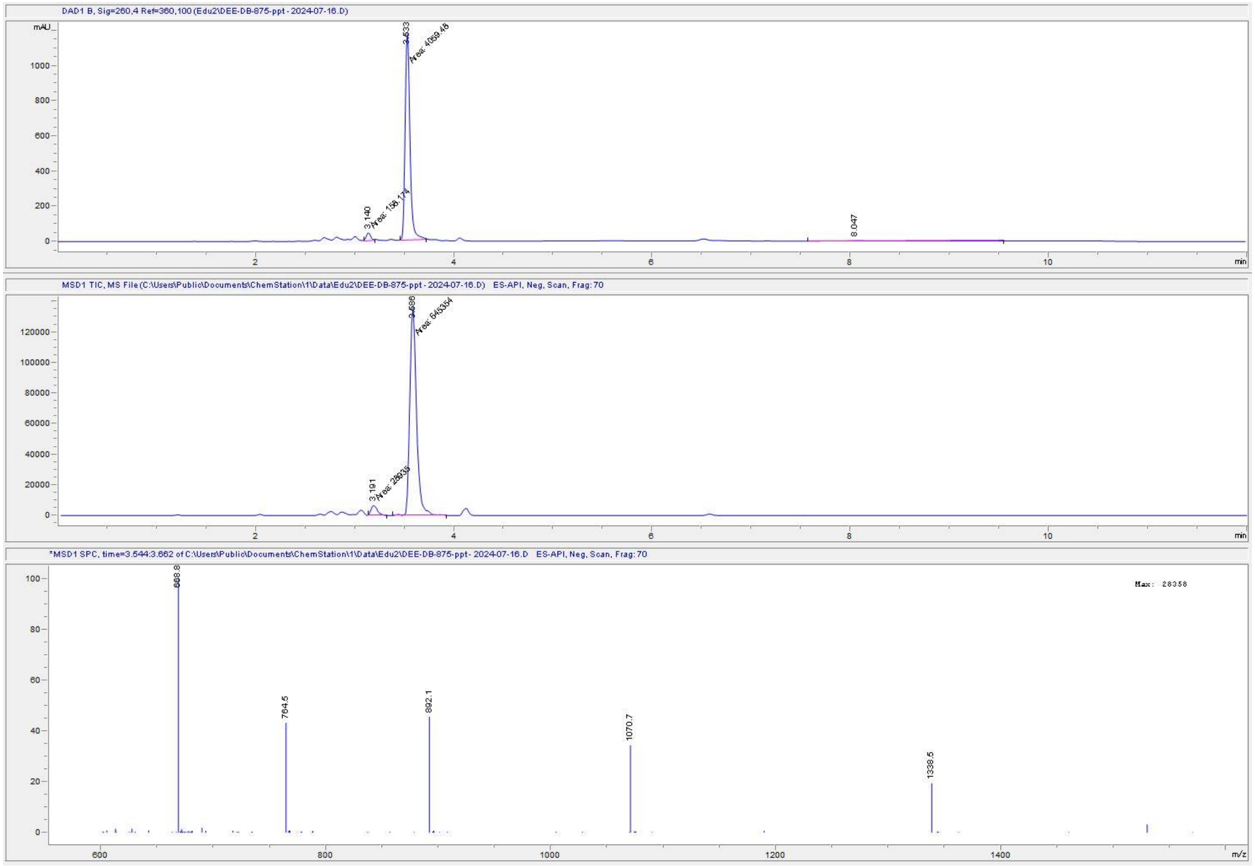

Figure S39. Analytical HPLC trace of **S42** with HPLC Method A. (Up) DAD chromatogram at 260 nm. (Middle) TIC chromatogram. (Below) Ionization of peak at 3.586 min containing reaction product.

DNA-conjugated arene S43

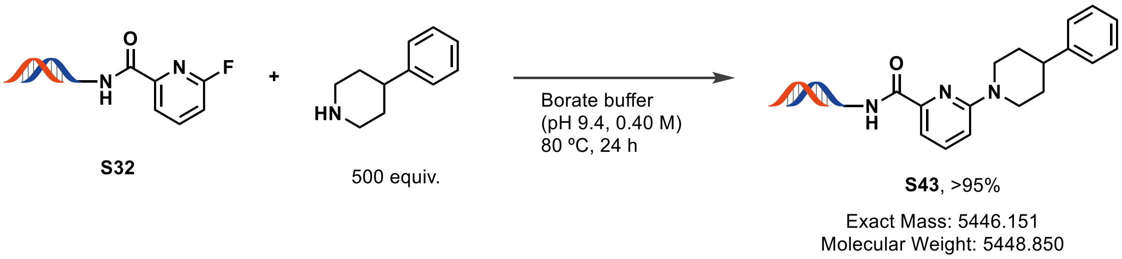

At 20–25 °C, 80 µL of **S32** (0.67 mM, 40 nmol, 1.0 equiv.) in borate buffer (pH 9.4, c = 400 mM) was added to a 1.5 mL Eppendorf tube. Next, 40 µL of a 4-phenylpiperidine stock solution (500 mM, 20.0 µmol, 500 equiv.) in DMSO was added. The mixture was vortexed for 5 seconds again, transferred into a pre-heated Thermocycler at 80 °C, and incubated at 80 °C for 16 hours at 1000 rpm. After 24 hours, an aliquot of 1 µL of the reaction mixture was diluted to 40 µL with water for LC–MS analysis.

Next, 12 µL of a 5 M solution of NaCl in water and 400 µL of ethanol at −20 °C were added to precipitate the DNA conjugate. The Eppendorf tube was placed in the freezer (−20 °C) for at least 1 hour, and then it was centrifuged at 4 °C and 10000 x g for at least 30 minutes. The supernatant was removed, the pellet was redissolved in 120 µL of water, and the procedure was repeated again. The remaining pellet was then dried under a flow of nitrogen, redissolved with 20 µL of water and stored in the freezer at −20 °C.

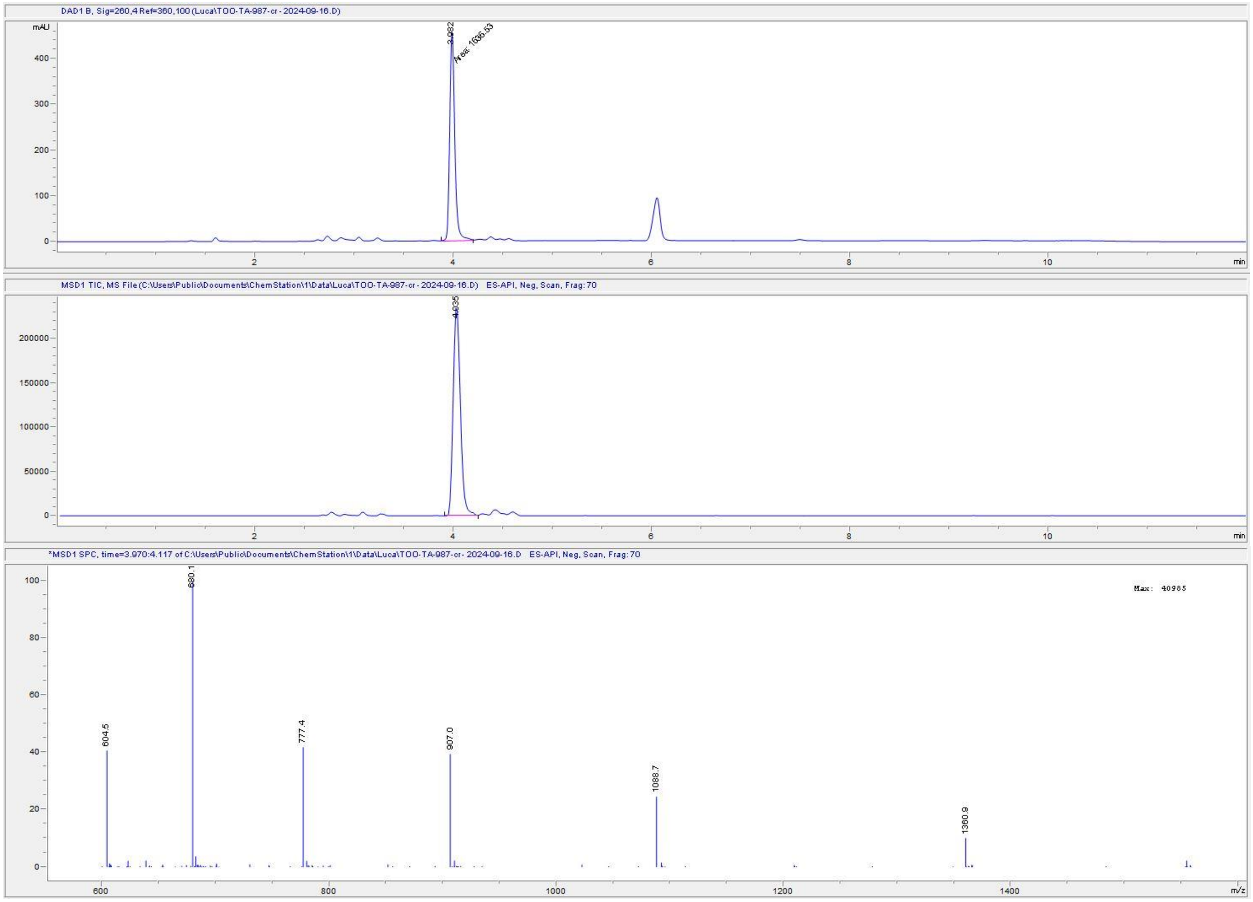

Figure S40. Analytical HPLC trace of **S43** with HPLC Method A. (Up) DAD chromatogram at 260 nm. (Middle) TIC chromatogram. (Below) Ionization of peak at 4.035 min containing reaction product.

DNA-conjugated arene S44

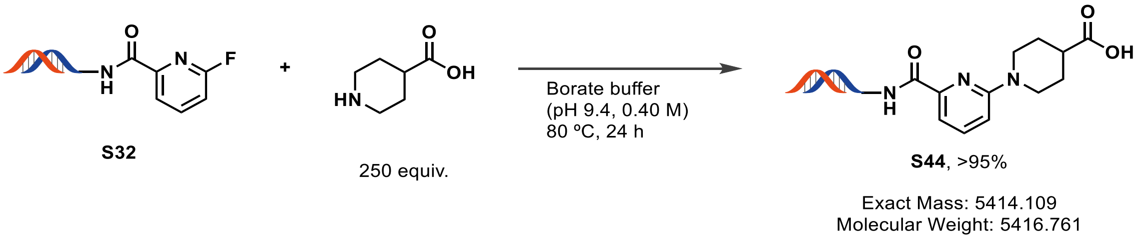

At 20–25 °C, 80 µL of **S32** (0.67 mM, 40 nmol, 1.0 equiv.) in borate buffer (pH 9.4, c = 400 mM) was added to a 1.5 mL Eppendorf tube. Next, 40 µL of a piperidine-4-carboxylic acid stock solution (500 mM, 20.0 µmol, 500 equiv.) in DMSO was added. The mixture was vortexed for 5 seconds again, transferred into a pre-heated Thermocycler at 80 °C, and incubated at 80 °C for 16 hours at 1000 rpm. After 24 hours, an aliquot of 1 µL of the reaction mixture was diluted to 40 µL with water for LC–MS analysis.

Next, 12 µL of a 5 M solution of NaCl in water and 400 µL of ethanol at −20 °C were added to precipitate the DNA conjugate. The Eppendorf tube was placed in the freezer (−20 °C) for at least 1 hour, and then it was centrifuged at 4 °C and 10000 x g for at least 30 minutes. The supernatant was removed, the pellet was redissolved in 120 µL of water, and the procedure was repeated again. The remaining pellet was then dried under a flow of nitrogen, redissolved with 20 µL of water and stored in the freezer at −20 °C.

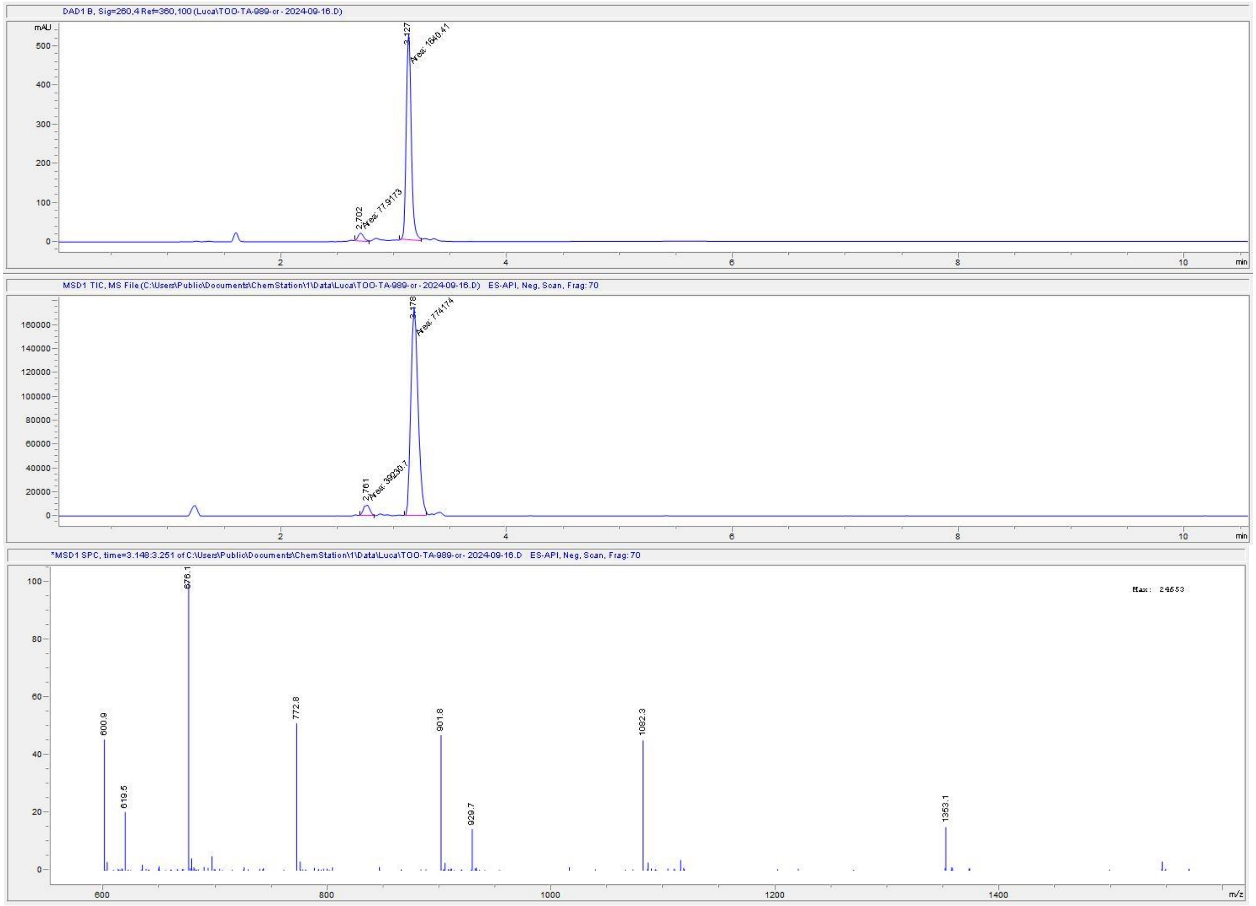

Figure S41. Analytical HPLC trace of **S44** with HPLC Method A. (Up) DAD chromatogram at 260 nm. (Middle) TIC chromatogram. (Below) Ionization of peak at 3.178 min containing reaction product.

DNA-conjugated arene S45

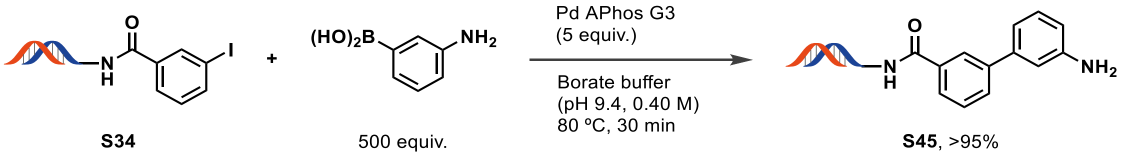

S34 500 equiv. **S45**, >95%

Exact Mass: 5557.146
Molecular Weight: 5559.906

At 20–25 °C, 25 µL of **S34** (1.0 mM, 25 nmol, 1.0 equiv.) in water was added to a 1.5 mL Eppendorf tube. Next, 50 µL of Borate buffer (pH 9.4, 500 mM) was added. 6.2 µL of an APhos Pd G3 stock solution (20 mM, 125 nmol, 5.0 equiv.) in DMA was added over the solution of **S34**. The mixture was vortexed for 5 seconds. Lastly, 25 µL of a (3-aminophenyl)boronic acid stock solution (500 mM, 12.5 µmol, 500 equiv.) in DMA was added. The mixture was vortexed for 5 seconds, transferred into a Thermocycler pre-heated at 80 °C, and incubated at 80 °C for 30 min. at 600 rpm.

After 15 minutes, 10 µL of a 100 mM solution of sodium diethyldithiocarbamate trihydrate in water were added to remove the palladium salts from the solution. The reaction mixture was centrifuged, and the supernatant was diluted to 200 µL with water. Over the sample, 20 µL of a 5 M solution of NaCl in water and 600 µL of ethanol at −20 °C were added to precipitate the DNA conjugate. The Eppendorf tube was placed in the freezer (−20 °C) for at least 1 hour, and then it was centrifuged at 4 °C and 10000 x g for at least 30 minutes. The remaining pellet was then dried under a flow of nitrogen, redissolved with 12.5 µL of water to 2.0 mM concentration and stored in the freezer at −20 °C.

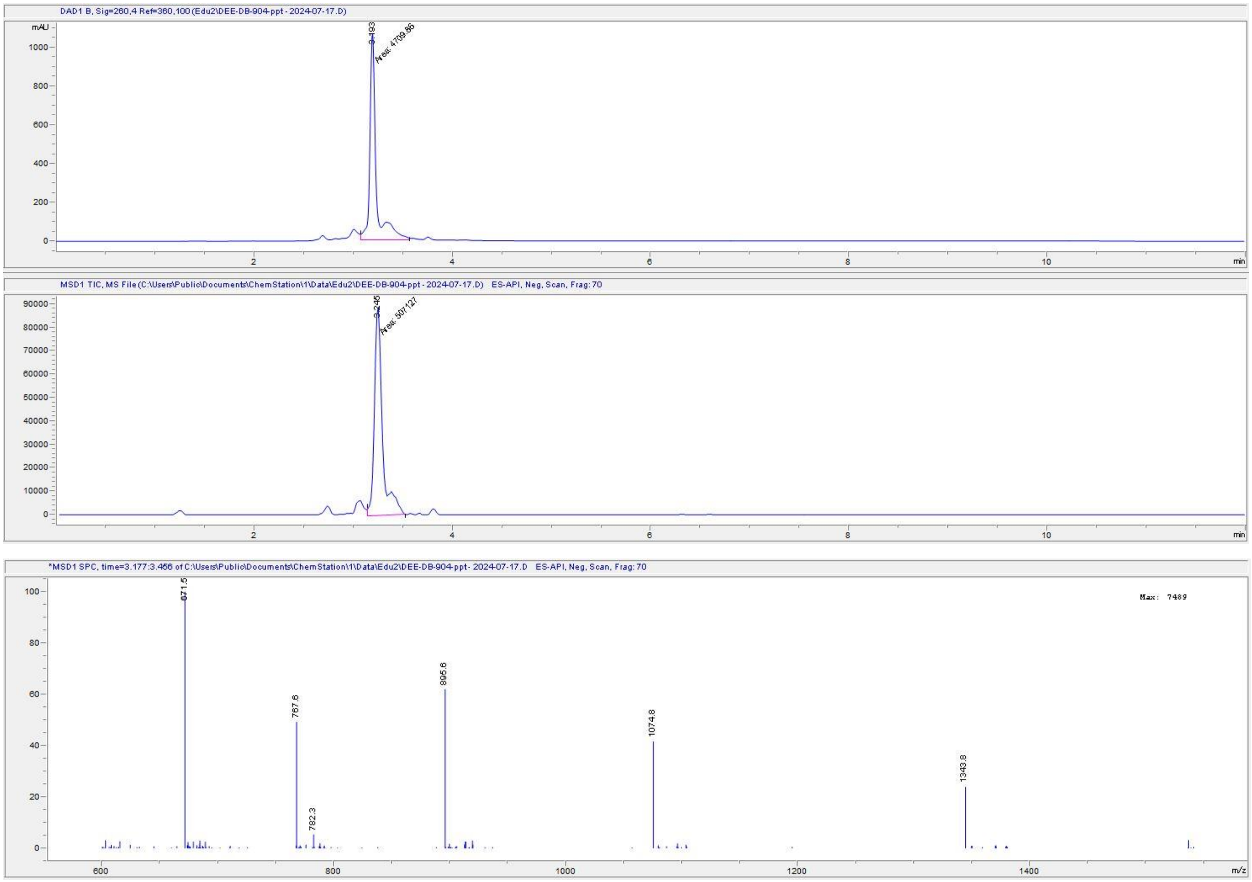

Figure S42. Analytical HPLC trace of **S45** with HPLC Method A. (Up) DAD chromatogram at 260 nm. (Middle) TIC chromatogram. (Below) Ionization of peak at 3.245 min containing reaction product.

DNA-conjugated C−H functionalization with selenoxide reagent

General procedure for C−H functionalization of DNA-conjugated arenes at pH 3.5

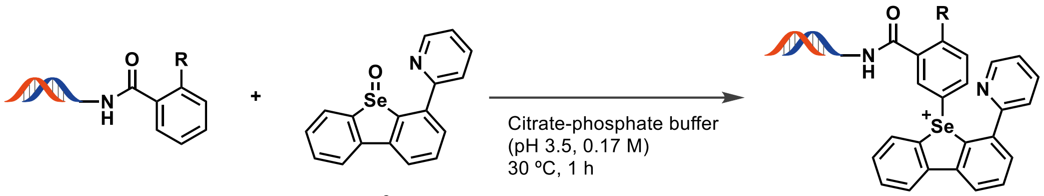

3
2 equiv.

At 20–25 °C, 1.0 µL of DNA-conjugated arene (2.0 mM, 2.0 nmol, 1.0 equiv.) in water was added to a 1.5 mL Eppendorf tube. Next, 1.0 µL of Citrate-phosphate buffer (pH 3.5, c = 500 mM) was added. Then, 1.0 µL of a selenoxide **3** stock solution (4.0 mM, 4.0 nmol, 2.0 equiv.) in water was added. The mixture was vortexed for 5 seconds, transferred into a Thermocycler pre-heated at 30 °C, and incubated at 30 °C for 1 hour at 600 rpm. After 1 hour, reaction was quenched by addition of 10 µL of borate buffer (pH 9.4, c = 500 mM), and an aliquot of 2 µL of the reaction mixture was diluted to 40 µL with water for LC–MS analysis.

Quenching the reaction mixture with a basic buffer (x2 volume of reaction mixture) prior to desalting was crucial to ensure DNA stability for storage over longer periods of time.

The solution of DNA conjugate was then desalted. DNA desalting and rebuffering was performed by charging the solution in an AMICON® filter unit from Sigma Aldrich (3 kD) in 300 µL of water, centrifuged at 4 °C and 10000 x g for at least 30 minutes, until the volume decreased to < 10 µL. Another 300 µL of water were added and the process was repeated all over again for at least 3 times. The remaining solution concentration was determined by A_{260} absorption using a Thermo Scientific™ NanoDrop™ One^{C}, concentration of the solution was adjusted to 2.0 mM and stored in the freezer at −20 °C.

General procedure for C−H functionalization of DNA-conjugated arenes at pH 3.0

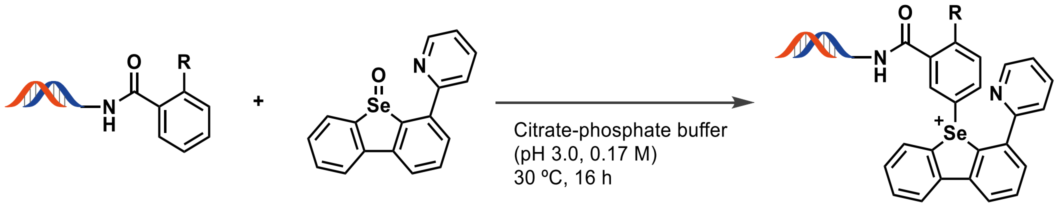

3
2 equiv.

At 20–25 °C, 1.0 µL of DNA-conjugated arene (2.0 mM, 2.0 nmol, 1.0 equiv.) in water was added to a 1.5 mL Eppendorf tube. Next, 1.0 µL of Citrate-phosphate buffer (pH 3.0, c = 500 mM) was added. Then, 1.0 µL of a selenoxide **3** stock solution (4.0 mM, 4.0 nmol, 2.0 equiv.) in water was added. The mixture was vortexed for 5 seconds, transferred into a Thermocycler pre-heated at 30 °C, and incubated at 30 °C for 16 hours at 600 rpm. After 1 hour, reaction was quenched by addition of 10 µL of borate buffer (pH 9.4, c = 500 mM), and an aliquot of 2 µL of the reaction mixture was diluted to 40 µL with water for LC–MS analysis.

Quenching the reaction mixture with a basic buffer (x2 volume of reaction mixture) prior to desalting was crucial to ensure DNA stability for storage over longer periods of time.

The solution of DNA conjugate was then desalted. DNA desalting and rebuffering was performed by

charging the solution in an AMICON® filter unit from Sigma Aldrich (3 kD) in 300 µL of water, centrifuged at 4 °C and 10000 x g for at least 30 minutes, until the volume decreased to < 10 µL. Another 300 µL of water were added and the process was repeated all over again for at least 3 times. The remaining solution concentration was determined by A_{260} absorption using a Thermo Scientific™ NanoDrop™ One^{C}, concentration of the solution was adjusted to 2.0 mM and stored in the freezer at −20 °C.

General procedure for C−H functionalization of DNA conjugates with excess of Selenoxide

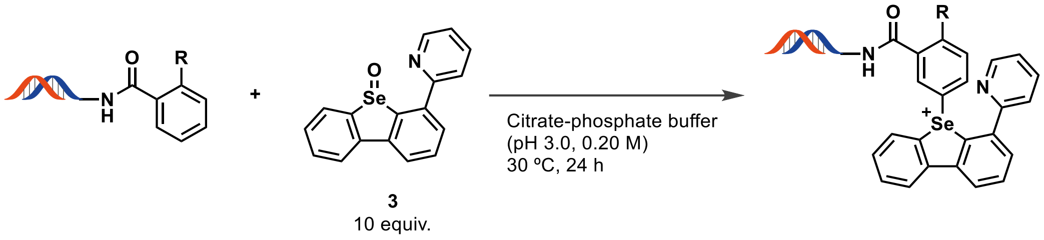

At 20–25 °C, 1.0 µL of DNA-conjugated arene (2.0 mM, 2.0 nmol, 1.0 equiv.) in water was added to a 1.5 mL Eppendorf tube. Next, 4.0 µL of Citrate-phosphate buffer (pH 3.0, c = 500 mM) was added. Then, 5.0 µL of a selenoxide **3** stock solution (4.0 mM, 20 nmol, 10 equiv.) in water was added. The mixture was vortexed for 5 seconds, transferred into a Thermocycler pre-heated at 30 °C, and incubated at 30 °C for 24 hours at 600 rpm. After 3 hours, reaction was quenched by addition of 10 µL of borate buffer (pH 9.4, c = 500 mM), and an aliquot of 2 µL of the reaction mixture was diluted to 40 µL with water for LC–MS analysis.

Quenching the reaction mixture with a basic buffer (x2 volume of reaction mixture) prior to desalting was crucial to ensure DNA stability for storage over longer periods of time.

The solution of DNA conjugate was then desalted. DNA desalting and rebuffering was performed by charging the solution in an AMICON® filter unit from Sigma Aldrich (3 kD) in 300 µL of water, centrifuged at 4 °C and 10000 x g for at least 30 minutes, until the volume decreased to < 10 µL. Another 300 µL of water were added and the process was repeated all over again for at least 3 times. The remaining solution concentration was determined by A_{260} absorption using a Thermo Scientific™ NanoDrop™ One^{C}, concentration of the solution was adjusted to 2.0 mM and stored in the freezer at −20 °C.

General procedure for C−H functionalization of less activated DNA conjugates

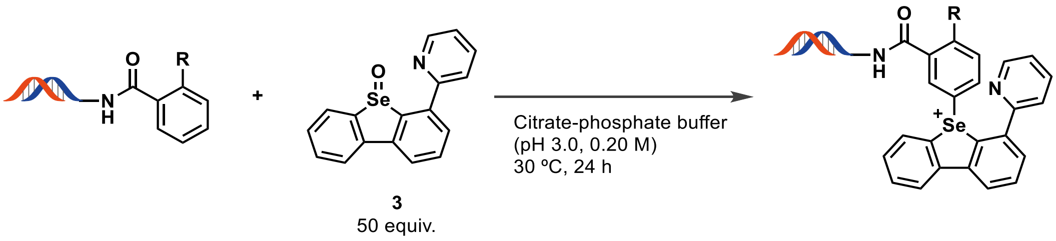

At 20–25 °C, 1.0 µL of DNA-conjugated arene (2.0 mM, 2.0 nmol, 1.0 equiv.) in water was added to a 1.5 mL Eppendorf tube. Next, 9.0 µL of Citrate-phosphate buffer (pH 3.0, c = 500 mM) was added. Then, 10 µL of a selenoxide **3** stock solution (10 mM, 100 nmol, 50 equiv.) in water was added. The mixture was vortexed for 5 seconds, transferred into a Thermocycler pre-heated at 30 °C, and incubated at 30 °C for 24 hours at 600 rpm. After 24 hours, reaction was quenched by addition of 20 µL of borate buffer (pH 9.4, c = 500 mM), and an aliquot of 2 µL of the reaction mixture was diluted to 40 µL with water for

LC–MS analysis.

Quenching the reaction mixture with a basic buffer (x2 volume of reaction mixture) prior to desalting was crucial to ensure DNA stability for storage over longer periods of time.

The solution of DNA conjugate was then desalted. DNA desalting and rebuffering was performed by charging the solution in an AMICON® filter unit from Sigma Aldrich (3 kD) in 300 µL of water, centrifuged at 4 °C and 10000 x g for at least 30 minutes, until the volume decreased to < 10 µL. Another 300 µL of water were added and the process was repeated all over again for at least 3 times. The remaining solution concentration was determined by A_{260} absorption using a Thermo Scientific™ NanoDrop™ One^{C}, concentration of the solution was adjusted to 2.0 mM and stored in the freezer at −20 °C.

General procedure for C−H functionalization of DNA-conjugated arenes at pH 2.0

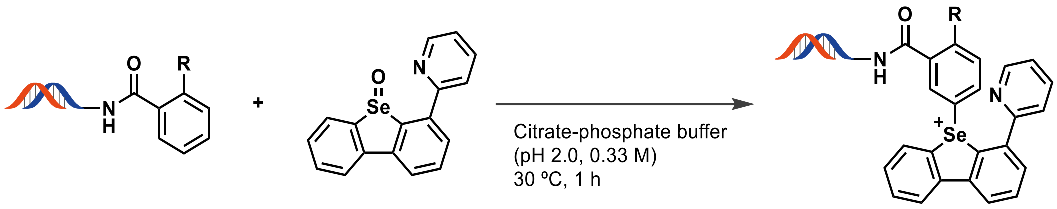

3
2 equiv.

At 20–25 °C, 1.0 µL of DNA-conjugated arene (2.0 mM, 2.0 nmol, 1.0 equiv.) in water was added to a 1.5 mL Eppendorf tube. Next, 1.0 µL of Citrate-phosphate buffer (pH 2.0, c = 1.00 M) was added. Then, 1.0 µL of a selenoxide **3** stock solution (4.0 mM, 4.0 nmol, 2.0 equiv.) in water was added. The mixture was vortexed for 5 seconds, transferred into a Thermocycler pre-heated at 30 °C, and incubated at 30 °C for 16 hours at 600 rpm. After 1 hour, reaction was quenched by addition of 10 µL of borate buffer (pH 9.4, c = 500 mM), and an aliquot of 2 µL of the reaction mixture was diluted to 40 µL with water for LC–MS analysis.

Quenching the reaction mixture with a basic buffer (x2 volume of reaction mixture) prior to desalting was crucial to ensure DNA stability for storage over longer periods of time.

The solution of DNA conjugate was then desalted. DNA desalting and rebuffering was performed by charging the solution in an AMICON® filter unit from Sigma Aldrich (3 kD) in 300 µL of water, centrifuged at 4 °C and 10000 x g for at least 30 minutes, until the volume decreased to < 10 µL. Another 300 µL of water were added and the process was repeated all over again for at least 3 times. The remaining solution concentration was determined by A_{260} absorption using a Thermo Scientific™ NanoDrop™ One^{C}, concentration of the solution was adjusted to 2.0 mM and stored in the freezer at −20 °C.

Stability of DNA-conjugated selenonium salts

On-DNA selenonium salts exhibit stability in aqueous solution at both 4°C and -20°C over extended periods of time, with samples remaining intact for as long as one year. While we have not detected hydrodefunctionalization under ambient light conditions, we advise against prolonged exposure of selenonium salt solutions to sunlight due to the potential for photochemical fragmentation. However, *quenching the reaction mixture with a basic buffer > pH 7 (x2 volume of reaction mixture) prior to storage, or desalting, is crucial to ensure DNA stability for storage over longer periods of time.*

DNA-conjugated C−H functionalization with selenoxide reagent 2

Synthesis of DNA-conjugated selenonium salt S46

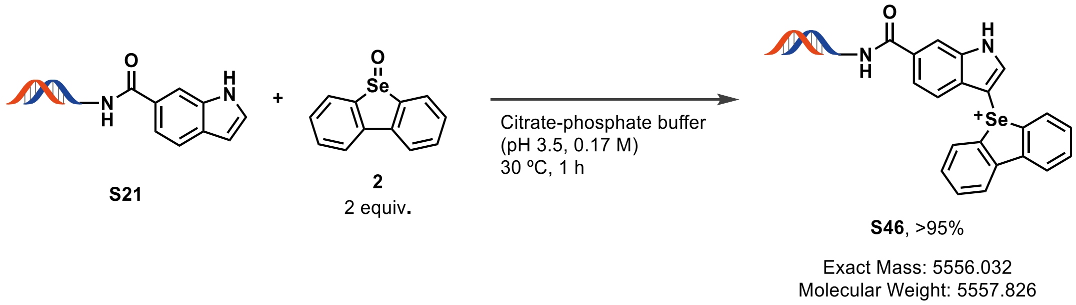

At 20–25 °C, 1.0 µL of **S21** (2.0 mM, 2.0 nmol, 1.0 equiv.) in water was added to a 1.5 mL Eppendorf tube. Next, 1.0 µL of Citrate-phosphate buffer (pH 3.5, c = 500 mM) was added. Then, 1.0 µL of a selenoxide **2** stock solution (4.0 mM, 4.0 nmol, 2.0 equiv.) in water was added. The mixture was vortexed for 5 seconds, transferred into a Thermocycler pre-heated at 30 °C, and incubated at 30 °C for 1 hour at 600 rpm. After 1 hour, reaction was quenched by addition of 10 µL of borate buffer (pH 9.4, c = 500 mM), and an aliquot of 2 µL of the reaction mixture was diluted to 40 µL with water for LC–MS analysis.

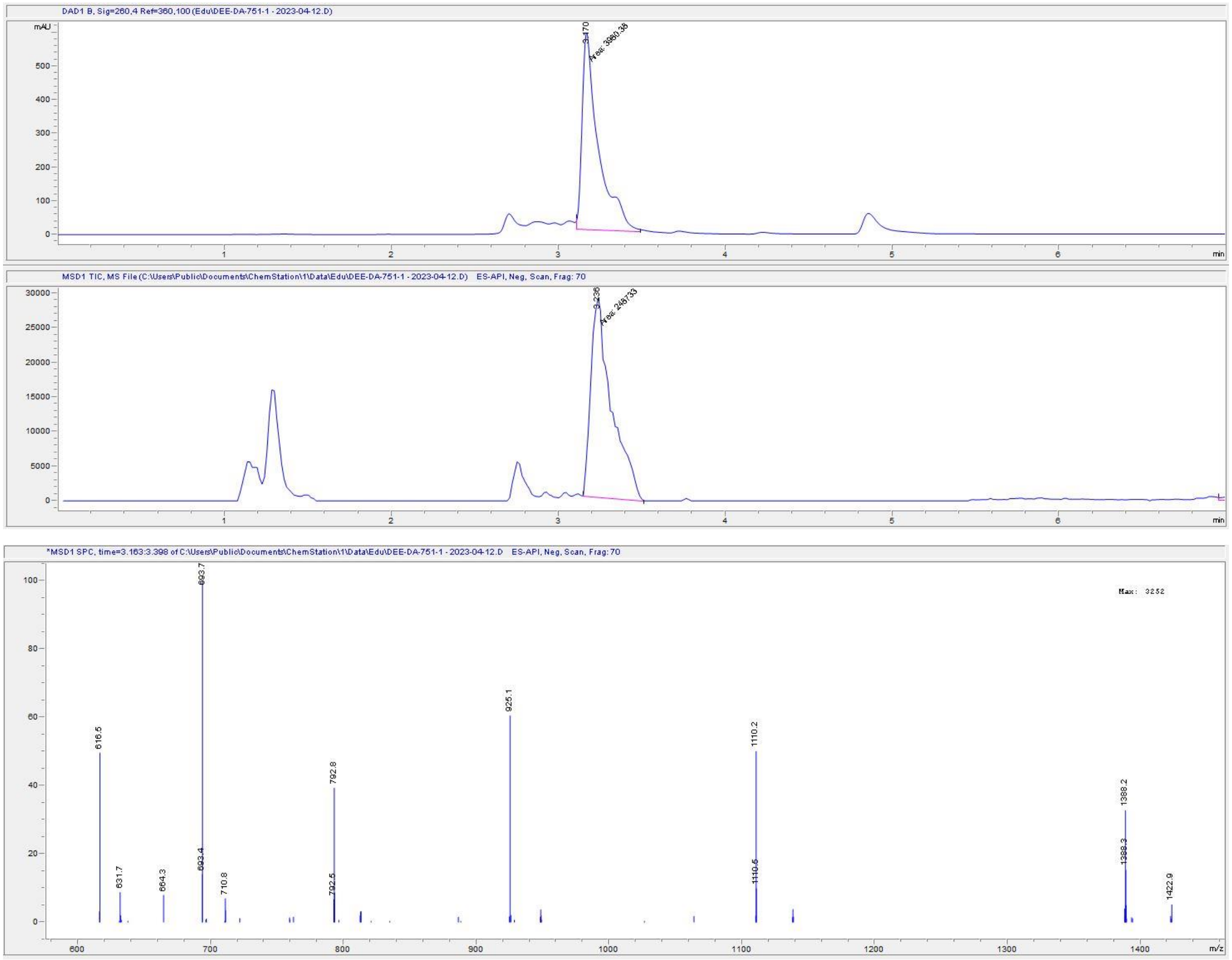

Figure S43. Analytical HPLC trace of **S46** with HPLC Method B. (Up) DAD chromatogram at 260 nm. (Middle) TIC chromatogram. (Below) Ionization of peak at 3.236 min containing reaction product.

Synthesis of DNA-conjugated selenonium salt S47

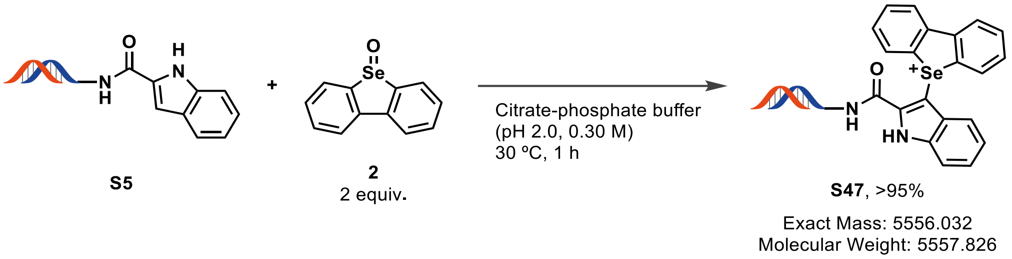

At 20–25 °C, 1.0 µL of **S5** (2.0 mM, 2.0 nmol, 1.0 equiv.) in water was added to a 1.5 mL Eppendorf tube. Next, 1.0 µL of Citrate-phosphate buffer (pH 2.0, c = 1.0 M) was added. Then, 1.0 µL of a selenoxide **2** stock solution (4.0 mM, 4.0 nmol, 2.0 equiv.) in water was added. The mixture was vortexed for 5 seconds, transferred into a Thermocycler pre-heated at 30 °C, and incubated at 30 °C for 1 hour at 600 rpm. After 1 hour, reaction was quenched by addition of 10 µL of borate buffer (pH 9.4, c = 500 mM), and an aliquot of 2 µL of the reaction mixture was diluted to 40 µL with water for LC–MS analysis.

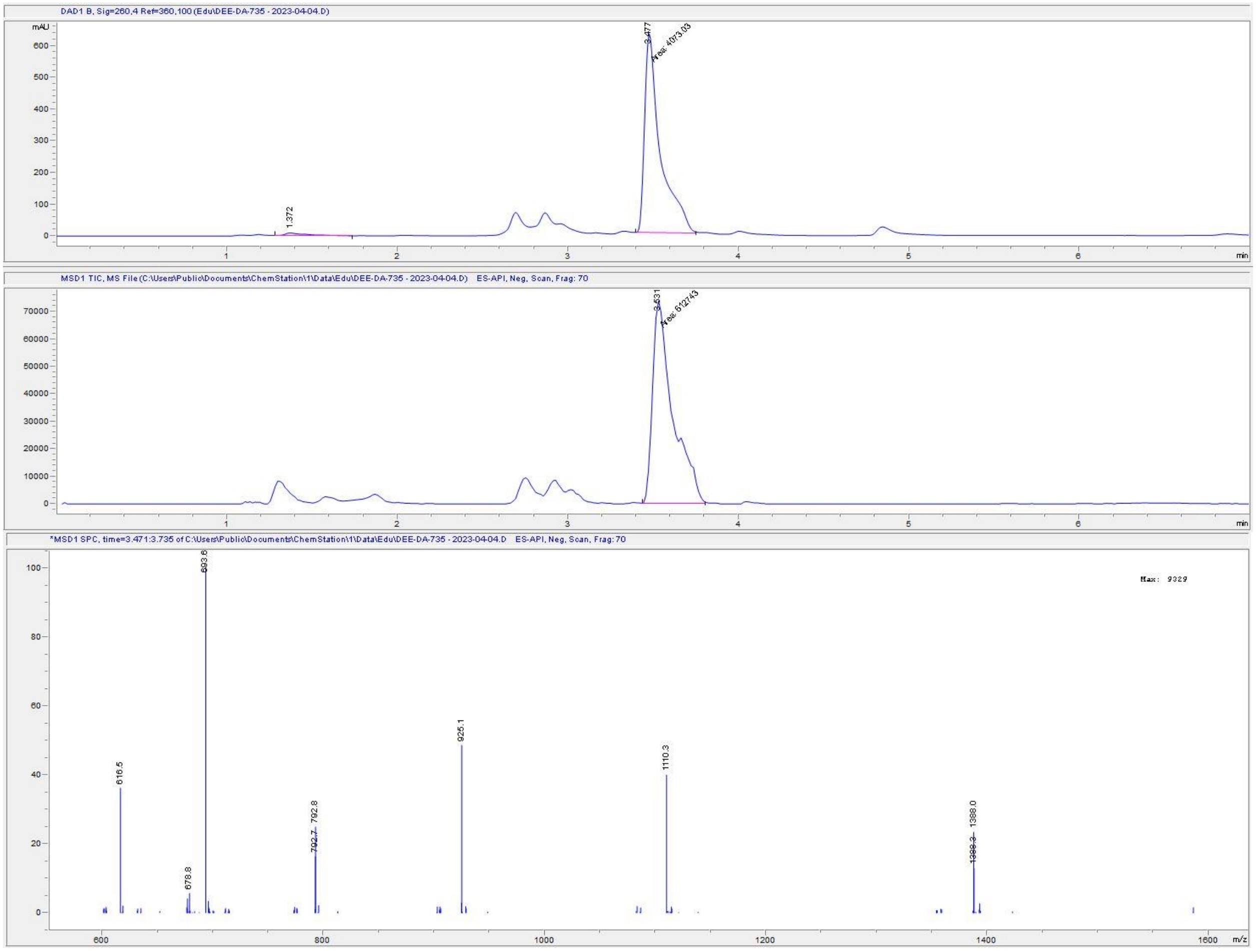

Figure S44. Analytical HPLC trace of **S47** with HPLC Method B. (Up) DAD chromatogram at 260 nm. (Middle) TIC chromatogram. (Below) Ionization of peak at 3.531 min containing reaction product.

Synthesis of DNA-conjugated selenonium salt S48

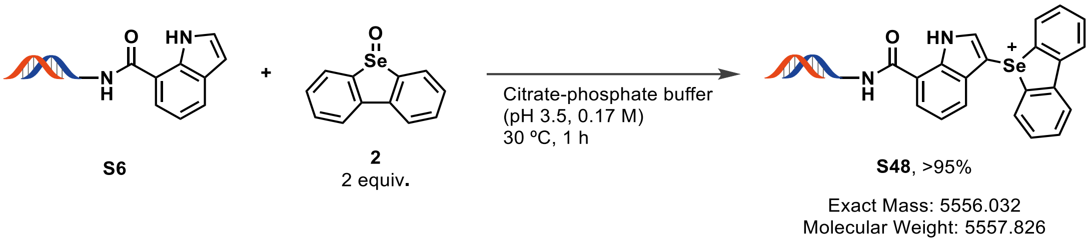

At 20–25 °C, 1.0 µL of **S6** (2.0 mM, 2.0 nmol, 1.0 equiv.) in water was added to a 1.5 mL Eppendorf tube. Next, 1.0 µL of Citrate-phosphate buffer (pH 3.5, c = 500 mM) was added. Then, 1.0 µL of a selenoxide **2** stock solution (4.0 mM, 4.0 nmol, 2.0 equiv.) in water was added. The mixture was vortexed for 5 seconds, transferred into a Thermocycler pre-heated at 30 °C, and incubated at 30 °C for 1 hour at 600 rpm. After 1 hour, reaction was quenched by addition of 10 µL of borate buffer (pH 9.4, c = 500 mM), and an aliquot of 2 µL of the reaction mixture was diluted to 40 µL with water for LC–MS analysis.

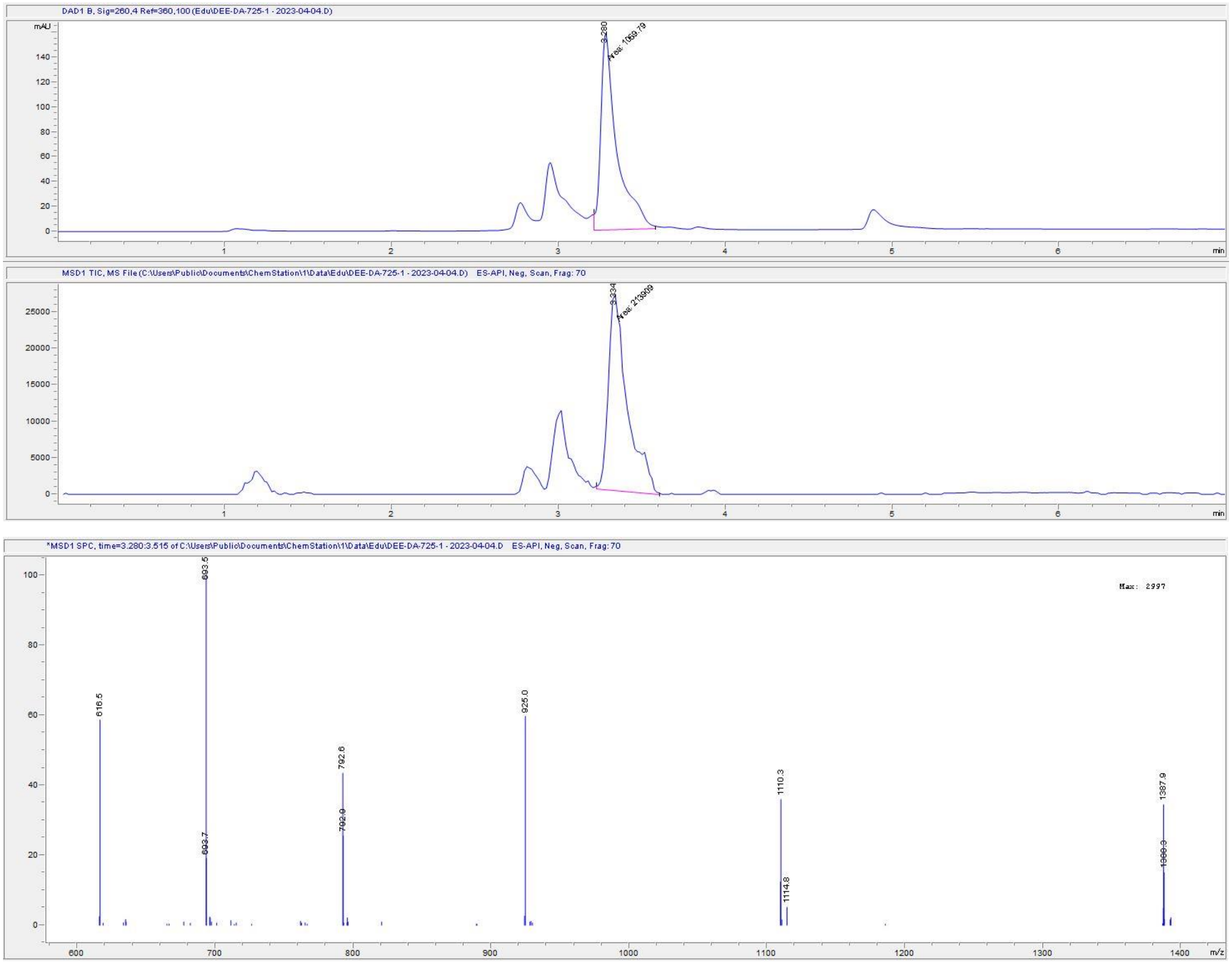

Figure S45. Analytical HPLC trace of **S48** with HPLC Method B. (Up) DAD chromatogram at 260 nm. (Middle) TIC chromatogram. (Below) Ionization of peak at 3.334 min containing reaction product.

Synthesis of DNA-conjugated selenonium salt S49

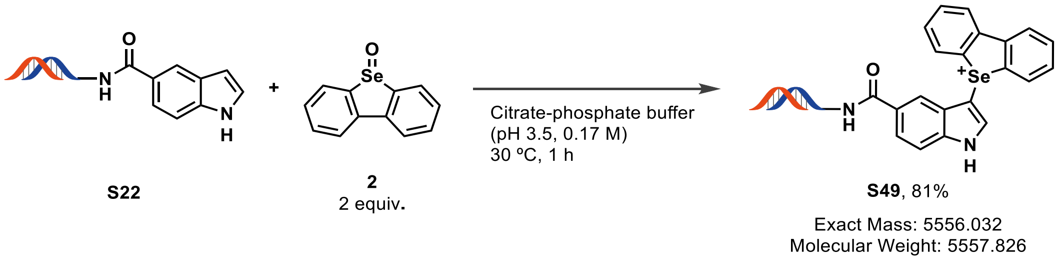

At 20–25 °C, 1.0 µL of **S22** (2.0 mM, 2.0 nmol, 1.0 equiv.) in water was added to a 1.5 mL Eppendorf tube. Next, 1.0 µL of Citrate-phosphate buffer (pH 3.5, c = 500 mM) was added. Then, 1.0 µL of a selenoxide **2** stock solution (4.0 mM, 4.0 nmol, 2.0 equiv.) in water was added. The mixture was vortexed for 5 seconds, transferred into a Thermocycler pre-heated at 30 °C, and incubated at 30 °C for 1 hour at 600 rpm. After 1 hour, reaction was quenched by addition of 10 µL of borate buffer (pH 9.4, c = 500 mM), and an aliquot of 2 µL of the reaction mixture was diluted to 40 µL with water for LC–MS analysis.

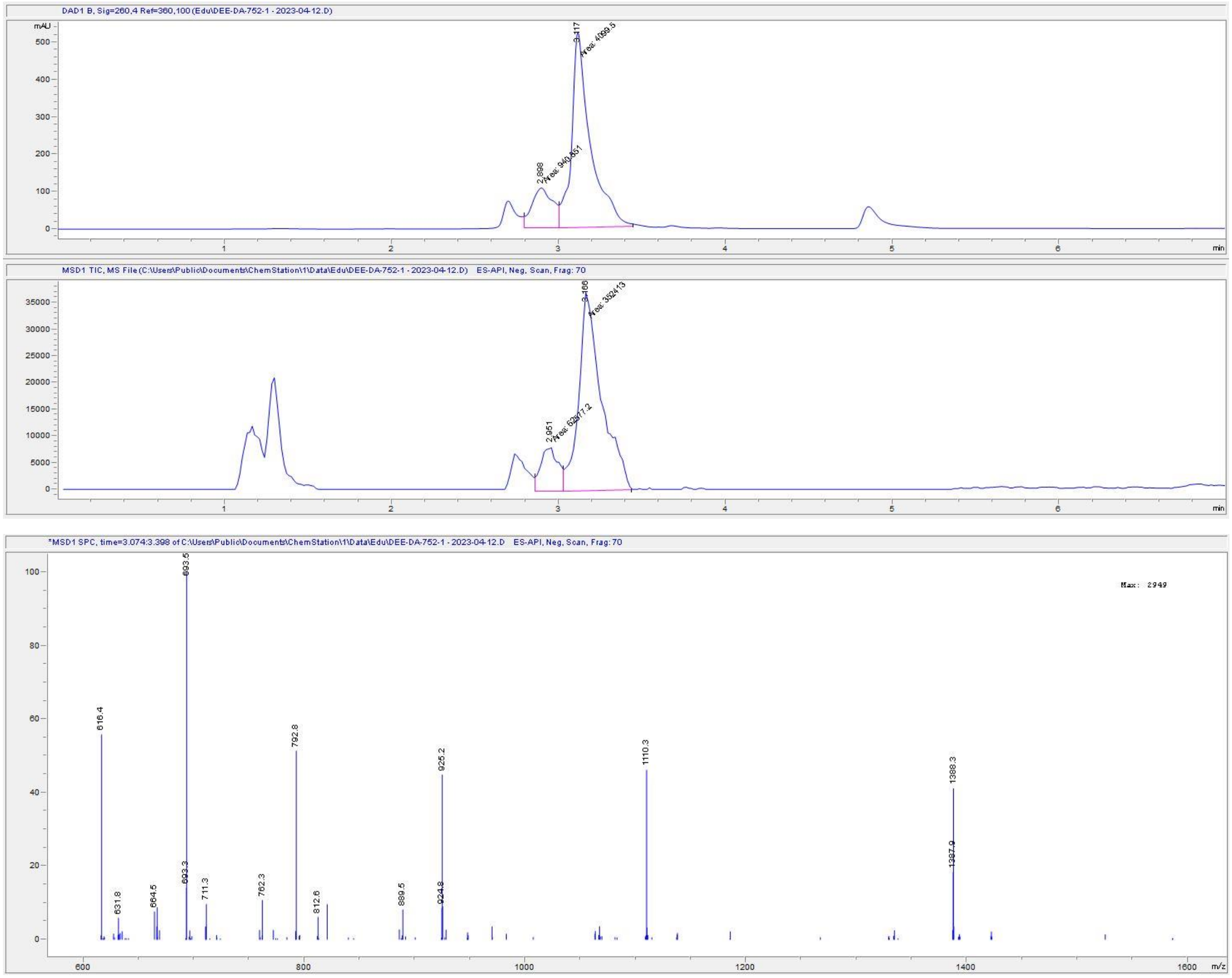

Figure S46. Analytical HPLC trace of **S49** with HPLC Method B. (Up) DAD chromatogram at 260 nm. (Middle) TIC chromatogram. (Below) Ionization of peak at 3.166 min containing reaction product.

Synthesis of DNA-conjugated selenonium salt S50

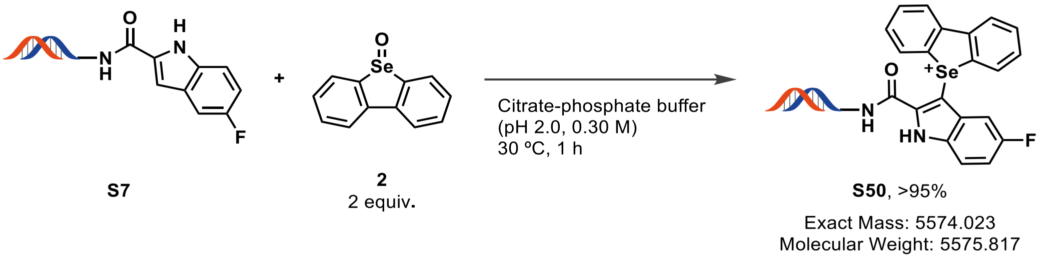

At 20–25 °C, 1.0 µL of **S7** (2.0 mM, 2.0 nmol, 1.0 equiv.) in water was added to a 1.5 mL Eppendorf tube. Next, 1.0 µL of Citrate-phosphate buffer (pH 2.0, c = 1.0 M) was added. Then, 1.0 µL of a selenoxide **2** stock solution (4.0 mM, 4.0 nmol, 2.0 equiv.) in water was added. The mixture was vortexed for 5 seconds, transferred into a Thermocycler pre-heated at 30 °C, and incubated at 30 °C for 1 hour at 600 rpm. After 1 hour, reaction was quenched by addition of 10 µL of borate buffer (pH 9.4, c = 500 mM), and an aliquot of 2 µL of the reaction mixture was diluted to 40 µL with water for LC–MS analysis.

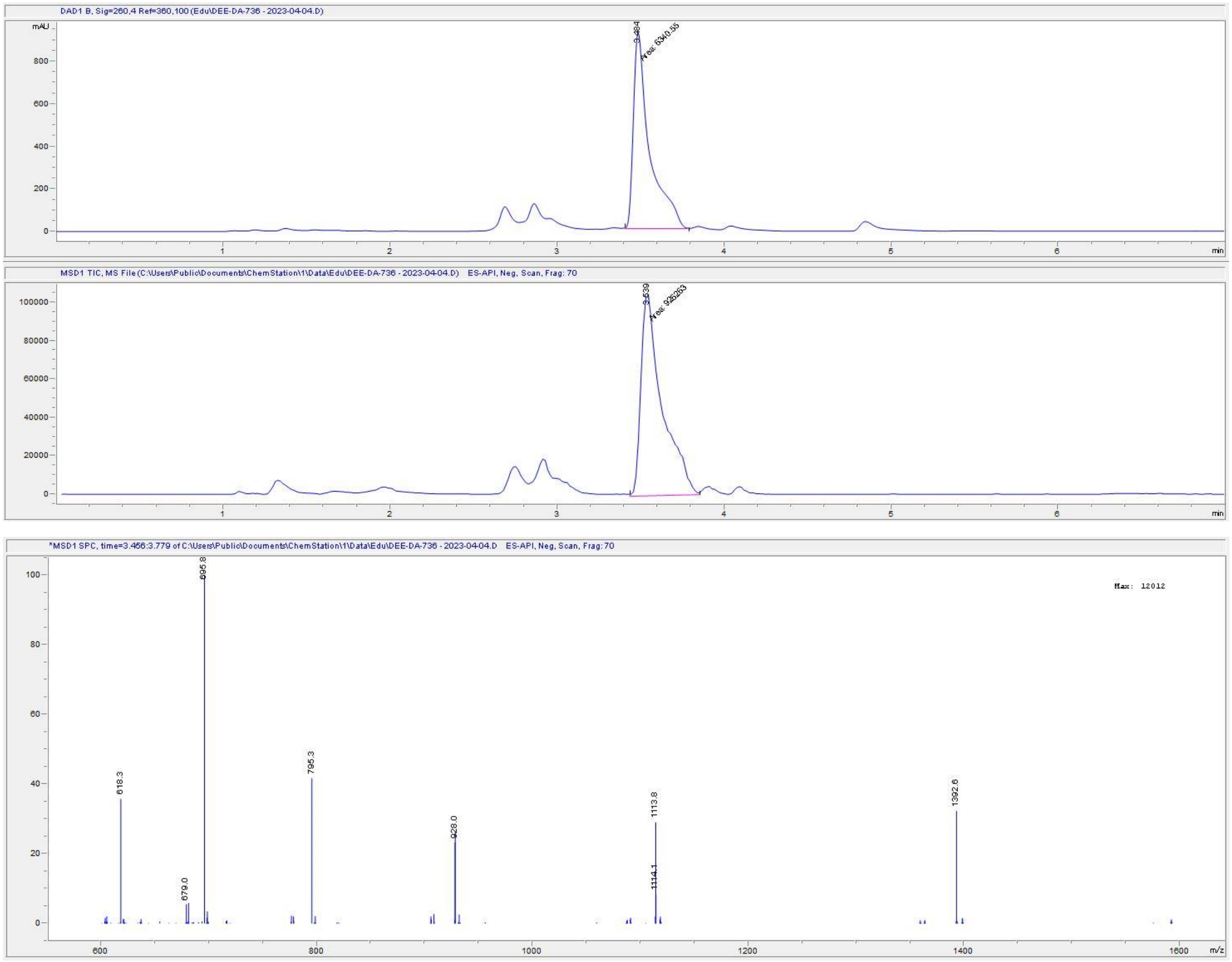

Figure S47. Analytical HPLC trace of **S50** with HPLC Method B. (Up) DAD chromatogram at 260 nm. (Middle) TIC chromatogram. (Below) Ionization of peak at 3.539 min containing reaction product.

Synthesis of DNA-conjugated selenonium salt S51

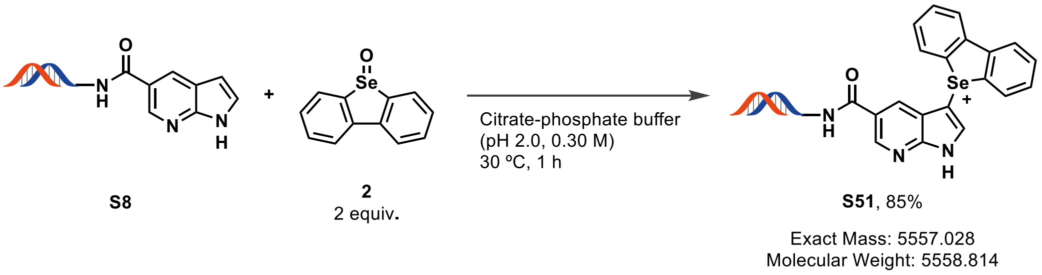

At 20–25 °C, 1.0 µL of **S8** (2.0 mM, 2.0 nmol, 1.0 equiv.) in water was added to a 1.5 mL Eppendorf tube. Next, 1.0 µL of Citrate-phosphate buffer (pH 2.0, c = 1.0 M) was added. Then, 1.0 µL of a selenoxide **2** stock solution (4.0 mM, 4.0 nmol, 2.0 equiv.) in water was added. The mixture was vortexed for 5 seconds, transferred into a Thermocycler pre-heated at 30 °C, and incubated at 30 °C for 1 hour at 600 rpm. After 1 hour, reaction was quenched by addition of 10 µL of borate buffer (pH 9.4, c = 500 mM), and an aliquot of 2 µL of the reaction mixture was diluted to 40 µL with water for LC–MS analysis.

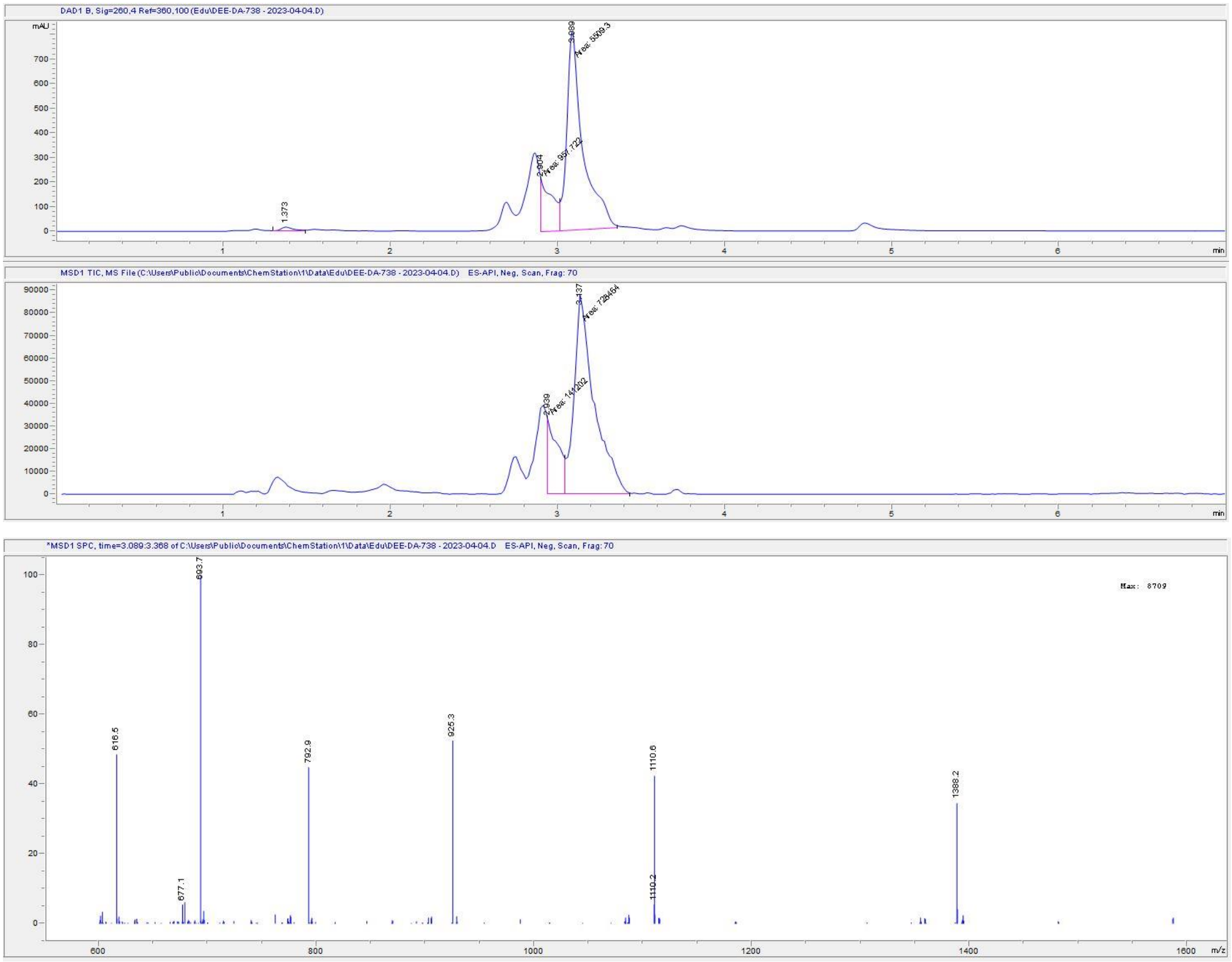

Figure S48. Analytical HPLC trace of **S51** with HPLC Method B. (Up) DAD chromatogram at 260 nm. (Middle) TIC chromatogram. (Below) Ionization of peak at 3.137 min containing reaction product.

Synthesis of DNA-conjugated selenonium salt S52

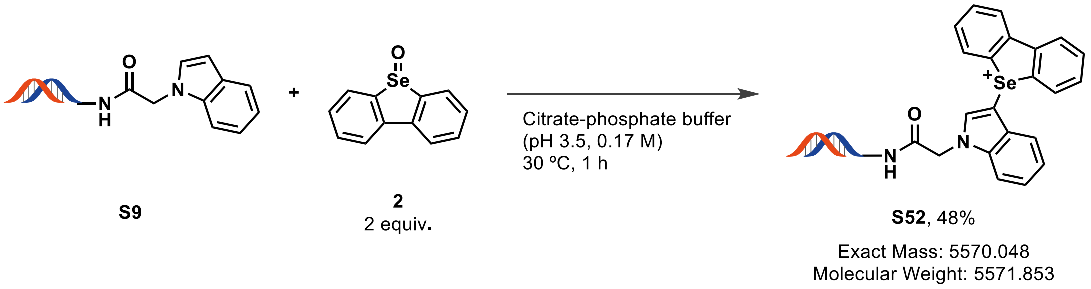

At 20–25 °C, 1.0 µL of **S9** (2.0 mM, 2.0 nmol, 1.0 equiv.) in water was added to a 1.5 mL Eppendorf tube. Next, 1.0 µL of Citrate-phosphate buffer (pH 3.5, c = 500 mM) was added. Then, 1.0 µL of a selenoxide **2** stock solution (4.0 mM, 4.0 nmol, 2.0 equiv.) in water was added. The mixture was vortexed for 5 seconds, transferred into a Thermocycler pre-heated at 30 °C, and incubated at 30 °C for 1 hour at 600 rpm. After 1 hour, reaction was quenched by addition of 10 µL of borate buffer (pH 9.4, c = 500 mM), and an aliquot of 2 µL of the reaction mixture was diluted to 40 µL with water for LC–MS analysis.

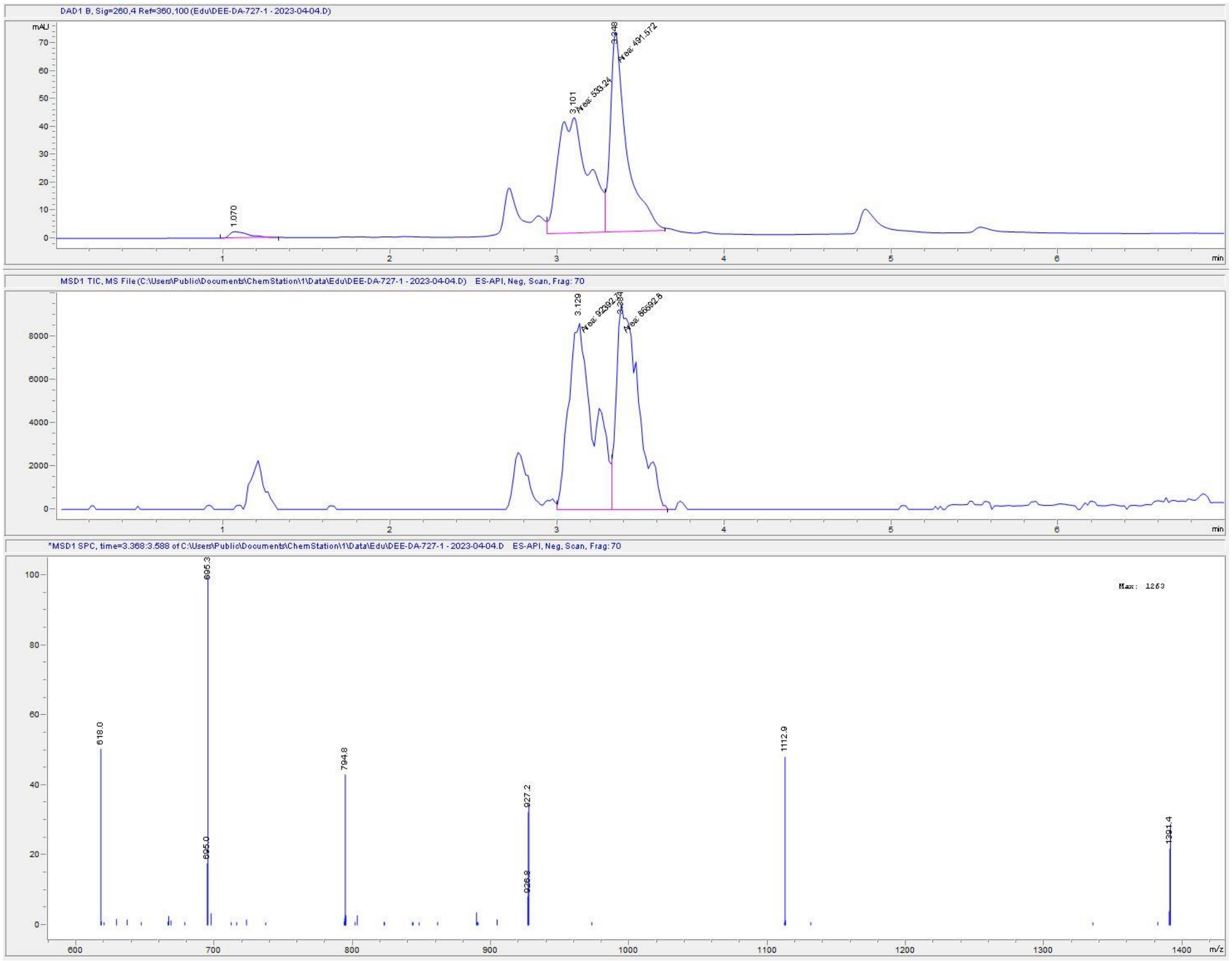

Figure S49. Analytical HPLC trace of **S52** with HPLC Method B. (Up) DAD chromatogram at 260 nm. (Middle) TIC chromatogram. (Below) Ionization of peak at 3.384 min containing reaction product.

Synthesis of DNA-conjugated selenonium salt S53

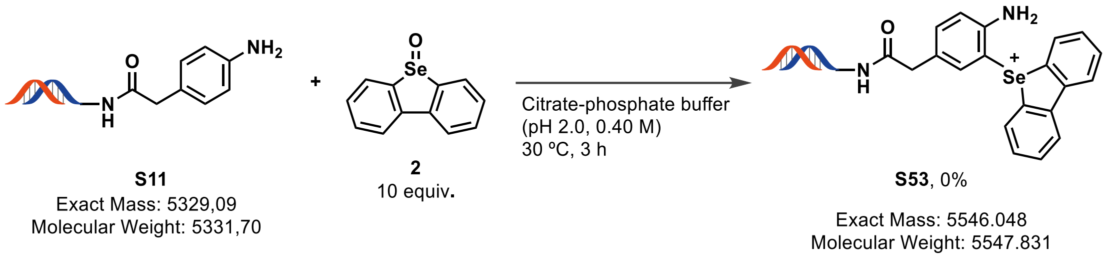

S11
Exact Mass: 5329,09
Molecular Weight: 5331,70

2
10 equiv**.**

S53, 0%

Exact Mass: 5546.048
Molecular Weight: 5547.831

At 20–25 °C, 1.0 µL of **S11** (2.0 mM, 2.0 nmol, 1.0 equiv.) in water was added to a 1.5 mL Eppendorf tube. Next, 4.0 µL of Citrate-phosphate buffer (pH 2.0, c = 1.0 M) was added. Then, 5.0 µL of a selenoxide **2** stock solution (2.0 mM, 20 nmol, 10 equiv.) in water was added. The mixture was vortexed for 5 seconds, transferred into a Thermocycler pre-heated at 30 °C, and incubated at 30 °C for 3 hours at 600 rpm. After 3 hours, reaction was quenched by addition of 10 µL of borate buffer (pH 9.4, c = 500 mM), and an aliquot of 2 µL of the reaction mixture was diluted to 40 µL with water for LC–MS analysis.

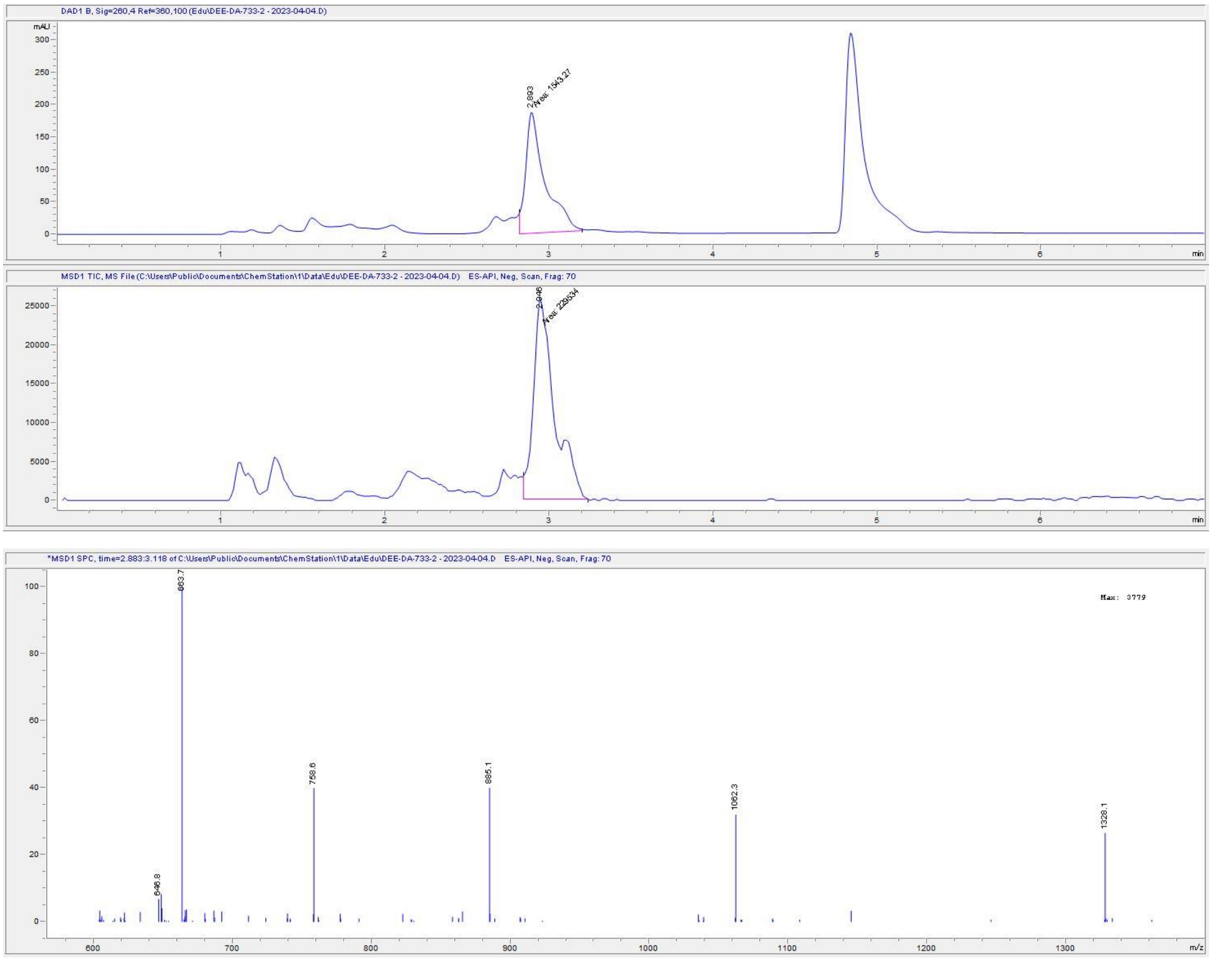

Figure S50. Analytical HPLC trace of **S53** with HPLC Method B. (Up) DAD chromatogram at 260 nm. (Middle) TIC chromatogram. (Below) Ionization of peak at 2.946 min containing starting material **S11**.

Synthesis of DNA-conjugated selenonium salt S54

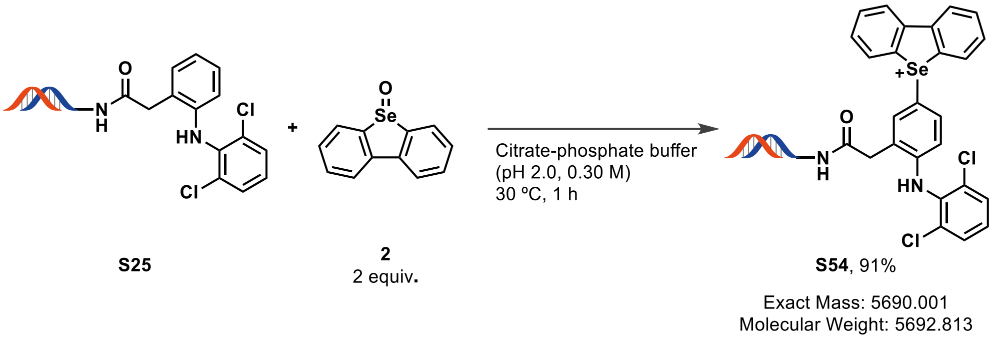

At 20–25 °C, 1.0 µL of **S25** (2.0 mM, 2.0 nmol, 1.0 equiv.) in water was added to a 1.5 mL Eppendorf tube. Next, 1.0 µL of Citrate-phosphate buffer (pH 2.0, c = 1.0 M) was added. Then, 1.0 µL of a selenoxide **2** stock solution (4.0 mM, 4.0 nmol, 2.0 equiv.) in water was added. The mixture was vortexed for 5 seconds, transferred into a Thermocycler pre-heated at 30 °C, and incubated at 30 °C for 1 hour at 600 rpm. After 1 hour, reaction was quenched by addition of 10 µL of borate buffer (pH 9.4, c = 500 mM), and an aliquot of 2 µL of the reaction mixture was diluted to 40 µL with water for LC–MS analysis.

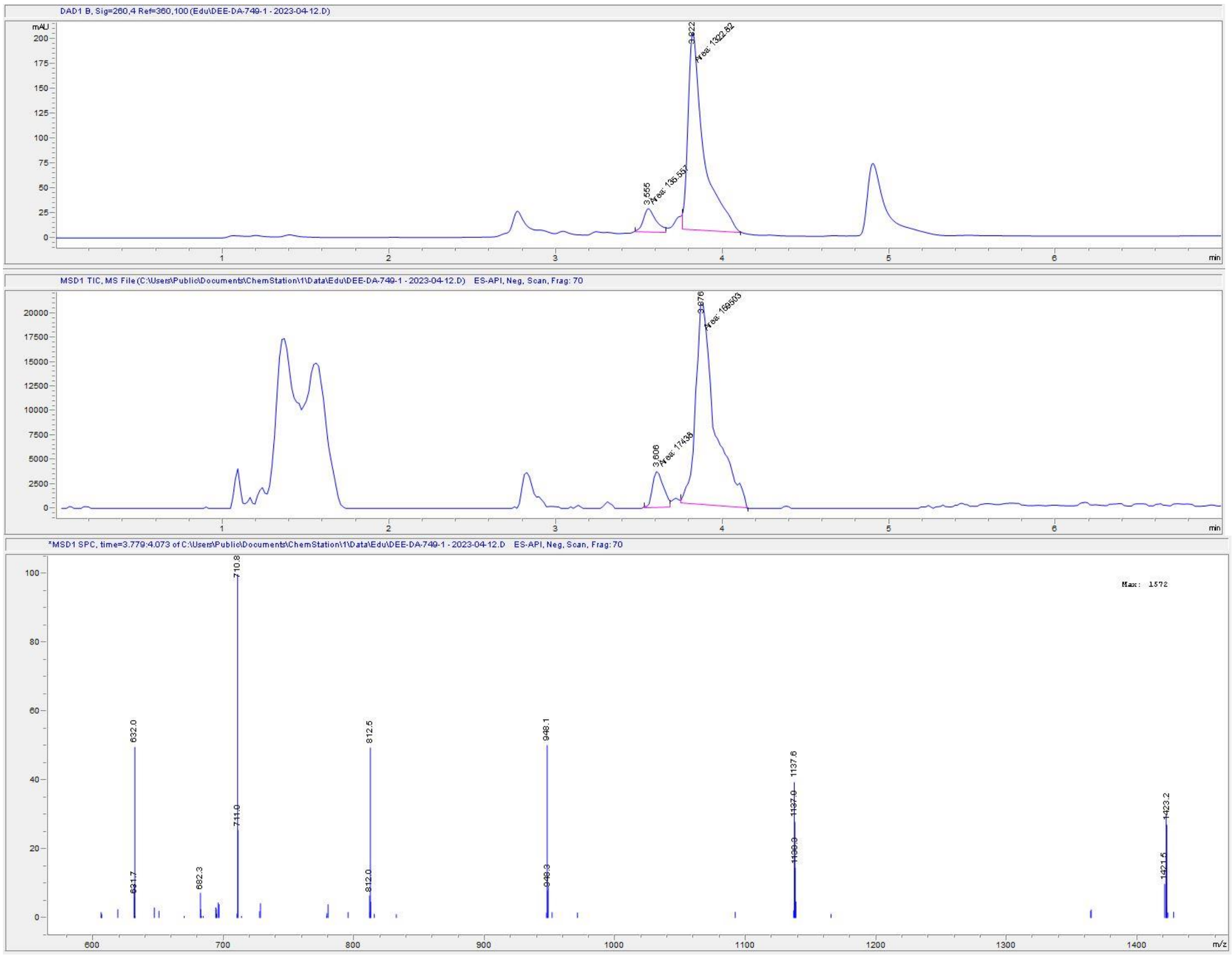

Figure S51. Analytical HPLC trace of **S54** with HPLC Method B. (Up) DAD chromatogram at 260 nm. (Middle) TIC chromatogram. (Below) Ionization of peak at 3.676 min containing reaction product.

Synthesis of DNA-conjugated selenonium salt S55

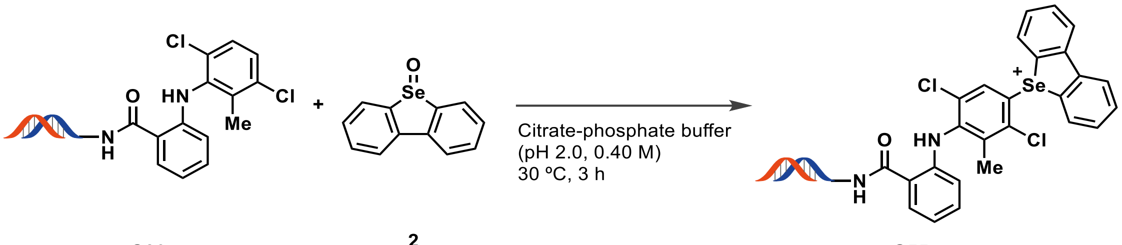

At 20–25 °C, 1.0 µL of **S20** (2.0 mM, 2.0 nmol, 1.0 equiv.) in water was added to a 1.5 mL Eppendorf tube. Next, 4.0 µL of Citrate-phosphate buffer (pH 2.0, c = 1.0 M) was added. Then, 5.0 µL of a selenoxide **2** stock solution (2.0 mM, 20 nmol, 10 equiv.) in water was added. The mixture was vortexed for 5 seconds, transferred into a Thermocycler pre-heated at 30 °C, and incubated at 30 °C for 3 hours at 600 rpm. After 3 hours, reaction was quenched by addition of 10 µL of borate buffer (pH 9.4, c = 500 mM), and an aliquot of 2 µL of the reaction mixture was diluted to 40 µL with water for LC–MS analysis.

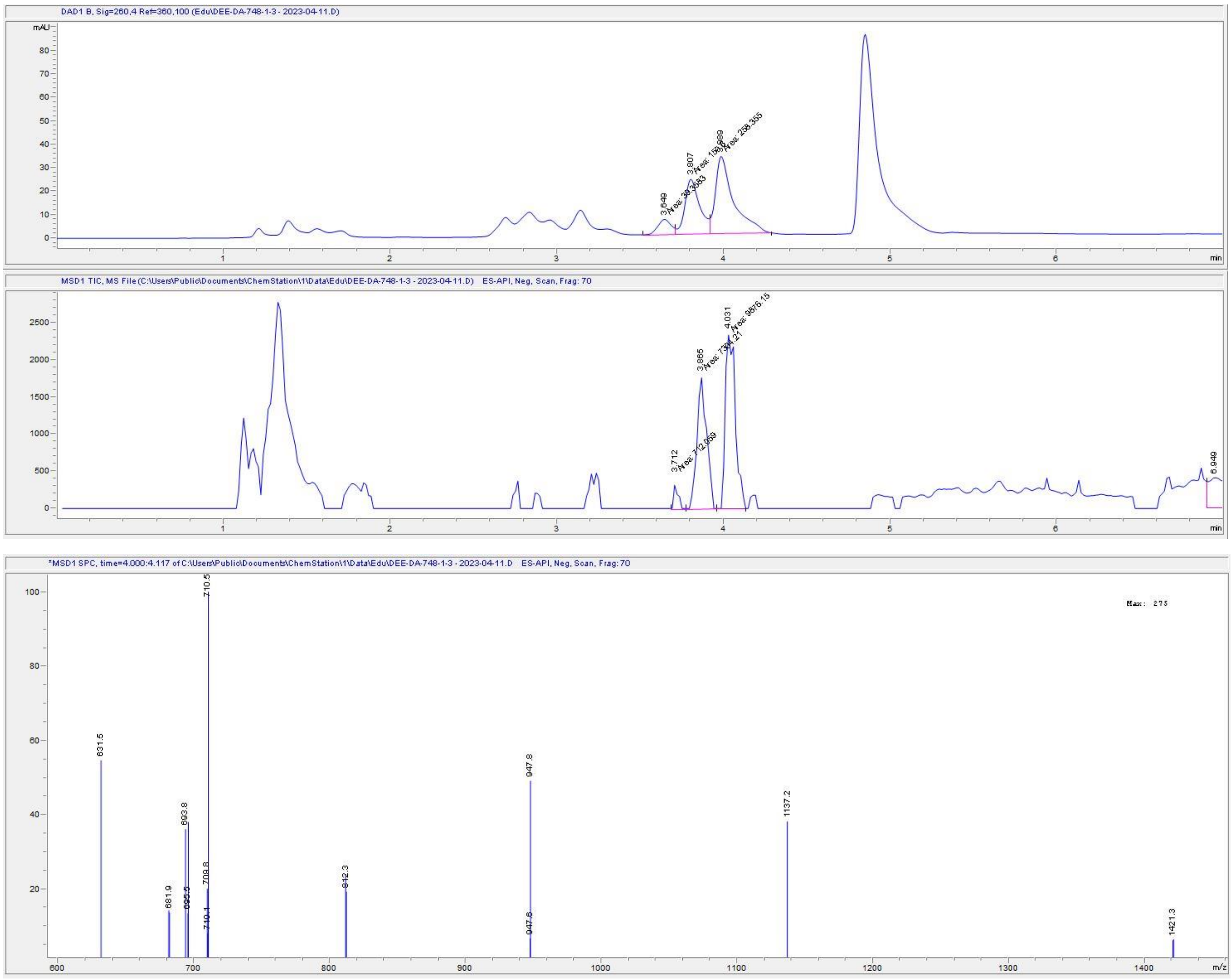

Figure S52. Analytical HPLC trace of **S55** with HPLC Method B. (Up) DAD chromatogram at 260 nm. (Middle) TIC chromatogram. (Below) Ionization of peak at 3.676 min containing reaction product.

Synthesis of DNA-conjugated selenonium salt S56

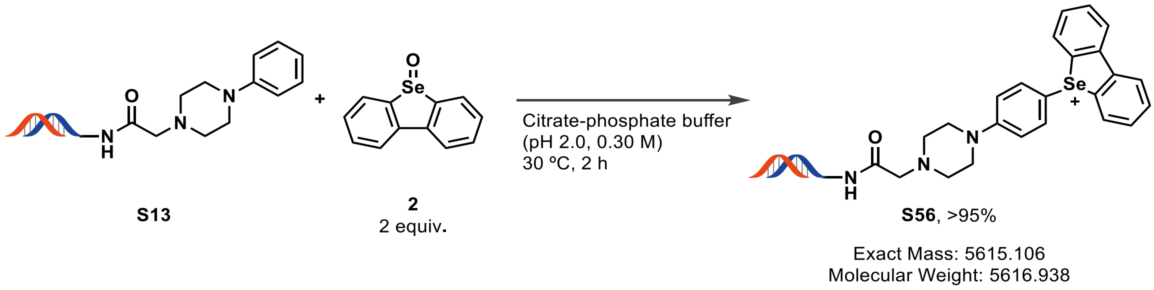

At 20–25 °C, 1.0 µL of **S13** (2.0 mM, 2.0 nmol, 1.0 equiv.) in water was added to a 1.5 mL Eppendorf tube. Next, 1.0 µL of Citrate-phosphate buffer (pH 2.0, c = 1.0 M) was added. Then, 1.0 µL of a selenoxide **2** stock solution (4.0 mM, 4.0 nmol, 2.0 equiv.) (4.0 mM, 4.0 nmol, 2.0 equiv.) in water was added. The mixture was vortexed for 5 seconds, transferred into a Thermocycler pre-heated at 30 °C, and incubated at 30 °C for 2 hours at 600 rpm. After 3 hours, reaction was quenched by addition of 10 µL of borate buffer (pH 9.4, c = 500 mM), and an aliquot of 2 µL of the reaction mixture was diluted to 40 µL with water for LC–MS analysis.

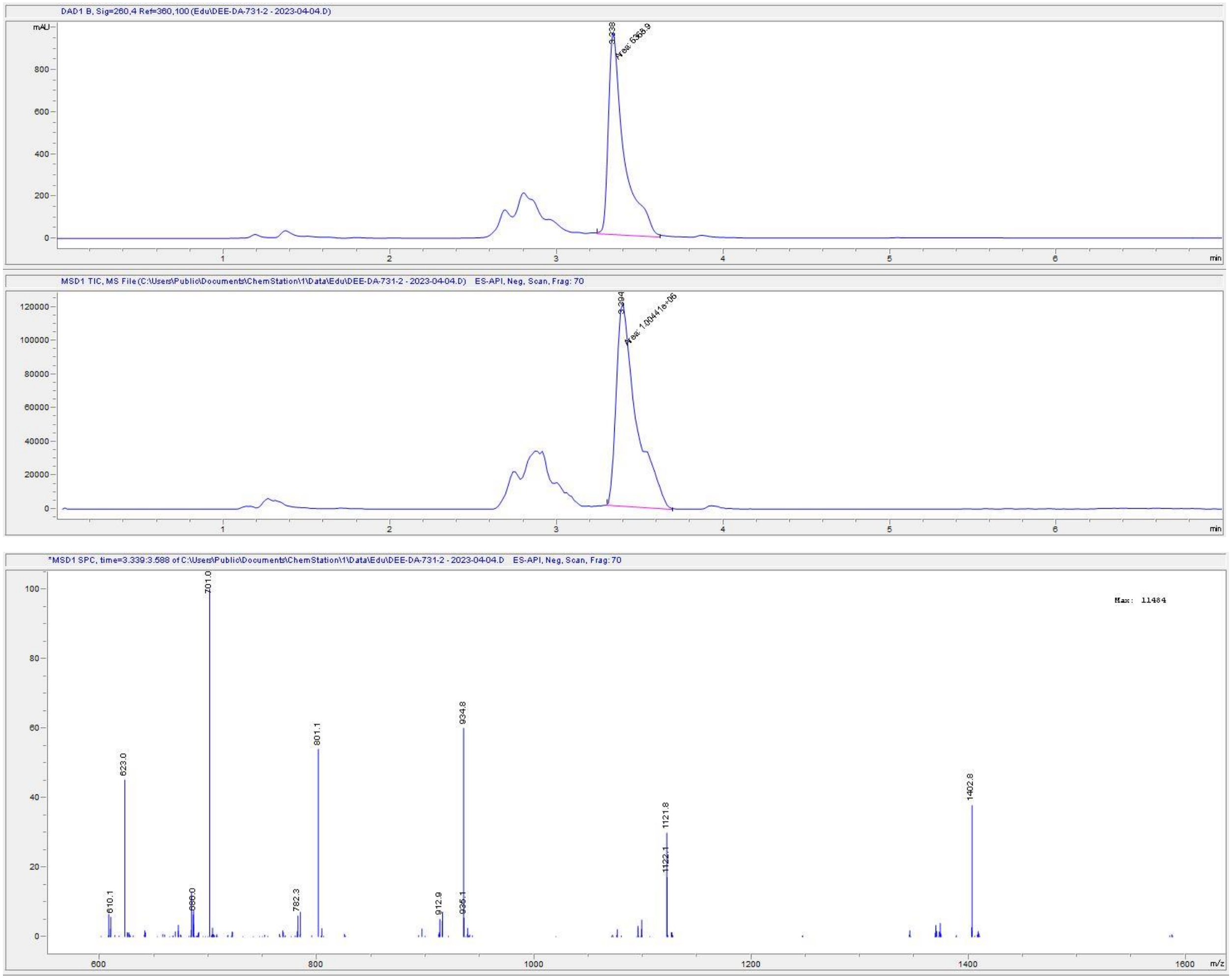

Figure S53. Analytical HPLC trace of **S56** with HPLC Method B. (Up) DAD chromatogram at 260 nm. (Middle) TIC chromatogram. (Below) Ionization of peak at 3.394 min containing reaction product.

Synthesis of DNA-conjugated selenonium salt S57

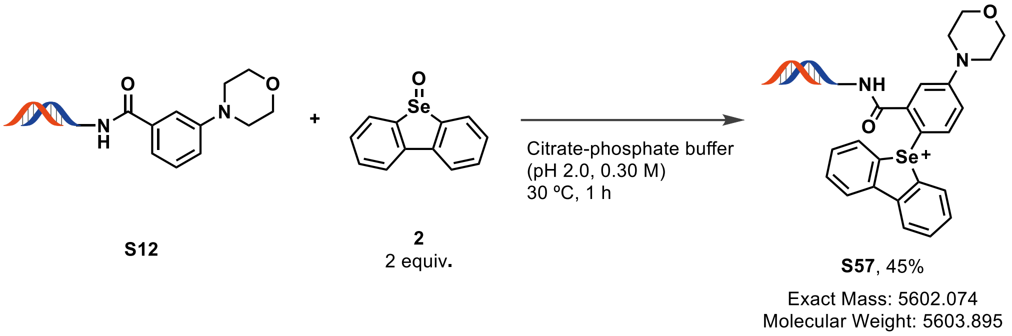

At 20–25 °C, 1.0 µL of **S12** (2.0 mM, 2.0 nmol, 1.0 equiv.) in water was added to a 1.5 mL Eppendorf tube. Next, 1.0 µL of Citrate-phosphate buffer (pH 3.0, c = 500 mM) was added. Then, 1.0 µL of a selenoxide **2** stock solution (4.0 mM, 4.0 nmol, 2.0 equiv.) in water was added. The mixture was vortexed for 5 seconds, transferred into a Thermocycler pre-heated at 30 °C, and incubated at 30 °C for 1 hour at 600 rpm. After 1 hour, reaction was quenched by addition of 10 µL of borate buffer (pH 9.4, c = 500 mM), and an aliquot of 2 µL of the reaction mixture was diluted to 40 µL with water for LC–MS analysis.

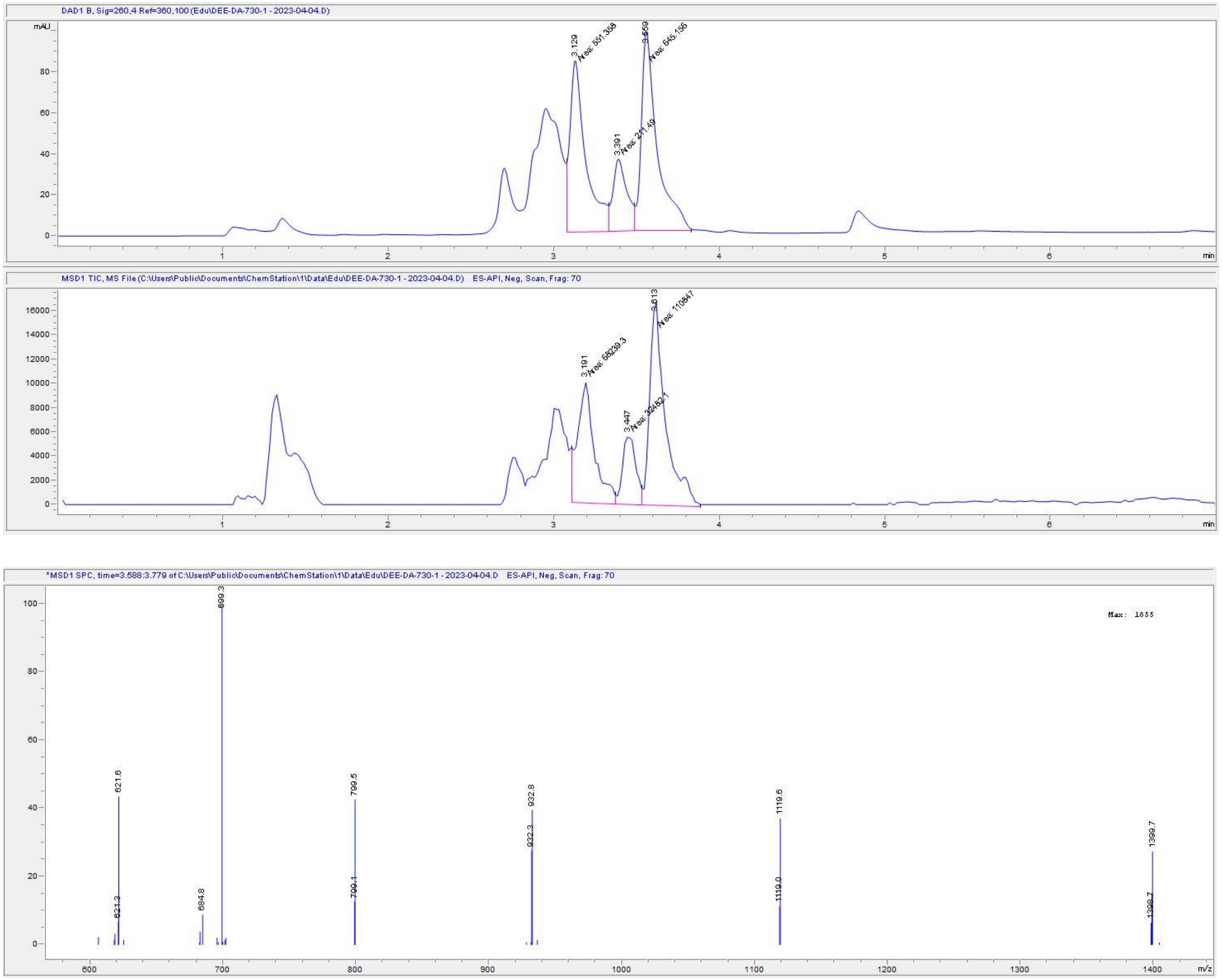

Figure S54. Analytical HPLC trace of **S57** with HPLC Method B. (Up) DAD chromatogram at 260 nm. (Middle) TIC chromatogram. (Below) Ionization of peak at 3.613 min containing reaction product.

Synthesis of DNA-conjugated selenonium salt S58

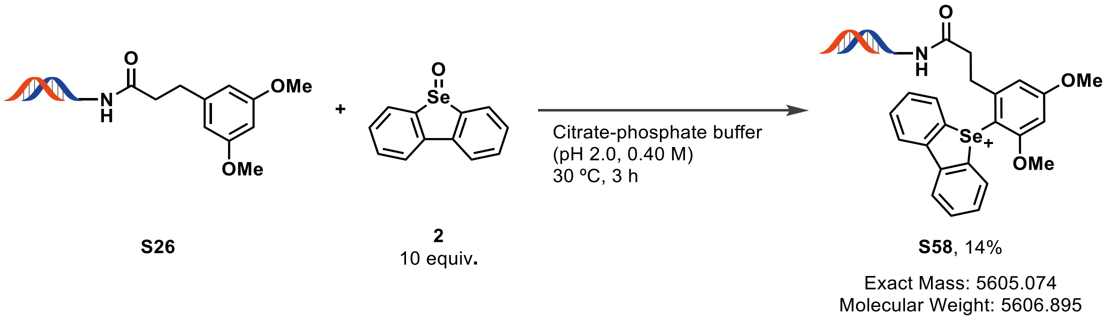

At 20–25 °C, 1.0 µL of **S26** (2.0 mM, 2.0 nmol, 1.0 equiv.) in water was added to a 1.5 mL Eppendorf tube. Next, 4.0 µL of Citrate-phosphate buffer (pH 3.0, c = 500 mM) was added. Then, 5.0 µL of a selenoxide **2** stock solution (2.0 mM, 20 nmol, 10 equiv.) in water was added. The mixture was vortexed for 5 seconds, transferred into a Thermocycler pre-heated at 30 °C, and incubated at 30 °C for 3 hours at 600 rpm. After 3 hours, reaction was quenched by addition of 10 µL of borate buffer (pH 9.4, c = 500 mM), and an aliquot of 2 µL of the reaction mixture was diluted to 40 µL with water for LC–MS analysis.

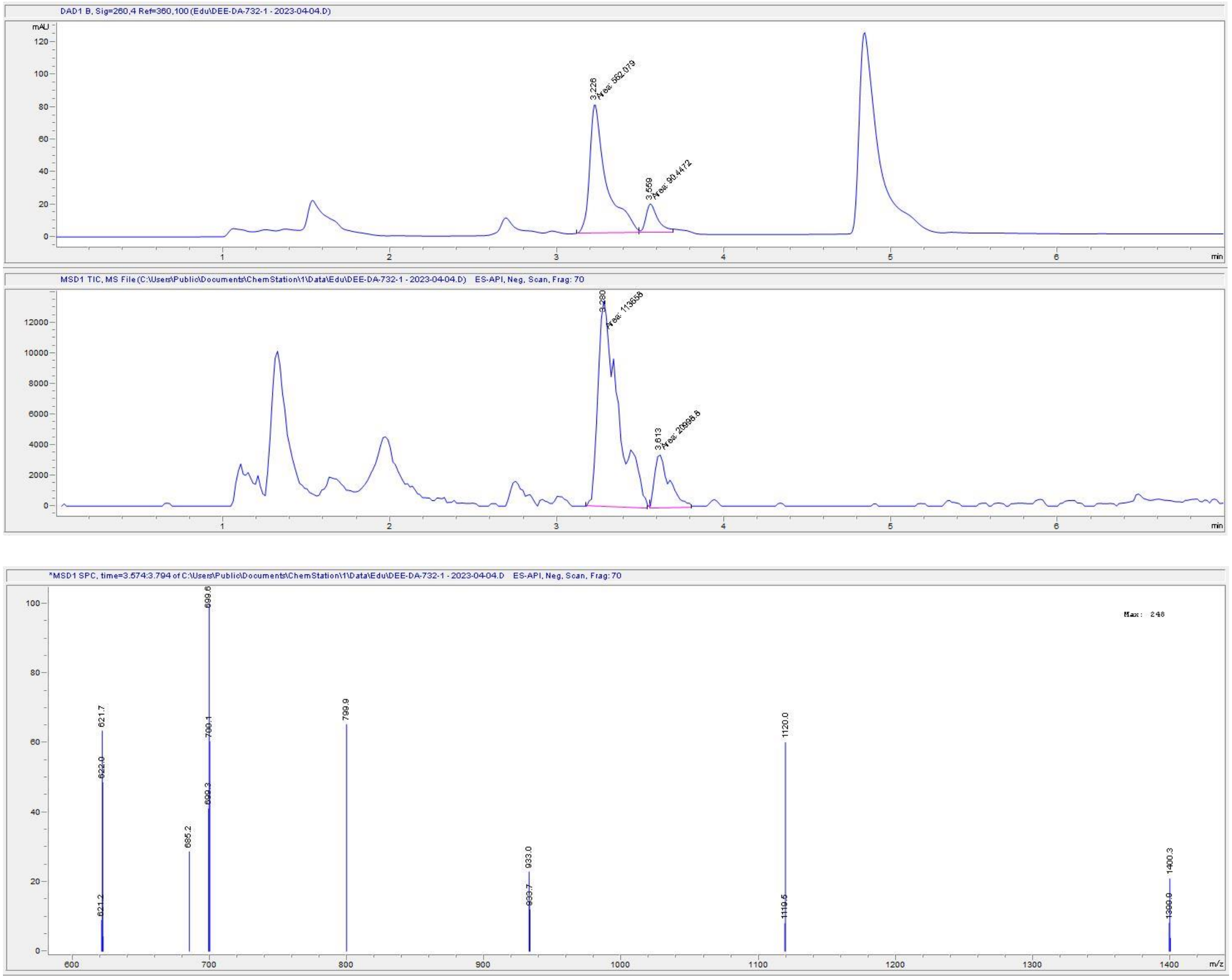

Figure S55. Analytical HPLC trace of **S58** with HPLC Method B. (Up) DAD chromatogram at 260 nm. (Middle) TIC chromatogram. (Below) Ionization of peak at 3.613 min containing reaction product.

DNA-conjugated C−H functionalization with selenoxide reagent 3

Synthesis of DNA-conjugated selenonium salt 4

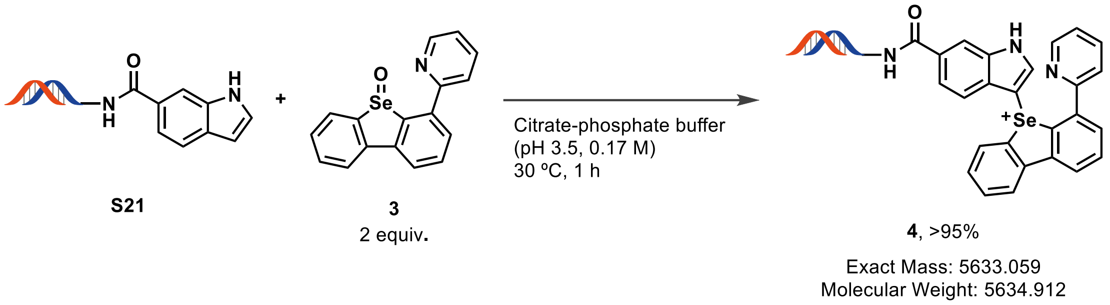

At 20–25 °C, 1.0 µL of **S21** (2.0 mM, 2.0 nmol, 1.0 equiv.) in water was added to a 1.5 mL Eppendorf tube. Next, 1.0 µL of Citrate-phosphate buffer (pH 3.5, c = 500 mM) was added. Then, 1.0 µL of a selenoxide **3** stock solution (4.0 mM, 4.0 nmol, 2.0 equiv.) in water was added. The mixture was vortexed for 5 seconds, transferred into a Thermocycler pre-heated at 30 °C, and incubated at 30 °C for 1 hour at 600 rpm. After 1 hour, reaction was quenched by addition of 10 µL of borate buffer (pH 9.4, c = 500 mM), and an aliquot of 2 µL of the reaction mixture was diluted to 40 µL with water for LC–MS analysis.

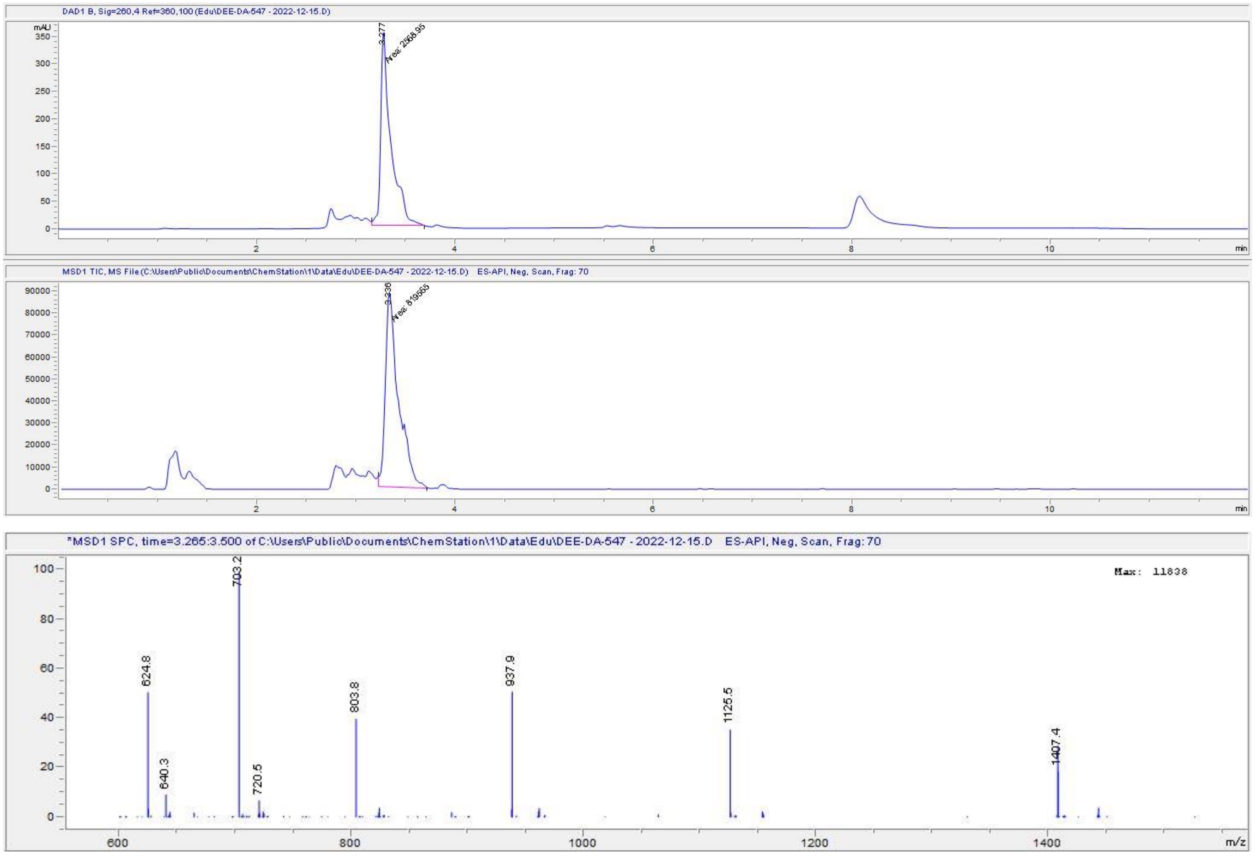

Figure S56. Analytical HPLC trace of **4** with HPLC Method A. (Up) DAD chromatogram at 260 nm. (Middle) TIC chromatogram. (Below) Ionization of peak at 3.336 min containing reaction product.

Synthesis of DNA-conjugated selenonium salt 5

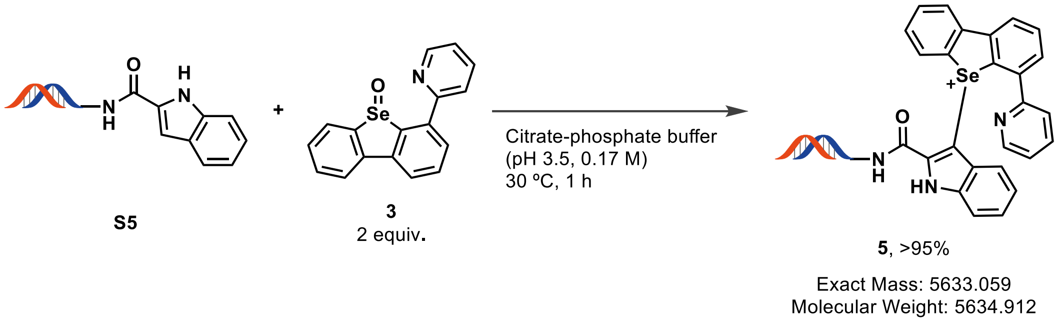

At 20–25 °C, 1.0 µL of **S5** (2.0 mM, 2.0 nmol, 1.0 equiv.) in water was added to a 1.5 mL Eppendorf tube. Next, 1.0 µL of Citrate-phosphate buffer (pH 3.5, c = 500 mM) was added. Then, 1.0 µL of a selenoxide **3** stock solution (4.0 mM, 4.0 nmol, 2.0 equiv.) in water was added. The mixture was vortexed for 5 seconds, transferred into a Thermocycler pre-heated at 30 °C, and incubated at 30 °C for 1 hour at 600 rpm. After 1 hour, reaction was quenched by addition of 10 µL of borate buffer (pH 9.4, c = 500 mM), and an aliquot of 2 µL of the reaction mixture was diluted to 40 µL with water for LC–MS analysis.

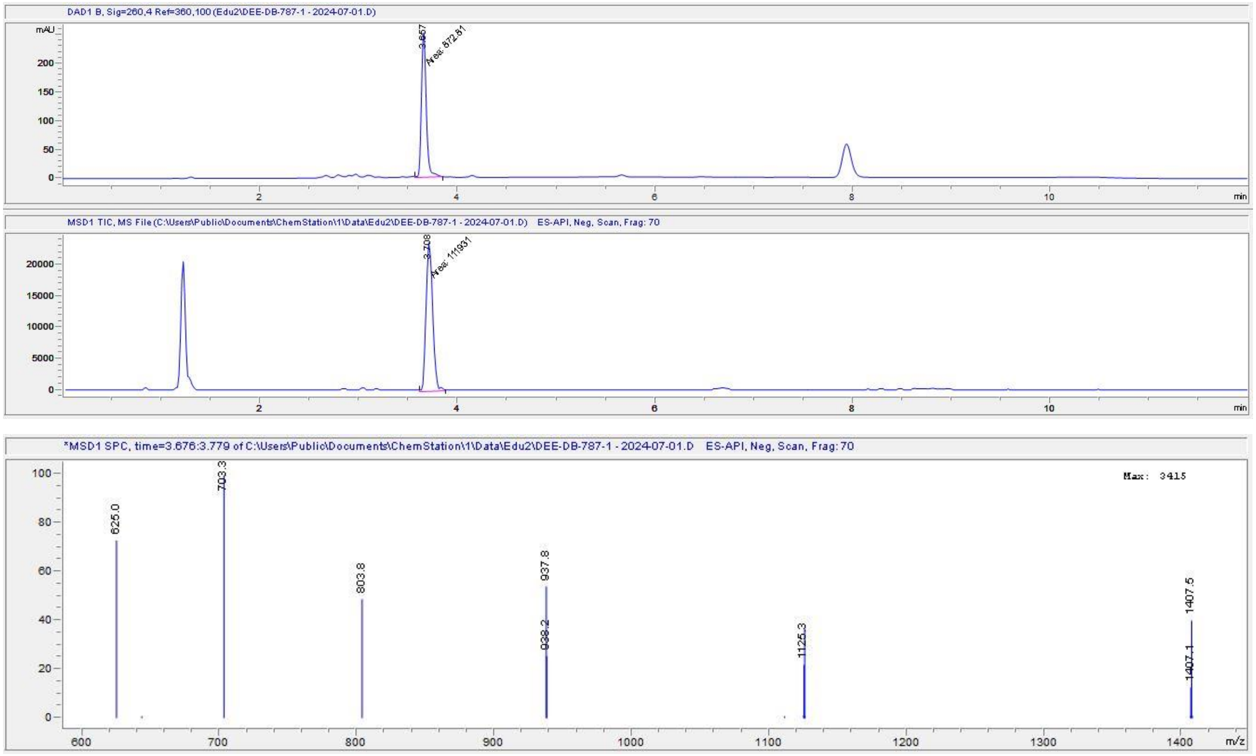

Figure S57. Analytical HPLC trace of **5** with HPLC Method A. (Up) DAD chromatogram at 260 nm. (Middle) TIC chromatogram. (Below) Ionization of peak 3.708 min containing reaction product.

Synthesis of DNA-conjugated selenonium salt 6

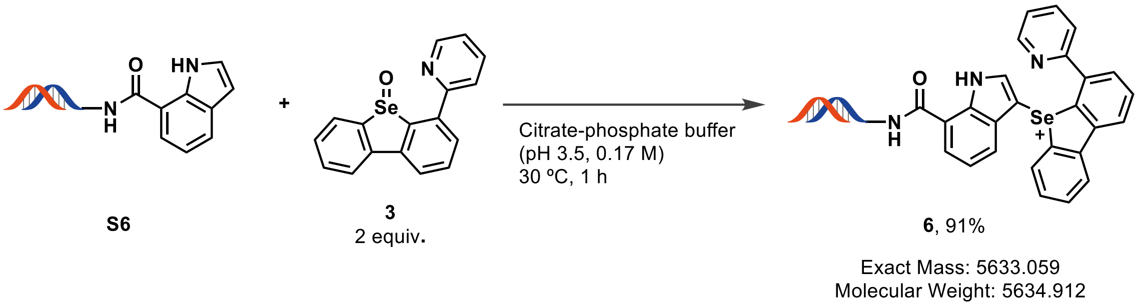

At 20–25 °C, 1.0 µL of **S6** (2.0 mM, 2.0 nmol, 1.0 equiv.) in water was added to a 1.5 mL Eppendorf tube. Next, 1.0 µL of Citrate-phosphate buffer (pH 3.5, c = 500 mM) was added. Then, 1.0 µL of a selenoxide **3** stock solution (4.0 mM, 4.0 nmol, 2.0 equiv.) in water was added. The mixture was vortexed for 5 seconds, transferred into a Thermocycler pre-heated at 30 °C, and incubated at 30 °C for 1 hour at 600 rpm. After 1 hour, reaction was quenched by addition of 10 µL of borate buffer (pH 9.4, c = 500 mM), and an aliquot of 2 µL of the reaction mixture was diluted to 40 µL with water for LC–MS analysis.

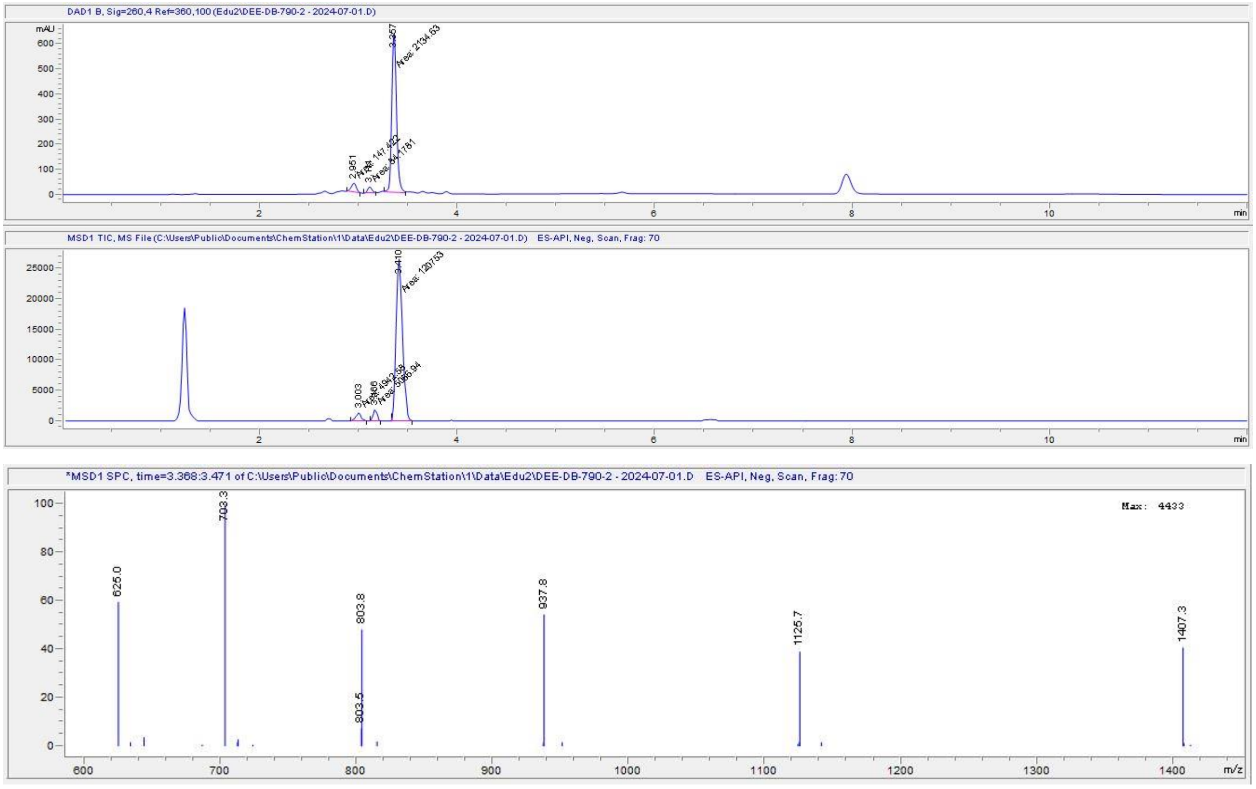

Figure S58. Analytical HPLC trace of **6** with HPLC Method A. (Up) DAD chromatogram at 260 nm. (Middle) TIC chromatogram. (Below) Ionization of peak at 3.410 min containing reaction product.

Synthesis of DNA-conjugated selenonium salt 7

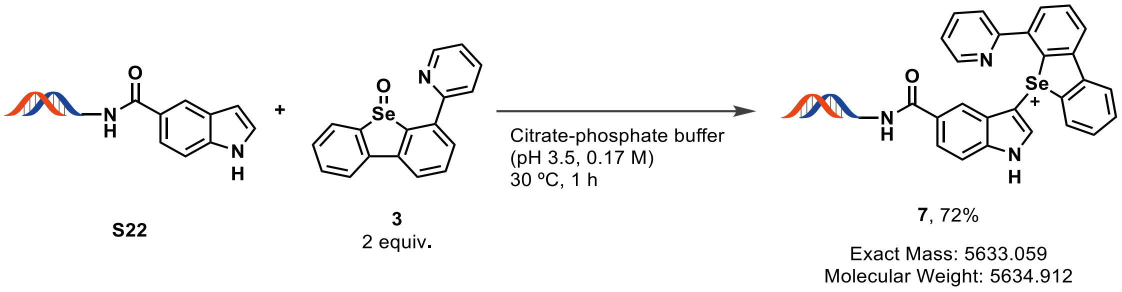

At 20–25 °C, 1.0 µL of **S22** (2.0 mM, 2.0 nmol, 1.0 equiv.) in water was added to a 1.5 mL Eppendorf tube. Next, 1.0 µL of Citrate-phosphate buffer (pH 3.5, c = 500 mM) was added. Then, 1.0 µL of a selenoxide **3** stock solution (4.0 mM, 4.0 nmol, 2.0 equiv.) in water was added. The mixture was vortexed for 5 seconds, transferred into a Thermocycler pre-heated at 30 °C, and incubated at 30 °C for 1 hour at 600 rpm. After 1 hour, reaction was quenched by addition of 10 µL of borate buffer (pH 9.4, c = 500 mM), and an aliquot of 2 µL of the reaction mixture was diluted to 40 µL with water for LC–MS analysis.

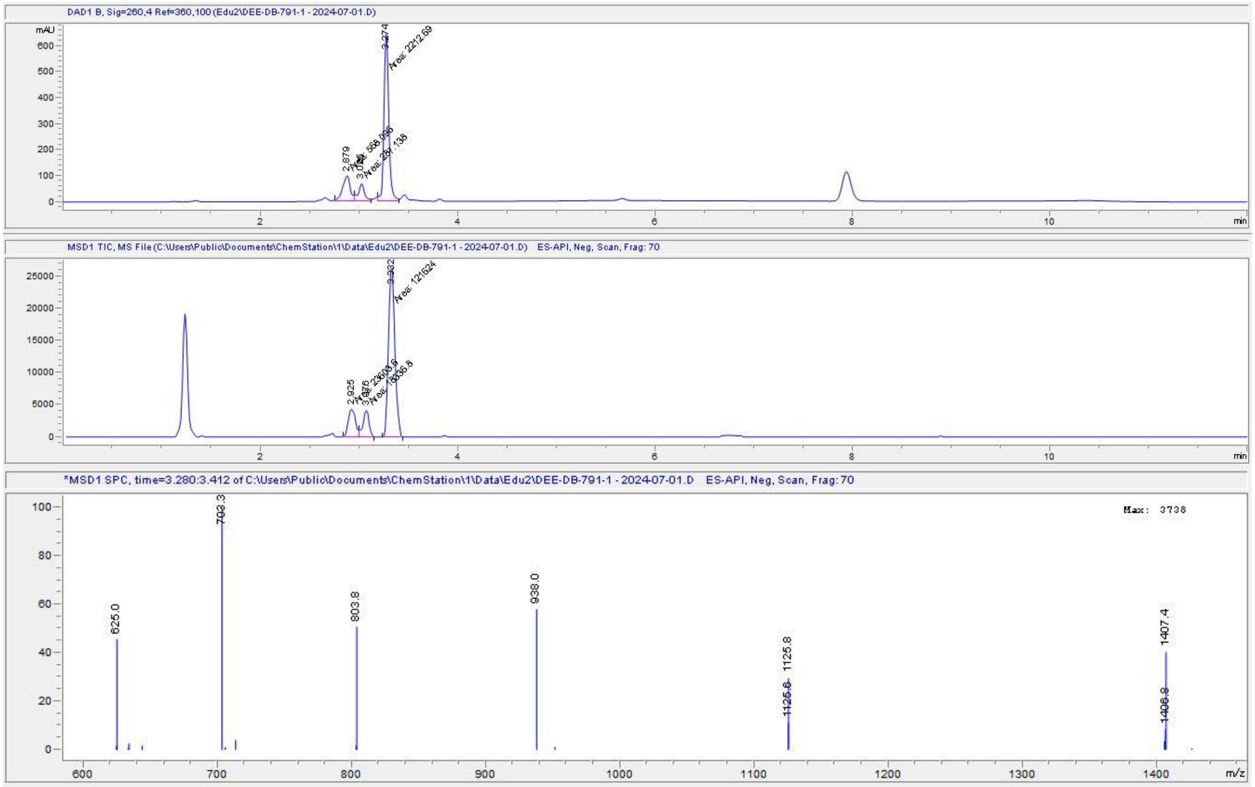

Figure S59. Analytical HPLC trace of **7** with HPLC Method A. (Up) DAD chromatogram at 260 nm. (Middle) TIC chromatogram. (Below) Ionization of peak at 3.332 min containing reaction product.

Synthesis of DNA-conjugated selenonium salt 8

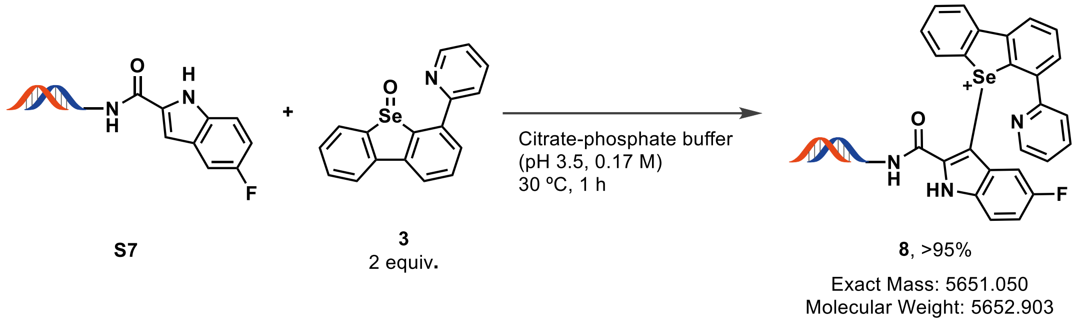

At 20–25 °C, 1.0 µL of **S7** (2.0 mM, 2.0 nmol, 1.0 equiv.) in water was added to a 1.5 mL Eppendorf tube. Next, 1.0 µL of Citrate-phosphate buffer (pH 3.5, c = 500 mM) was added. Then, 1.0 µL of a selenoxide **3** stock solution (4.0 mM, 4.0 nmol, 2.0 equiv.) in water was added. The mixture was vortexed for 5 seconds, transferred into a Thermocycler pre-heated at 30 °C, and incubated at 30 °C for 1 hour at 600 rpm. After 1 hour, reaction was quenched by addition of 10 µL of borate buffer (pH 9.4, c = 500 mM), and an aliquot of 2 µL of the reaction mixture was diluted to 40 µL with water for LC–MS analysis.

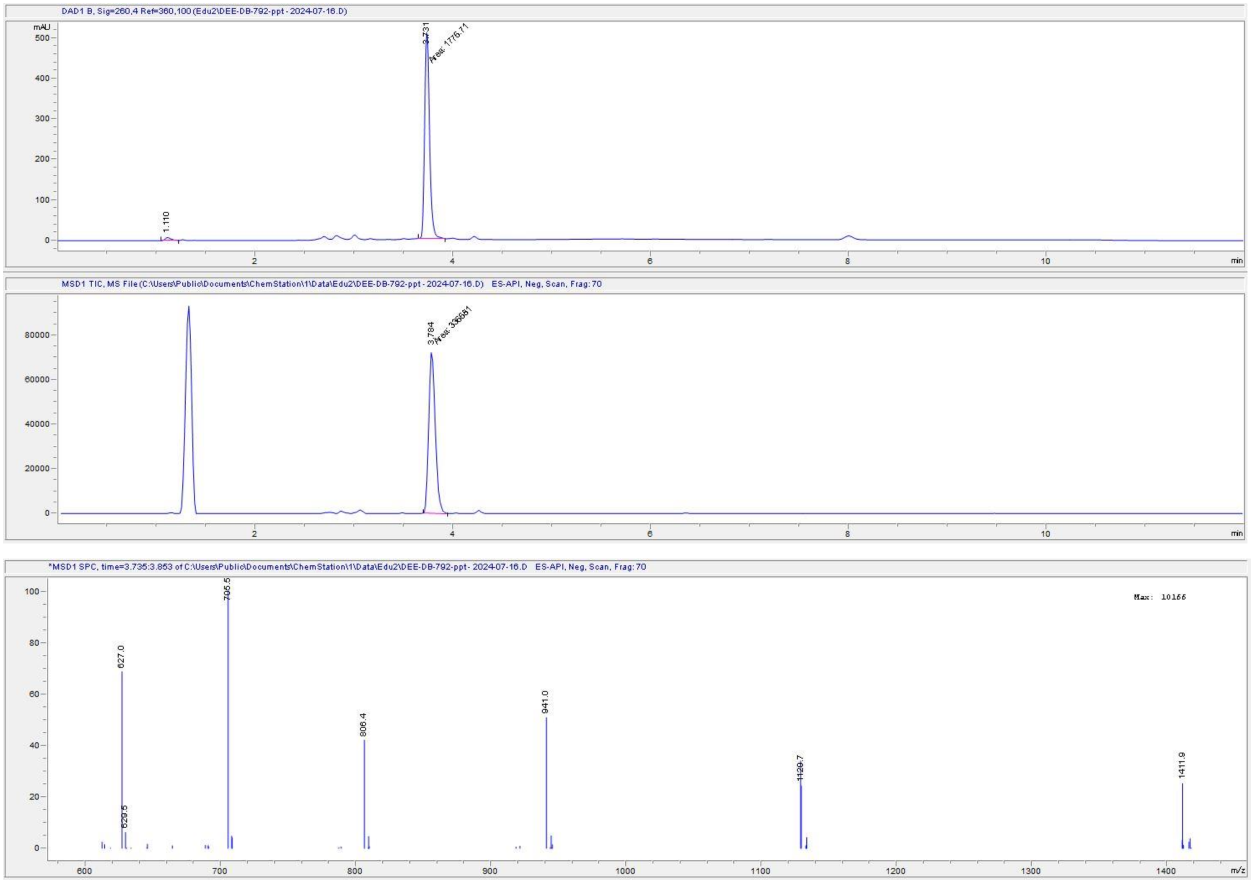

Figure S60. Analytical HPLC trace of **8** with HPLC Method A. (Up) DAD chromatogram at 260 nm. (Middle) TIC chromatogram. (Below) Ionization of peak at 3.784 min containing reaction product.

Synthesis of DNA-conjugated selenonium salt 9

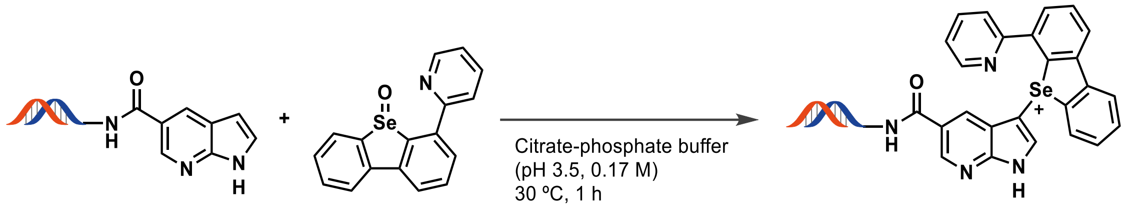

S8 **3** 2 equiv. **9**, >95%

Exact Mass: 5634.054
Molecular Weight: 5635.900

At 20–25 °C, 1.0 µL of **S8** (2.0 mM, 2.0 nmol, 1.0 equiv.) in water was added to a 1.5 mL Eppendorf tube. Next, 1.0 µL of Citrate-phosphate buffer (pH 3.5, c = 500 mM) was added. Then, 1.0 µL of a selenoxide **3** stock solution (4.0 mM, 4.0 nmol, 2.0 equiv.) in water was added. The mixture was vortexed for 5 seconds, transferred into a Thermocycler pre-heated at 30 °C, and incubated at 30 °C for 1 hour at 600 rpm. After 1 hour, reaction was quenched by addition of 10 µL of borate buffer (pH 9.4, c = 500 mM), and an aliquot of 2 µL of the reaction mixture was diluted to 40 µL with water for LC–MS analysis.

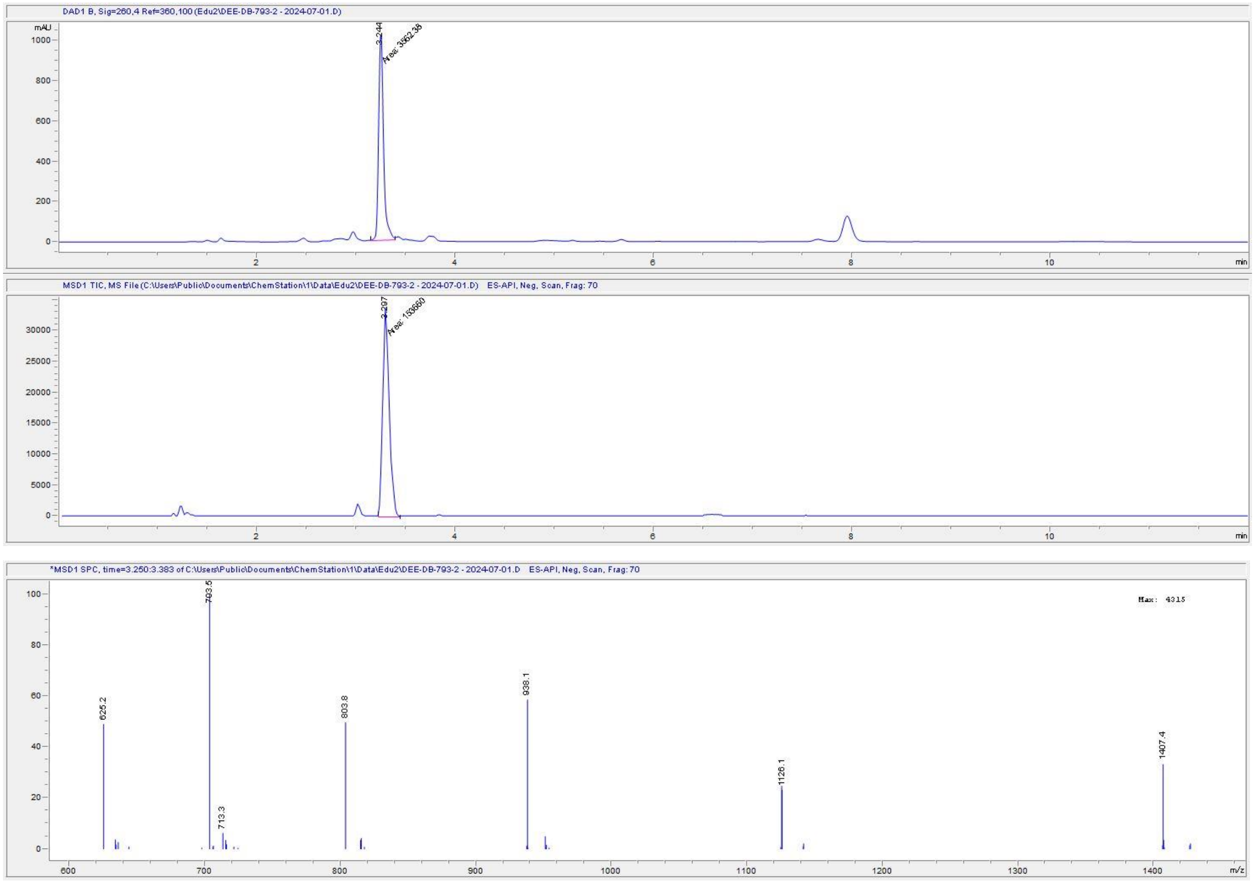

Figure S61. Analytical HPLC trace of **9** with HPLC Method A. (Up) DAD chromatogram at 260 nm. (Middle) TIC chromatogram. (Below) Ionization of peak at 3.297 min containing reaction product.

Synthesis of DNA-conjugated selenonium salt 10

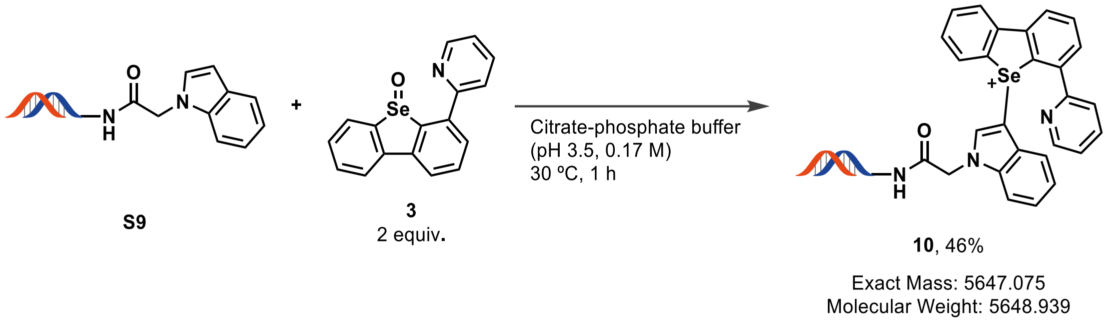

At 20–25 °C, 1.0 µL of **S9** (2.0 mM, 2.0 nmol, 1.0 equiv.) in water was added to a 1.5 mL Eppendorf tube. Next, 1.0 µL of Citrate-phosphate buffer (pH 3.5, c = 500 mM) was added. Then, 1.0 µL of a selenoxide **3** stock solution (4.0 mM, 4.0 nmol, 2.0 equiv.) in water was added. The mixture was vortexed for 5 seconds, transferred into a Thermocycler pre-heated at 30 °C, and incubated at 30 °C for 1 hour at 600 rpm. After 1 hour, reaction was quenched by addition of 10 µL of borate buffer (pH 9.4, c = 500 mM), and an aliquot of 2 µL of the reaction mixture was diluted to 40 µL with water for LC–MS analysis. *Conversion was calculated using the TIC chromatogram.*

Oxidation byproducts refer to unidentified byproducts with a MW of **S9**+16 and **S9**+32.

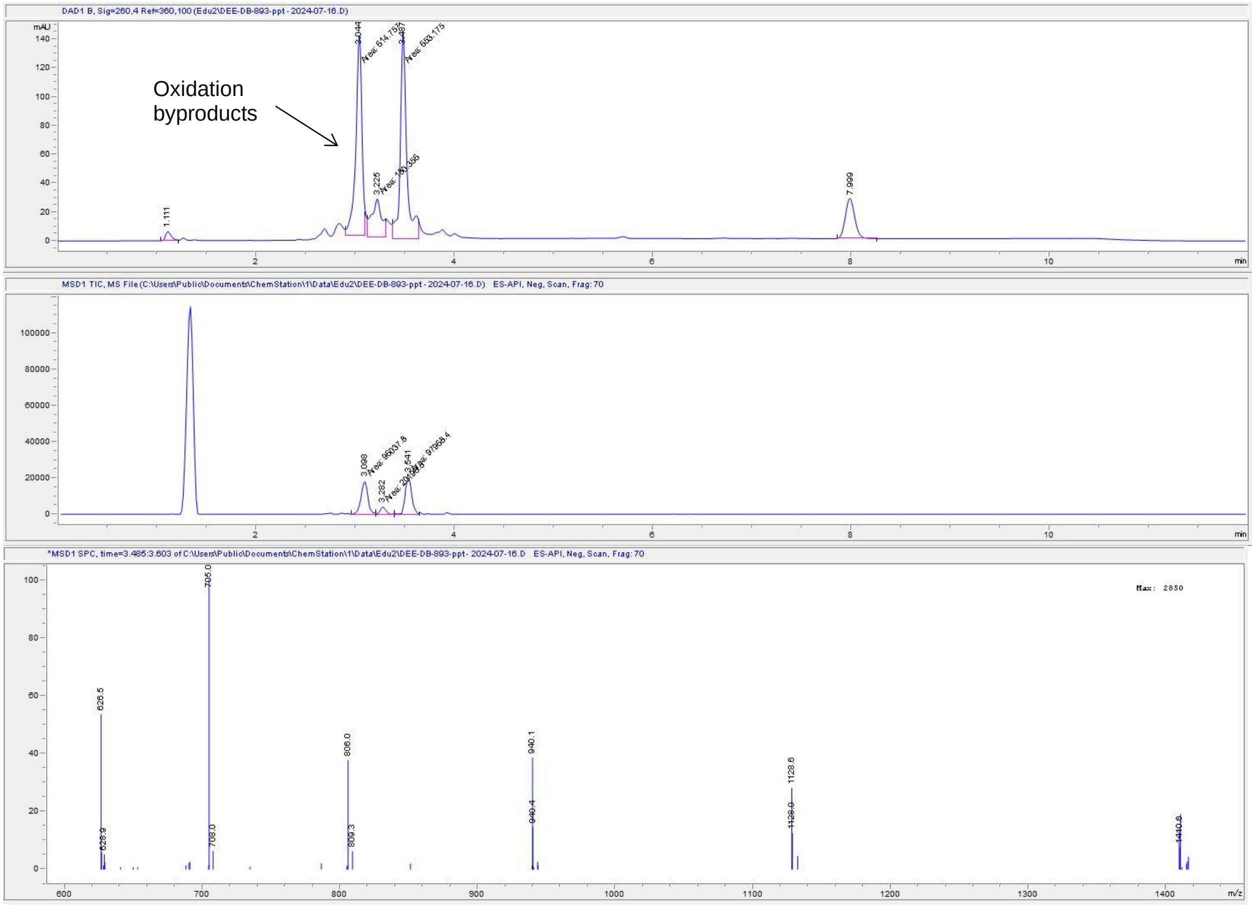

Figure S62. Analytical HPLC trace of **10** with HPLC Method A. (Up) DAD chromatogram at 260 nm. (Middle) TIC chromatogram. (Below) Ionization of peak at 3.541 min containing reaction product.

Synthesis of DNA-conjugated selenonium salt 11

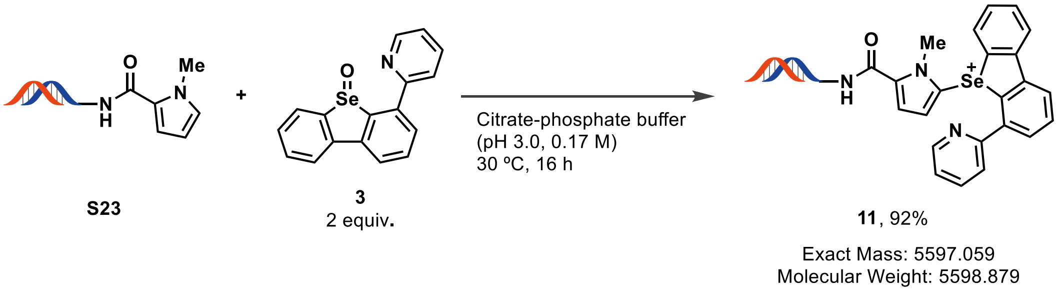

At 20–25 °C, 1.0 µL of **S23** (2.0 mM, 2.0 nmol, 1.0 equiv.) in water was added to a 1.5 mL Eppendorf tube. Next, 1.0 µL of Citrate-phosphate buffer (pH 3.0, c = 500 mM) was added. Then, 1.0 µL of a selenoxide **3** stock solution (4.0 mM, 4.0 nmol, 2.0 equiv.) in water was added. The mixture was vortexed for 5 seconds, transferred into a Thermocycler pre-heated at 30 °C, and incubated at 30 °C for 16 hours at 600 rpm. After 1 hour, reaction was quenched by addition of 10 µL of borate buffer (pH 9.4, c = 500 mM), and an aliquot of 2 µL of the reaction mixture was diluted to 40 µL with water for LC–MS analysis.

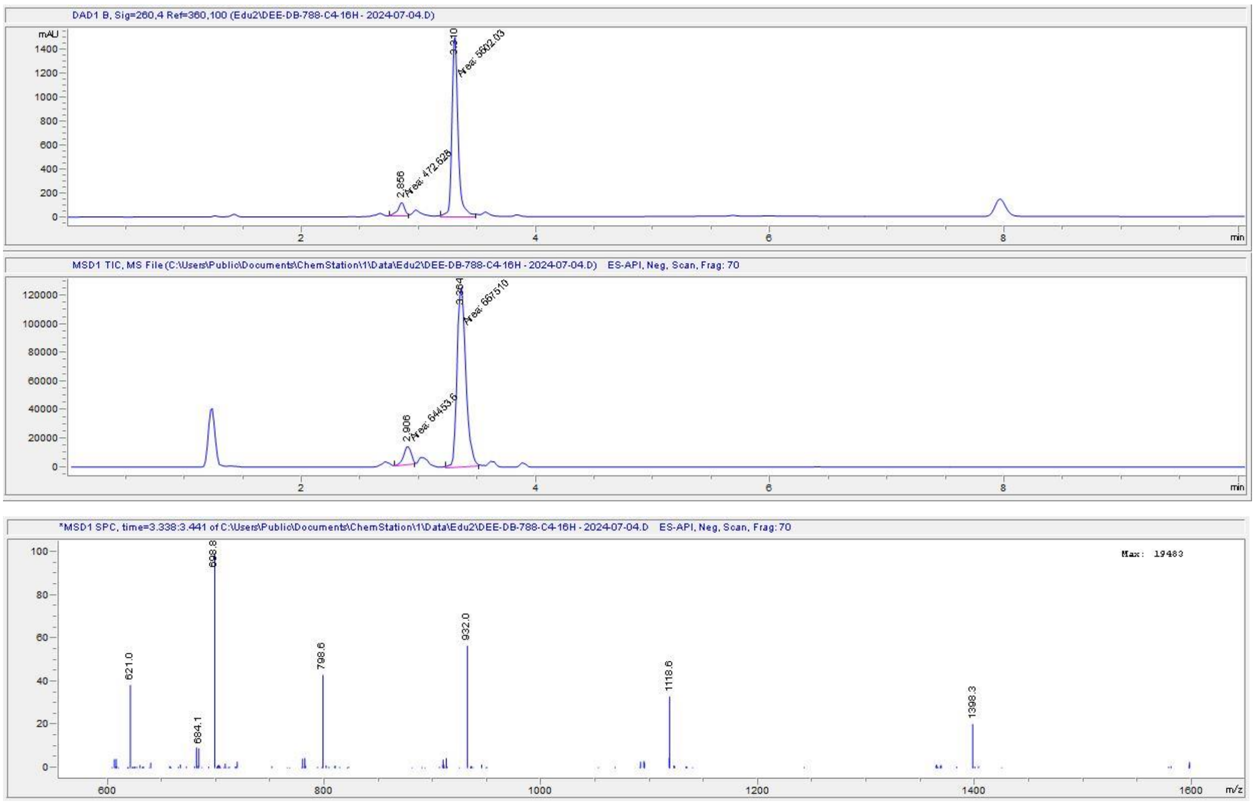

Figure S63. Analytical HPLC trace of **11** with HPLC Method A. (Up) DAD chromatogram at 260 nm. (Middle) TIC chromatogram. (Below) Ionization of peak at 3.364 min containing reaction product.

Synthesis of DNA-conjugated selenonium salt 12

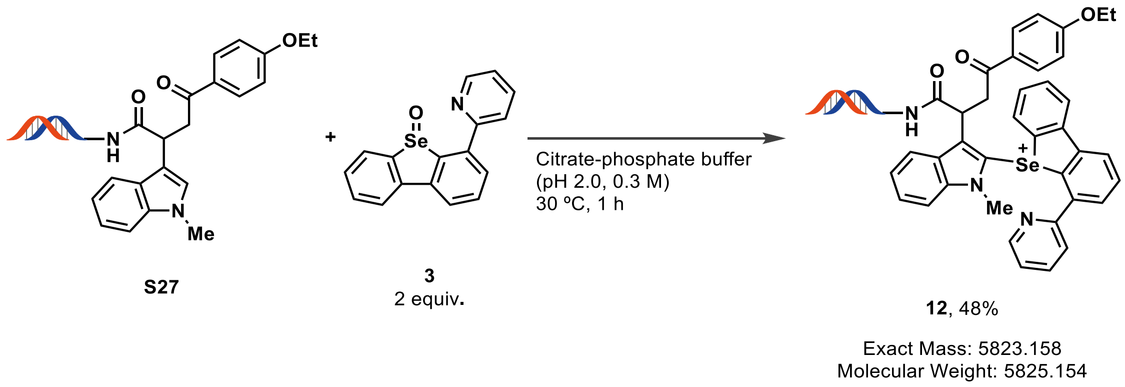

At 20–25 °C, 1.0 µL of **S27** (2.0 mM, 2.0 nmol, 1.0 equiv.) in water was added to a 1.5 mL Eppendorf tube. Next, 1.0 µL of Citrate-phosphate buffer (pH 2.0, c = 1.00 M) was added. Then, 1.0 µL of a selenoxide **3** stock solution (4.0 mM, 4.0 nmol, 2.0 equiv.) in water was added. The mixture was vortexed for 5 seconds, transferred into a Thermocycler pre-heated at 30 °C, and incubated at 30 °C for 1 hour at 600 rpm. After 1 hour, reaction was quenched by addition of 10 µL of borate buffer (pH 9.4, c = 500 mM), and an aliquot of 2 µL of the reaction mixture was diluted to 40 µL with water for LC–MS analysis.

Oxidation byproducts refer to unidentified byproducts with a MW of **S27**+16 and **S27**+32.

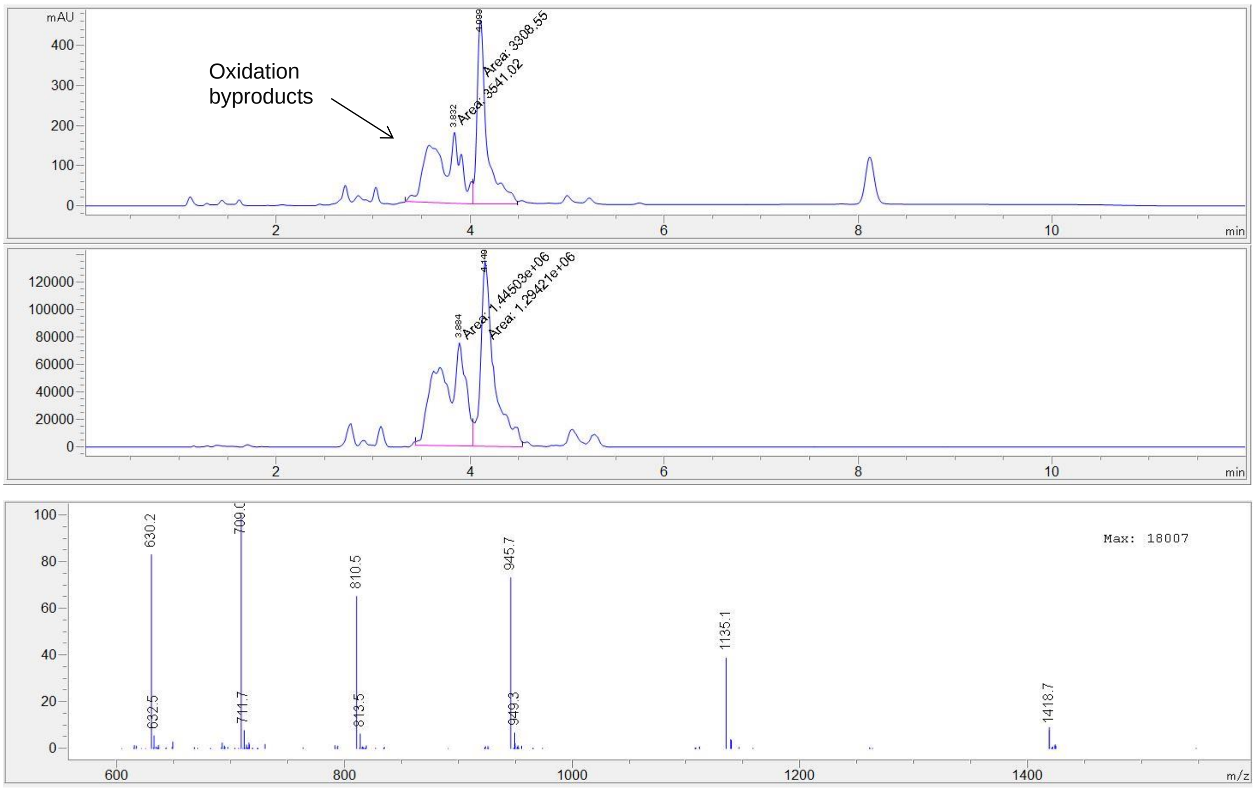

Figure S64. Analytical HPLC trace of **12** with HPLC Method A. (Up) DAD chromatogram at 260 nm. (Middle) TIC chromatogram. (Below) Ionization of peak at 4.149 min containing reaction product.

Synthesis of DNA-conjugated selenonium salt 13

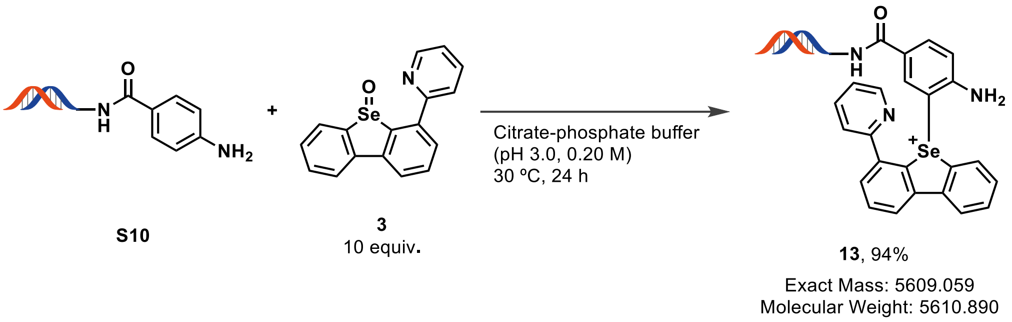

At 20–25 °C, 1.0 µL of **S10** (2.0 mM, 2.0 nmol, 1.0 equiv.) in water was added to a 1.5 mL Eppendorf tube. Next, 4.0 µL of Citrate-phosphate buffer (pH 3.0, c = 500 mM) was added. Then, 5.0 µL of a selenoxide **3** stock solution (4.0 mM, 20 nmol, 10 equiv.) in water was added. The mixture was vortexed for 5 seconds, transferred into a Thermocycler pre-heated at 30 °C, and incubated at 30 °C for 16 hours at 600 rpm. After 16 hours, reaction was quenched by addition of 10 µL of borate buffer (pH 9.4, c = 500 mM), and an aliquot of 2 µL of the reaction mixture was diluted to 40 µL with water for LC–MS analysis.

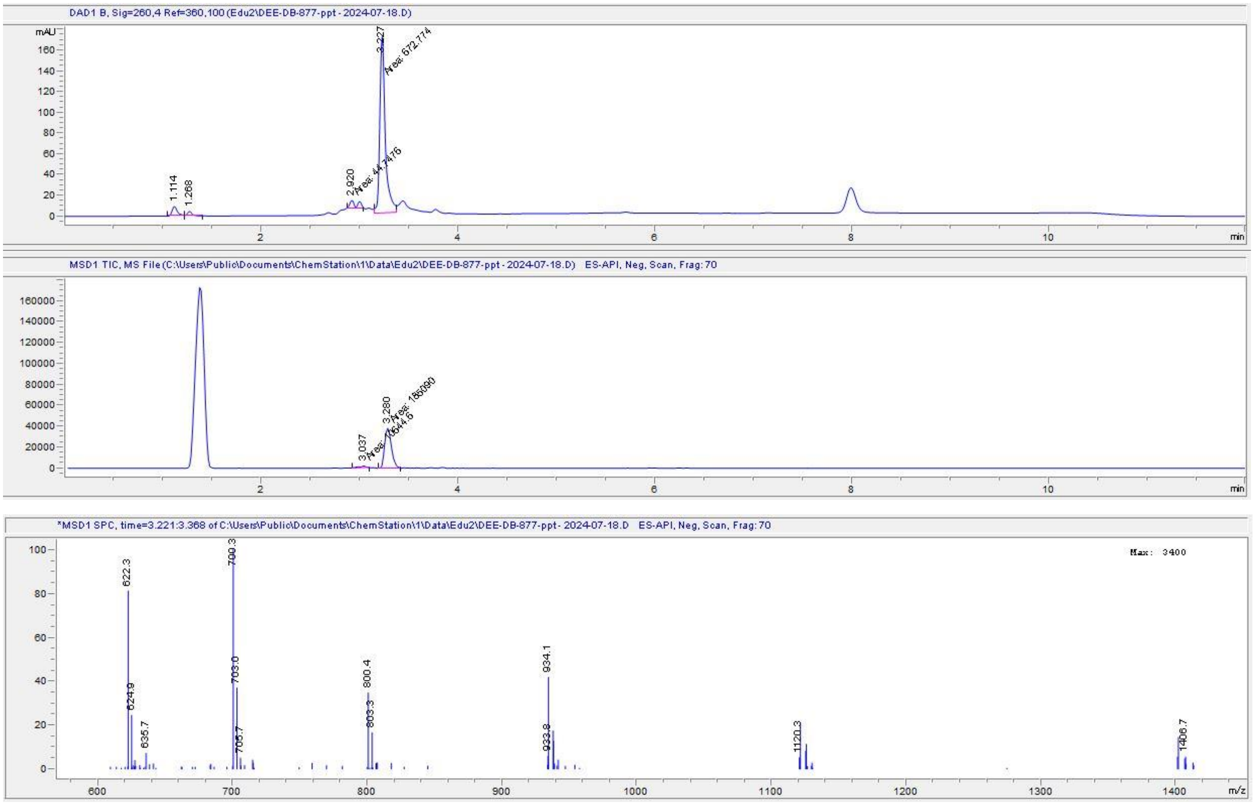

Figure S65. Analytical HPLC trace of **13** with HPLC Method A. (Up) DAD chromatogram at 260 nm. (Middle) TIC chromatogram. (Below) Ionization of peak at 3.280 min containing reaction product.

Synthesis of DNA-conjugated selenonium salt 14

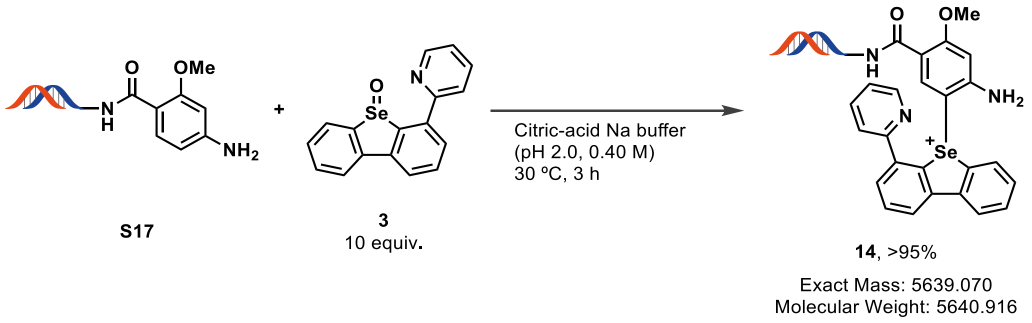

At 20–25 °C, 1.0 µL of **S17** (2.0 mM, 2.0 nmol, 1.0 equiv.) in water was added to a 1.5 mL Eppendorf tube. Next, 4.0 µL of Citrate-phosphate buffer (pH 3.0, c = 500 mM) was added. Then, 5.0 µL of a selenoxide **3** stock solution (4.0 mM, 20 nmol, 10 equiv.) in water was added. The mixture was vortexed for 5 seconds, transferred into a Thermocycler pre-heated at 30 °C, and incubated at 30 °C for 3 hours at 600 rpm. After 16 hours, reaction was quenched by addition of 10 µL of borate buffer (pH 9.4, c = 500 mM), and an aliquot of 2 µL of the reaction mixture was diluted to 40 µL with water for LC–MS analysis.

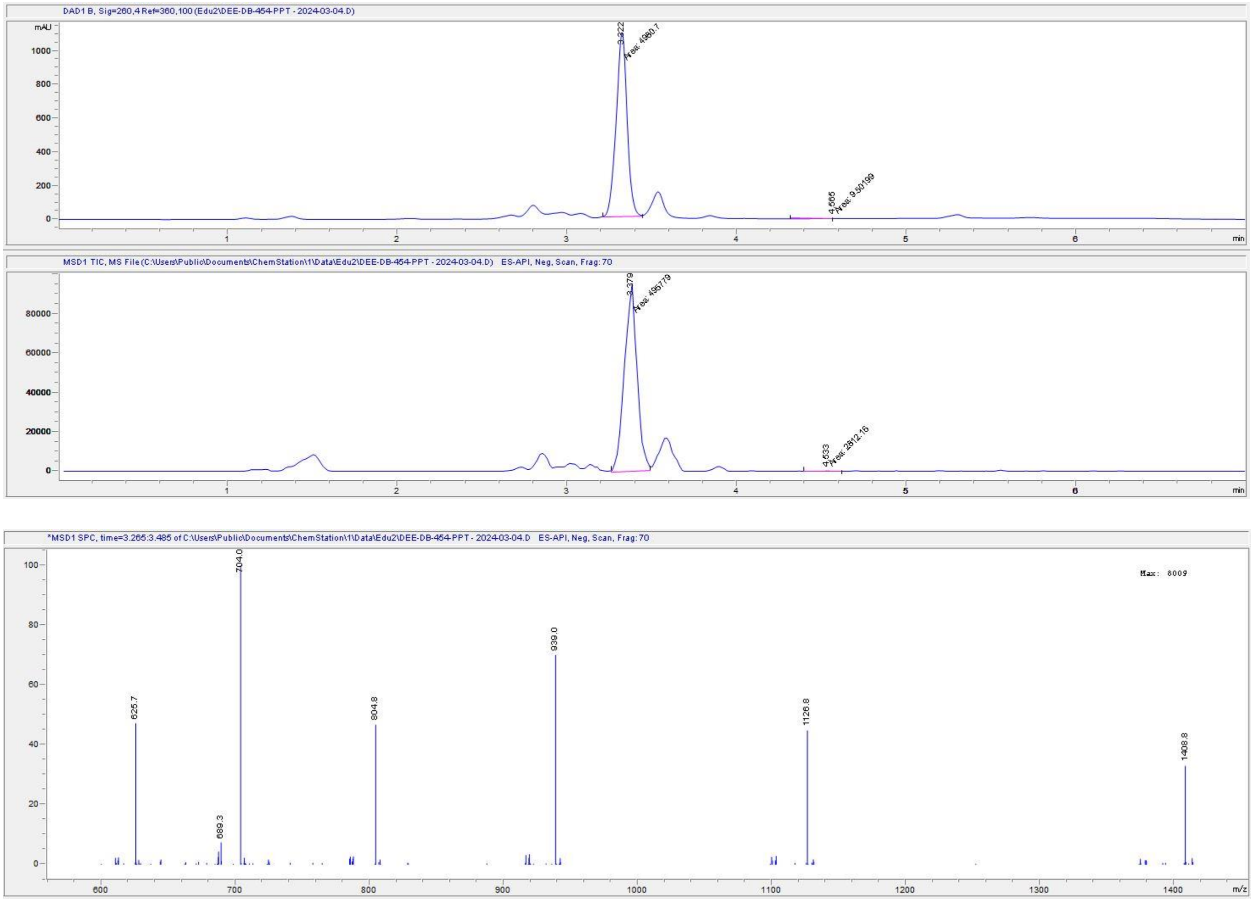

Figure S66. Analytical HPLC trace of **14** with HPLC Method B. (Up) DAD chromatogram at 260 nm. (Middle) TIC chromatogram. (Below) Ionization of peak at 3.379 min containing reaction product.

Synthesis of DNA-conjugated selenonium salt 15

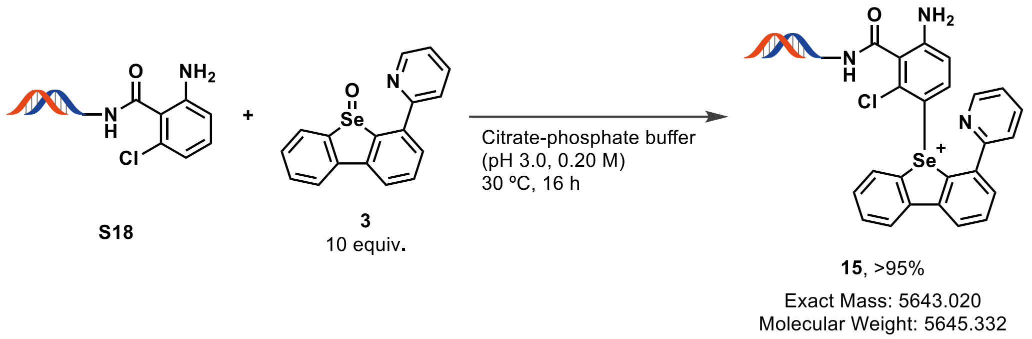

At 20–25 °C, 1.0 µL of **S18** (2.0 mM, 2.0 nmol, 1.0 equiv.) in water was added to a 1.5 mL Eppendorf tube. Next, 4.0 µL of Citrate-phosphate buffer (pH 3.0, c = 500 mM) was added. Then, 5.0 µL of a selenoxide **3** stock solution (4.0 mM, 20 nmol, 10 equiv.) in water was added. The mixture was vortexed for 5 seconds, transferred into a Thermocycler pre-heated at 30 °C, and incubated at 30 °C for 16 hours at 600 rpm. After 16 hours, reaction was quenched by addition of 10 µL of borate buffer (pH 9.4, c = 500 mM), and an aliquot of 2 µL of the reaction mixture was diluted to 40 µL with water for LC–MS analysis.

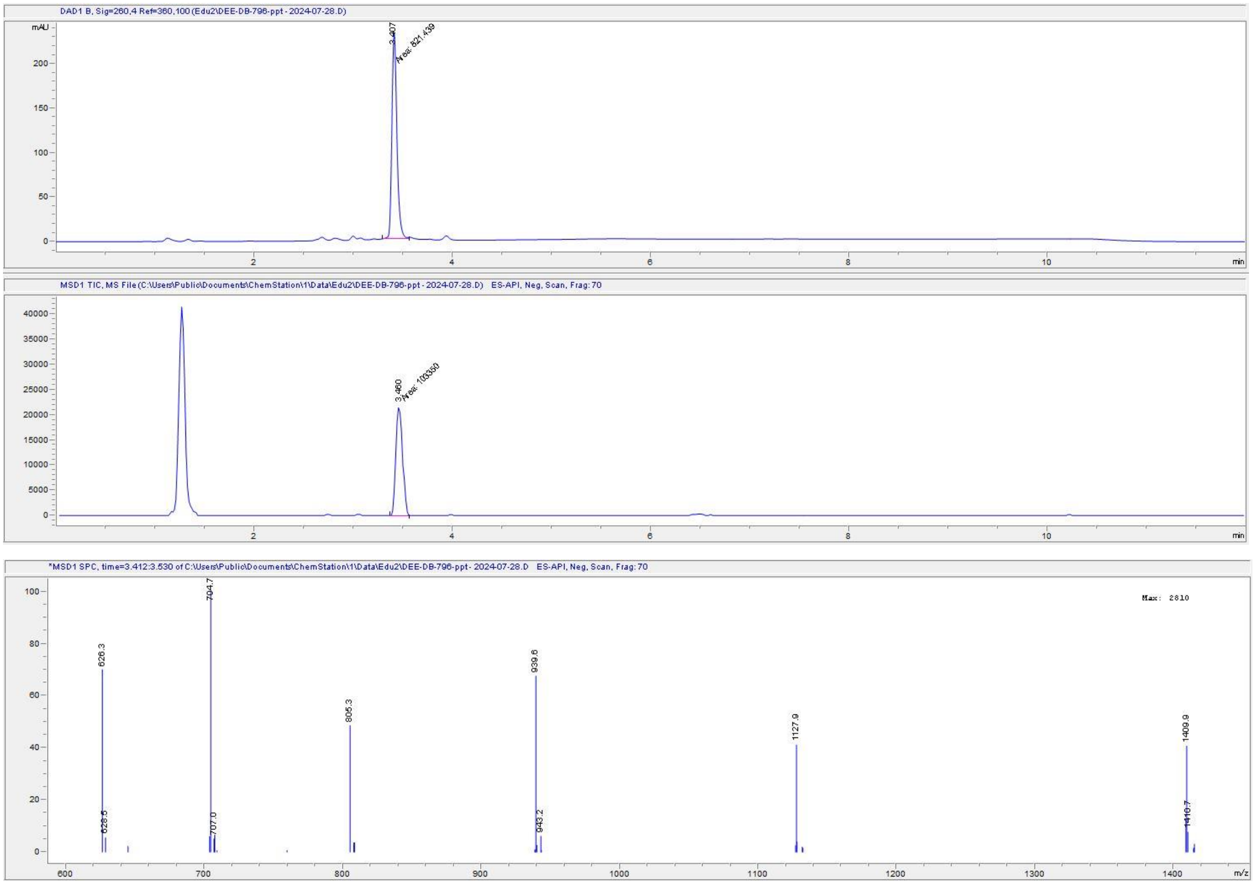

Figure S67. Analytical HPLC trace of **15** with HPLC Method A. (Up) DAD chromatogram at 260 nm. (Middle) TIC chromatogram. (Below) Ionization of peak at 3.460 min containing reaction product.

Synthesis of DNA-conjugated selenonium salt 16

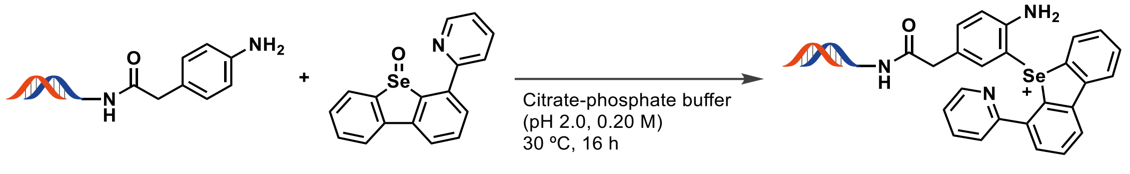

At 20–25 °C, 1.0 µL of **S11** (2.0 mM, 2.0 nmol, 1.0 equiv.) in water was added to a 1.5 mL Eppendorf tube. Next, 4.0 µL of Citrate-phosphate buffer (pH 3.0, c = 500 mM) was added. Then, 5.0 µL of a selenoxide **3** stock solution (4.0 mM, 20 nmol, 10 equiv.) in water was added. The mixture was vortexed for 5 seconds, transferred into a Thermocycler pre-heated at 30 °C, and incubated at 30 °C for 16 hours at 600 rpm. After 16 hours, reaction was quenched by addition of 10 µL of borate buffer (pH 9.4, c = 500 mM), and an aliquot of 2 µL of the reaction mixture was diluted to 40 µL with water for LC–MS analysis.

Oxidation byproducts refer to unidentified byproducts with a MW of **S11**+16 and **S11**+32.

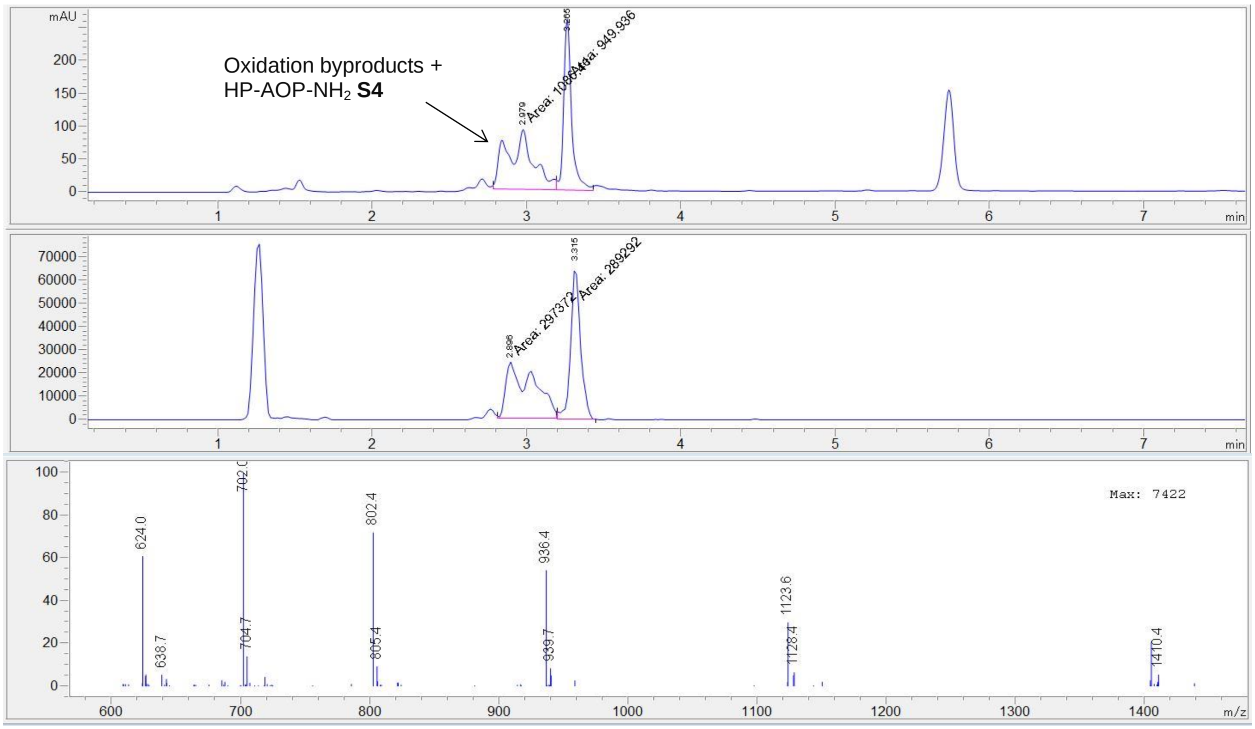

Figure S68. Analytical HPLC trace of **16** with HPLC Method A. (Up) DAD chromatogram at 260 nm. (Middle) TIC chromatogram. (Below) Ionization of peak at 3.315 min containing reaction product.

Synthesis of DNA-conjugated selenonium salt 17

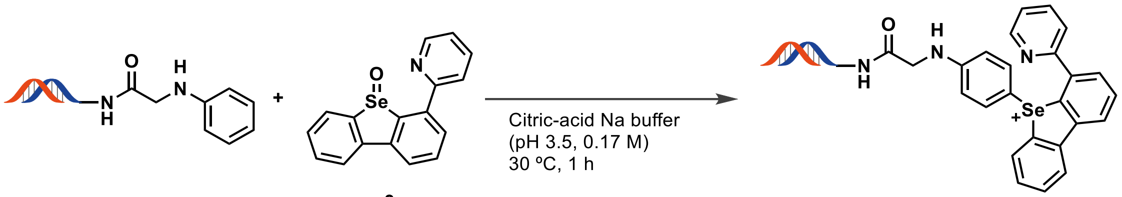

S24   **3** 2 equiv.   **17**, >95%

Exact Mass: 5623.075
Molecular Weight: 5624.917

At 20–25 °C, 1.0 µL of **S24** (2.0 mM, 2.0 nmol, 1.0 equiv.) in water was added to a 1.5 mL Eppendorf tube. Next, 1.0 µL of Citrate-phosphate buffer (pH 3.5, c = 500 mM) was added. Then, 1.0 µL of a selenoxide **3** stock solution (4.0 mM, 4.0 nmol, 2.0 equiv.) in water was added. The mixture was vortexed for 5 seconds, transferred into a Thermocycler pre-heated at 30 °C, and incubated at 30 °C for 1 hour at 600 rpm. After 1 hour, reaction was quenched by addition of 10 µL of borate buffer (pH 9.4, c = 500 mM), and an aliquot of 2 µL of the reaction mixture was diluted to 40 µL with water for LC–MS analysis.

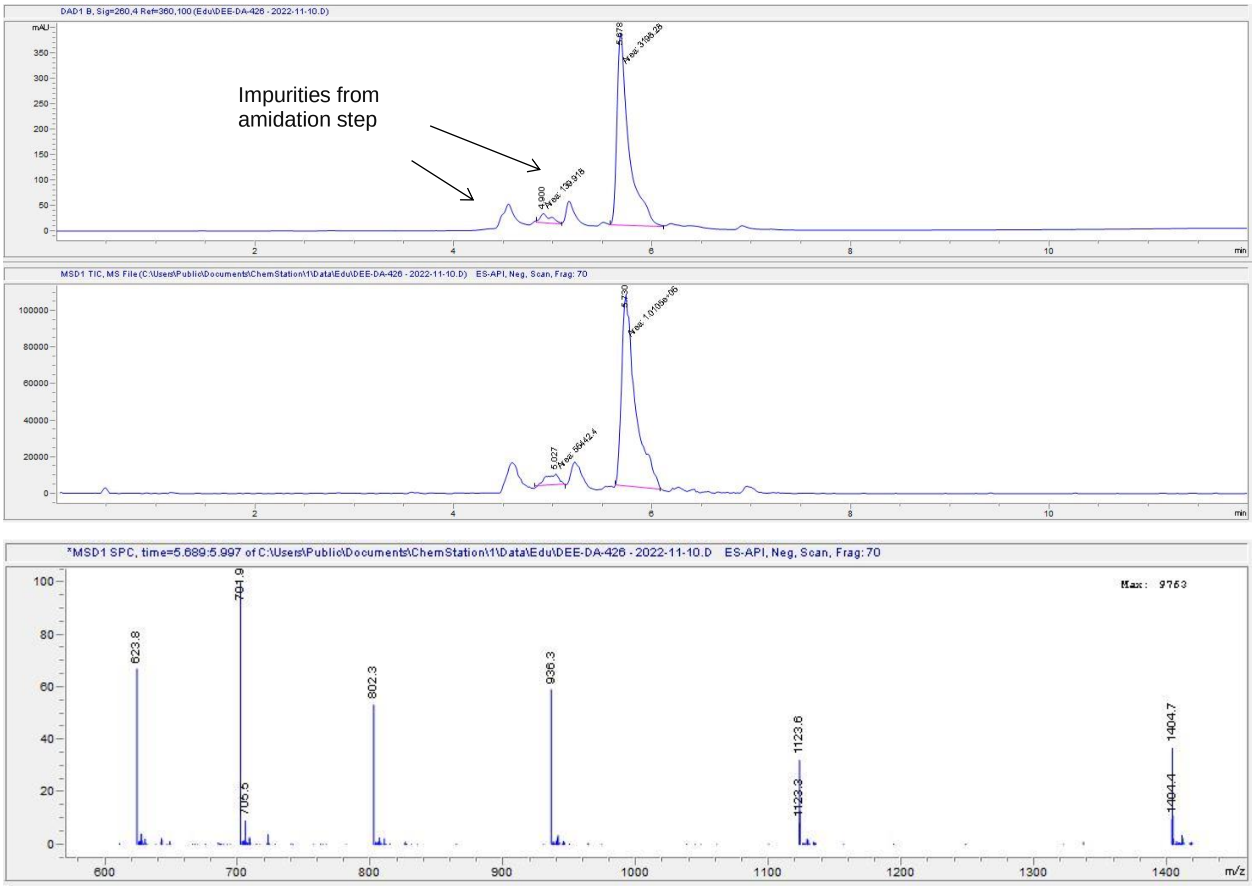

Figure S69. Analytical HPLC trace of **17** with HPLC Method A. (Up) DAD chromatogram at 260 nm. (Middle) TIC chromatogram. (Below) Ionization of peak at 5.730 min containing reaction product.

Synthesis of DNA-conjugated selenonium salt 18

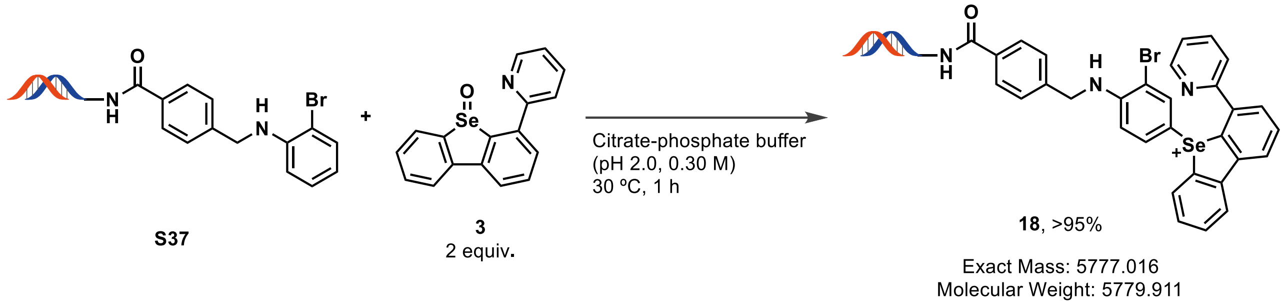

At 20–25 °C, 1.0 µL of **S37** (2.0 mM, 2.0 nmol, 1.0 equiv.) in water was added to a 1.5 mL Eppendorf tube. Next, 1.0 µL of Citrate-phosphate buffer (pH 3.0, c = 500 mM) was added. Then, 1.0 µL of a selenoxide **3** stock solution (4.0 mM, 4.0 nmol, 2.0 equiv.) in water was added. The mixture was vortexed for 5 seconds, transferred into a Thermocycler pre-heated at 30 °C, and incubated at 30 °C for 1 hour at 600 rpm. After 1 hour, reaction was quenched by addition of 10 µL of borate buffer (pH 9.4, c = 500 mM), and an aliquot of 2 µL of the reaction mixture was diluted to 40 µL with water for LC–MS analysis.

*Purity of **18**: 79%. Purity of **S32**: 80%. Conversion of **S32** to **18**: >95%.*

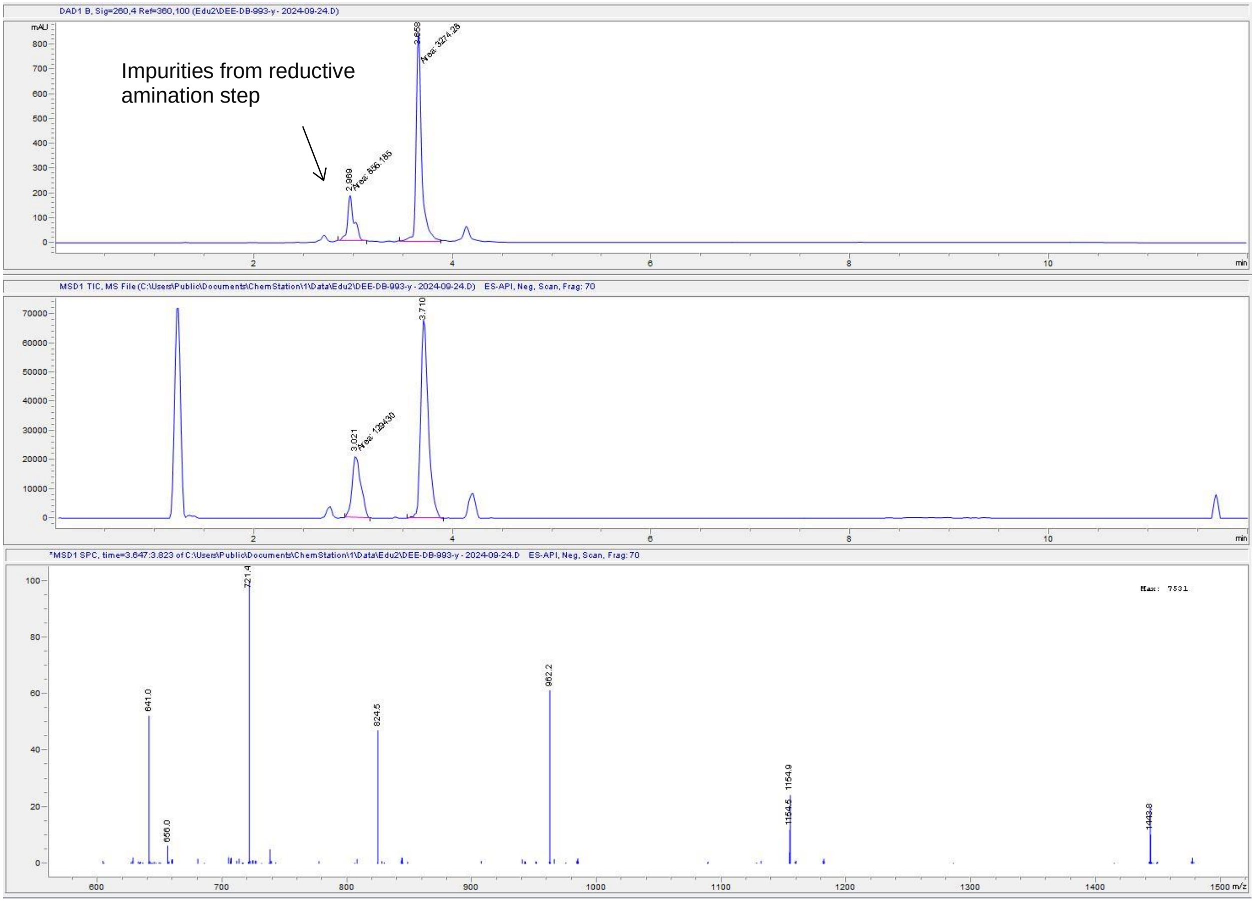

Figure S70. Analytical HPLC trace of **18** with HPLC Method A. (Up) DAD chromatogram at 260 nm. (Middle) TIC chromatogram. (Below) Ionization of peak at 3.710 min containing reaction product.

Synthesis of DNA-conjugated selenonium salt 19

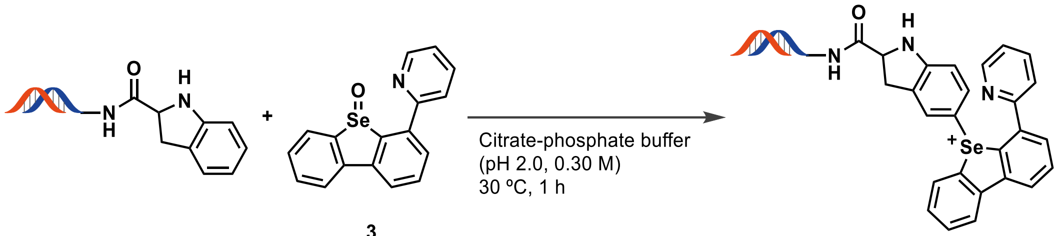

S19 | **3** 2 equiv. | **19**, 65%

Exact Mass: 5635.074
Molecular Weight: 5636.928

At 20–25 °C, 1.0 µL of **S19** (2.0 mM, 2.0 nmol, 1.0 equiv.) in water was added to a 1.5 mL Eppendorf tube. Next, 1.0 µL of Citrate-phosphate buffer (pH 2.0, c = 1.0 M) was added. Then, 1.0 µL of a selenoxide **3** stock solution (4.0 mM, 4.0 nmol, 2.0 equiv.) in water was added. The mixture was vortexed for 5 seconds, transferred into a Thermocycler pre-heated at 30 °C, and incubated at 30 °C for 1 hour at 600 rpm. After 1 hour, reaction was quenched by addition of 10 µL of borate buffer (pH 9.4, c = 500 mM), and an aliquot of 2 µL of the reaction mixture was diluted to 40 µL with water for LC–MS analysis.

Oxidation byproduct refers to an unidentified byproduct with a MW of **S19**+16.

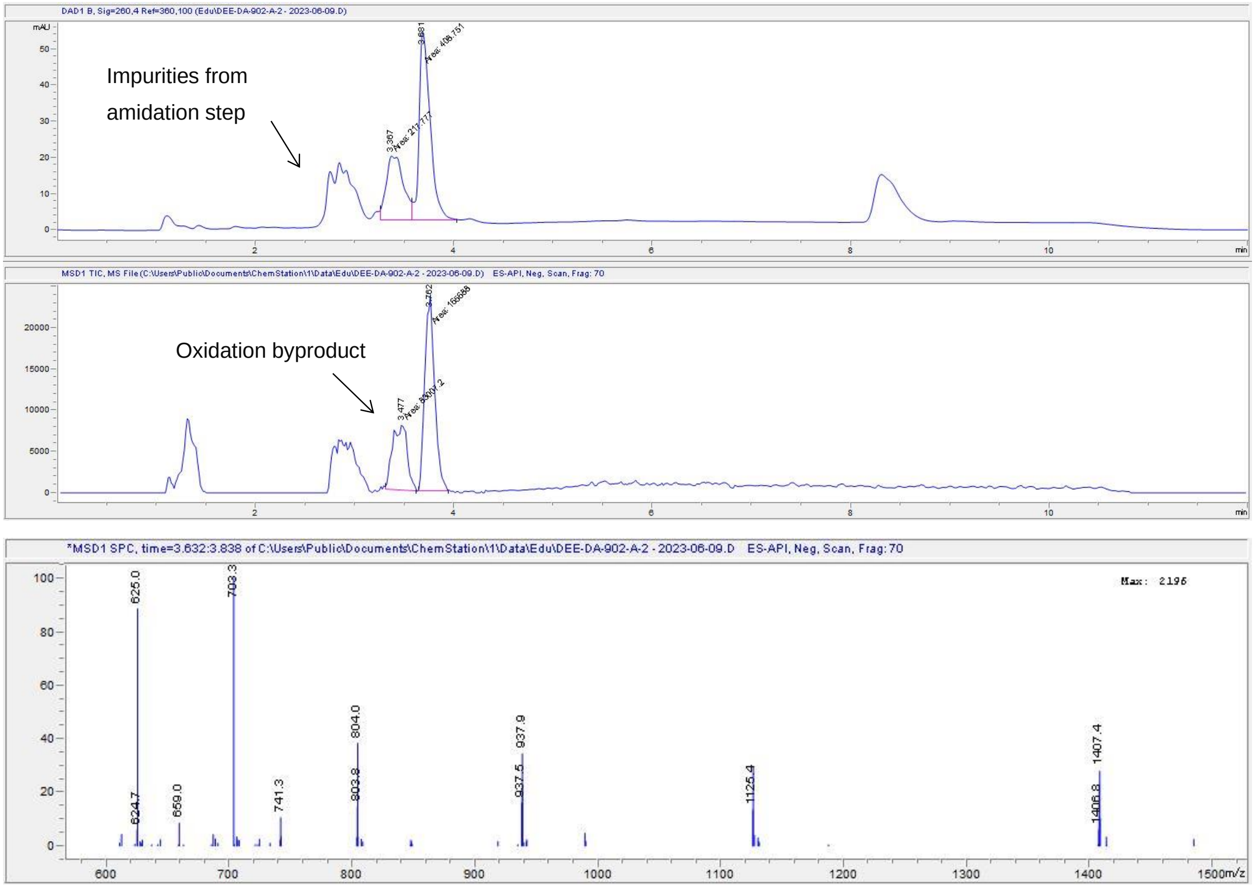

Figure S71. Analytical HPLC trace of **19** with HPLC Method A. (Up) DAD chromatogram at 260 nm. (Middle) TIC chromatogram. (Below) Ionization of peak at 3.762 min containing reaction product.

Synthesis of DNA-conjugated selenonium salt 20

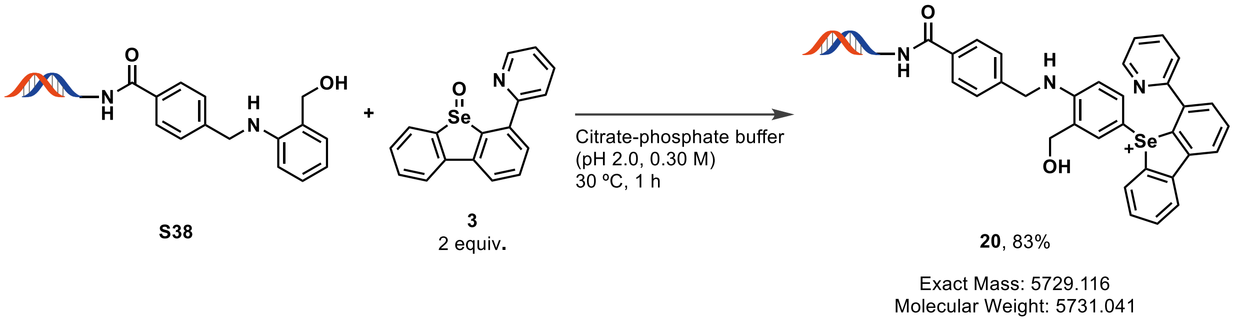

At 20–25 °C, 1.0 µL of **S38** (2.0 mM, 2.0 nmol, 1.0 equiv.) in water was added to a 1.5 mL Eppendorf tube. Next, 1.0 µL of Citrate-phosphate buffer (pH 2.0, c = 1.0 M) was added. Then, 1.0 µL of a selenoxide **3** stock solution (4.0 mM, 4.0 nmol, 2.0 equiv.) in water was added. The mixture was vortexed for 5 seconds, transferred into a Thermocycler pre-heated at 30 °C, and incubated at 30 °C for 1 hour at 600 rpm. After 1 hour, reaction was quenched by addition of 10 µL of borate buffer (pH 9.4, c = 500 mM), and an aliquot of 2 µL of the reaction mixture was diluted to 40 µL with water for LC–MS analysis.

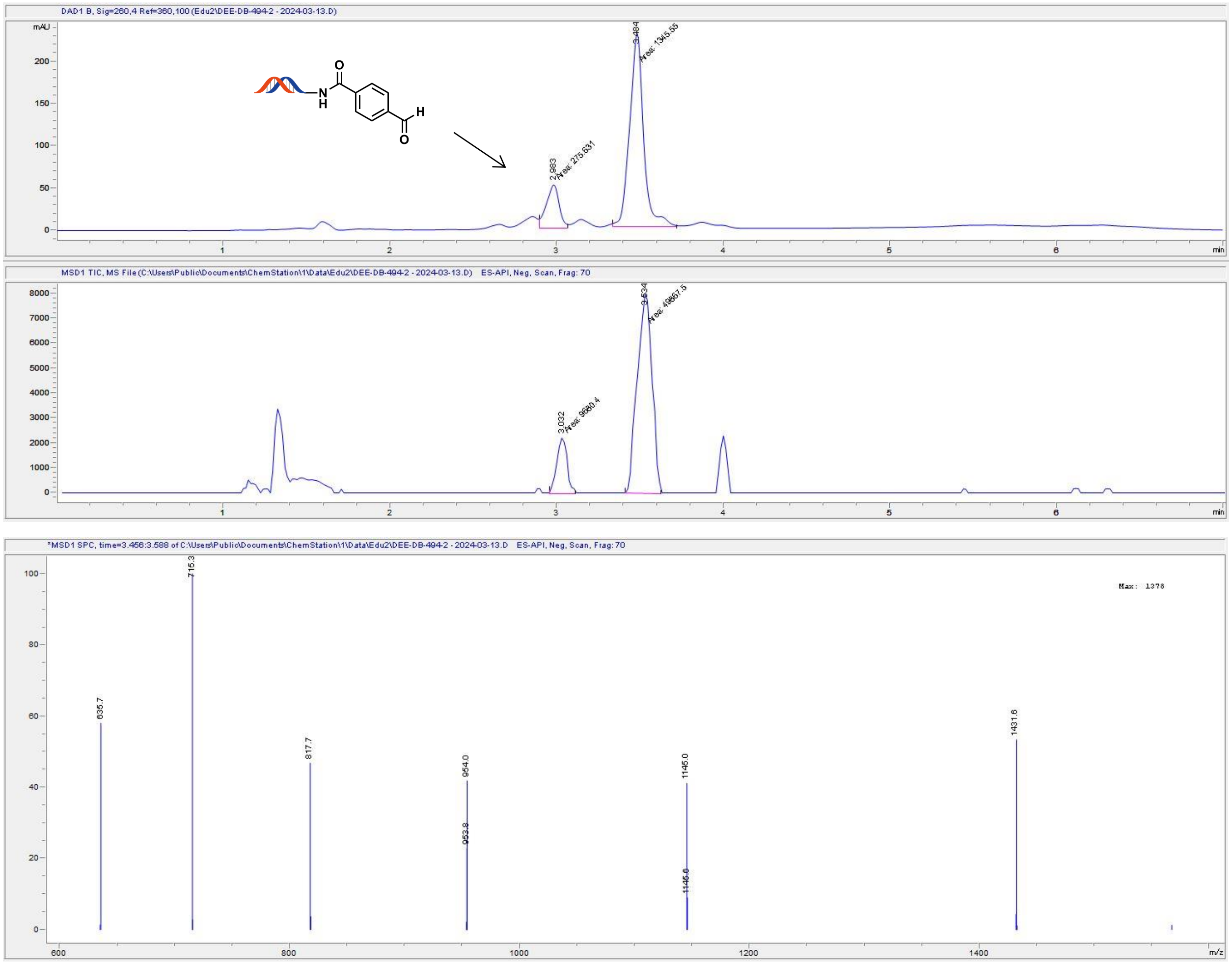

Figure S72. Analytical HPLC trace of **20** with HPLC Method B. (Up) DAD chromatogram at 260 nm. (Middle) TIC chromatogram. (Below) Ionization of peak at 3.534 min containing reaction product.

Synthesis of DNA-conjugated selenonium salt 21

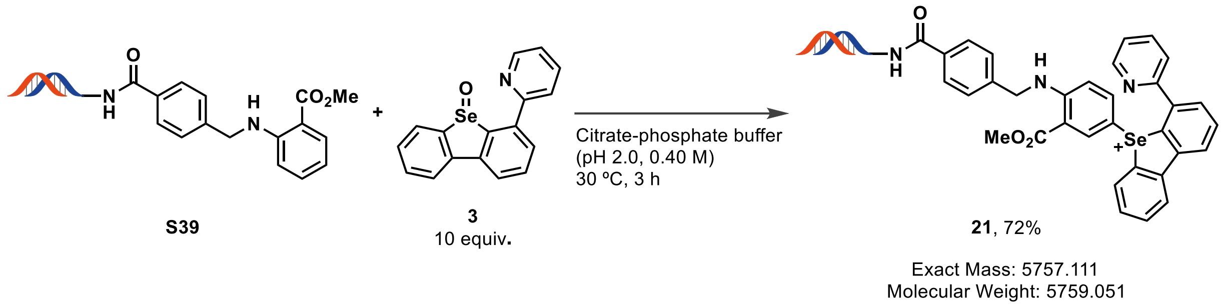

At 20–25 °C, 1.0 µL of **S39** (2.0 mM, 2.0 nmol, 1.0 equiv.) in water was added to a 1.5 mL Eppendorf tube. Next, 4.0 µL of Citrate-phosphate buffer (pH 2.0, c = 1.0 M) was added. Then, 5.0 µL of a selenoxide **3** stock solution (4.0 mM, 20 nmol, 10 equiv.) in water was added. The mixture was vortexed for 5 seconds, transferred into a Thermocycler pre-heated at 30 °C, and incubated at 30 °C for 3 hours at 600 rpm. After 3 hours, reaction was quenched by addition of 10 µL of borate buffer (pH 9.4, c = 500 mM), and an aliquot of 2 µL of the reaction mixture was diluted to 40 µL with water for LC–MS analysis.

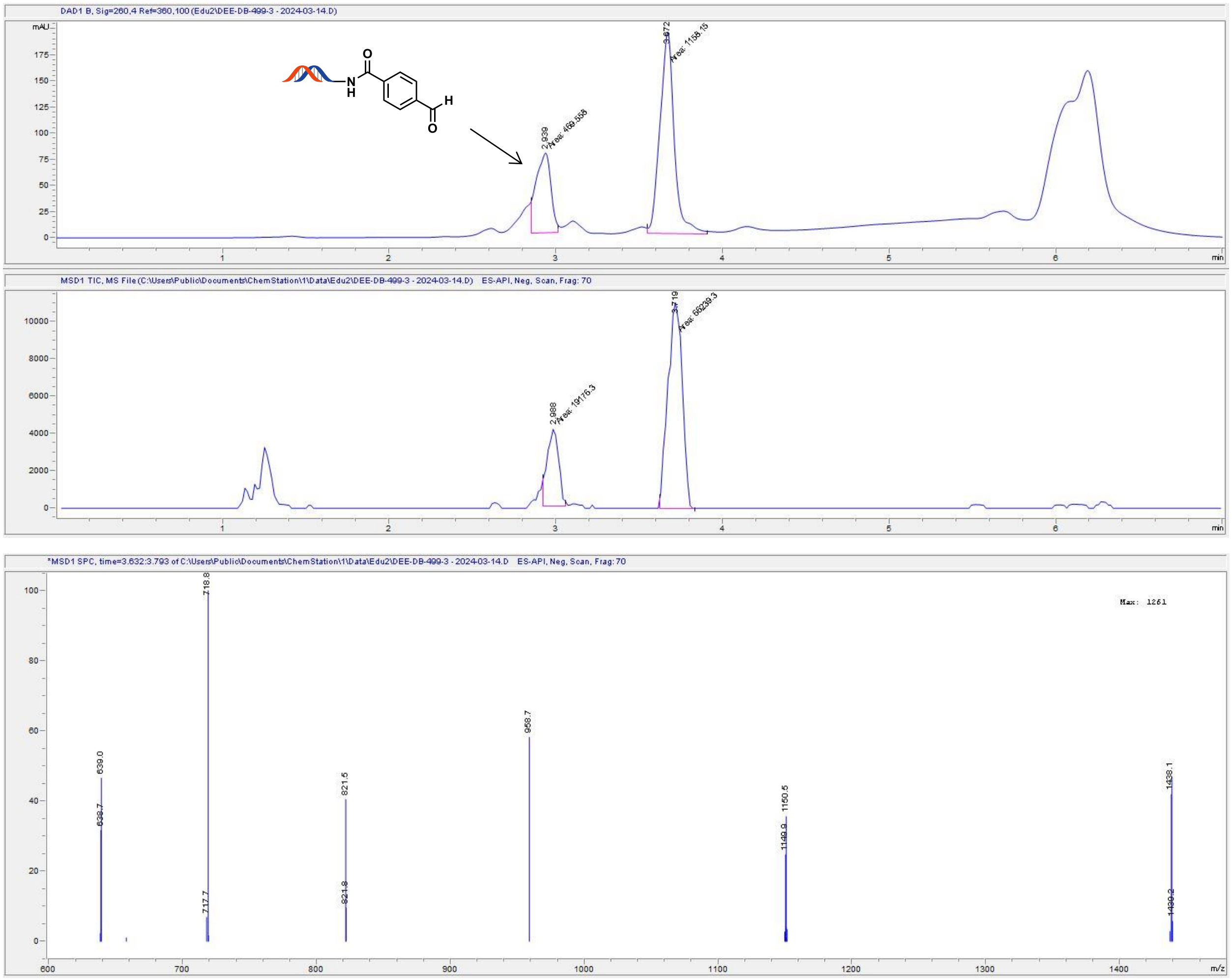

Figure S73. Analytical HPLC trace of **21** with HPLC Method B. (Up) DAD chromatogram at 260 nm. (Middle) TIC chromatogram. (Below) Ionization of peak at 3.719 min containing reaction product.

Synthesis of DNA-conjugated selenonium salt 22

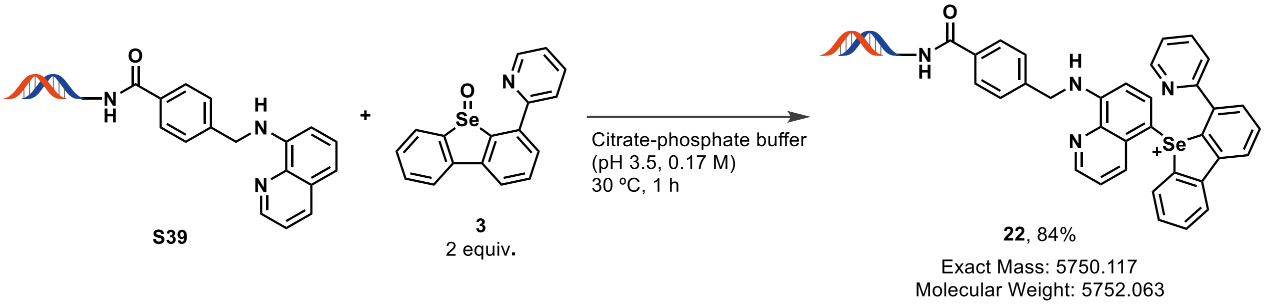

At 20–25 °C, 1.0 µL of **S39** (2.0 mM, 2.0 nmol, 1.0 equiv.) in water was added to a 1.5 mL Eppendorf tube. Next, 1.0 µL of Citrate-phosphate buffer (pH 3.5, c = 500 mM) was added. Then, 1.0 µL of a selenoxide **3** stock solution (4.0 mM, 4.0 nmol, 2.0 equiv.) in water was added. The mixture was vortexed for 5 seconds, transferred into a Thermocycler pre-heated at 30 °C, and incubated at 30 °C for 1 hour at 600 rpm. After 1 hour, reaction was quenched by addition of 10 µL of borate buffer (pH 9.4, c = 500 mM), and an aliquot of 2 µL of the reaction mixture was diluted to 40 µL with water for LC–MS analysis.

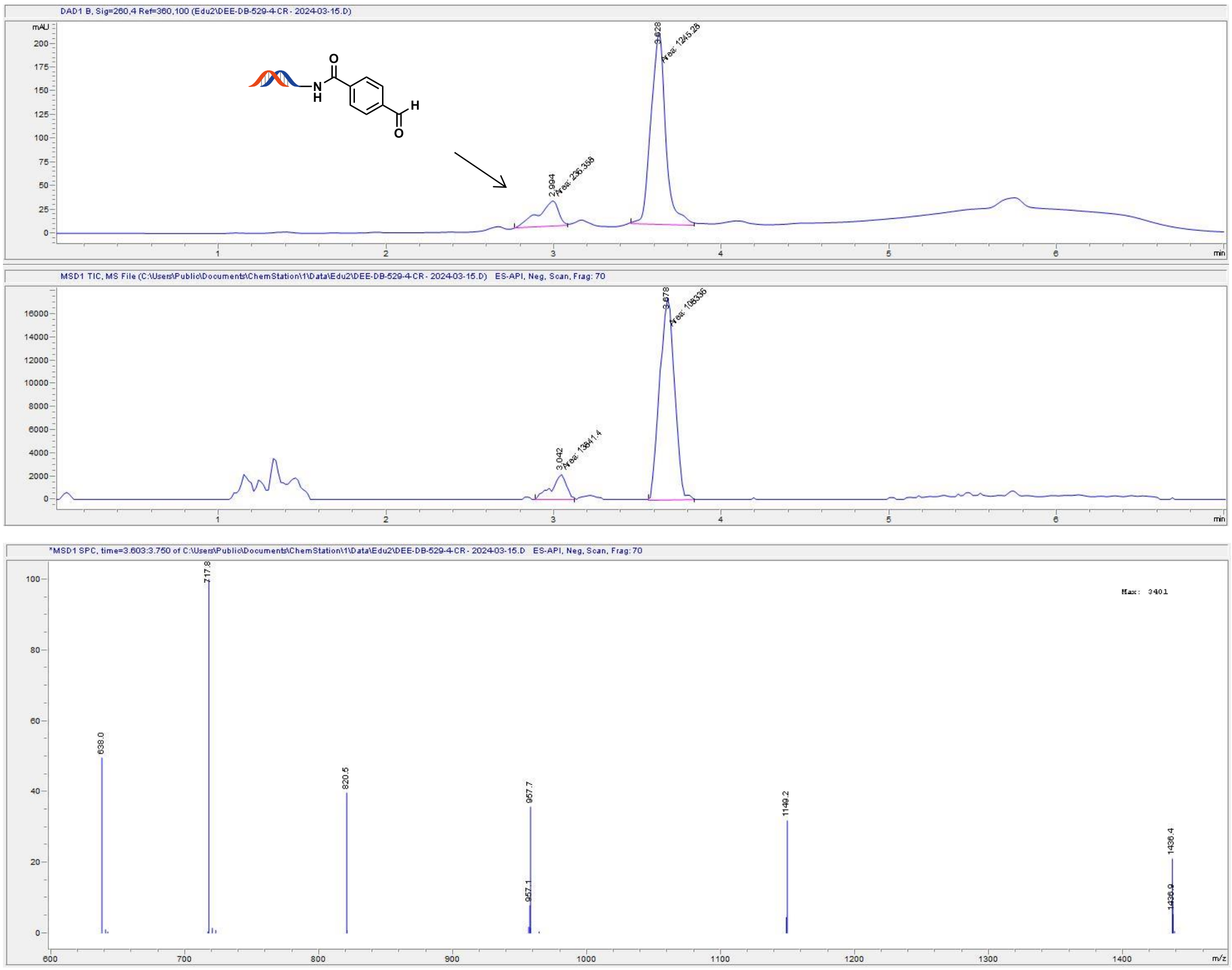

Figure S74. Analytical HPLC trace of **22** with HPLC Method B. (Up) DAD chromatogram at 260 nm. (Middle) TIC chromatogram. (Below) Ionization of peak at 3.678 min containing reaction product.

Synthesis of DNA-conjugated selenonium salt 23

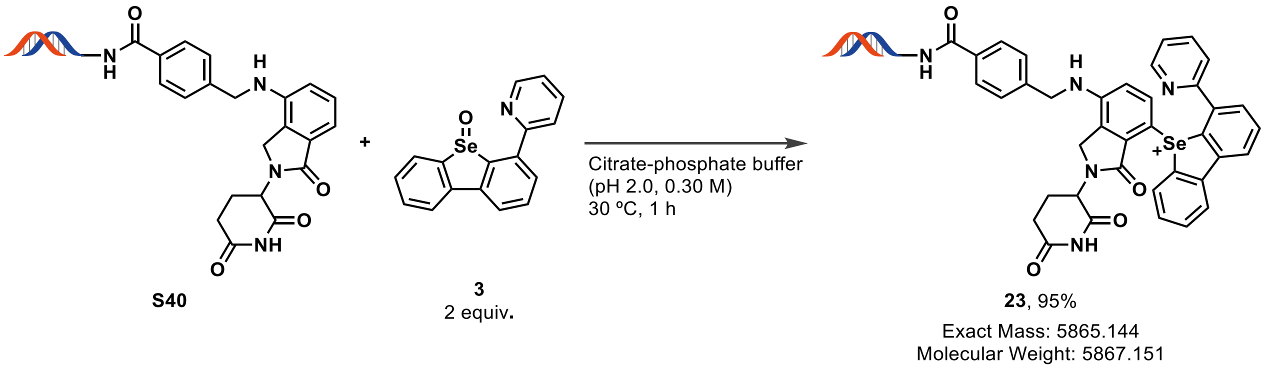

At 20–25 °C, 1.0 µL of **S40** (2.0 mM, 2.0 nmol, 1.0 equiv.) in water was added to a 1.5 mL Eppendorf tube. Next, 1.0 µL of Citrate-phosphate buffer (pH 2.0, c = 1.0 M) was added. Then, 1.0 µL of a selenoxide **3** stock solution (4.0 mM, 4.0 nmol, 2.0 equiv.) in water was added. The mixture was vortexed for 5 seconds, transferred into a Thermocycler pre-heated at 30 °C, and incubated at 30 °C for 1 hour at 600 rpm. After 1 hour, reaction was quenched by addition of 10 µL of borate buffer (pH 9.4, c = 500 mM), and an aliquot of 2 µL of the reaction mixture was diluted to 40 µL with water for LC–MS analysis.

*Purity of **23**: 76%. Purity of **S36**: 80%. Conversion of **S36** to **23**: 95%.*

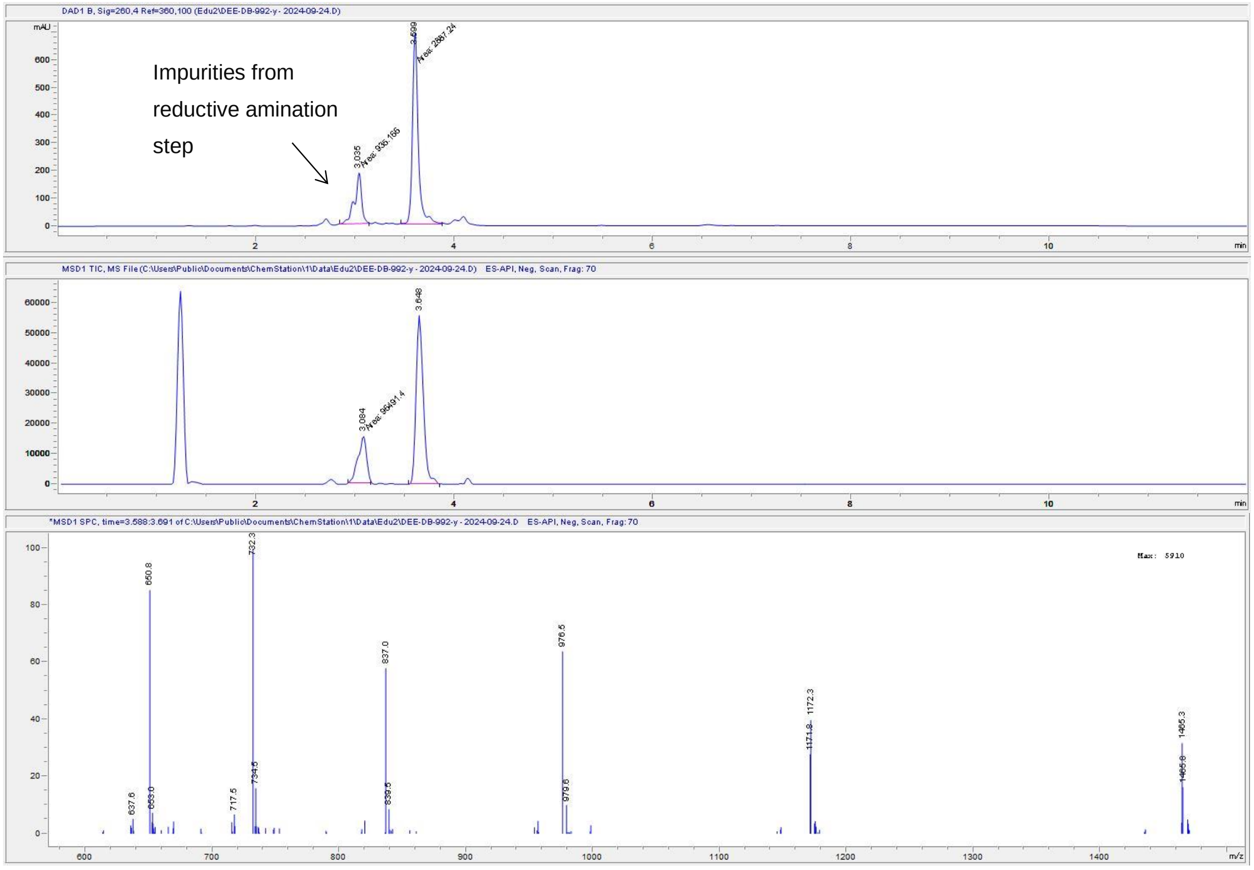

Figure S75. Analytical HPLC trace of **23** with HPLC Method A. (Up) DAD chromatogram at 260 nm. (Middle) TIC chromatogram. (Below) Ionization of peak at 3.648 min containing reaction product.

Synthesis of DNA-conjugated selenonium salt 24

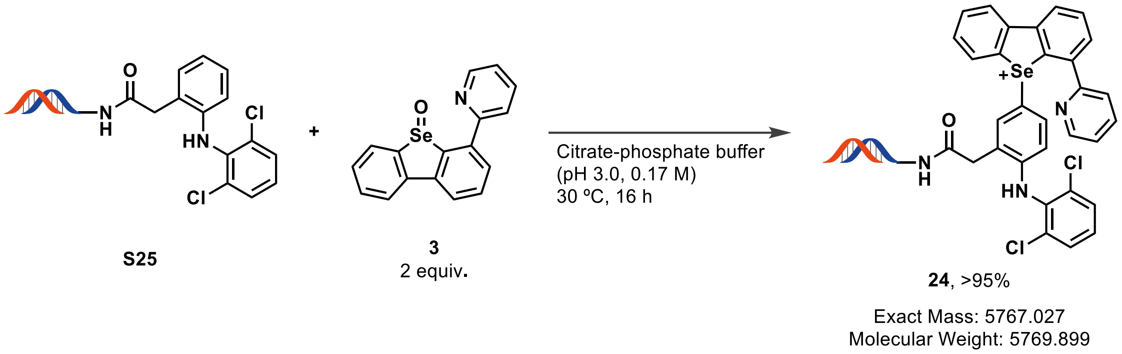

At 20–25 °C, 200 µL of **S25** (2.0 mM, 400 nmol, 1.0 equiv.) in water was added to two 1.5 mL Eppendorf tubes. Next, 200 µL of Citrate-phosphate buffer (pH 3.0, c = 500 mM) was added. Then, 200 µL of a selenoxide **3** stock solution (2.0 mM, 800 nmol, 2.0 equiv.) in water was added. The mixture was vortexed for 5 seconds, transferred into a Thermocycler pre-heated at 30 °C, and incubated at 30 °C for 16 hours at 600 rpm. After 16 hours, reaction was quenched by addition of 400 µL of borate buffer (pH 9.4, c = 500 mM), and an aliquot of 2 µL of the reaction mixture was diluted to 40 µL with water for LC–MS analysis.

The solution of DNA conjugate was then desalted. DNA desalting and rebuffering was performed by charging the solution in an AMICON® filter unit from Sigma Aldrich (3 kD) in 300 µL of water, centrifuged at 4 °C and 10000 x g for at least 30 minutes, until the volume decreased to < 10 µL. Another 300 µL of water were added and the process was repeated all over again for at least 3 times. The remaining solution concentration was determined by A_{260} absorption using a Thermo Scientific™ NanoDrop™ One^{C}, concentration of the solution was adjusted to 2.0 mM and stored in the freezer at −20 °C.

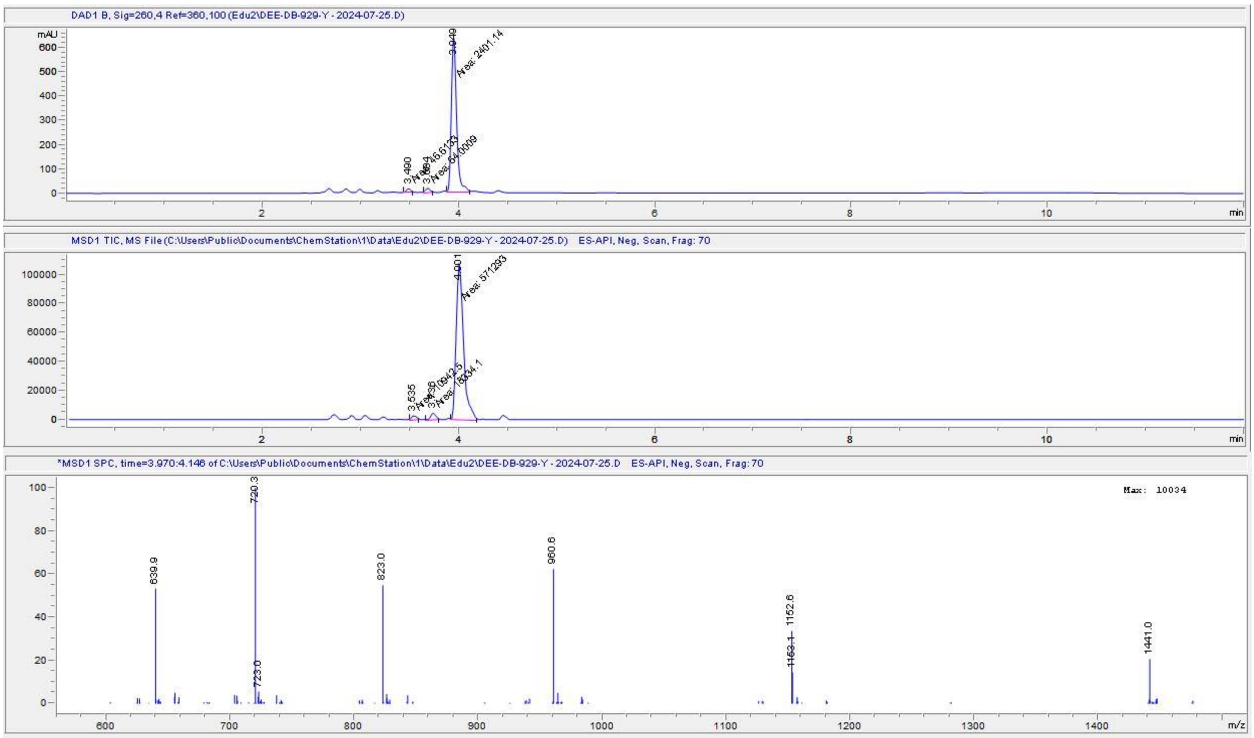

Figure S76. Analytical HPLC trace of **24** with HPLC Method A. (Up) DAD chromatogram at 260 nm. (Middle) TIC chromatogram. (Below) Ionization of peak at 4.001 min containing reaction product.

Synthesis of DNA-conjugated selenonium salt 25

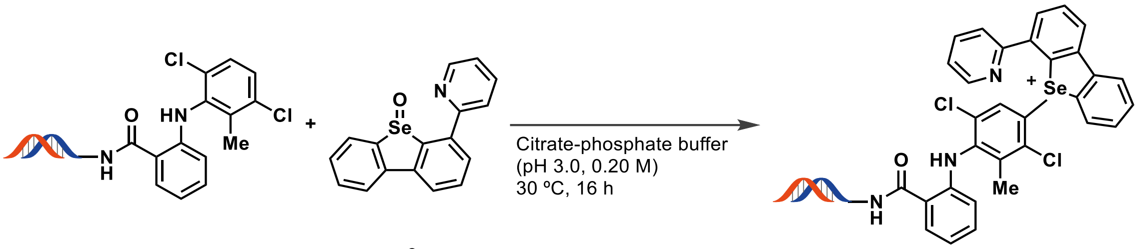

S20

3
10 equiv.

25, 95%
Exact Mass: 5767.027
Molecular Weight: 5769.899

At 20–25 °C, 1.0 µL of **S20** (2.0 mM, 2.0 nmol, 1.0 equiv.) in water was added to a 1.5 mL Eppendorf tube. Next, 4.0 µL of Citrate-phosphate buffer (pH 3.0, c = 500 mM) was added. Then, 5.0 µL of a selenoxide **3** stock solution (4.0 mM, 20 nmol, 10 equiv.) in water was added. The mixture was vortexed for 5 seconds, transferred into a Thermocycler pre-heated at 30 °C, and incubated at 30 °C for 16 hours at 600 rpm. After 16 hours, reaction was quenched by addition of 10 µL of borate buffer (pH 9.4, c = 500 mM), and an aliquot of 2 µL of the reaction mixture was diluted to 40 µL with water for LC–MS analysis.

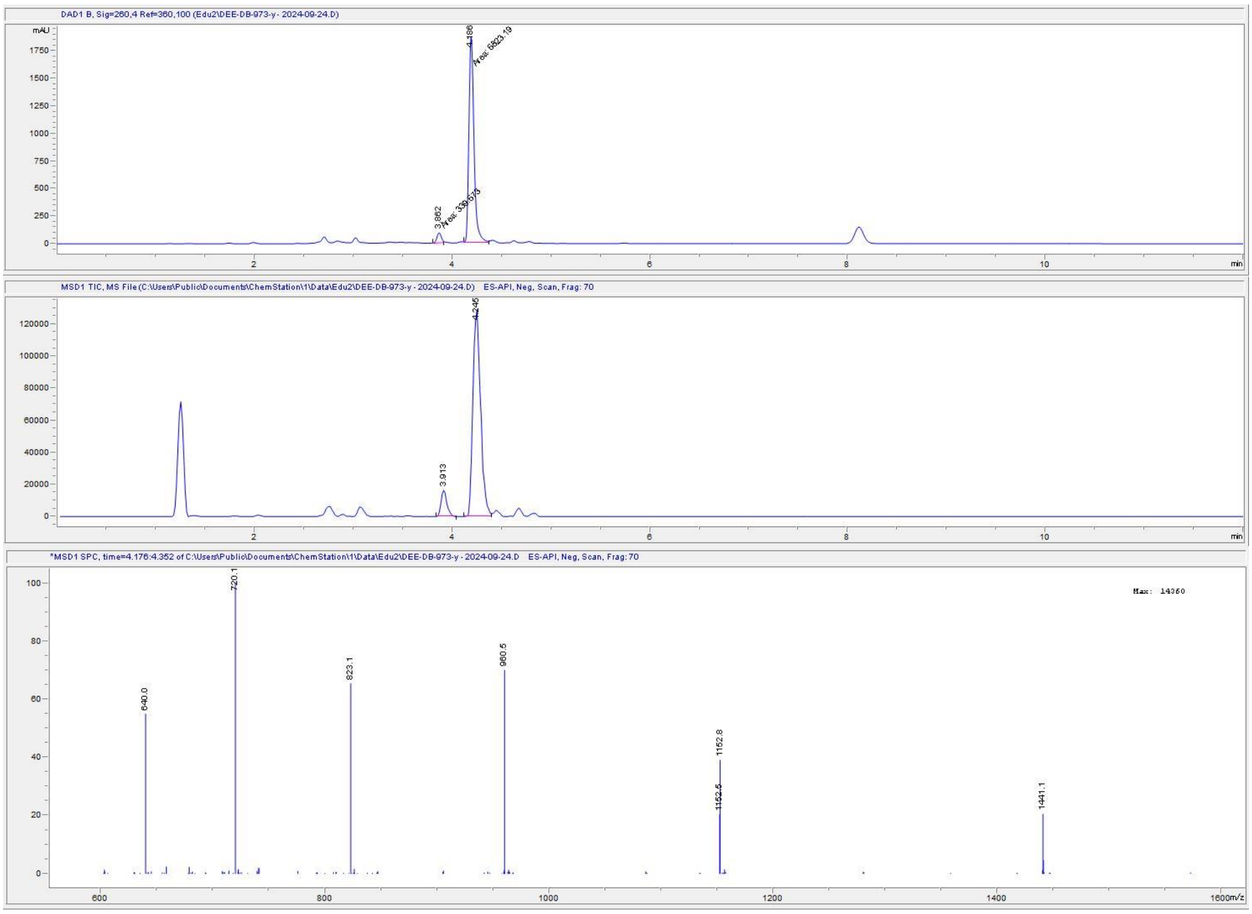

Figure S77. Analytical HPLC trace of **25** with HPLC Method A. (Up) DAD chromatogram at 260 nm. (Middle) TIC chromatogram. (Below) Ionization of peak at 4.245 min containing reaction product.

Synthesis of DNA-conjugated selenonium salt 26

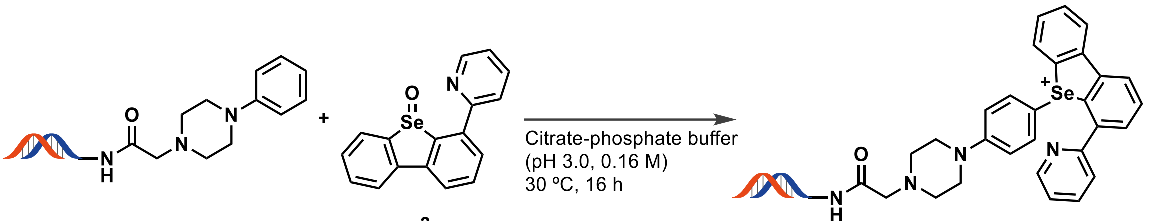

S13 **3** 2 equiv. **26**, >95%

Exact Mass: 5692.132
Molecular Weight: 5694.024

At 20–25 °C, 1.0 µL of **S13** (2.0 mM, 2.0 nmol, 1.0 equiv.) in water was added to a 1.5 mL Eppendorf tube. Next, 1.0 µL of Citrate-phosphate buffer (pH 3.0, c = 500 mM) was added. Then, 1.0 µL of a selenoxide **3** stock solution (4.0 mM, 4.0 nmol, 2.0 equiv.) in water was added. The mixture was vortexed for 5 seconds, transferred into a Thermocycler pre-heated at 30 °C, and incubated at 30 °C for 16 hours at 600 rpm. After 16 hours, reaction was quenched by addition of 10 µL of borate buffer (pH 9.4, c = 500 mM), and an aliquot of 2 µL of the reaction mixture was diluted to 40 µL with water for LC–MS analysis.

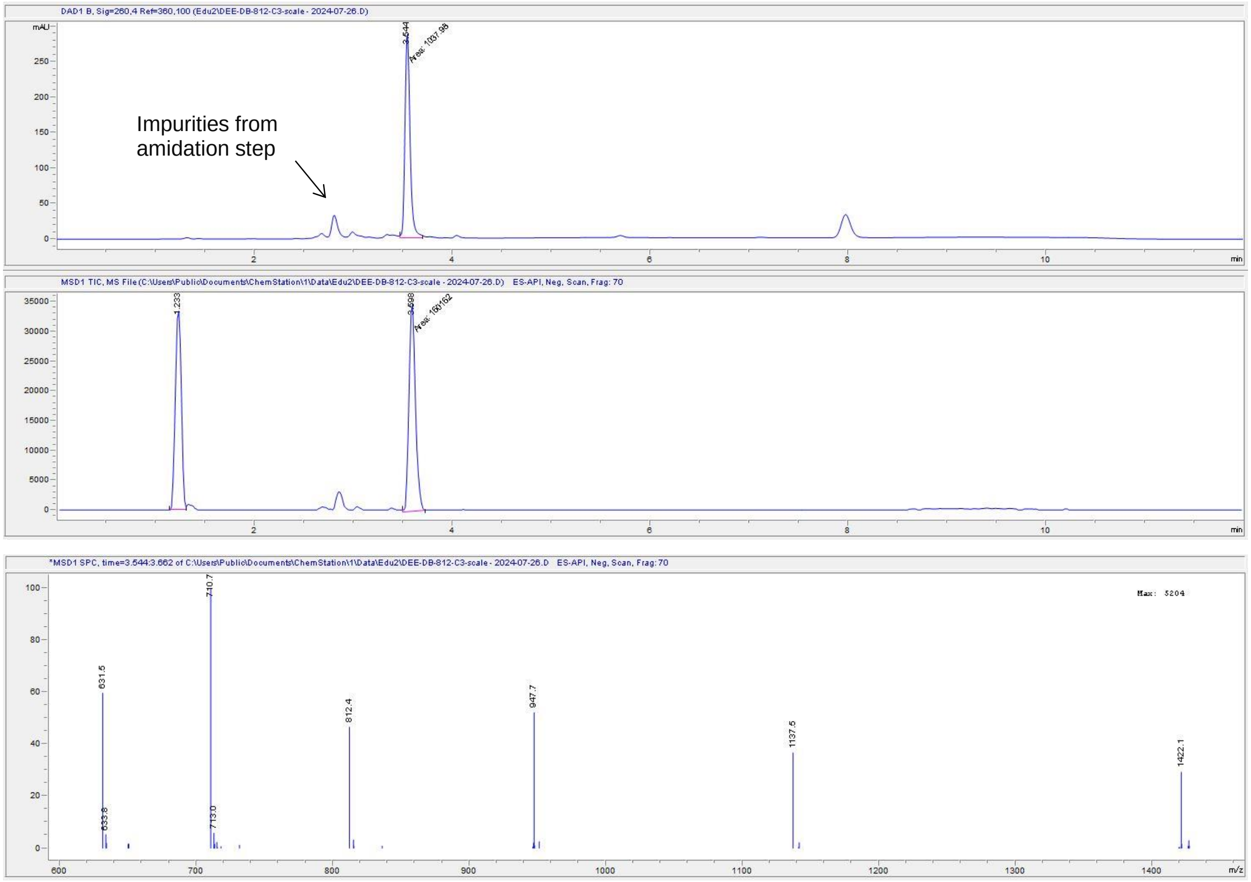

Figure S78. Analytical HPLC trace of **26** with HPLC Method A. (Up) DAD chromatogram at 260 nm. (Middle) TIC chromatogram. (Below) Ionization of peak at 3.598 min containing reaction product.

Synthesis of DNA-conjugated selenonium salt 27

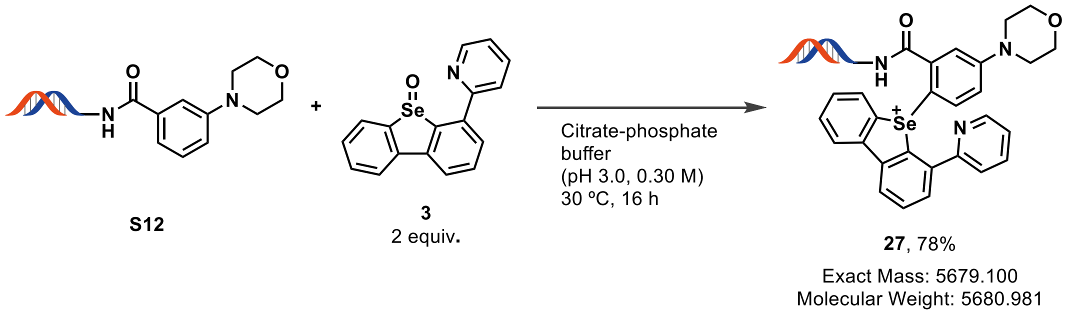

At 20–25 °C, 1.0 µL of **S12** (2.0 mM, 2.0 nmol, 1.0 equiv.) in water was added to a 1.5 mL Eppendorf tube. Next, 1.0 µL of Citrate-phosphate buffer (pH 3.0, c = 500 mM) was added. Then, 1.0 µL of a selenoxide **3** stock solution (4.0 mM, 4.0 nmol, 2.0 equiv.) in water was added. The mixture was vortexed for 5 seconds, transferred into a Thermocycler pre-heated at 30 °C, and incubated at 30 °C for 16 hours at 600 rpm. After 16 hours, reaction was quenched by addition of 10 µL of borate buffer (pH 9.4, c = 500 mM), and an aliquot of 2 µL of the reaction mixture was diluted to 40 µL with water for LC–MS analysis.

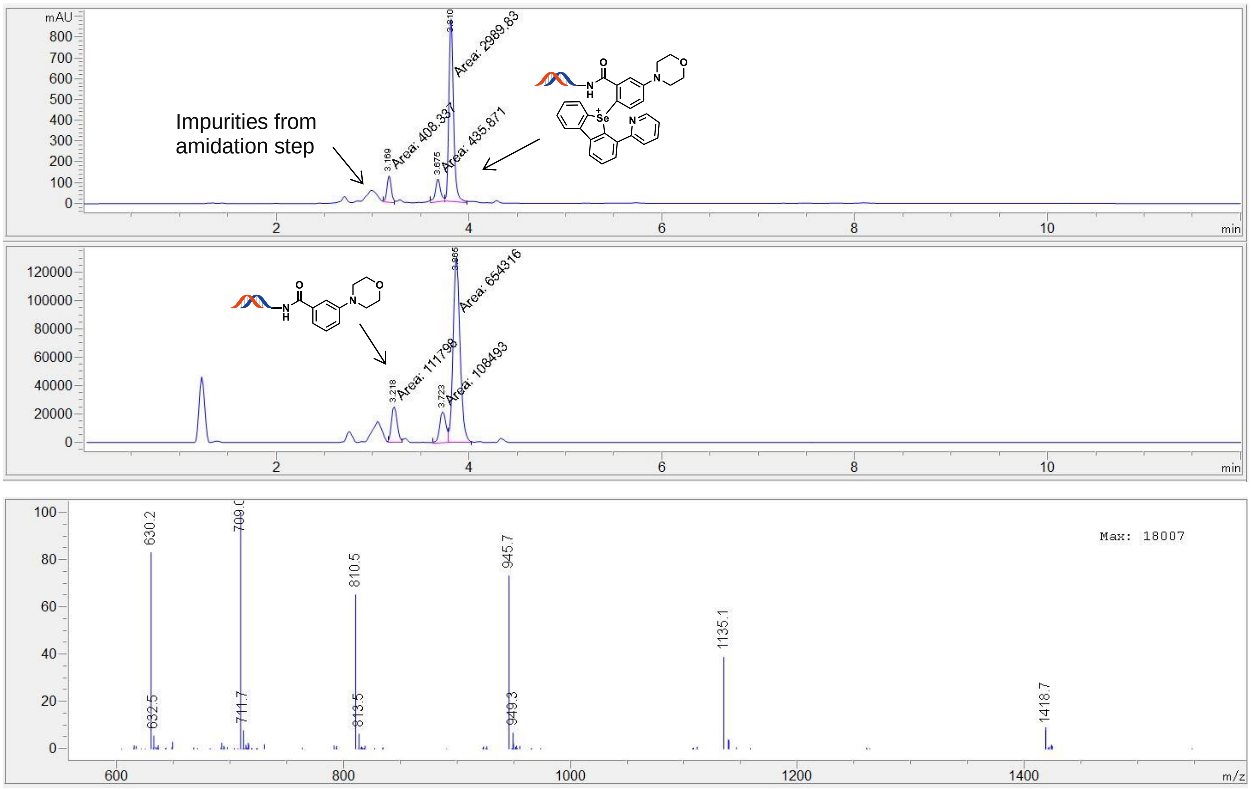

Figure S79. Analytical HPLC trace of **27** with HPLC Method A. (Up) DAD chromatogram at 260 nm. (Middle) TIC chromatogram. (Below) Ionization of peak at 3.865 min containing reaction product.

Synthesis of DNA-conjugated selenonium salt 28

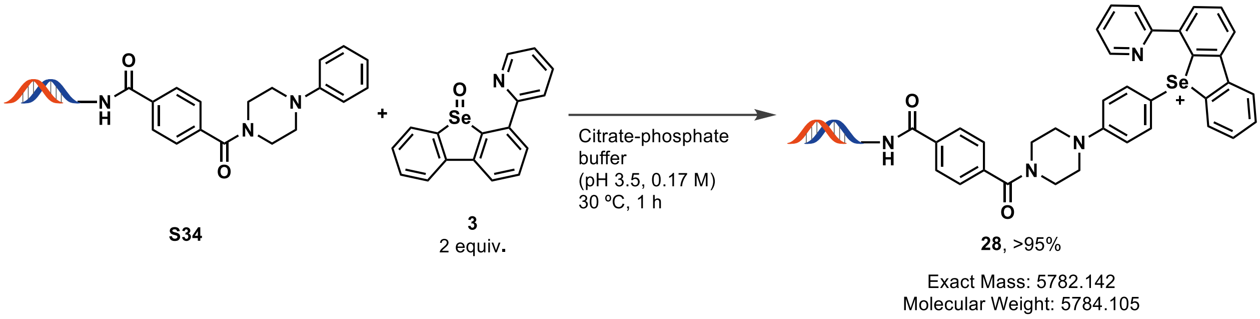

At 20–25 °C, 1.0 µL of **S34** (2.0 mM, 2.0 nmol, 1.0 equiv.) in water was added to a 1.5 mL Eppendorf tube. Next, 1.0 µL of Citrate-phosphate buffer (pH 3.5, c = 500 mM) was added. Then, 1.0 µL of a selenoxide **3** stock solution (4.0 mM, 4.0 nmol, 2.0 equiv.) in water was added. The mixture was vortexed for 5 seconds, transferred into a Thermocycler pre-heated at 30 °C, and incubated at 30 °C for 1 hour at 600 rpm. After 1 hour, reaction was quenched by addition of 10 µL of borate buffer (pH 9.4, c = 500 mM), and an aliquot of 2 µL of the reaction mixture was diluted to 40 µL with water for LC–MS analysis.

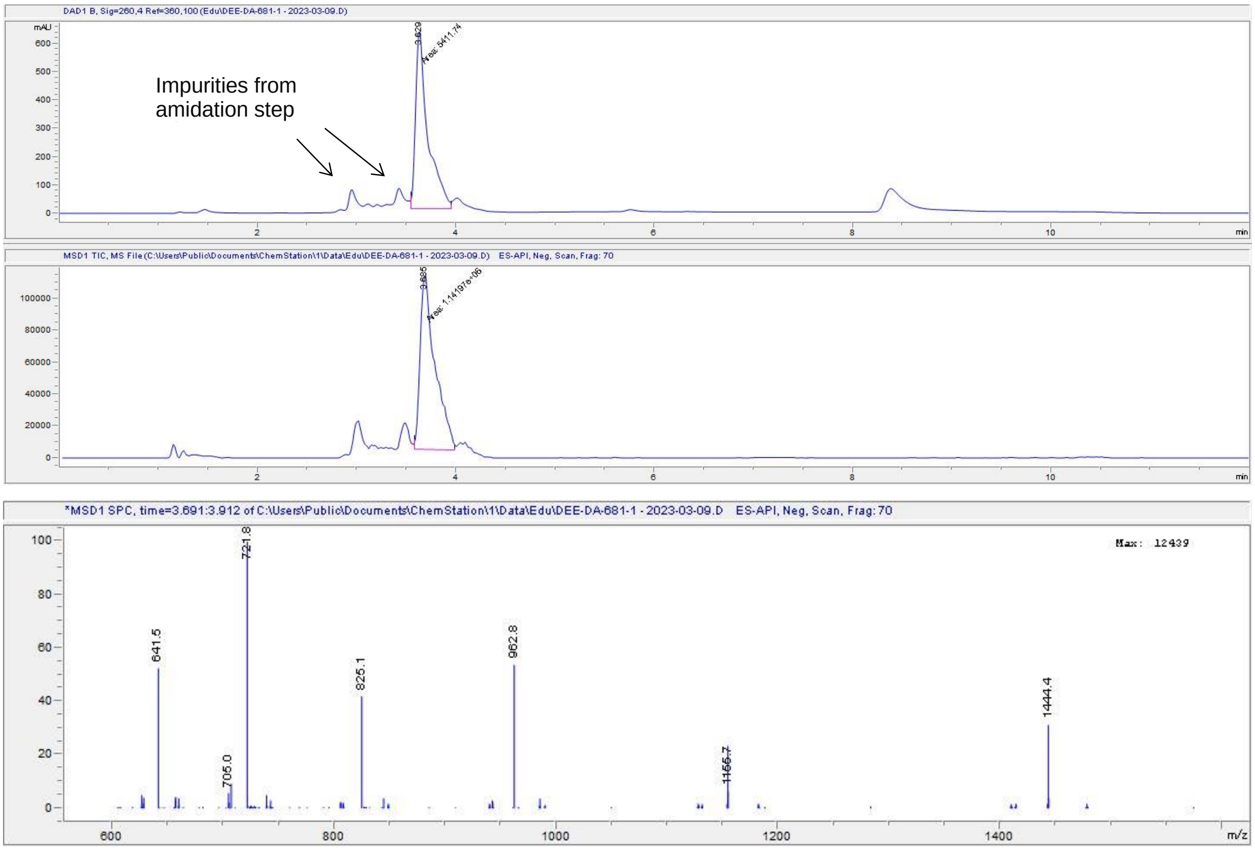

Figure S80. Analytical HPLC trace of **28** with HPLC Method A. (Up) DAD chromatogram at 260 nm. (Middle) TIC chromatogram. (Below) Ionization of peak at 3.685 min containing reaction product.

Synthesis of DNA-conjugated selenonium salt 29

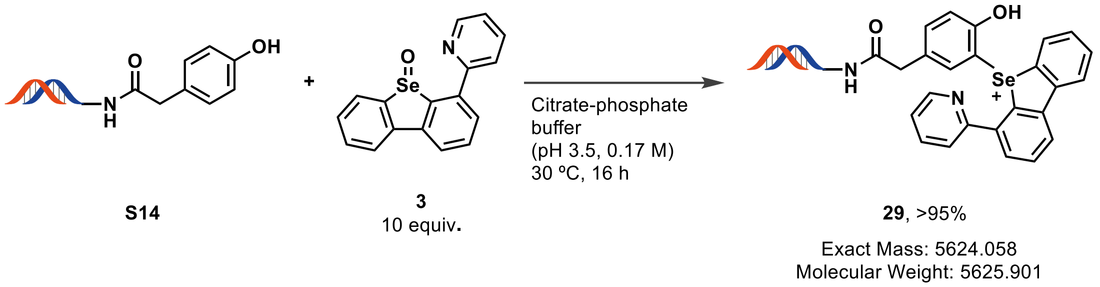

At 20–25 °C, 1.0 µL of **S14** (2.0 mM, 2.0 nmol, 1.0 equiv.) in water was added to a 1.5 mL Eppendorf tube. Next, 4.0 µL of Citrate-phosphate buffer (pH 3.5, c = 500 mM) was added. Then, 5.0 µL of a selenoxide **3** stock solution (4.0 mM, 20 nmol, 10 equiv.) in water was added. The mixture was vortexed for 5 seconds, transferred into a Thermocycler pre-heated at 30 °C, and incubated at 30 °C for 16 hours at 600 rpm. After 16 hours, reaction was quenched by addition of 10 µL of borate buffer (pH 9.4, c = 500 mM), and an aliquot of 2 µL of the reaction mixture was diluted to 40 µL with water for LC–MS analysis.

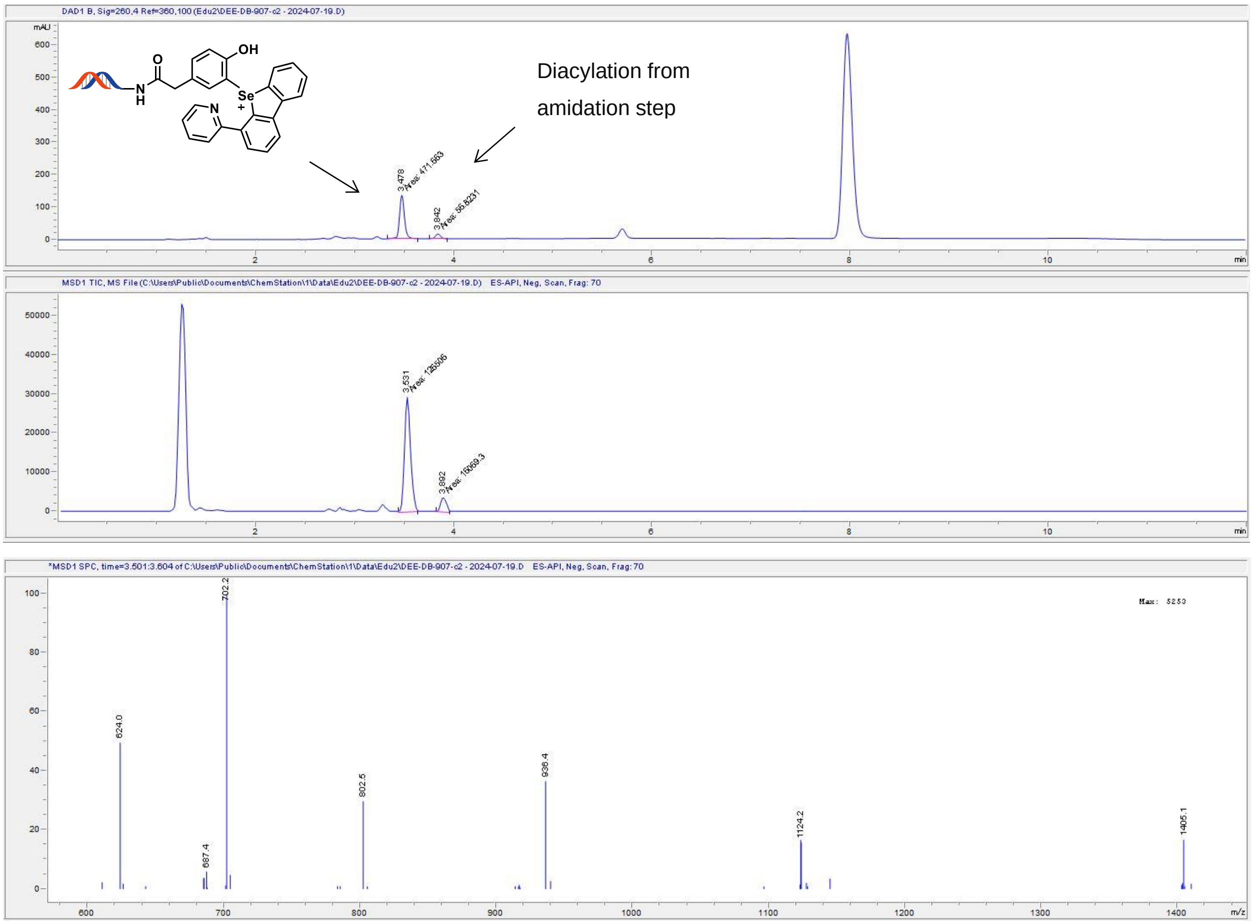

Figure S81. Analytical HPLC trace of **29** with HPLC Method A. (Up) DAD chromatogram at 260 nm. (Middle) TIC chromatogram. (Below) Ionization of peak at 3.531 min containing reaction product.

Synthesis of DNA-conjugated selenonium salt 30

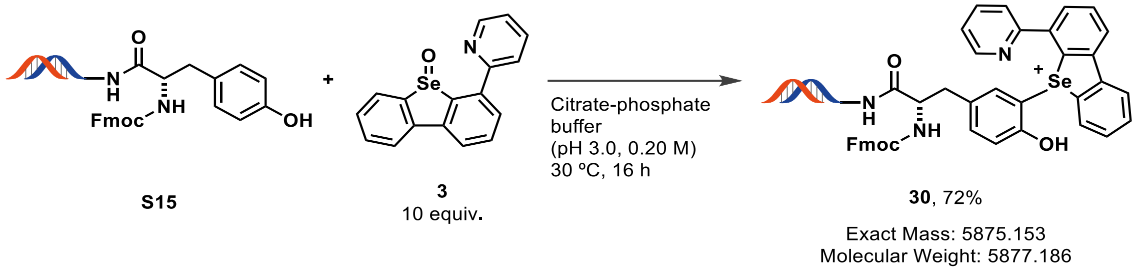

At 20–25 °C, 1.0 µL of **S15** (2.0 mM, 2.0 nmol, 1.0 equiv.) in water was added to a 1.5 mL Eppendorf tube. Next, 4.0 µL of Citrate-phosphate buffer (pH 3.0, c = 500 mM) was added. Then, 5.0 µL of a selenoxide **3** stock solution (4.0 mM, 20 nmol, 10 equiv.) in water was added. The mixture was vortexed for 5 seconds, transferred into a Thermocycler pre-heated at 30 °C, and incubated at 30 °C for 16 hours at 600 rpm. After 16 hours, reaction was quenched by addition of 10 µL of borate buffer (pH 9.4, c = 500 mM), and an aliquot of 2 µL of the reaction mixture was diluted to 40 µL with water for LC–MS analysis.

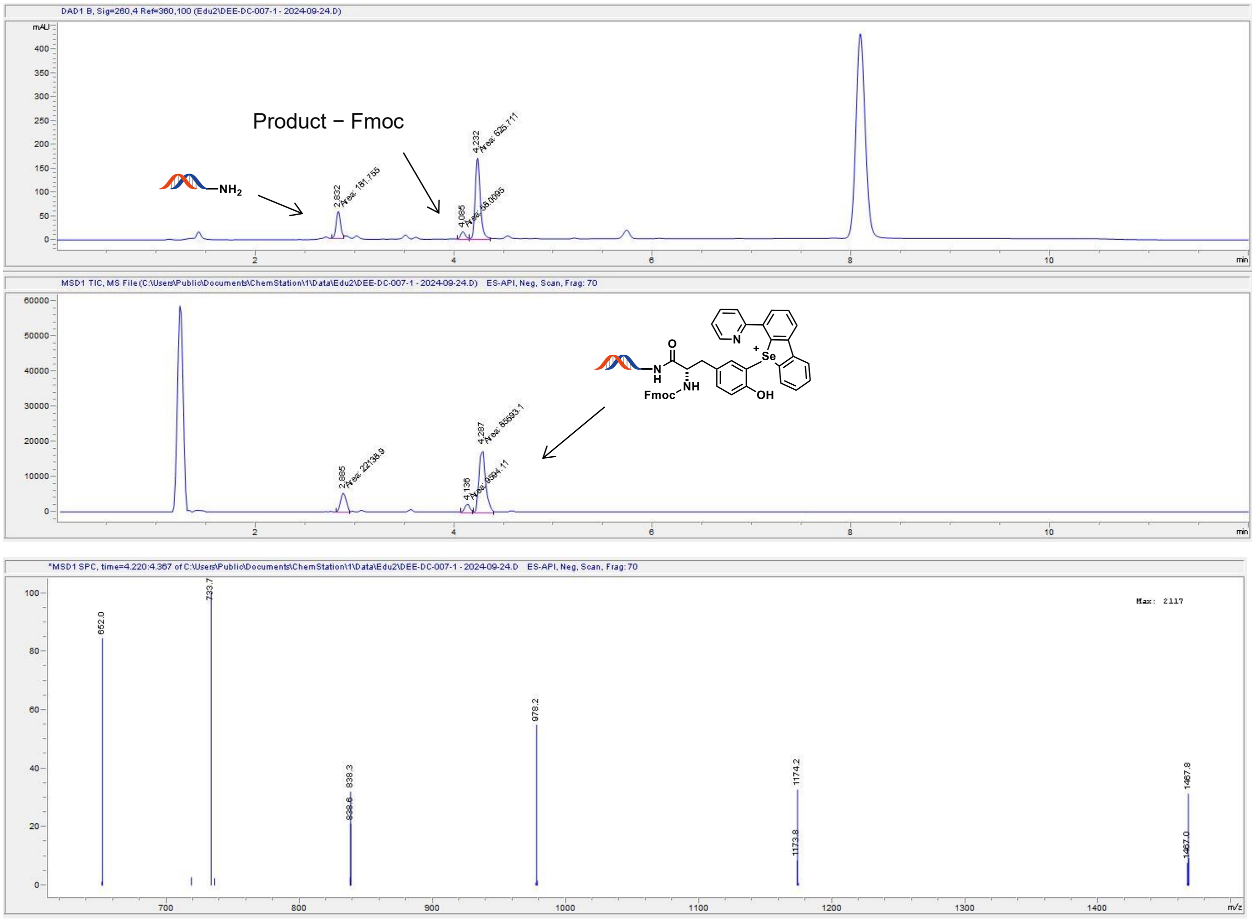

Figure S82. Analytical HPLC trace of **30** with HPLC Method A. (Up) DAD chromatogram at 260 nm. (Middle) TIC chromatogram. (Below) Ionization of peak at 4.287 min containing reaction product.

Synthesis of DNA-conjugated selenonium salt 31

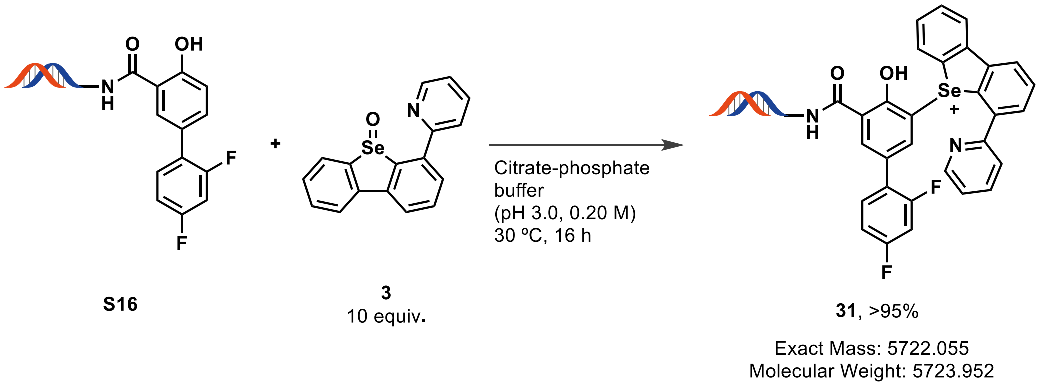

At 20–25 °C, 1.0 µL of **S16** (2.0 mM, 2.0 nmol, 1.0 equiv.) in water was added to a 1.5 mL Eppendorf tube. Next, 4.0 µL of Citrate-phosphate buffer (pH 3.0, c = 500 mM) was added. Then, 5.0 µL of a selenoxide **3** stock solution (4.0 mM, 20 nmol, 10 equiv.) in water was added. The mixture was vortexed for 5 seconds, transferred into a Thermocycler pre-heated at 30 °C, and incubated at 30 °C for 16 hours at 600 rpm. After 16 hours, reaction was quenched by addition of 10 µL of borate buffer (pH 9.4, c = 500 mM), and an aliquot of 2 µL of the reaction mixture was diluted to 40 µL with water for LC–MS analysis.

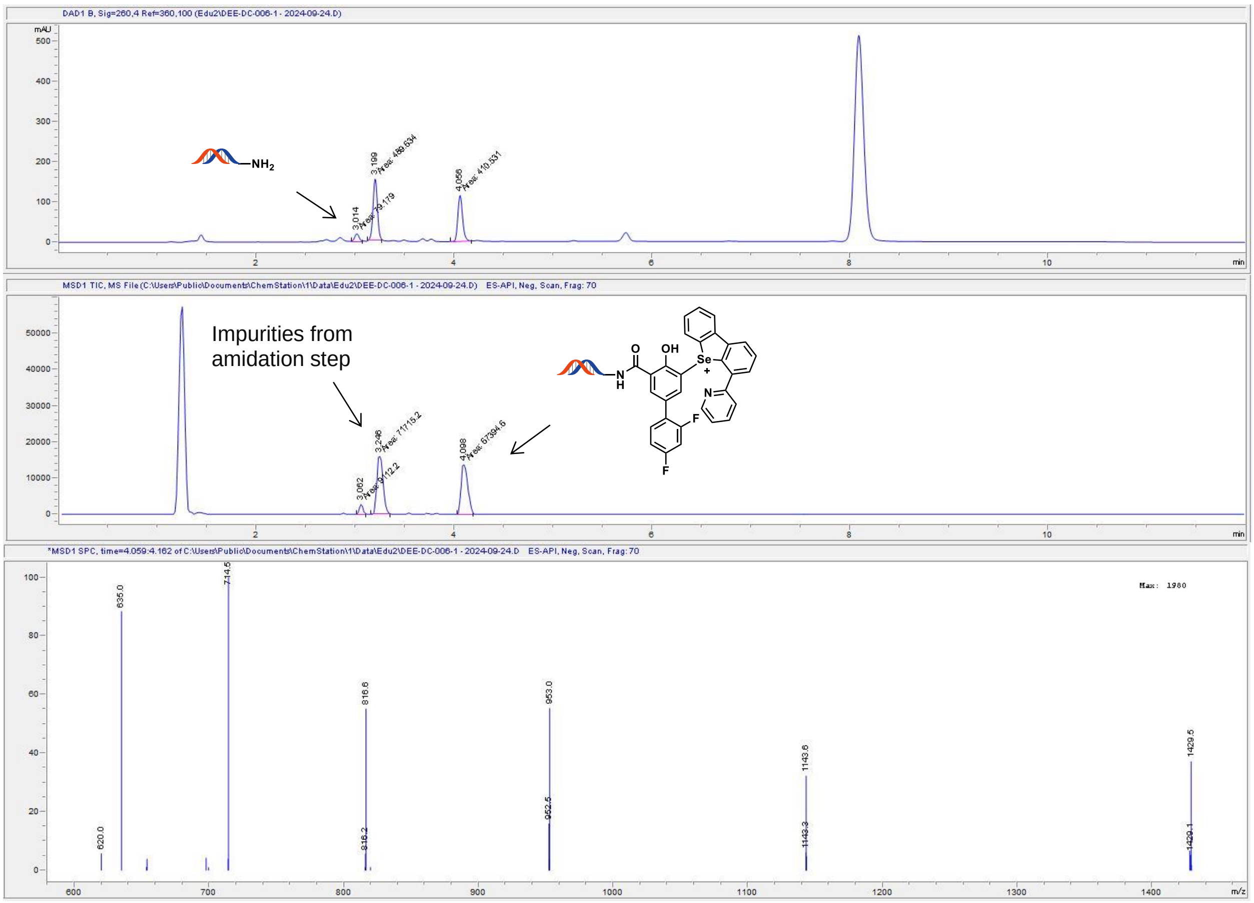

Figure S83. Analytical HPLC trace of **31** with HPLC Method A. (Up) DAD chromatogram at 260 nm. (Middle) TIC chromatogram. (Below) Ionization of peak at 4.098 min containing reaction product.

Synthesis of DNA-conjugated selenonium salt 32

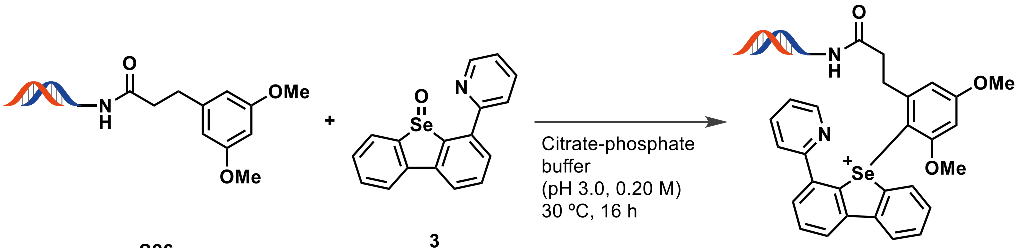

S26

3
10 equiv.

32, >95%
Exact Mass: 5682.100
Molecular Weight: 5683.981

At 20–25 °C, 1.0 µL of **S26** (2.0 mM, 2.0 nmol, 1.0 equiv.) in water was added to a 1.5 mL Eppendorf tube. Next, 4.0 µL of Citrate-phosphate buffer (pH 3.0, c = 500 mM) was added. Then, 5.0 µL of a selenoxide **3** stock solution (4.0 mM, 20 nmol, 10 equiv.) in water was added. The mixture was vortexed for 5 seconds, transferred into a Thermocycler pre-heated at 30 °C, and incubated at 30 °C for 16 hours at 600 rpm. After 16 hours, reaction was quenched by addition of 10 µL of borate buffer (pH 9.4, c = 500 mM), and an aliquot of 2 µL of the reaction mixture was diluted to 40 µL with water for LC–MS analysis.

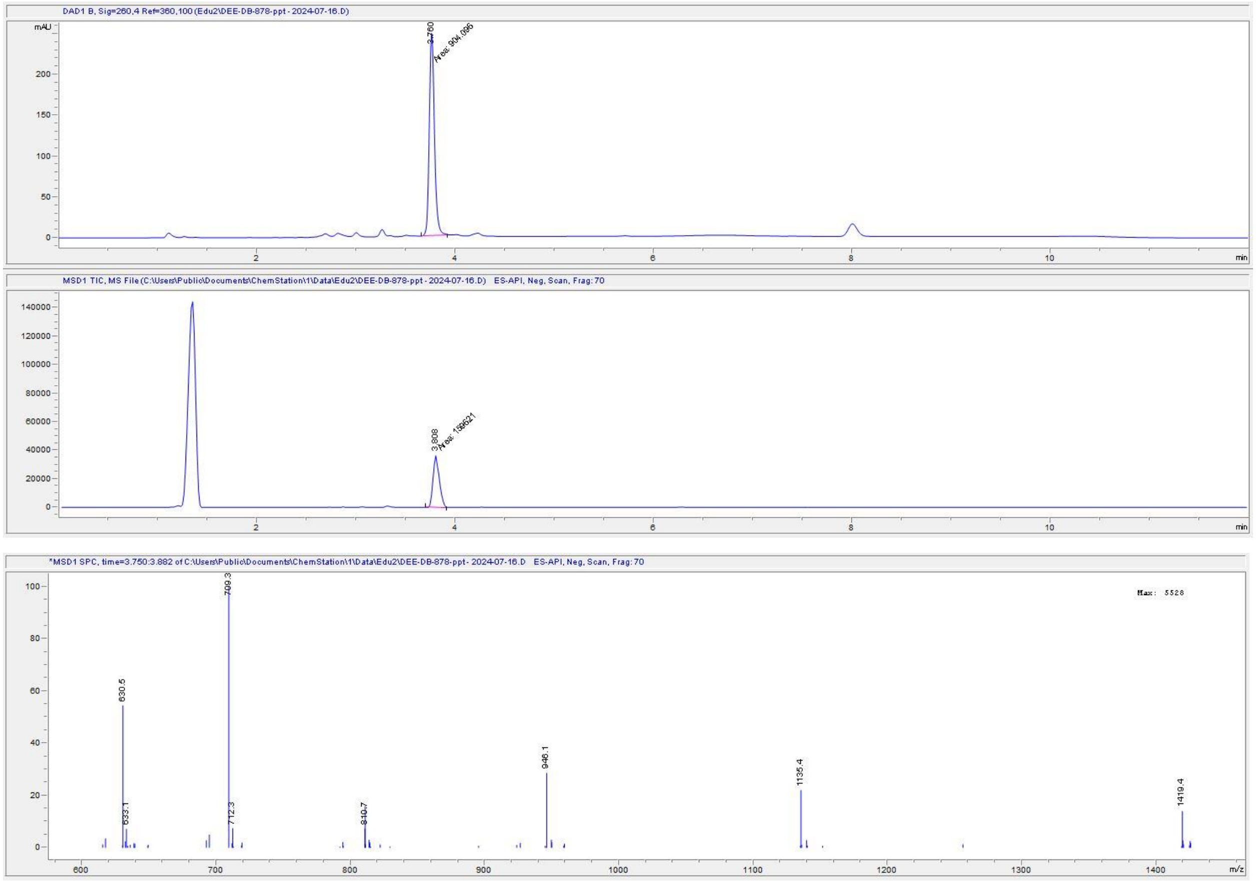

Figure S84. Analytical HPLC trace of **32** with HPLC Method A. (Up) DAD chromatogram at 260 nm. (Middle) TIC chromatogram. (Below) Ionization of peak at 3.808 min containing reaction product.

Synthesis of DNA-conjugated selenonium salt 33

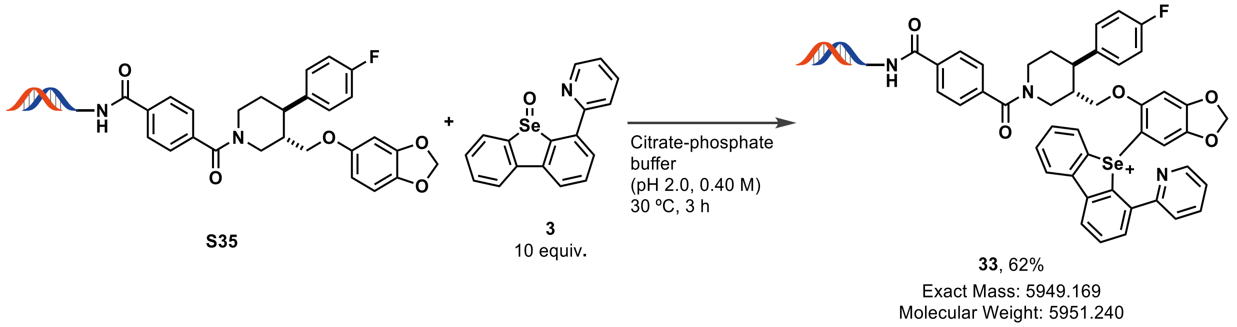

At 20–25 °C, 1.0 µL of **S35** (2.0 mM, 2.0 nmol, 1.0 equiv.) in water was added to a 1.5 mL Eppendorf tube. Next, 4.0 µL of Citrate-phosphate buffer (pH 2.0, c = 1.0 M) was added. Then, 5.0 µL of a selenoxide **3** stock solution (4.0 mM, 20 nmol, 10 equiv.) in water was added. The mixture was vortexed for 5 seconds, transferred into a Thermocycler pre-heated at 30 °C, and incubated at 30 °C for 3 hours at 600 rpm. After 3 hours, reaction was quenched by addition of 10 µL of borate buffer (pH 9.4, c = 500 mM), and an aliquot of 2 µL of the reaction mixture was diluted to 40 µL with water for LC–MS analysis.

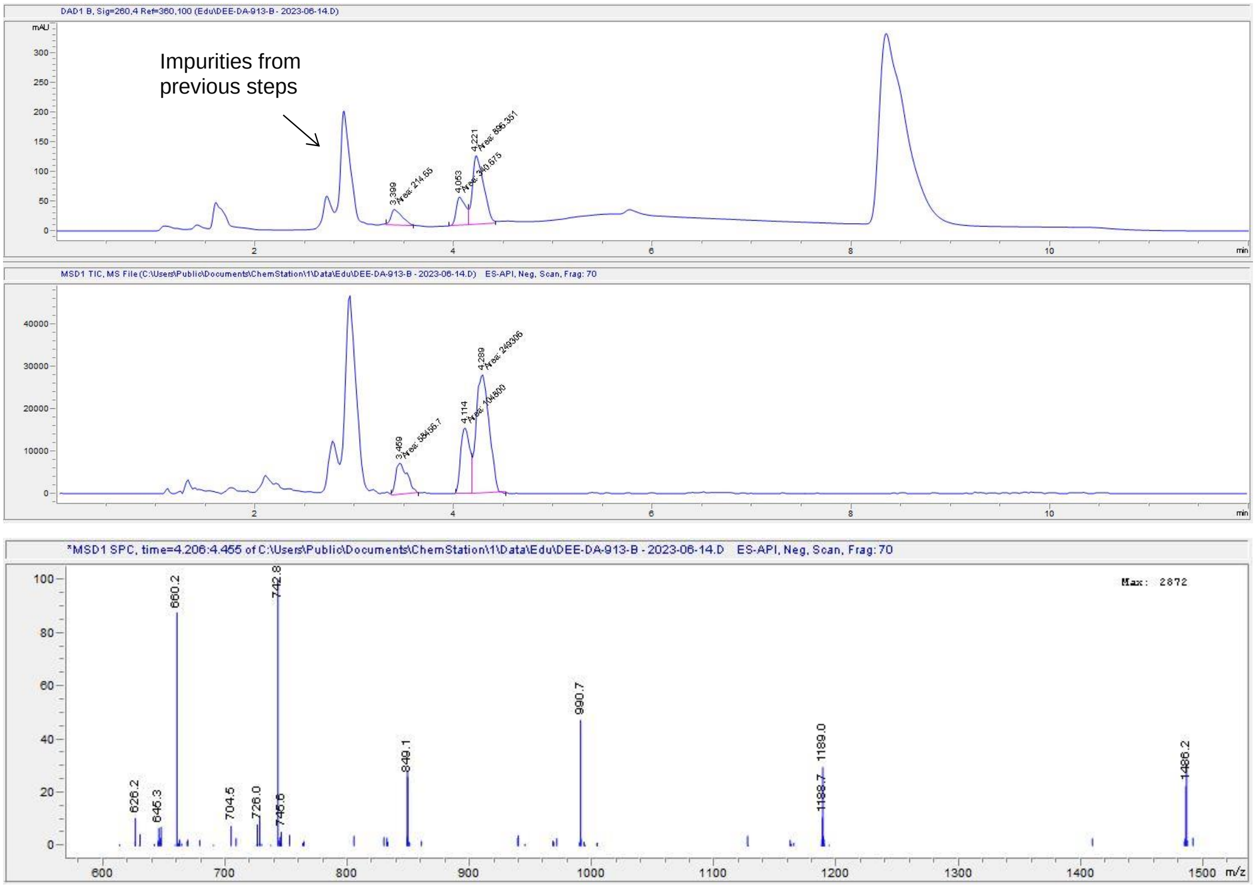

Figure S85. Analytical HPLC trace of **33** with HPLC Method A. (Up) DAD chromatogram at 260 nm. (Middle) TIC chromatogram. (Below) Ionization of peak at 4.289 min containing reaction product.

Synthesis of DNA-conjugated selenonium salt 34

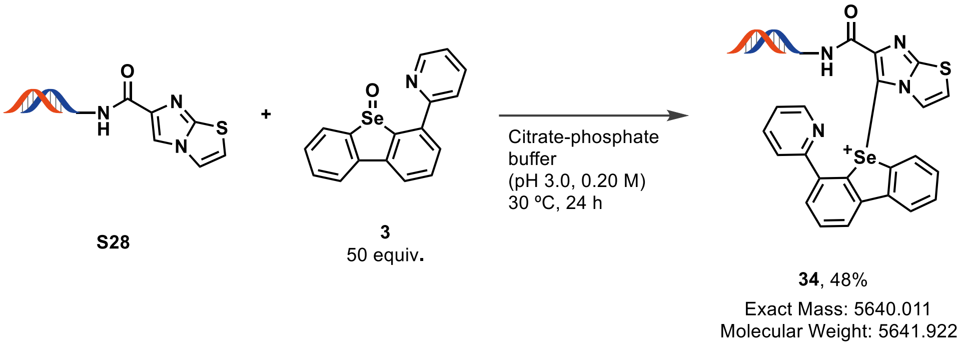

At 20–25 °C, 1.0 µL of **S28** (2.0 mM, 2.0 nmol, 1.0 equiv.) in water was added to a 1.5 mL Eppendorf tube. Next, 9.0 µL of Citrate-phosphate buffer (pH 3.0, c = 500 mM) was added. Then, 10.0 µL of a selenoxide **3** stock solution (10.0 mM, 20 nmol, 50 equiv.) in water was added. The mixture was vortexed for 5 seconds, transferred into a Thermocycler pre-heated at 30 °C, and incubated at 30 °C for 24 hours at 600 rpm. After 24 hours, reaction was quenched by addition of 20 µL of borate buffer (pH 9.4, c = 500 mM), and an aliquot of 2 µL of the reaction mixture was diluted to 40 µL with water for LC–MS analysis.

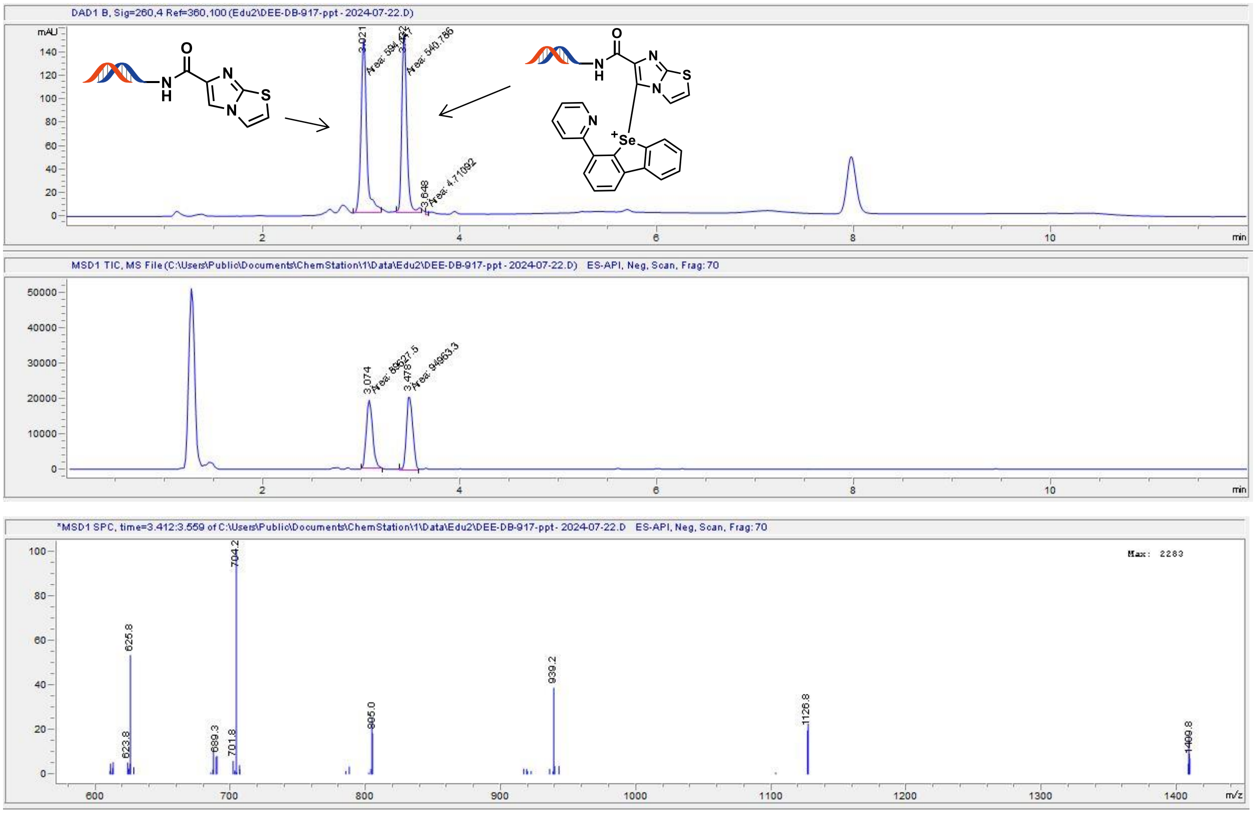

Figure S86. Analytical HPLC trace of **34** with HPLC Method A. (Up) DAD chromatogram at 260 nm. (Middle) TIC chromatogram. (Below) Ionization of peak at 3.478 min containing reaction product.

Synthesis of DNA-conjugated selenonium salt 35

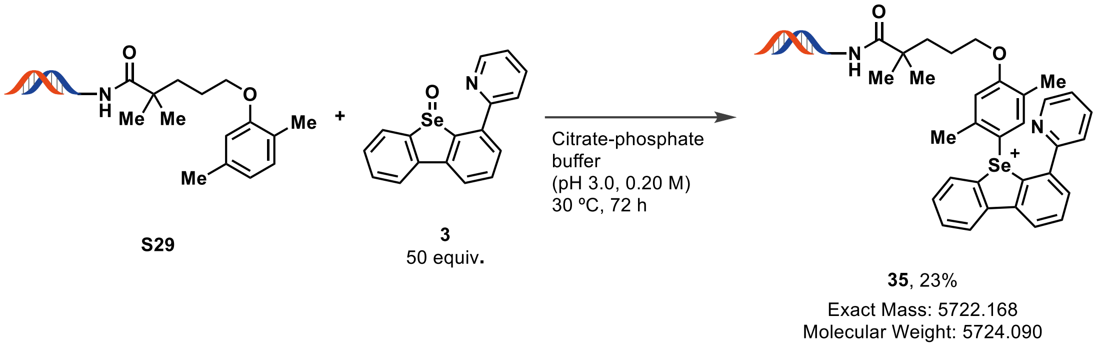

At 20–25 °C, 1.0 µL of **S26** (2.0 mM, 2.0 nmol, 1.0 equiv.) in water was added to a 1.5 mL Eppendorf tube. Next, 9.0 µL of Citrate-phosphate buffer (pH 3.0, c = 500 mM) was added. Then, 10.0 µL of a selenoxide **3** stock solution (10.0 mM, 20 nmol, 50 equiv.) in water was added. The mixture was vortexed for 5 seconds, transferred into a Thermocycler pre-heated at 30 °C, and incubated at 30 °C for 24 hours at 600 rpm. After 72 hours, reaction was quenched by addition of 20 µL of borate buffer (pH 9.4, c = 500 mM), and an aliquot of 2 µL of the reaction mixture was diluted to 40 µL with water for LC–MS analysis.

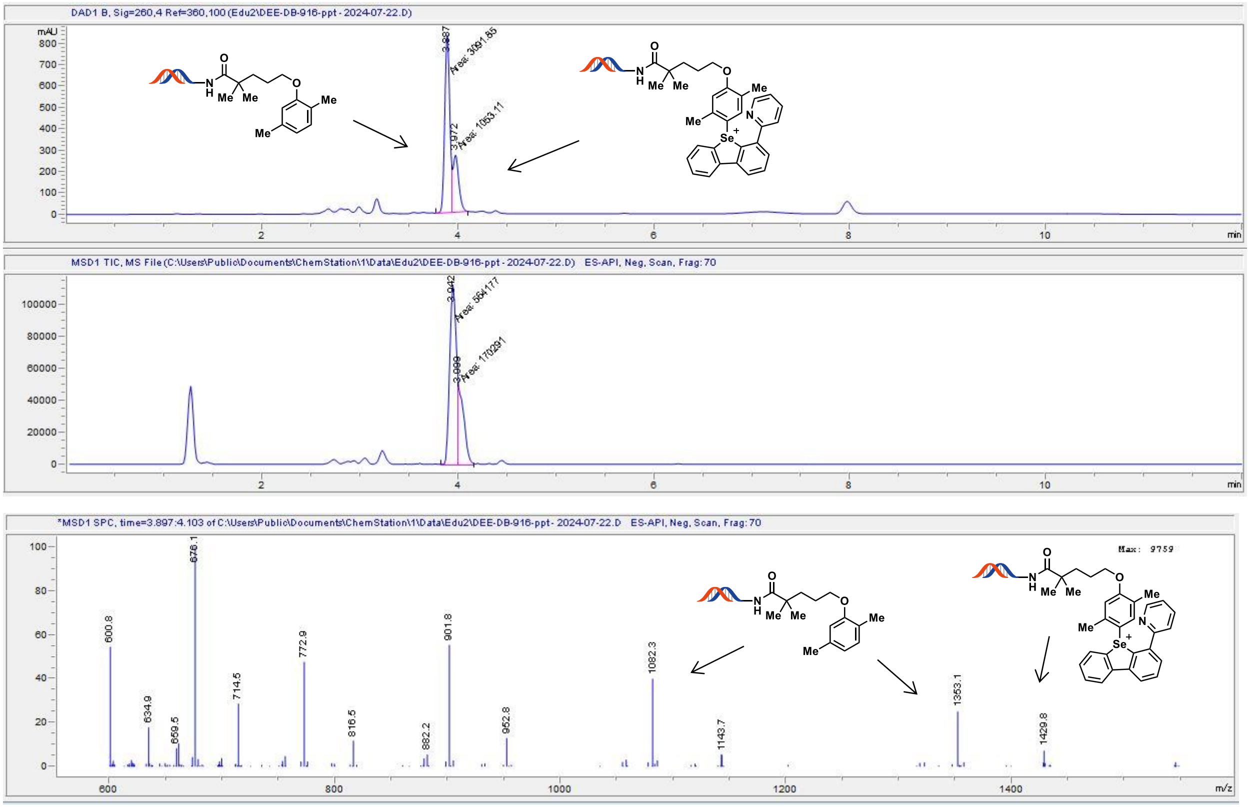

Figure S87. Analytical HPLC trace of **35** with HPLC Method A. (Up) DAD chromatogram at 260 nm. (Middle) TIC chromatogram. (Below) Ionization of peak at 3.942 min containing reaction product.

Synthesis of DNA-conjugated selenonium salt 36

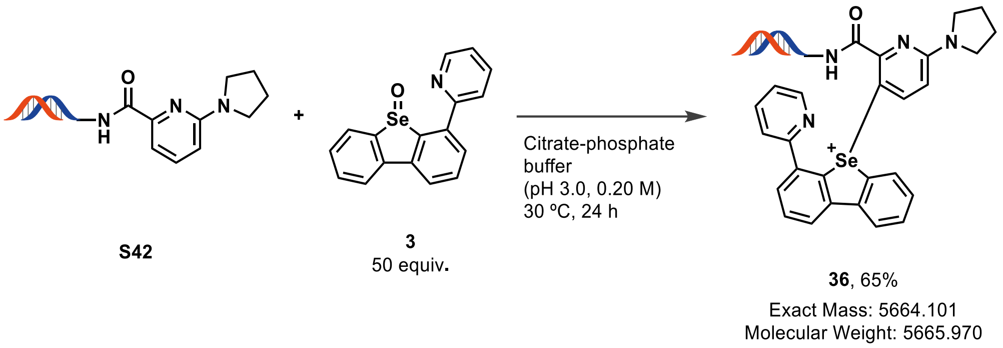

At 20–25 °C, 1.0 µL of **S42** (2.0 mM, 2.0 nmol, 1.0 equiv.) in water was added to a 1.5 mL Eppendorf tube. Next, 9.0 µL of Citrate-phosphate buffer (pH 3.0, c = 500 mM) was added. Then, 10.0 µL of a selenoxide **3** stock solution (10.0 mM, 20 nmol, 50 equiv.) in water was added. The mixture was vortexed for 5 seconds, transferred into a Thermocycler pre-heated at 30 °C, and incubated at 30 °C for 24 hours at 600 rpm. After 24 hours, reaction was quenched by addition of 20 µL of borate buffer (pH 9.4, c = 500 mM), and an aliquot of 2 µL of the reaction mixture was diluted to 40 µL with water for LC–MS analysis.

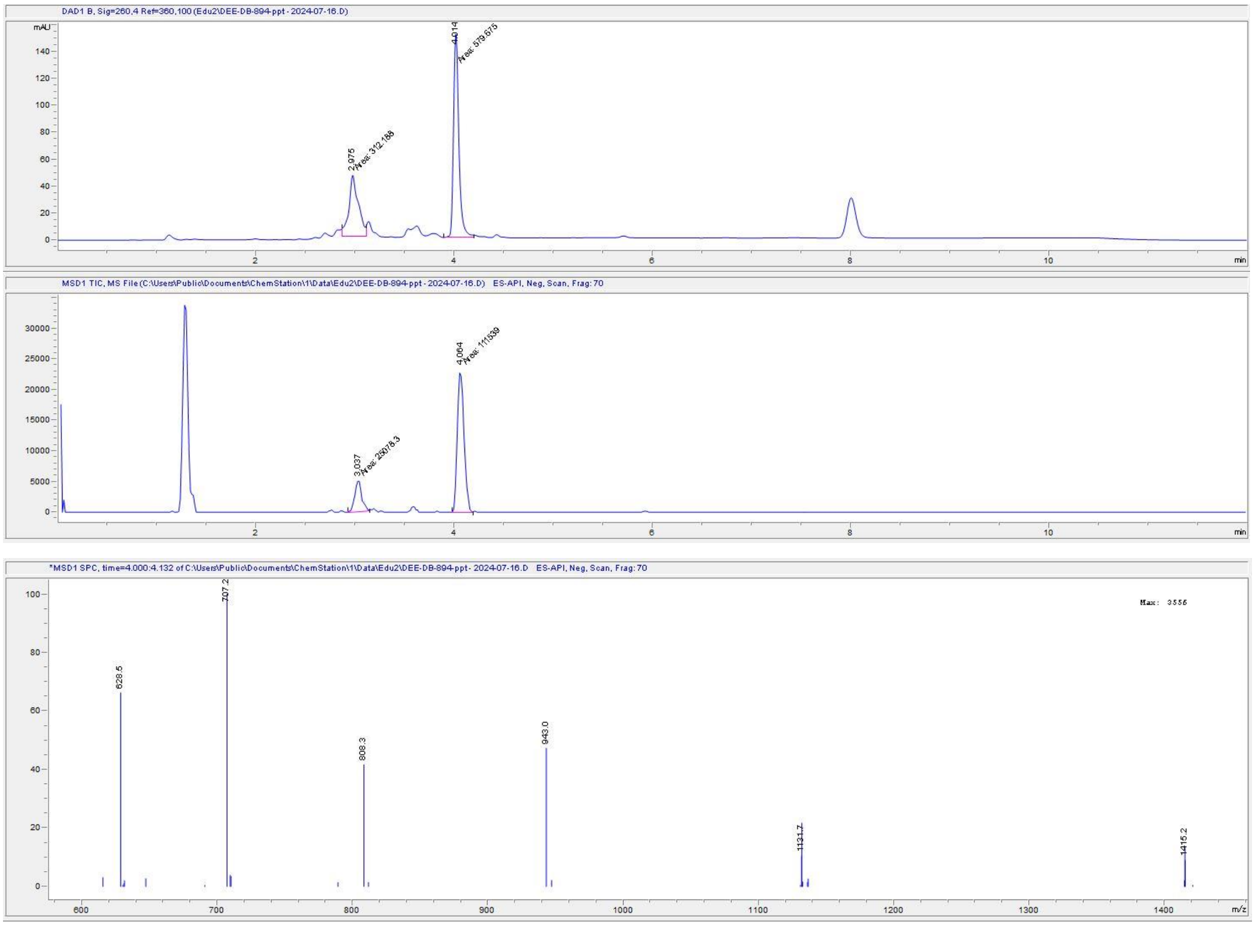

Figure S88. Analytical HPLC trace of **32** with HPLC Method A. (Up) DAD chromatogram at 260 nm. (Middle) TIC chromatogram. (Below) Ionization of peak at 4.064 min containing reaction product.

Synthesis of DNA-conjugated selenonium salt 37

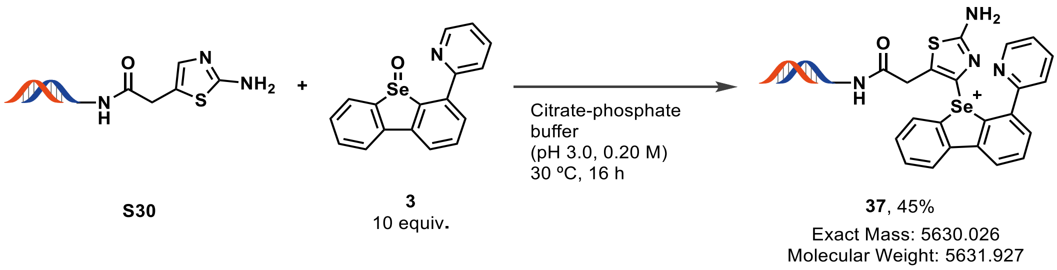

At 20–25 °C, 1.0 µL of **S30** (2.0 mM, 2.0 nmol, 1.0 equiv.) in water was added to a 1.5 mL Eppendorf tube. Next, 4.0 µL of Citrate-phosphate buffer (pH 3.0, c = 500 mM) was added. Then, 5.0 µL of a selenoxide **3** stock solution (4.0 mM, 20 nmol, 10 equiv.) in water was added. The mixture was vortexed for 5 seconds, transferred into a Thermocycler pre-heated at 30 °C, and incubated at 30 °C for 16 hours at 600 rpm. After 16 hours, reaction was quenched by addition of 10 µL of borate buffer (pH 9.4, c = 500 mM), and an aliquot of 2 µL of the reaction mixture was diluted to 40 µL with water for LC–MS analysis.

Oxidation byproducts refer to unidentified byproducts with a MW of **S30**+16 and **S30**+32.

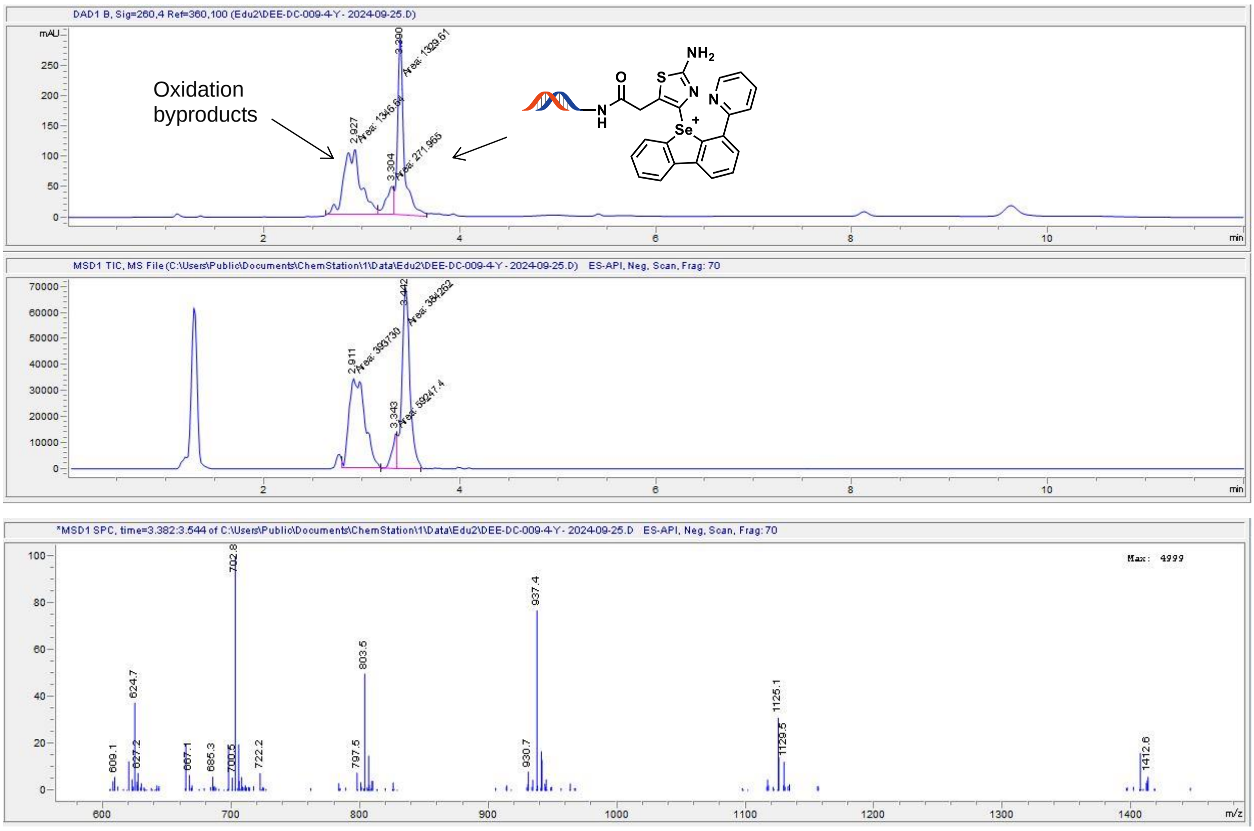

Figure S89. Analytical HPLC trace of **37** with HPLC Method A. (Up) DAD chromatogram at 260 nm. (Middle) TIC chromatogram. (Below) Ionization of peak at 3.442 min containing reaction product.

Synthesis of DNA-conjugated selenonium salt 38

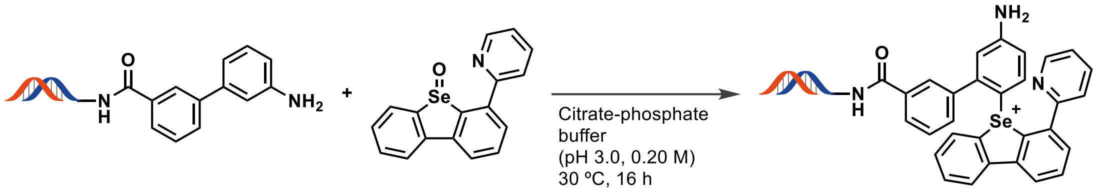

At 20–25 °C, 1.0 µL of **S45** (2.0 mM, 2.0 nmol, 1.0 equiv.) in water was added to a 1.5 mL Eppendorf tube. Next, 4.0 µL of Citrate-phosphate buffer (pH 3.0, c = 500 mM) was added. Then, 5.0 µL of a selenoxide **3** stock solution (4.0 mM, 20 nmol, 10 equiv.) in water was added. The mixture was vortexed for 5 seconds, transferred into a Thermocycler pre-heated at 30 °C, and incubated at 30 °C for 16 hours at 600 rpm. After 16 hours, reaction was quenched by addition of 10 µL of borate buffer (pH 9.4, c = 500 mM), and an aliquot of 2 µL of the reaction mixture was diluted to 40 µL with water for LC–MS analysis.

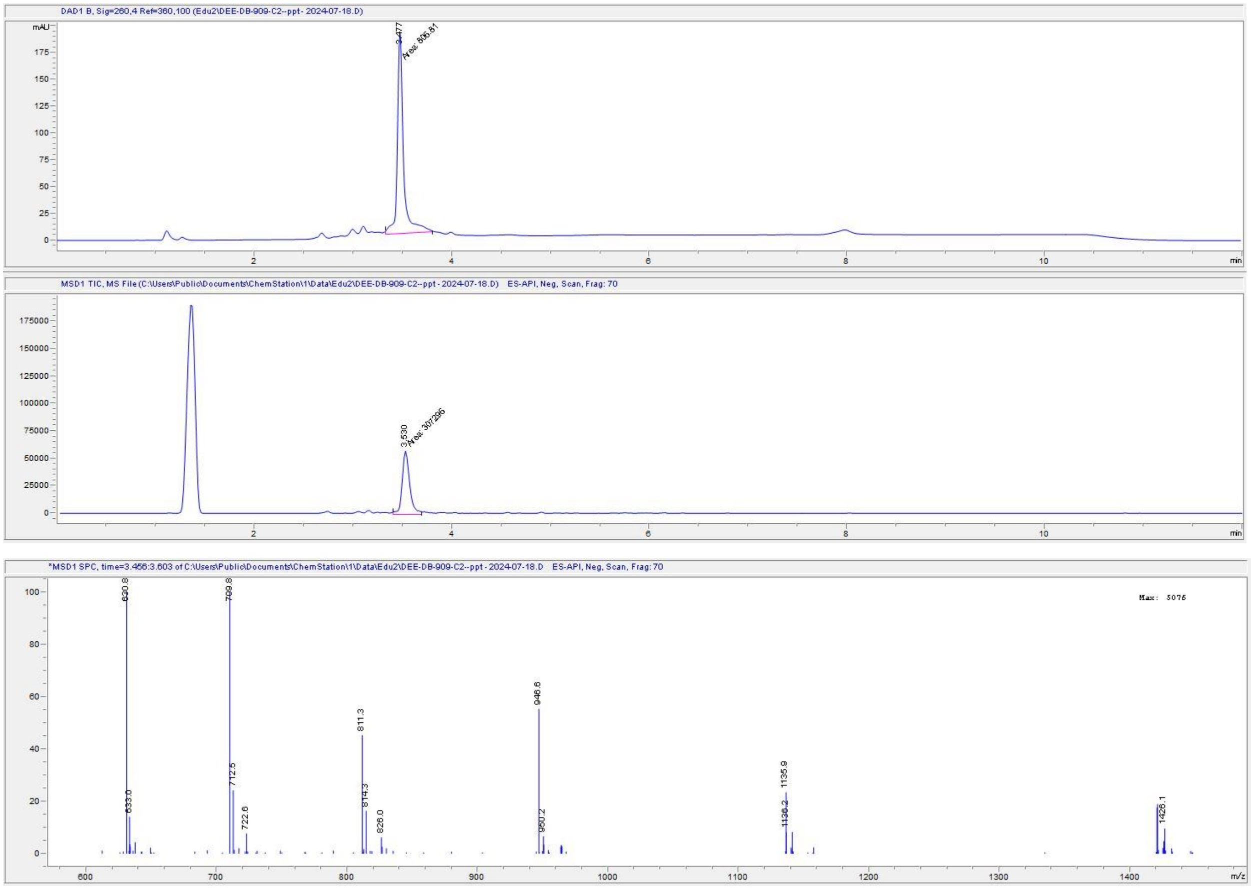

Figure S90. Analytical HPLC trace of **38** with HPLC Method A. (Up) DAD chromatogram at 260 nm. (Middle) TIC chromatogram. (Below) Ionization of peak at 3.530 min containing reaction product.

Failed DNA-conjugated substrates for C−H functionalization

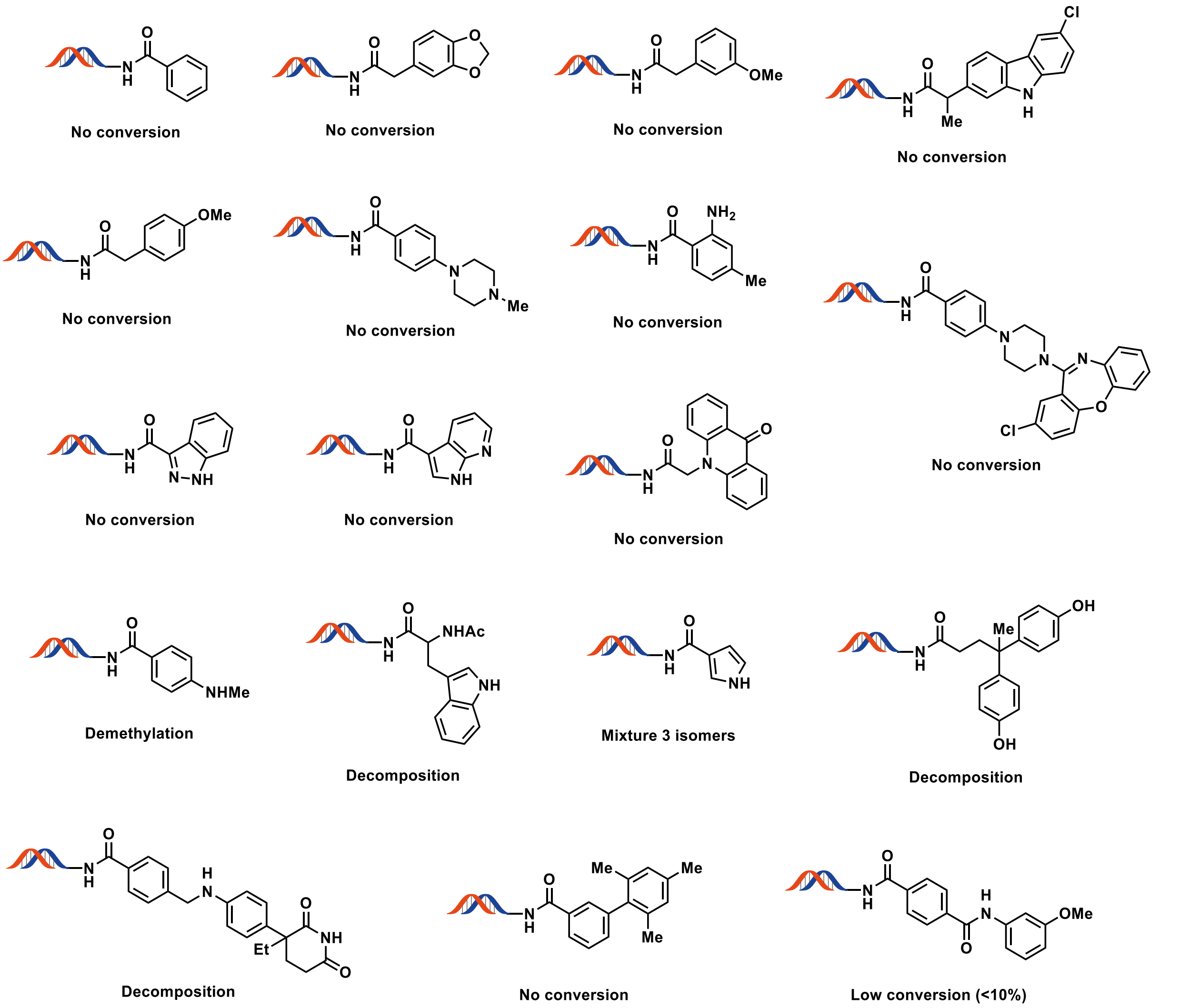

Figure S91. Failed on-DNA substrates

DNA-conjugated functionalization of selenonium salts

Iodination of DNA-conjugated selenonium salt 24

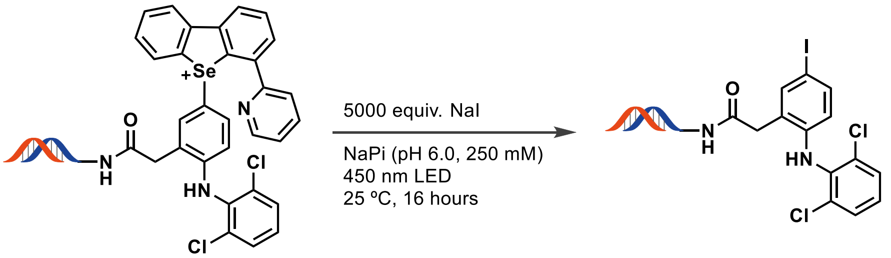

24

39, 86%

Exact Mass: 5584.927
Molecular Weight: 5588.550

At 20–25 °C, 5.0 µL of **24** (2.0 mM, 10.0 nmol, 1.0 equiv.) in water was added to a 1.5 mL Eppendorf tube. Next, 25.0 µL of a NaI stock solution (2.00 M, 50 µmol, 5000 equiv.) in phosphate buffer (NaP$_i$, pH 6.0, 300 mM) was added. A light stream of argon was blown over the solution for 30 seconds. The mixture was vortexed for 5 seconds, transferred into a Penn PhD Photoreactor M2, and irradiated at 450 nm for 16 hours at 25 °C.

After 15 minutes, tover the sample, 5 µL of a 5 M solution of NaCl in water and 150 µL of ethanol at −20 °C were added to precipitate the DNA conjugate. The Eppendorf tube was placed in the freezer (−20 °C) for at least 1 hour, and then it was centrifuged at 4 °C and 10000 x g for at least 30 minutes. The supernatant was removed, and the pellet was redissolved in 200 µL of water for LC–MS analysis.

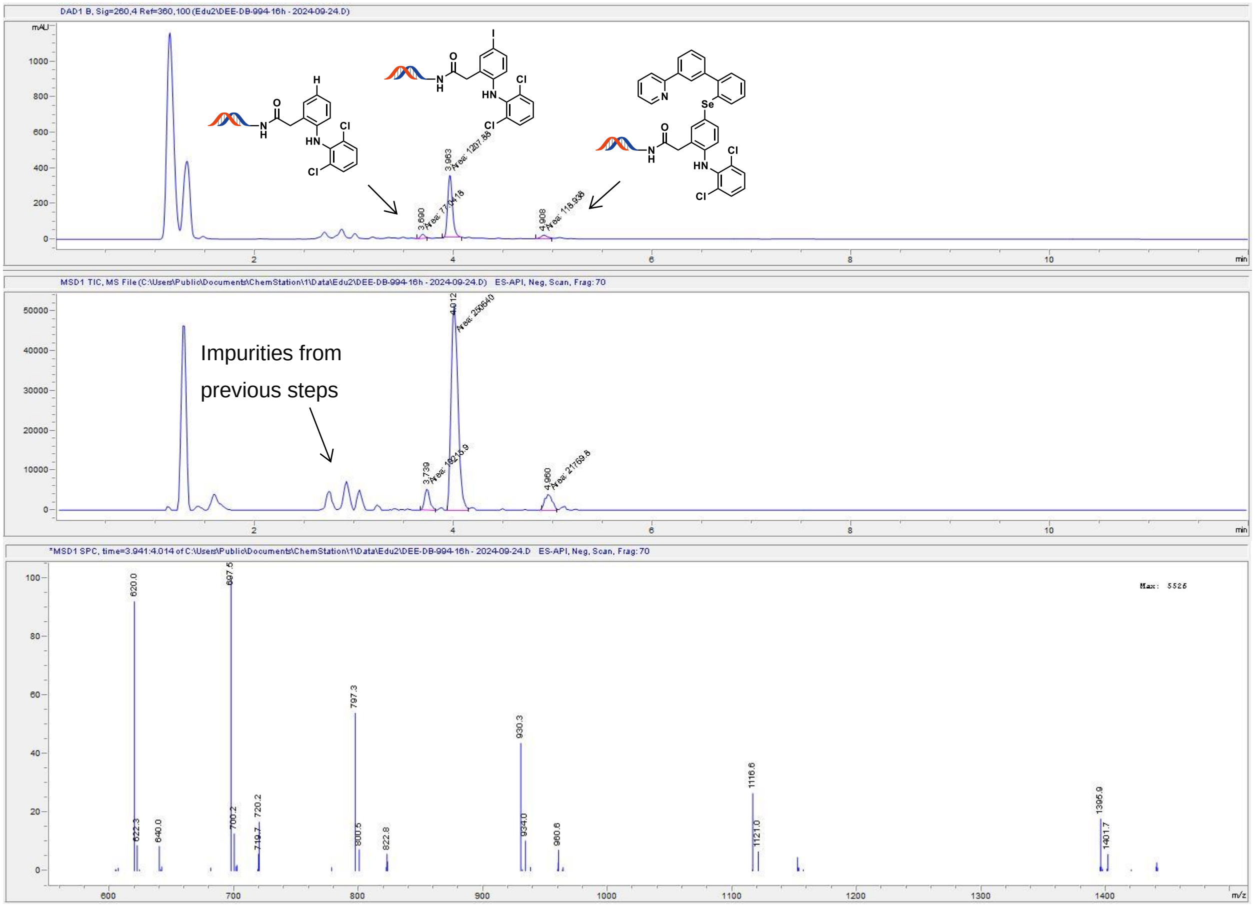

Figure S92. Analytical HPLC trace of **39** with HPLC Method A. (Up) DAD chromatogram at 260 nm. (Middle) TIC chromatogram. (Below) Ionization of peak at 4.012 min containing reaction product.

Suzuki coupling of DNA-conjugated selenonium salt 24 with phenylboronic acid

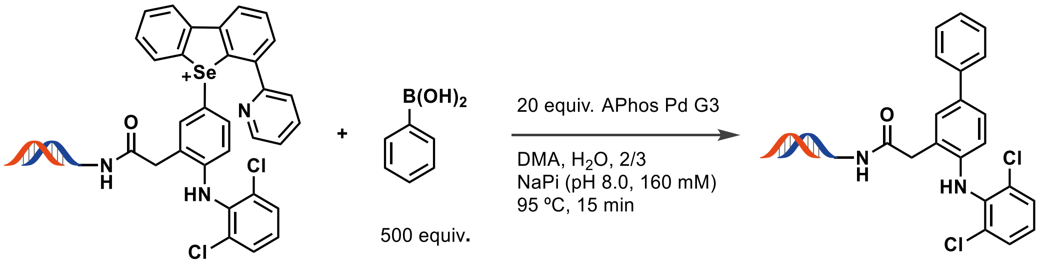

24

40, 80%

Exact Mass: 5535.061
Molecular Weight: 5538.751

At 20–25 °C, 2.0 µL of **24** (1.0 mM, 2.0 nmol, 1.0 equiv.) in water was added to a 1.5 mL Eppendorf tube. Next, 4.0 µL of phosphate buffer (NaPi, pH 8.0, 500 mM) was added. 2.0 µL of an APhos Pd G3 stock solution (20 mM, 20 nmol, 20 equiv.) in DMA was added over the solution of **24**. The mixture was vortexed for 5 seconds. Lastly, 2.0 µL of a phenylboronic acid stock solution (500 mM, 1000 nmol, 500 equiv.) in DMA was added. The mixture was vortexed for 5 seconds, transferred into a Thermocycler pre-heated at 95 °C, and incubated at 95 °C for 15 min. at 600 rpm.

After 15 minutes, 10 µL of a 100 mM solution of sodium diethyldithiocarbamate trihydrate in water were added to remove the palladium salts from the solution. The reaction mixture was centrifuged and an aliquot of 8 µL of the supernatant was diluted to 40 µL with water. Over the sample, 5 µL of a 5 M solution of NaCl in water and 150 µL of ethanol at −20 °C were added to precipitate the DNA conjugate. The Eppendorf tube was placed in the freezer (−20 °C) for at least 1 hour, and then it was centrifuged at 4 °C and 10000 x g for at least 30 minutes. The supernatant was removed, and the pellet was redissolved in 50 µL of water for LC–MS analysis.

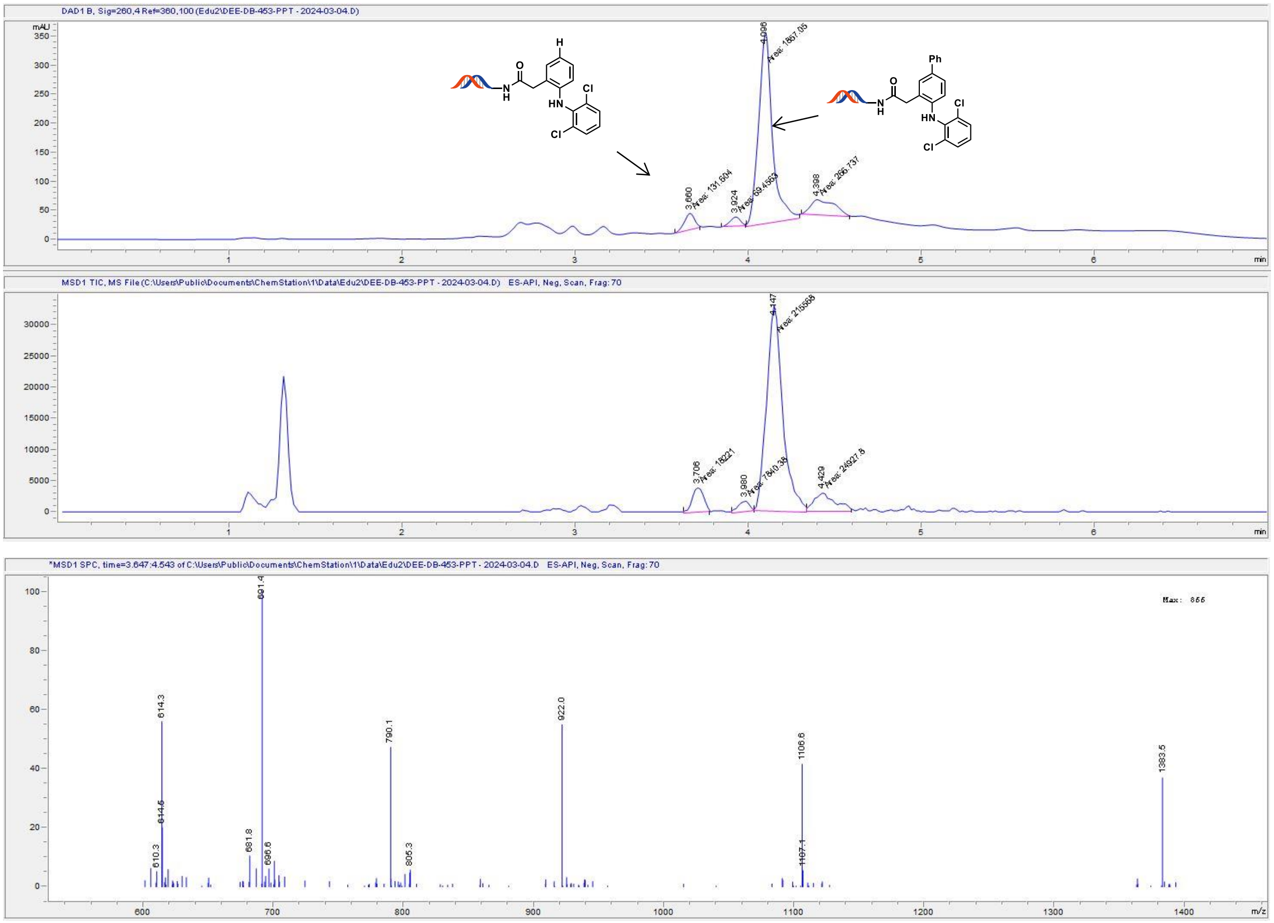

Figure S93. Analytical HPLC trace of **40** with HPLC Method B. (Up) DAD chromatogram at 260 nm. (Middle) TIC chromatogram. (Below) Ionization of peak at 4.147 min containing reaction product.

Suzuki coupling of DNA-conjugated selenonium salt 24 with trimethylboroxine

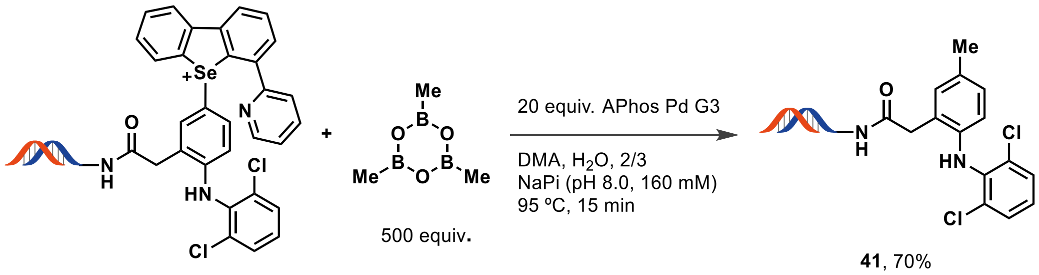

24

41, 70%
Exact Mass: 5473.046
Molecular Weight: 5476.680

At 20–25 °C, 2.0 µL of **24** (1.0 mM, 2.0 nmol, 1.0 equiv.) in water was added to a 1.5 mL Eppendorf tube. Next, 4.0 µL of phosphate buffer (NaP_i, pH 8.0, 500 mM) was added. 2.0 µL of an APhos Pd G3 stock solution (20 mM, 40 nmol, 20 equiv.) in DMA was added over the solution of **24.** The mixture was

vortexed for 5 seconds. Lastly, 2.0 µL of a trimethylboroxine stock solution (500 mM, 1000 nmol, 500 equiv.) in DMA was added. The mixture was vortexed for 5 seconds, transferred into a Thermocycler pre-heated at 95 °C, and incubated at 95 °C for 15 min. at 600 rpm.

After 15 minutes, 10 µL of a solution 100 mM of sodium diethyldithiocarbamate trihydrate in water were added to remove the palladium salts from the solution. Reaction mixture was centrifuged and an aliquot of 8 µL of the supernatant was diluted to 40 µL with water. Over the sample, 5 µL of a 5 M solution of NaCl in water and 150 µL of ethanol at −20 °C were added to precipitate the DNA conjugate. The Eppendorf tube was placed in the freezer (−20 °C) for at least 1 hour, and then it was centrifuged at 4 °C and 10000 x g for at least 30 minutes. The supernatant was removed, and the pellet was redissolved in 50 µL of water for LC–MS analysis.

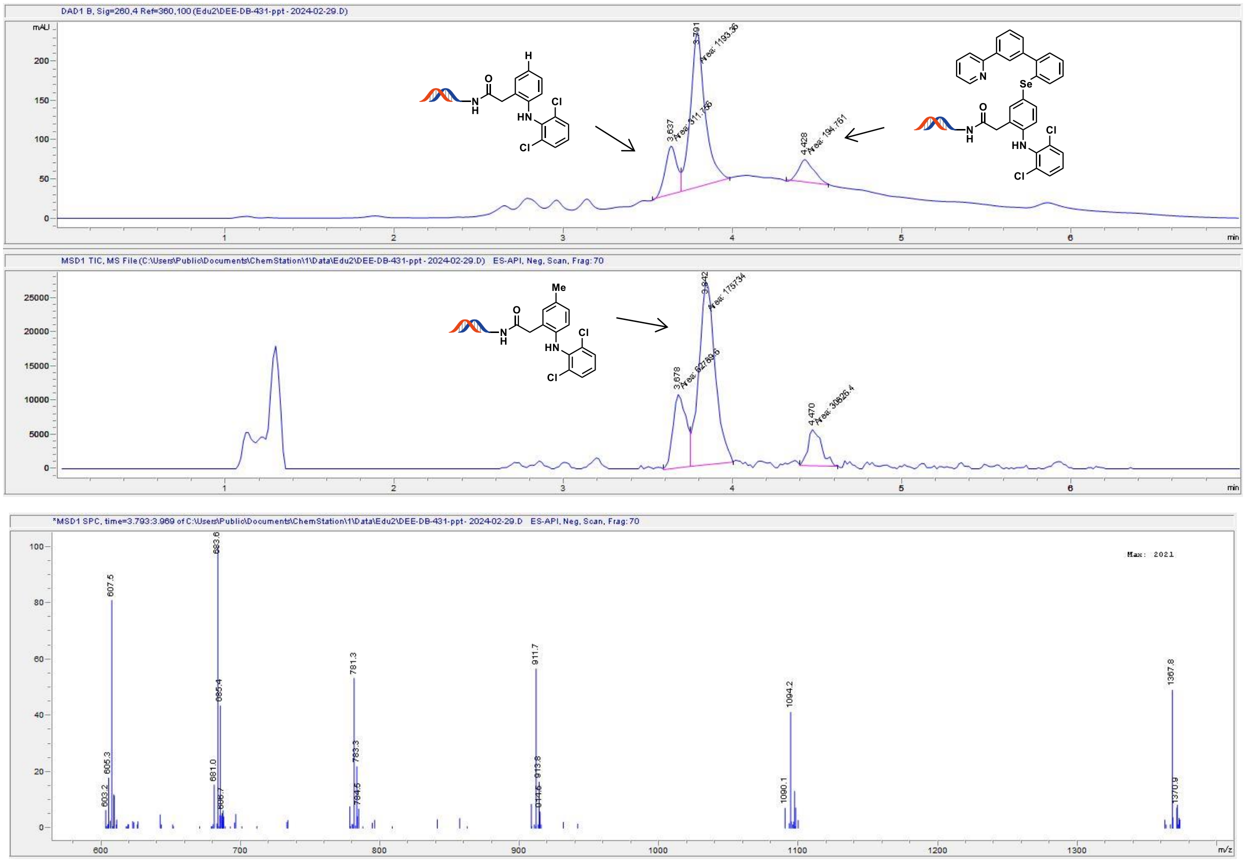

Figure S94. Analytical HPLC trace of **41** with HPLC Method B. (Up) DAD chromatogram at 260 nm. (Middle) TIC chromatogram. (Below) Ionization of peak at 3.842 min containing reaction product.

Hydroxycarbonylation of DNA-conjugated selenonium salt 24

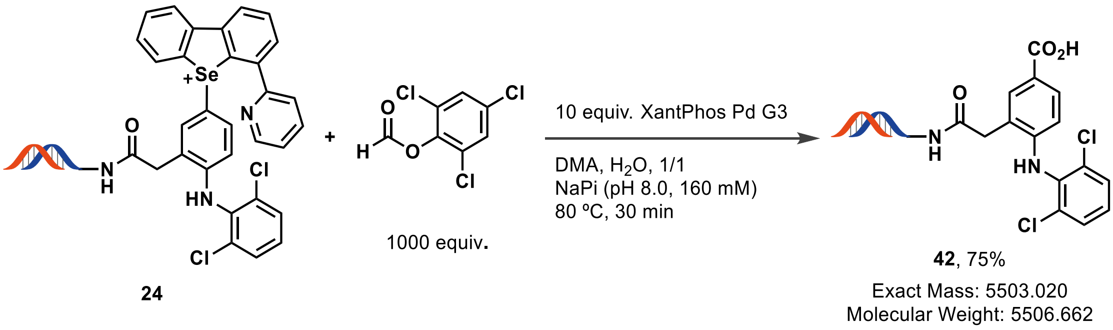

1000 equiv.

24

42, 75%

Exact Mass: 5503.020
Molecular Weight: 5506.662

At 20–25 °C, 2.0 µL of **24** (1.0 mM, 2.0 nmol, 1.0 equiv.) in water was added to a 1.5 mL Eppendorf tube.

Next, 4.0 µL of phosphate buffer (NaP$_i$, pH 8.0, 500 mM) was added. 2.0 µL of a XantPhos Pd G3 stock solution (10 mM, 20 nmol, 10 equiv.) in DMA was added over the solution of **24**. The mixture was vortexed for 5 seconds. Lastly, 4.0 µL of a trichlorophenyl formate stock solution (500 mM, 1000 nmol, 1000 equiv.) in DMA was added. The mixture was vortexed for 5 seconds, transferred into a Thermocycler pre-heated at 80 °C, and incubated at 80 °C for 30 min. at 600 rpm.

After 15 minutes, 10 µL of a solution 100 mM of sodium diethyldithiocarbamate trihydrate in water were added to remove the palladium salts from the solution. Reaction mixture was centrifuged and an aliquot of 8 µL of the supernatant was diluted to 40 µL with water. Over the sample, 5 µL of a 5 M solution of NaCl in water and 150 µL of ethanol at −20 °C were added to precipitate the DNA conjugate. The Eppendorf tube was placed in the freezer (−20 °C) for at least 1 hour, and then it was centrifuged at 4 °C and 10000 x g for at least 30 minutes. The supernatant was removed, and the pellet was redissolved in 50 µL of water for LC–MS analysis. *Conversion was calculated using the TIC chromatogram.*

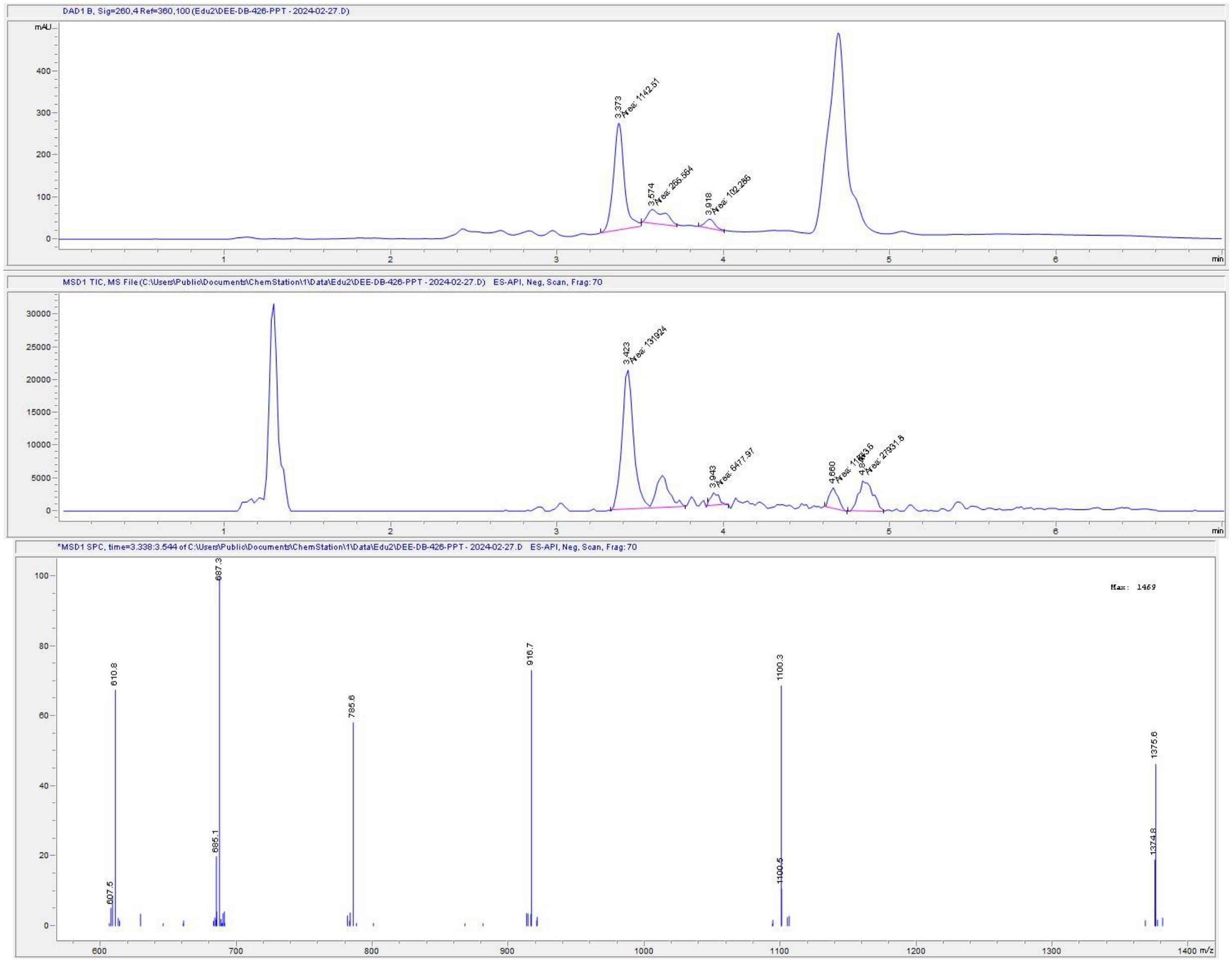

Figure S95. Analytical HPLC trace of **42** with HPLC Method B. (Up) DAD chromatogram at 260 nm. (Middle) TIC chromatogram. (Below) Ionization of peak at 3.423 min containing reaction product. Conversion was calculated using the TIC chromatogram.

Cyanation of DNA-conjugated selenonium salt 24

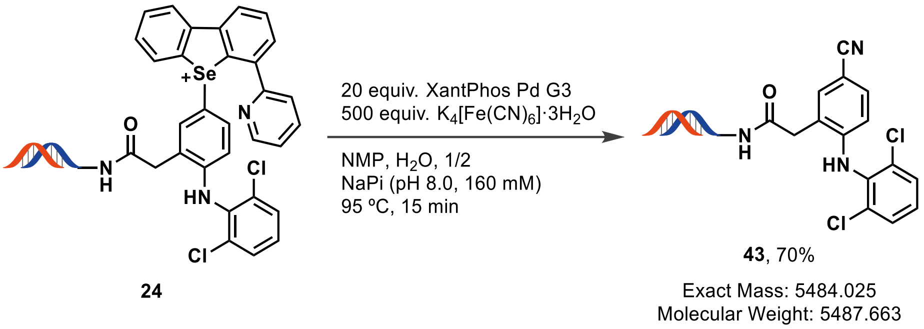

At 20–25 °C, 2.0 µL of **24** (1.0 mM, 2.0 nmol, 1.0 equiv.) in water was added to a 1.5 mL Eppendorf tube. Next, 4.0 µL of phosphate buffer (NaPi, pH 8.0, 500 mM) was added. 4.0 µL of a XantPhos Pd G3 stock solution (10 mM, 40 nmol, 20 equiv.) in NMP was added over the solution of **24**. The mixture was vortexed for 5 seconds. Lastly, 2.0 µL of a potassium ferrocyanide stock solution (500 mM, 1000 nmol, 500 equiv.) in water was added. The mixture was vortexed for 5 seconds, transferred into a Thermocycler pre-heated at 95 °C, and incubated at 95 °C for 15 min. at 600 rpm.

After 15 minutes, 10 µL of a solution 100 mM of sodium diethyldithiocarbamate trihydrate in water were added to remove the palladium salts from the solution. Reaction mixture was centrifuged and an aliquot of 8 µL of the supernatant was diluted to 40 µL with water. Over the sample, 5 µL of a 5 M solution of NaCl in water and 150 µL of ethanol at −20 °C were added to precipitate the DNA conjugate. The Eppendorf tube was placed in the freezer (−20 °C) for at least 1 hour, and then it was centrifuged at 4 °C and 10000 x g for at least 30 minutes. The supernatant was removed, and the pellet was redissolved in 50 µL of water for LC–MS analysis.

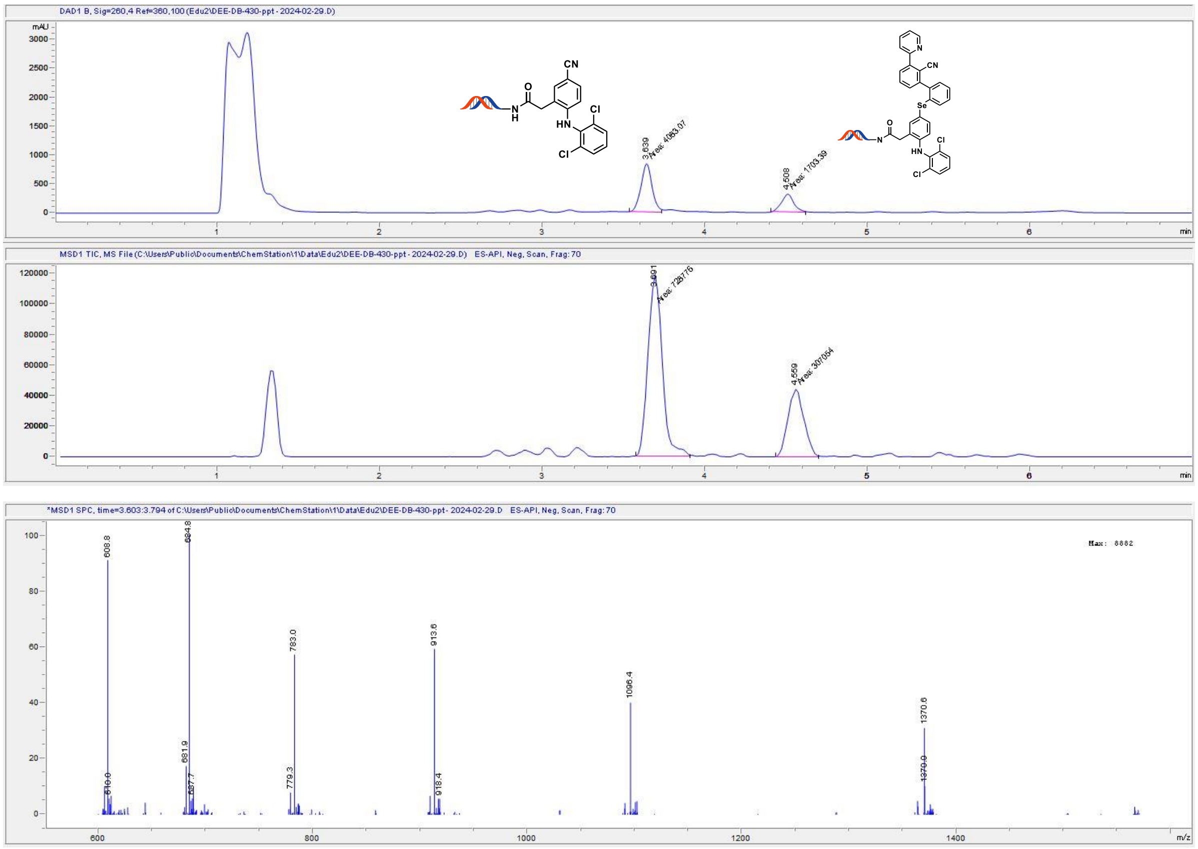

Figure S96. Analytical HPLC trace of **43** with HPLC Method B. (Up) DAD chromatogram at 260 nm.

(Middle) TIC chromatogram. (Below) Ionization of peak at 3.691 min containing reaction product.

C–S coupling of DNA-conjugated selenonium salt 24

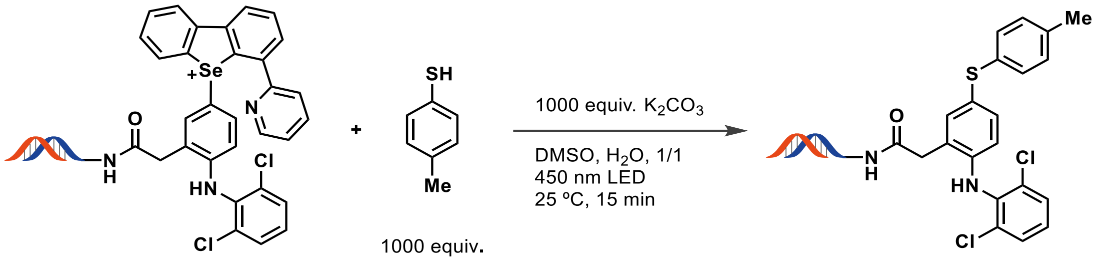

24 **44**, 74%

Exact Mass: 5581.049
Molecular Weight: 5584.838

At 20–25 °C, 5.0 µL of **24** (2.0 mM, 10 nmol, 1.0 equiv.) in water was added to a 1.5 mL Eppendorf tube. Next, 10.0 µL of a fresh solution 4-methylbenzenethiol stock solution (1.0 M, 10.0 µmol, 1000 equiv.) in DMSO was added. Then, 5.0 µL of a sodium carbonate stock solution (2.0 M, 10.0 µmol, 1000 equiv.) in water was added. A light stream of argon was blown over the solution for 30 seconds. The mixture was vortexed for 5 seconds, transferred into a Penn PhD Photoreactor M2, and irradiated at 450 nm for 15 minutes at 25 °C. After the corresponding time, an aliquot of 2 µL of the reaction mixture was diluted to 40 µL with water for LC–MS analysis. *Conversion was calculated using the TIC chromatogram.*

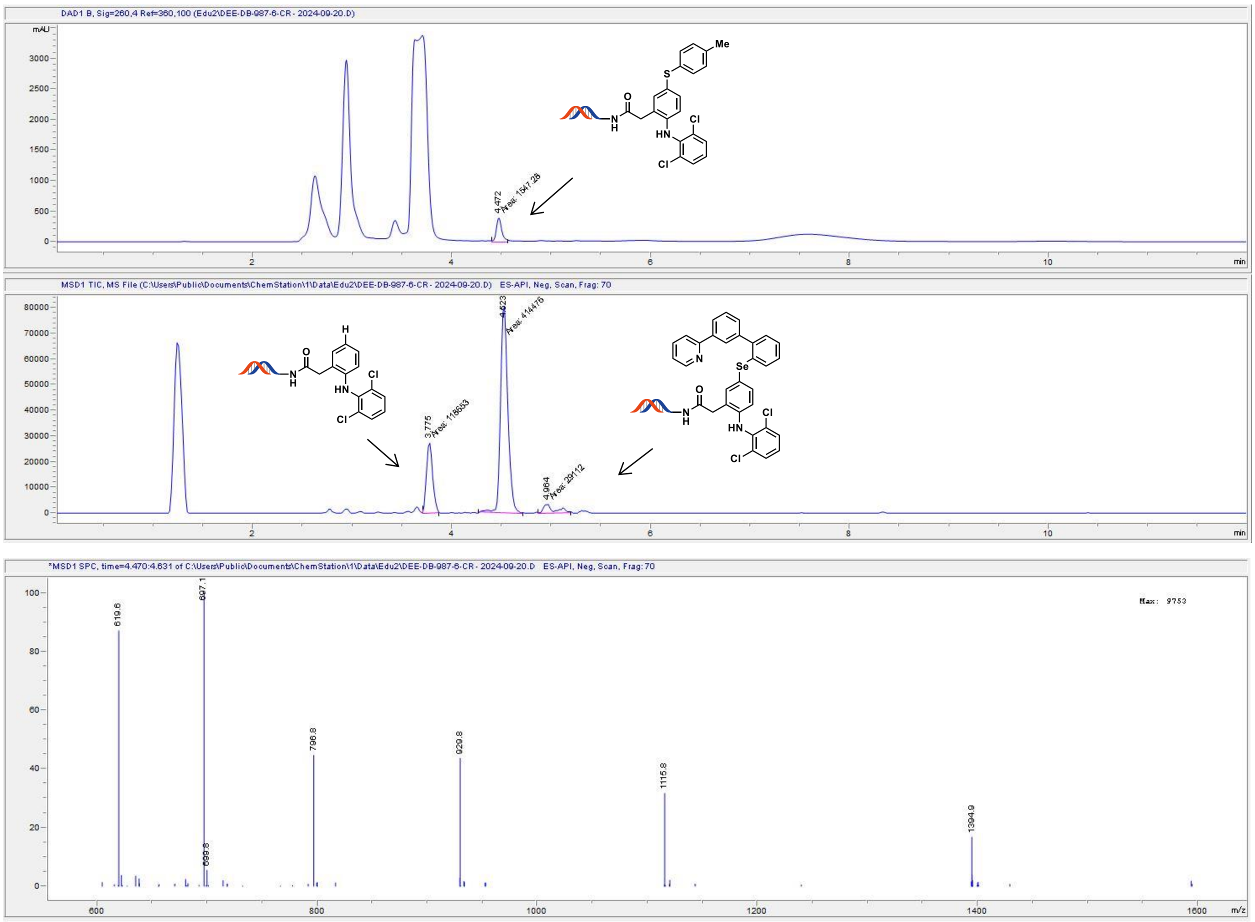

Figure S97. Analytical HPLC trace of **44** with HPLC Method A. (Up) DAD chromatogram at 260 nm. (Middle) TIC chromatogram. (Below) Ionization of peak at 4.523 min containing reaction product. Conversion was calculated using the TIC chromatogram.

Minisci reaction of DNA-conjugated selenonium salt 24

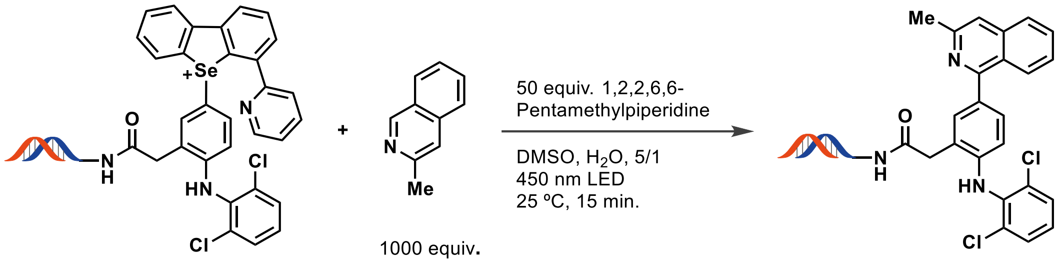

24 **45**, 57%

Exact Mass: 5600.088
Molecular Weight: 5603.826

At 20–25 °C, 5.0 µL of **24** (2.0 mM, 10.0 nmol, 1.0 equiv.) in water was added to a 1.5 mL Eppendorf tube. Next, 5.0 µL of a 1,2,2,6,6-Pentamethylpiperidine stock solution (100 mM, 500 nmol, 50 equiv.) in DMSO was added. Then, 20.0 µL of a 3-methylisoquinoline stock solution (500 mM, 10.0 µmol, 1000 equiv.) in DMSO was added. A light stream of argon was blown over the solution for 30 seconds. The mixture was vortexed for 5 seconds, transferred into a Penn PhD Photoreactor M2, and irradiated at 450 nm for 15 minutes at 25 °C. After 15 minutes, reaction mixture was centrifuged and diluted to 40 µL with water. Over the sample, 7 µL of a 5 M solution of NaCl in water and 230 µL of ethanol at −20 °C were added to precipitate the DNA conjugate. The Eppendorf tube was placed in the freezer (−20 °C) for at least 1 hour, and then it was centrifuged at 4 °C and 10000 x g for at least 30 minutes. The supernatant was removed, and the pellet was redissolved in 50 µL of water for LC–MS analysis.

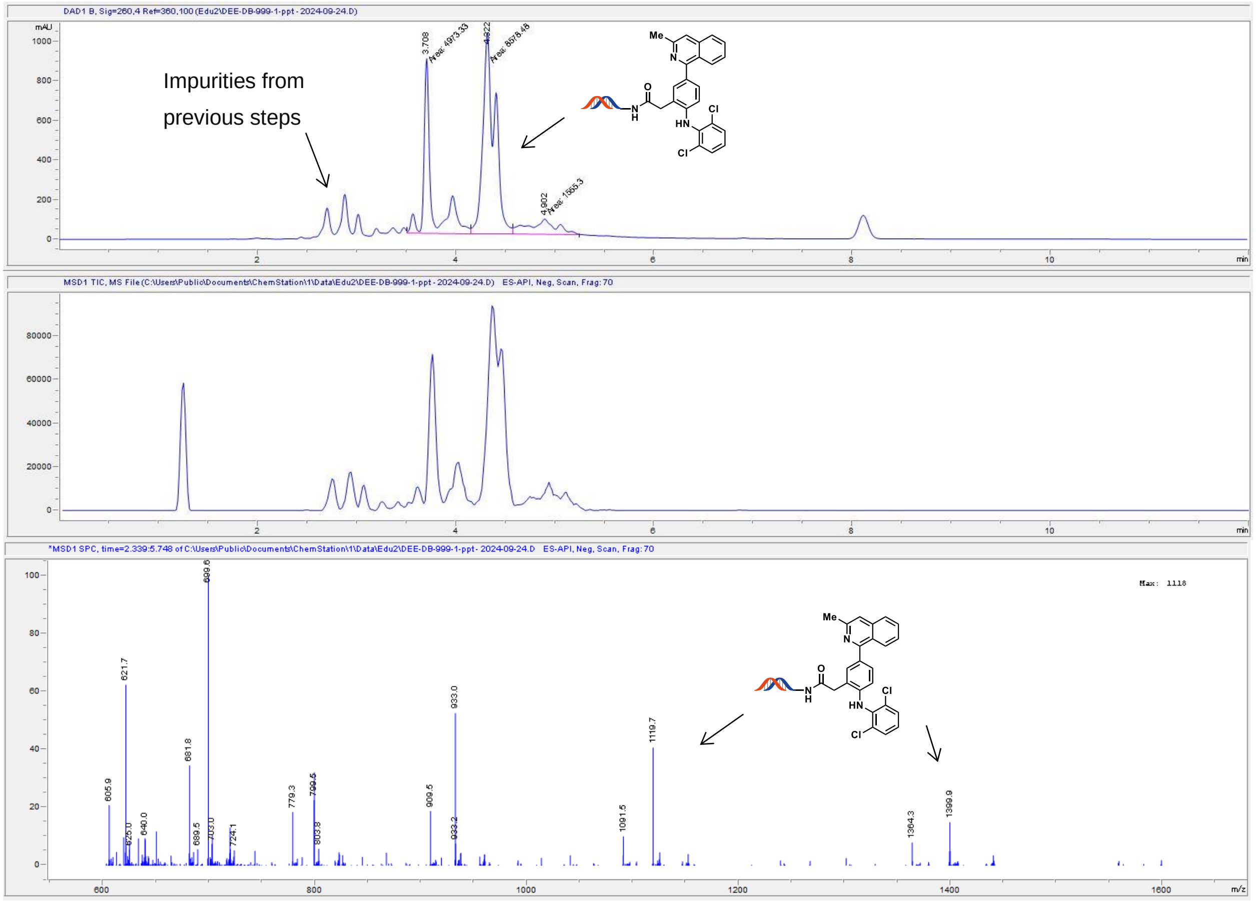

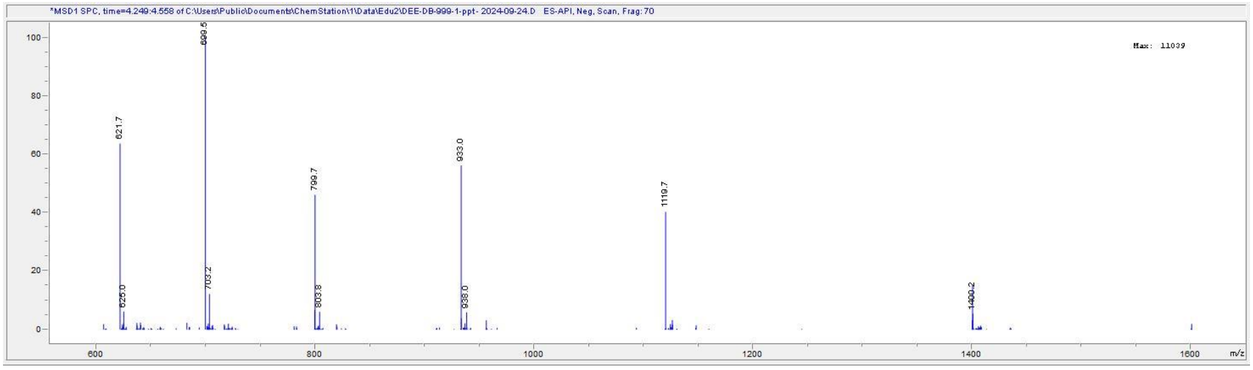

Figure S98. Analytical HPLC trace of **45** with HPLC Method A. (Up) DAD chromatogram at 260 nm. (Middle up) TIC chromatogram. (Middle down) Ionization of the full chromatogram. (Below) Ionization of peak from 4.249–4.558 min containing reaction product.

Suzuki coupling of DNA-conjugated selenonium salt 24 with 2-methoxypyrimidine-5-boronic acid

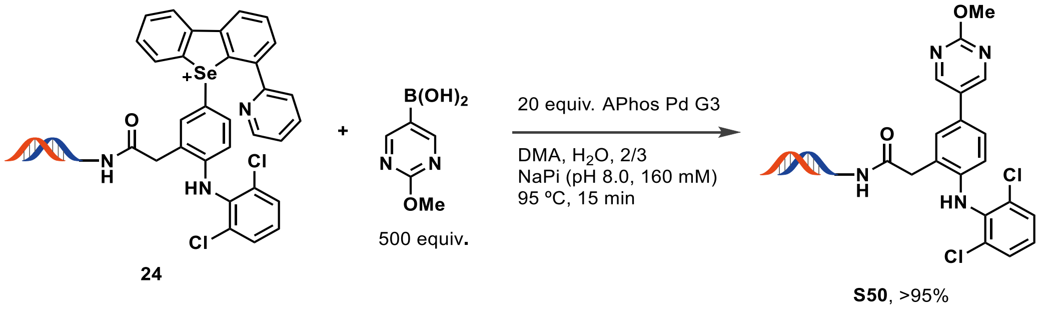

At 20–25 °C, 2.0 µL of **24** (1.0 mM, 2.0 nmol, 1.0 equiv.) in water was added to a 1.5 mL Eppendorf tube. Next, 4.0 µL of Phosphate buffer (NaPi, pH 8.0, 500 mM) was added. 2.0 µL of an APhos Pd G3 stock solution (20 mM, 20 nmol, 20 equiv.) in DMA was added over the solution of **24.** The mixture was vortexed for 5 seconds. Lastly, 2.0 µL of a 2-methoxypyrimidine-5-boronic acid stock solution (500 mM, 1000 nmol, 500 equiv.) in DMA was added. The mixture was vortexed for 5 seconds, transferred into a Thermocycler pre-heated at 95 °C, and incubated at 95 °C for 15 min. at 600 rpm.

After 15 minutes, 10 µL of a 100 mM solution of sodium diethyldithiocarbamate trihydrate in water were added to remove the palladium salts from the solution. The reaction mixture was centrifuged and an aliquot of 8 µL of the supernatant was diluted to 40 µL with water. Over the sample, 5 µL of a 5 M solution of NaCl in water and 150 µL of ethanol at −20 °C were added to precipitate the DNA conjugate. The Eppendorf tube was placed in the freezer (−20 °C) for at least 1 hour, and then it was centrifuged at 4 °C and 10000 x g for at least 30 minutes. The supernatant was removed, and the pellet was redissolved in 50 µL of water for LC–MS analysis.

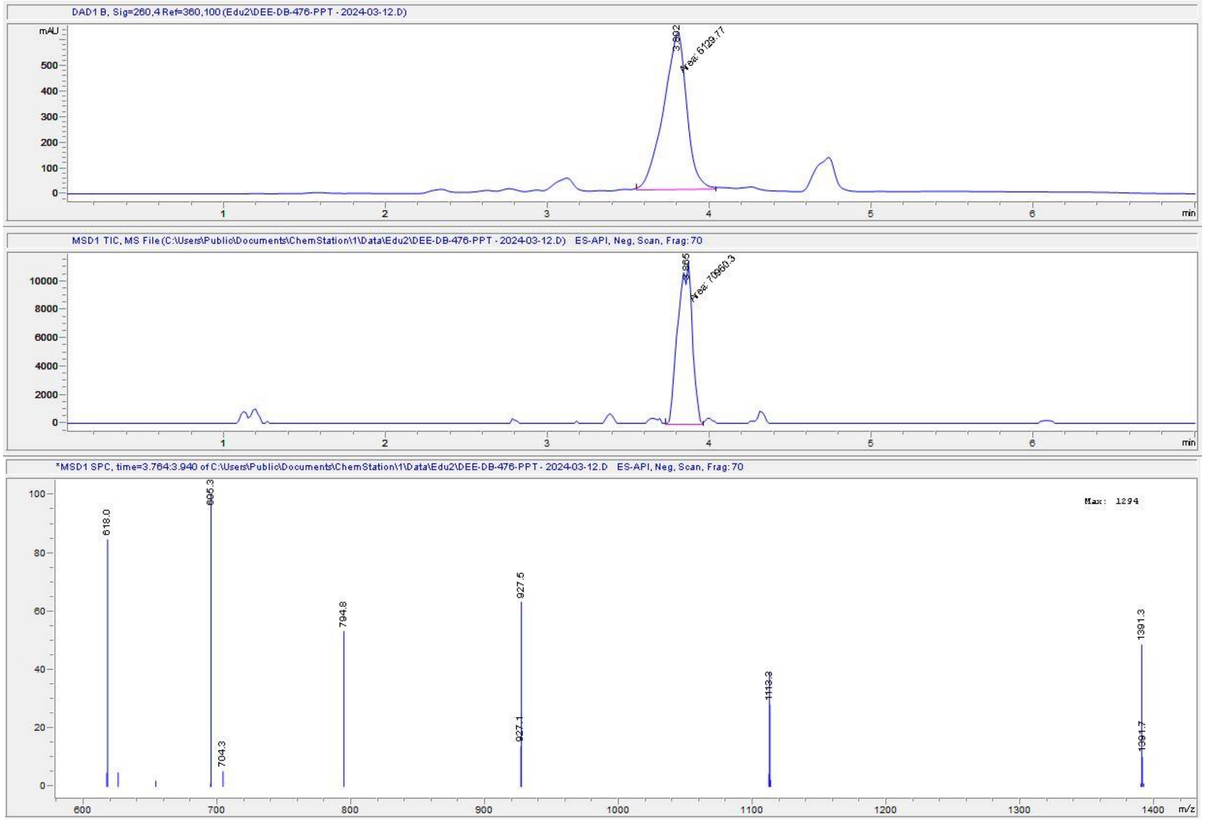

Figure S99. Analytical HPLC trace of **S50** with HPLC Method B. (Up) DAD chromatogram at 260 nm. (Middle) TIC chromatogram. (Below) Ionization of peak at 3.865 min containing reaction product.

Suzuki coupling of DNA-conjugated selenonium salt 7 with 2-methoxypyrimidine-5-boronic acid

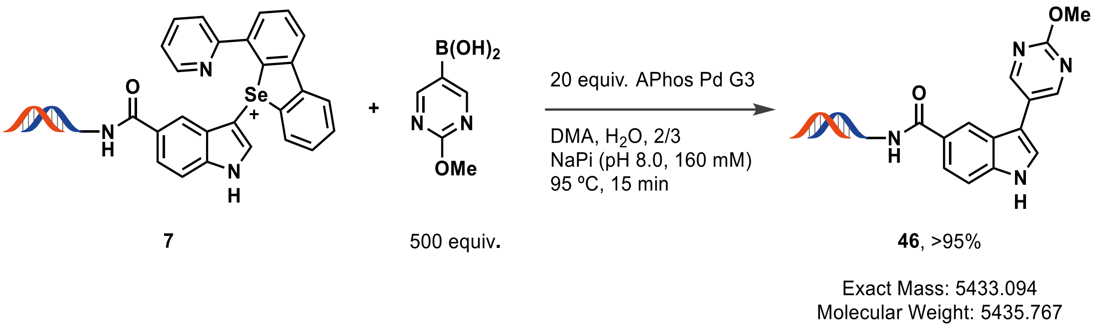

At 20–25 °C, 2.0 µL of **7** (1.0 mM, 2.0 nmol, 1.0 equiv.) in water was added to a 1.5 mL Eppendorf tube. Next, 4.0 µL of Phosphate buffer (NaPi, pH 8.0, 500 mM) was added. 2.0 µL of an APhos Pd G3 stock solution (20 mM, 20 nmol, 20 equiv.) in DMA was added over the solution of **7**. The mixture was vortexed for 5 seconds. Lastly, 2.0 µL of a 2-methoxypyrimidine-5-boronic acid stock solution (500 mM, 1000 nmol, 500 equiv.) in DMA was added. The mixture was vortexed for 5 seconds, transferred into a Thermocycler pre-heated at 95 °C, and incubated at 95 °C for 15 min. at 600 rpm.

After 15 minutes, 10 µL of a 100 mM solution of sodium diethyldithiocarbamate trihydrate in water were added to remove the palladium salts from the solution. The reaction mixture was centrifuged and an aliquot of 8 µL of the supernatant was diluted to 40 µL with water. Over the sample, 5 µL of a 5 M solution of NaCl in water and 150 µL of ethanol at −20 °C were added to precipitate the DNA conjugate. The Eppendorf tube was placed in the freezer (−20 °C) for at least 1 hour, and then it was centrifuged at 4 °C and 10000 x g for at least 30 minutes. The supernatant was removed, and the pellet was redissolved in 50 µL of water for LC–MS analysis.

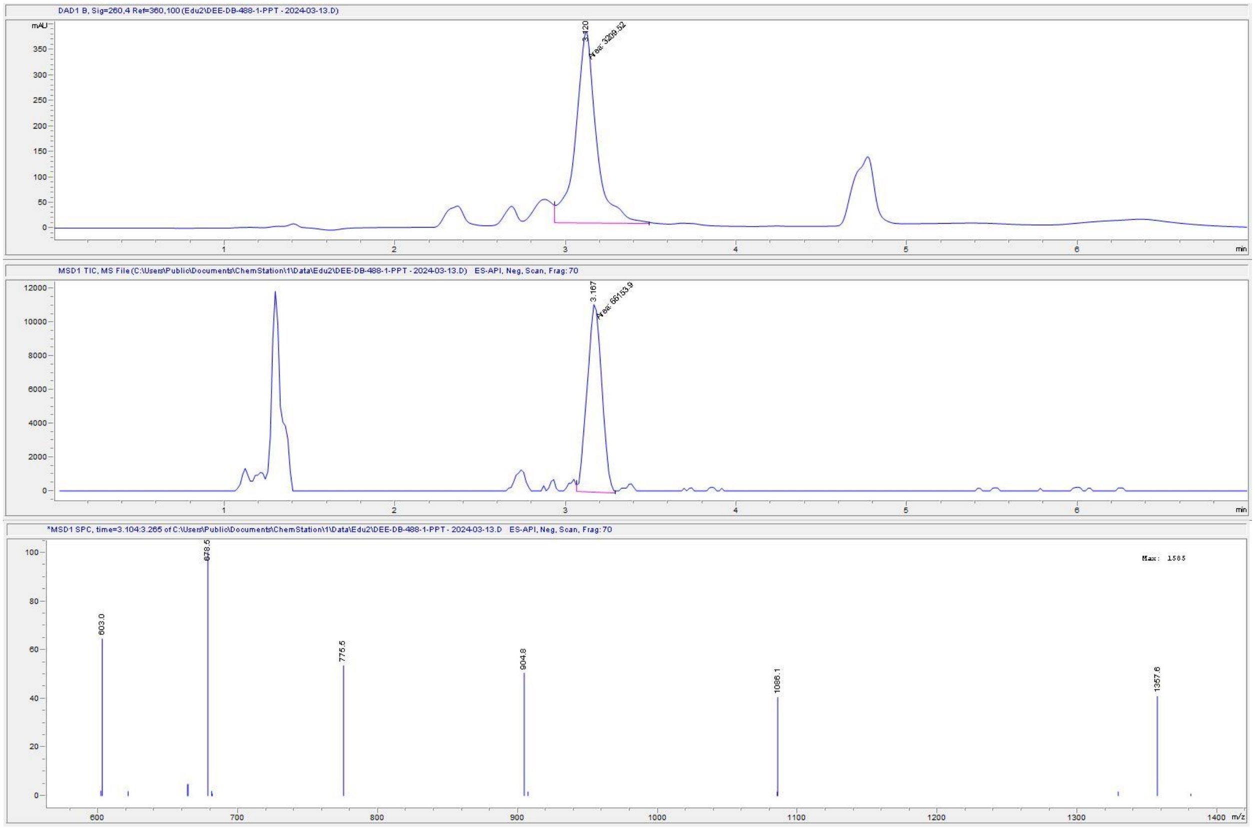

Figure S100. Analytical HPLC trace of **46** with HPLC Method B. (Up) DAD chromatogram at 260 nm. (Middle) TIC chromatogram. (Below) Ionization of peak at 3.167 min containing reaction product.

Suzuki coupling of DNA-conjugated selenonium salt 15 with 2-methoxypyrimidine-5-boronic acid

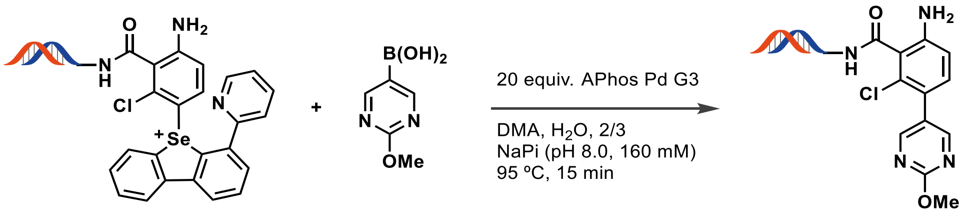

15 500 equiv. **47**, >95%

Exact Mass: 5443.054
Molecular Weight: 5446.186

At 20–25 °C, 2.0 µL of **15** (1.0 mM, 2.0 nmol, 1.0 equiv.) in water was added to a 1.5 mL Eppendorf tube. Next, 4.0 µL of Phosphate buffer (NaPi, pH 8.0, 500 mM) was added. 2.0 µL of an APhos Pd G3 stock solution (20 mM, 20 nmol, 20 equiv.) in DMA was added over the solution of **15**. The mixture was vortexed for 5 seconds. Lastly, 2.0 µL of a 2-methoxypyrimidine-5-boronic acid stock solution (500 mM, 1000 nmol, 500 equiv.) in DMA was added. The mixture was vortexed for 5 seconds, transferred into a Thermocycler pre-heated at 95 °C, and incubated at 95 °C for 15 min. at 600 rpm.

After 15 minutes, 10 µL of a 100 mM solution of sodium diethyldithiocarbamate trihydrate in water were added to remove the palladium salts from the solution. The reaction mixture was centrifuged and an aliquot of 8 µL of the supernatant was diluted to 40 µL with water. Over the sample, 5 µL of a 5 M solution of NaCl in water and 150 µL of ethanol at −20 °C were added to precipitate the DNA conjugate. The Eppendorf tube was placed in the freezer (−20 °C) for at least 1 hour, and then it was centrifuged at 4 °C and 10000 x g for at least 30 minutes. The supernatant was removed, and the pellet was redissolved in 50 µL of water for LC–MS analysis.

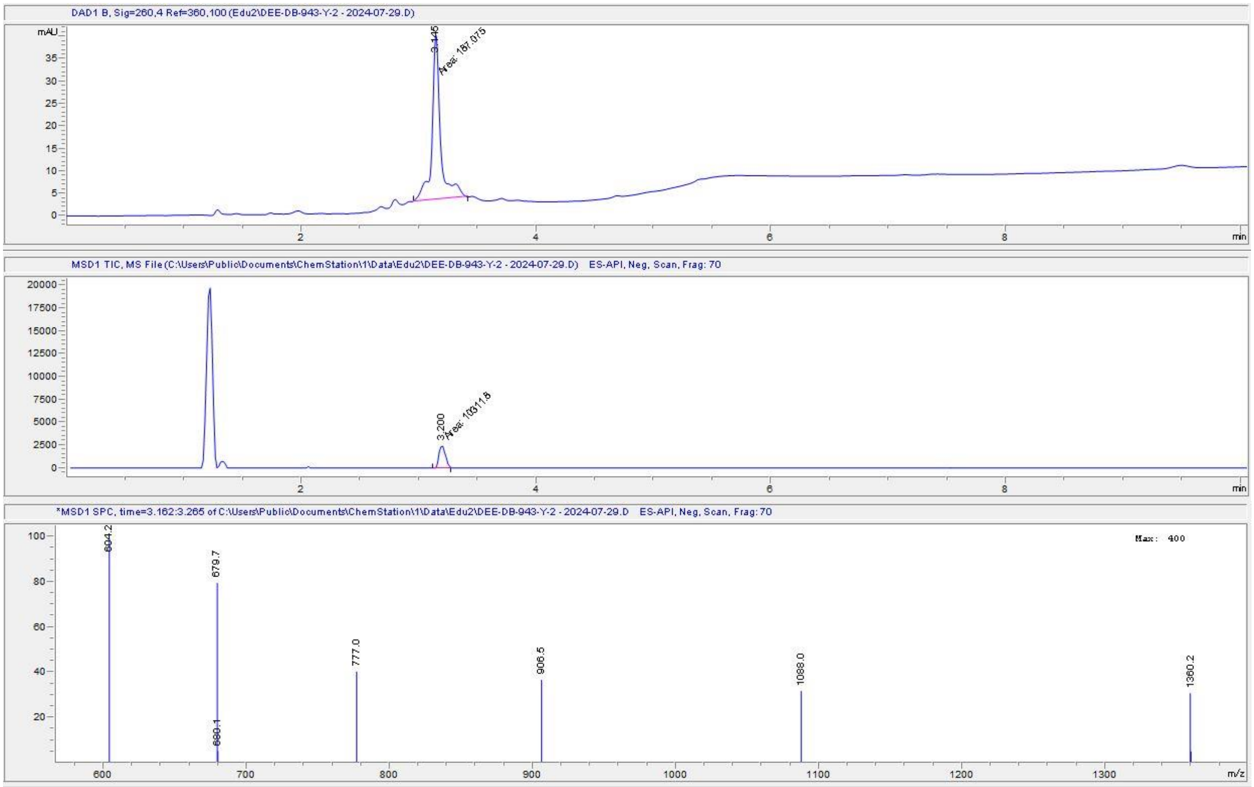

Figure S101. Analytical HPLC trace of **47** with HPLC Method A. (Up) DAD chromatogram at 260 nm. (Middle) TIC chromatogram. (Below) Ionization of peak at 3.200 min containing reaction product.

Suzuki coupling of DNA-conjugated selenonium salt 28 with 2-methoxypyrimidine-5-boronic acid

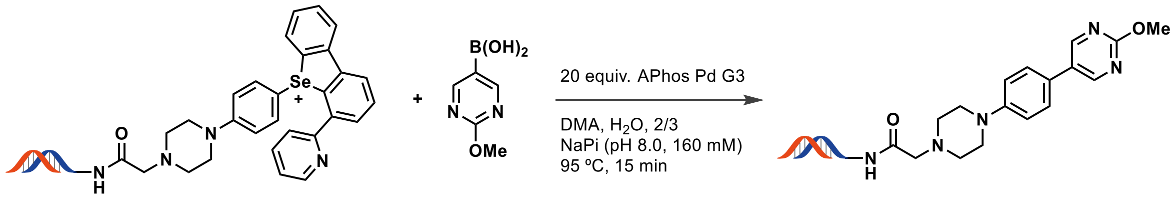

28 500 equiv. **48**, >95%

Exact Mass: 5492.168
Molecular Weight: 5494.879

At 20–25 °C, 2.0 µL of **28** (1.0 mM, 2.0 nmol, 1.0 equiv.) in water was added to a 1.5 mL Eppendorf tube. Next, 4.0 µL of Phosphate buffer (NaPi, pH 8.0, 500 mM) was added. 2.0 µL of an APhos Pd G3 stock solution (20 mM, 20 nmol, 20 equiv.) in DMA was added over the solution of **28**. The mixture was vortexed for 5 seconds. Lastly, 2.0 µL of a 2-methoxypyrimidine-5-boronic acid stock solution (500 mM, 1000 nmol, 500 equiv.) in DMA was added. The mixture was vortexed for 5 seconds, transferred into a Thermocycler pre-heated at 95 °C, and incubated at 95 °C for 15 min. at 600 rpm.

After 15 minutes, 10 µL of a 100 mM solution of sodium diethyldithiocarbamate trihydrate in water were added to remove the palladium salts from the solution. The reaction mixture was centrifuged and an aliquot of 8 µL of the supernatant was diluted to 40 µL with water. Over the sample, 5 µL of a 5 M solution of NaCl in water and 150 µL of ethanol at −20 °C were added to precipitate the DNA conjugate. The Eppendorf tube was placed in the freezer (−20 °C) for at least 1 hour, and then it was centrifuged at 4 °C and 10000 x g for at least 30 minutes. The supernatant was removed, and the pellet was redissolved in 50 µL of water for LC–MS analysis.

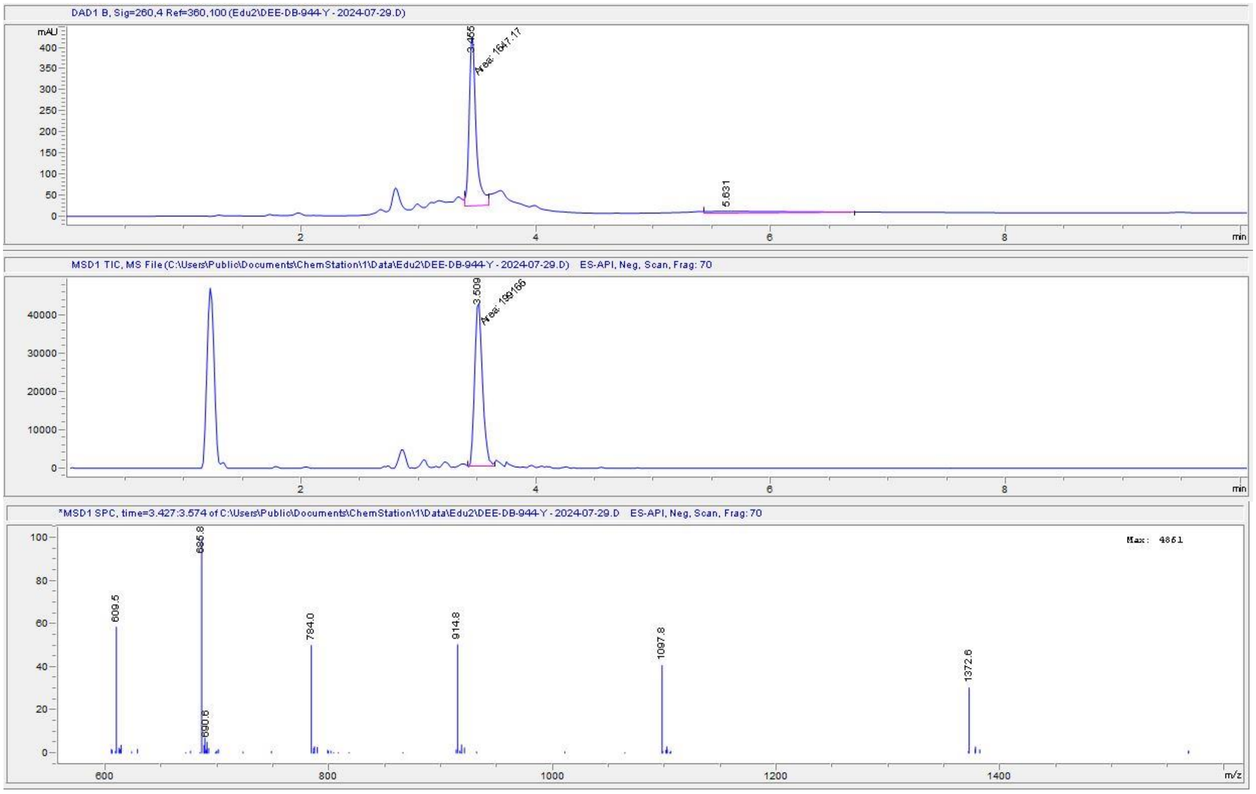

Figure S102. Analytical HPLC trace of **48** with HPLC Method A. (Up) DAD chromatogram at 260 nm. (Middle) TIC chromatogram. (Below) Ionization of peak at 3.509 min containing reaction product.

Suzuki coupling of DNA-conjugated selenonium salt 23 with 6-chloro-3-pyridineboronic acid pinacol ester

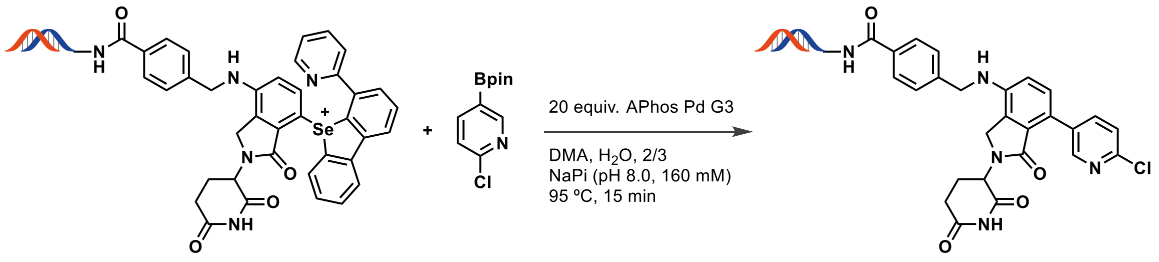

23

49, 64%

Exact Mass: 5668.133
Molecular Weight: 5671.433

At 20–25 °C, 2.0 µL of **23** (1.0 mM, 2.0 nmol, 1.0 equiv.) in water was added to a 1.5 mL Eppendorf tube. Next, 4.0 µL of Phosphate buffer (NaPi, pH 8.0, 500 mM) was added. 2.0 µL of an APhos Pd G3 stock solution (20 mM, 20 nmol, 20 equiv.) in DMA was added over the solution of **23**. The mixture was vortexed for 5 seconds. Lastly, 2.0 µL of a 6-chloro-3-pyridineboronic acid pinacol ester stock solution (500 mM, 1000 nmol, 500 equiv.) in DMA was added. The mixture was vortexed for 5 seconds, transferred into a Thermocycler pre-heated at 95 °C, and incubated at 95 °C for 15 min. at 600 rpm.

After 15 minutes, 10 µL of a 100 mM solution of sodium diethyldithiocarbamate trihydrate in water were added to remove the palladium salts from the solution. The reaction mixture was centrifuged and an aliquot of 8 µL of the supernatant was diluted to 40 µL with water. Over the sample, 5 µL of a 5 M solution of NaCl in water and 150 µL of ethanol at −20 °C were added to precipitate the DNA conjugate. The Eppendorf tube was placed in the freezer (−20 °C) for at least 1 hour, and then it was centrifuged at 4 °C and 10000 x g for at least 30 minutes. The supernatant was removed, and the pellet was redissolved in

50 µL of water for LC–MS analysis. *Conversion was calculated using the TIC chromatogram.*

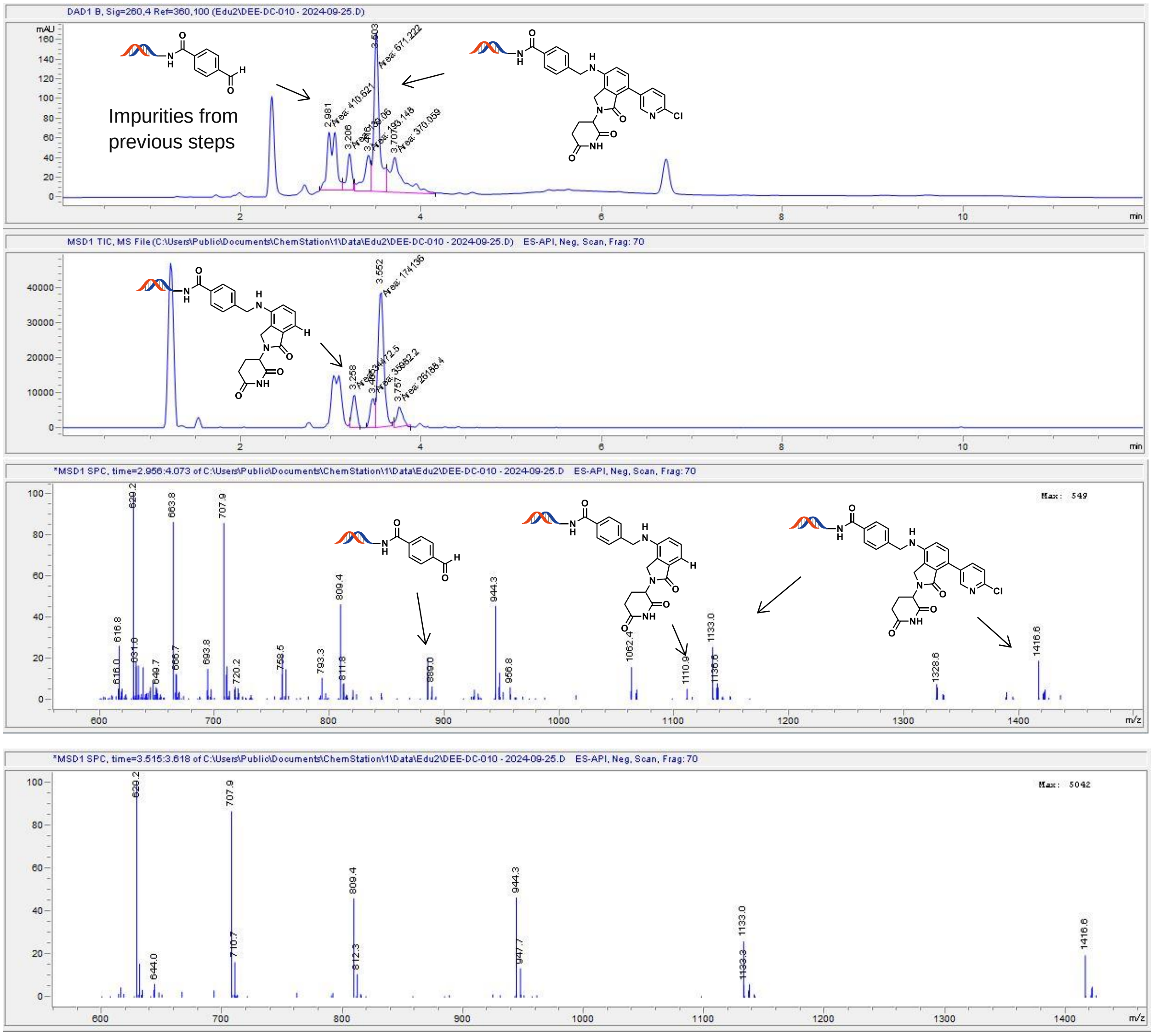

Figure S103. Analytical HPLC trace of **49** with HPLC Method A. (Up) DAD chromatogram at 260 nm. (Middle up) TIC chromatogram. (Middle down) Ionization of the full chromatogram. (Below) Ionization of peak at 3.552 min containing reaction product. Conversion was calculated using the TIC chromatogram.

Suzuki coupling of DNA-conjugated selenonium salt 25 with 2-methylpyridine-4-boronic acid

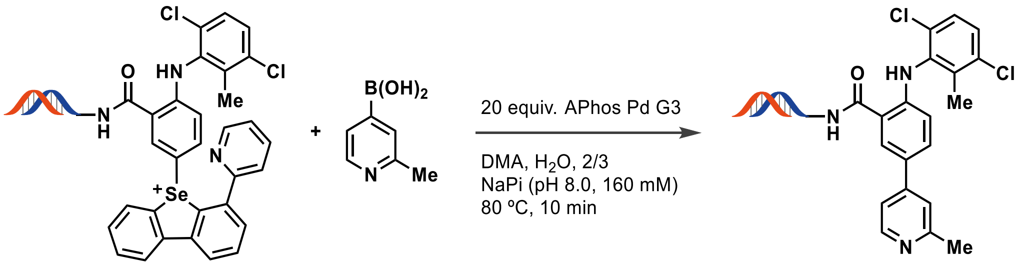

25 500 equiv. **50**, 79%

Exact Mass: 5550.072
Molecular Weight: 5553.766

At 20–25 °C, 2.0 µL of **25** (1.0 mM, 2.0 nmol, 1.0 equiv.) in water was added to a 1.5 mL Eppendorf tube. Next, 4.0 µL of Phosphate buffer (NaPi, pH 8.0, 500 mM) was added. 2.0 µL of an APhos Pd G3 stock solution (20 mM, 20 nmol, 20 equiv.) in DMA was added over the solution of **25**. The mixture was

vortexed for 5 seconds. Lastly, 2.0 µL of a 2-methylpyridine-4-boronic acid stock solution (500 mM, 1000 nmol, 500 equiv.) in DMA was added. The mixture was vortexed for 5 seconds, transferred into a Thermocycler pre-heated at 80 °C, and incubated at 80 °C for 10 min. at 600 rpm.

After 15 minutes, 10 µL of a 100 mM solution of sodium diethyldithiocarbamate trihydrate in water were added to remove the palladium salts from the solution. The reaction mixture was centrifuged and an aliquot of 8 µL of the supernatant was diluted to 40 µL with water. Over the sample, 5 µL of a 5 M solution of NaCl in water and 150 µL of ethanol at −20 °C were added to precipitate the DNA conjugate. The Eppendorf tube was placed in the freezer (−20 °C) for at least 1 hour, and then it was centrifuged at 4 °C and 10000 x g for at least 30 minutes. The supernatant was removed, and the pellet was redissolved in 50 µL of water for LC–MS analysis.

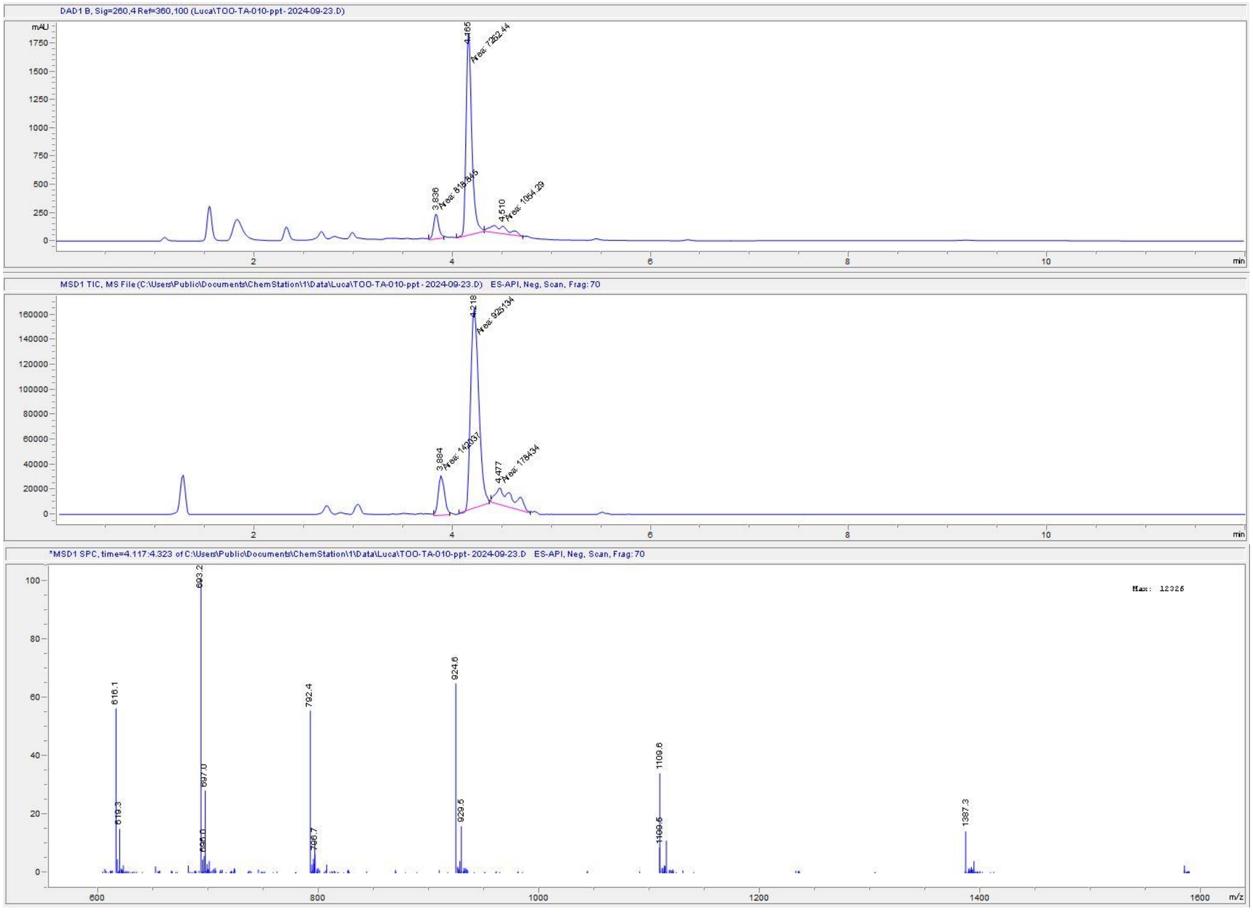

Figure S104. Analytical HPLC trace of **50** with HPLC Method A. (Up) DAD chromatogram at 260 nm. (Middle) TIC chromatogram. (Below) Ionization of peak at 4.218 min containing reaction product.

Minisci reaction of DNA-conjugated selenonium salt 18 with 3-methylisoquinoline

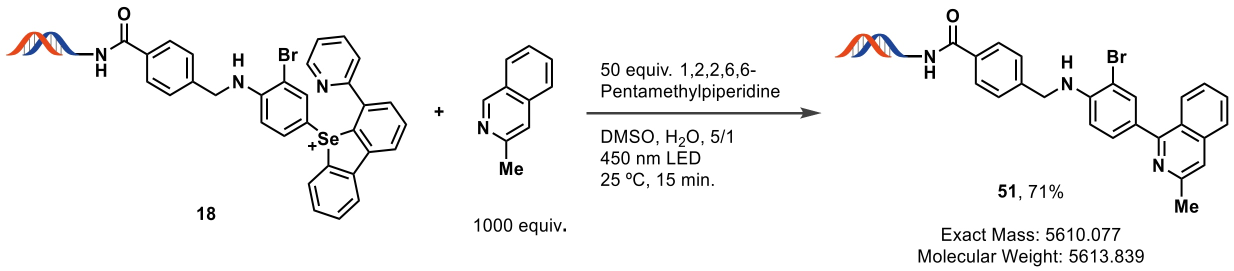

At 20–25 °C, 1.0 µL of **18** (2.0 mM, 2.0 nmol, 1.0 equiv.) in water was added to a 1.5 mL Eppendorf tube. Next, 1.0 µL of a 1,2,2,6,6-Pentamethylpiperidine stock solution (100 mM, 100 nmol, 50 equiv.) in DMSO

was added. Then, 4.0 µL of a 3-methylisoquinoline stock solution (500 mM, 2000 nmol, 1000 equiv.) in DMSO was added. A light stream of argon was blown over the solution for 30 seconds. The mixture was vortexed for 5 seconds, transferred into a Penn PhD Photoreactor M2, and irradiated at 450 nm for 15 minutes at 25 °C. After 15 minutes, reaction mixture was diluted in 50 µL of water for LC–MS analysis.

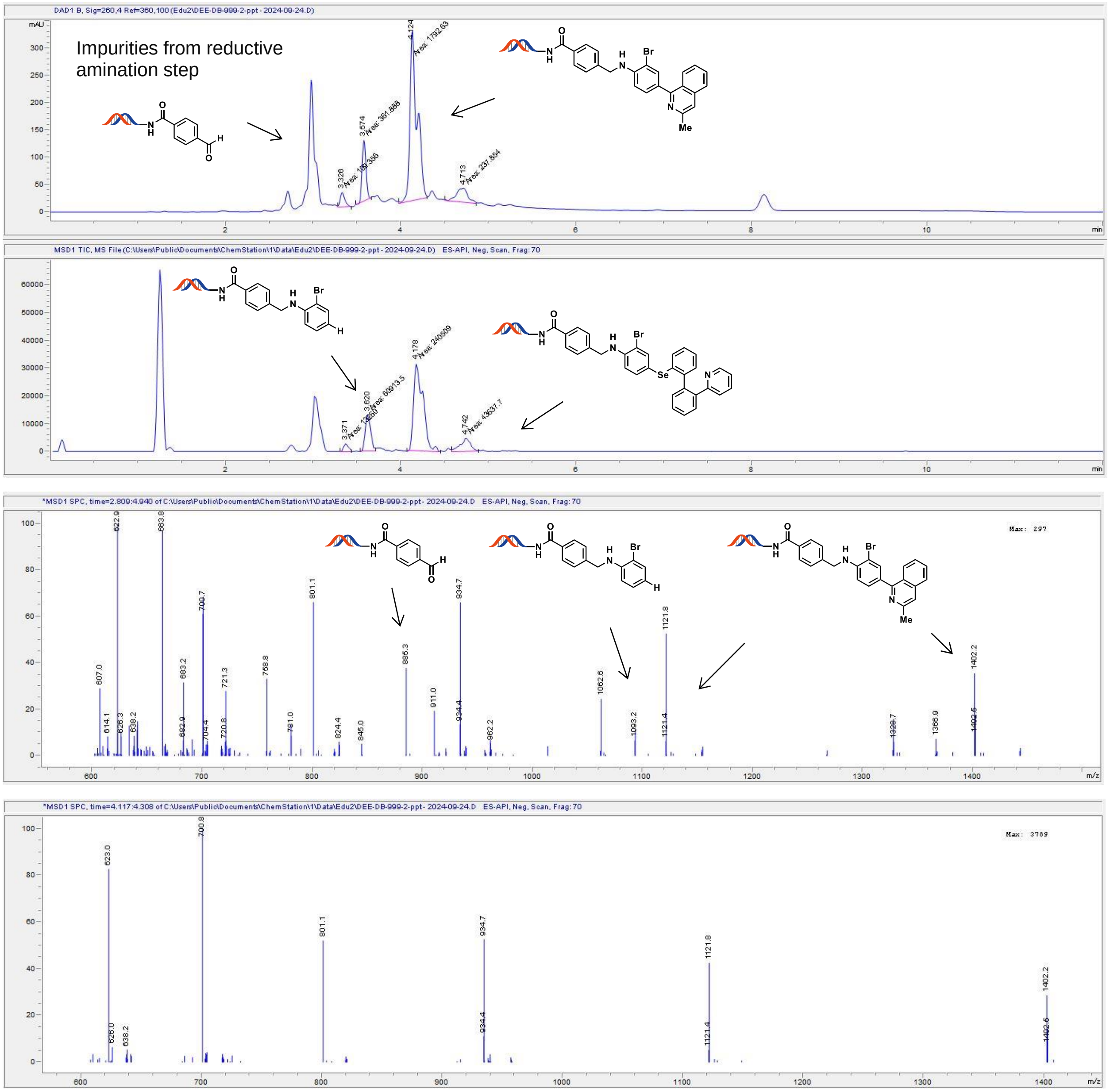

Figure S105. Analytical HPLC trace of **51** with HPLC Method A. (Up) DAD chromatogram at 260 nm. (Middle up) TIC chromatogram. (Middle down) Ionization of the full chromatogram. (Below) Ionization of peak at 4.178 min containing reaction product.

Proof-of-concept mock libraries

Mock library 1 – Cycle 1

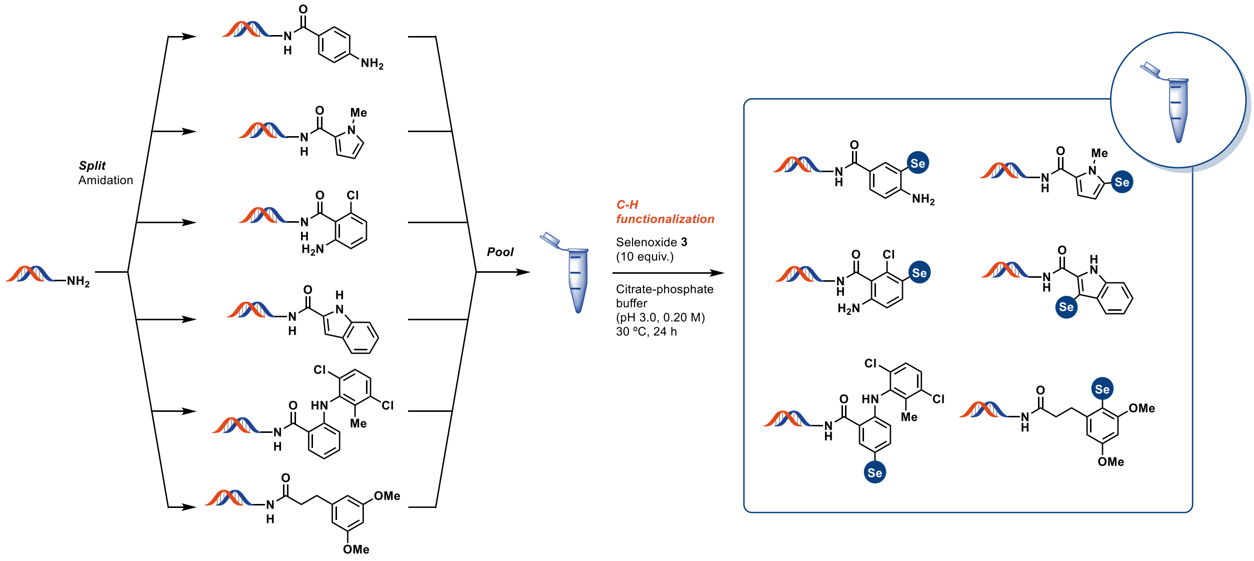

Cycle 1: Amide coupling

DNA-conjugated arenes **S5**, **S10**, **S18**, **S20**, **S23**, and **S26** were synthesized as described in section "Preparation of DNA-conjugated substrates". Then, 1.0 µL of each of the DNA-conjugated arenes (2.0 mM, 2.0 nmol, 1.0 equiv.) were pooled into a 1.5 mL Eppendorf tube, diluted with 300 µL of water and purified by charging the solution in an AMICON® filter unit from Sigma Aldrich (3 kD), centrifuged at 4 °C and 10000 x g for at least 30 minutes, until the volume decreased to < 10 µL. Another 300 µL of water were added and the process was repeated all over again for at least 3 times. The remaining solution concentration was determined by A_{260} absorption using a Thermo Scientific™ NanoDrop™ One^{C}, concentration of the solution was adjusted to 2.0 mM and used for the next step of the mock library. An aliquot of 0.5 µL of the pool mixture was diluted to 40 µL with water for LC–MS analysis. *All DNA-conjugated arenes were detected*.

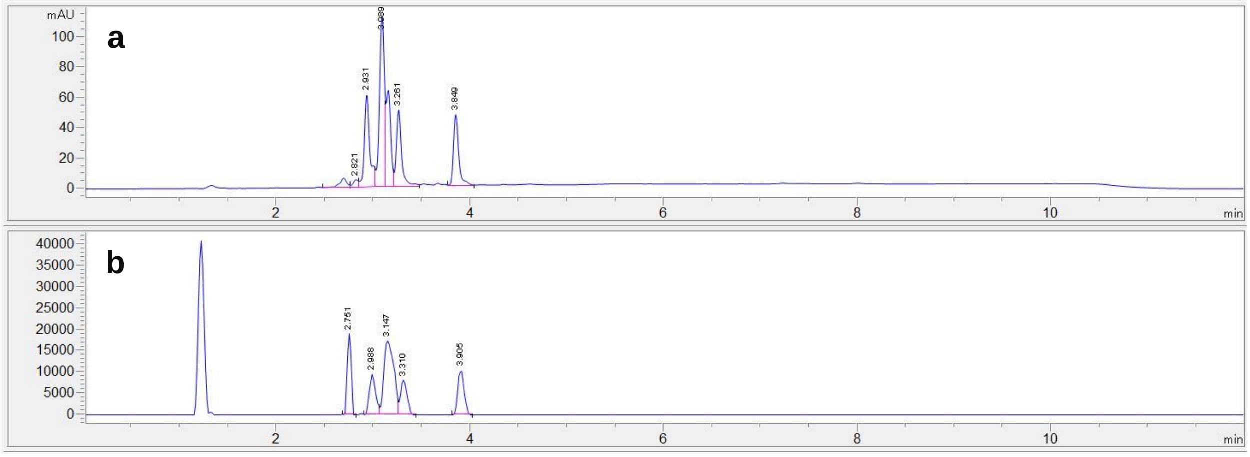

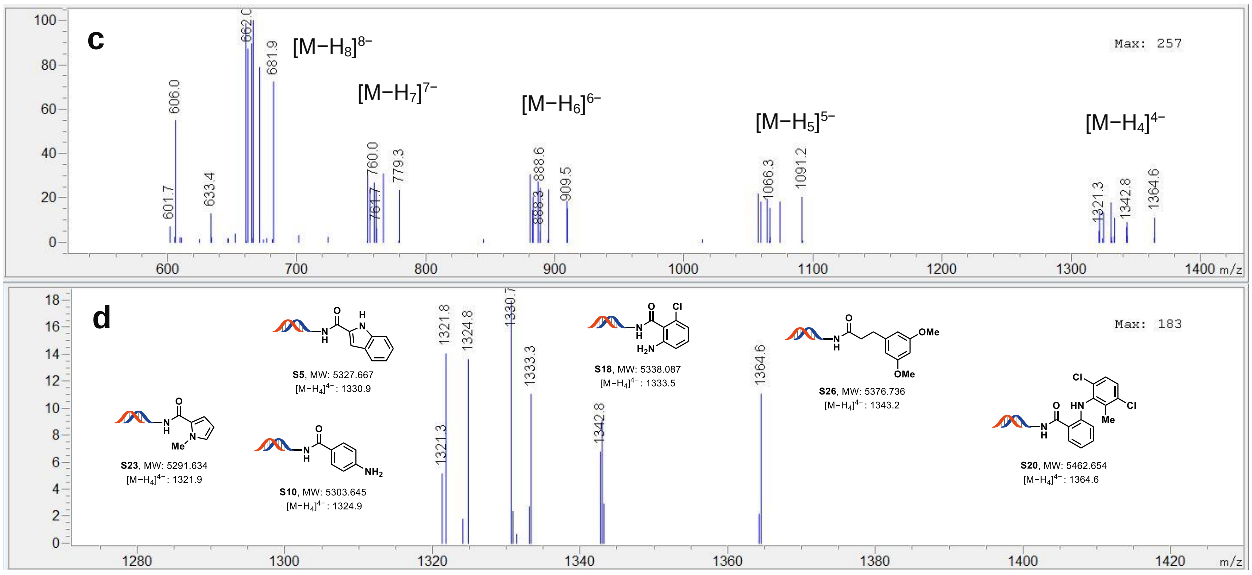

Figure S106. Analytical HPLC trace of the pool of mock library 1, cycle 1 before C−H functionalization with HPLC Method A. (a) DAD chromatogram at 260 nm. (b) TIC chromatogram. (c) Ionization of the full chromatogram. (d) Zoom of the MS spectrum m/z 1200–1500. *All DNA-conjugated arenes were detected*.

Cycle 1: C–H functionalization of mock library 1, cycle 1 pool

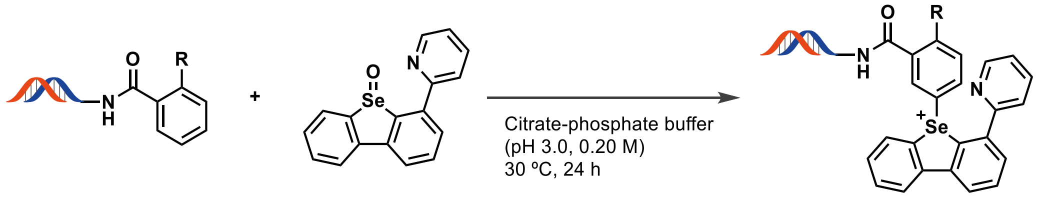

3
10 equiv.

At 20–25 °C, 5.0 µL of **mock library 1, cycle 1 pool** (2.0 mM, 10.0 nmol, 1.0 equiv.) in water was added to a 1.5 mL Eppendorf tube. Next, 20 µL of Citrate-phosphate buffer (pH 3.0, c = 500 mM) was added. Then, 25 µL of a selenoxide **3** stock solution (4.0 mM, 100 nmol, 10 equiv.) in water was added. The mixture was vortexed for 5 seconds, transferred into a Thermocycler pre-heated at 30 °C, and incubated at 30 °C for 24 hours at 600 rpm. After 24 hours, reaction was quenched by addition of 50 µL of borate buffer (pH 9.4, c = 500 mM). The crude reaction was purified by charging the solution in an AMICON® filter unit from Sigma Aldrich (3 kD) in 300 µL of water, centrifuged at 4 °C and 10000 x g for at least 30 minutes, until the volume decreased to < 10 µL. Another 300 µL of water were added and the process was repeated all over again for at least 3 times. The remaining solution concentration was determined by A_{260} absorption using a Thermo Scientific™ NanoDrop™ One^{C}, concentration of the solution was adjusted to 2.0 mM and stored in the freezer at −20 °C. Recovery of the DNA as measured by NanoDrop™ was quantitative (ca. 95%). An aliquot of 0.5 µL of the reaction mixture was diluted to 40 µL with water for LC–MS analysis. ***All potential C-H functionalization products were detected in the LC–MS analysis***.

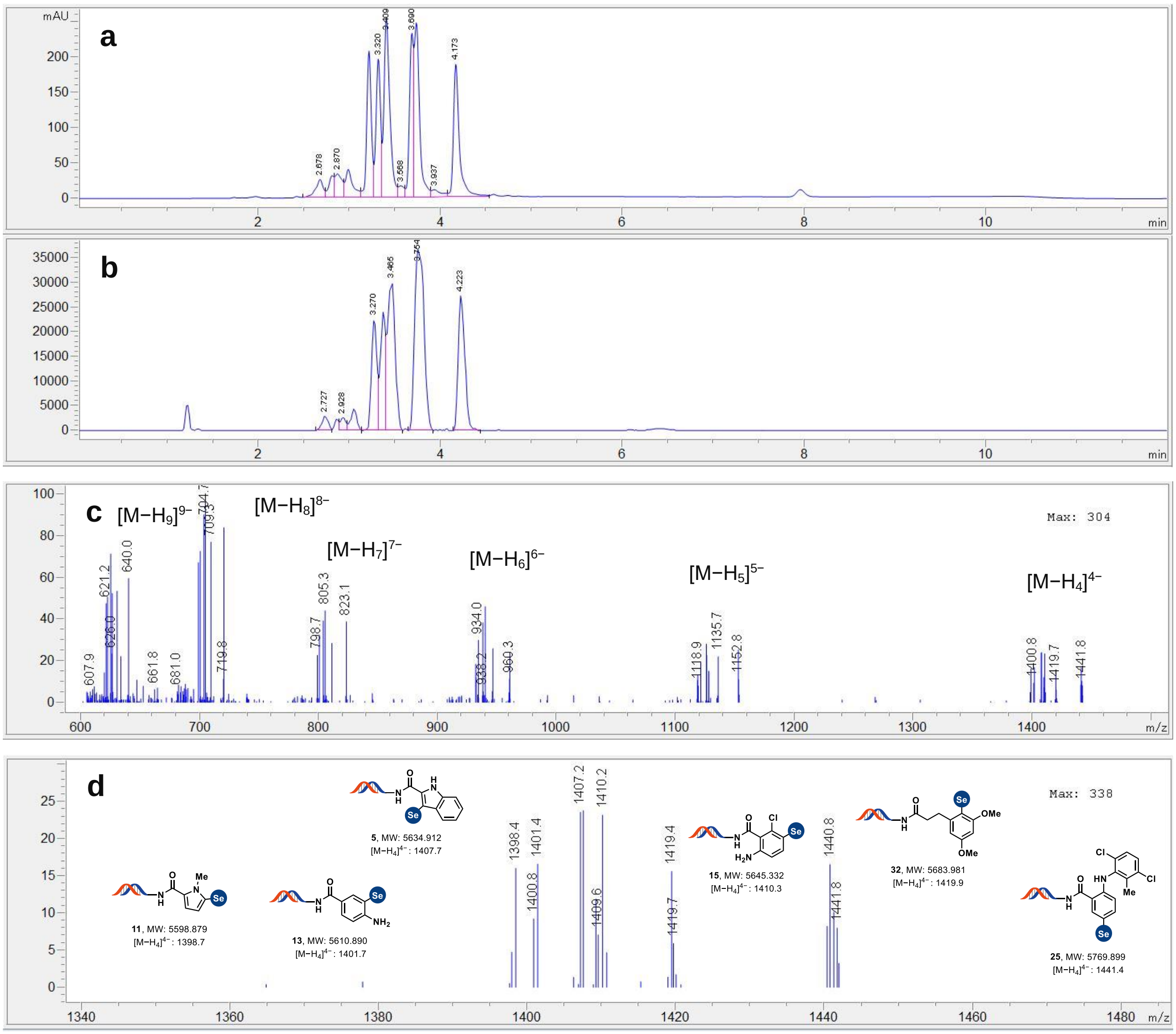

Figure S107. Analytical HPLC trace of the pool of mock library 1, cycle 1 after C−H functionalization with HPLC Method A. (a) DAD chromatogram at 260 nm. (b) TIC chromatogram. (c) Ionization of the full chromatogram. (d) Zoom of the MS spectrum m/z 1300─1500. *All potential C-H functionalization products were detected in the LC–MS analysis*.

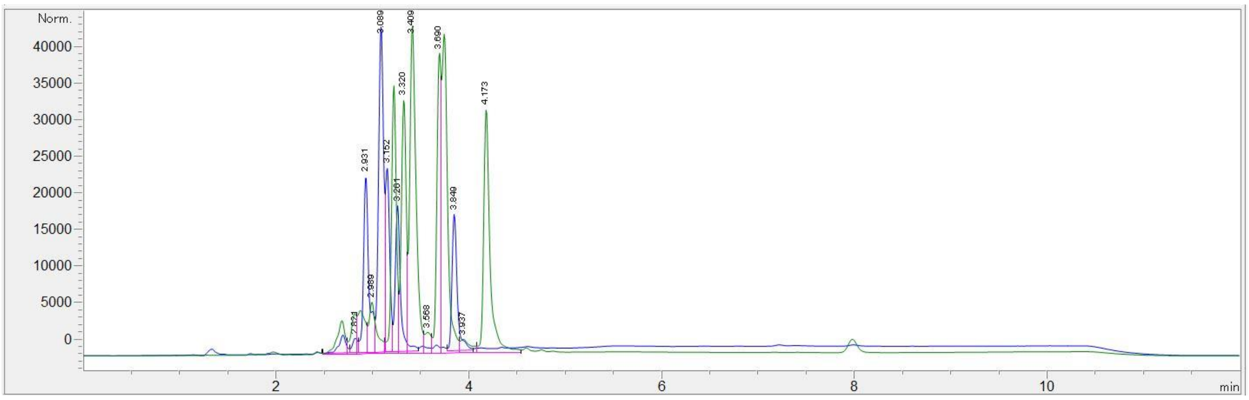

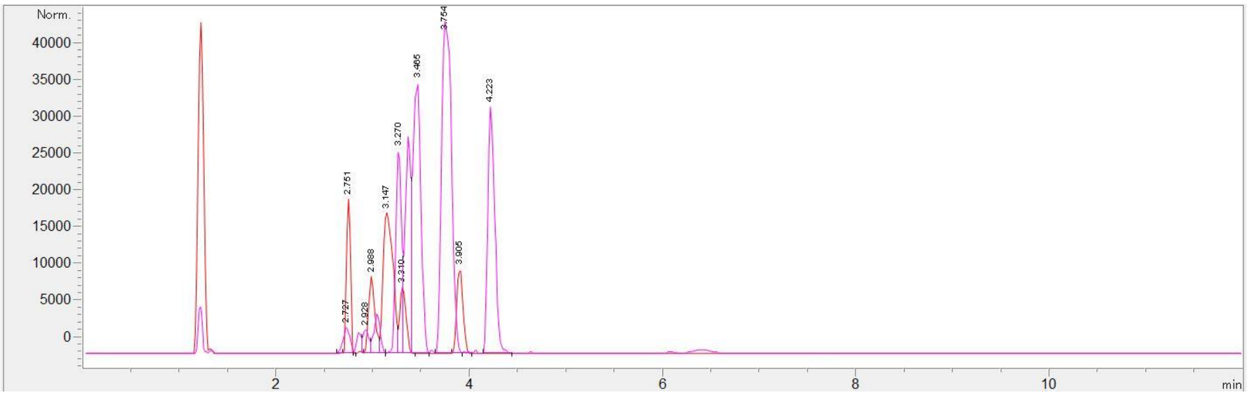

Figure S108. Overlay of analytical HPLC traces of the pool of mock library 1, cycle 1 before and after C−H functionalization with HPLC Method A. (Up) DAD chromatograms at 260 nm, before C−H functionalization (blue), after C−H functionalization (green). (Below) TIC chromatograms, before C−H functionalization (red), after C−H functionalization (pink).

Mock library 1 – Cycle 2

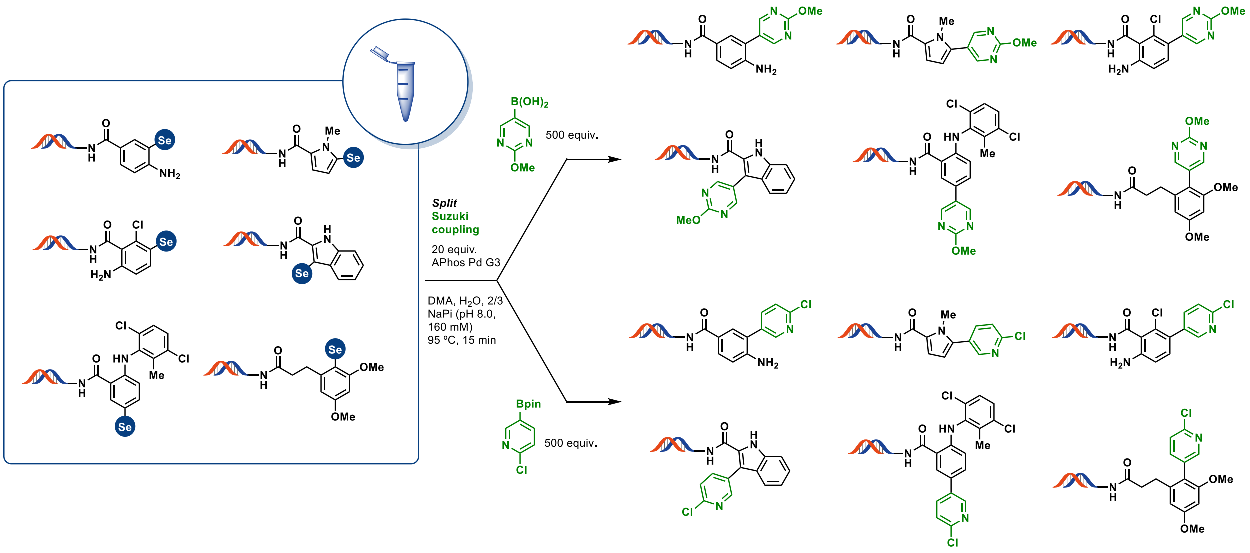

Cycle 2: Suzuki coupling of pool of DNA-conjugated selenonium salts 1 with 2-methoxypyrimidine-5-boronic acid

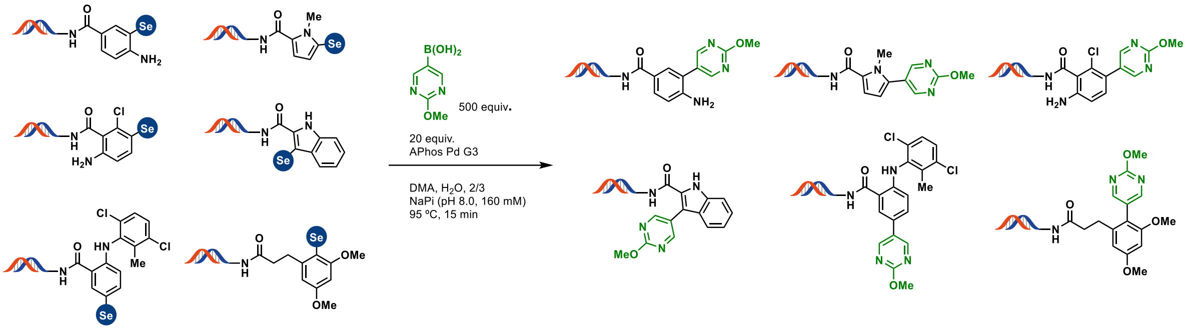

At 20–25 °C, 5.0 µL of the **pool of DNA-conjugated selenonium salts 1** (1.0 mM, 5.0 nmol, 1.0 equiv.) in water was added to a 1.5 mL Eppendorf tube. Next, 10 µL of Phosphate buffer (NaPi, pH 8.0, 500 mM) was added. 5.0 µL of an APhos Pd G3 stock solution (20 mM, 20 nmol, 20 equiv.) in DMA was added over the solution. The mixture was vortexed for 5 seconds. Lastly, 5.0 µL of a 2-methoxypyrimidine-5-boronic acid stock solution (500 mM, 1000 nmol, 500 equiv.) in DMA was added. The mixture was vortexed for 5 seconds, transferred into a Thermocycler pre-heated at 95 °C, and incubated at 95 °C for 15 min. at 600 rpm. After 15 minutes, 15 µL of a 100 mM solution of sodium diethyldithiocarbamate trihydrate in water were added to remove the palladium salts from the solution. The reaction mixture was centrifuged and 35 µL of the supernatant were collected. Over the supernatant, 4 µL of a 5 M solution of NaCl in water and 120 µL of ethanol at −20 °C were added to precipitate the DNA conjugate. The Eppendorf tube was placed in the freezer (−20 °C) for at least 1 hour, and then it was centrifuged at 4 °C and 10000 x g for at least 30 minutes. The supernatant was removed, and the pellet was redissolved in 150 µL of water for LC–MS analysis. ***All potential Suzuki coupling products were detected in the LC–MS analysis***. Other observed MS signals were corresponding to dehydrofunctionalization byproducts.

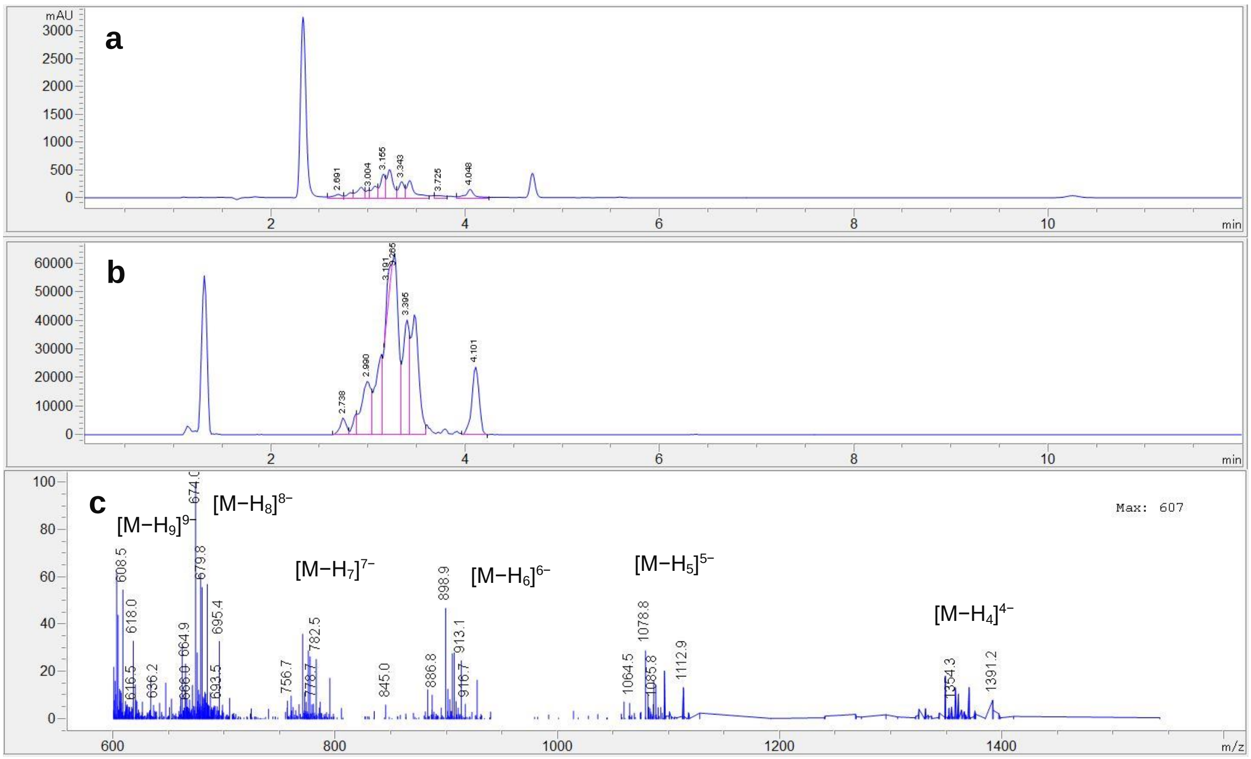

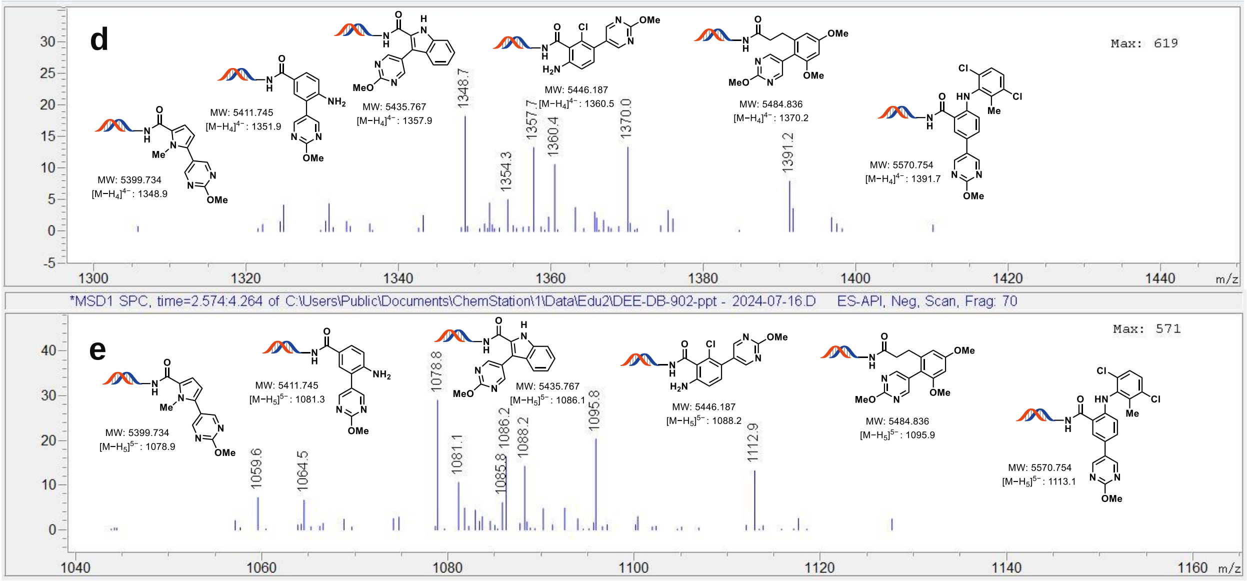

Figure S109. Analytical HPLC trace of the Suzuki coupling of mock library 1, cycle 2 with 2-methoxypyrimidine-5-boronic acid with HPLC Method A. (a) DAD chromatogram at 260 nm. (b) TIC chromatogram. (c) Ionization of the full chromatogram. (d) Zoom of the MS spectrum m/z 1300–1500. (e) Zoom of the MS spectrum m/z 1000–1200. *All potential Suzuki coupling products were detected in the LC–MS analysis*.

Cycle 2: Suzuki coupling of pool of DNA-conjugated selenonium salts 1 with 6-chloro-3-pyridineboronic acid pinacol ester

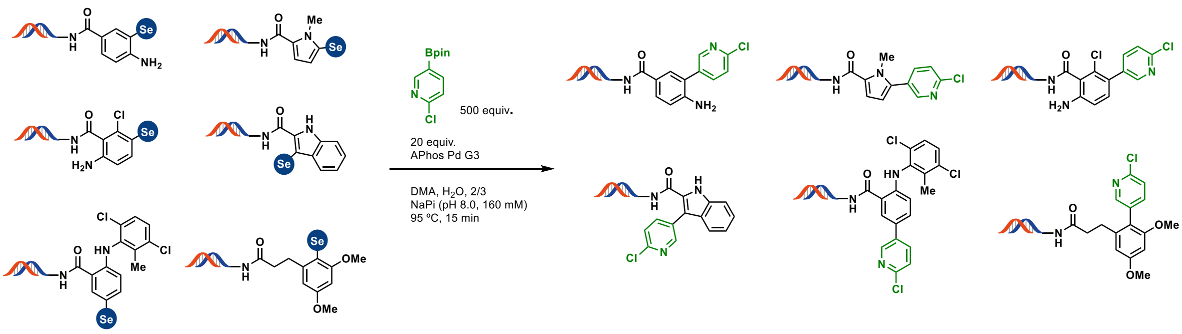

At 20–25 °C, 5.0 µL of the **pool of DNA-conjugated selenonium salts 1** (1.0 mM, 5.0 nmol, 1.0 equiv.) in water was added to a 1.5 mL Eppendorf tube. Next, 10 µL of Phosphate buffer (NaPi, pH 8.0, 500 mM) was added. 5.0 µL of an APhos Pd G3 stock solution (20 mM, 20 nmol, 20 equiv.) in DMA was added over the solution. The mixture was vortexed for 5 seconds. Lastly, 5.0 µL of a 6-chloro-3-pyridineboronic acid pinacol ester stock solution (500 mM, 1000 nmol, 500 equiv.) in DMA was added. The mixture was vortexed for 5 seconds, transferred into a Thermocycler pre-heated at 95 °C, and incubated at 95 °C for 15 min. at 600 rpm. After 15 minutes, 15 µL of a 100 mM solution of sodium diethyldithiocarbamate trihydrate in water were added to remove the palladium salts from the solution. The reaction mixture was centrifuged and 35 µL of the supernatant were collected. Over the supernatant, 4 µL of a 5 M solution of NaCl in water and 120 µL of ethanol at −20 °C were added to precipitate the DNA conjugate. The Eppendorf tube was placed in the freezer (−20 °C) for at least 1 hour, and then it was centrifuged at 4 °C and 10000 x g for at least 30 minutes. The supernatant was removed, and the pellet was redissolved in 150 µL of water for LC–MS analysis. ***All potential Suzuki coupling products were detected in the LC–MS analysis***. Other observed MS signals were corresponding to dehydrofunctionalization byproducts.

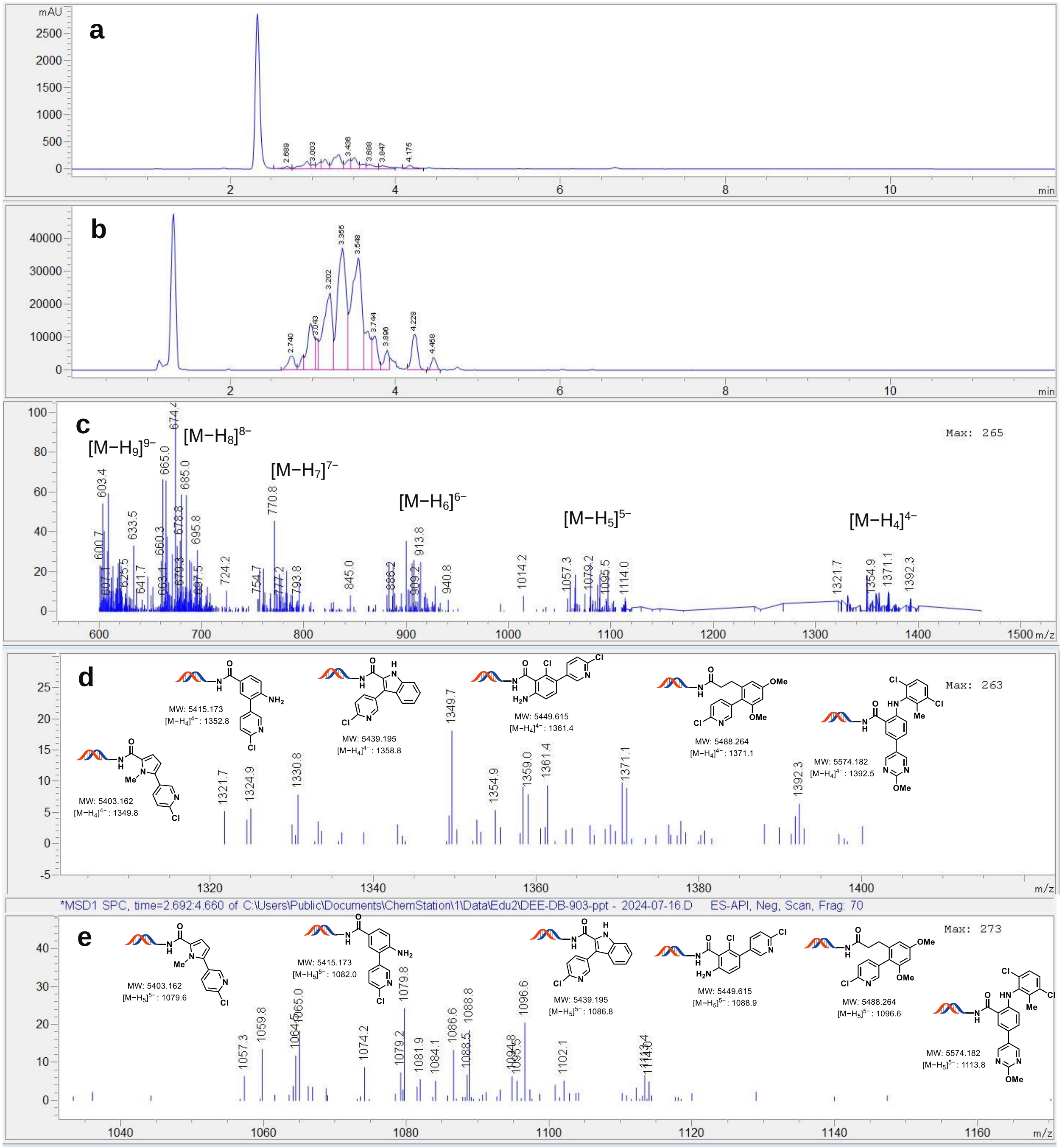

Figure S110. Analytical HPLC trace of the Suzuki coupling of mock library 1, cycle 2 with 6-Chloro-3-pyridineboronic acid pinacol ester with HPLC Method A. (a) DAD chromatogram at 260 nm. (b) TIC chromatogram. (c) Ionization of the full chromatogram. (d) Zoom of the MS spectrum m/z 1300–1500. (e) Zoom of the MS spectrum m/z 1000–1200. *All potential Suzuki coupling products were detected in the LC–MS analysis*.

Mock library 2 – Cycle 1 & 2

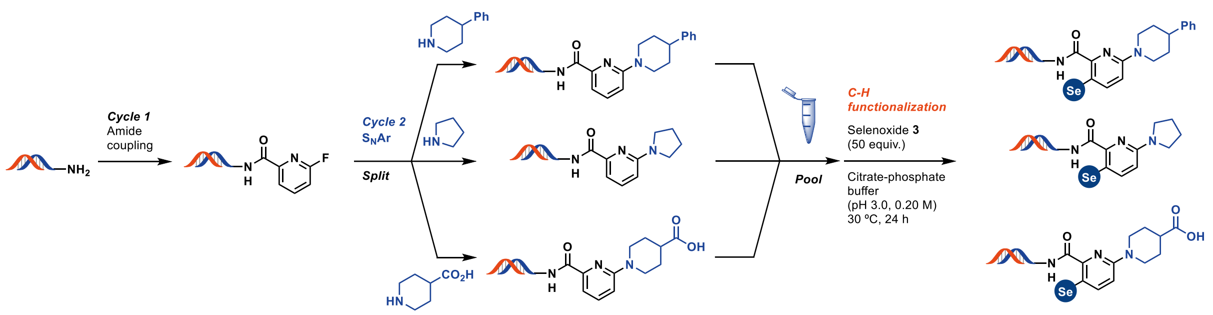

Cycle 1: Amide coupling

DNA-conjugated arene **S32** was synthesized as described in section "Preparation of DNA-conjugated substrates".

Cycle 2: S$_N$Ar

DNA-conjugated arenes **S42**, **S43**, and **S44** were synthesized as described in section "Preparation of DNA-conjugated substrates". Then, 10 µL of each of the DNA-conjugated arenes (2.0 mM, 40 nmol, 1.0 equiv.) were pooled into a 1.5 mL Eppendorf tube, diluted with 300 µL of water and purified by charging the solution in an AMICON® filter unit from Sigma Aldrich (3 kD), centrifuged at 4 °C and 10000 x g for at least 30 minutes, until the volume decreased to < 10 µL. Another 300 µL of water were added and the process was repeated all over again for at least 3 times. The remaining solution concentration was determined by A$_{260}$ absorption using a Thermo Scientific™ NanoDrop™ One^C, concentration of the solution was adjusted to 2.0 mM and used for the next step of the mock library. An aliquot of 0.5 µL of the pool mixture was diluted to 40 µL with water for LC–MS analysis. *All DNA-conjugated arenes were detected*.

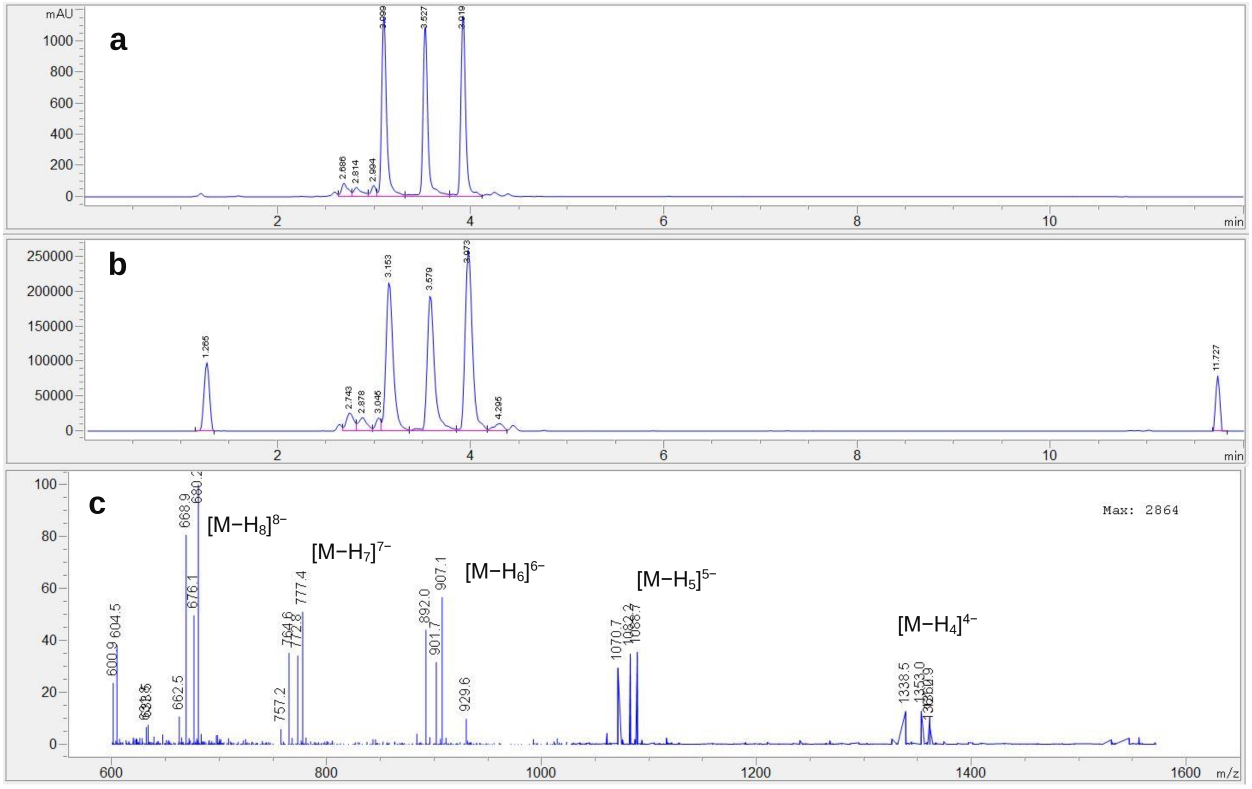

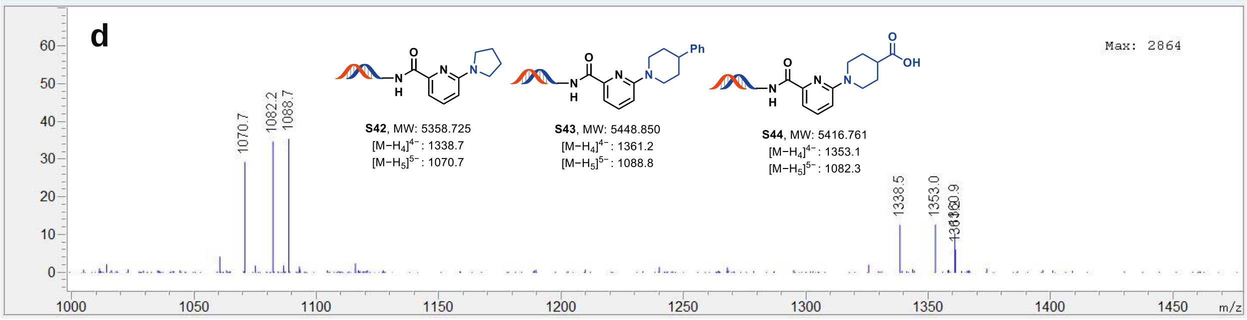

Figure S111. Analytical HPLC trace of the pool of mock library 2, cycle 2 before C−H functionalization with HPLC Method A. (a) DAD chromatogram at 260 nm. (b) TIC chromatogram. (c) Ionization of the full chromatogram. (d) Zoom of the MS spectrum m/z 1000–1500. *All DNA-conjugated arenes were detected*.

Cycle 2: C–H functionalization of mock library 2, cycle 2 pool

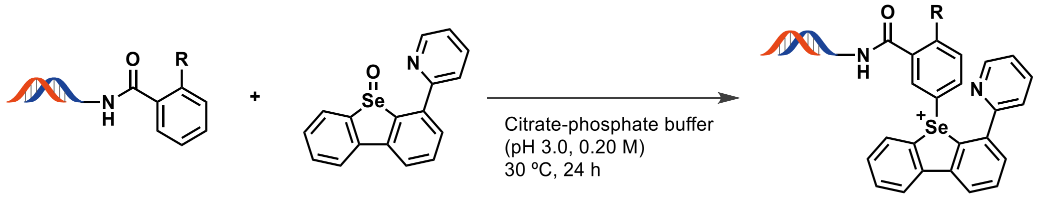

3
50 equiv.

At 20–25 °C, 60 µL of **mock library 2, cycle 2 pool** (2.0 mM, 120 nmol, 1.0 equiv.) in water was added to two 1.5 mL Eppendorf tubes. Next, 540 µL of Citrate-phosphate buffer (pH 3.0, c = 500 mM) was added. Then, 600 µL of a selenoxide **3** stock solution (4.0 mM, 6.00 µmol, 50 equiv.) in water was added. The mixtures were vortexed for 5 seconds, transferred into a Thermocycler pre-heated at 30 °C, and incubated at 30 °C for 24 hours at 600 rpm. After 24 hours, each of the reactions were quenched by addition of 300 µL of borate buffer (pH 9.4, c = 500 mM). The crude reaction was purified by charging the solution in an AMICON® filter unit from Sigma Aldrich (3 kD) in portions of 300 µL of water, centrifuged at 4 °C and 10000 x g for at least 30 minutes, until the volume decreased to < 10 µL. Another 300 µL of water were added and the process was repeated all over again for at least 3 times. The remaining solution concentration was determined by A_{260} absorption using a Thermo Scientific™ NanoDrop™ One^{C}, concentration of the solution was adjusted to 2.0 mM and stored in the freezer at −20 °C. Recovery of the DNA as measured by NanoDrop™ was quantitative (ca. 95%). An aliquot of 1 µL of the reaction mixture was diluted to 40 µL with water for LC–MS analysis. ***All potential C-H functionalization products were detected in the LC–MS analysis***.

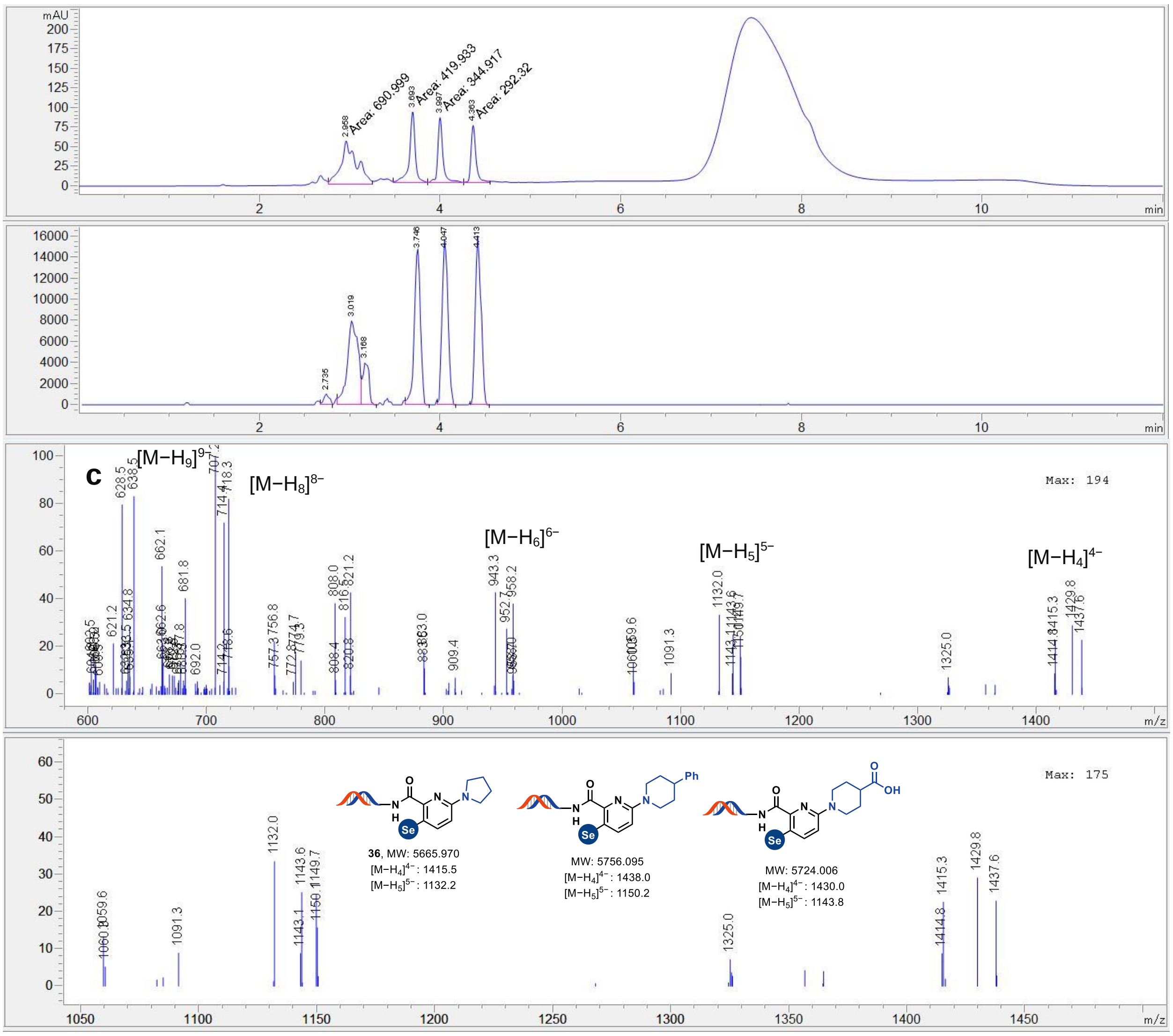

Figure S112. Analytical HPLC trace of the pool of mock library 2, cycle 2 after C−H functionalization with HPLC Method A. (a) DAD chromatogram at 260 nm. (b) TIC chromatogram. (c) Ionization of the full chromatogram. (d) Zoom of the MS spectrum m/z 1000–1500. *All potential C-H functionalization products were detected in the LC–MS analysis*.

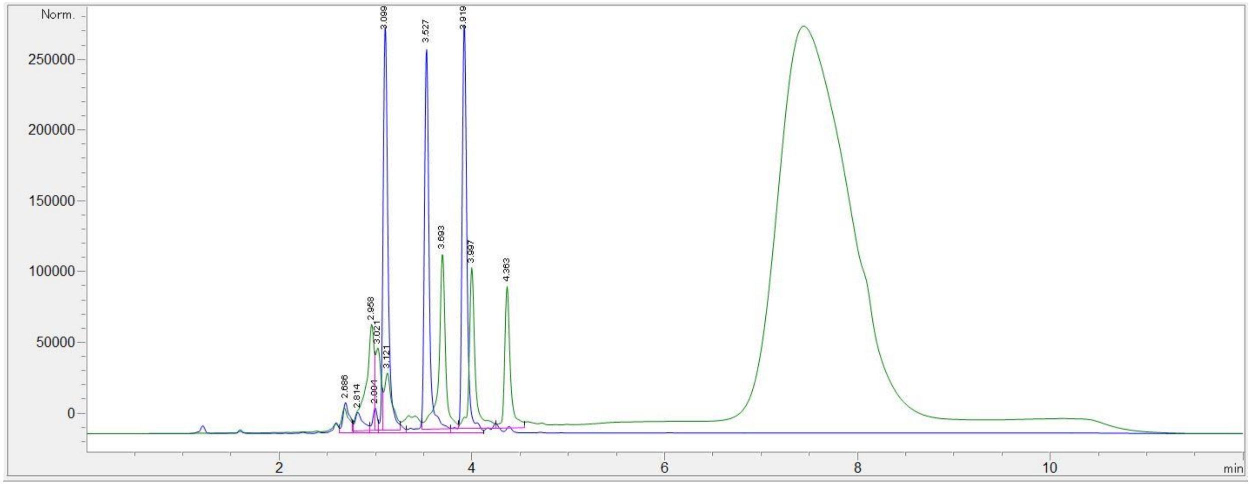

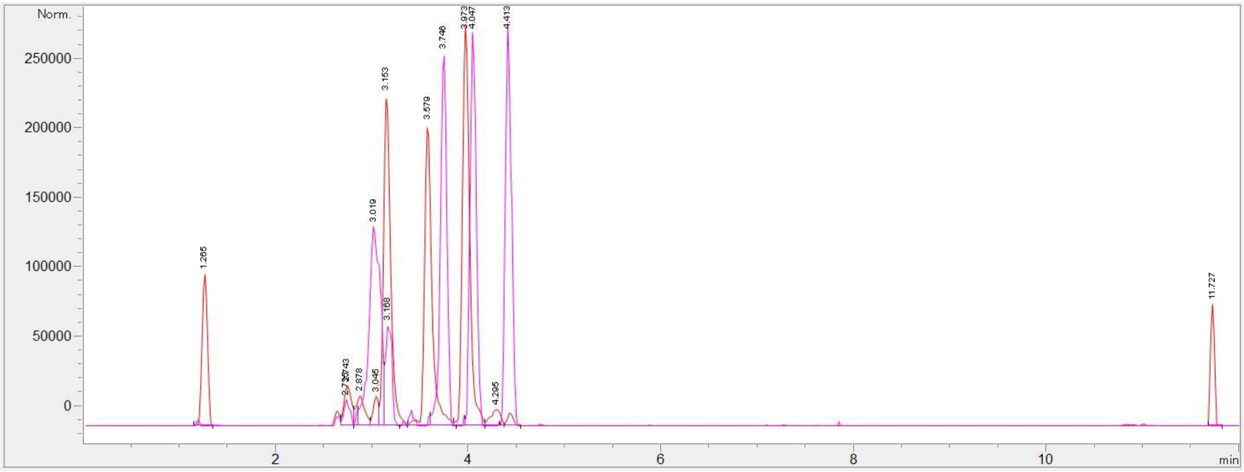

Figure S113. Overlay of analytical HPLC traces of the pool of mock library 2, cycle 2 before and after C−H functionalization with HPLC Method A. (Up) DAD chromatograms at 260 nm, before C−H functionalization (blue), after C−H functionalization (green). (Below) TIC chromatograms, before C−H functionalization (red), after C−H functionalization (pink).

Mock library 2 – Cycle 3

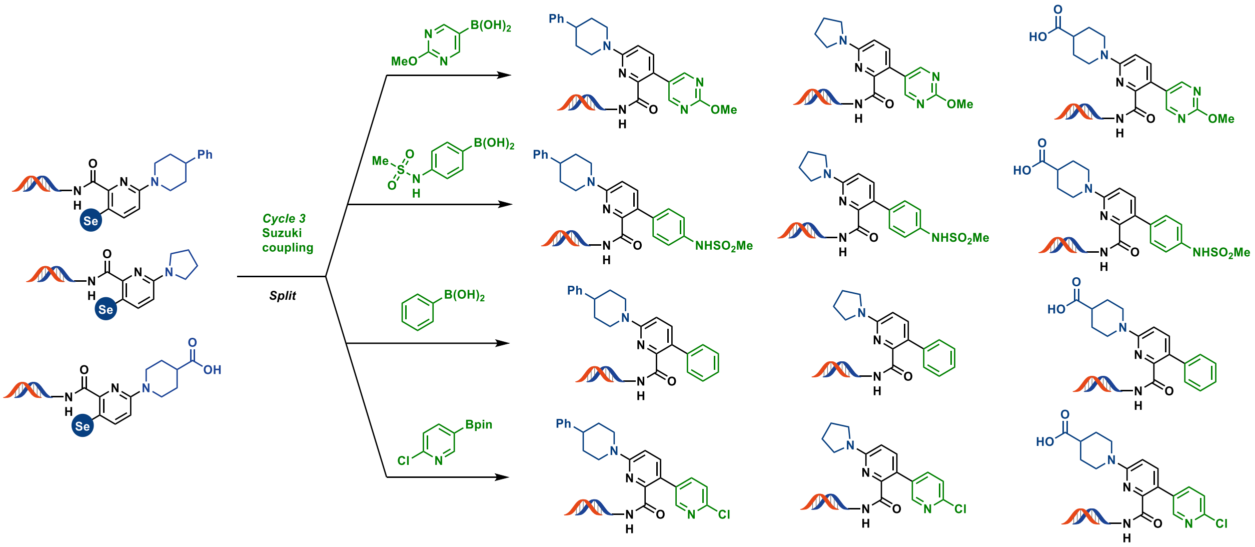

Cycle 3: Suzuki coupling of pool of DNA-conjugated selenonium salts 2 with 2-methoxypyrimidine-5-boronic acid

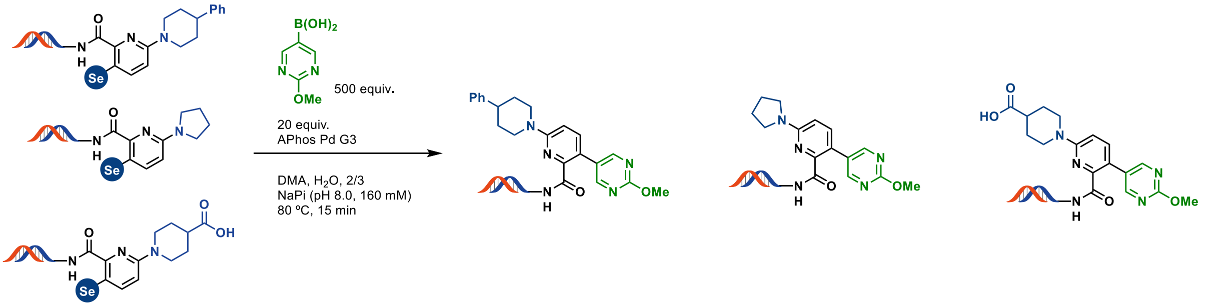

At 20–25 °C, 5.0 µL of the **pool of DNA-conjugated selenonium salts 2** (1.0 mM, 5.0 nmol, 1.0 equiv.) in water was added to a 1.5 mL Eppendorf tube. Next, 10 µL of Phosphate buffer (NaPi, pH 8.0, 500 mM) was added. 5.0 µL of an APhos Pd G3 stock solution (20 mM, 20 nmol, 20 equiv.) in DMA was added over the solution. The mixture was vortexed for 5 seconds. Lastly, 5.0 µL of a 2-methoxypyrimidine-5-

boronic acid stock solution (500 mM, 1000 nmol, 500 equiv.) in DMA was added. The mixture was vortexed for 5 seconds, transferred into a Thermocycler pre-heated at 80 °C, and incubated at 80 °C for 15 min. at 600 rpm. After 15 minutes, 15 µL of a 100 mM solution of sodium diethyldithiocarbamate trihydrate in water were added to remove the palladium salts from the solution. The reaction mixture was centrifuged and 35 µL of the supernatant were collected. Over the supernatant, 4 µL of a 5 M solution of NaCl in water and 120 µL of ethanol at −20 °C were added to precipitate the DNA conjugate. The Eppendorf tube was placed in the freezer (−20 °C) for at least 1 hour, and then it was centrifuged at 4 °C and 10000 x g for at least 30 minutes. The supernatant was removed, and the pellet was redissolved in 150 µL of water for LC–MS analysis. ***All potential Suzuki coupling products were detected in the LC–MS analysis***. *Detected MS signals included sodium aggregates e.g. $[M{-}H_6{+}Na^+]^{5-}$ and $[M{-}H_5{+}Na^+]^{4-}$.* Other observed MS signals were corresponding to dehydrofunctionalization byproducts.

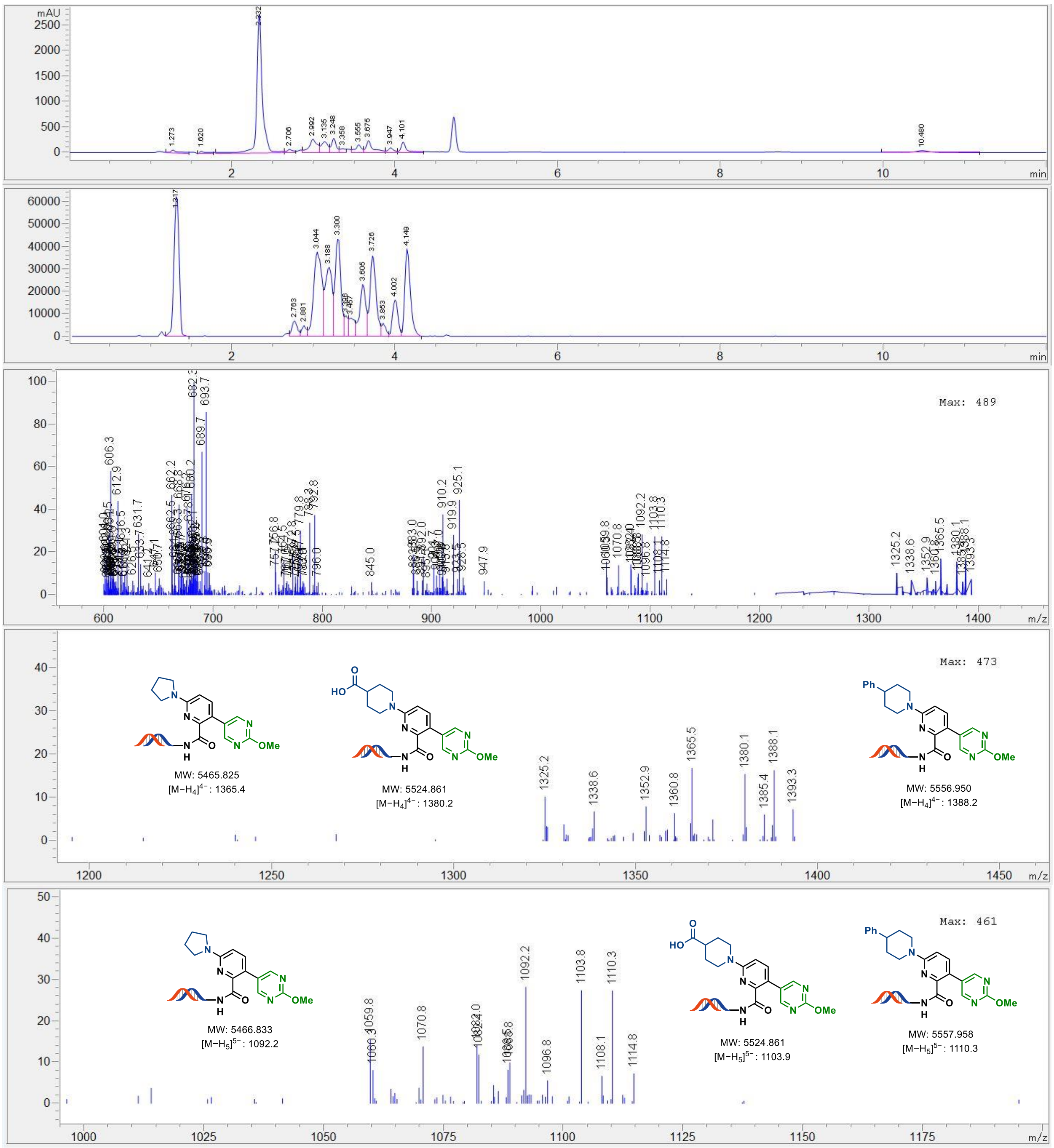

Figure S114. Analytical HPLC trace of the Suzuki coupling of mock library 2, cycle 3 with 2-

methoxypyrimidine-5-boronic acid with HPLC Method A. (a) DAD chromatogram at 260 nm. (b) TIC chromatogram. (c) Ionization of the full chromatogram. (d) Zoom of the MS spectrum m/z 1200–1500. (e) Zoom of the MS spectrum m/z 1000–1200. *All potential Suzuki coupling products were detected in the LC–MS analysis*.

Cycle 3: Suzuki coupling of pool of DNA-conjugated selenonium salts 2 with (4-(methylsulfonamido)phenyl)boronic acid

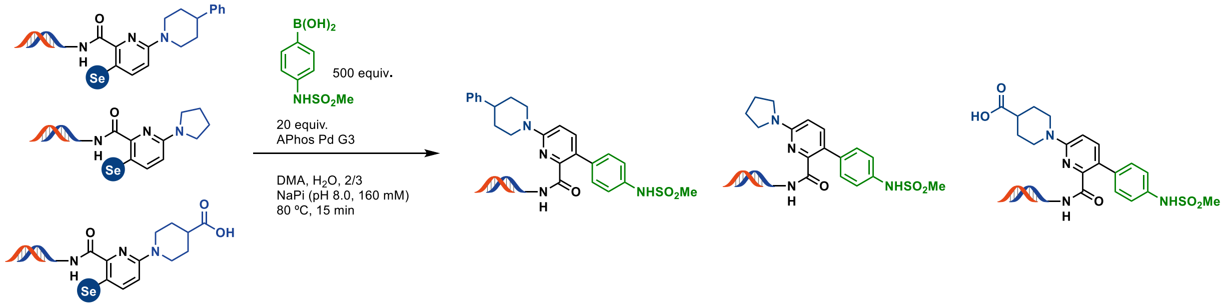

At 20–25 °C, 5.0 µL of the **pool of DNA-conjugated selenonium salts 2** (1.0 mM, 5.0 nmol, 1.0 equiv.) in water was added to a 1.5 mL Eppendorf tube. Next, 10 µL of Phosphate buffer (NaPi, pH 8.0, 500 mM) was added. 5.0 µL of an APhos Pd G3 stock solution (20 mM, 20 nmol, 20 equiv.) in DMA was added over the solution. The mixture was vortexed for 5 seconds. Lastly, 5.0 µL of a (4-(methylsulfonamido)phenyl)boronic acid stock solution (500 mM, 1000 nmol, 500 equiv.) in DMA was added. The mixture was vortexed for 5 seconds, transferred into a Thermocycler pre-heated at 80 °C, and incubated at 80 °C for 15 min. at 600 rpm. After 15 minutes, 15 µL of a 100 mM solution of sodium diethyldithiocarbamate trihydrate in water were added to remove the palladium salts from the solution. The reaction mixture was centrifuged and 35 µL of the supernatant were collected. Over the supernatant, 4 µL of a 5 M solution of NaCl in water and 120 µL of ethanol at −20 °C were added to precipitate the DNA conjugate. The Eppendorf tube was placed in the freezer (−20 °C) for at least 1 hour, and then it was centrifuged at 4 °C and 10000 x g for at least 30 minutes. The supernatant was removed, and the pellet was redissolved in 150 µL of water for LC–MS analysis. ***All potential Suzuki coupling products were detected in the LC–MS analysis.*** Other observed MS signals were corresponding to dehydrofunctionalization byproducts.

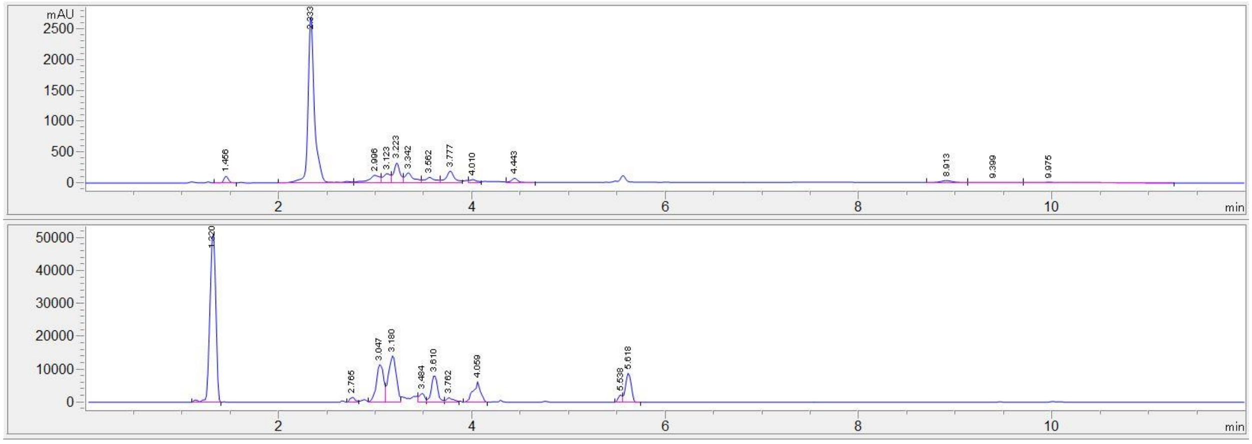

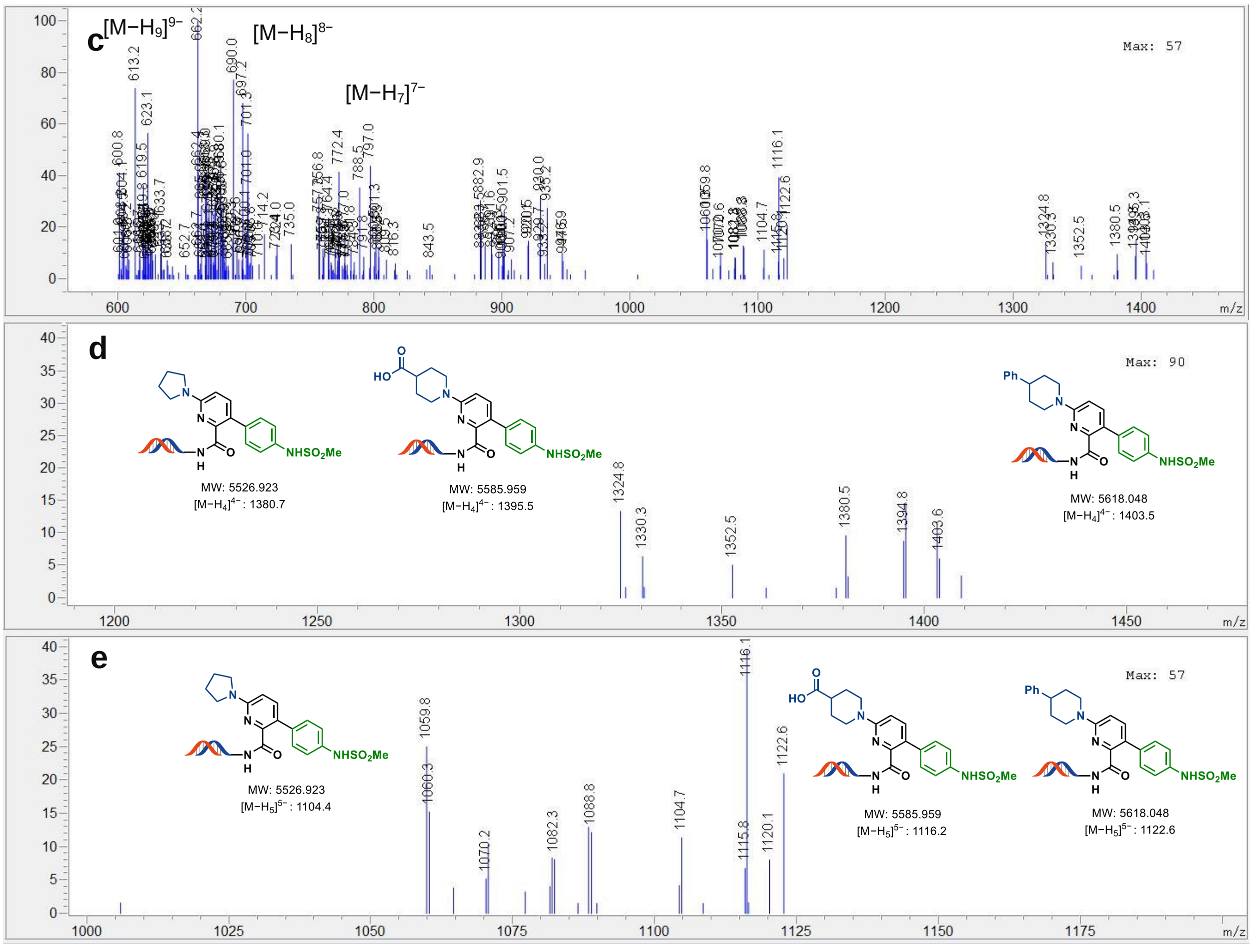

Figure S115. Analytical HPLC trace of the Suzuki coupling of mock library 2, cycle 3 with (4-(methylsulfonamido)phenyl)boronic acid with HPLC Method A. (a) DAD chromatogram at 260 nm. (b) TIC chromatogram. (c) Ionization of the full chromatogram. (d) Zoom of the MS spectrum m/z 1200–1500. (e) Zoom of the MS spectrum m/z 1000–1200. *All potential Suzuki coupling products were detected in the LC–MS analysis*.

Cycle 3: Suzuki coupling of pool of DNA-conjugated selenonium salts 2 with phenylboronic acid

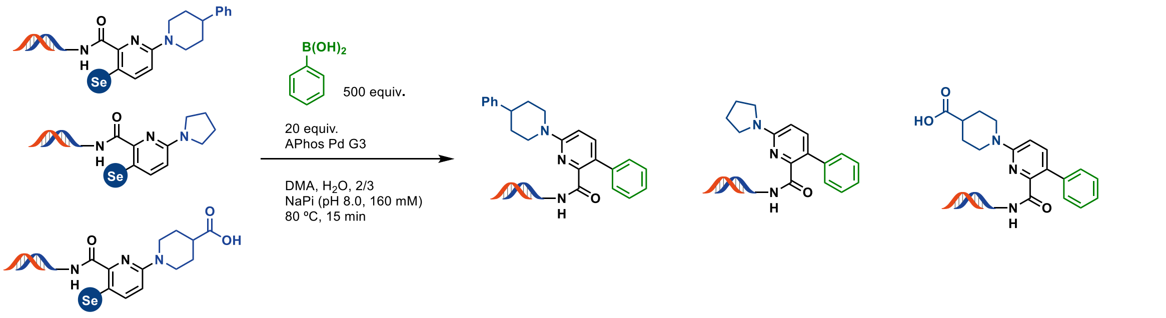

At 20–25 °C, 5.0 µL of the **pool of DNA-conjugated selenonium salts 2** (1.0 mM, 5.0 nmol, 1.0 equiv.) in water was added to a 1.5 mL Eppendorf tube. Next, 10 µL of Phosphate buffer (NaPi, pH 8.0, 500 mM) was added. 5.0 µL of an APhos Pd G3 stock solution (20 mM, 20 nmol, 20 equiv.) in DMA was added over the solution. The mixture was vortexed for 5 seconds. Lastly, 5.0 µL of a phenylboronic acid stock solution (500 mM, 1000 nmol, 500 equiv.) in DMA was added. The mixture was vortexed for 5 seconds, transferred into a Thermocycler pre-heated at 80 °C, and incubated at 80 °C for 15 min. at 600 rpm. After 15 minutes, 15 µL of a 100 mM solution of sodium diethyldithiocarbamate trihydrate in water were added to remove the palladium salts from the solution. The reaction mixture was centrifuged and 35 µL of the

supernatant were collected. Over the supernatant, 4 µL of a 5 M solution of NaCl in water and 120 µL of ethanol at −20 °C were added to precipitate the DNA conjugate. The Eppendorf tube was placed in the freezer (−20 °C) for at least 1 hour, and then it was centrifuged at 4 °C and 10000 x g for at least 30 minutes. The supernatant was removed, and the pellet was redissolved in 150 µL of water for LC–MS analysis. ***All potential Suzuki coupling products were detected in the LC–MS analysis.*** *Detected MS signals included sodium aggregates e.g.* $[M-H_6+Na^+]^{5-}$ *and* $[M-H_5+Na^+]^{4-}$. Other observed MS signals were corresponding to dehydrofunctionalization byproducts.

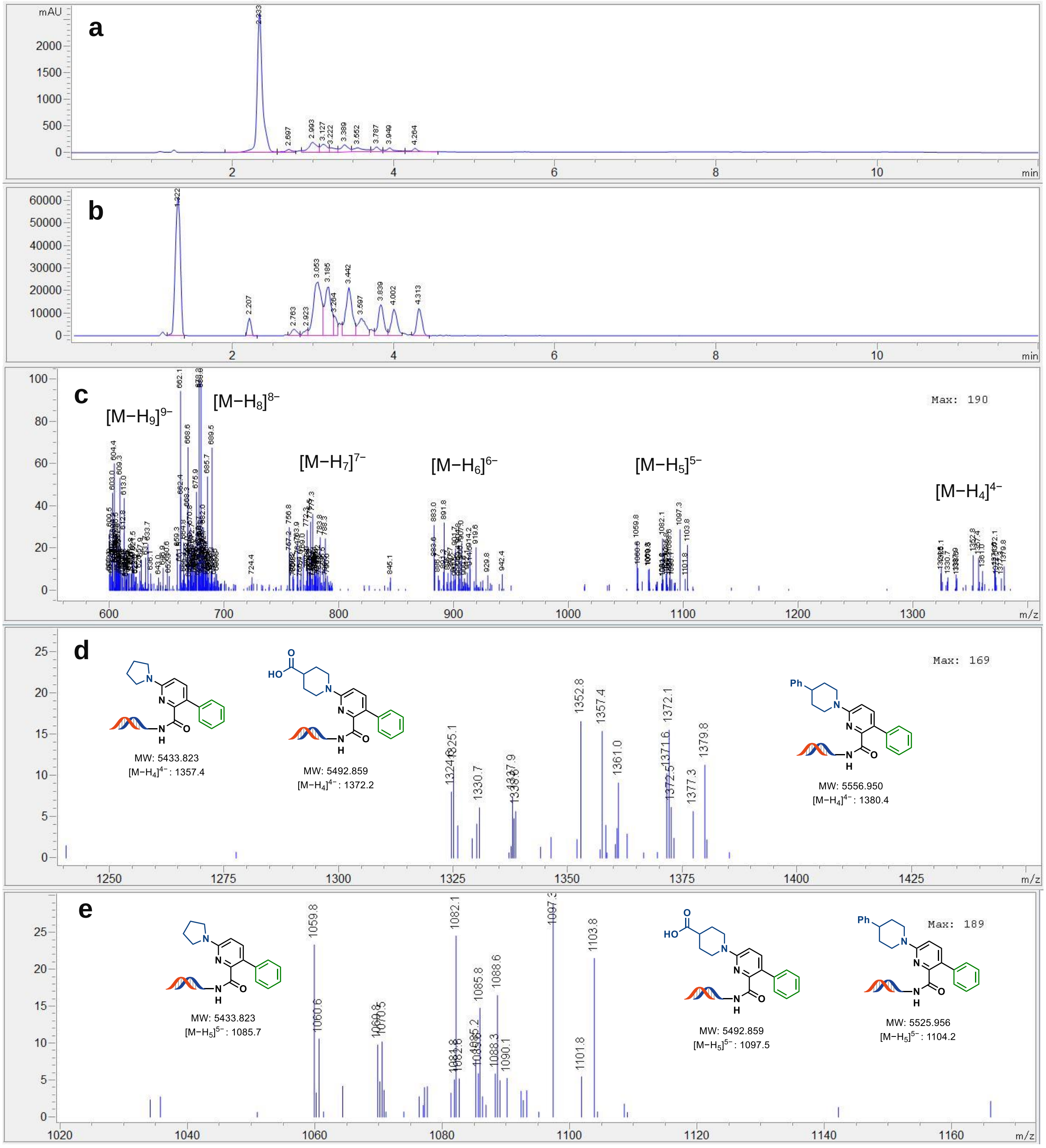

Figure S116. Analytical HPLC trace of the Suzuki coupling of mock library 2, cycle 3 with phenylboronic acid with HPLC Method A. (a) DAD chromatogram at 260 nm. (b) TIC chromatogram. (c) Ionization of the full chromatogram. (d) Zoom of the MS spectrum m/z 1200–1500. (e) Zoom of the MS spectrum m/z 1000–1200. *All potential Suzuki coupling products were detected in the LC–MS analysis*.

Cycle 3: Suzuki coupling of pool of DNA-conjugated selenonium salts 2 with 6-chloro-3-pyridineboronic acid pinacol ester

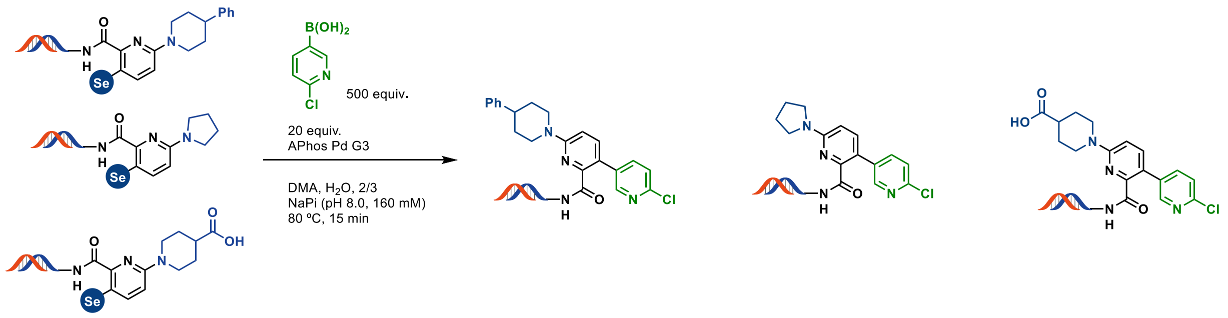

At 20–25 °C, 5.0 µL of the **pool of DNA-conjugated selenonium salts 2** (1.0 mM, 5.0 nmol, 1.0 equiv.) in water was added to a 1.5 mL Eppendorf tube. Next, 10 µL of Phosphate buffer (NaPi, pH 8.0, 500 mM) was added. 5.0 µL of an APhos Pd G3 stock solution (20 mM, 20 nmol, 20 equiv.) in DMA was added over the solution. The mixture was vortexed for 5 seconds. Lastly, 5.0 µL of a 6-chloro-3-pyridineboronic acid pinacol ester stock solution (500 mM, 1000 nmol, 500 equiv.) in DMA was added. The mixture was vortexed for 5 seconds, transferred into a Thermocycler pre-heated at 80 °C, and incubated at 80 °C for 15 min. at 600 rpm. After 15 minutes, 15 µL of a 100 mM solution of sodium diethyldithiocarbamate trihydrate in water were added to remove the palladium salts from the solution. The reaction mixture was centrifuged and 35 µL of the supernatant were collected. Over the supernatant, 4 µL of a 5 M solution of NaCl in water and 120 µL of ethanol at −20 °C were added to precipitate the DNA conjugate. The Eppendorf tube was placed in the freezer (−20 °C) for at least 1 hour, and then it was centrifuged at 4 °C and 10000 x g for at least 30 minutes. The supernatant was removed, and the pellet was redissolved in 150 µL of water for LC–MS analysis. ***All potential Suzuki coupling products were detected in the LC–MS analysis.*** *Detected MS signals included sodium aggregates e.g. $[M{-}H_6{+}Na^+]^{5-}$ and $[M{-}H_5{+}Na^+]^{4-}$.* Other observed MS signals were corresponding to dehydrofunctionalization byproducts.

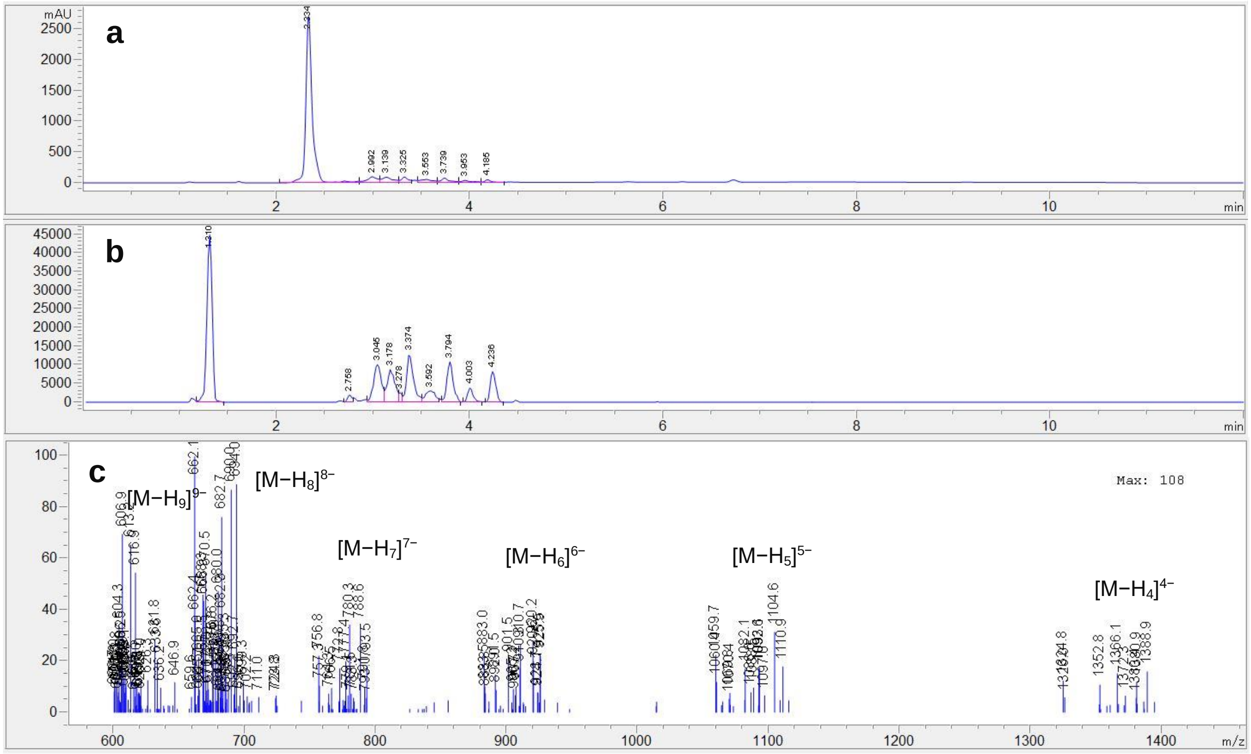

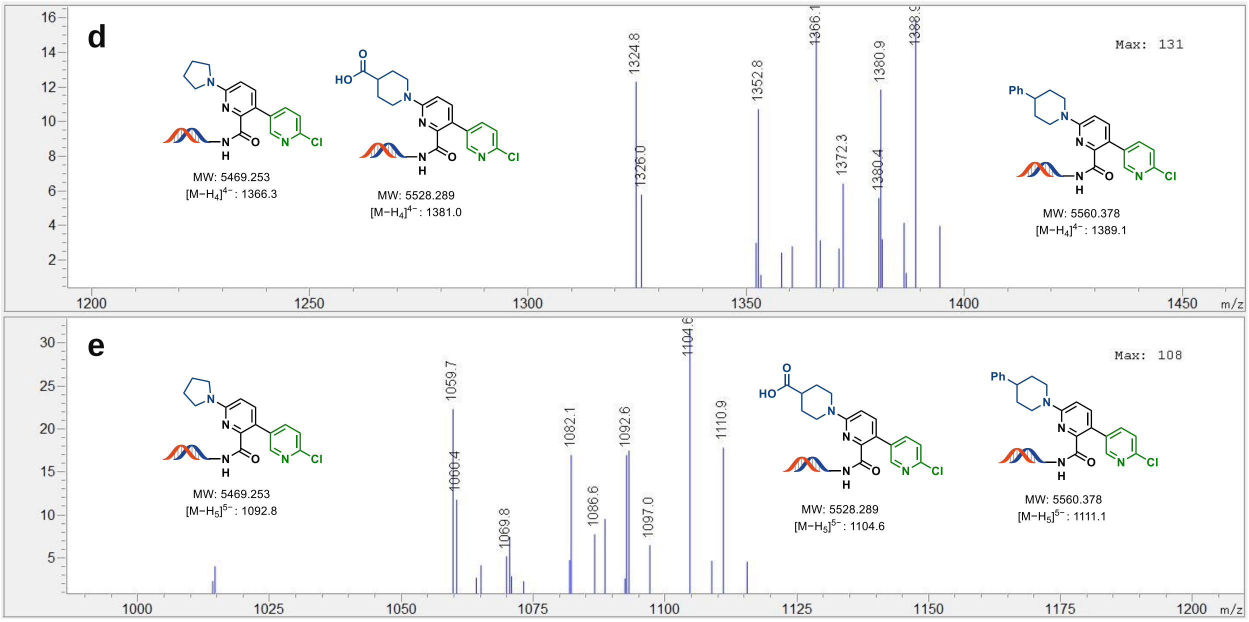

Figure S117. Analytical HPLC trace of the Suzuki coupling of mock library 2, cycle 3 with 6-chloro-3-pyridineboronic acid pinacol ester with HPLC Method A. (a) DAD chromatogram at 260 nm. (b) TIC chromatogram. (c) Ionization of the full chromatogram. (d) Zoom of the MS spectrum m/z 1200–1500. (e) Zoom of the MS spectrum m/z 1000–1200. *All potential Suzuki coupling products were detected in the LC–MS analysis*.

DNA stability tests

DNA stability under acidic conditions

To evaluate DNA stability under C−H functionalization conditions, we subjected a sample HP–AOP–NH_2 **S4** to varying pH levels and reagent concentrations, then analyzed it by LC–MS at different time intervals. DNA fragmentation under acidic conditions, indicated by the loss of one or more nucleobases, can be detectable through LC–MS analysis[5].

C−H functionalization of DNA-conjugated arenes at pH 3.5

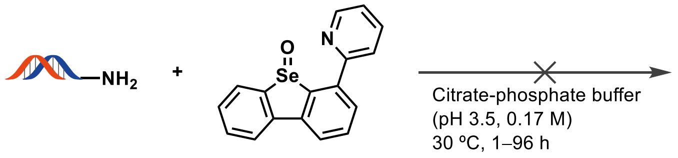

S4

3
2 equiv.

At 20–25 °C, 1.0 µL of **S4** (2.0 mM, 2.0 nmol, 1.0 equiv.) in water was added to a 1.5 mL Eppendorf tube. Next, 1.0 µL of Citrate-phosphate buffer (pH 3.5, c = 500 mM) was added. Then, 1.0 µL of a selenoxide **3** stock solution (4.0 mM, 4.0 nmol, 2.0 equiv.) in water was added. The mixture was vortexed for 5 seconds, transferred into a Thermocycler pre-heated at 30 °C, and incubated at 30 °C for 96 hours at 600 rpm. After the indicated time for each sample, an aliquot of 0.5 µL of the reaction mixture was diluted to 40 µL with water for LC–MS analysis.

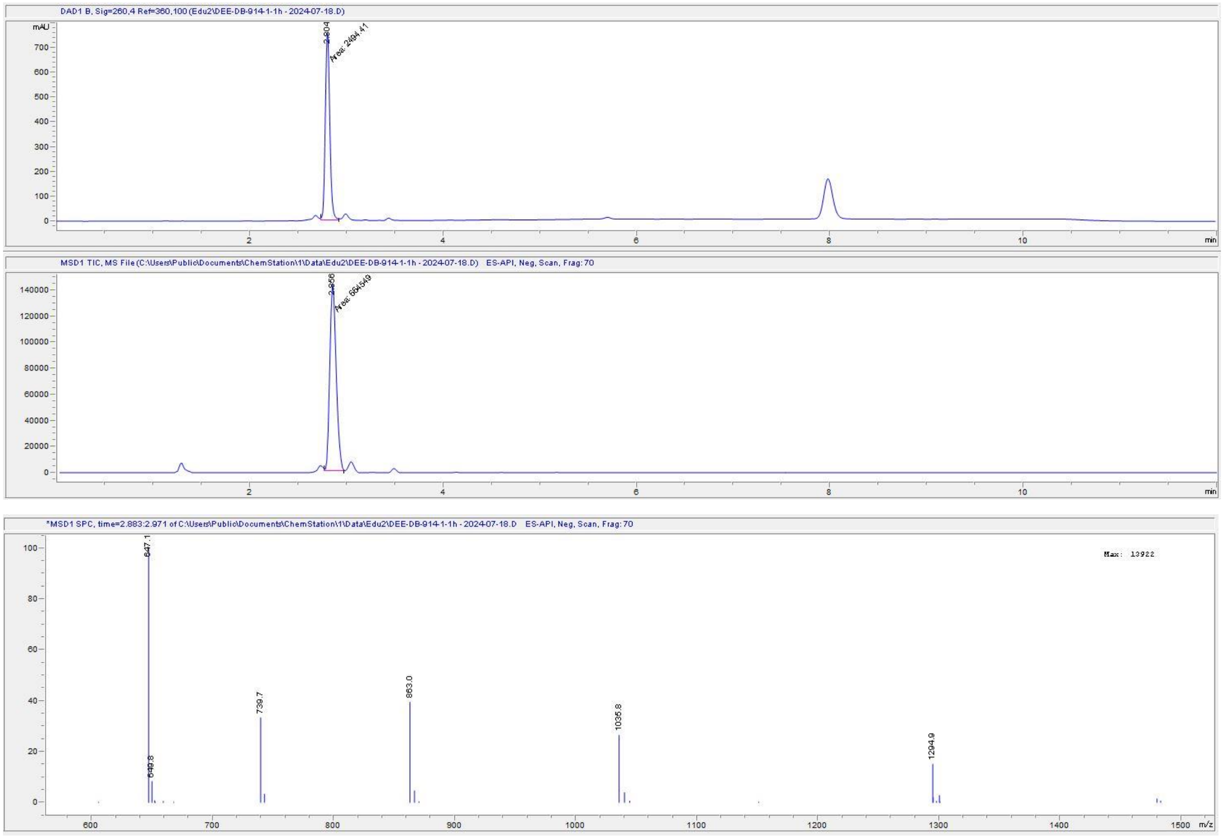

Figure S118. Analytical HPLC trace of C−H functionalization of **S4** at pH 3.5 and 2 equiv. of Selenoxide **3** after 1 h with HPLC Method A. (Up) DAD chromatogram at 260 nm. (Middle) TIC chromatogram. (Below) Ionization of peak at 2.856 min containing **S4**. No observable DNA damage.

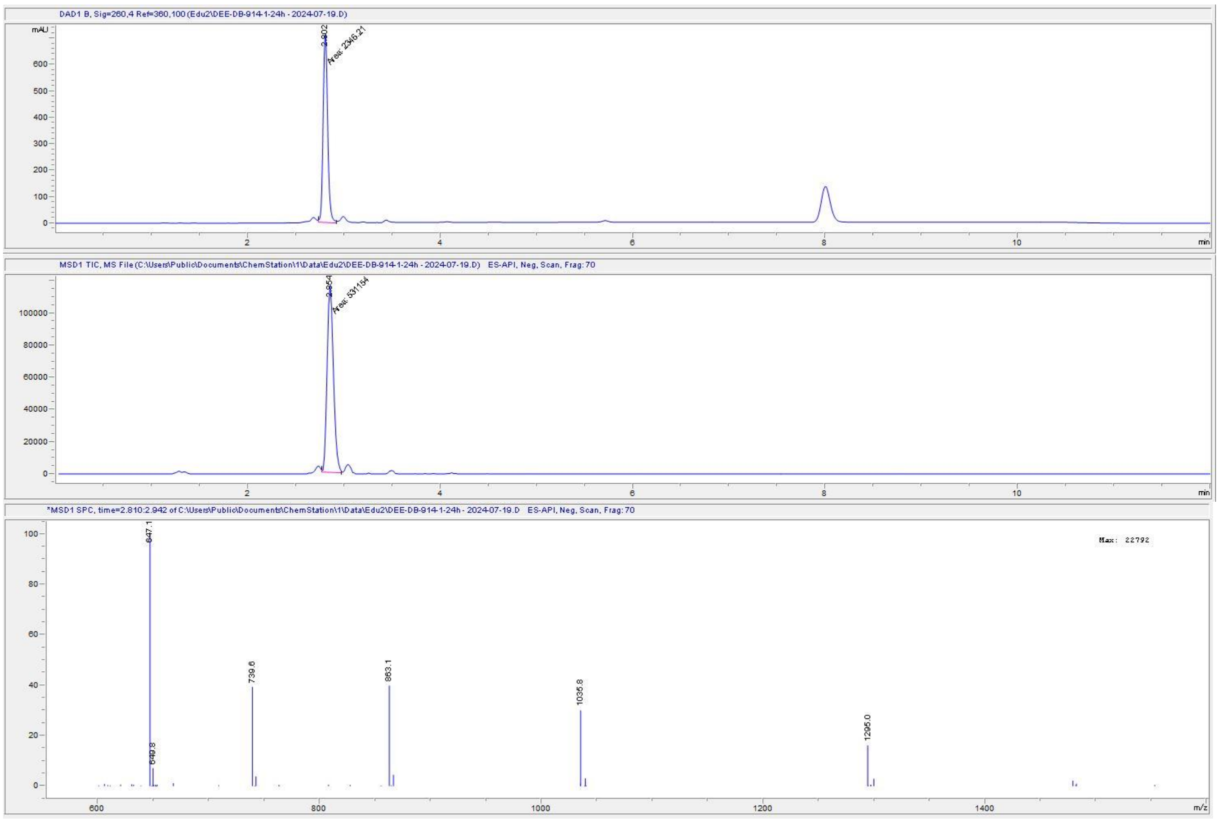

Figure S119. Analytical HPLC trace of C−H functionalization of **S4** at pH 3.5 and 2 equiv. of Selenoxide **3** after 24 h with HPLC Method A. (Up) DAD chromatogram at 260 nm. (Middle) TIC chromatogram.

(Below) Ionization of peak at 2.854 min containing **S4**. No observable DNA damage.

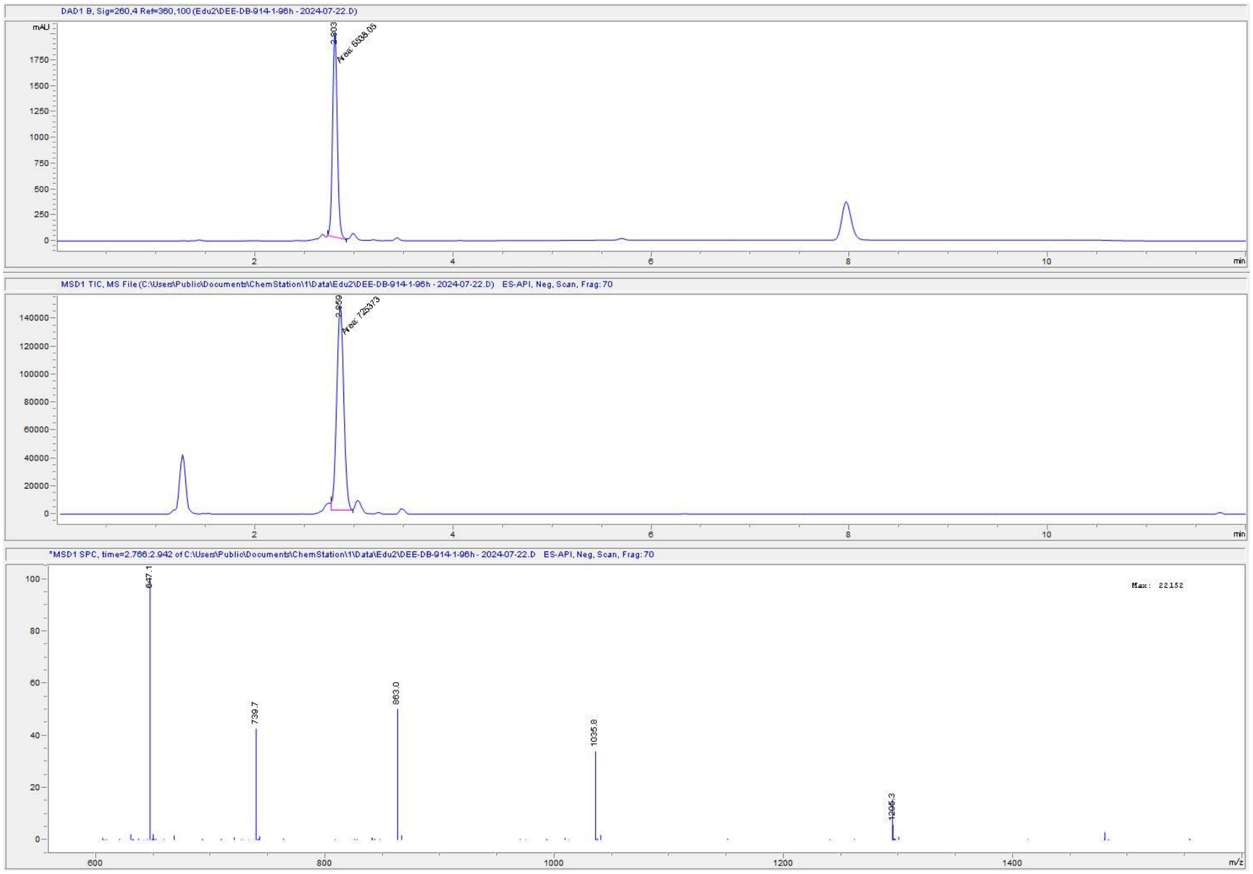

Figure S120. Analytical HPLC trace of C−H functionalization of **S4** at pH 3.5 and 2 equiv. of Selenoxide **3** after 96 h with HPLC Method A. (Up) DAD chromatogram at 260 nm. (Middle) TIC chromatogram. (Below) Ionization of peak at 2.859 min containing **S4**. No observable DNA damage.

C−H functionalization of DNA-conjugated arenes at pH 3.5 and 10 equiv. of Selenoxide 3

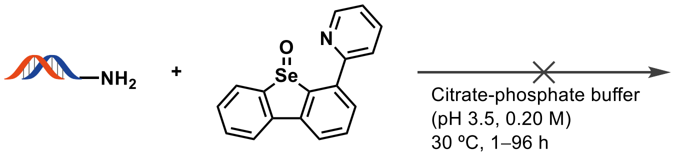

S4

3
10 equiv.

At 20–25 °C, 1.0 µL of **S4** (2.0 mM, 2.0 nmol, 1.0 equiv.) in water was added to a 1.5 mL Eppendorf tube. Next, 4.0 µL of Citrate-phosphate buffer (pH 3.5, c = 500 mM) was added. Then, 5.0 µL of a selenoxide **3** stock solution (4.0 mM, 20 nmol, 10 equiv.) in water was added. The mixture was vortexed for 5 seconds, transferred into a Thermocycler pre-heated at 30 °C, and incubated at 30 °C for 96 hours at 600 rpm. After the indicated time for each sample, an aliquot of 0.5 µL of the reaction mixture was diluted to 40 µL with water for LC–MS analysis.

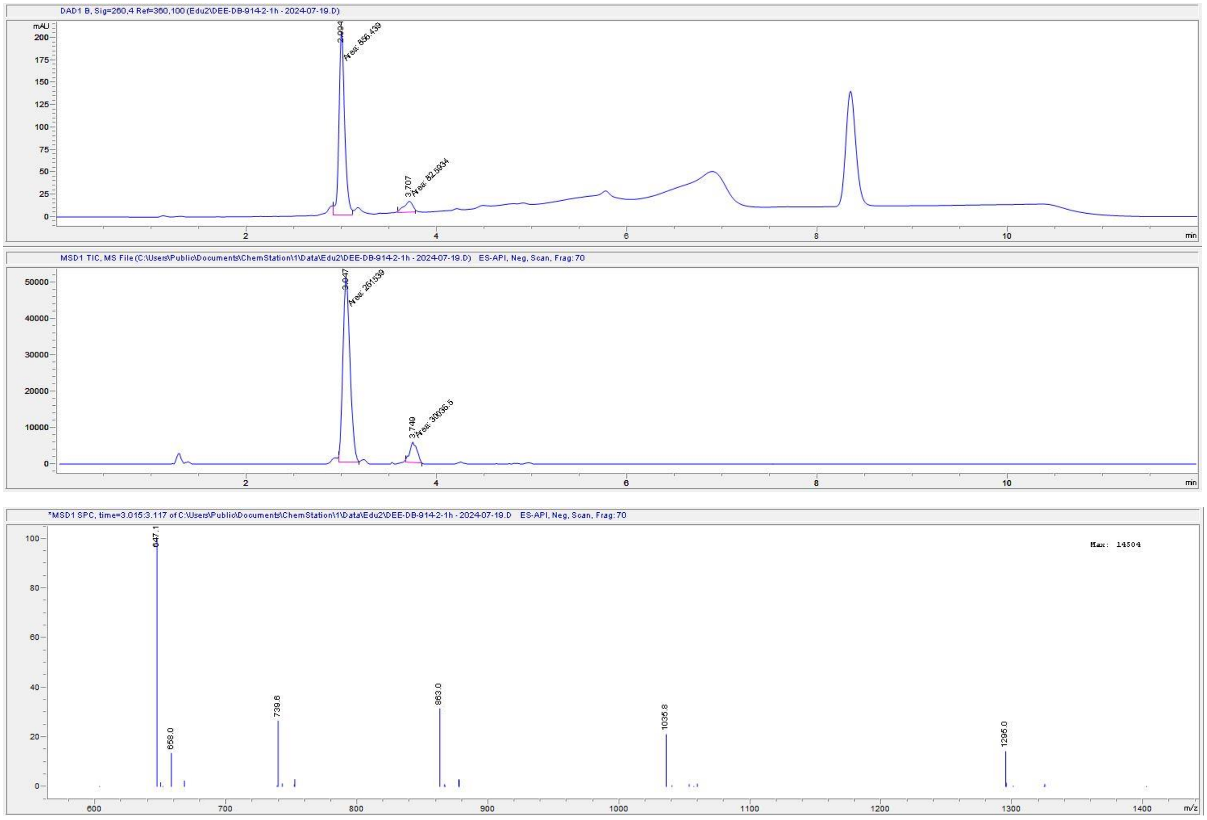

Figure S121. Analytical HPLC trace of C−H functionalization of **S4** at pH 3.5 and 10 equiv. of Selenoxide **3** after 1 h with HPLC Method A. (Up) DAD chromatogram at 260 nm. (Middle) TIC chromatogram. (Below) Ionization of peak at 3.047 min containing **S4**. No observable DNA damage.

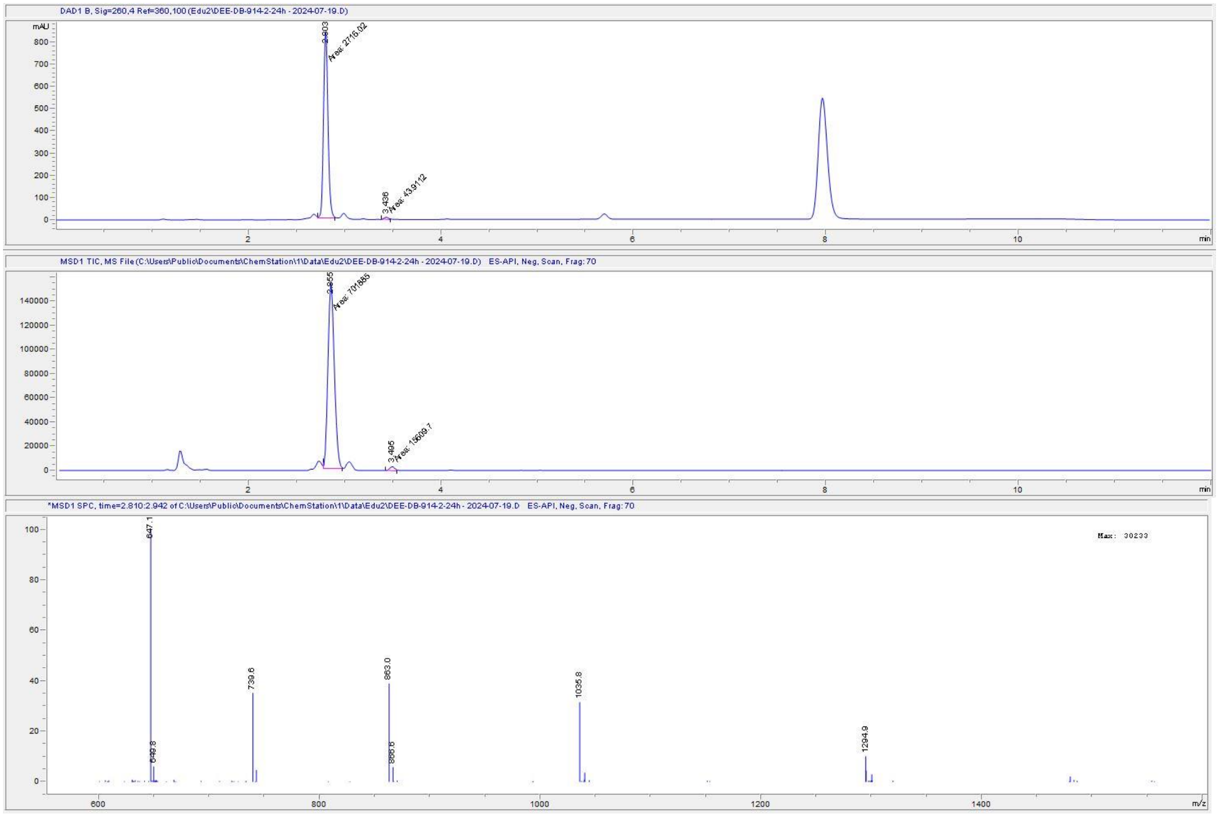

Figure S122. Analytical HPLC trace of C−H functionalization of **S4** at pH 3.5 and 10 equiv. of Selenoxide **3** after 24 h with HPLC Method A. (Up) DAD chromatogram at 260 nm. (Middle) TIC chromatogram. (Below) Ionization of peak at 2.855 min containing **S4**. No observable DNA damage.

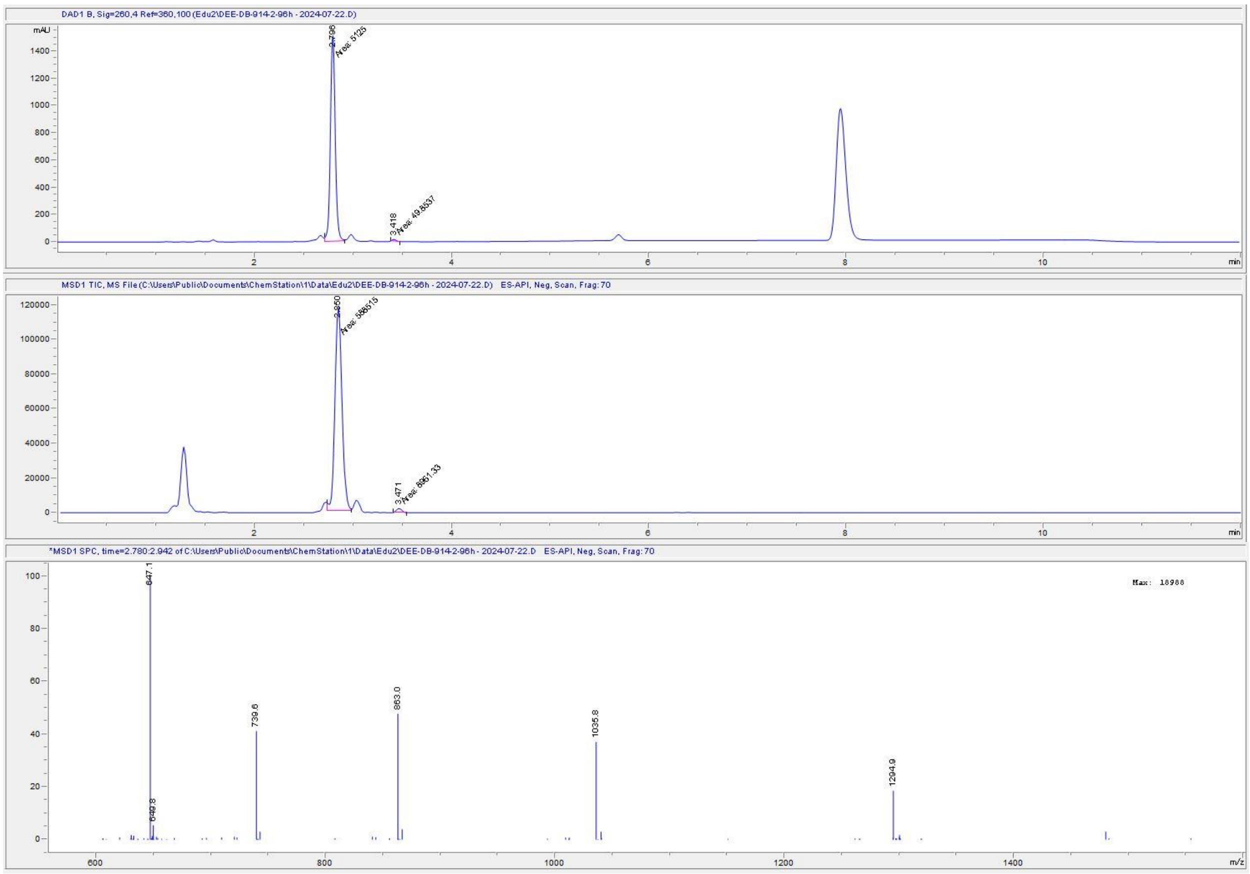

Figure S123. Analytical HPLC trace C−H functionalization of **S4** at pH 3.5 and 10 equiv. of Selenoxide **3** after 96 h with HPLC Method A. (Up) DAD chromatogram at 260 nm. (Middle) TIC chromatogram. (Below) Ionization of peak at 2.850 min containing **S4**. No observable DNA damage.

C−H functionalization of DNA-conjugated arenes at pH 3

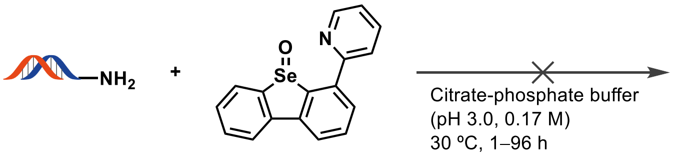

S4 **3** 2 equiv.

At 20–25 °C, 1.0 µL of **S4** (2.0 mM, 2.0 nmol, 1.0 equiv.) in water was added to a 1.5 mL Eppendorf tube. Next, 1.0 µL of Citrate-phosphate buffer (pH 3.0, c = 500 mM) was added. Then, 1.0 µL of a selenoxide **3** stock solution (4.0 mM, 4.0 nmol, 2.0 equiv.) in water was added. The mixture was vortexed for 5 seconds, transferred into a Thermocycler pre-heated at 30 °C, and incubated at 30 °C for 96 hours at 600 rpm. After the indicated time for each sample, an aliquot of 0.5 µL of the reaction mixture was diluted to 40 µL with water for LC–MS analysis.

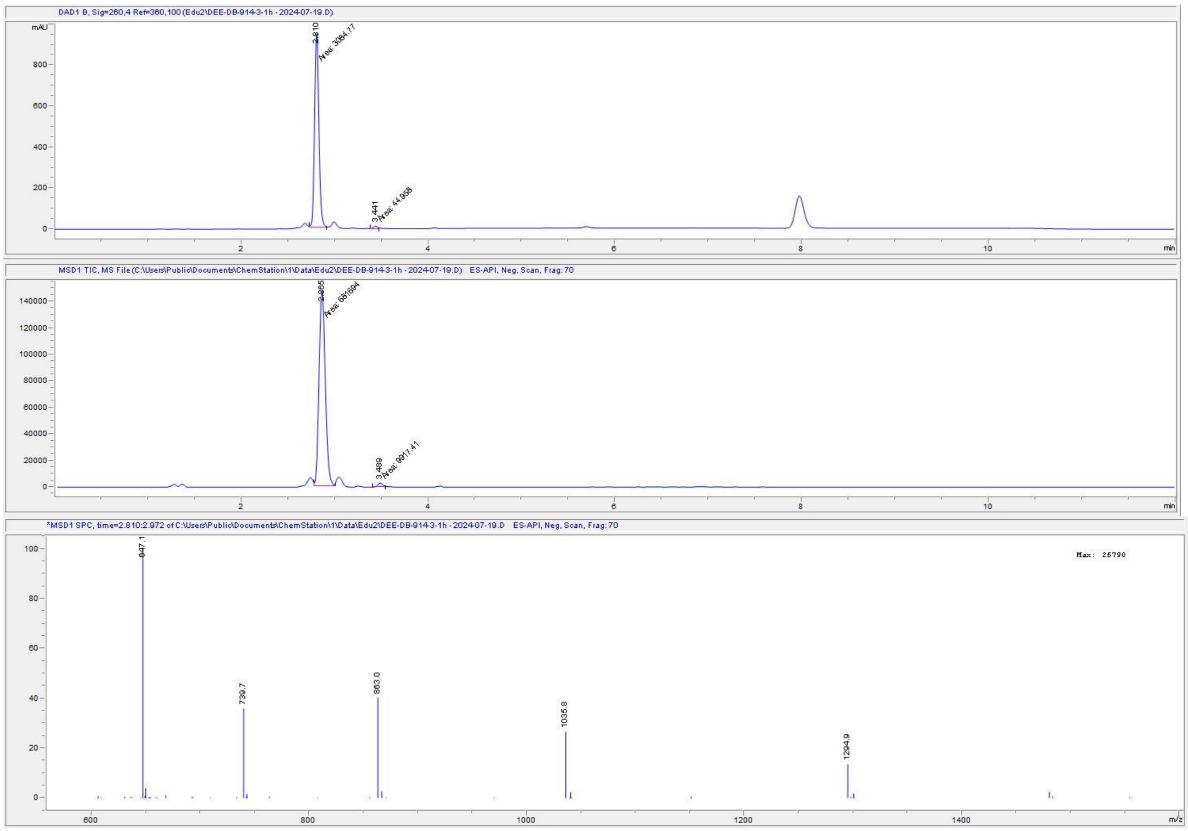

Figure S124. Analytical HPLC trace of C−H functionalization of **S4** at pH 3.0 and 2 equiv. of Selenoxide **3** after 1 h with HPLC Method A. (Up) DAD chromatogram at 260 nm. (Middle) TIC chromatogram. (Below) Ionization of peak at 2.865 min containing **S4**. No observable DNA damage.

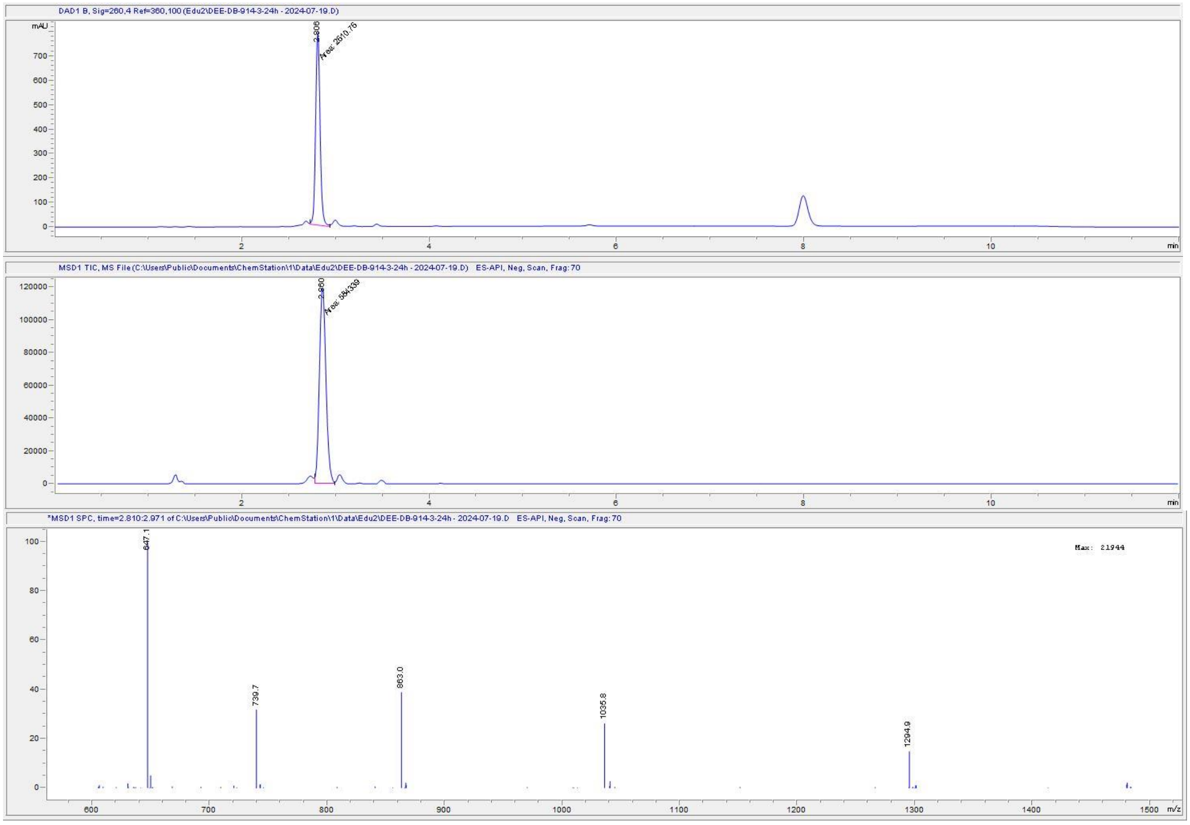

Figure S125. Analytical HPLC trace of C−H functionalization of **S4** at pH 3.0 and 2 equiv. of Selenoxide **3** after 24 h with HPLC Method A. (Up) DAD chromatogram at 260 nm. (Middle) TIC chromatogram. (Below) Ionization of peak at 2.860 min containing **S4**. No observable DNA damage.

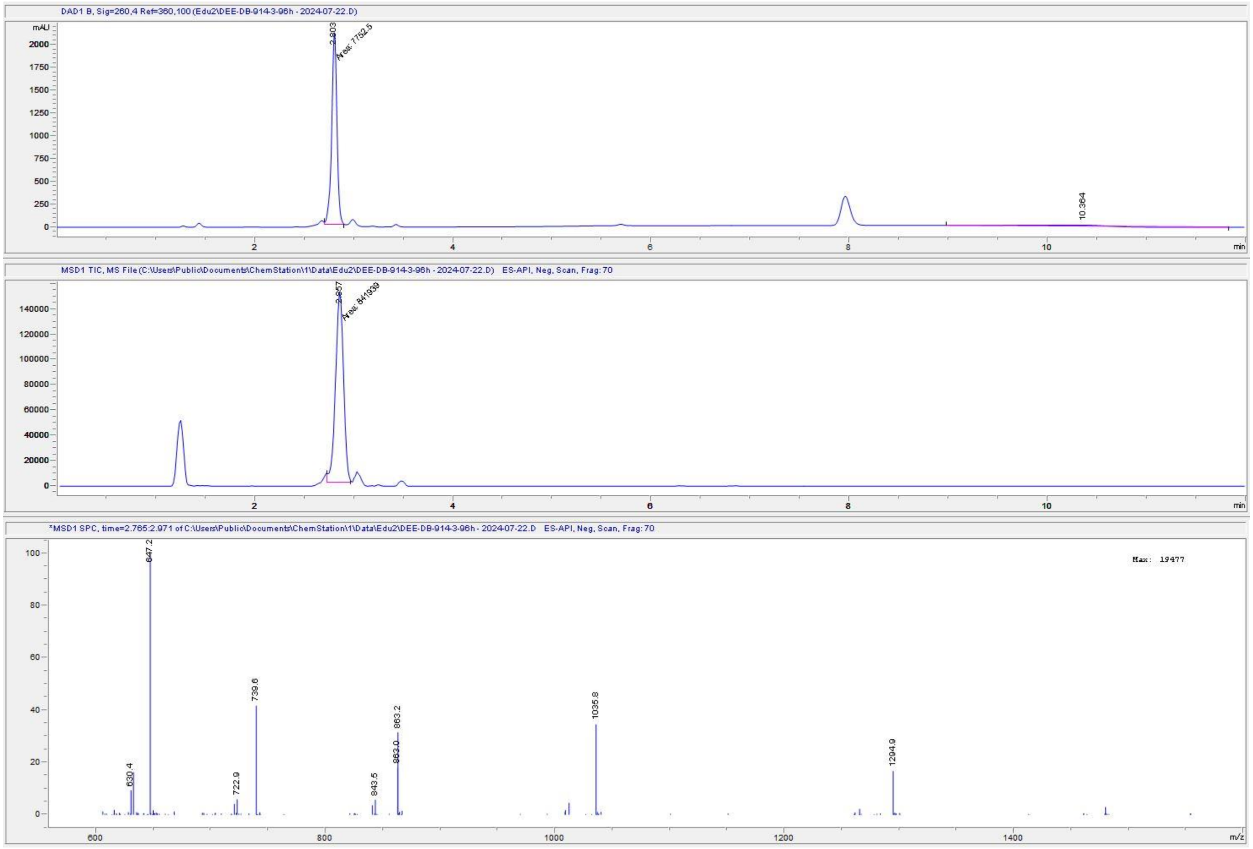

Figure S126. Analytical HPLC trace of C−H functionalization of **S4** at pH 3.0 and 2 equiv. of Selenoxide **3** after 96 h with HPLC Method A. (Up) DAD chromatogram at 260 nm. (Middle) TIC chromatogram. (Below) Ionization of peak at 2.859 min containing **S4**. The appearance of a new set of minor peaks in the ionization chromatogram corresponds with **S4**-Uracil and **S4**-Adenine.

C−H functionalization of DNA-conjugated arenes at pH 3.0 and 10 equiv. of Selenoxide 3

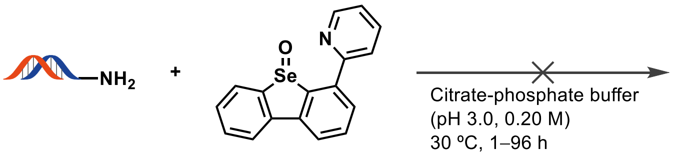

S4 **3**
10 equiv.

At 20–25 °C, 1.0 µL of **S4** (2.0 mM, 2.0 nmol, 1.0 equiv.) in water was added to a 1.5 mL Eppendorf tube. Next, 4.0 µL of Citrate-phosphate buffer (pH 3.0, c = 500 mM) was added. Then, 5.0 µL of a selenoxide **3** stock solution (4.0 mM, 20 nmol, 10 equiv.) in water was added. The mixture was vortexed for 5 seconds, transferred into a Thermocycler pre-heated at 30 °C, and incubated at 30 °C for 96 hours at 600 rpm. After the indicated time for each sample, an aliquot of 0.5 µL of the reaction mixture was diluted to 40 µL with water for LC–MS analysis.

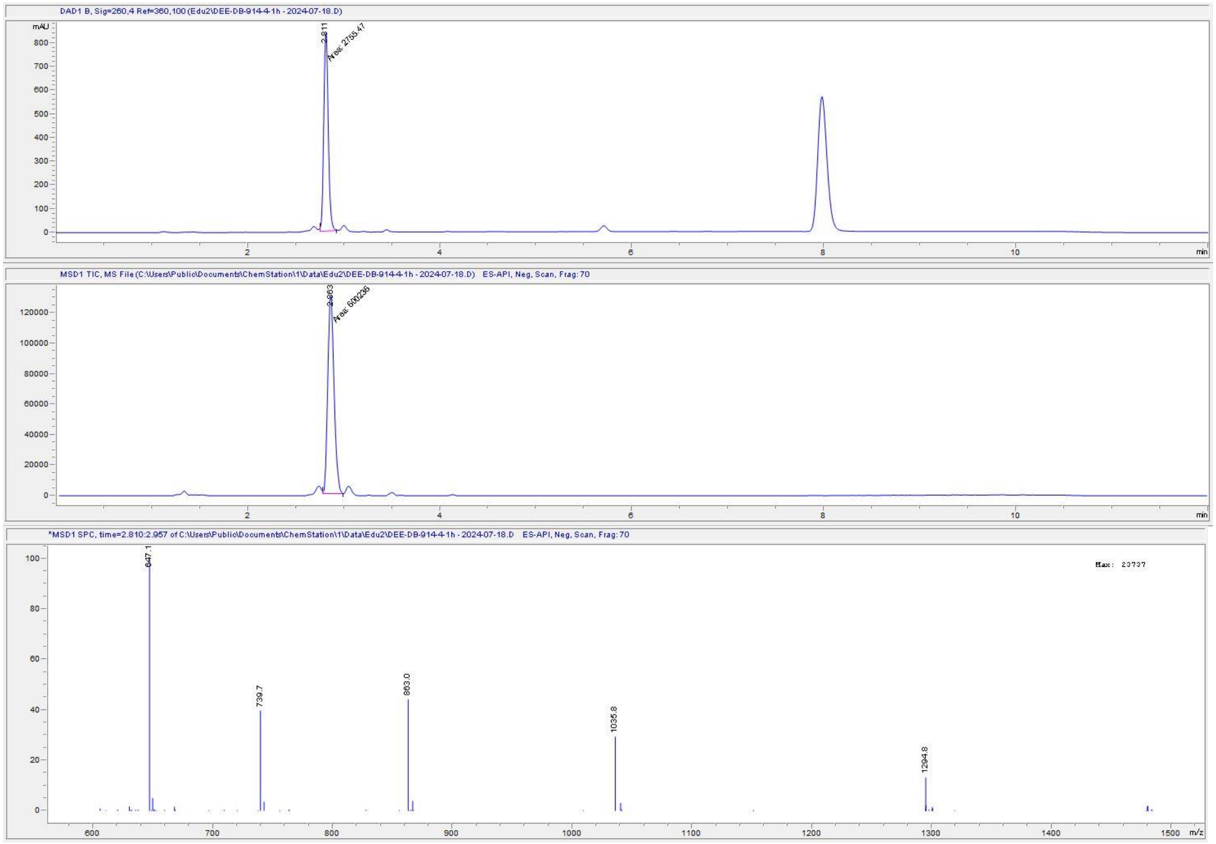

Figure S127. Analytical HPLC trace of C−H functionalization of **S4** at pH 3.0 and 10 equiv. of Selenoxide **3** after 1 h with HPLC Method A. (Up) DAD chromatogram at 260 nm. (Middle) TIC chromatogram. (Below) Ionization of peak at 2.863 min containing **S4**. No observable DNA damage.

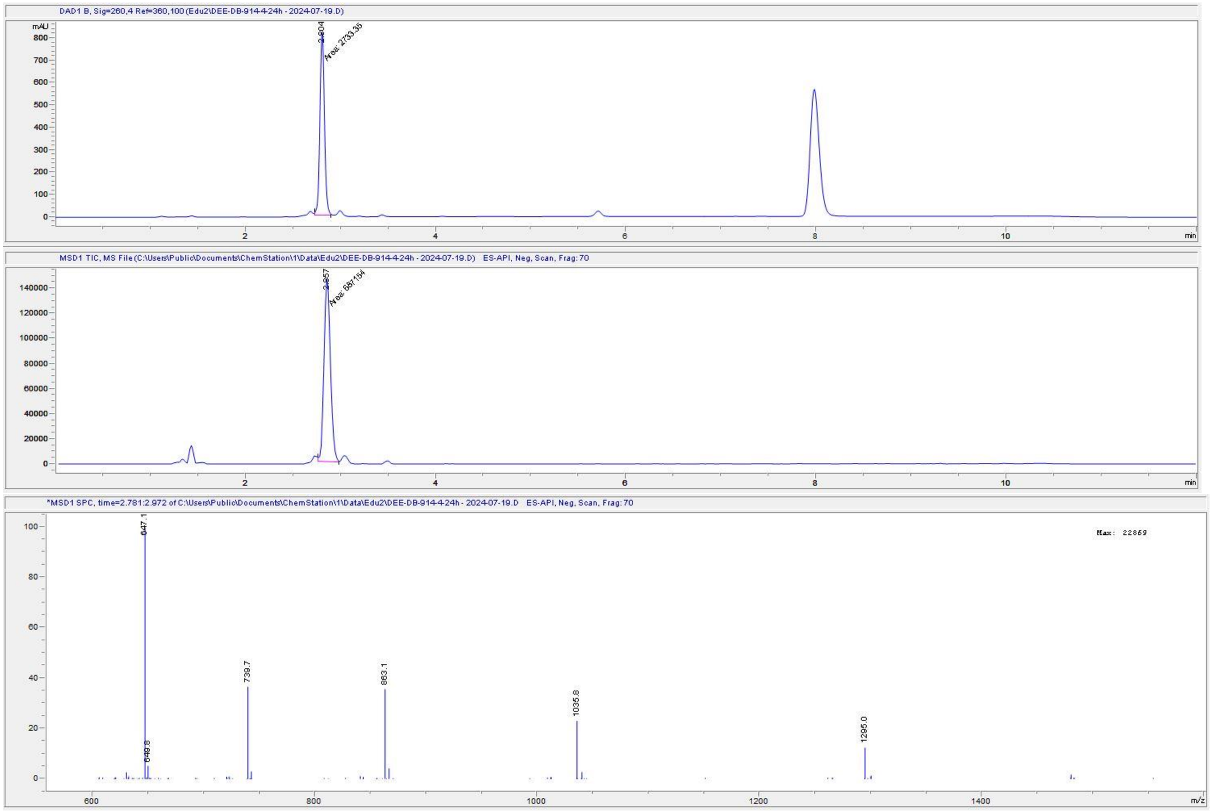

Figure S128. Analytical HPLC trace of C−H functionalization of **S4** at pH 3.0 and 10 equiv. of Selenoxide **3** after 24 h with HPLC Method A. (Up) DAD chromatogram at 260 nm. (Middle) TIC chromatogram. (Below) Ionization of peak at 2.857 min containing **S4**. No observable DNA damage.

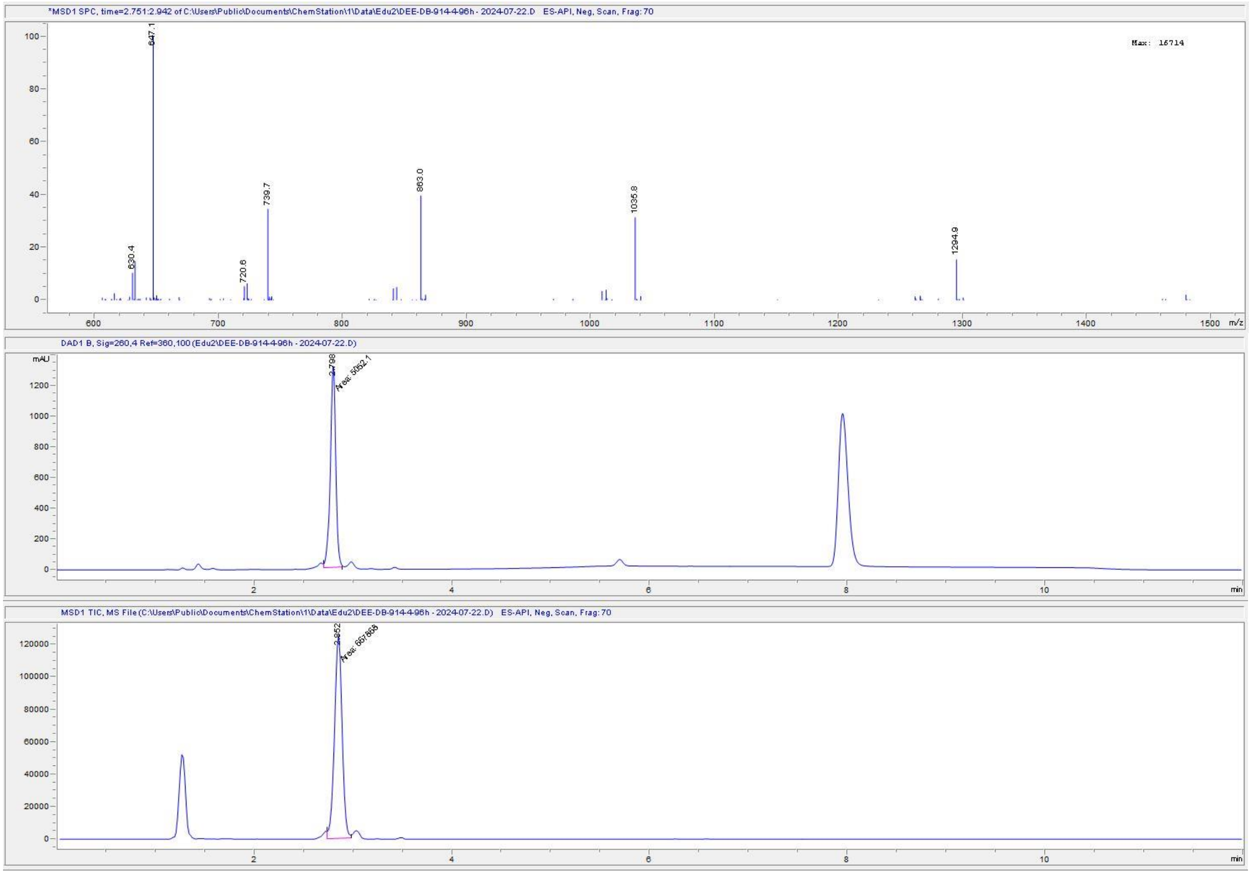

Figure S129. Analytical HPLC trace C−H functionalization of **S4** at pH 3.5 and 10 equiv. of Selenoxide **3** after 96 h with HPLC Method A. (Up) DAD chromatogram at 260 nm. (Middle) TIC chromatogram. (Below) Ionization of peak at 2.852 min containing **S4**. The appearance of a new set of minor peaks in the ionization chromatogram corresponds with **S4**-Uracil and **S4**-Adenine.

C−H functionalization of DNA-conjugated arenes at pH 3.0 and 50 equiv. of Selenoxide 3

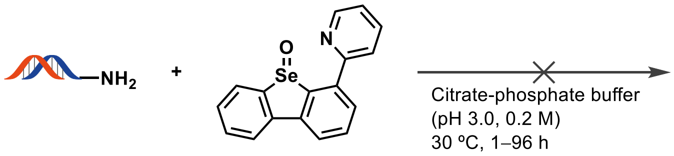

S4 **3** 50 equiv.

At 20–25 °C, 1.0 µL of **S4** (2.0 mM, 2.0 nmol, 1.0 equiv.) in water was added to a 1.5 mL Eppendorf tube. Next, 9.0 µL of Citrate-phosphate buffer (pH 3.0, c = 500 mM) was added. Then, 10 µL of a selenoxide **3** stock solution (10 mM, 100 nmol, 50 equiv.) in water was added. The mixture was vortexed for 5 seconds, transferred into a Thermocycler pre-heated at 30 °C, and incubated at 30 °C for 96 hours at 600 rpm. After the indicated time for each sample, an aliquot of 0.5 µL of the reaction mixture was diluted to 40 µL with water for LC–MS analysis.

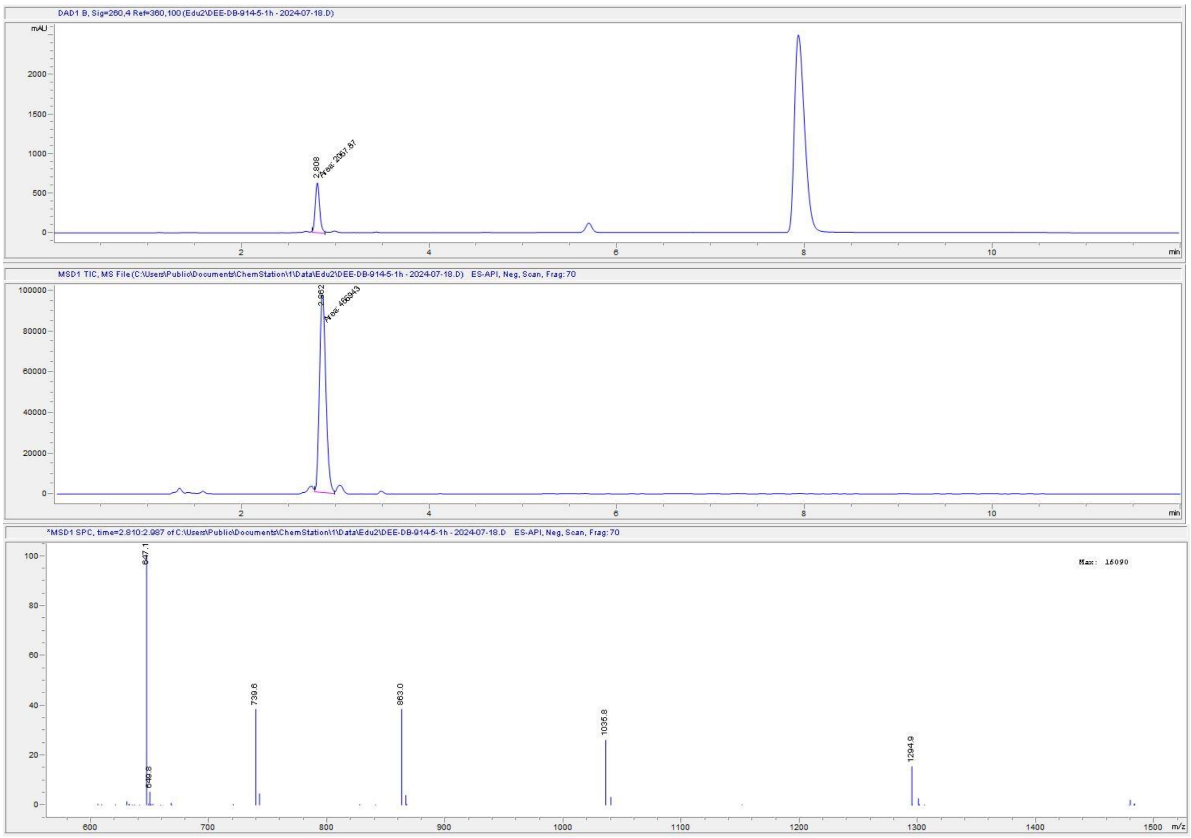

Figure S130. Analytical HPLC trace of C−H functionalization of **S4** at pH 3.0 and 50 equiv. of Selenoxide **3** after 1 h with HPLC Method A. (Up) DAD chromatogram at 260 nm. (Middle) TIC chromatogram. (Below) Ionization of peak at 2.862 min containing **S4**. No observable DNA damage.

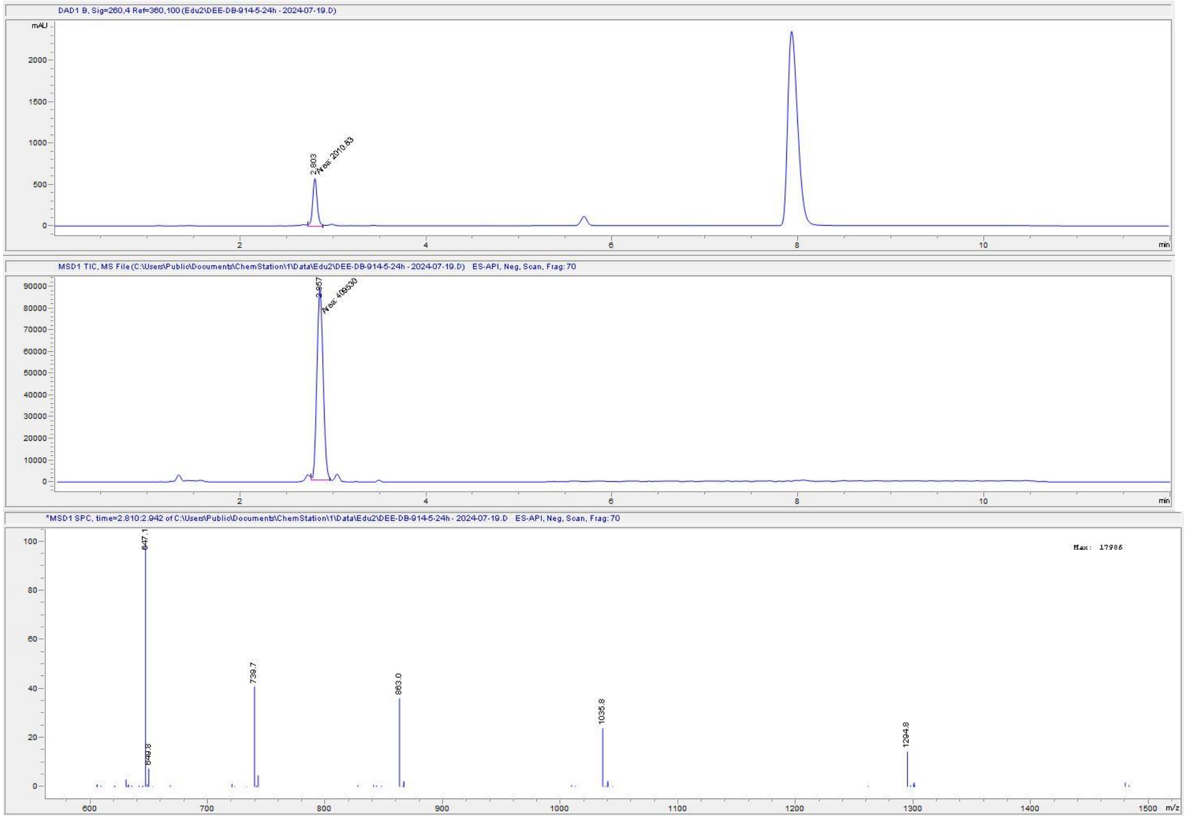

Figure S131. Analytical HPLC trace of C−H functionalization of **S4** at pH 3.0 and 50 equiv. of Selenoxide **3** after 24 h with HPLC Method A. (Up) DAD chromatogram at 260 nm. (Middle) TIC chromatogram. (Below) Ionization of peak at 2.857 min containing **S4**. No observable DNA damage.

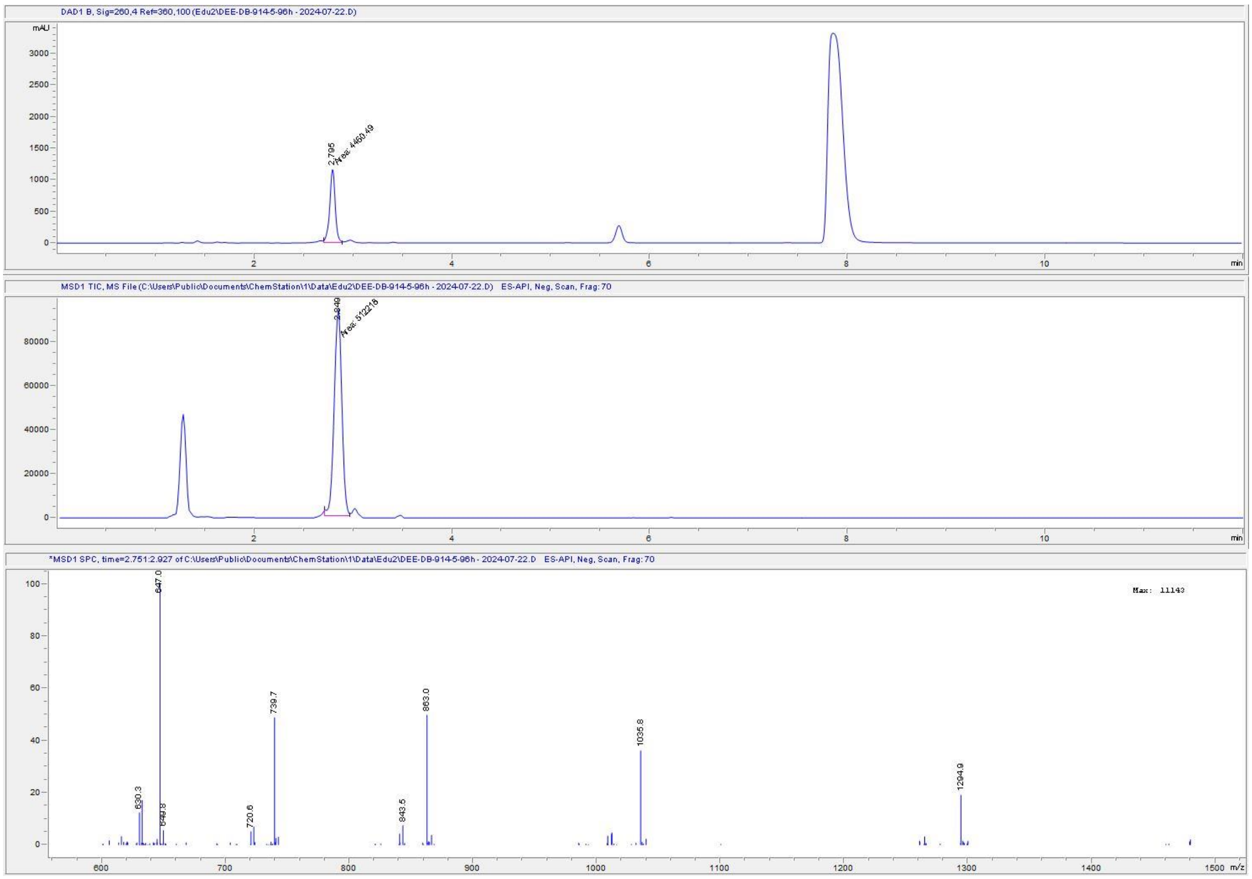

Figure S132. Analytical HPLC trace C−H functionalization of **S4** at pH 3.5 and 50 equiv. of Selenoxide **3** after 96 h with HPLC Method A. (Up) DAD chromatogram at 260 nm. (Middle) TIC chromatogram. (Below) Ionization of peak at 2.849 min containing **S4**. The appearance of a new set of minor peaks in the ionization chromatogram corresponds with **S4**-Uracil and **S4**-Adenine.

C−H functionalization of DNA-conjugated arenes at pH 2

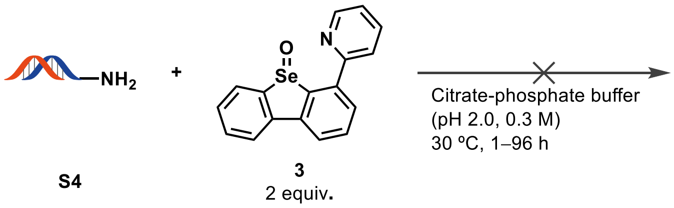

At 20–25 °C, 1.0 µL of **S4** (2.0 mM, 2.0 nmol, 1.0 equiv.) in water was added to a 1.5 mL Eppendorf tube. Next, 1.0 µL of Citrate-phosphate buffer (pH 2.0, c = 1.00 M) was added. Then, 1.0 µL of a selenoxide **3** stock solution (4.0 mM, 4.0 nmol, 2.0 equiv.) in water was added. The mixture was vortexed for 5 seconds, transferred into a Thermocycler pre-heated at 30 °C, and incubated at 30 °C for 96 hours at 600 rpm. After the indicated time for each sample, an aliquot of 0.5 µL of the reaction mixture was diluted to 40 µL with water for LC–MS analysis.

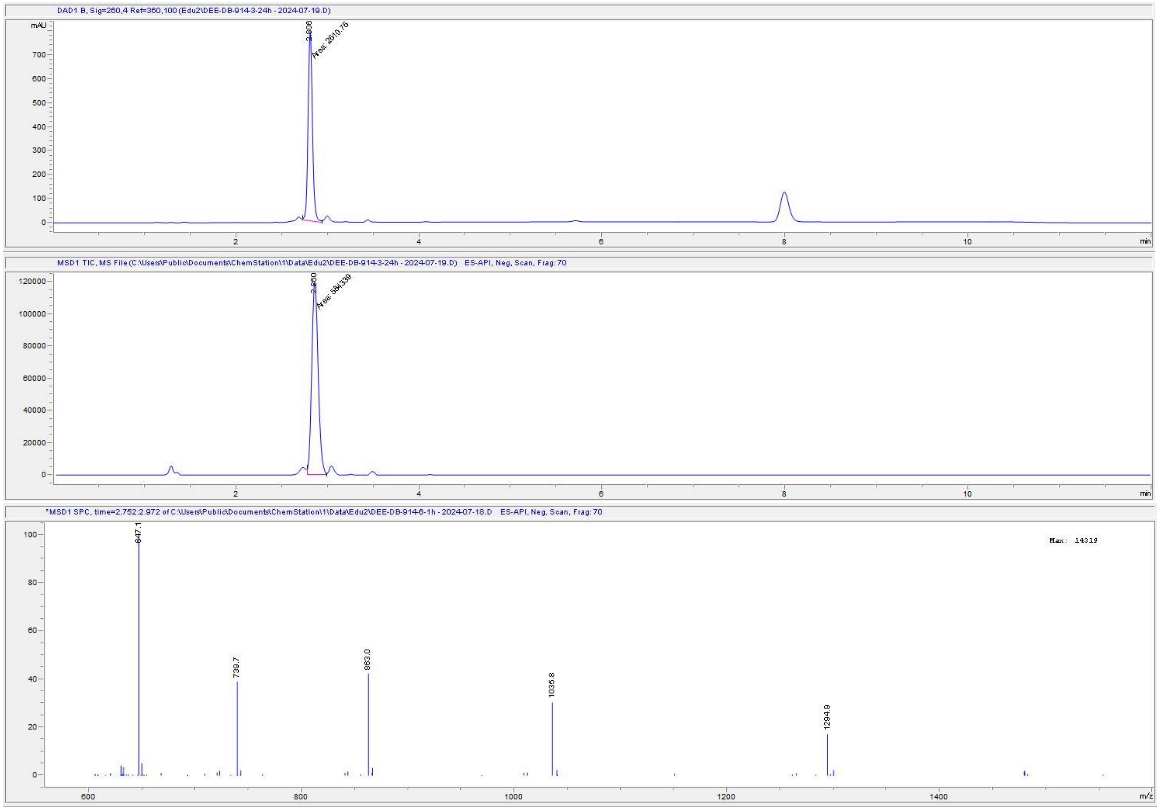

Figure S133. Analytical HPLC trace of C−H functionalization of **S4** at pH 2.0 and 2 equiv. of Selenoxide **3** after 1 h with HPLC Method A. (Up) DAD chromatogram at 260 nm. (Middle) TIC chromatogram. (Below) Ionization of peak at 2.865 min containing **S4**. No observable DNA damage.

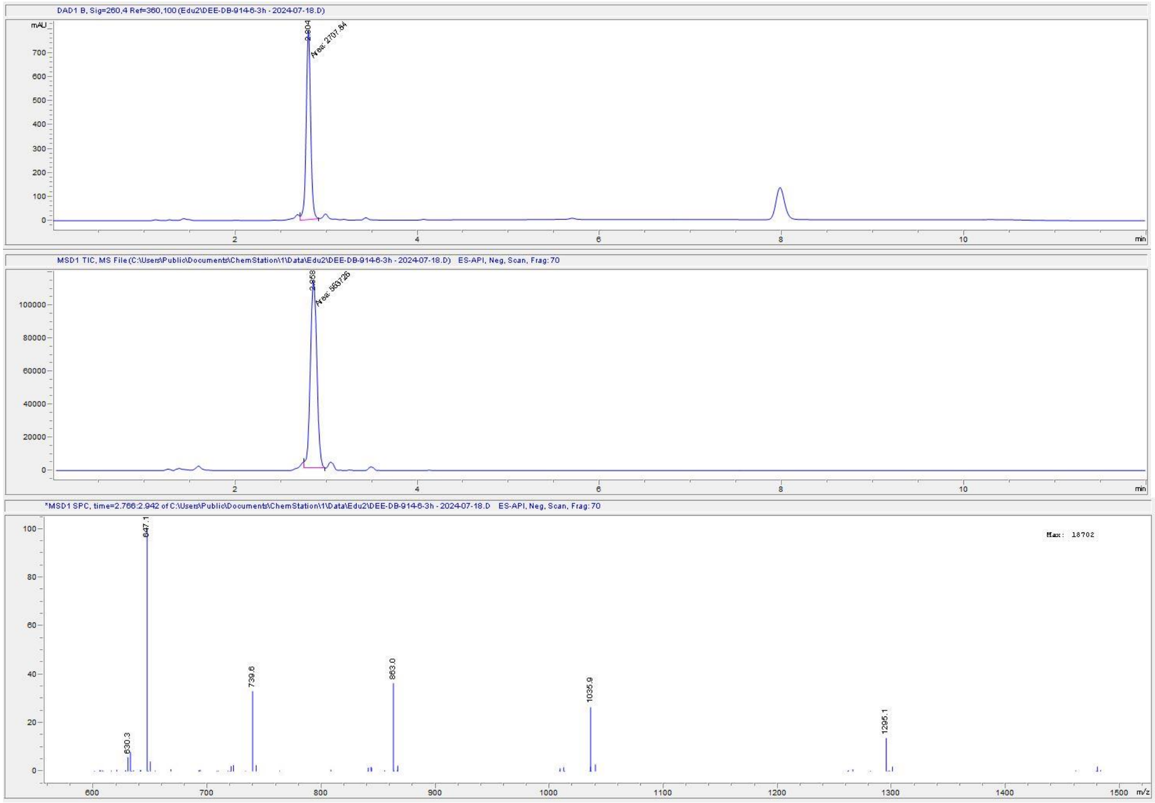

Figure S134. Analytical HPLC trace of C−H functionalization of **S4** at pH 2.0 and 2 equiv. of Selenoxide **3** after 3 h with HPLC Method A. (Up) DAD chromatogram at 260 nm. (Middle) TIC chromatogram. (Below) Ionization of peak at 2.858 min containing **S4**. The appearance of a new set of minor peaks in the ionization chromatogram corresponds with **S4**-Uracil and **S4**-Adenine.

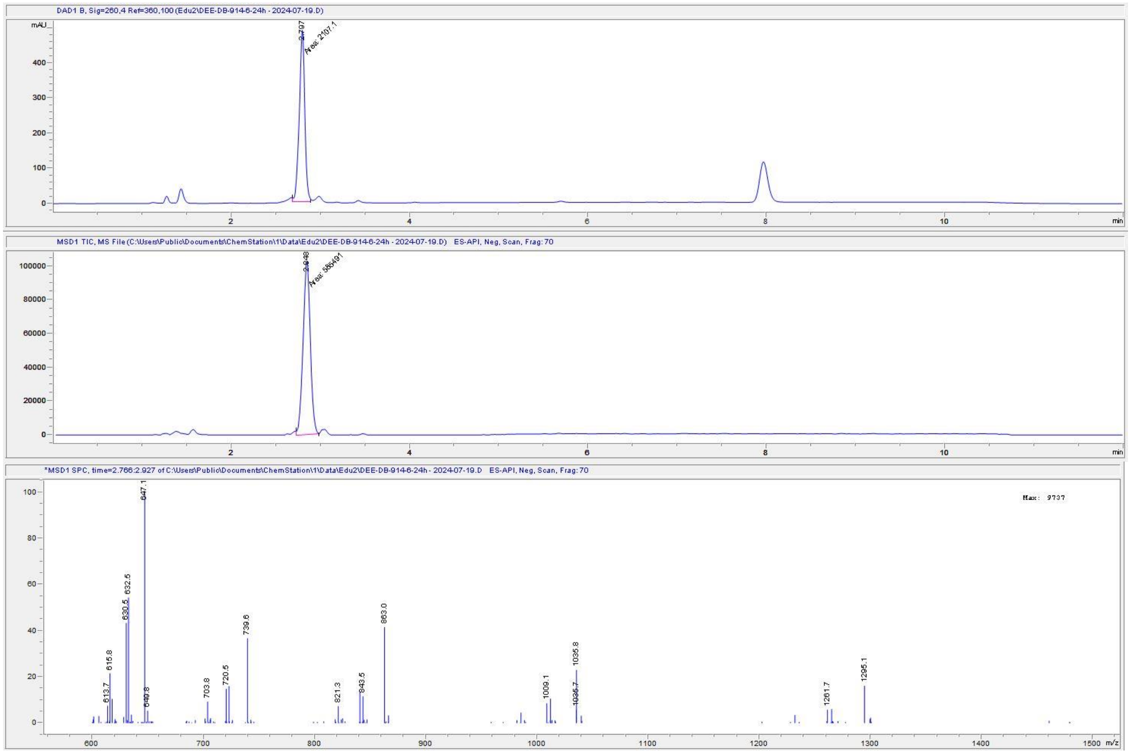

Figure S135. Analytical HPLC trace of C−H functionalization of **S4** at pH 2.0 and 2 equiv. of Selenoxide **3** after 24 h with HPLC Method A. (Up) DAD chromatogram at 260 nm. (Middle) TIC chromatogram. (Below) Ionization of peak at 2.848 min containing **S4**. The appearance of a new set of peaks in the ionization chromatogram corresponds with **S4**-Uracil and **S4**-Adenine.

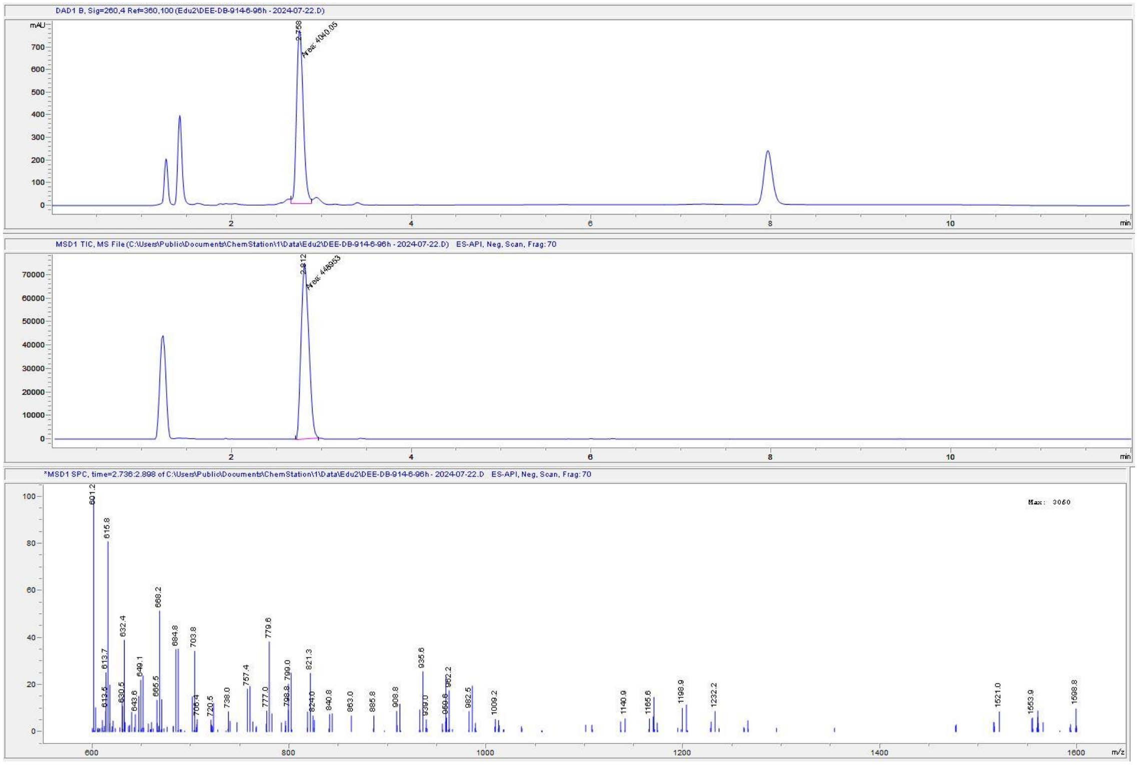

Figure S136. Analytical HPLC trace of C−H functionalization of **S4** at pH 2.0 and 2 equiv. of Selenoxide **3** after 96 h with HPLC Method A. (Up) DAD chromatogram at 260 nm. (Middle) TIC chromatogram. (Below) Ionization of peak at 2.848 min containing **S4**. The appearance of a new range of peaks in the ionization chromatogram supports major decomposition of the DNA sample. The set of peaks corresponding to **S4** is no longer observable.

C−H functionalization of DNA-conjugated arenes at pH 2 and 10 equiv. of Selenoxide 3

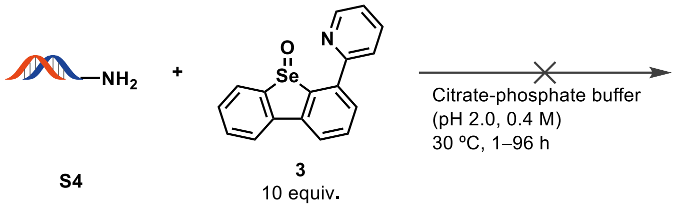

At 20–25 °C, 1.0 µL of **S4** (2.0 mM, 2.0 nmol, 1.0 equiv.) in water was added to a 1.5 mL Eppendorf tube. Next, 4.0 µL of Citrate-phosphate buffer (pH 2.0, c = 1.00 M) was added. Then, 5.0 µL of a selenoxide **3** stock solution (4.0 mM, 20 nmol, 100 equiv.) in water was added. The mixture was vortexed for 5 seconds, transferred into a Thermocycler pre-heated at 30 °C, and incubated at 30 °C for 96 hours at 600 rpm. After the indicated time for each sample, an aliquot of 0.5 µL of the reaction mixture was diluted to 40 µL with water for LC–MS analysis.

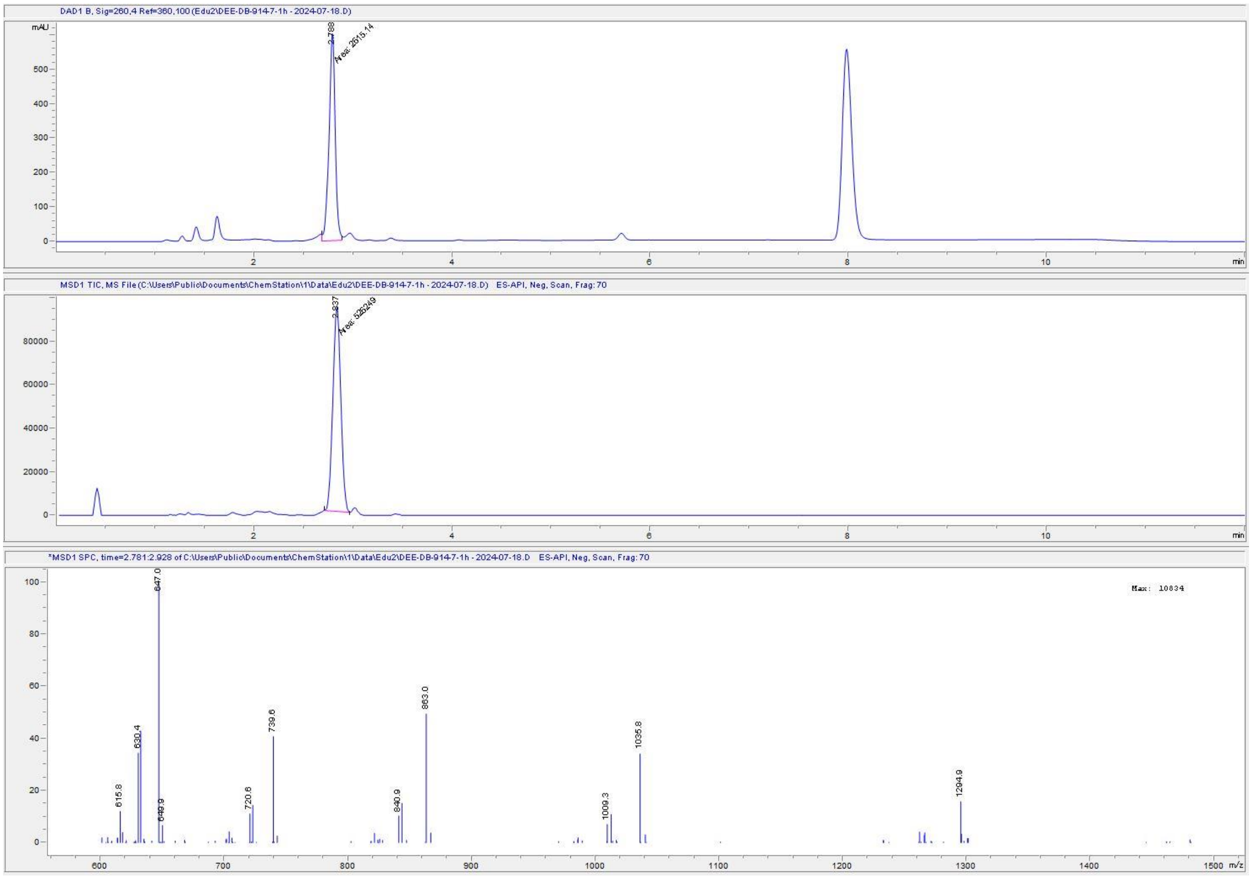

Figure S137. Analytical HPLC trace of C−H functionalization of **S4** at pH 2.0 and 10 equiv. of Selenoxide **3** after 1 h with HPLC Method A. (Up) DAD chromatogram at 260 nm. (Middle) TIC chromatogram. (Below) Ionization of peak at 2.837 min containing **S4**. The appearance of a new set of minor peaks in the ionization chromatogram corresponds with **S4**-Uracil and **S4**-Adenine.

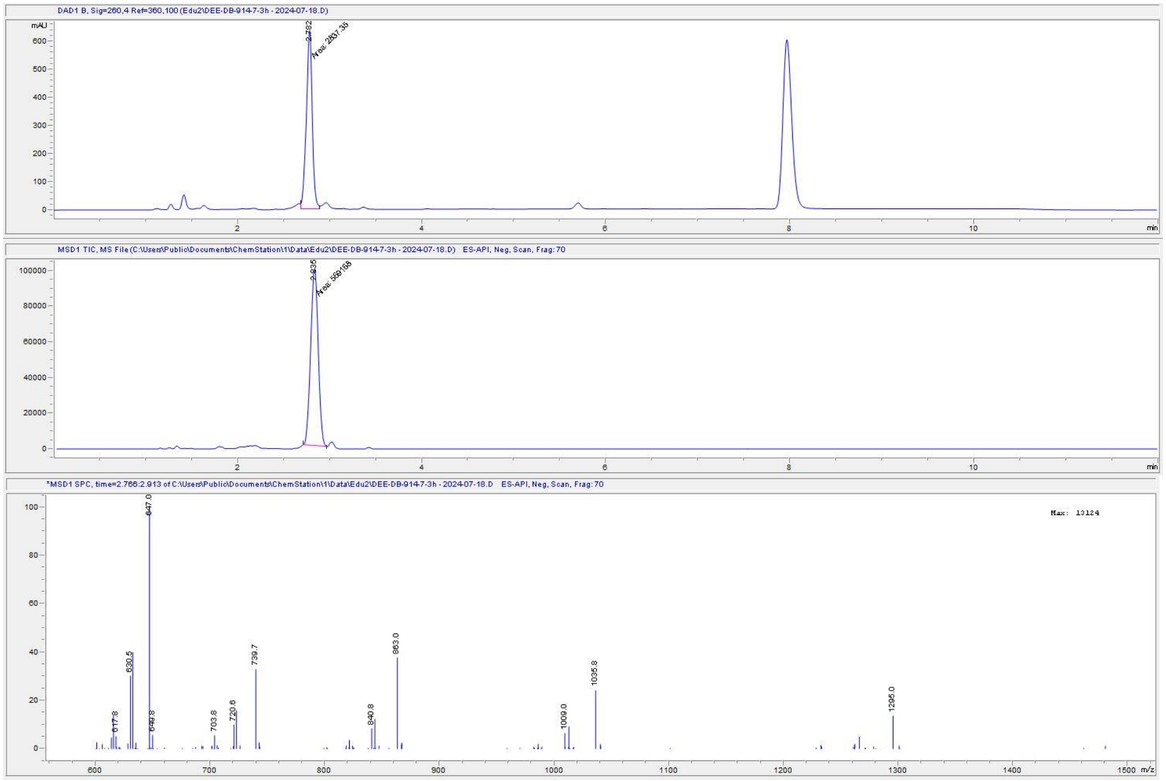

Figure S138. Analytical HPLC trace of C−H functionalization of **S4** at pH 2.0 and 10 equiv. of Selenoxide **3** after 3 h with HPLC Method A. (Up) DAD chromatogram at 260 nm. (Middle) TIC chromatogram. (Below) Ionization of peak at 2.835 min containing **S4**. The appearance of a new set of minor peaks in the ionization chromatogram corresponds with **S4**-Uracil and **S4**-Adenine.

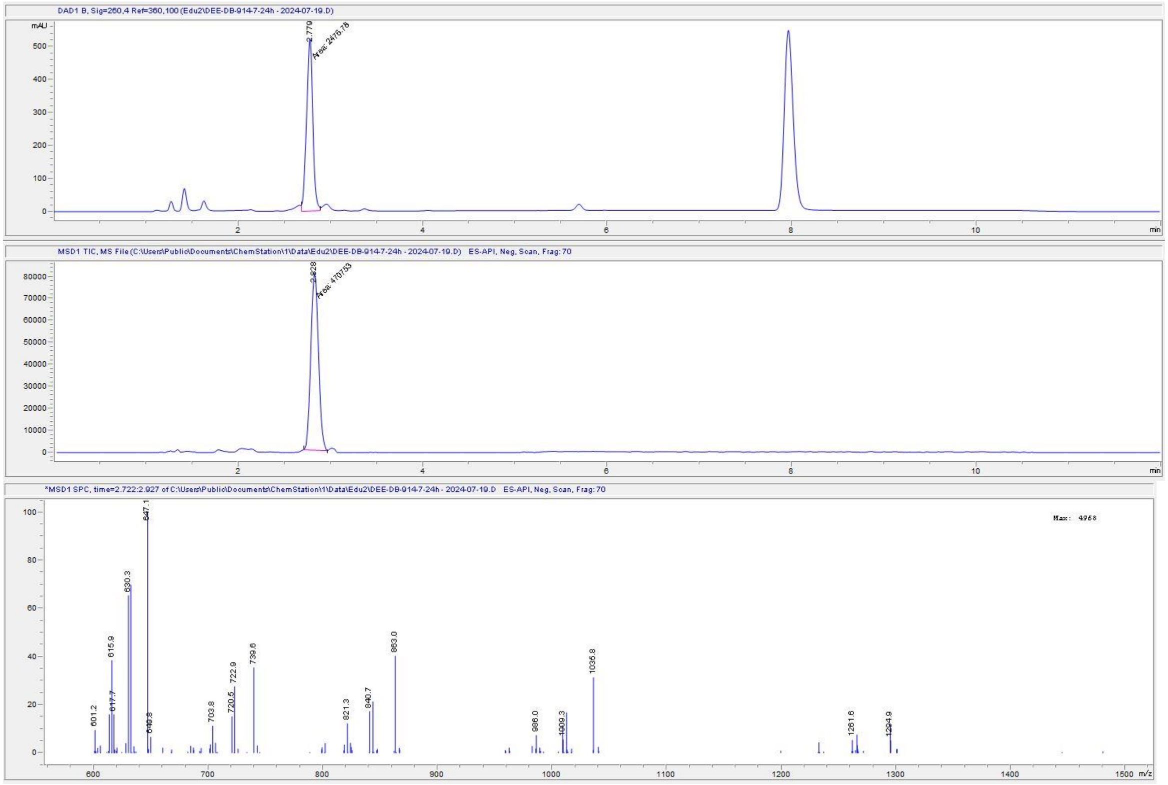

Figure S139. Analytical HPLC trace of C−H functionalization of **S4** at pH 2.0 and 10 equiv. of Selenoxide **3** after 24 h with HPLC Method A. (Up) DAD chromatogram at 260 nm. (Middle) TIC chromatogram. (Below) Ionization of peak at 2.828 min containing **S4**. The appearance of a new set of peaks in the

ionization chromatogram corresponds with **S4**-Uracil and **S4**-Adenine.

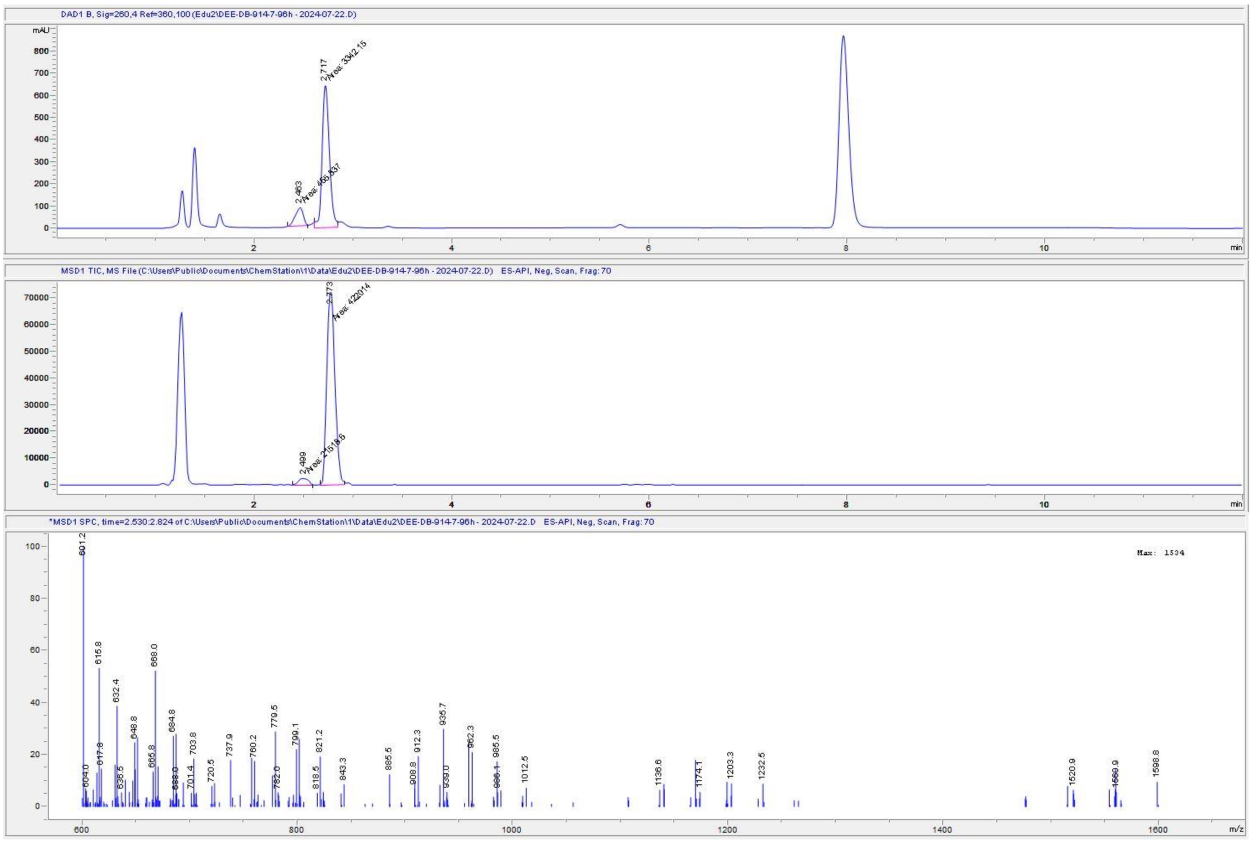

Figure S140. Analytical HPLC trace of C−H functionalization of **S4** at pH 2.0 and 2 equiv. of Selenoxide **3** after 96 h with HPLC Method A. (Up) DAD chromatogram at 260 nm. (Middle) TIC chromatogram. (Below) Ionization of peak at 2.773 min containing **S4**. The appearance of a new range of peaks in the ionization chromatogram supports major decomposition of the DNA sample. The set of peaks corresponding to **S4** is no longer observable.

DNA ligation of DNA-conjugated selenonium salt 24

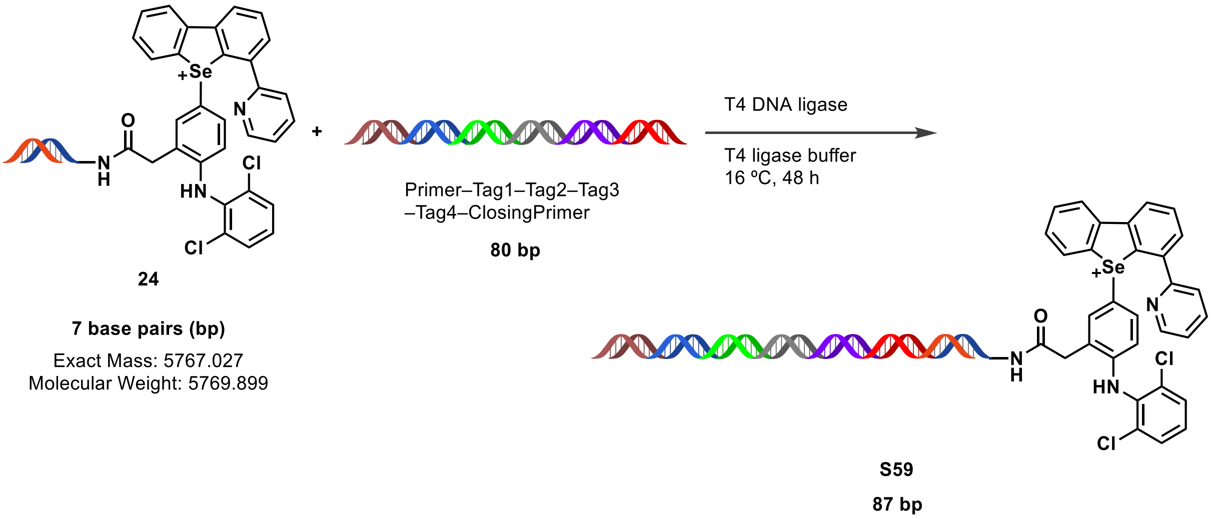

At 4 °C, 5.0 µL of **24** (1.0 mM, 5 nmol, 1.0 equiv.) in water was added to a 1.5 mL Eppendorf tube. Next, 13 µL of a stock solution of the DNA fragment Primer–Tag1–Tag2–Tag3–Tag4–ClosingPrimer (0.47 mM, 6.1 nmol, 1.2 equiv.) in water was added and cooled to 16 °C. In another Eppendorf tube at 4 °C, 2.0 µL of 10X T4 DNA ligase buffer and 0.20 µL of T4 DNA ligase were premixed. The mixture was added over

the solution of **24**, vortexed for 5 seconds, transferred into a Thermocycler pre-cooled at 16 °C, and incubated at 16 °C for 48 hours without stirring. After 48 hours, the reaction mixture was centrifuged and diluted to 40 µL with water, 4 µL of a 5 M NaCl solution in water and 132 µL of cold ethanol were added to precipitate the DNA conjugate. The ligation was analyzed by GEL electrophoresis with ethidium bromide as dye, 3% agarose gel and 75V for 1 hour to 3 hours.

GEL electrophoresis protocol

- 3% agarose gel (0.9 g) to 30 mL of buffer. 5 uL of ethidium bromide (10 ng/µL) as dye added to the gel.

8 lanes:

- Lane 1: Selenonium salt **24** (100 ng).
- Lane 2: DNA ladder (Bio-Rad Laboratories EZ Load 20 bp Molecular Ruler)
- Lane 3: Ligation mixture of **S59** (100 ng).
- Lane 4: Primer–Tag1–Tag2–Tag3–Tag4–ClosingPrimer (100 ng).
- Lane 5: Selenonium salt **24** (50 ng).
- Lane 6: Ligation mixture of **S59** (50 ng).
- Lane 7: Primer–Tag1–Tag2–Tag3–Tag4–ClosingPrimer (50 ng).
- Lane 8: DNA ladder (Bio-Rad Laboratories EZ Load 20 bp Molecular Ruler)

After loading the samples, the gel was run at 75V for 1 hour to 3 hours.

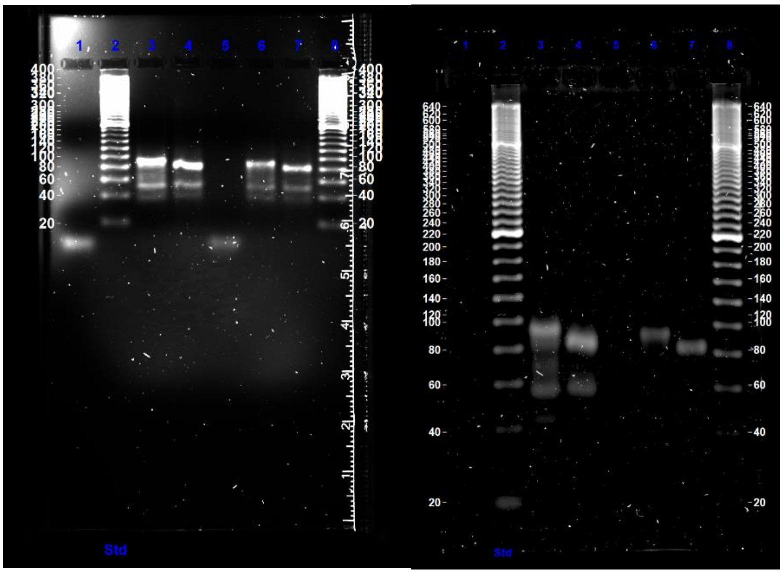

Figure S141. Images of the DNA gel after ethidium bromide staining. (Left) 1 hour. (Right) 3 hours. After 1 hour, selenonium salt **24** is still visible in lanes 1 and 5, but no **24** is detected in the lanes corresponding to the ligation mixture (lanes 3 and 6). DNA conjugates in lanes 3, 4, 6, and 7 fall within the range of 80–100 bp. The DNA conjugates in lanes 3 and 6 migrated less distance in the gel, correlating with the presence of a ligation product with a higher base pair count (**S59**). After 3 hours, **24** is not detected in the gel anymore. White lines and numbers on the side of the gel indicate bps of the reference bands of the ladder (lanes 2 and 8)

DNA ligation of DNA-conjugate S25

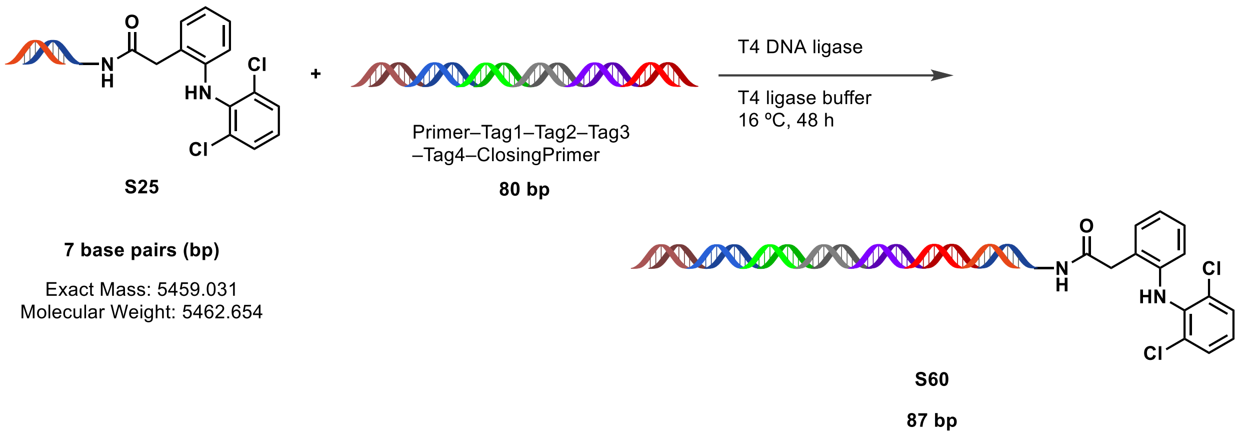

At 4 °C, 6.0 µL of **S25** (1.0 mM, 6 nmol, 1.2 equiv.) in water was added to a 1.5 mL Eppendorf tube. Next, 10.6 µL of a stock solution of the DNA fragment Primer–Tag1–Tag2–Tag3–Tag4–ClosingPrimer (0.47 mM, 5.0 nmol, 1.0 equiv.) in water was added and cooled down to 16 °C. In another Eppendorf tube at 4 °C, 2.0 µL of 10X T4 DNA ligase buffer and 0.20 µL of T4 DNA ligase were premixed. The mixture was added over the solution of **S25**, vortexed for 5 seconds, transferred into a Thermocycler pre-cooled at 16 °C, and incubated at 16 °C for 48 hours without stirring. After 24 hours, reaction mixture was centrifuged and diluted to 40 µL with water, 4 µL of a 5 M NaCl solution in water and 132 µL of cold ethanol were added to precipitate the DNA conjugate.

DNA desalting and rebuffering was performed by charging the solution in an AMICON® filter unit from Sigma Aldrich (50 kD) in 300 µL of water, centrifuged at 4 °C and 10000 x g for at least 30 minutes, until the volume decreased to < 10 µL. Another 300 µL of water were added and the process was repeated all over again for at least 3 times. The remaining solution concentration was determined by A_{260} absorption using a Thermo Scientific™ NanoDrop™ One^{C}, concentration of the solution was adjusted to 2.0 mM and stored in the freezer at −20 °C.

The ligation was analyzed by GEL electrophoresis with SYBR® Green as dye, 3% agarose gel and 75V for 1 hour to 3 hours.

GEL electrophoresis protocol

- 3% agarose gel (0.9 g) to 30 mL of buffer.
  4 lanes:
  - Lane 1: DNA ladder (Bio-Rad Laboratories EZ Load 20 bp Molecular Ruler)
  - Lane 2: DNA conjugate **S25** (10 ng).
  - Lane 3: Primer–Tag1–Tag2–Tag3–Tag4–ClosingPrimer (5 ng).
  - Lane 4: Ligation mixture of **S60** (5 ng).
  - Lane 5: DNA conjugate **S25** (100 ng).
  - Lane 6: Primer–Tag1–Tag2–Tag3–Tag4–ClosingPrimer (30 ng).
  - Lane 7: Ligation mixture of **S60** (30 ng).
  - Lane 8: DNA ladder (Bio-Rad Laboratories EZ Load 20 bp Molecular Ruler)

After loading the samples, the gel was run at 75V for 1 hour to 3 hours.

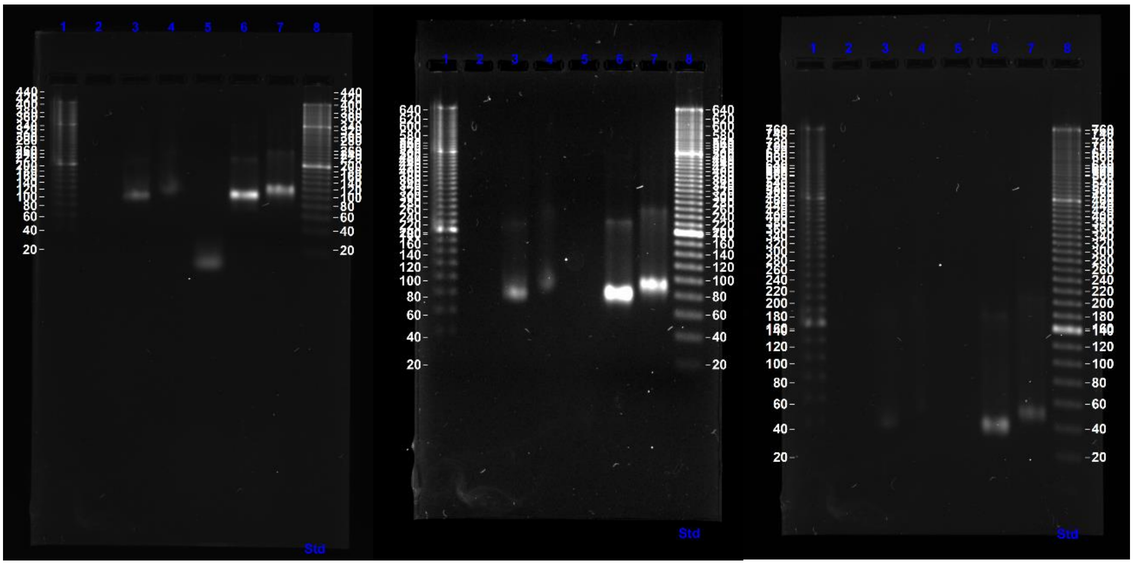

Figure S142. Images of the DNA gel after the corresponding time at 75V. (Left) 1 hour. (Middle) 2 hours (Right) 3 hours. After 1 hour, DNA-conjugate **S25** is still visible in lane 5, but no **S25** is detected in lanes 4 or 7 corresponding to the ligation mixture. DNA conjugates in lanes 3,4,6 and 7 fall within the range of 80–100 bp. The DNA conjugate in lane 7 migrated less distance in the gel, correlating with the presence of a ligation product with a higher base pair count (**S60**). After 2 and 3 hours, **S25** is not detected anymore. White lines and numbers on the side of the gel indicate bps of the reference bands of the ladder (lane 8).

C–H functionalization of DNA-conjugate S60

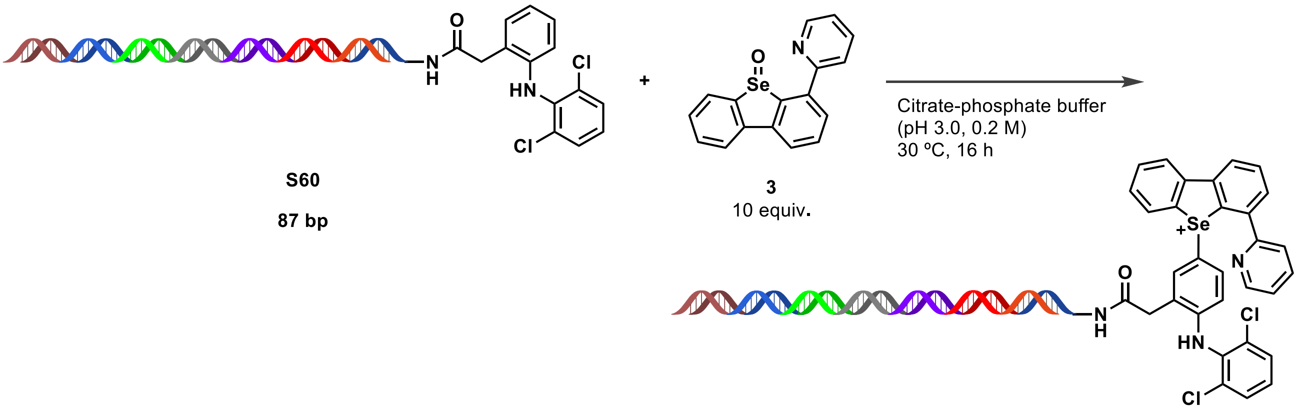

At 20–25 °C, 0.5 µL of **S60** (2.0 mM, 1.0 nmol, 1.0 equiv.) in water was added to a 1.5 mL Eppendorf tube. Next, 2.0 µL of Citrate-phosphate buffer (pH 3.0, c = 500 mM) was added. Then, 2.5 µL of a selenoxide **3** stock solution (4.0 mM, 10 nmol, 10 equiv.) in water was added. The mixture was vortexed for 5 seconds, transferred into a Thermocycler pre-heated at 30 °C, and incubated at 30 °C for 16 hours at 600 rpm. After 16 hours, reaction was quenched by addition of 10 µL of borate buffer (pH 9.4, c = 500 mM). Reaction mixture was centrifuged and diluted to 45 µL with water, 6 µL of a 5 M NaCl solution in water and 180 µL of cold ethanol were added to precipitate the DNA conjugate.

DNA desalting and rebuffering was performed by charging the solution in an AMICON® filter unit from Sigma Aldrich (50 kD) in 300 µL of water, centrifuged at 4 °C and10000 x g for at least 30 minutes, until the volume decreased to < 10 µL. Another 300 µL of water were added and the process was repeated all over again for at least 3 times. The remaining solution concentration was determined by A_{260} absorption

using a Thermo Scientific™ NanoDrop™ One^{C}, concentration of the solution was adjusted to 100 ng/µL and stored in the freezer at −20 °C.

The reaction was replicated five times to ensure consistency and accuracy in the qPCR measurements.

The ligation was analyzed by GEL electrophoresis with SYBR® Green as dye, 3% agarose gel and 75V for 1 hour to 3 hours.

GEL electrophoresis protocol

- 3.5% agarose gel (1.05 g) to 30 mL of buffer.
  8 lanes:
  - Lane 1: DNA ladder (Bio-Rad Laboratories EZ Load 20 bp Molecular Ruler).
  - Lane 2: DNA conjugate **S25** (120 ng).
  - Lane 3: Primer–Tag1–Tag2–Tag3–Tag4–ClosingPrimer (60 ng).
  - Lane 4: **S59-1**, C─H functionalization mixture of **S60** (60 ng). 1st repetition.
  - Lane 5: **S59-2**, C─H functionalization mixture of **S60** (60 ng). 2nd repetition.
  - Lane 6: **S59-3**, C─H functionalization mixture of **S60** (60 ng). 3rd repetition.
  - Lane 7: Primer–Tag1–Tag2–Tag3–Tag4–ClosingPrimer (30 ng).
  - Lane 8: DNA ladder (Bio-Rad Laboratories EZ Load 20 bp Molecular Ruler).

After loading the samples, the gel was run at 75V for 1 hour to 3 hours.

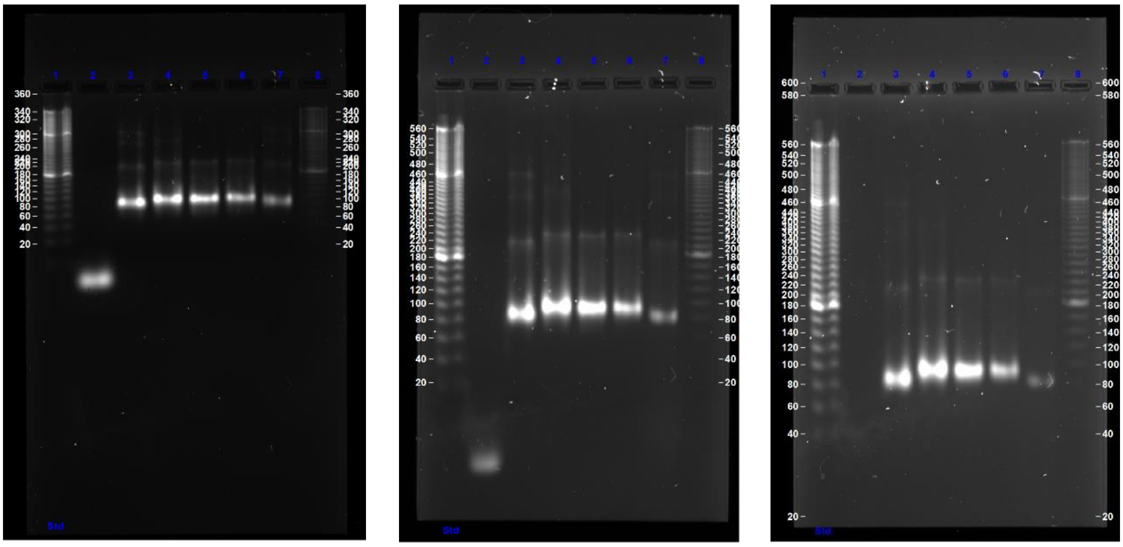

Figure S143. Images of the DNA gel after the corresponding time at 75V. (Left) 1 hour. (Middle) 2 hours (Right) 3 hours. After 1 hour, DNA conjugate **S25** is still visible in lane 2, but no **S25** is detected in any other lanes corresponding to the C─H functionalizations. DNA conjugates in lanes 3─7 fall within the range of 80─100 bp. The DNA conjugate in lanes 4, 5 and 6 migrated less distance in the gel, correlating with the presence of a ligation product with a higher base pair count. After 3 hours, **S25** is not detected anymore. White lines and numbers on the side of the gel indicate bps of the reference bands of the ladder (lane 1 and 8).

qPCR procedure

qPCR was performed with the KAPA SYBR® FASTqPCR Master Mix on a qPCR cycler MyiQTM Optics Module-Thermocycler (Bio-Rad Laboratories). Preparation of PCR-Mastermix:

- 18 µL of PCR-Mastermix contain 10 µL 10 µl KAPA SYBR® FASTqPCR Master Mix, 0.5 µL 10 µM forward primer, 0.5 µl 10 µM reverse primer, and 7 µL ddH_2O

All samples were subjected to PCR cycles as follows:

- 391 pM DNA samples were diluted as follows:

**Sample**	**dilution**	**c (DNA) / pM**	**m (DNA) / pg**
01	1:4	98	11
02	1:16	24	2.7
03	1:64	6.1	0.68
04	1:256	1.5	0.17
05	1:1024	0.38	$4.3 \cdot 10^{-2}$
06	1:4096	0.095	$1.1 \cdot 10^{-2}$
07	1:16384	0.024	$0.27 \cdot 10^{-2}$
08	1:65536	0.006	$6.7 \cdot 10^{-4}$

- 2.0 µL of the samples were added to a well in a 96 well plate. Afterwards, 18 µL of the PCR-Mastermix were added to the DNA. The PCR was conducted utilizing the following settings:

2 min. – 95 °C

15 s. – 95 °C } 40x
1 min. – 60 °C }

1 min. – 95 °C

30 s. – 60–90 °C in 1 °C steps (melting curve)

The fluorescence raw data was processed with LinRegPCR 11.0. The C_t values were determined and plotted against $\log_{10}$(dilution). The measurements were conducted six times, and all values are reported as mean $\pm$ SD.

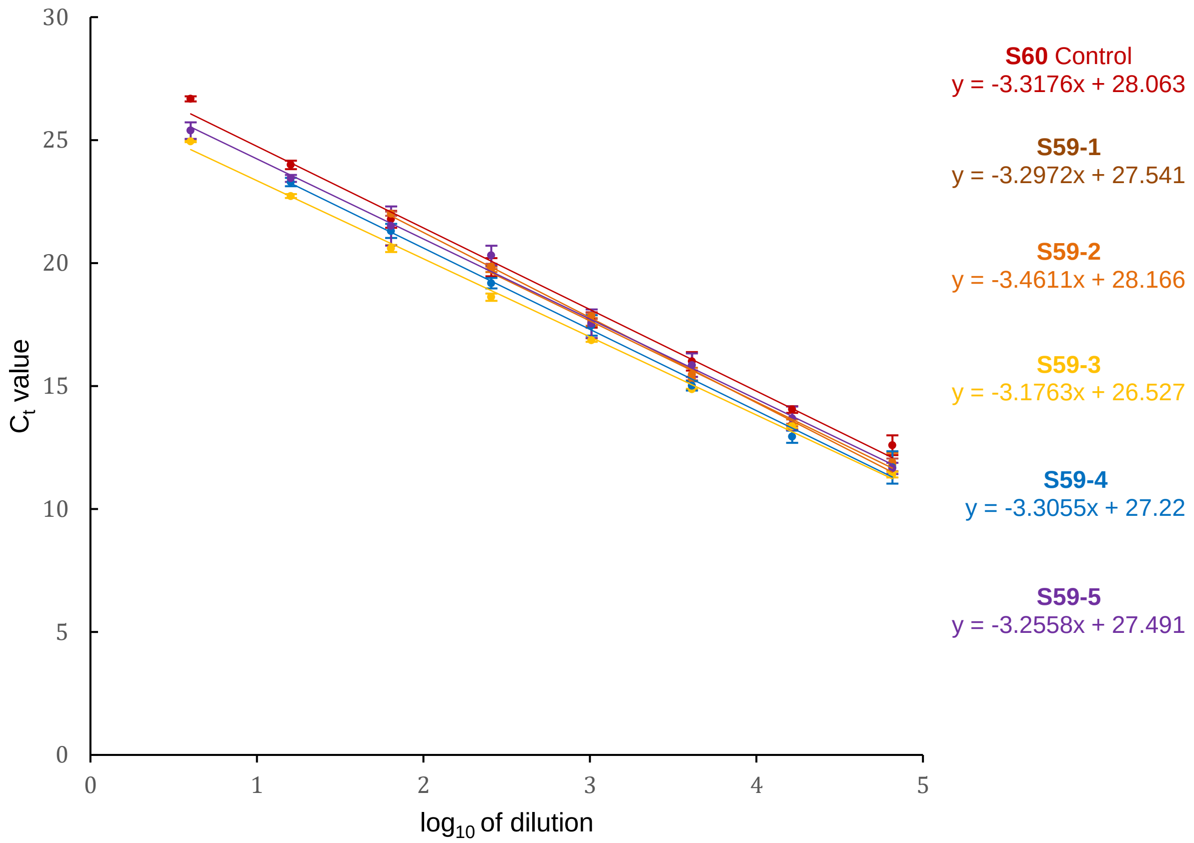

Figure S144. qPCR efficiency comparison between control **S60** and selenation reactions **S59-X** samples.

"Off-DNA" preparation of selenonium salts

Preparation of selenonium salt S61

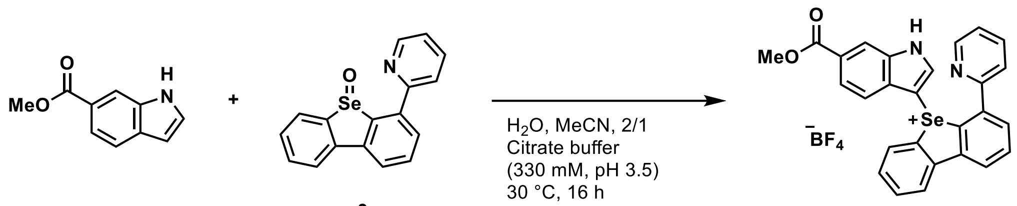

3
1.2 equiv.

S61, 78%

At 20–25 °C, a 4 mL glass vial equipped with a teflon-coated magnetic stirring bar was charged with methyl indole-6-carboxylate (3.5 mg, 20 µmol, 1.0 equiv.) and selenoxide **3** (7.8 mg, 24 µmol, 1.2 equiv.). Next, citrate buffer (2.0 mL, c = 330 mM, pH 3.5) and MeCN (1.0 mL, final volume percentage 33%) were added to the vial. The vial was placed in a pre-heated heating block at 30 °C and the reaction mixture was stired for 16 hours. The resulting mixture was diluted with 5 mL of DCM and poured into a separatory funnel. The DCM layer was washed with aqueous $NaBF_4$ solution (2 × ca. 10 ml, 5 % w/w), and with water (2 × ca. 10 ml). The DCM layer was dried over $MgSO_4$, filtered, and the solvent was removed under reduced pressure to afford a colorless solid. The resulting solid was purified by column chromatography on silica gel eluting with DCM/MeOH (9:1, v/v) to afford the product as a colorless solid (8.9 mg, 16 µmol, 78 %).

NMR Spectroscopy:

^{1}H NMR (600 MHz, DMSO, 298 K, δ): 8.96 (ddd, *J* = 5.0, 1.8, 0.9 Hz, 1H), 8.66 (dd, *J* = 7.8, 1.1 Hz,

1H), 8.58 – 8.52 (m, 2H), 8.46 – 8.42 (m, 2H), 8.15 – 8.09 (m, 2H), 8.04 (ddd, *J* = 8.1, 7.5, 1.7 Hz, 1H), 8.00 (dd, *J* = 1.5, 0.7 Hz, 1H), 7.84 (ddd, *J* = 7.8, 7.3, 1.1 Hz, 1H), 7.61 (ddd, *J* = 7.8, 7.3, 1.2 Hz, 1H), 7.55 (ddd, *J* = 7.5, 4.9, 0.9 Hz, 1H), 7.40 (dd, *J* = 8.6, 1.5 Hz, 1H), 6.60 (dd, *J* = 8.7, 0.7 Hz, 1H), 3.77 (s, 3H).

^{13}C NMR (151 MHz, DMSO, 298 K, δ): 166.3, 148.9, 147.3, 143.1, 139.3, 139.0, 138.1, 136.4, 135.7, 135.6, 134.1, 132.1, 130.9, 129.8, 128.7, 128.1, 127.2, 125.6, 124.8, 124.4, 123.6, 121.4, 120.8, 116.9, 114.8, 98.4, 52.0.

^{19}F NMR (565 MHz, DMSO, 298 K, δ): −148.2, −148.3.

^{77}Se NMR (115 MHz, DMSO, 298 K, δ): 484.5.

HRMS-ESI (m/z) calculated for $C_{27}H_{19}N_2O_2Se^+$ $[M+H]^+$ 483.0606; found, 483.0609; deviation: −0.6 ppm.

Preparation of selenonium salt S62

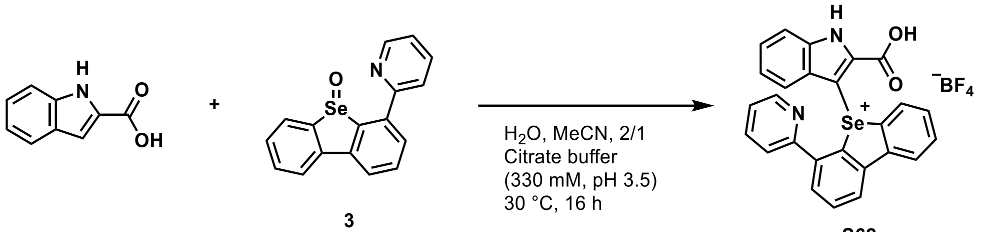

3
1.2 equiv.

S62

At 20–25 °C, a 4 mL glass vial equipped with a teflon-coated magnetic stirring bar was charged with indole-2-carboxylic acid (3.2 mg, 20 µmol, 1.0 equiv.) and selenoxide **3** (7.8 mg, 24 µmol, 1.2 equiv.). Next, citrate buffer (2.0 mL, c = 330 mM, pH 3.5) and MeCN (1.0 mL, final volume percentage 33%) were added to the vial. The vial was placed in a pre-heated heating block at 30 °C and the reaction mixture was stirred for 16 hours. The resulting mixture was diluted with 5 mL of DCM and poured into a separatory funnel. The DCM layer was washed with aqueous $NaBF_4$ solution (2 × ca. 10 ml, 5 % w/w), and with water (2 × ca. 10 ml). The DCM layer was dried over $MgSO_4$, filtered, and the solvent was removed under reduced pressure to afford a colorless solid. The resulting solid was washed with methanol (2 × ca. 3 mL) to afford the product as a colorless solid (5.6 mg, 10 µmol, 50 %).

NMR Spectroscopy:

^{1}H NMR (600 MHz, DMSO, 298 K, δ): 12.48 (s, 1H), 8.63 (ddd, *J* = 9.2, 8.0, 1.1 Hz, 2H), 8.49 (dt, *J* = 8.3, 1.0 Hz, 1H), 8.45 – 8.38 (m, 2H), 8.24 – 8.17 (m, 2H), 8.01 (ddd, *J* = 8.1, 7.5, 1.7 Hz, 1H), 7.76 – 7.70 (m, 1H), 7.57 (ddd, *J* = 7.9, 7.3, 1.2 Hz, 1H), 7.41 (ddd, *J* = 7.5, 4.9, 1.0 Hz, 1H), 7.32 (dt, *J* = 8.3, 1.0 Hz, 1H), 6.97 (t, *J* = 1.2 Hz, 1H), 6.57 (ddd, *J* = 8.3, 7.1, 1.1 Hz, 1H), 5.68 (dt, *J* = 8.5, 1.0 Hz, 1H).

^{13}C NMR (151 MHz, DMSO, 298 K, δ): 163.0, 149.4, 146.8, 144.3, 140.7, 139.0, 138.8, 137.5, 136.3, 134.3, 131.7, 130.8, 128.3, 128.0, 126.6, 125.5, 124.7, 124.1, 124.1, 123.8, 121.2, 120.7, 118.1, 117.0, 113.7, 102.0.

^{19}F NMR (565 MHz, DMSO, 298 K, δ): −148.2, −148.3.

^{77}Se NMR (115 MHz, DMSO, 298 K, δ): 500.5.

HRMS-ESI (m/z) calculated for $C_{26}H_{17}N_2O_2Se^+$ $[M-BF_4]^+$ 469.0450; found, 469.0443; deviation: +1.5 ppm.

Preparation of selenonium salt S63

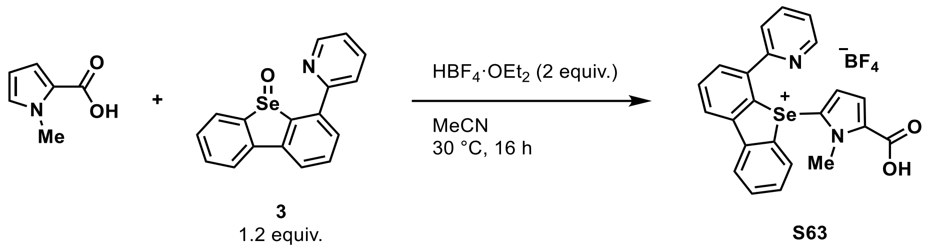

At 20–25 °C, a 4 mL glass vial equipped with a teflon-coated magnetic stirring bar was charged with *N*-methylpyrrole-2-carboxylic acid (2.5 mg, 20 µmol, 1.0 equiv.) and selenoxide **3** (7.8 mg, 24 µmol, 1.2 equiv.). Next, 2.0 mL of MeCN (c = 10 mM) were added to the vial, followed by $HBF_4·OEt_2$ (5.4 µL, 40 µmol, 2.00 equiv.). The vial was placed in a pre-heated heating block at 30 °C and the reaction mixture was stirred for 16 hours. The resulting mixture was diluted with 5 mL of DCM and poured into a separatory funnel. The DCM layer was washed with water (3 × ca. 10 ml). The DCM layer was dried over $MgSO_4$, filtered, and the solvent was removed under reduced pressure. The resulting solid was triturated and washed with diethylether (2 × ca. 3 mL) to afford the product as a colorless solid (7 mg, 14 µmol, 67 %).

NMR Spectroscopy:

^{1}H NMR (600 MHz, MeOD, 298 K, δ): 8.92 (ddd, *J* = 4.9, 1.7, 0.9 Hz, 1H), 8.42 (td, *J* = 7.5, 1.1 Hz, 2H), 8.37 – 8.31 (m, 2H), 8.07 – 8.00 (m, 3H), 7.84 – 7.78 (m, 1H), 7.65 (td, *J* = 7.6, 1.2 Hz, 1H), 7.54 (ddd, *J* = 7.5, 4.9, 1.0 Hz, 1H), 7.13 (d, *J* = 2.1 Hz, 1H), 6.37 (d, *J* = 2.1 Hz, 1H), 3.78 (s, 3H).

^{13}C NMR (151 MHz, MeOD, 298 K, δ): 167.5, 150.6, 148.3, 144.2, 140.5, 140.3, 140.0, 137.2, 135.3, 134.2, 133.3, 132.3, 132.1, 129.9, 129.4, 127.9, 126.6, 126.0, 125.5, 121.7, 114.7, 106.7, 37.6.

^{19}F NMR (565 MHz, MeOD, 298 K, δ): −154.7, −154.8.

^{77}Se NMR (115 MHz, MeOD, 298 K, δ): 483.4.

HRMS-ESI (m/z) calculated for $C_{23}H_{17}N_2O_2Se^+$ $[M-BF_4]^+$ 433.0450; found, 433.0448; deviation: +0.4 ppm.

Preparation of selenonium salt S64

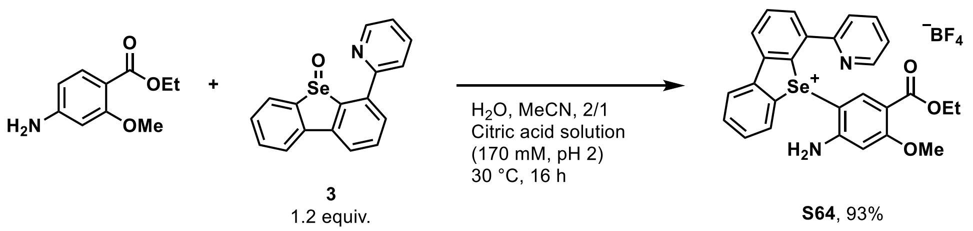

At 20–25 °C, a 4 mL glass vial equipped with a teflon-coated magnetic stirring bar was charged with ethyl 4-amino-2-methoxybenzoate (3.9 mg, 20 µmol, 1.0 equiv.) and selenoxide **3** (7.8 mg, 24 µmol, 1.2 equiv.). Next, citrate buffer (2.0 mL, c = 170 mM, pH 2) and MeCN (1.0 mL, final volume percentage 33%) were added to the vial. The vial was placed in a pre-heated heating block at 30 °C and the reaction mixture was stirred for 16 hours. The resulting mixture was diluted with 5 mL of DCM and poured into a separatory funnel. The DCM layer was washed with aqueous $NaBF_4$ solution (2 × ca. 10 ml, 5 % w/w), and with water (2 × ca. 10 ml). The DCM layer was dried over $MgSO_4$, filtered, and the solvent was removed under reduced pressure. The resulting solid was triturated and washed with diethylether (2 × ca.

3 mL) to afford the product as a colorless solid (9 mg, 18 µmol, 90 %).

NMR Spectroscopy:

^{1}H NMR (600 MHz, CD_3CN, 298 K, δ): 8.93 (ddd, *J* = 4.9, 1.7, 0.9 Hz, 1H), 8.43 (ddd, *J* = 8.0, 4.0, 1.1 Hz, 2H), 8.33 (ddd, *J* = 7.9, 1.2, 0.5 Hz, 1H), 8.29 (dt, *J* = 8.2, 1.0 Hz, 1H), 8.10 (t, *J* = 7.8 Hz, 1H), 8.06 – 8.00 (m, 2H), 7.86 – 7.81 (m, 1H), 7.70 – 7.63 (m, 1H), 7.50 (ddd, *J* = 7.5, 5.0, 1.0 Hz, 1H), 6.83 (s, 1H), 6.48 (s, 1H), 5.78 (bs, 2H), 3.92 (q, *J* = 7.1 Hz, 2H), 3.73 (s, 3H), 1.03 (t, *J* = 7.1 Hz, 3H).

^{13}C NMR (151 MHz, CD_3CN, 298 K, δ): 164.3, 164.2, 154.3, 149.5, 148.7, 144.6, 140.3, 139.7, 139.3, 137.0, 136.0, 133.9, 133.7, 132.5, 129.1, 128.7, 128.6, 126.9, 125.9, 125.9, 121.8, 112.5, 105.6, 100.9, 61.2, 56.7, 14.3.

^{19}F NMR (565 MHz, CD_3CN, 298 K, δ): −151.7, −151.8.

^{77}Se NMR (115 MHz, CD_3CN, 298 K, δ): 493.0.

HRMS-ESI (m/z) calculated for $C_{27}H_{23}N_2O_3Se^+$ [M-BF_4]$^+$ 503.0868; found, 503.0867; deviation: +0.2 ppm.

Preparation of selenonium salt S65

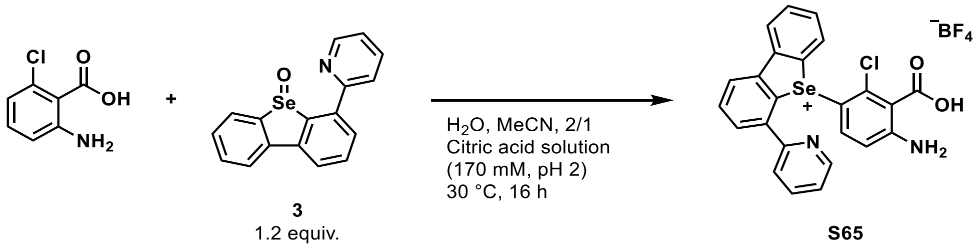

At 20–25 °C, a 4 mL glass vial equipped with a teflon-coated magnetic stirring bar was charged with ethyl 2-amino-6-chlorobenzoic acid (3.4 mg, 20 µmol, 1.0 equiv.) and selenoxide **3** (7.8 mg, 24 µmol, 1.2 equiv.). Next, citrate buffer (2.0 mL, c = 170 mM, pH 2) and MeCN (1.0 mL, final volume percentage 33%) were added to the vial. The vial was placed in a pre-heated heating block at 30 °C and the reaction mixture was stirred for 16 hours. The resulting mixture was diluted with 5 mL of DCM and poured into a separatory funnel. The DCM layer was washed with aqueous $NaBF_4$ solution (2 × ca. 10 ml, 5 % w/w), and with water (2 × ca. 10 ml). The DCM layer was dried over $MgSO_4$, filtered, and the solvent was removed under reduced pressure. The crude product was purified by column chromatography on silica gel eluting with DCM/MeOH (8:2 v/v), and the resulting solid was triturated and washed with diethylether (2 × ca. 3 mL) to afford the product as a colorless solid (8 mg, 14 µmol, 70 %).

NMR Spectroscopy:

^{1}H NMR (600 MHz, DMSO, 298 K, δ): 8.75 (ddd, *J* = 4.9, 1.7, 0.9 Hz, 1H), 8.66 (dd, *J* = 8.1, 1.1 Hz, 1H), 8.63 (dd, *J* = 7.8, 1.1 Hz, 1H), 8.56 (dt, *J* = 8.4, 1.0 Hz, 1H), 8.51 – 8.44 (m, 1H), 8.16 (t, *J* = 7.7 Hz, 1H), 8.12 (ddd, *J* = 8.1, 7.5, 1.7 Hz, 1H), 8.07 (dt, *J* = 7.7, 0.9 Hz, 1H), 7.82 (td, *J* = 7.6, 1.1 Hz, 1H), 7.64 (td, *J* = 7.6, 1.2 Hz, 1H), 7.57 (ddd, *J* = 7.5, 4.9, 1.0 Hz, 1H), 6.35 (d, *J* = 9.1 Hz, 1H), 6.12 (s, 1H), 3.37 (s, 2H).

^{13}C NMR (151 MHz, DMSO, 298 K, δ): 166.2, 151.1, 148.3, 147.2, 143.8, 140.4, 139.4, 138.2, 135.5, 134.8, 133.1, 132.1, 131.2, 128.2, 128.0, 127.6, 127.5, 126.1, 125.1, 125.0, 121.1, 120.3, 116.0, 115.4.

^{19}F NMR (565 MHz, CD_3CN, 298 K, δ): −148.2, −148.3.

^{77}Se NMR (115 MHz, CD_3CN, 298 K, δ): 522.2.

HRMS-ESI (m/z) calculated for $C_{24}H_{16}ClN_2O_2Se^+$ $[M-BF_4]^+$ 479.0060; found, 479.0060; deviation: +0.0 ppm.

Preparation of selenonium salt S66

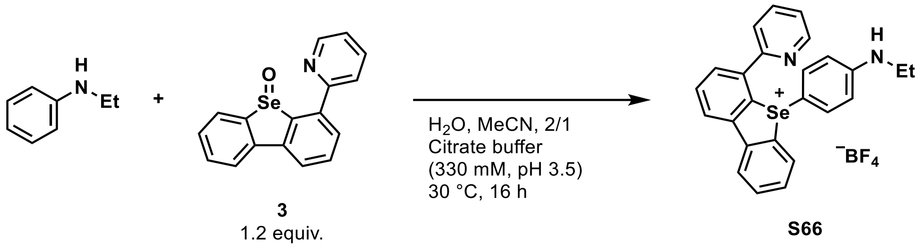

At 20–25 °C, a 4 mL glass vial equipped with a teflon-coated magnetic stirring bar was charged with *N*-ethylaniline (2.4 mg, 20 µmol, 1.0 equiv.) and selenoxide **3** (7.8 mg, 24 µmol, 1.2 equiv.). Next, citrate buffer (2.0 mL, c = 330 mM, pH 3.5) and MeCN (1.0 mL, final volume percentage 33%) were added to the vial. The vial was placed in a pre-heated heating block at 30 °C and the reaction mixture was stirred for 16 hours. The resulting mixture was diluted with 5 mL of DCM and poured into a separatory funnel. The DCM layer was washed with aqueous $NaBF_4$ solution (2 × ca. 10 ml, 5 % w/w), and with water (2 × ca. 10 ml). The DCM layer was dried over $MgSO_4$, filtered, and the solvent was removed under reduced pressure to afford the product as a colorless solid (3.3 mg, 6.4 µmol, 32 %).

NMR Spectroscopy:

^{1}H NMR (600 MHz, DMSO, 298 K, δ): δ 8.92 (ddd, *J* = 4.9, 1.8, 0.9 Hz, 1H), 8.56 (ddd, *J* = 11.6, 8.0, 1.1 Hz, 2H), 8.50 – 8.48 (m, 1H), 8.47 (ddd, *J* = 8.0, 1.2, 0.5 Hz, 1H), 8.14 (ddd, *J* = 7.8, 1.2, 0.5 Hz, 1H), 8.12 – 8.05 (m, 2H), 7.82 (ddd, *J* = 7.8, 7.3, 1.1 Hz, 1H), 7.64 (ddd, *J* = 7.8, 7.3, 1.2 Hz, 1H), 7.58 (ddd, *J* = 7.5, 4.9, 1.0 Hz, 1H), 7.14 – 7.09 (m, 2H), 6.45 – 6.40 (m, 2H), 6.32 (t, *J* = 5.2 Hz, 1H), 2.91 (qd, *J* = 7.2, 5.2 Hz, 2H).

^{13}C NMR (151 MHz, DMSO, 298 K, δ): 151.4, 148.9, 147.3, 142.8, 139.8, 139.0, 138.9, 135.2, 133.9, 131.9, 131.4, 130.8, 130.7, 128.6, 127.1, 125.6, 124.8, 124.5, 120.8, 114.3, 112.6, 36.7, 14.0.

^{19}F NMR (565 MHz, DMSO, 298 K, δ): −148.2, −148.3.

^{77}Se NMR (115 MHz, DMSO, 298 K, δ): 538.1.

HRMS-ESI (m/z) calculated for $C_{25}H_{21}N_2Se^+$ $[M-BF_4]^+$ 429.0865; found, 429.0866; deviation: −0.2 ppm.

Preparation of selenonium salt S67

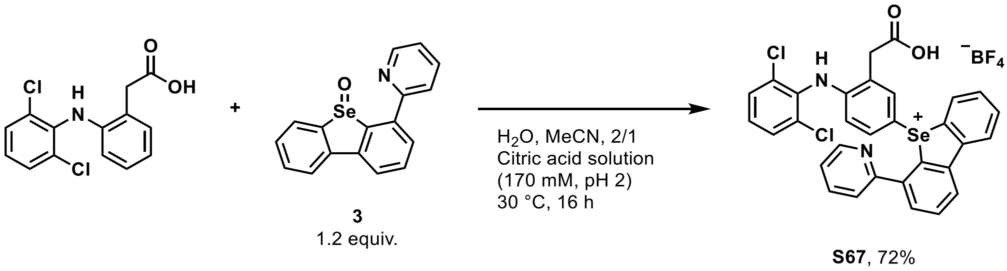

At 20–25 °C, a 4 mL glass vial equipped with a teflon-coated magnetic stirring bar was charged with diclofenac (5.9 mg, 20 µmol, 1.0 equiv.) and selenoxide **3** (7.8 mg, 24 µmol, 1.2 equiv.). Next, citrate buffer (2.0 mL, c = 170 mM, pH 2) and MeCN (1.0 mL, final volume percentage 33%) were added to the vial. The vial was placed in a pre-heated heating block at 30 °C and the reaction mixture was stirred for 16 hours. The resulting mixture was diluted with 5 mL of DCM and poured into a separatory funnel. The DCM layer was washed with aqueous $NaBF_4$ solution (2 × ca. 10 ml, 5 % w/w), and with water (2 × ca. 10 ml). The DCM layer was dried over $MgSO_4$, filtered, and the solvent was removed under reduced pressure and the residue was purified by column chromatography on silica gel eluting with DCM/MeOH (8:2, v/v) to afford the product as colorless solid (10 mg, 14 µmol, 72 %).

NMR Spectroscopy:

^{1}H NMR (600 MHz, DMSO, 298 K, δ): 11.73 (s, 1H), 8.90 (ddd, *J* = 4.9, 1.8, 0.9 Hz, 1H), 8.56 (ddd, *J* = 14.8, 7.9, 1.1 Hz, 2H), 8.48 (ddd, *J* = 9.5, 7.6, 1.1 Hz, 2H), 8.20 (dd, *J* = 7.7, 1.1 Hz, 1H), 8.12 – 8.04 (m, 2H), 7.82 (td, *J* = 7.6, 1.1 Hz, 1H), 7.64 (td, *J* = 7.6, 1.2 Hz, 1H), 7.57 (ddd, *J* = 7.5, 4.9, 1.0 Hz, 1H), 7.42 (d, *J* = 8.0 Hz, 2H), 7.17 – 7.08 (m, 2H), 6.96 (d, *J* = 2.4 Hz, 1H), 6.06 (d, *J* = 8.7 Hz, 1H), 3.13 (q, *J* = 12.2 Hz, 2H).

^{13}C NMR (126 MHz, DMSO, 298 K, δ): 172.1, 148.9, 147.6, 147.3, 142.8, 139.5, 139.1, 139.0, 136.8, 135.4, 134.0, 132.0, 131.2, 130.9, 130.4, 129.9, 129.5, 129.1, 128.8, 127.9, 127.2, 125.7, 125.6, 124.8, 124.5, 120.9, 119.4, 115.1, 45.8.

^{19}F NMR (565 MHz, DMSO, 298 K, δ): −148.2, −148.3.

^{77}Se NMR (115 MHz, DMSO, 298 K, δ) 538.1.

HRMS-ESI (m/z) calculated for $C_{31}H_{21}Cl_2N_2O_2Se^+$ $[M-BF_4]^+$ 603.0140; found, 603.0140; deviation: +0.0 ppm.

Preparation of selenonium salt S68

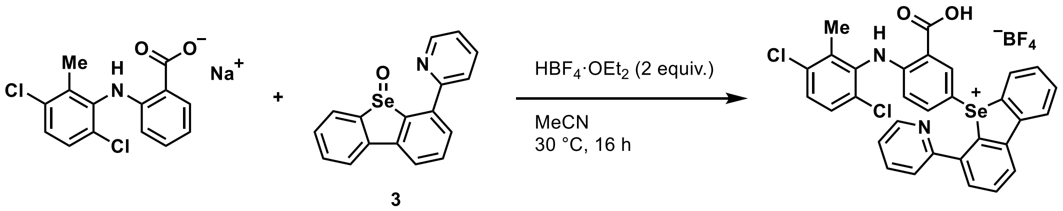

3
1.2 equiv.

S68, 87%

At 20–25 °C, a 4 mL glass vial equipped with a teflon-coated magnetic stirring bar was charged with meclofenamate sodium (6.4 mg, 20 µmol, 1.0 equiv.) and selenoxide **3** (7.8 mg, 24 µmol, 1.2 equiv.). Next, 2.0 mL of MeCN (c = 10 mM) were added to the vial, followed by $HBF_4{\cdot}OEt_2$ (8.1 µL, 60 µmol, 3.00 equiv.). The vial was placed in a pre-heated heating block at 30 °C and the reaction mixture was stirred for 16 hours. The resulting mixture was diluted with 5 mL of DCM and poured into a separatory funnel. The DCM layer was washed with water (3 × ca. 10 ml). The DCM layer was dried over $MgSO_4$, filtered, and the solvent was removed under reduced pressure. The resulting solid was triturated and washed with diethylether (2 × ca. 3 mL) to afford the product as a colorless solid (12 mg, 17 µmol, 87 %).

NMR Spectroscopy:

^{1}H NMR (600 MHz, CD_3CN, 298 K, δ): 8.81 (ddd, *J* = 4.9, 1.7, 0.9 Hz, 1H), 8.43 – 8.37 (m, 2H), 8.34 – 8.27 (m, 3H), 8.09 – 8.01 (m, 2H), 8.01 – 7.98 (m, 1H), 7.83 (tdd, *J* = 7.8, 1.1, 0.5 Hz, 1H), 7.68 – 7.63 (m, 1H), 7.53 (ddd, *J* = 7.5, 4.9, 1.0 Hz, 1H), 7.34 (dd, *J* = 8.3, 3.9 Hz, 1H), 7.24 (dq, *J* = 8.4, 0.7 Hz, 1H), 7.06 (ddd, *J* = 9.1, 6.5, 2.4 Hz, 1H), 6.14 (dd, *J* = 9.2, 0.9 Hz, 1H), 2.32 (dd, *J* = 3.9, 0.7 Hz, 3H).
^{13}C {^{1}H} NMR (151 MHz, CD_3CN, 298 K, δ): 168.9, 151.6, 149.7 (d, *J* = 1.5 Hz), 148.0, 144.2, 140.1, 140.0, 138.9, 138.0, 137.0, 135.7, 135.3, 135.2, 134.7 (d, *J* = 1.9 Hz), 134.1, 133.6, 132.3, 132.1 (d, *J* = 2.5 Hz), 131.1, 131.1, 129.7 (d, *J* = 1.8 Hz), 129.3, 128.9, 128.2, 126.6, 126.0, 125.6, 121.8, 116.4, 113.2, 20.3.

^{19}F NMR (565 MHz, CD_3CN, 298 K, δ): −152.4, −152.5.

^{77}Se NMR (115 MHz, CD_3CN, 298 K, δ): 527.8.

HRMS-ESI (m/z) calculated for $C_{31}H_{21}Cl_2N_2O_2Se^+$ $[M-BF_4]^+$ 603.0140; found, 603.0136; deviation: +0.7 ppm.

Preparation of selenonium salt S69

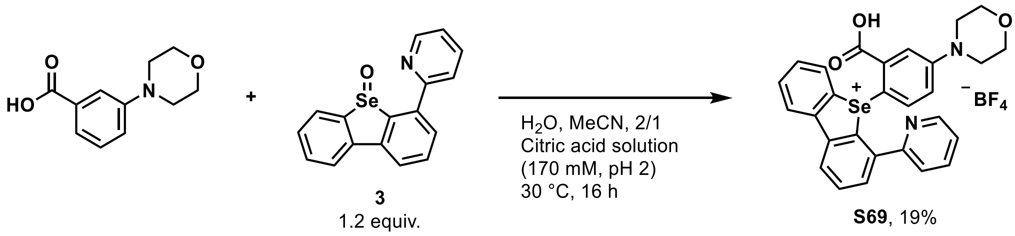

At 20–25 °C, a 4 mL glass vial equipped with a teflon-coated magnetic stirring bar was charged with 3-morpholinobenzoic acid (4.1 mg, 20 µmol, 1.0 equiv.) and selenoxide **3** (7.8 mg, 24 µmol, 1.2 equiv.). Next, citrate buffer (2.0 mL, c = 170 mM, pH 2) and MeCN (1.0 mL, final volume percentage 33%) were added to the vial. The vial was placed in a pre-heated heating block at 30 °C and the reaction mixture was stirred for 16 hours. The solvent was removed under reduced pressure and the residue was purified by column chromatography on silica gel eluting with DCM/MeOH (7:3, v/v) to afford the product as colorless solid (2.3 mg, 3.8 µmol, 19 %).

NMR Spectroscopy:

^{1}H NMR (600 MHz, DMSO, 298 K, δ): 8.58 (dd, *J* = 8.0, 1.0 Hz, 1H), 8.51 – 8.47 (m, 2H), 8.36 (ddd, *J* = 7.9, 1.2, 0.5 Hz, 1H), 8.32 – 8.28 (m, 2H), 8.07 – 8.01 (m, 2H), 7.73 – 7.64 (m, 1H), 7.56 (d, *J* = 3.1 Hz, 1H), 7.51 (ddd, *J* = 8.0, 7.3, 1.3 Hz, 1H), 7.40 (ddd, *J* = 7.5, 4.8, 1.0 Hz, 1H), 6.61 (dd, *J* = 9.1, 3.1 Hz, 1H), 6.22 (d, *J* = 9.0 Hz, 1H), 3.64 – 3.60 (m, 4H), 3.09 – 3.00 (m, 4H).

^{13}C NMR (151 MHz, DMSO, 298 K, δ): 166.9, 152.7, 150.5, 147.1, 144.0, 140.6, 138.6, 138.2, 138.1, 138.0, 133.4, 132.1, 131.5, 130.0, 129.1, 126.7, 125.2, 124.8, 124.3, 123.9, 122.6, 120.8, 116.7, 115.6, 65.8, 47.1.

^{19}F NMR (565 MHz, DMSO, 298 K, δ): −148.2, −148.3.

^{77}Se NMR (115 MHz, DMSO, 298 K, δ): 564.8.

HRMS-ESI (m/z) calculated for $C_{28}H_{23}N_2O_3Se^+$ $[M+H]^+$ 515.0868; found, 515.0872; deviation: −0.8 ppm.

Preparation of selenonium salt S70

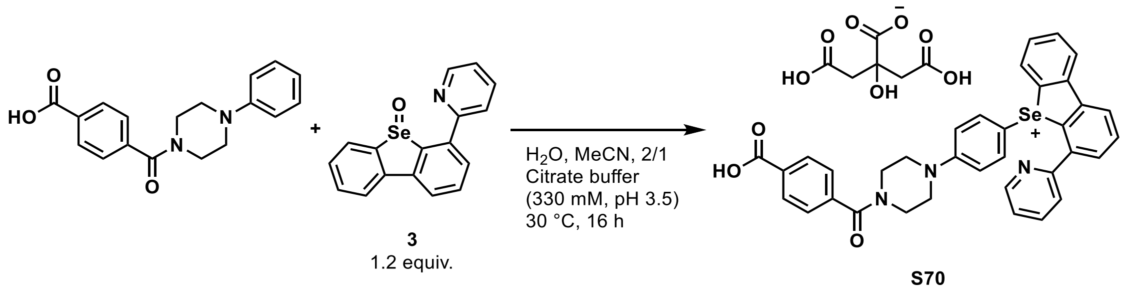

At 20–25 °C, a 4 mL glass vial equipped with a teflon-coated magnetic stirring bar was charged with *N*-ethylaniline (2.4 mg, 20 µmol, 1.0 equiv.) and selenoxide **3** (7.8 mg, 24 µmol, 1.2 equiv.). Next, citrate buffer (2.0 mL, c = 330 mM, pH 3.5) and MeCN (1.0 mL, final volume percentage 33%) were added to the vial. The vial was placed in a pre-heated heating block at 30 °C and the reaction mixture was stirred for 16 hours. After 16 hours, a white solid precipitated. Reaction mixture was centrifuged at 3000 rpm for 5 min, supernatant was removed and the resulting solid was washed with DCM (2 × ca. 3 mL) to afford the product as a colorless solid (13 mg, 16 µmol, 80 %).

NMR Spectroscopy:

^{1}H NMR (600 MHz, DMSO, 298 K, δ): 8.90 (ddd, *J* = 4.9, 1.8, 0.9 Hz, 1H), 8.58 (ddd, *J* = 13.9, 7.9, 1.1 Hz, 2H), 8.52 – 8.46 (m, 2H), 8.18 (dd, *J* = 7.8, 1.1 Hz, 1H), 8.09 (dt, *J* = 9.9, 7.8 Hz, 2H), 7.96 (d, *J* = 8.3 Hz, 2H), 7.82 (td, *J* = 7.6, 1.1 Hz, 1H), 7.64 (td, *J* = 7.6, 1.2 Hz, 1H), 7.58 (ddd, *J* = 7.5, 4.9, 1.0 Hz, 1H), 7.46 (d, *J* = 8.3 Hz, 2H), 7.27 (d, *J* = 9.2 Hz, 2H), 6.84 (d, *J* = 9.4 Hz, 2H), 3.75 – 3.01 (m, 8H), 2.54 (d, *J* = 15.1 Hz, 2H), 2.47 (d, *J* = 15.1 Hz, 2H).

^{13}C NMR (126 MHz, DMSO, 298 K, δ): δ 177.2, 171.3, 168.3, 167.5, 152.1, 148.9, 147.3, 143.0, 139.9, 139.2, 139.1, 139.0, 135.2, 134.1, 132.0, 131.1, 130.8, 130.5, 129.3, 128.7, 127.1, 127.0, 125.7, 124.9, 124.5, 120.9, 118.9, 115.6, 71.0, 46.6, 46.3, 44.5, 41.1, 40.4.

HRMS-ESI (m/z) calculated for $C_{35}H_{28}N_3O_3Se^+$ [M-$C_6H_7O_7$]$^+$ 618.1290; found, 618.1288; deviation: +0.3 ppm.

Preparation of selenonium salt S71

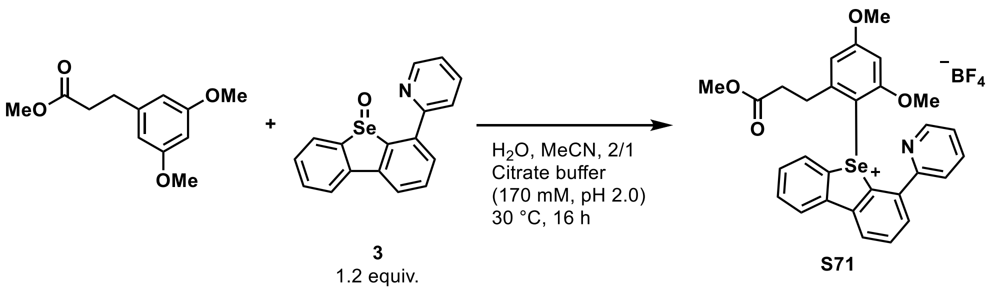

At 20–25 °C, a 4 mL glass vial equipped with a teflon-coated magnetic stirring bar was charged with methyl 3-(3,5-dimethoxyphenyl)propanoate (4.2 mg, 20 µmol, 1.0 equiv.) and selenoxide **3** (7.8 mg, 24 µmol, 1.2 equiv.). Next, citrate buffer (2.0 mL, c = 170 mM, pH 2) and MeCN (1.0 mL, final volume percentage 33%) were added to the vial. The vial was placed in a pre-heated heating block at 30 °C and the reaction mixture was stirred for 16 hours. The reaction mixture was diluted with 10 mL of DCM, poured into a separatory funnel, and washed with aqueous $NaBF_4$ solution (2 × ca. 10 ml, 5 % w/w) and

with water (2 × ca. 10 ml). The DCM layer was dried over $MgSO_4$, filtered, the solvent was removed under reduced pressure. The resulting solid was washed with methanol (2 × ca. 3 mL) to afford the product as a colorless solid (11 mg, 18 µmol, 89 %).

NMR Spectroscopy: (mixture of 2 rotamers)

^{1}**H NMR** (600 MHz, CD_3CN, 298 K, δ): ^{1}H NMR (600 MHz, CD_3CN) δ 8.68 (ddd, *J* = 5.0, 1.8, 0.9 Hz, 1H), 8.44 – 8.37 (m, 0.5H), 8.36 (dd, *J* = 7.7, 1.1 Hz, 1H), 8.32 – 8.26 (m, 2.34H), 8.22 – 8.18 (m, 1H), 8.17 – 8.13 (m, 1H), 8.07 (t, *J* = 7.7 Hz, 0.17H), 8.03 (ddd, *J* = 8.1, 7.5, 1.7 Hz, 0.17H), 7.99 – 7.95 (m, 2H), 7.87 – 7.80 (m, 0.34H), 7.79 (ddd, *J* = 7.8, 7.4, 1.1 Hz, 1H), 7.67 – 7.63 (m, 0.17H), 7.61 (ddd, *J* = 7.9, 7.3, 1.3 Hz, 1H), 7.43 (dtd, *J* = 7.5, 4.9, 1.0 Hz, 1.17H), 6.79 (d, *J* = 2.7 Hz, 0.17H), 6.67 (dd, *J* = 2.6, 0.7 Hz, 1H), 6.26 (d, *J* = 2.6 Hz, 0.17H), 6.14 (d, *J* = 2.6 Hz, 1H), 4.27 (s, 0.5H), 3.91 (ddd, *J* = 14.3, 10.5, 4.7 Hz, 1H), 3.77 (s, 0.5H), 3.77 (s, 3H), 3.71 (s, 3H), 3.46 – 3.40 (m, 1H), 3.39 (s, 0.5H), 3.16 – 3.10 (m, 1H), 3.07 – 3.00 (m, 1H), 2.85 (s, 2H), 1.85 – 1.79 (m, 0.34H), 1.65 – 1.58 (m, 0.17H), 1.46 (ddd, *J* = 16.8, 10.0, 6.8 Hz, 0.17H).

^{13}**C NMR** (151 MHz, CD_3CN, 298 K, δ): 175.1, 172.2, 165.6, 165.2, 161.9, 160.9, 150.9, 149.3, 148.8, 148.4, 147.7, 146.3, 144.9, 144.6, 141.6, 140.3, 140.0, 139.9, 137.8, 137.7, 137.2, 136.5, 135.8, 134.6, 133.5, 133.0, 132.4, 131.7, 129.5, 129.2, 129.0, 128.2, 128.0, 127.3, 126.1, 126.1, 125.7, 125.7, 124.7, 122.0, 121.8, 111.1, 110.7, 110.0, 99.7, 99.4, 58.5, 56.7, 56.7, 56.3, 52.9, 52.1, 35.8, 34.2, 30.8, 26.5.

^{19}**F NMR** (565 MHz, CD_3CN, 298 K, δ): −151.8, −151.9.

^{77}**Se NMR** (115 MHz, CD_3CN, 298 K, δ): δ 499.1, 490.1.

HRMS-ESI (m/z) calculated for $C_{28}H_{24}NO_4Se^+$ [M-BF_4]$^+$ 532.1022; found, 532.1023; deviation: −0.2 ppm.

“Off-DNA” functionalization of selenonium salts

Preparation of S72

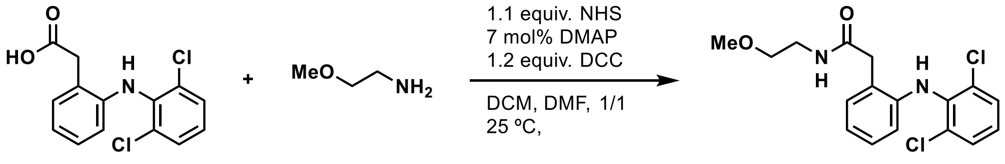

S64, 72%

Under ambient atmosphere, a 50 mL round-bottom flask equipped with a teflon-coated magnetic stirring bar was charged with diclofenac (1.18 g, 4.00 mmol, 1.00 equiv.), and DCM (10 mL, 0.40 M). Subsequently, NHS (506 mg, 4.40 mmol, 1.10 equiv.), and DMAP (34 mg, 0.28 mmol, 7 mol %) were added. Thereafter, DCC (990 mg, 4.80 mmol, 1.20 equiv.) was dissolved in DCM (10 mL) and added dropwise to the mixture. The reaction was stirred for 40 minutes at ambient temperature (20–25 °C) and monitored by TLC (EtOAc/Hex, 3:1). Upon completion of the reaction, the reaction mixture was filtered and the solvent was removed under reduced pressure. The residue was dissolved in DMF (20 mL, c = 0.2 M), and 2-methoxyethylamine (384 µL, 330 mg, 4.40 mmol, 1.10 equiv.) was added. The mixture was stirred for 30 minutes at ambient temperature (20–25 °C) and monitored by TLC (EtOAc/Hex, 3:2). Upon completion of the reaction, the mixture was diluted with EtOAc and washed with saturated solution of ammonium chloride. The organic layer was dried over magnesium sulfate and the solvent was removed

under reduced pressure. The crude product was purified by column chromatography on silica gel eluting with EtOAc/Hex (3:2, v/v) to afford the product as a colorless solid (900 mg, 64 %).

R*f* = 0.46 (silica gel, EtOAc / hexanes, 3:2 (v/v))

NMR Spectroscopy:

^{1}H NMR (600 MHz, $CDCl_3$, 298 K, δ): 7.50 (s, 1H), 7.34 (d, *J* = 8.1 Hz, 2H), 7.17 (ddd, *J* = 7.5, 1.6, 0.5 Hz, 1H), 7.14 – 7.08 (m, 1H), 7.02 – 6.95 (m, 1H), 6.95 – 6.89 (m, 1H), 6.54 – 6.49 (m, 1H), 6.09 (s, 1H), 3.68 (s, 2H), 3.45 (m, 4H), 3.34 (s, 3H).

^{13}C NMR (151 MHz, $CDCl_3$, 298 K, δ): 171.8, 143.1, 137.8, 130.7, 130.1, 129.0, 128.0, 124.8, 124.2, 121.6, 117.7, 71.2, 58.9, 41.3, 39.6.

HRMS-ESI (m/z) calculated for $C_{17}H_{18}Cl_2N_2O_2$ [M^+], 352.0740; found, 352.0742; deviation: −0.6 ppm.

Preparation of selenonium salt S73

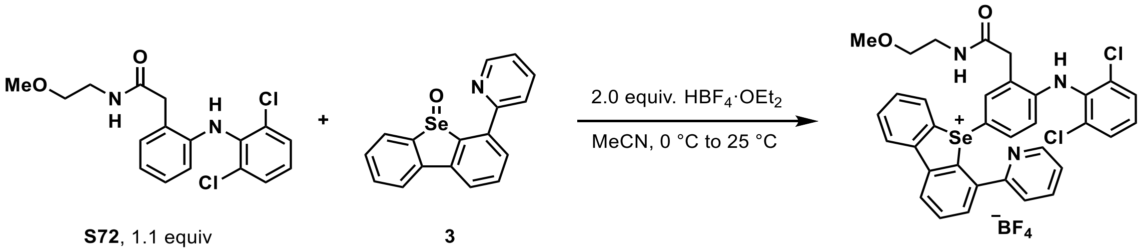

S72, 1.1 equiv **3** **S73**, 46%

Under ambient atmosphere, a 20 mL vial equipped with a teflon-coated magnetic stirring bar was charged with selenoxide **3** (300 mg, 0.925 mmol, 1.00 equiv.) and MeCN (9.25 mL, c = 0.1 M). Subsequently, **S72** (360 mg, 1.02 mmol, 1.10 equiv.) was added at room temperature (20–25 °C). The mixture was cooled to 0 °C using an ice water bath, after which $HBF_4 \cdot OEt_2$ (252 µL, 1.85 mmol, 2.00 equiv.) was added dropwise. The mixture was allowed to warm to 20–25 °C and stirred overnight. To the reaction was added Et_2O in portions, causing a colorless solid to precipitate which was collected via filtration. The solid was washed with Et_2O and dried in vacuo to afford the product as a colorless solid (316 mg, 46 %).

NMR Spectroscopy:

^{1}H NMR (600 MHz, DMSO, 298 K, δ): 8.96 (ddd, *J* = 4.9, 1.7, 0.9 Hz, 1H), 8.57 (dd, *J* = 7.8, 1.0 Hz, 1H), 8.54 (dd, *J* = 8.1, 1.1 Hz, 1H), 8.51 – 8.45 (m, 3H), 8.39 (t, *J* = 5.6 Hz, 1H), 8.22 (ddd, *J* = 7.8, 1.2, 0.5 Hz, 1H), 8.11 (ddd, *J* = 8.1, 7.5, 1.7 Hz, 1H), 8.06 (t, *J* = 7.7 Hz, 1H), 7.87 – 7.81 (m, 1H), 7.65 (ddd, *J* = 7.9, 7.3, 1.2 Hz, 1H), 7.60 (ddd, *J* = 7.5, 4.9, 1.0 Hz, 1H), 7.50 (d, *J* = 8.1 Hz, 2H), 7.40 (d, *J* = 2.4 Hz, 1H), 7.23 (t, *J* = 8.1 Hz, 1H), 7.00 (dd, *J* = 8.8, 2.4 Hz, 1H), 6.07 (d, *J* = 8.9 Hz, 1H), 3.47 (s, 2H), 3.31 (dd, *J* = 5.9, 5.2 Hz, 2H), 3.24 (s, 3H), 3.22 – 3.12 (m, 2H).

^{13}C NMR (151 MHz, CD_3CN, 298 K, δ): 170.2, 149.1, 147.3, 146.3, 142.8, 139.3, 139.1, 135.3, 135.3, 134.0, 132.1, 131.8, 131.5, 131.5, 130.9, 129.2, 129.0, 127.3, 127.1, 125.7, 125.1, 124.8, 124.5, 123.9, 122.9, 122.8, 121.0, 120.7, 120.4, 115.2, 70.5, 58.0, 38.7, 38.7.

^{19}F NMR (565 MHz, CD_3CN, 298 K, δ): −148.2, −148.3.

^{77}Se NMR (115 MHz, CD_3CN, 298 K, δ): δ 538.2.

HRMS-ESI (m/z) calculated for $C_{34}H_{28}Cl_2N_3O_2Se^+$ [$M-BF_4$]$^+$, 660.0718; found, 660.0721;

deviation: −0.5 ppm.

"Off-DNA" Iodination of selenonium salt (S74)

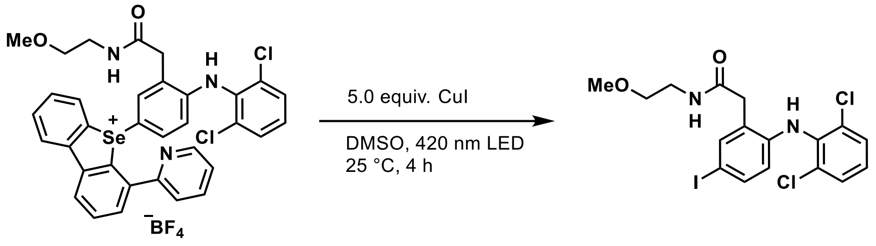

S73 **S74**, 20%

Under ambient atmosphere, a 4 mL vial equipped with a teflon-coated magnetic stirring bar was charged with selenonium salt **S73** (80 mg, 0.11 mmol, 1.0 equiv.), CuI (102 mg, 0.535 mmol, 5.00 equiv.) and DMSO (2 mL, c = 0.05 M). The reaction vial was purged with argon, transferred into a Penn PhD Photoreactor M2 and irradiated at 420 nm with stirring for 4 hours. After 4 hours, the solvent was removed under reduced pressure and the crude product was purified by column chromatography on silica gel eluting with hexanes/EtOAc (gradient from 9:1 to 7:3 v/v) to afford the product as a colorless solid (10 mg, 20 %).

NMR Spectroscopy:

> **^{1}H NMR** (500 MHz, CD_2Cl_2, 298 K, δ): 8.01 (s, 1H), 7.48 (d, *J* = 2.1 Hz, 1H), 7.36 (dd, *J* = 7.3, 3.1 Hz, 3H), 7.03 (t, *J* = 8.1 Hz, 1H), 6.24 (d, *J* = 8.5 Hz, 1H), 6.17 (s, 1H), 3.59 (s, 2H), 3.49 – 3.41 (m, 4H), 3.34 (s, 3H).

> **^{13}C NMR** (126 MHz, CD_2Cl_2, 298 K, δ): 171.6, 143.9, 139.3, 137.6, 136.8, 130.5, 129.4, 128.0, 125.1, 119.6, 83.2, 71.2, 59.0, 40.9, 40.0.

HRMS-ESI (m/z) calculated for $C_{17}H_{17}Cl_2IN_2O_2$ [M^+] 478.9785; found 478.9785; deviation: +0.0 ppm.

"Off-DNA" Palladium mediated coupling of selenonium salt (S75)

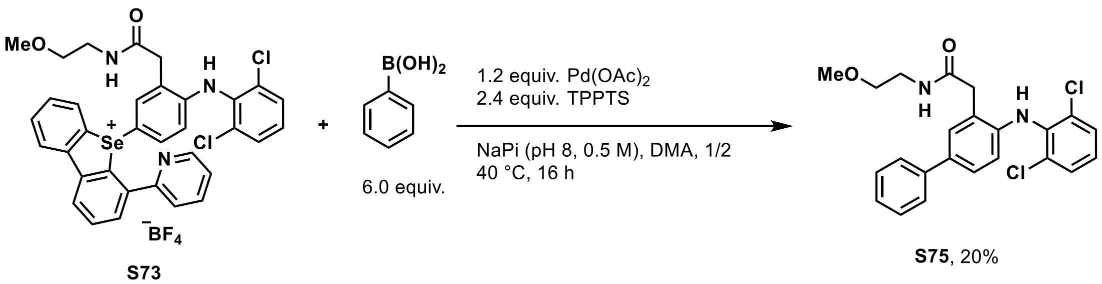

S73 **S75**, 20%

Under ambient atmosphere, a 4 mL vial equipped with a Teflon-coated magnetic stirring bar was charged with selenonium salt **S73** (50 mg, 0.067 mmol, 1.0 equiv.), phenylboronic acid (49 mg, 0.40 mmol, 6.0 equiv.) and DMA (447 µL, c = 0.15 M). Another 4 mL vial with a Teflon-coated magnetic stirring bar was charged with palladium acetate (18 mg, 80 µmol, 1.2 equiv.), 3,3',3''-phosphanetriyltris(benzenesulfonic acid) trisodium salt (TPPTS) (91 mg, 0.16 mmol, 2.40 equiv.), DMA (447 µL) and NaPi buffer (447 µL, pH 8, c = 500 mM). Both solutions were stirred at ambient temperature (20–25 °C) for 10 minutes and mixed in 4 mL vial (final concentration selenonium salt = 50 mM). The vial was placed at 40 °C in a pre-heated heating block and the reaction mixture was stirred for 16 hours. The solvent was removed under reduced pressure and the crude product was purified by column chromatography on silica gel eluting with

hexanes/EtOAc (gradient from 9:1 to 7:3 v/v) to afford the product as a colorless solid (6 mg, 20 %).

NMR Spectroscopy:

^{1}H NMR (500 MHz, CD_2Cl_2, 298 K, δ): 7.96 (s, 1H), 7.59 – 7.55 (m, 2H), 7.44 – 7.38 (m, 5H), 7.34 (dd, *J* = 8.3, 2.2 Hz, 1H), 7.32 – 7.27 (m, 1H), 7.04 (t, *J* = 8.1 Hz, 1H), 6.55 (dd, *J* = 8.3, 1.5 Hz, 1H), 6.23 (s, 1H), 3.73 (s, 2H), 3.49 – 3.43 (m, 4H), 3.33 (s, 3H).

^{13}C NMR (126 MHz, CD_2Cl_2, 298 K, δ): 171.6, 142.8, 140.6, 137.7, 134.0, 130.1, 129.2, 128.9, 128.7, 126.7, 126.5, 126.2, 125.4, 124.4, 117.5, 70.9, 58.5, 41.1, 39.5.

HRMS-ESI (m/z) calculated for $C_{24}H_{24}Cl_2N_2O_2S$ $[M+Na]^+$ 451.0951; found 451.0951; deviation: 0.0 ppm.

"Off-DNA" C-S coupling of selenonium salt (S76)

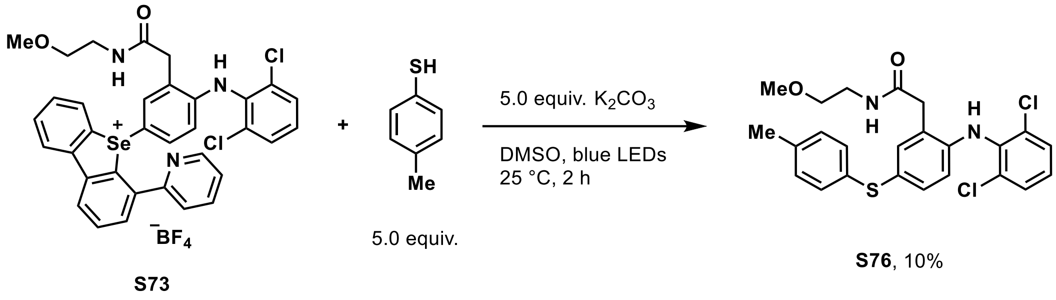

Under ambient atmosphere, a 4 mL vial equipped with a teflon-coated magnetic stirring bar was charged with selenonium salt **S73** (80 mg, 0.11 mmol, 1.0 equiv.), 4-Methylthiophenol (11 µL, 67 mg, 0.54 mmol, 5.0 equiv.), K_2CO_3 (74 mg, 0.54 mmol, 5.0 equiv.) and DMSO (2.0 mL, c = 0.050 M). The reaction vial was purged with argon, transferred into a Penn PhD Photoreactor M2 and irradiated at 450 nm with stirring for 2 hours. Upon completion of the reaction, the solvent was removed under reduced pressure. The crude product was purified by column chromatography on silica gel eluting with toluene/EtOAc (8:2 v/v) to afford the product as a colorless solid (5 mg, 10 %).

NMR Spectroscopy:

^{1}H NMR (600 MHz, CD_2Cl_2, 298 K, δ): 8.08 (s, 1H), 7.37 (d, *J* = 8.1 Hz, 2H), 7.24 (d, *J* = 2.2 Hz, 1H), 7.17 – 7.14 (m, 2H), 7.13 (ddd, *J* = 8.4, 2.2, 0.5 Hz, 1H), 7.10 – 7.07 (m, 2H), 7.03 (t, 1H), 6.43 (d, 1H), 6.17 (s, 1H), 3.61 (s, 2H), 3.46 – 3.41 (m, 4H), 3.32 (s, 3H), 2.30 (s, 3H).

^{13}C NMR (151 MHz, CD_2Cl_2, 298 K, δ): 171.8, 143.7, 137.8, 136.8, 135.2, 134.4, 132.6, 130.5, 130.2, 129.9, 129.3, 126.4, 126.2, 124.9, 118.3, 71.2, 58.9, 41.2, 39.9, 21.1.

HRMS-ESI (m/z) calculated for $C_{24}H_{24}Cl_2N_2O_2S$ $[M^+]$ 497.0829; found 497.0828; deviation: +0.2 ppm.

Synthesis of DNA-conjugated selenonium salts by amide coupling

Synthesis of DNA-conjugated selenonium salt 11 by amide coupling

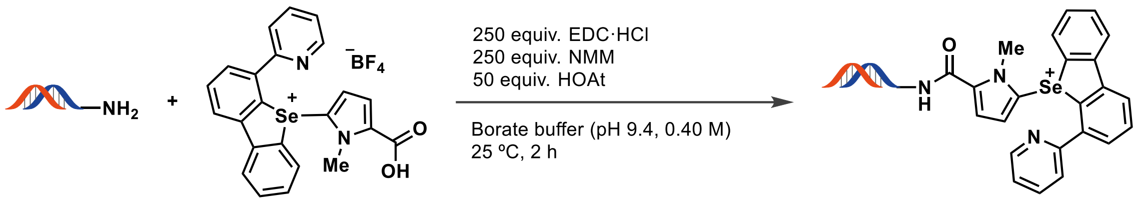

S63
500 equiv.

11, >95%

Exact Mass: 5597.059
Molecular Weight: 5598.879

At 20–25 °C, 20 µL of **S63** stock solution (500 mM, 10 µmol, 500 equiv.) in DMSO was mixed with 10 µL of a HOAt stock solution in DMSO (100 mM, 1.0 µmol, 50 equiv.), and 10 µL of an EDC·HCl stock solution in DMSO (500 mM, 5.0 µmol, 250 equiv.) in a 1.5 mL Eppendorf tube. The mixture was vortexed for 5 seconds. Next, 10 µL of a NMM stock solution (500 mM, 5.0 µmol, 250 equiv.) in DMSO was added. The mixture was vortexed for 5 seconds again, and left standing at 20–25 °C for 15 min. In another 1.5 mL Eppendorf tube, 60 µL of HP–AOP–NH_2 (0.33 mM, 20 nmol, 1.0 equiv.) in borate buffer (pH 9.4, c = 400 mM) was added, and the premix of acid, HOAt, EDC·HCl and NMM was added over the solution. The mixture was vortexed for 5 seconds, transferred into a Thermocycler at 25 °C, and incubated at 25 °C for 2 hours at 600 rpm.

Next, 10 µL of a 5 M solution of NaCl in water and 360 µL of ethanol at −20 °C were added to precipitate the DNA conjugate. The Eppendorf tube was placed in the freezer (−20 °C) for at least 1 hour, and then it was centrifuged at 4 °C and10000 x g for at least 30 minutes. The supernatant was removed, the pellet was redissolved in 300 µL of water, and the procedure was repeated again. The remaining pellet was then dried under a flow of nitrogen, redissolved with 50 µL of water. An aliquot of 1 µL of the reaction mixture was diluted to 40 µL with water for LC–MS analysis.

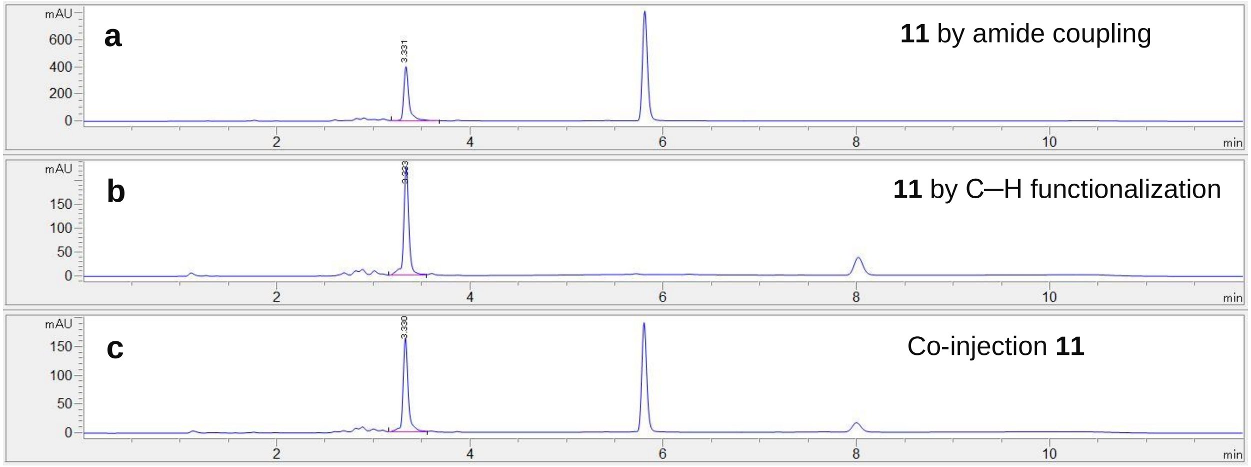

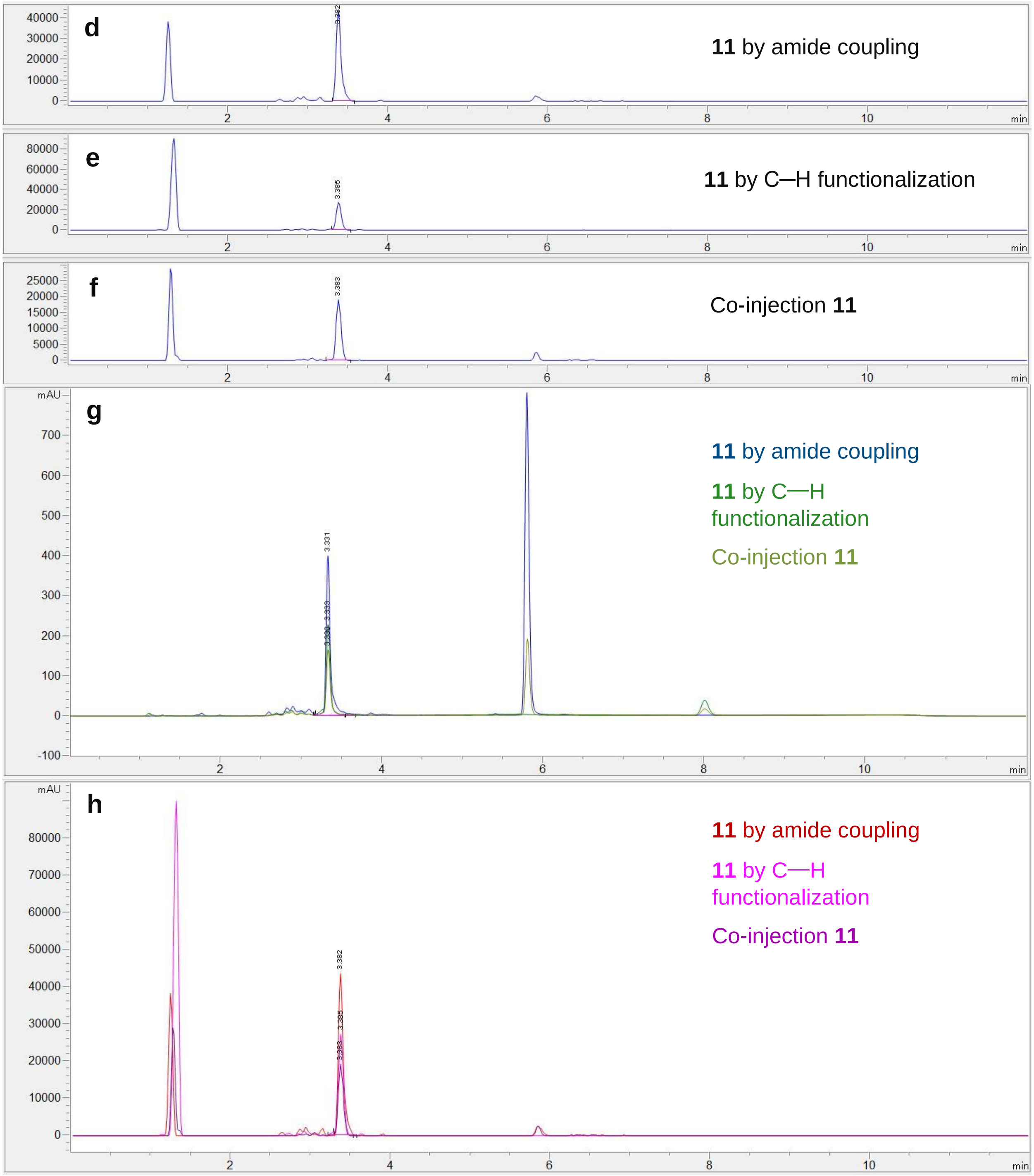

Figure S145. Analytical HPLC trace of **11** with HPLC Method A. DAD chromatograms of **11** at 260 nm. (a) By amide coupling (b) by C─H functionalization (c) Co-injection of **11** by synthesized by both methods. TIC chromatograms. (d) By amide coupling (e) by C─H functionalization (f) Co-injection of **11** by synthesized by both methods. (g) Overlay of DAD chromatograms of **11** by different synthetic methods. (g) Overlay of TIC chromatograms of **11** by different synthetic method.

Synthesis of DNA-conjugated selenonium salt 15 by amide coupling

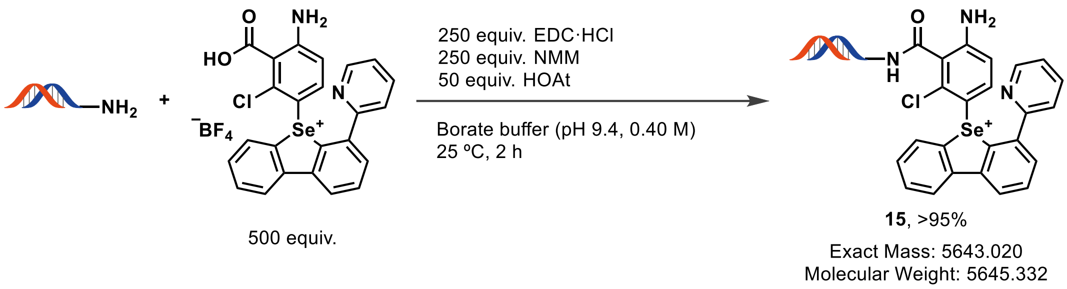

At 20–25 °C, 20 µL of **S65** stock solution (500 mM, 10 µmol, 500 equiv.) in DMSO was mixed with 10 µL of a HOAt stock solution in DMSO (100 mM, 1.0 µmol, 50 equiv.), and 10 µL of an EDC·HCl stock solution in DMSO (500 mM, 5.0 µmol, 250 equiv.) in a 1.5 mL Eppendorf tube. The mixture was vortexed for 5 seconds. Next, 10 µL of a NMM stock solution (500 mM, 5.0 µmol, 250 equiv.) in DMSO was added. The mixture was vortexed for 5 seconds again, and left standing at 20–25 °C for 15 min. In another 1.5 mL Eppendorf tube, 60 µL of HP–AOP–NH_2 (0.33 mM, 20 nmol, 1.0 equiv.) in borate buffer (pH 9.4, c = 400 mM) was added, and the premix of acid, HOAt, EDC·HCl and NMM was added over the solution. The mixture was vortexed for 5 seconds, transferred into a Thermocycler at 25 °C, and incubated at 25 °C for 2 hours at 600 rpm.

Next, 10 µL of a 5 M solution of NaCl in water and 360 µL of ethanol at −20 °C were added to precipitate the DNA conjugate. The Eppendorf tube was placed in the freezer (−20 °C) for at least 1 hour, and then it was centrifuged at 4 °C and 10000 x g for at least 30 minutes. The supernatant was removed, the pellet was redissolved in 300 µL of water, and the procedure was repeated again. The remaining pellet was then dried under a flow of nitrogen, redissolved with 50 µL of water. An aliquot of 1 µL of the reaction mixture was diluted to 40 µL with water for LC–MS analysis.

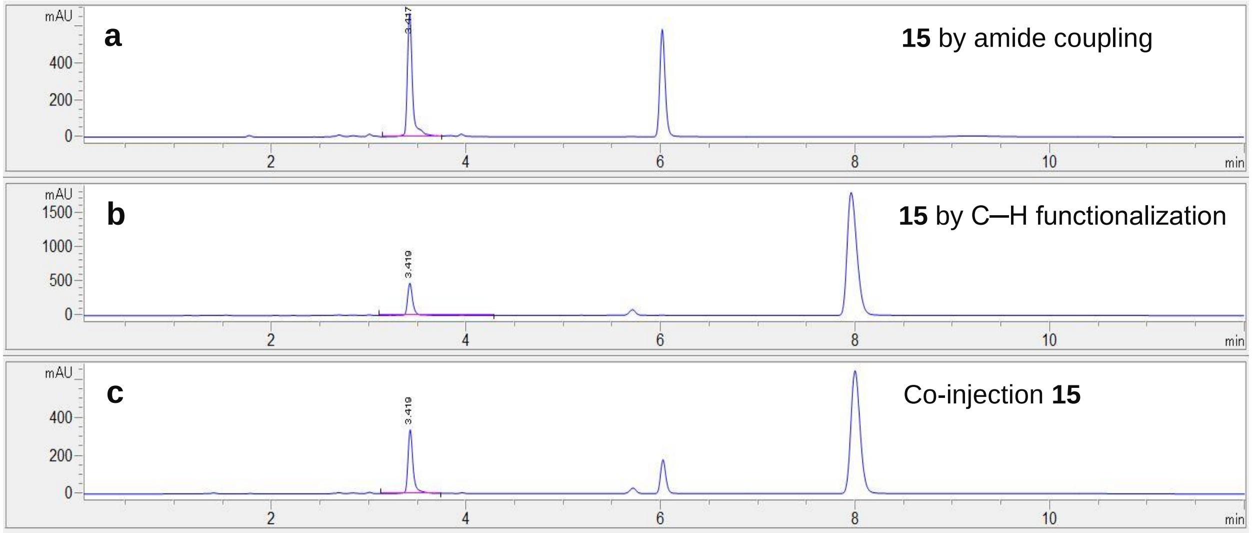

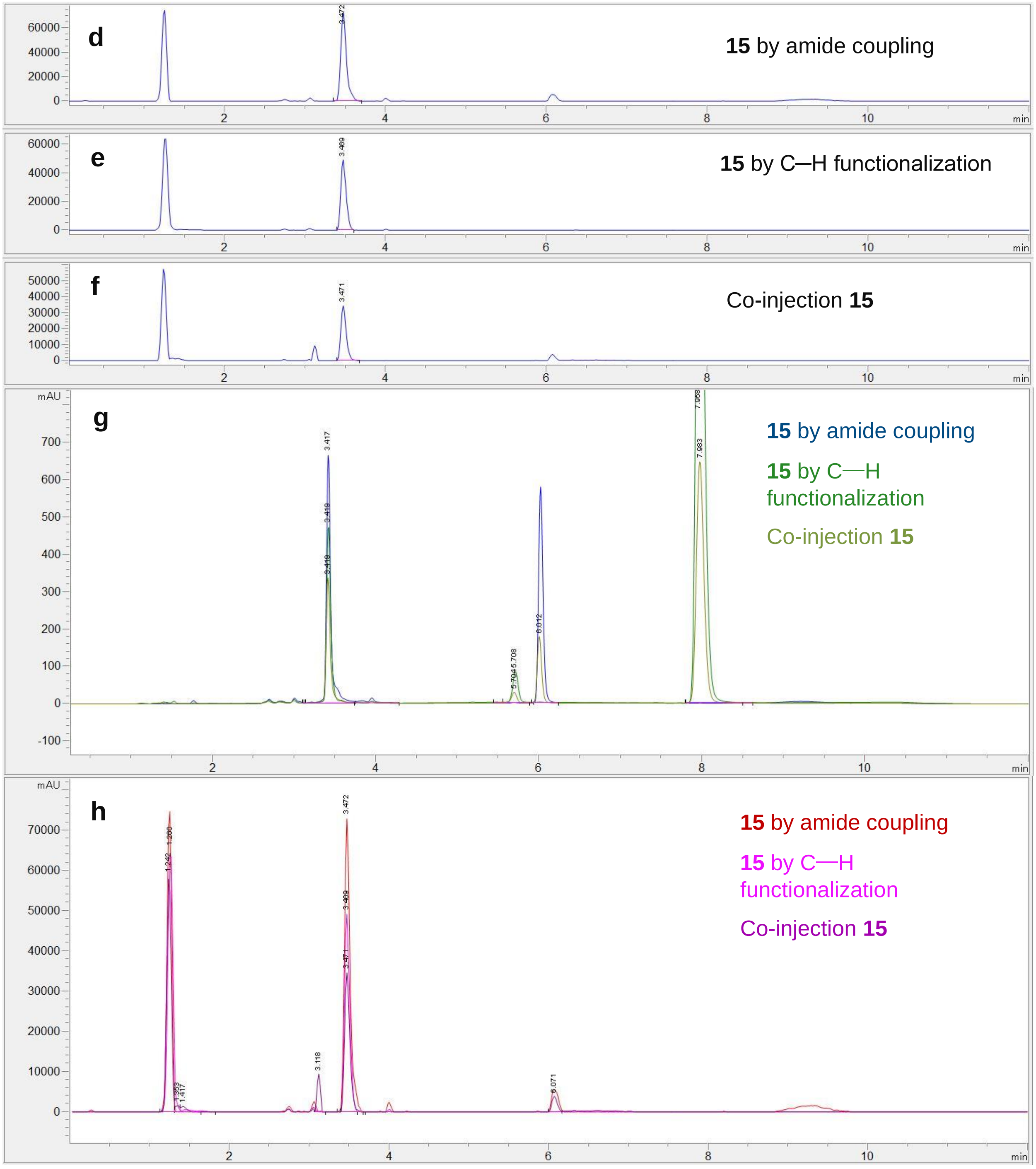

Figure S146. Analytical HPLC trace of **15** with HPLC Method A. DAD chromatograms of **15** at 260 nm. (a) By amide coupling (b) by C─H functionalization (c) Co-injection of **15** by synthesized by both methods. TIC chromatograms. (d) By amide coupling (e) by C─H functionalization (f) Co-injection of **15** by synthesized by both methods. (g) Overlay of DAD chromatograms of **15** by different synthetic methods. (g) Overlay of TIC chromatograms of **15** by different synthetic method.

Synthesis of DNA-conjugated selenonium salt 25 by amide coupling

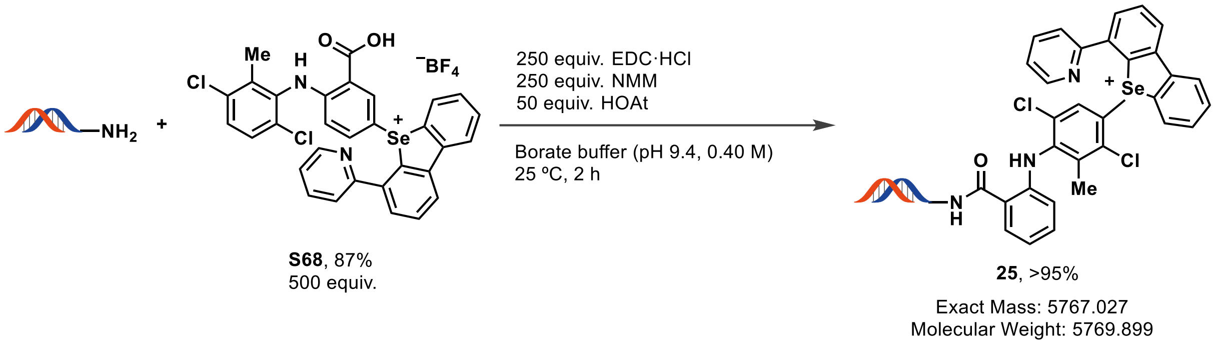

At 20–25 °C, 20 µL of **S68** stock solution (500 mM, 10 µmol, 500 equiv.) in DMSO was mixed with 10 µL of a HOAt stock solution in DMSO (100 mM, 1.0 µmol, 50 equiv.), and 10 µL of an EDC·HCl stock solution in DMSO (500 mM, 5.0 µmol, 250 equiv.) in a 1.5 mL Eppendorf tube. The mixture was vortexed for 5 seconds. Next, 10 µL of a NMM stock solution (500 mM, 5.0 µmol, 250 equiv.) in DMSO was added. The mixture was vortexed for 5 seconds again, and left standing at 20–25 °C for 15 min. In another 1.5 mL Eppendorf tube, 60 µL of HP–AOP–NH_2 (0.33 mM, 20 nmol, 1.0 equiv.) in borate buffer (pH 9.4, c = 400 mM) was added, and the premix of acid, HOAt, EDC·HCl and NMM was added over the solution. The mixture was vortexed for 5 seconds, transferred into a Thermocycler at 25 °C, and incubated at 25 °C for 2 hours at 600 rpm.

Next, 10 µL of a 5 M solution of NaCl in water and 360 µL of ethanol at −20 °C were added to precipitate the DNA conjugate. The Eppendorf tube was placed in the freezer (−20 °C) for at least 1 hour, and then it was centrifuged at 4 °C and 10000 x g for at least 30 minutes. The supernatant was removed, the pellet was redissolved in 300 µL of water, and the procedure was repeated again. The remaining pellet was then dried under a flow of nitrogen, redissolved with 50 µL of water. An aliquot of 1 µL of the reaction mixture was diluted to 40 µL with water for LC–MS analysis.

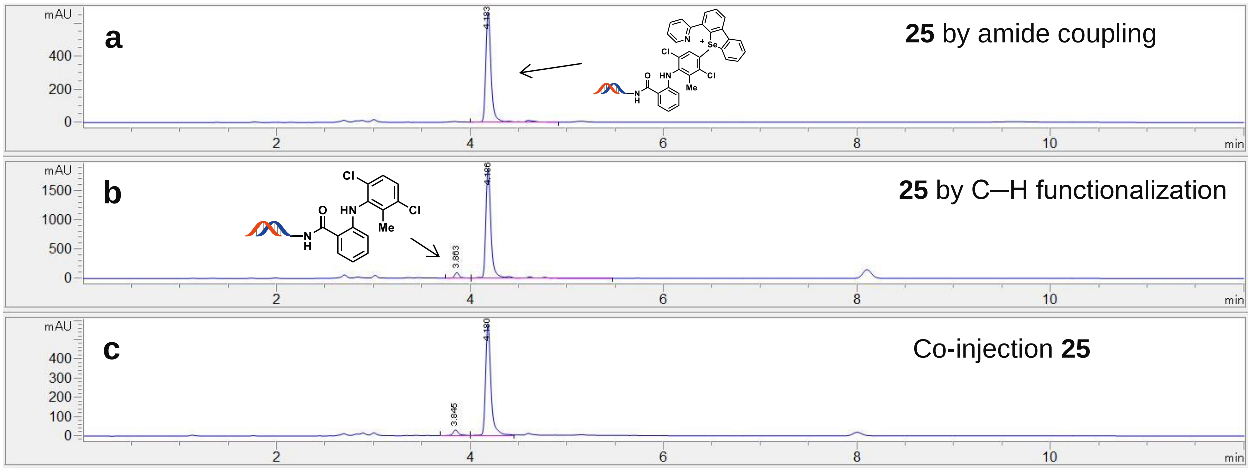

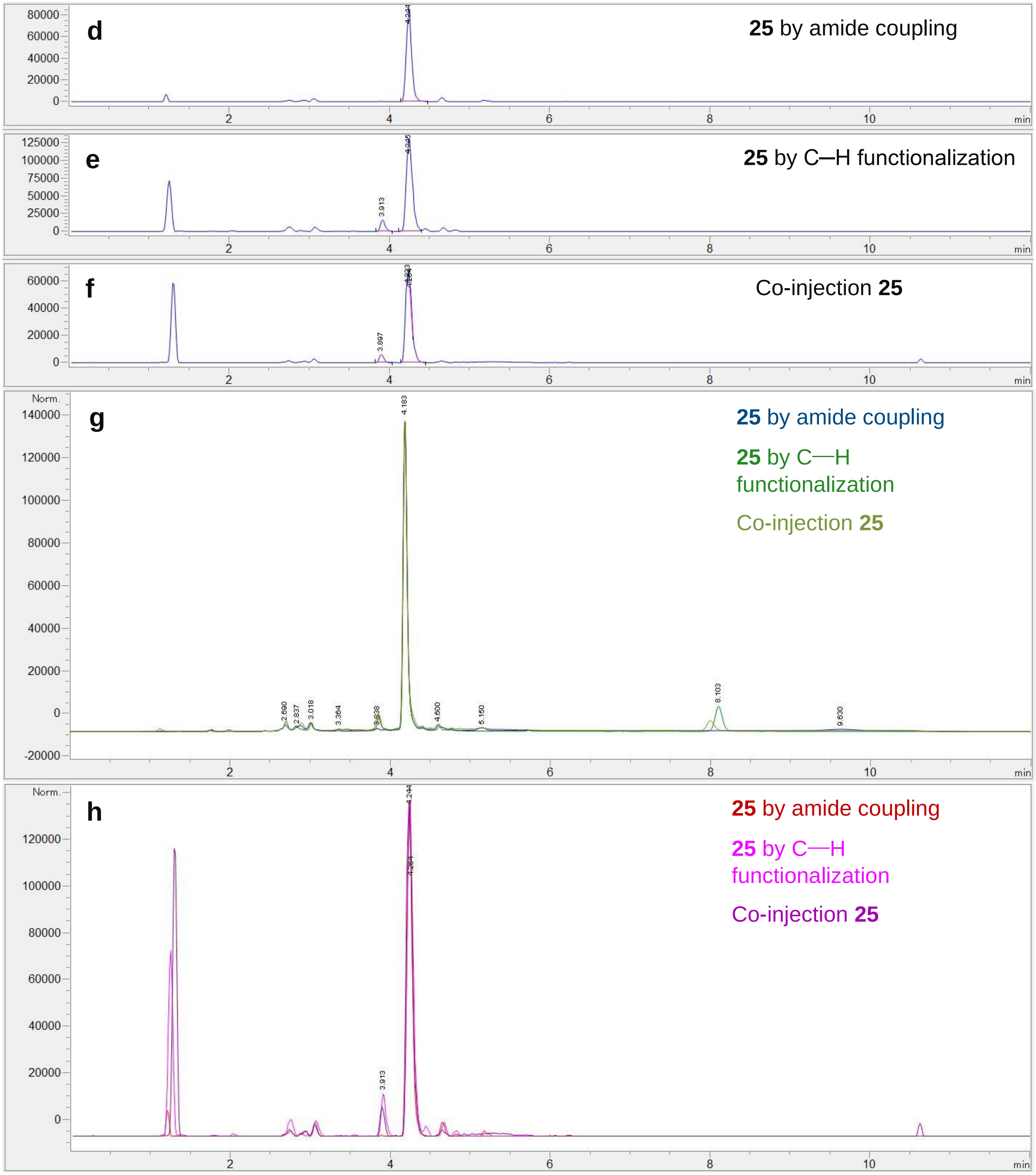

Figure S147. Analytical HPLC trace of **25** with HPLC Method A. DAD chromatograms of **25** at 260 nm. (a) By amide coupling (b) by C─H functionalization (c) Co-injection of **25** by synthesized by both methods. TIC chromatograms. (d) By amide coupling (e) by C─H functionalization (f) Co-injection of **25** by synthesized by both methods. (g) Overlay of DAD chromatograms of **25** by different synthetic methods. (g) Overlay of TIC chromatograms of **25** by different synthetic method.

NMR DATA

NMR-Characterization of “off DNA” selenonium salts

^{1}H NMR of **S1**
600 MHz, $CDCl_3$, 298 K

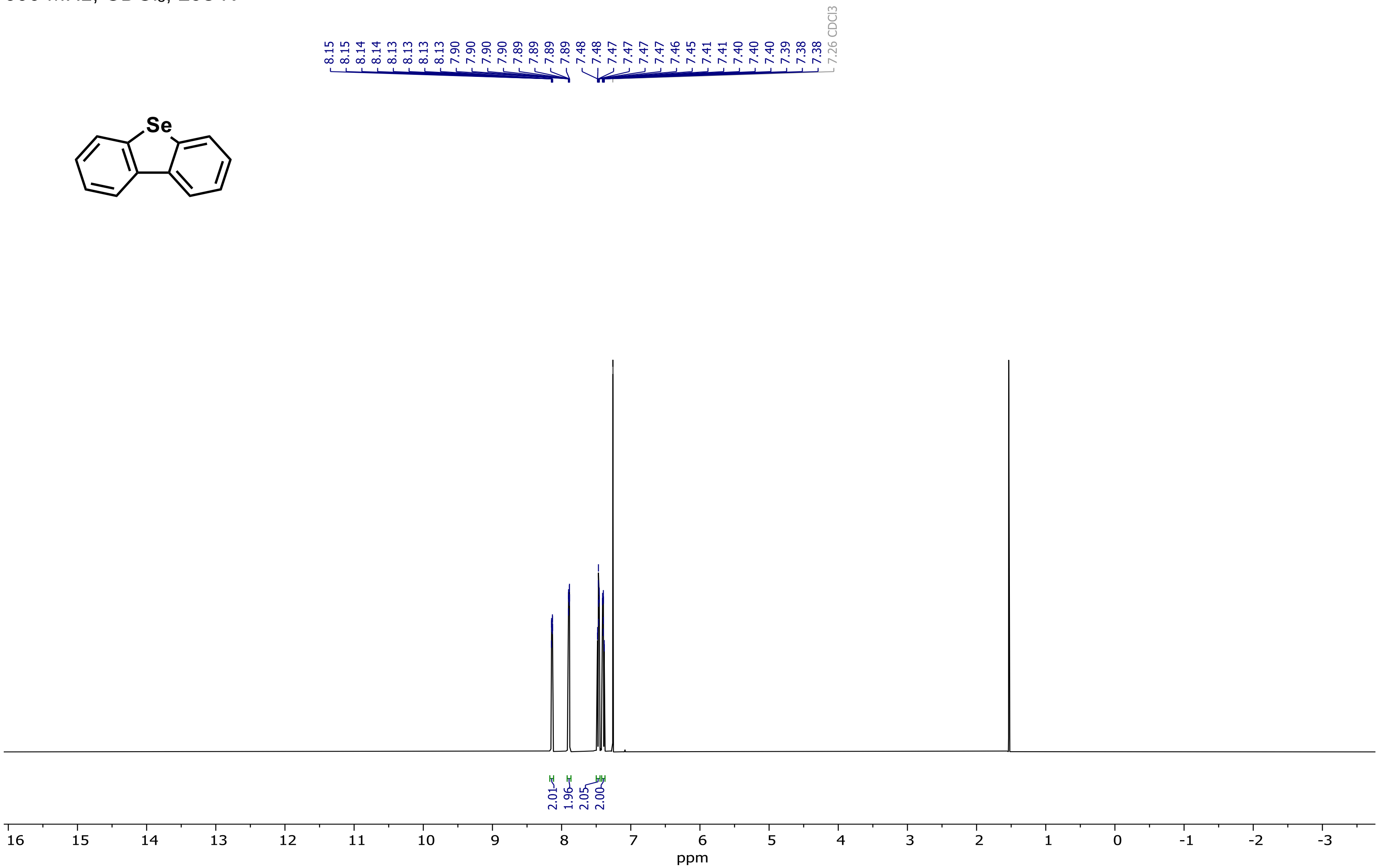

^{13}C NMR of **S1**
151 MHz, $CDCl_3$, 298 K

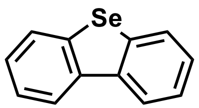

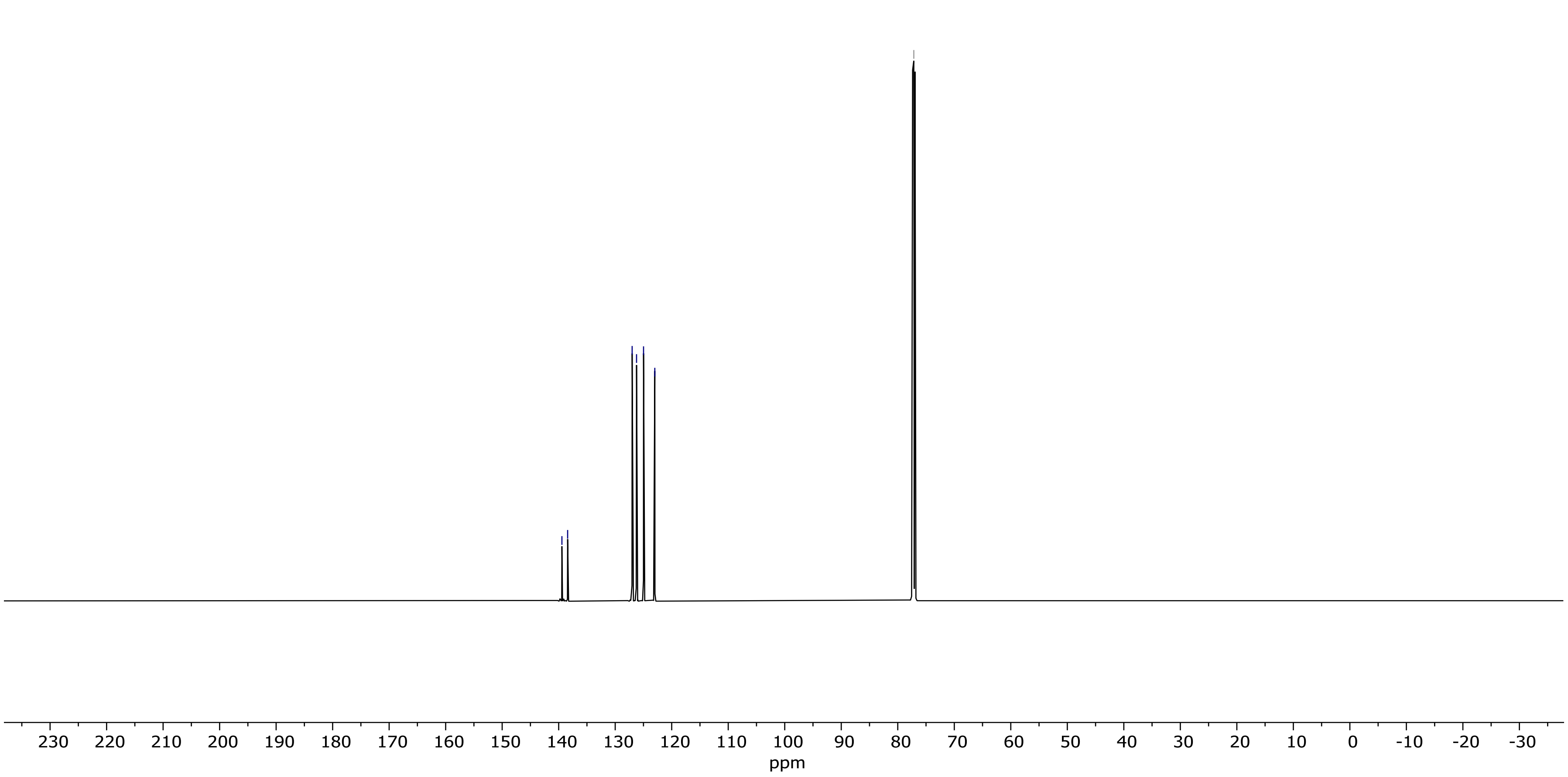

^{77}Se NMR of **S1**
115 MHz, $CDCl_3$, 298 K

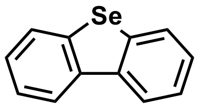

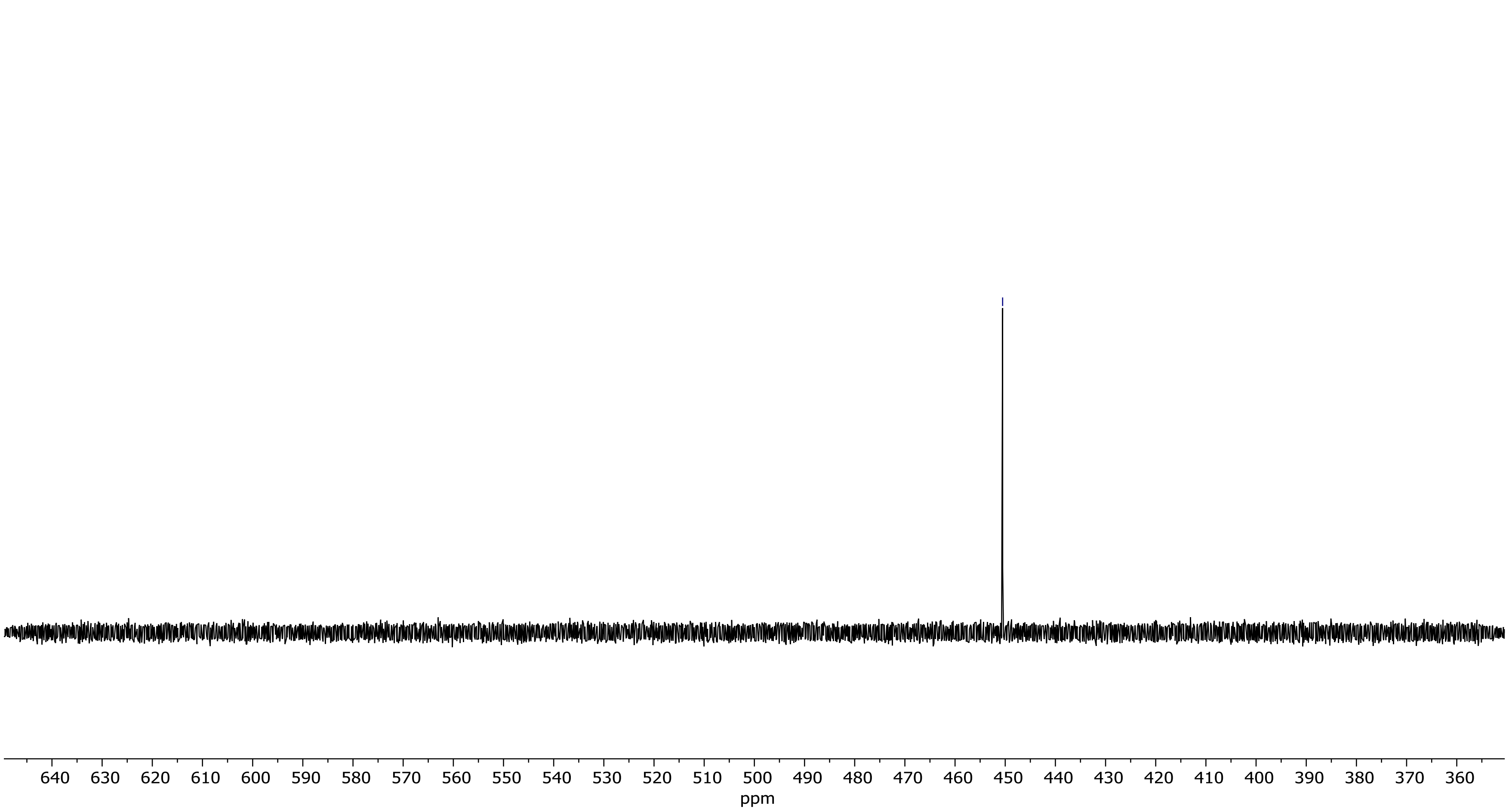

^{1}H NMR of **2**
600 MHz, DMSO, 298 K

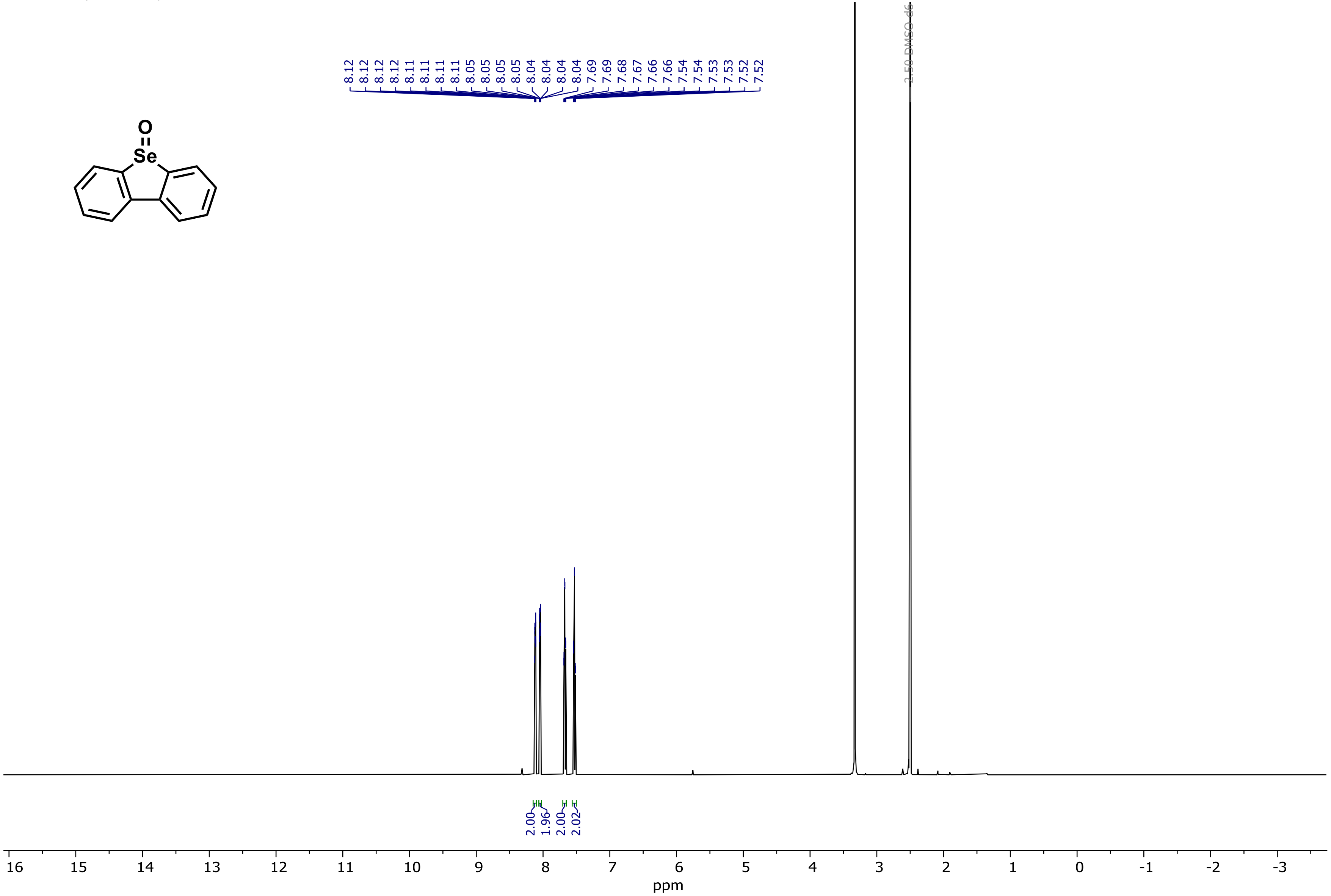

^{13}C NMR of **2**
151 MHz, DMSO, 298 K

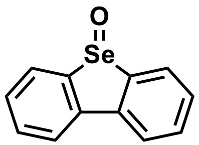

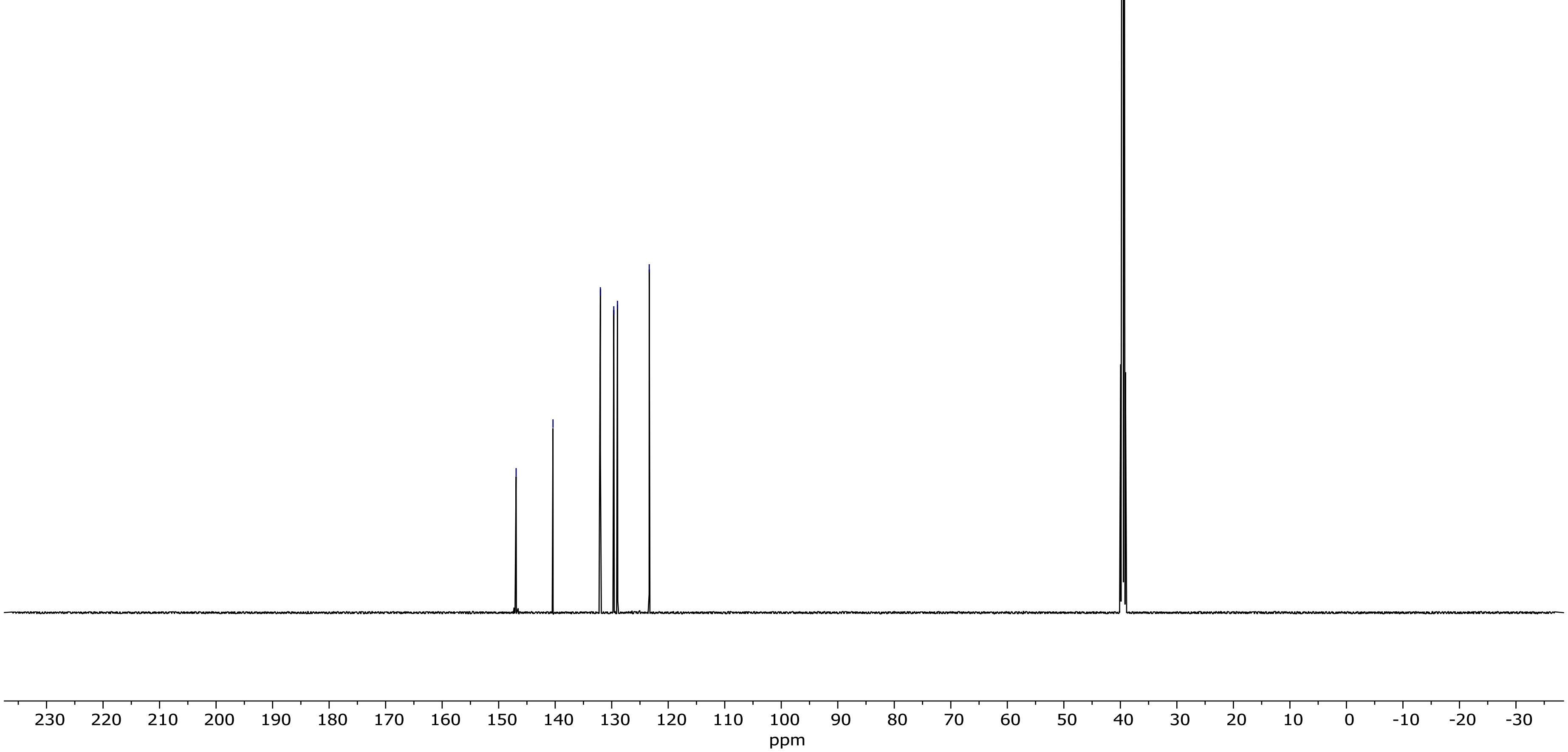

^{77}Se NMR of **2**
115 MHz, DMSO, 298 K

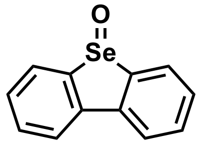

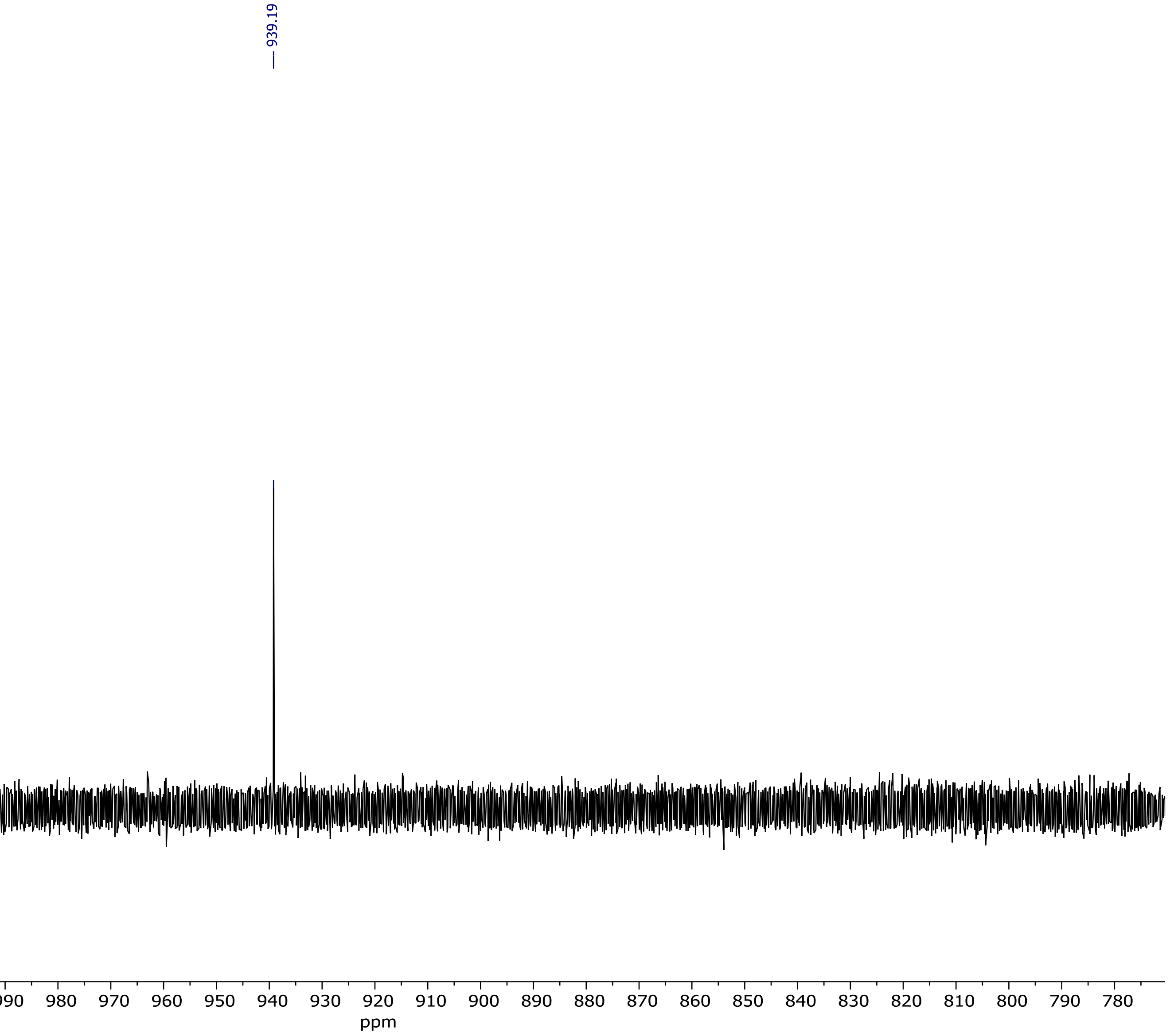

^{1}H NMR of **S3**
600 MHz, $CDCl_3$, 298 K

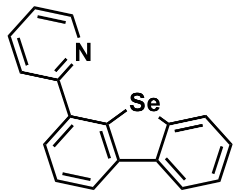

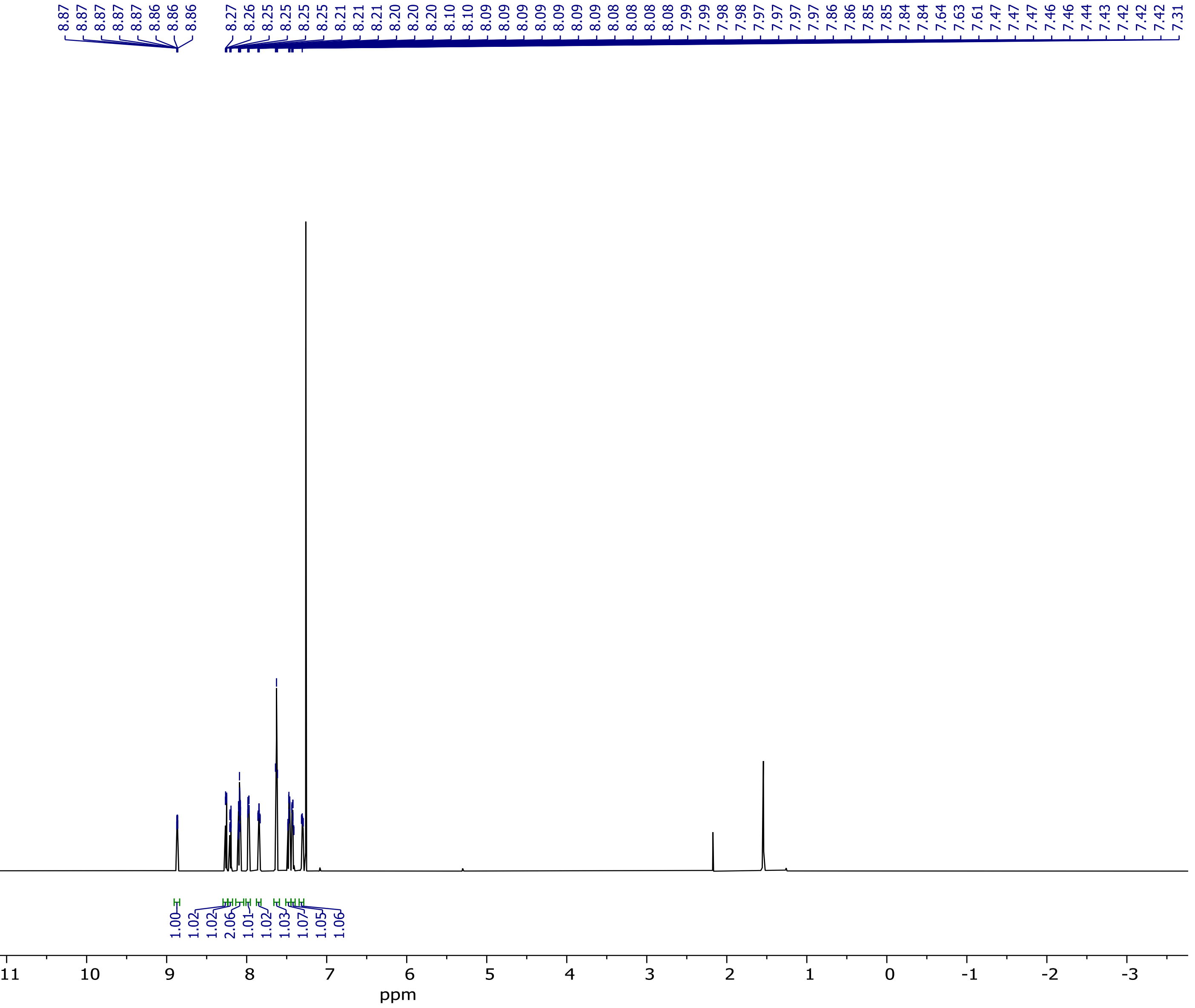

^{13}C NMR of **S3**
151 MHz, $CDCl_3$, 298 K

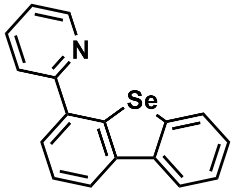

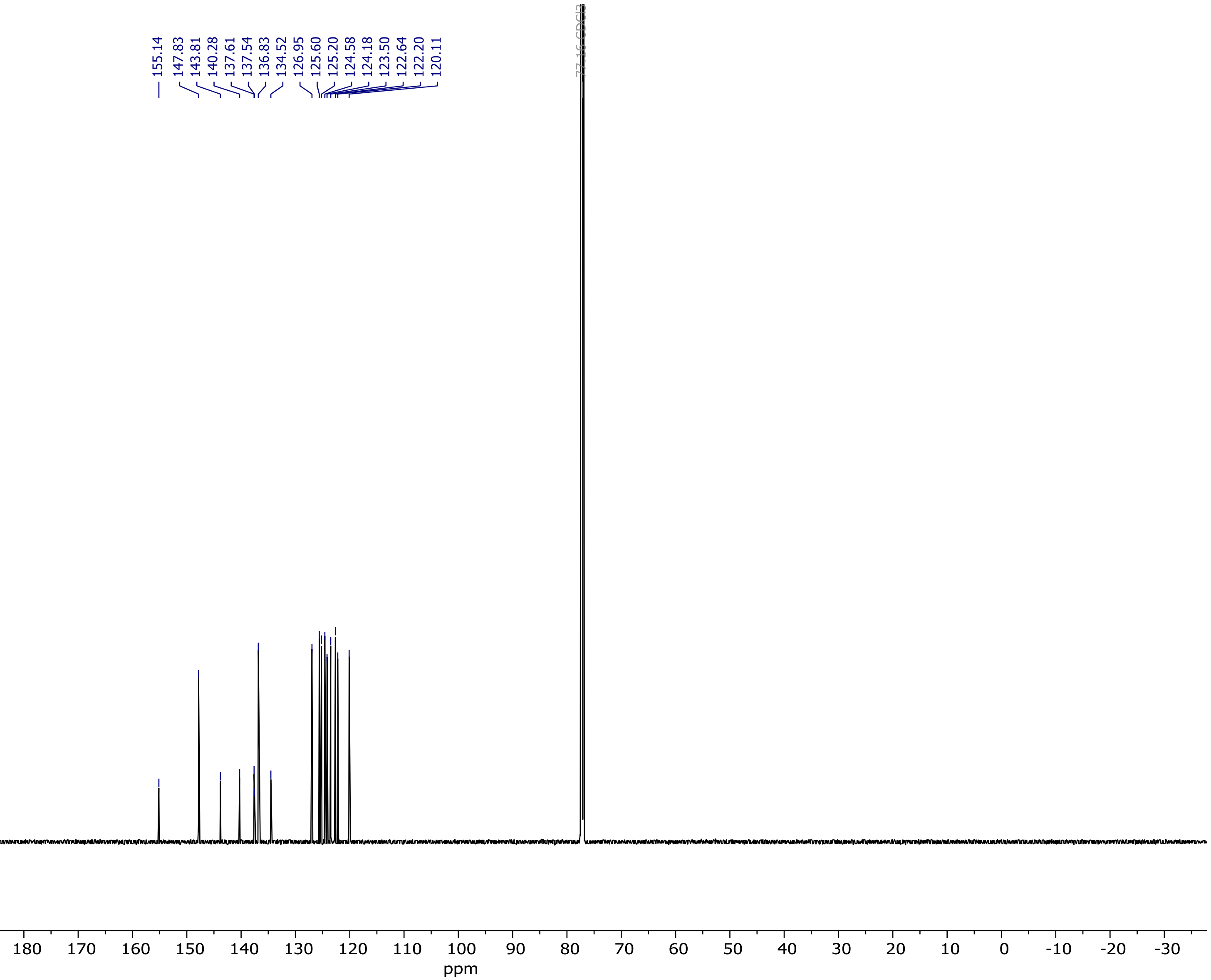

^{77}Se NMR of **S3**
115 MHz, $CDCl_3$, 298 K

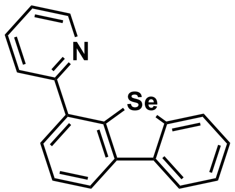

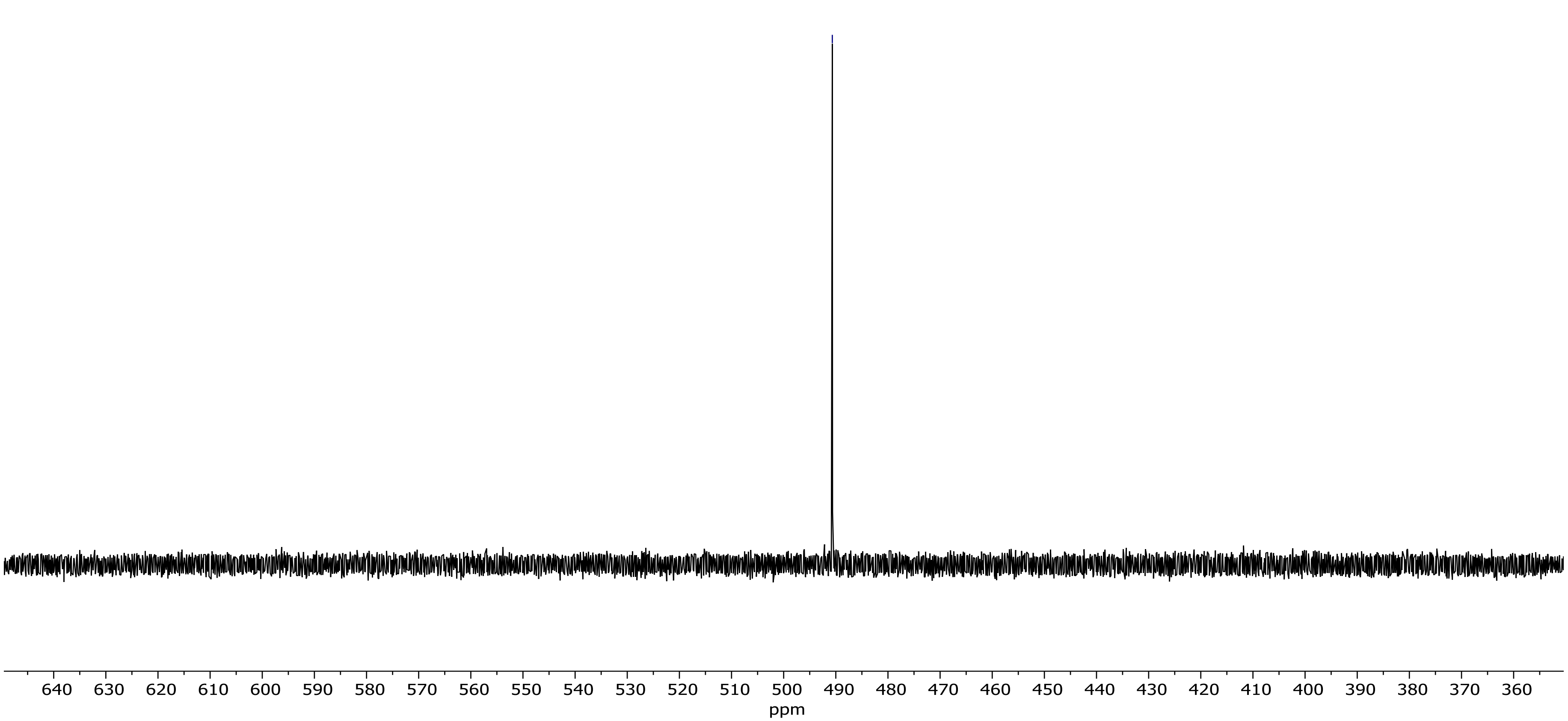

^{1}H NMR of **3**
600 MHz, DMSO, 298 K

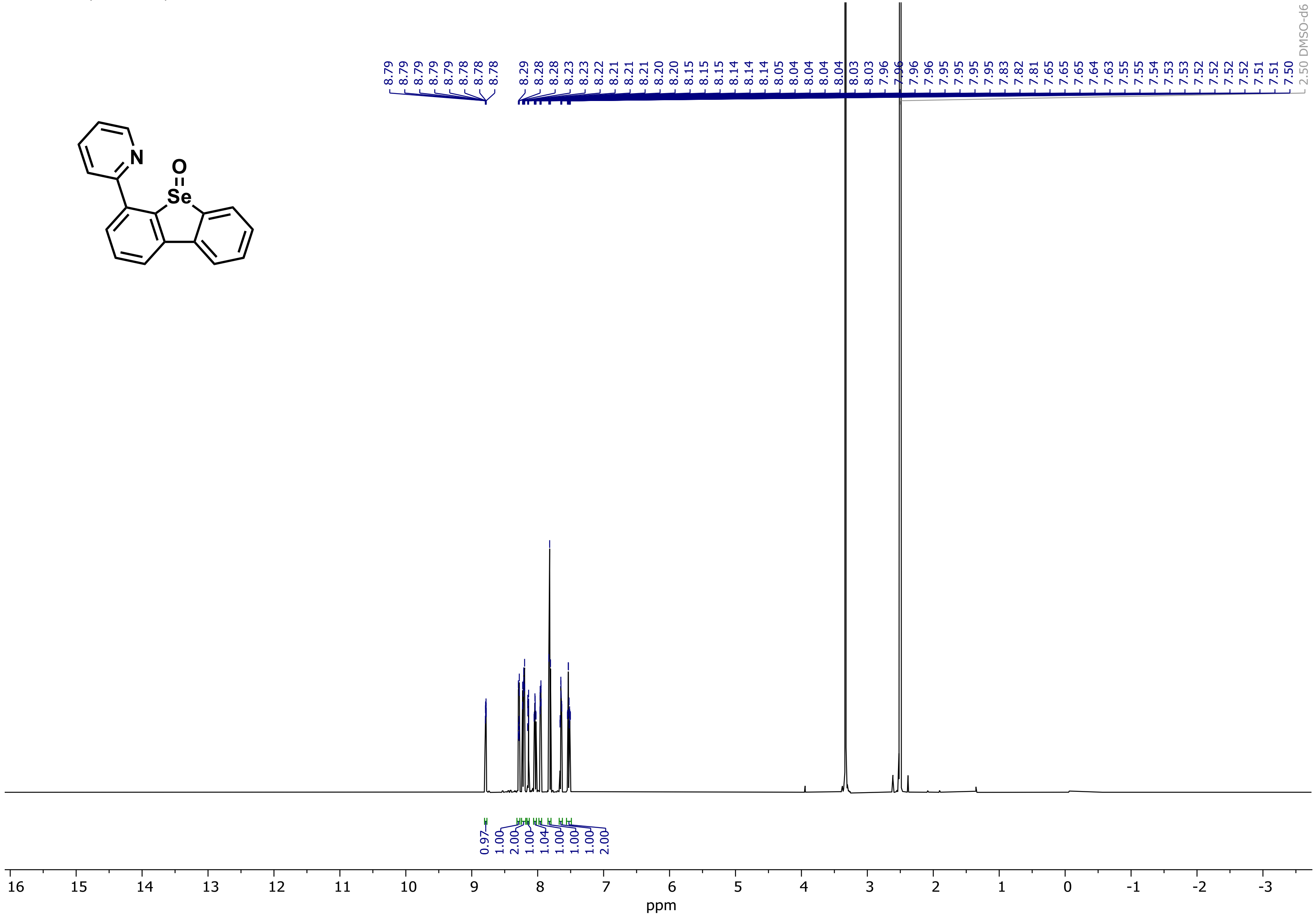

^{13}C NMR of **3**
151 MHz, DMSO, 298 K

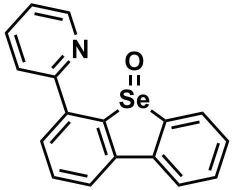

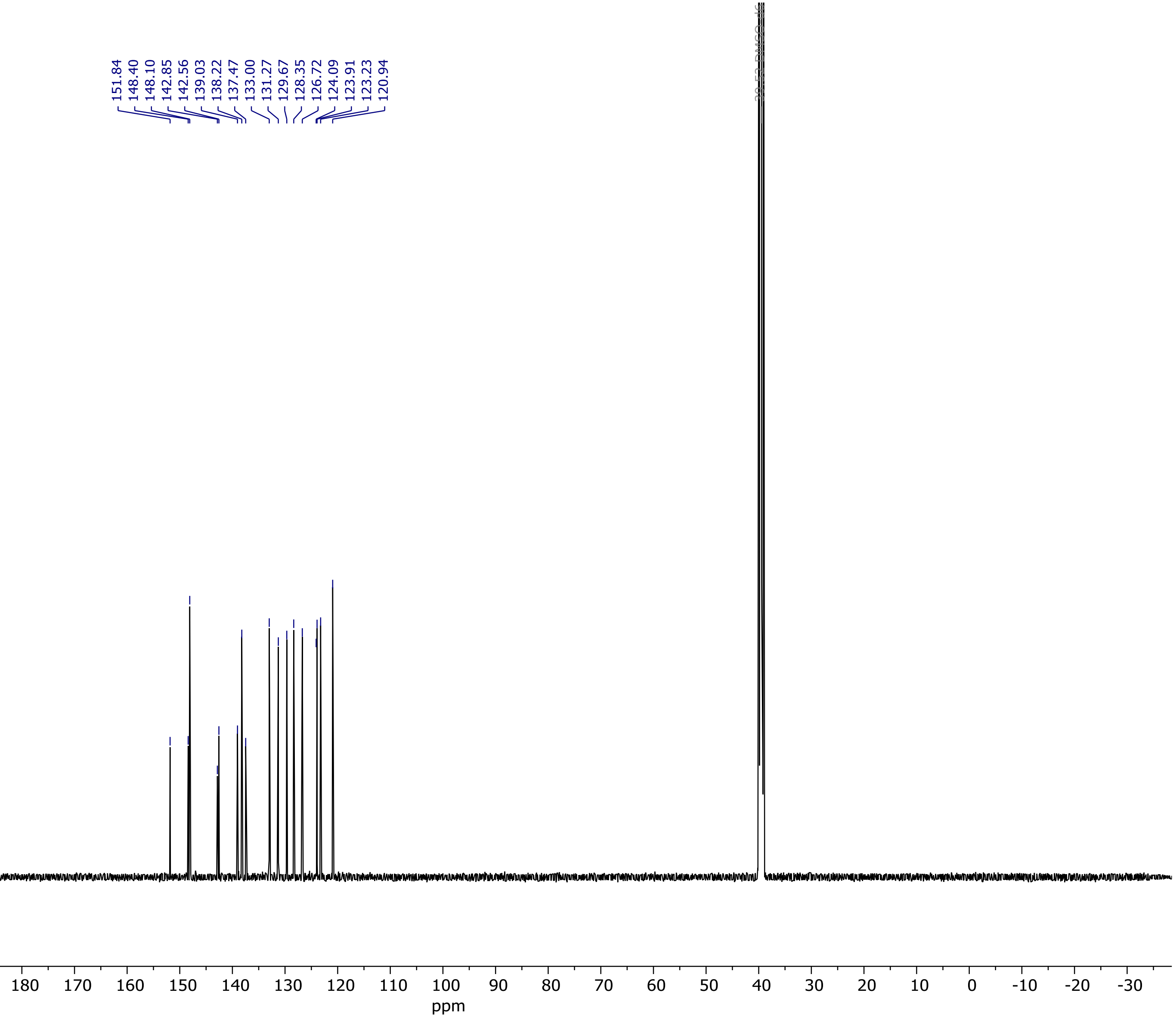

^{77}Se NMR of **3**
115 MHz, DMSO, 298 K

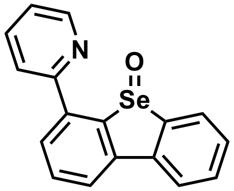

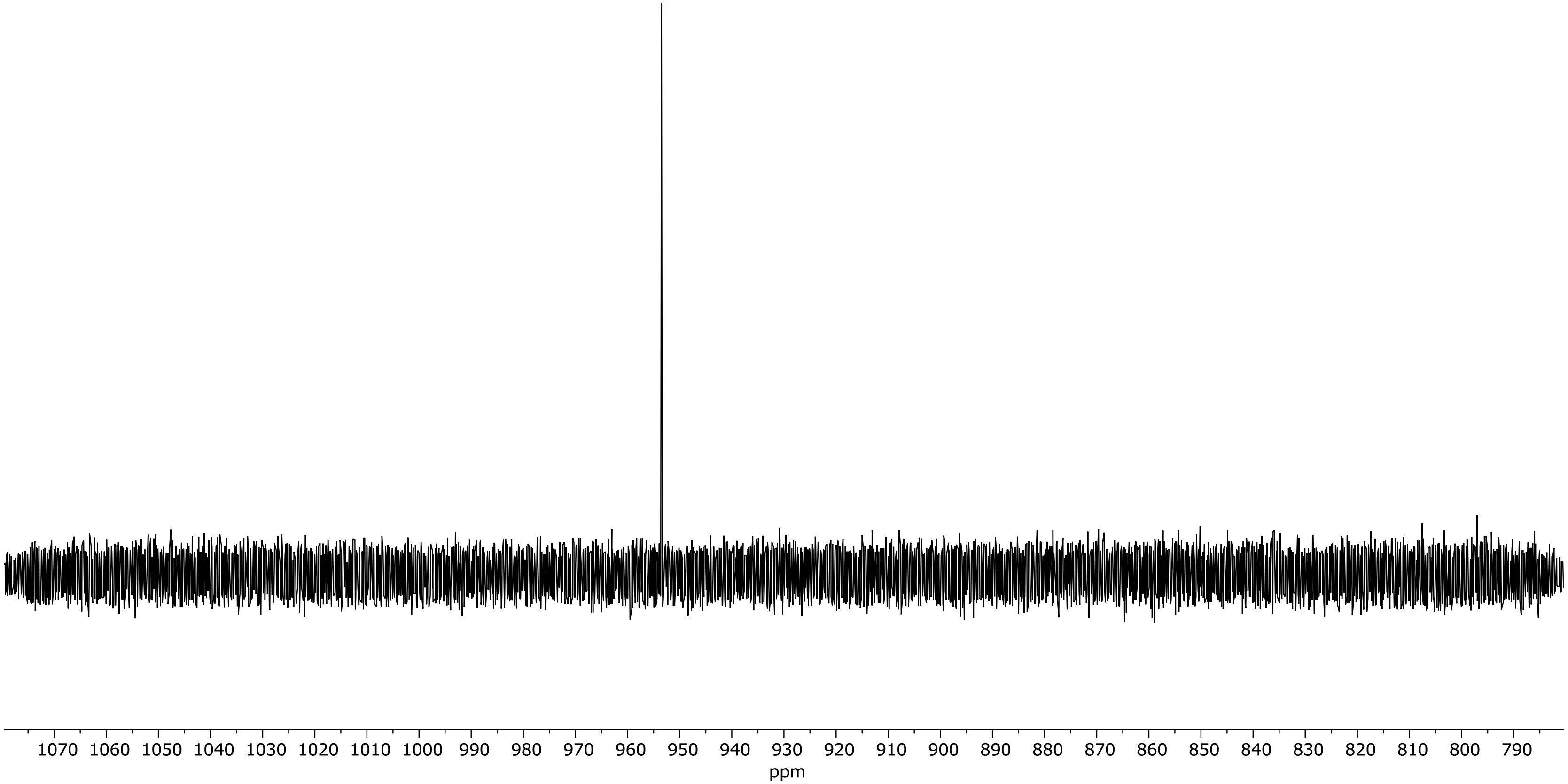

^{1}H NMR of **S61**
600 MHz, DMSO, 298 K

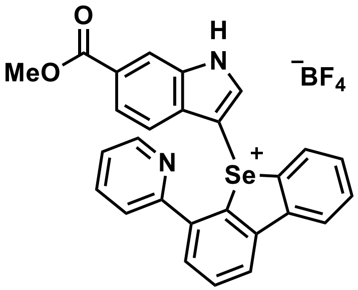

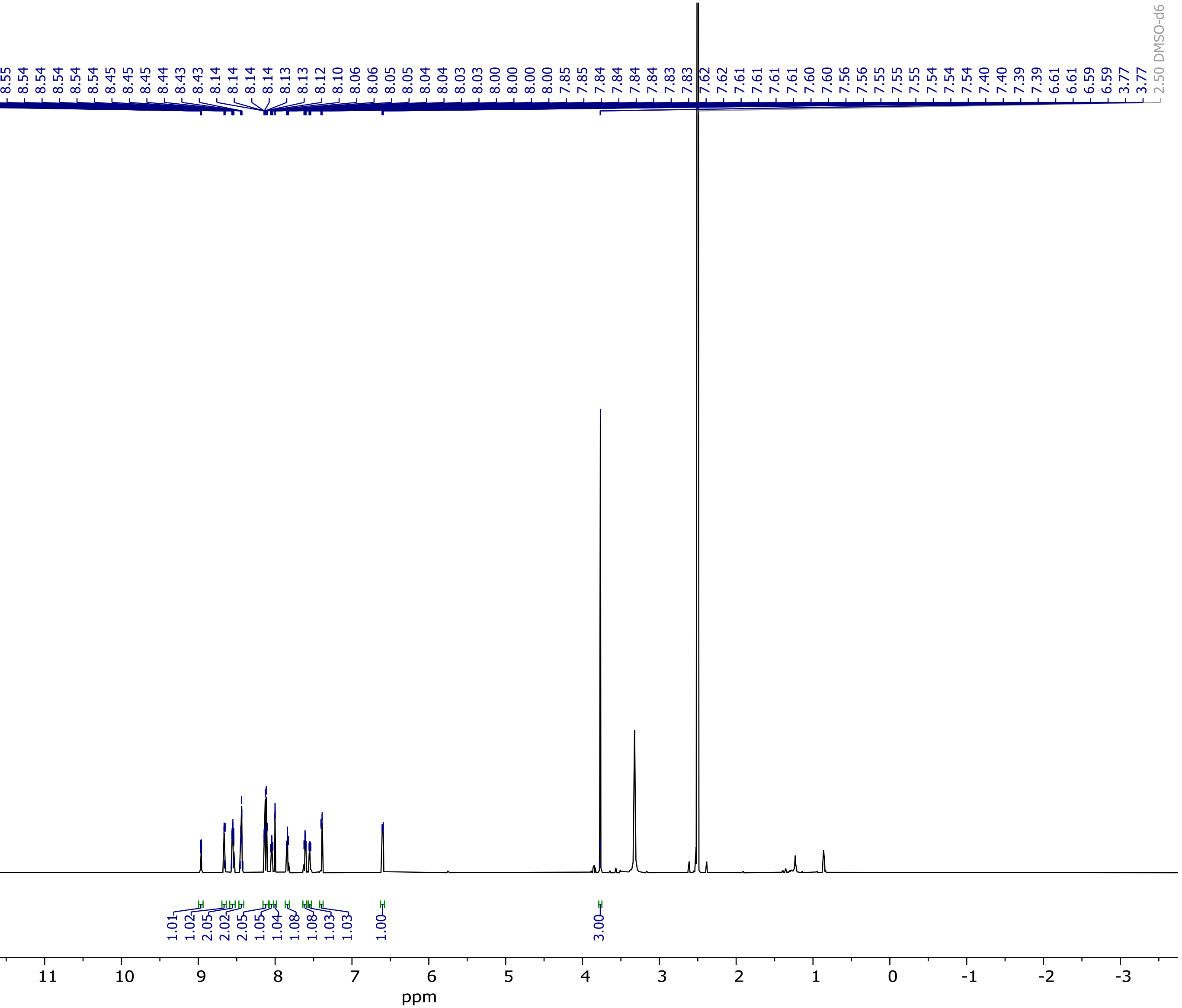

^{13}C NMR of **S61**
151 MHz, DMSO, 298 K

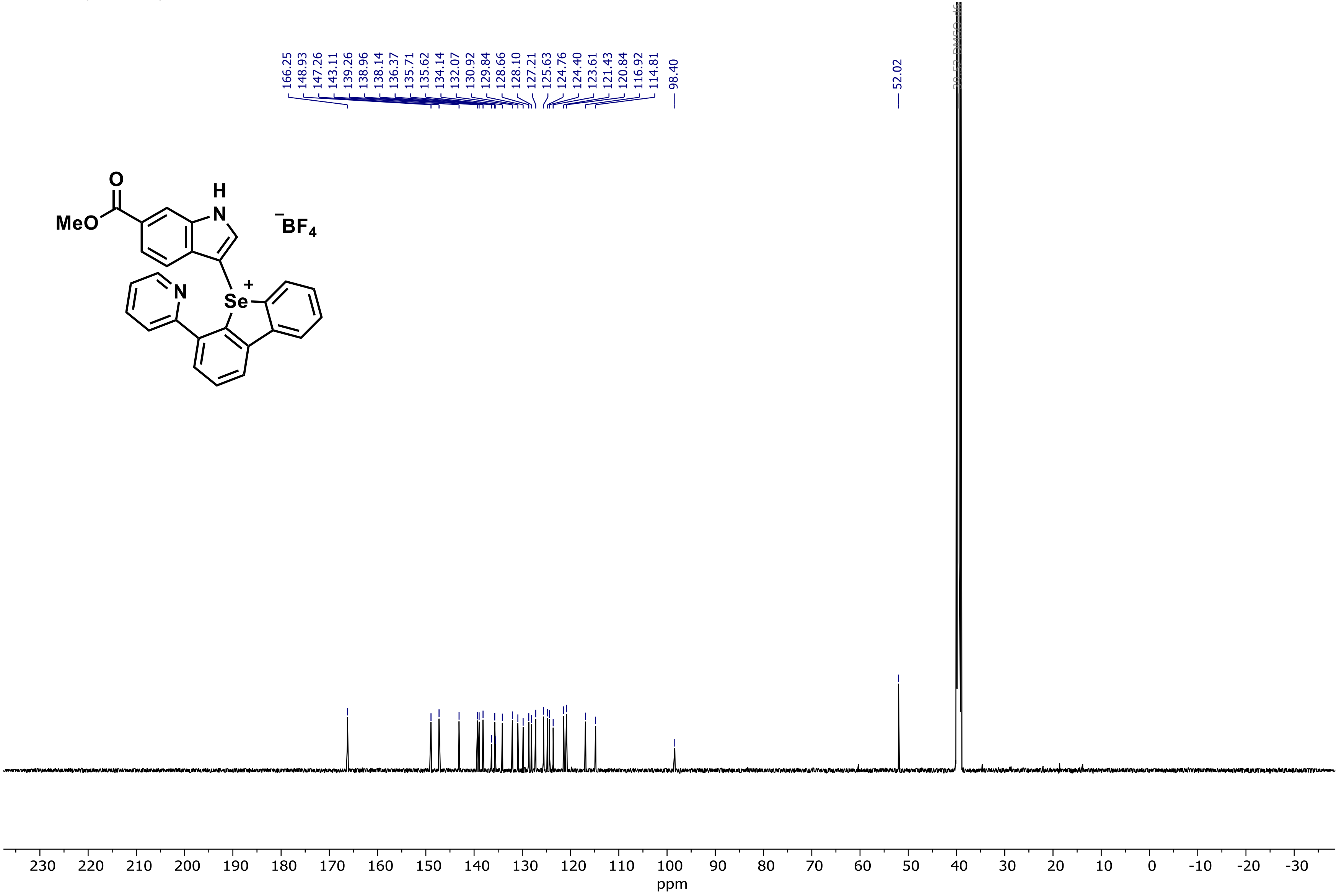

^{19}F NMR of **S61**
565 MHz, DMSO, 298 K

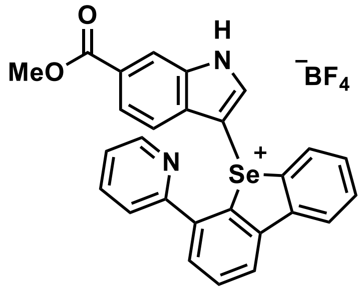

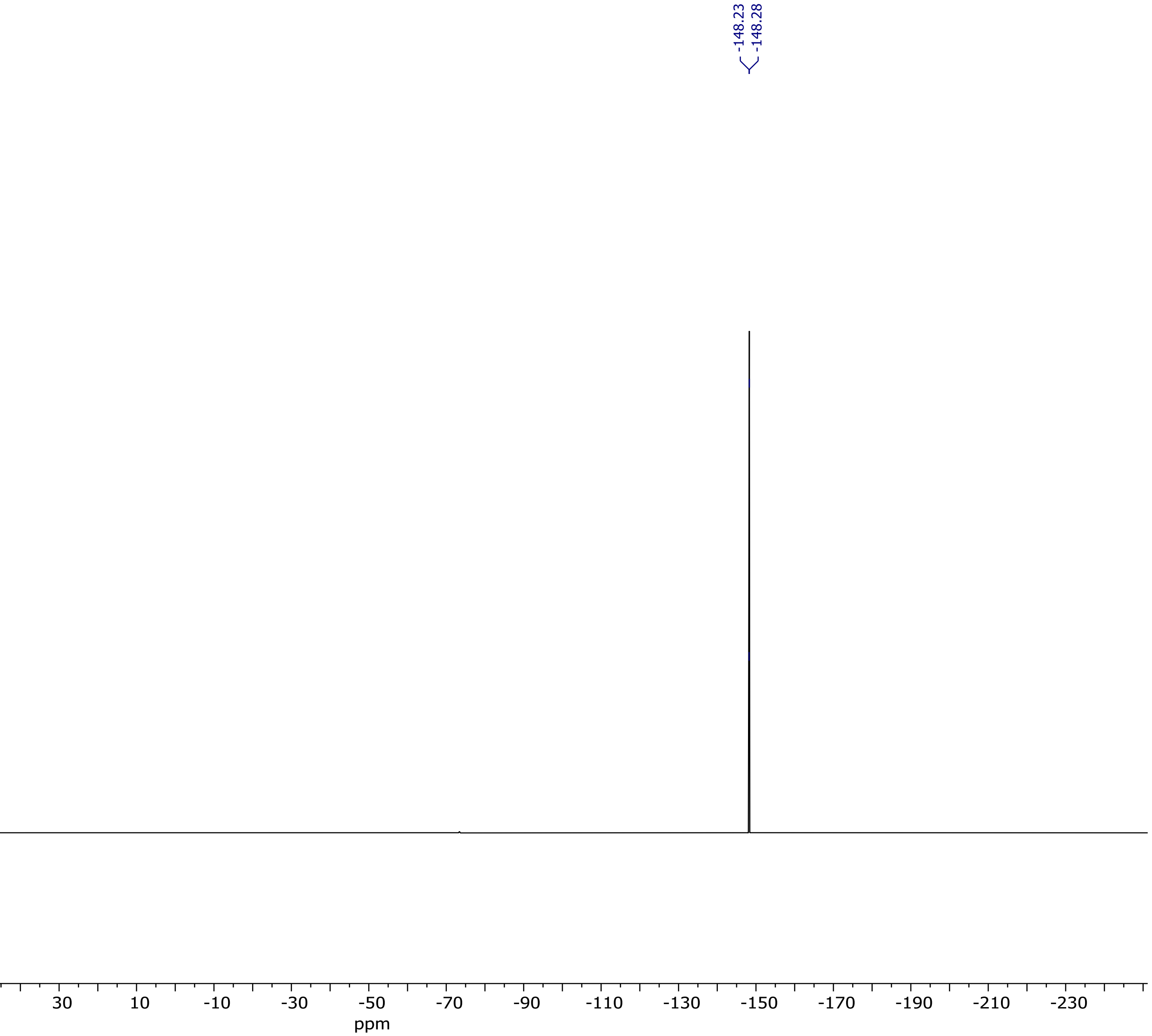

^{77}Se NMR of **S61**
115 MHz, DMSO, 298 K

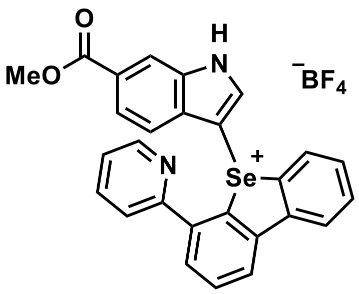

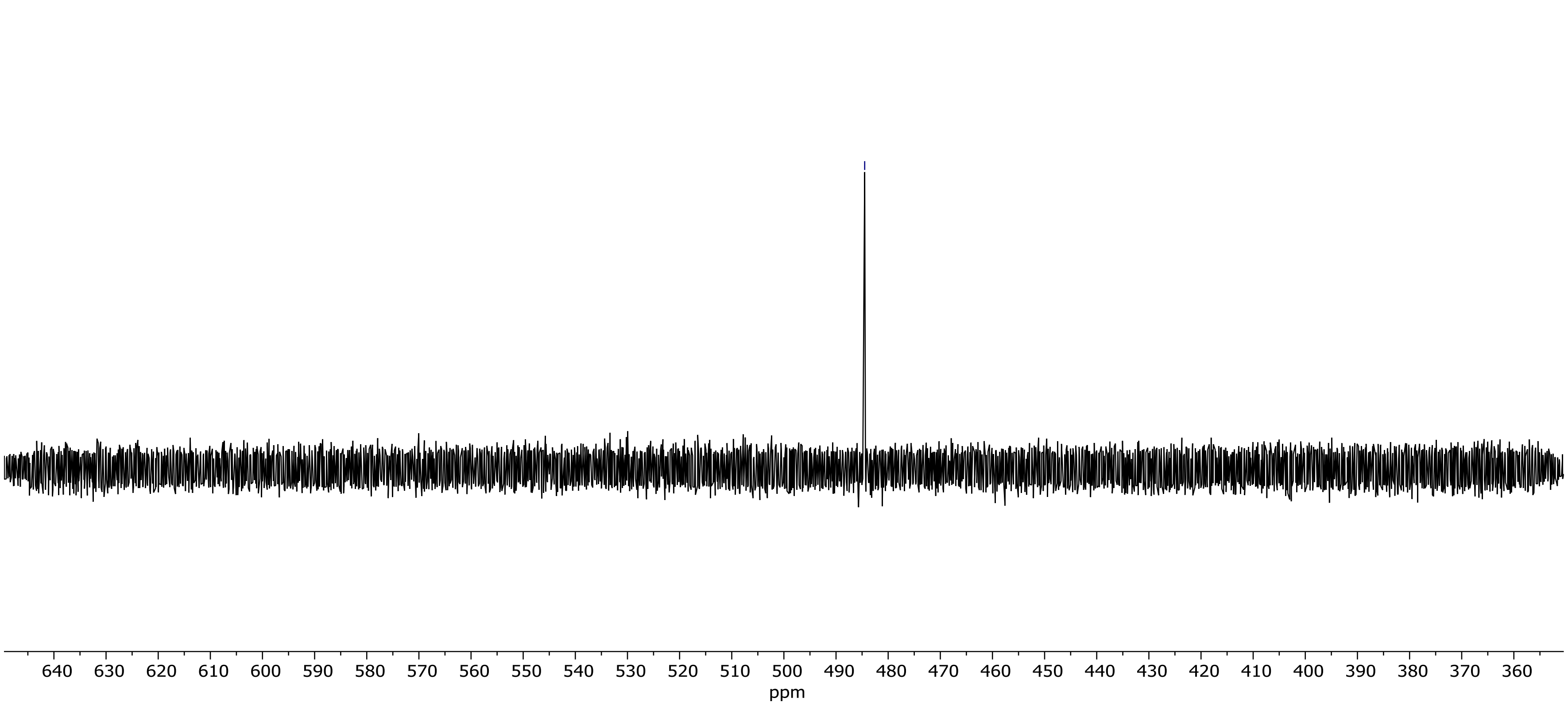

^{1}H NMR of **S62**
600 MHz, DMSO, 298 K

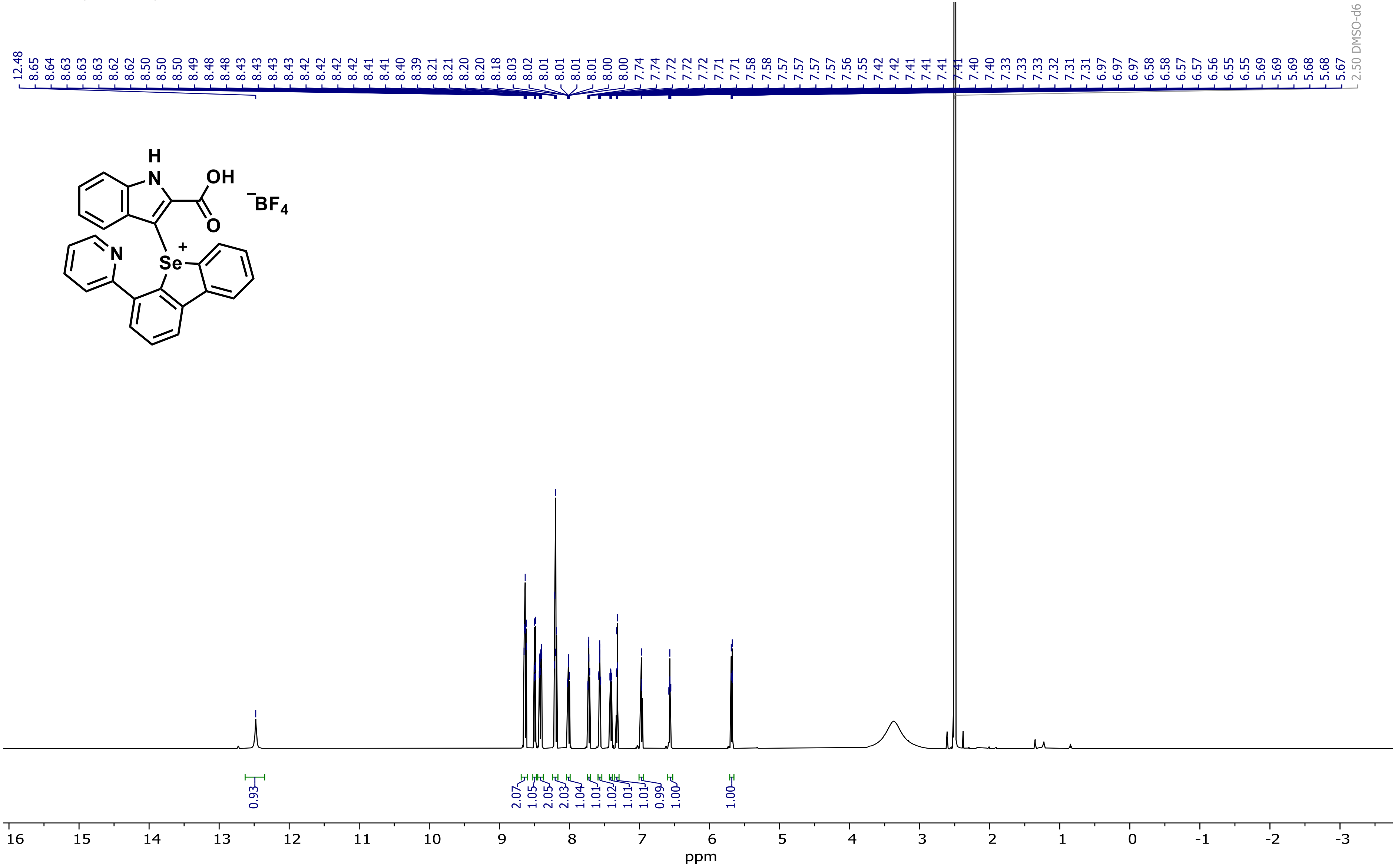

^{13}C NMR of **S62**
151 MHz, DMSO, 298 K

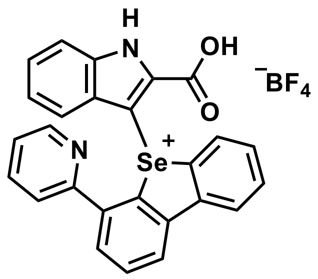

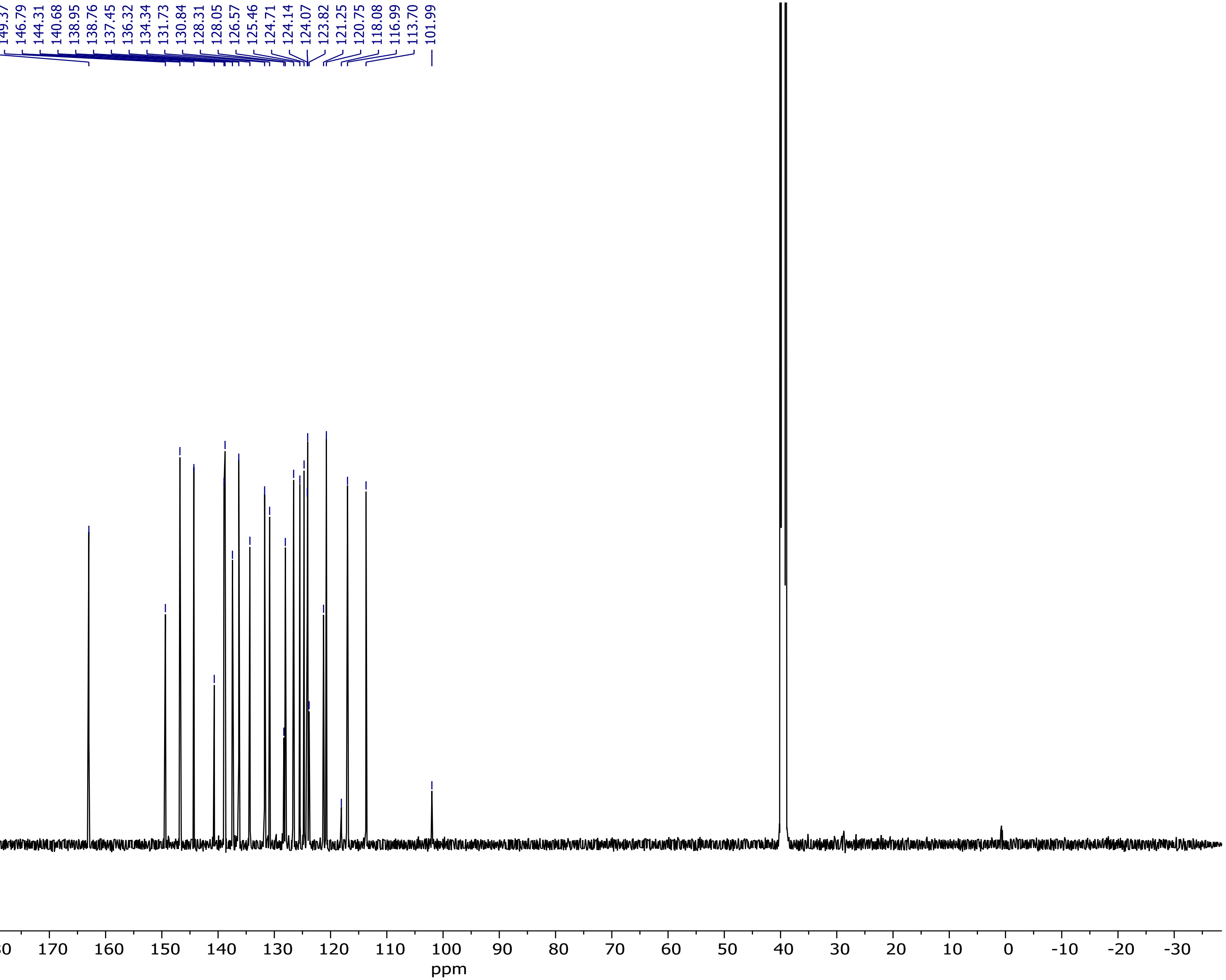

^{19}F NMR of **S62**
565 MHz, DMSO, 298 K

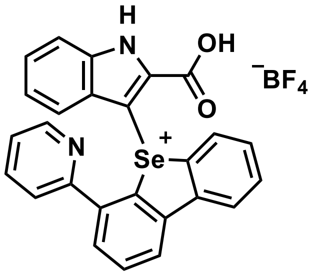

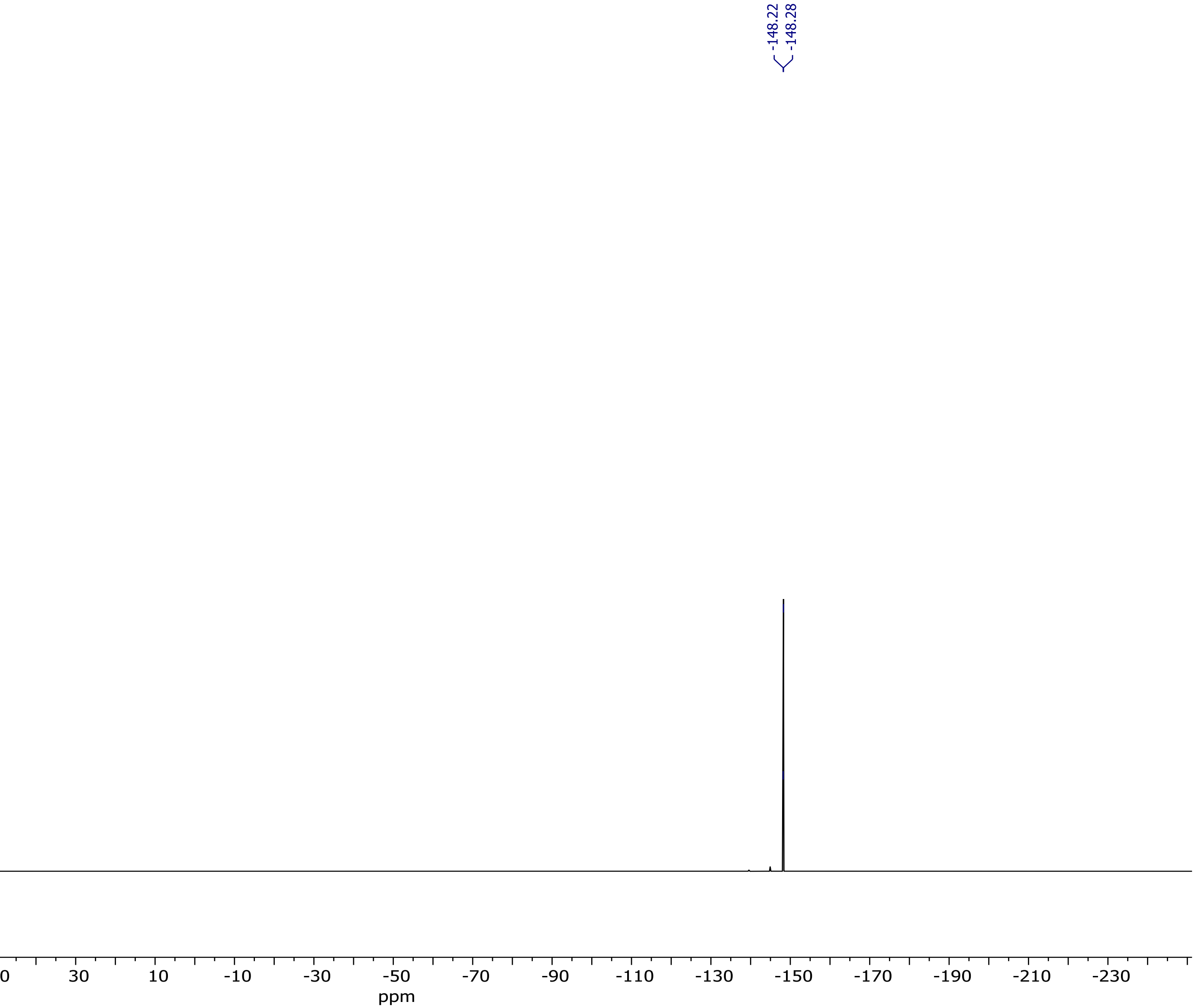

^{77}Se NMR of **S62**
115 MHz, DMSO, 298 K

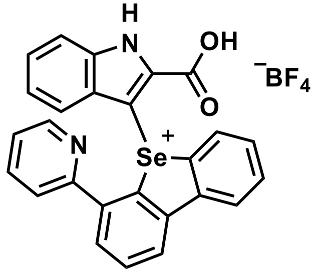

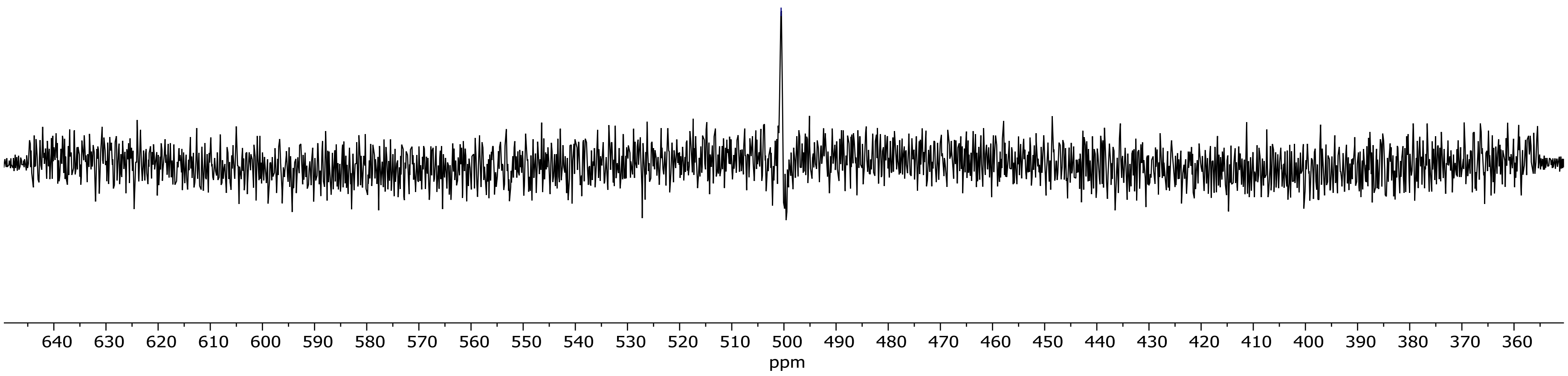

^{1}H NMR of **S63**
600 MHz, CD_3OD, 298 K

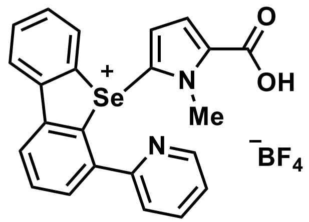

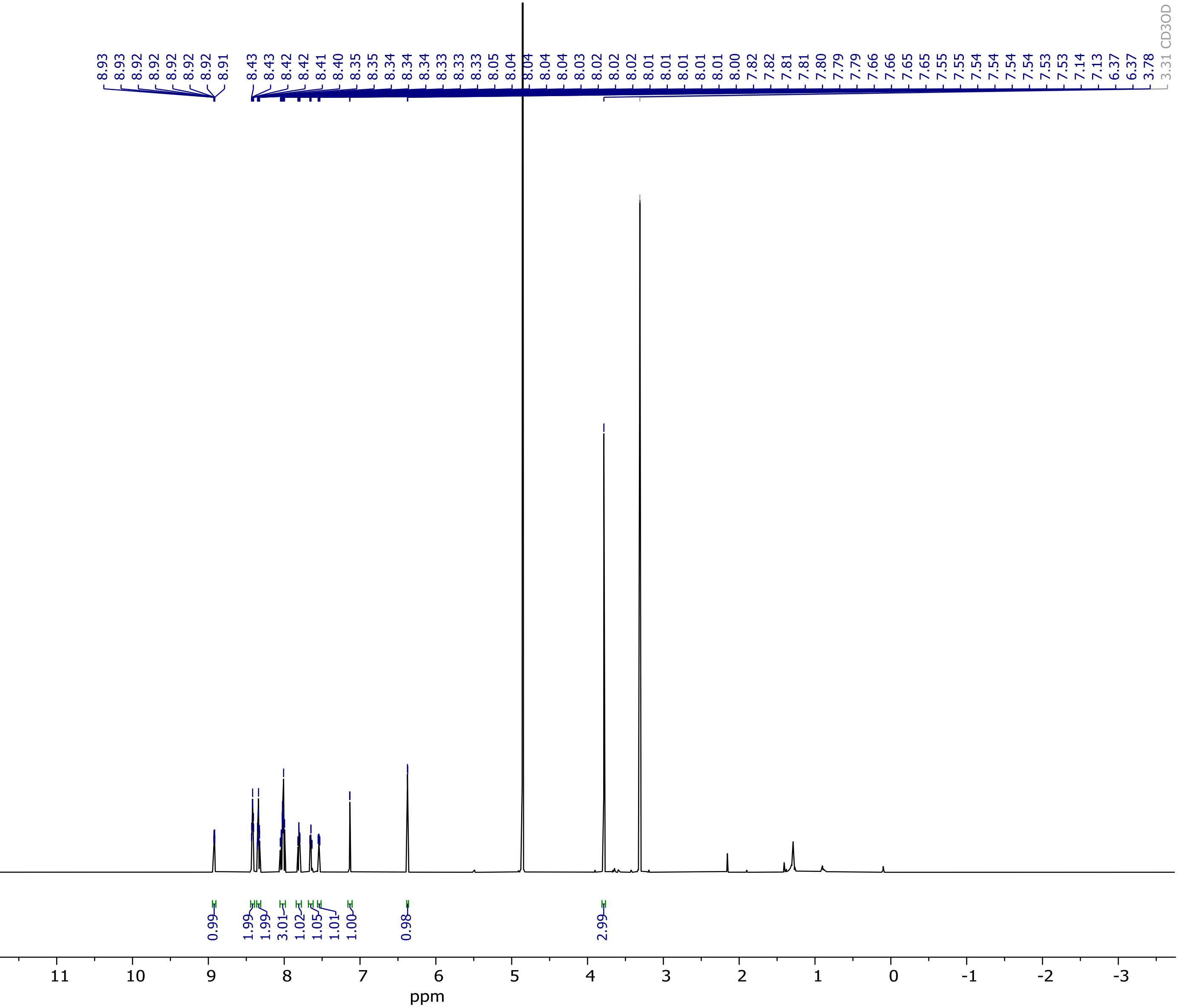

^{13}C NMR of **S63**
151 MHz, CD_3OD, 298 K

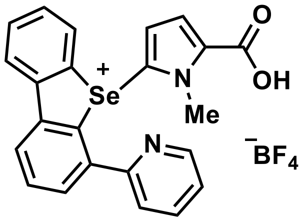

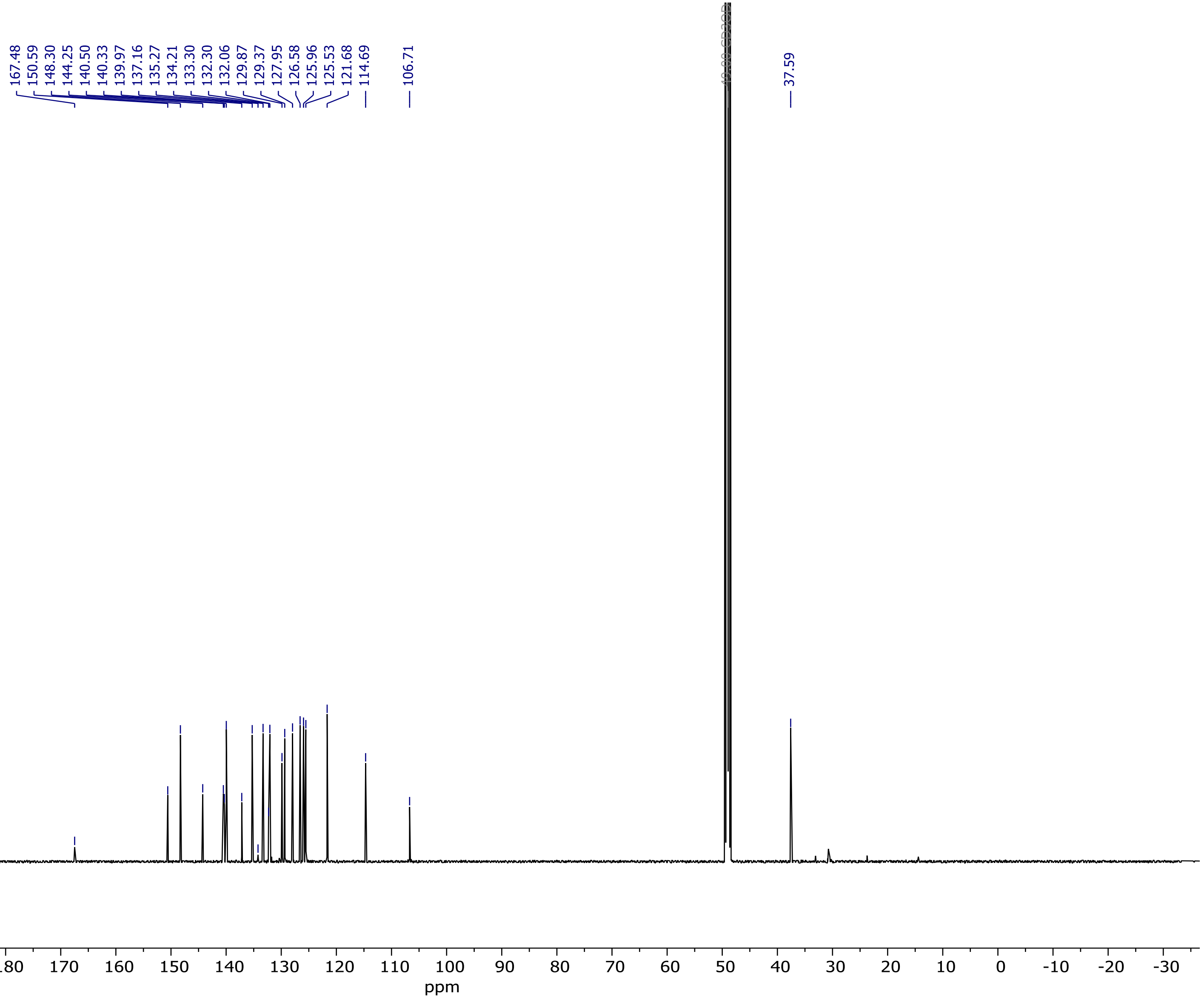

^{19}F NMR of **S63**
565 MHz, CD_3OD, 298 K

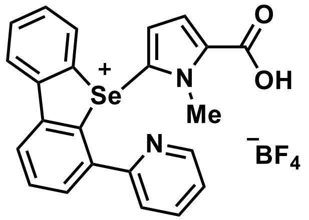

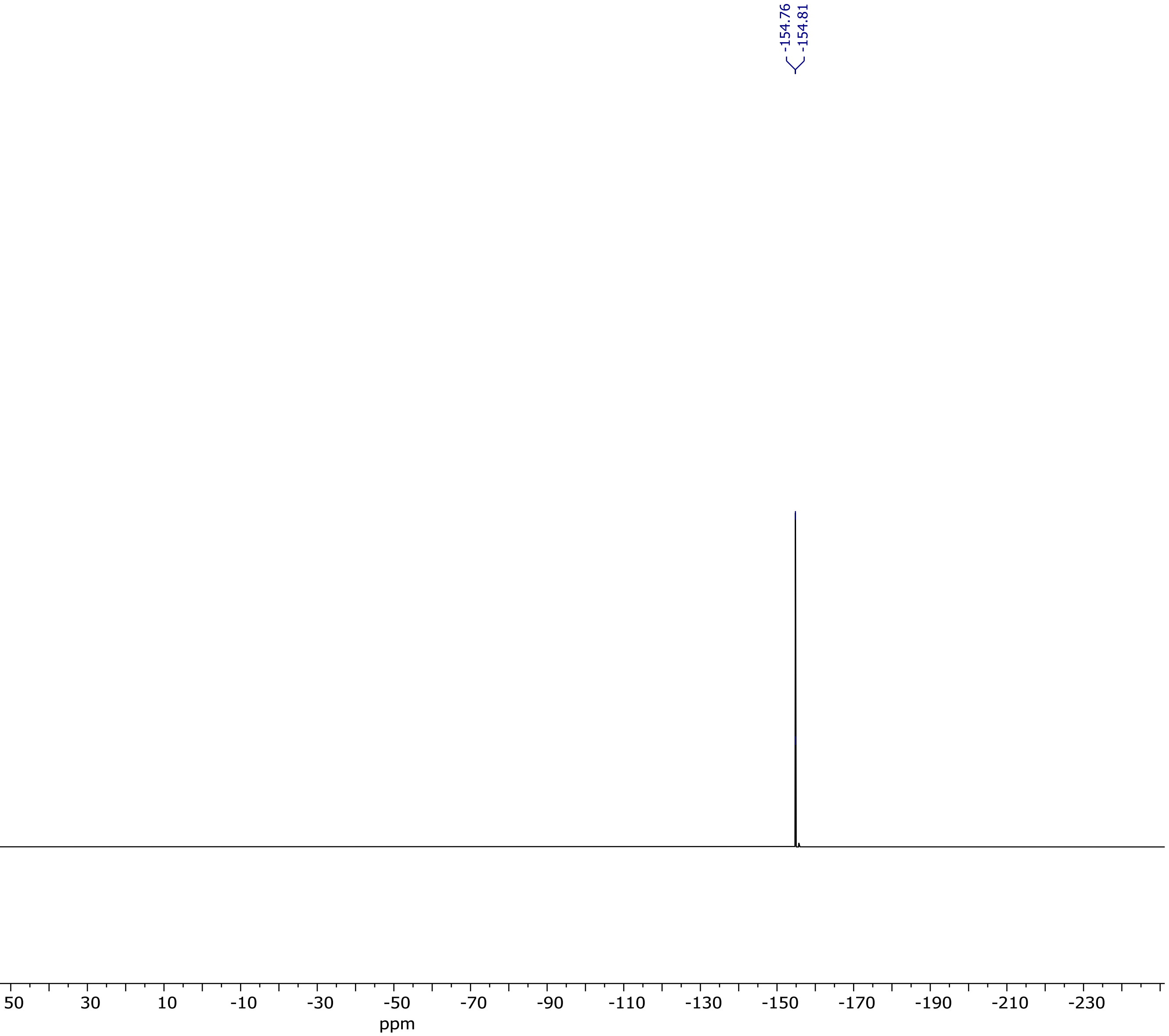

^{77}Se NMR of **S63**
115 MHz, CD_3OD, 298 K

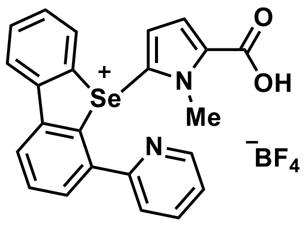

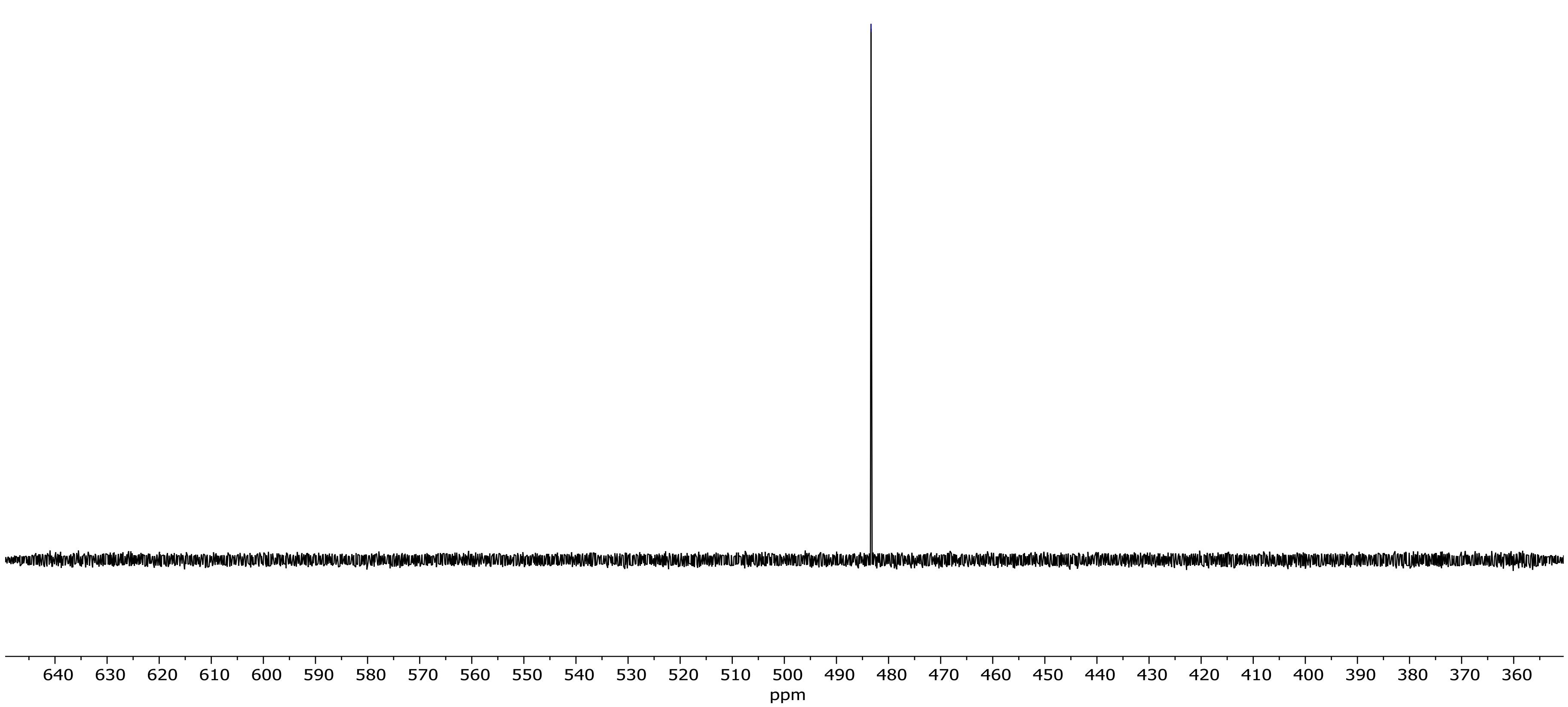

^{1}H NMR of **S64**
600 MHz, CD_3CN, 298 K

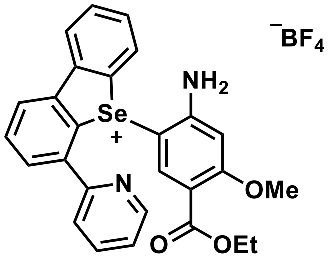

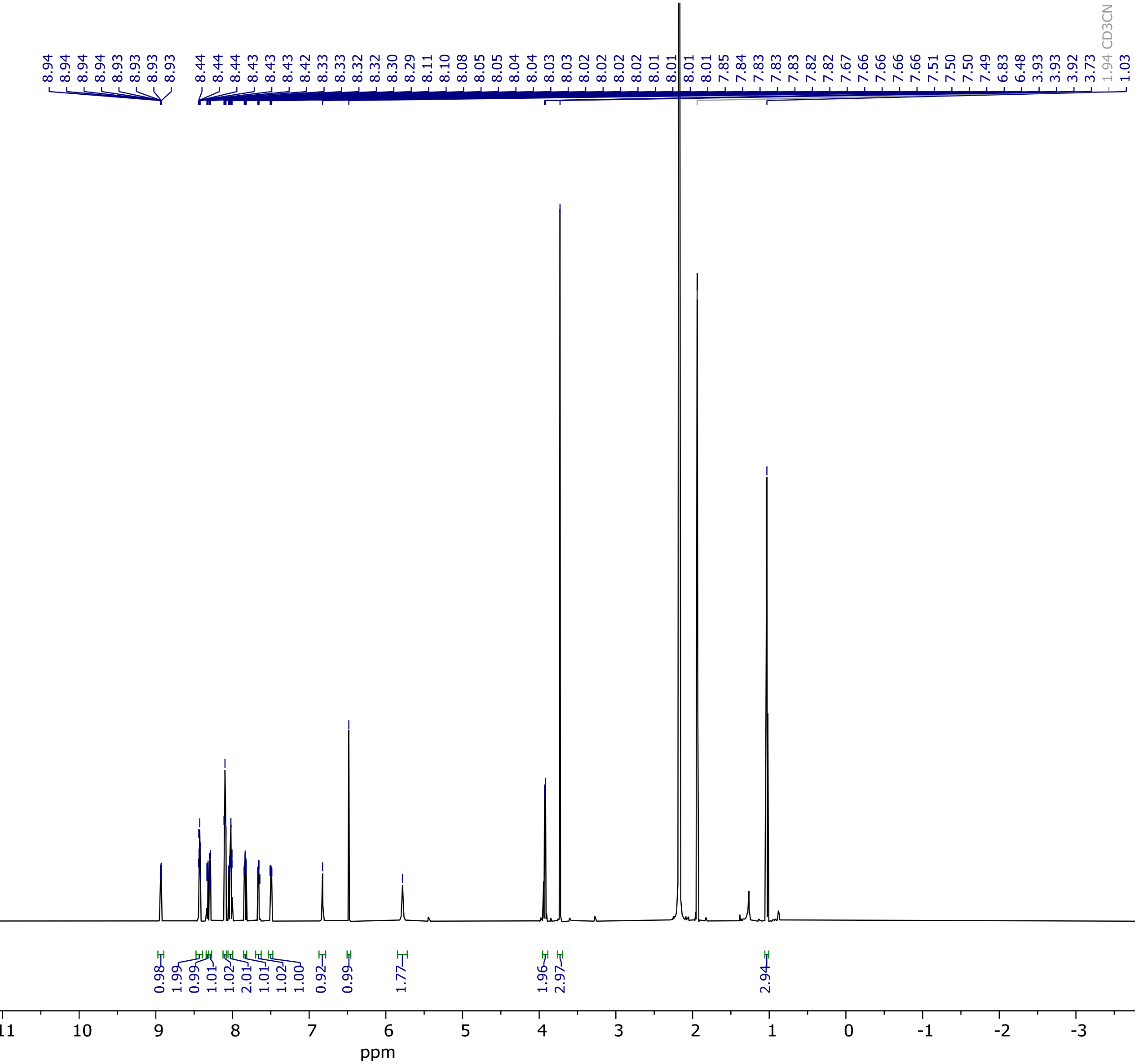

^{13}C NMR of **S64**
151 MHz, CD_3CN, 298 K

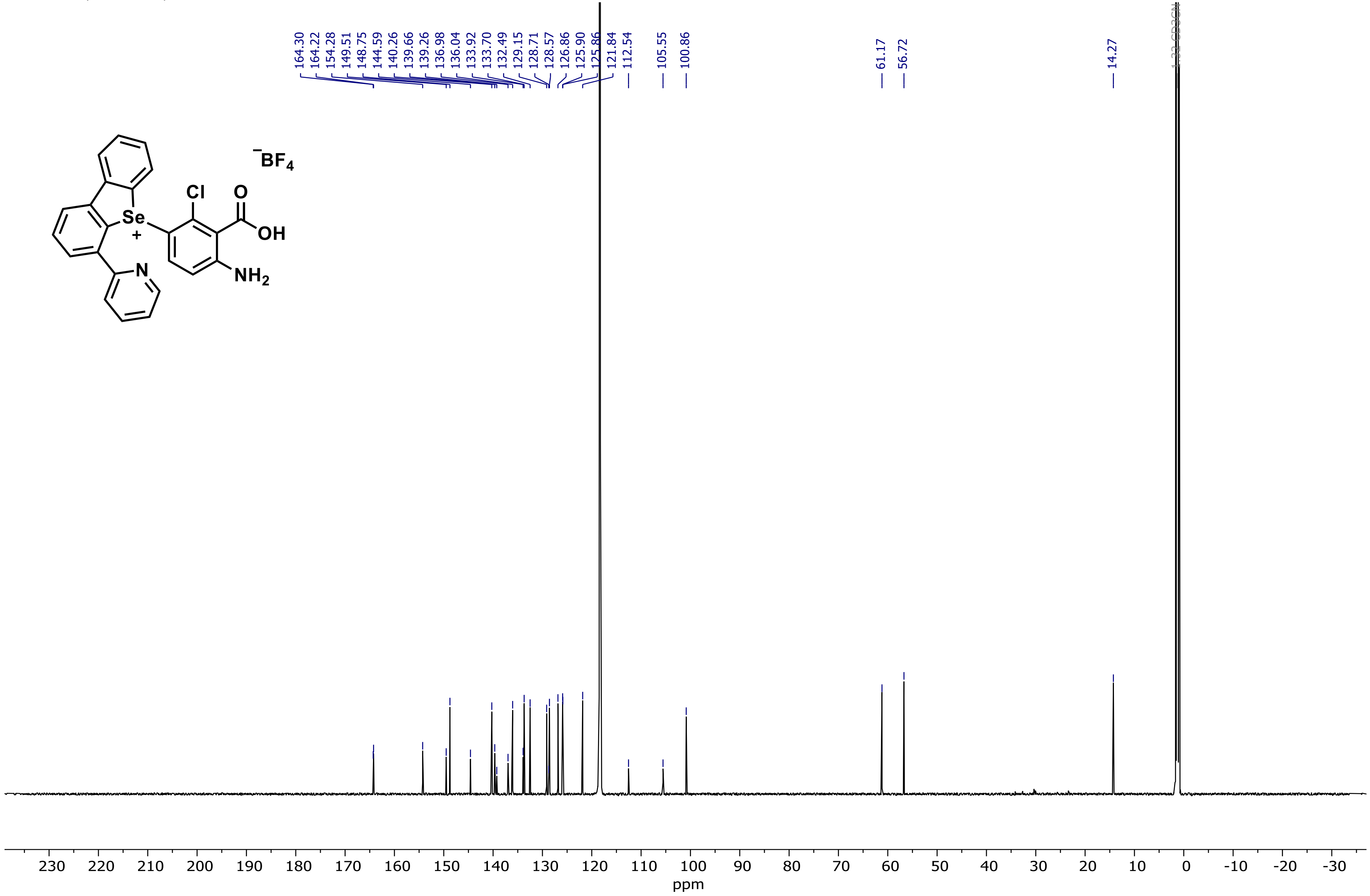

^{19}F NMR of **S64**
565 MHz, CD_3CN, 298 K

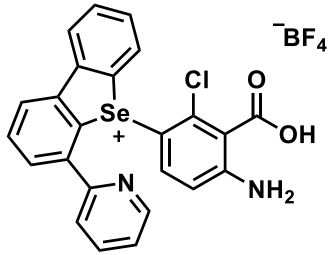

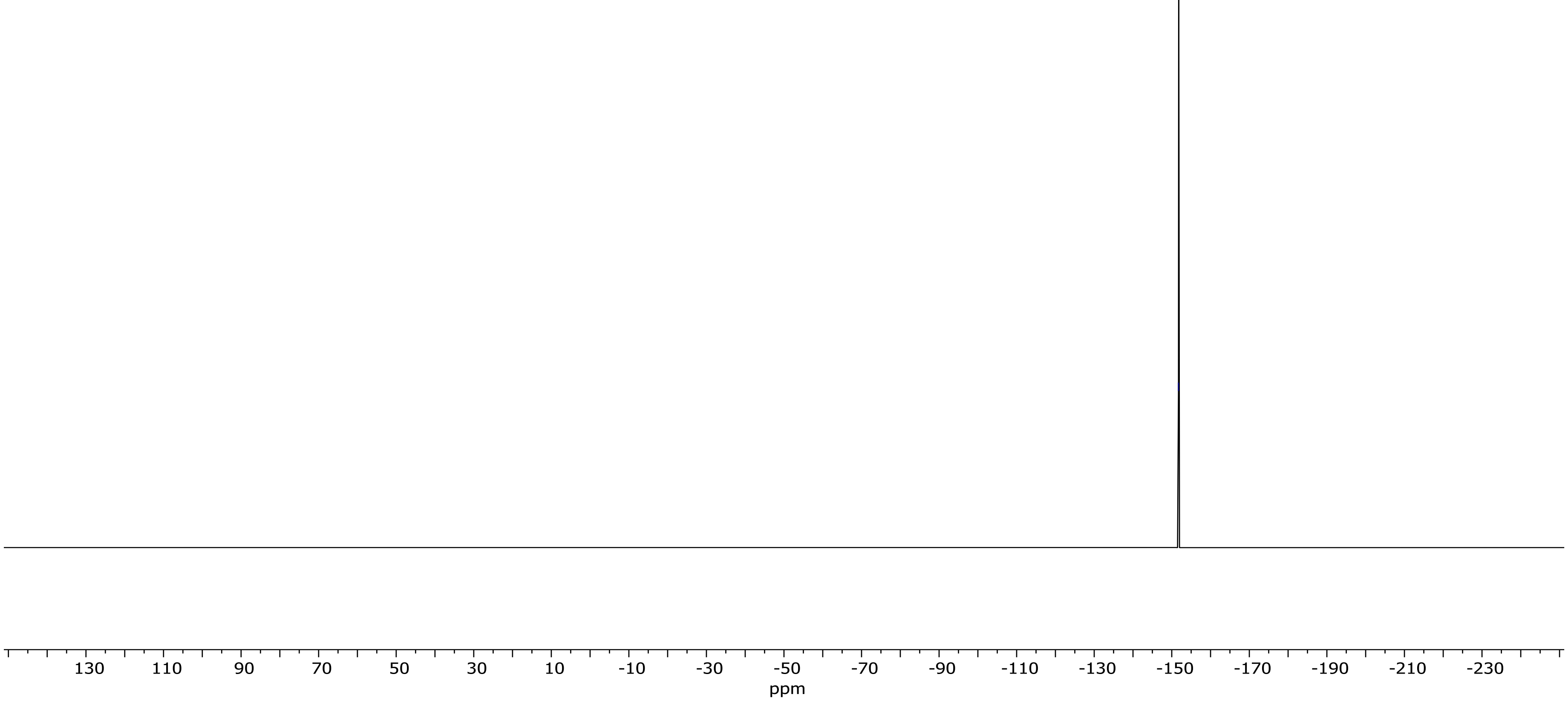

^{77}Se NMR of **S64**
115 MHz, CD_3CN, 298 K

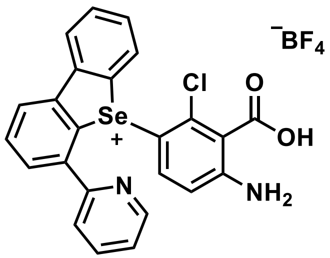

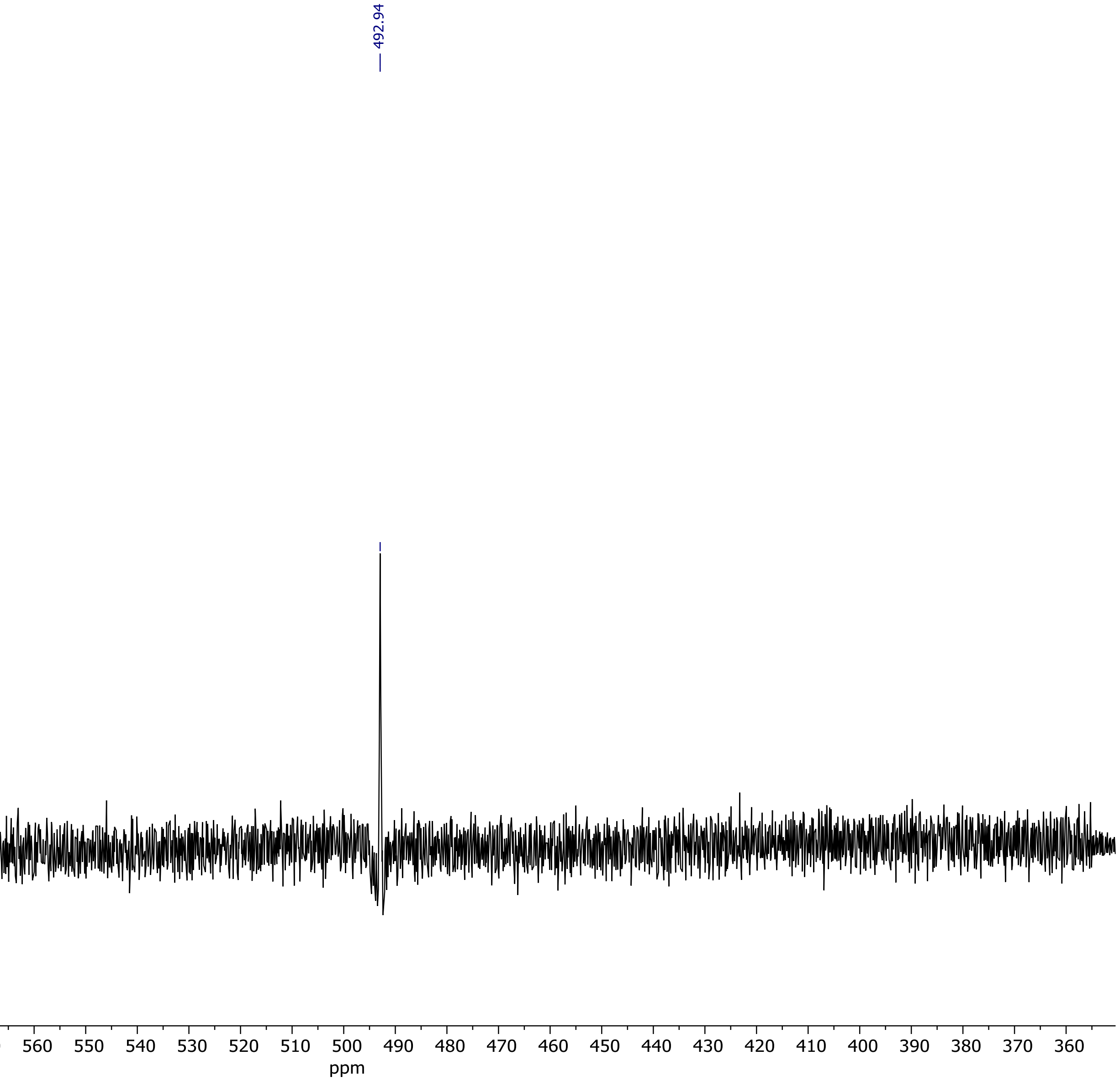

^{1}H NMR of **S65**
600 MHz, DMSO, 298 K

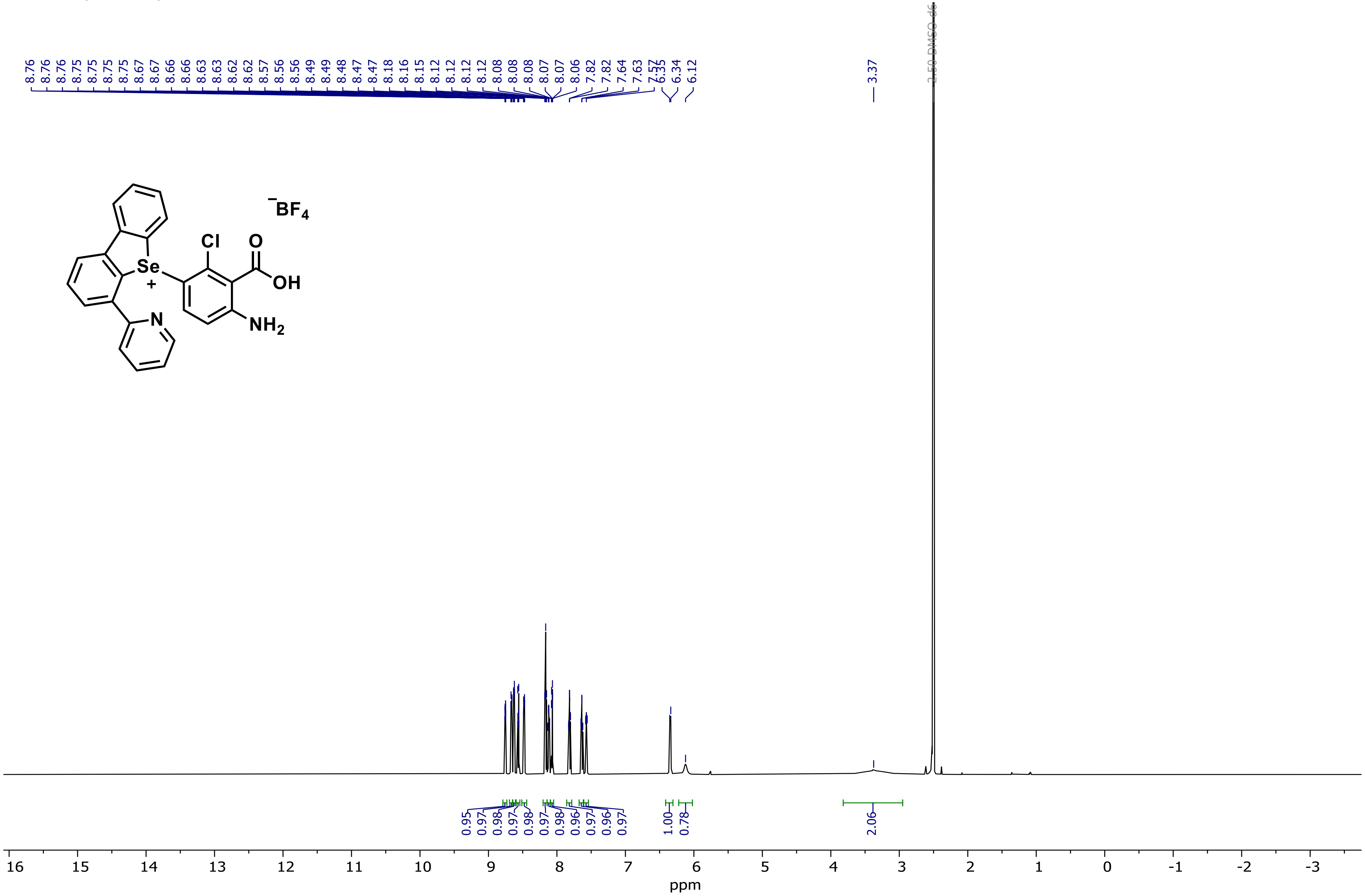

^{13}C NMR of **S65**
151 MHz, DMSO, 298 K

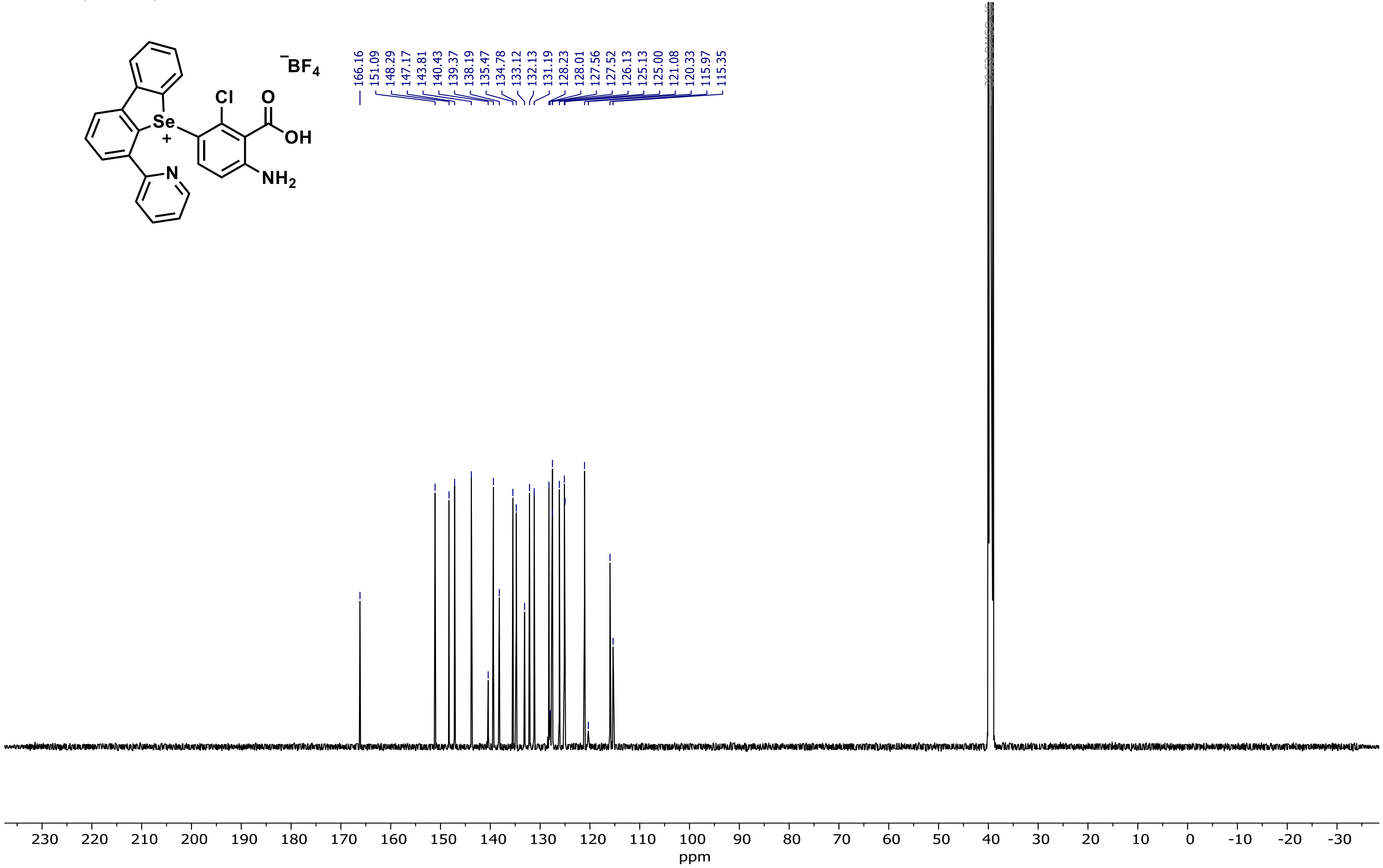

^{19}F NMR of **S65**
565 MHz, DMSO, 298 K

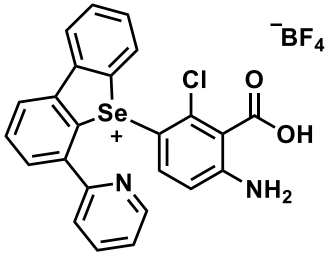

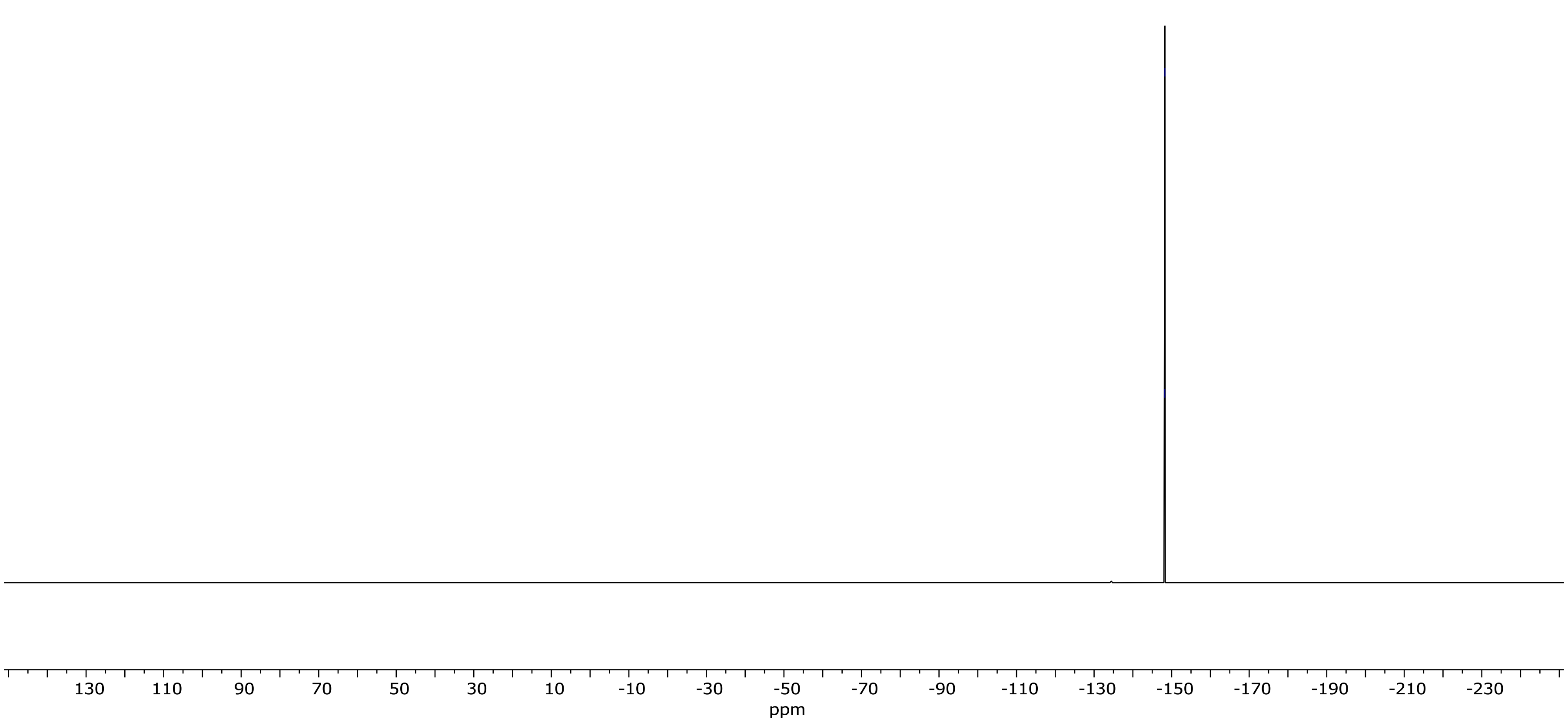

^{77}Se NMR of **S65**
115 MHz, DMSO, 298 K

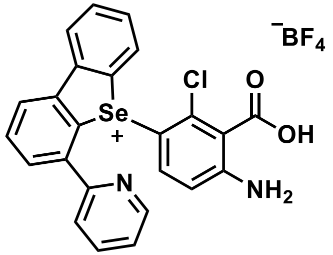

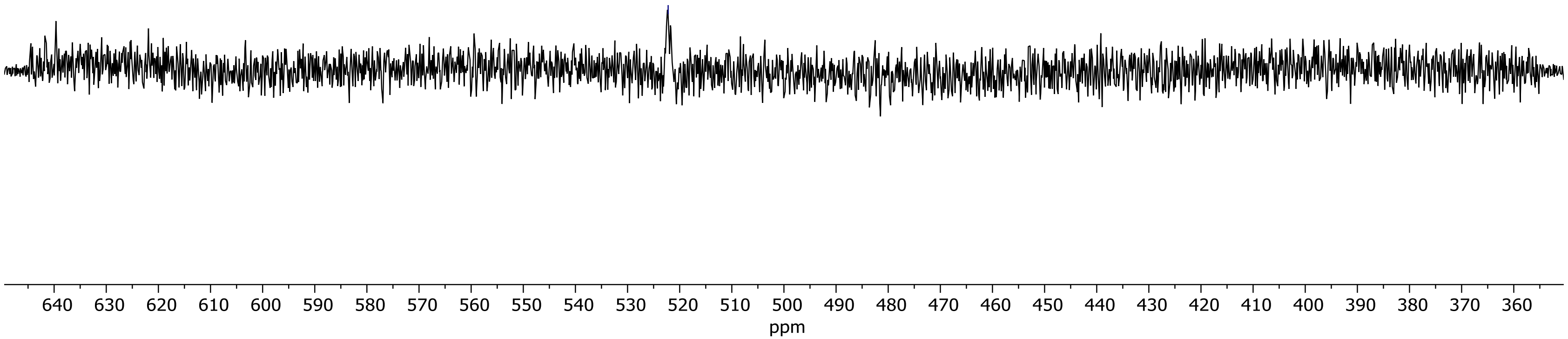

^{1}H NMR of **S66**
600 MHz, DMSO, 298 K

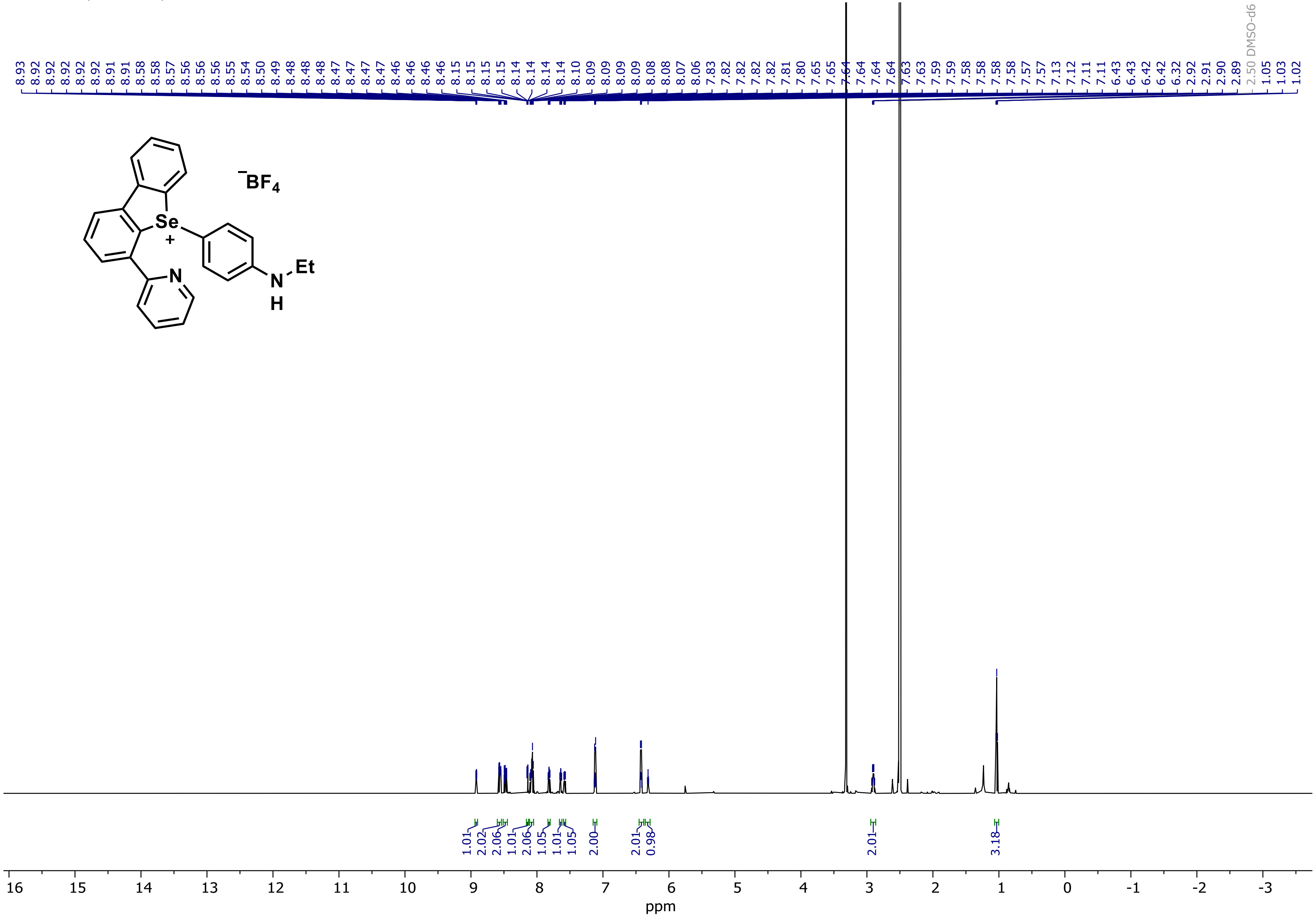

^{13}C NMR of **S66**
151 MHz, DMSO, 298 K

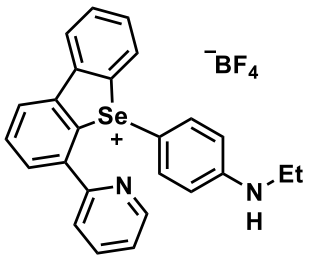

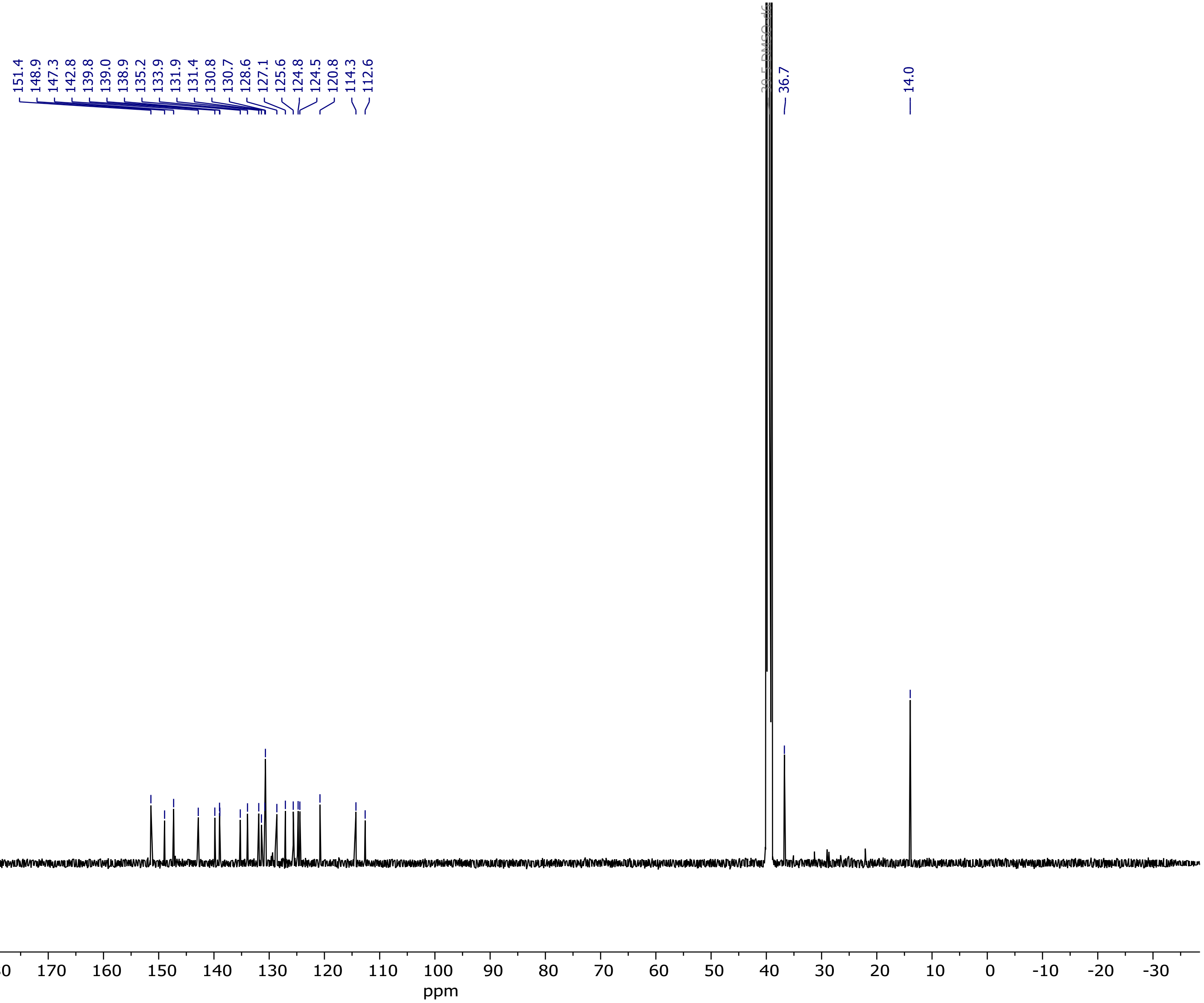

^{19}F NMR of **S66**
565 MHz, DMSO, 298 K

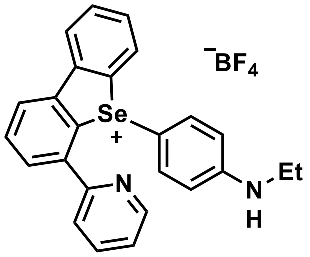

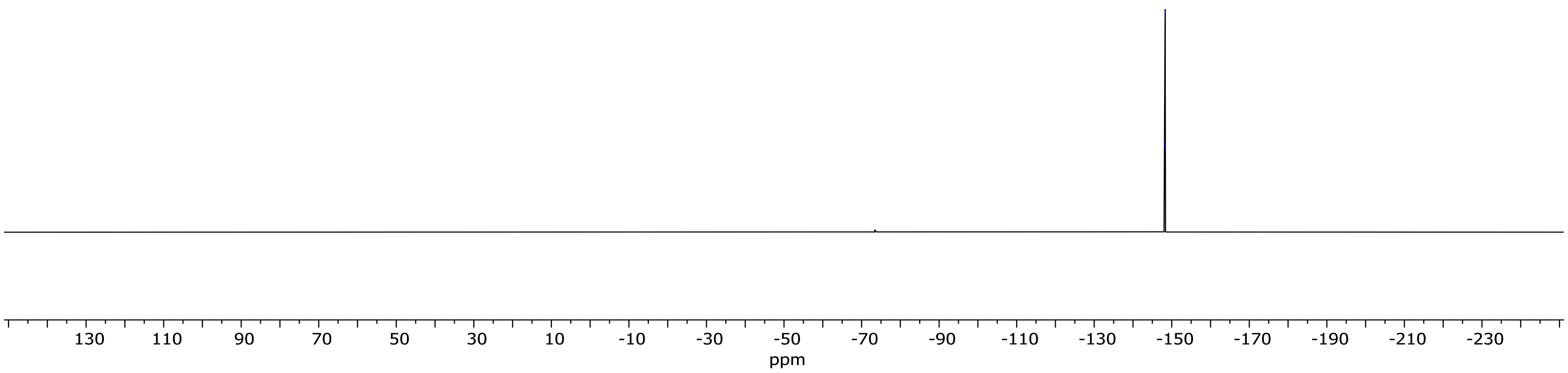

^{77}Se NMR of **S66**
115 MHz, DMSO, 298 K

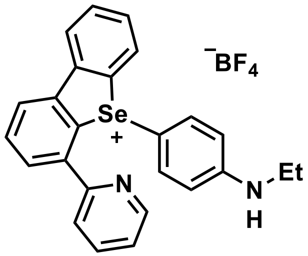

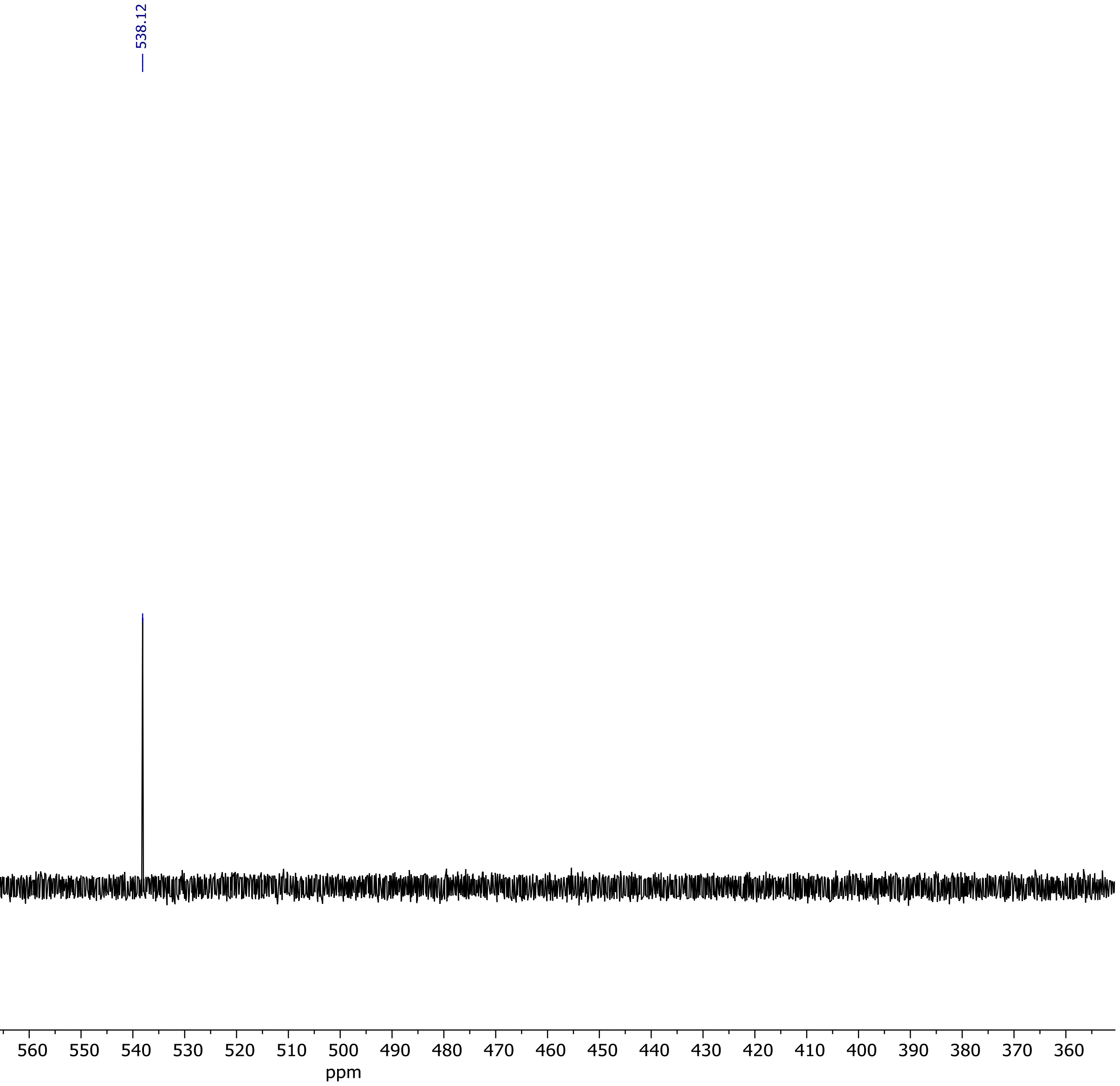

^{1}H NMR of **S67**
600 MHz, DMSO, 298 K

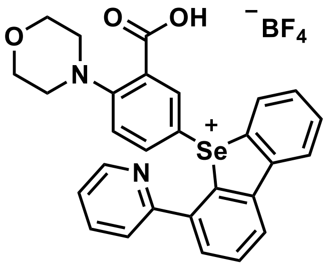

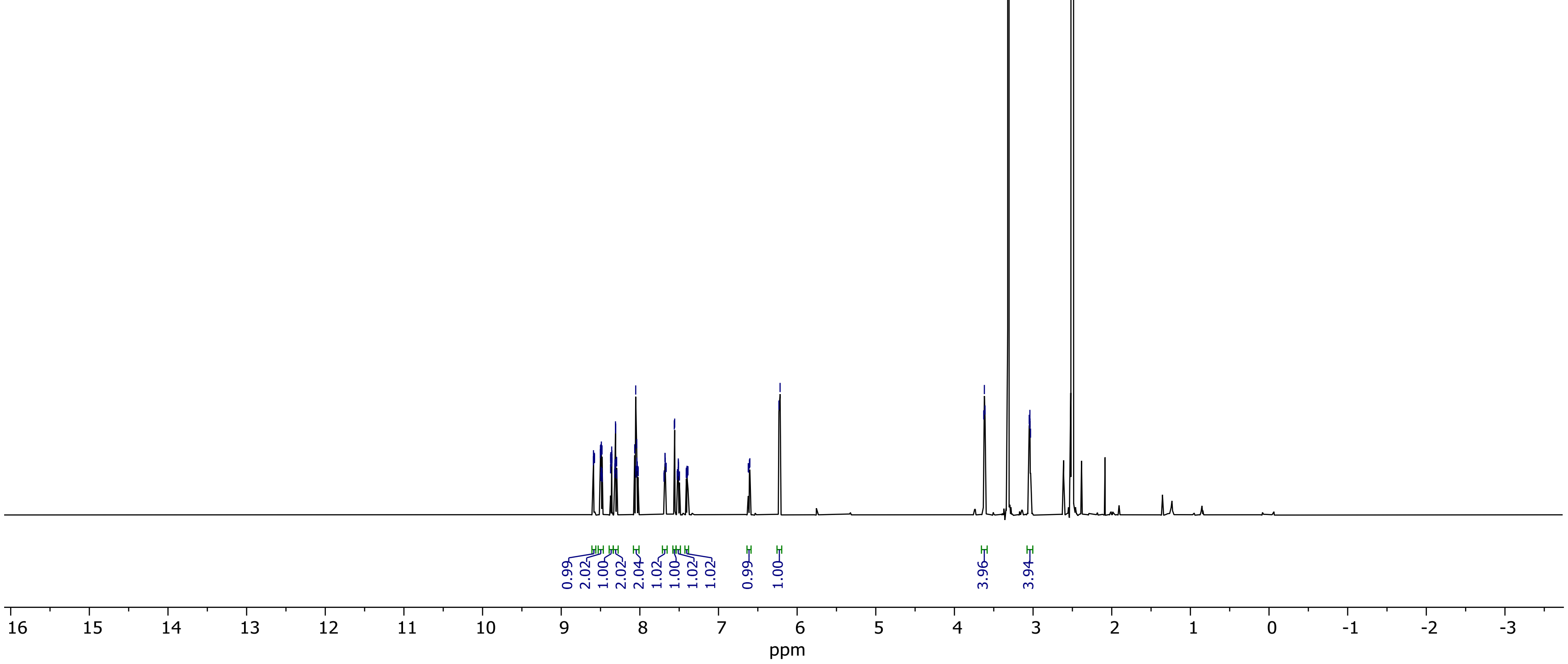

^{13}C NMR of **S67**
151 MHz, DMSO, 298 K

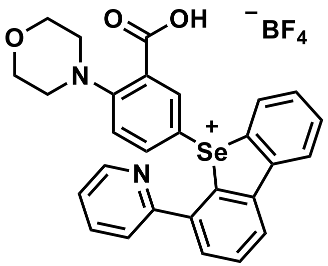

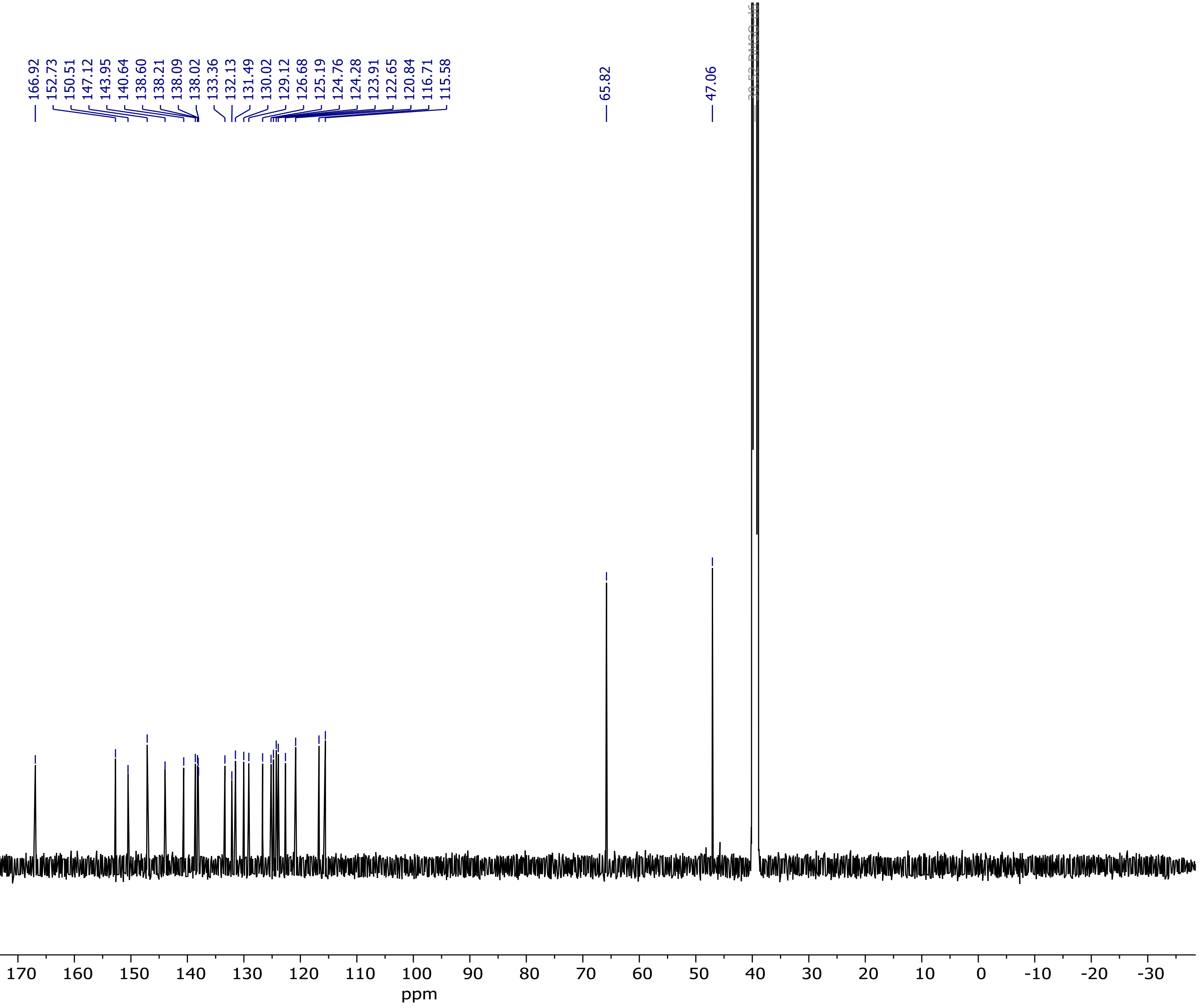

^{77}Se NMR of **S67**
115 MHz, DMSO, 298 K

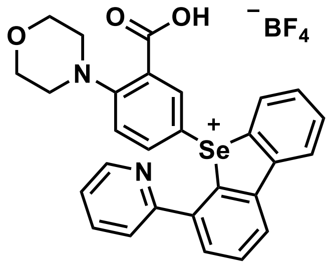

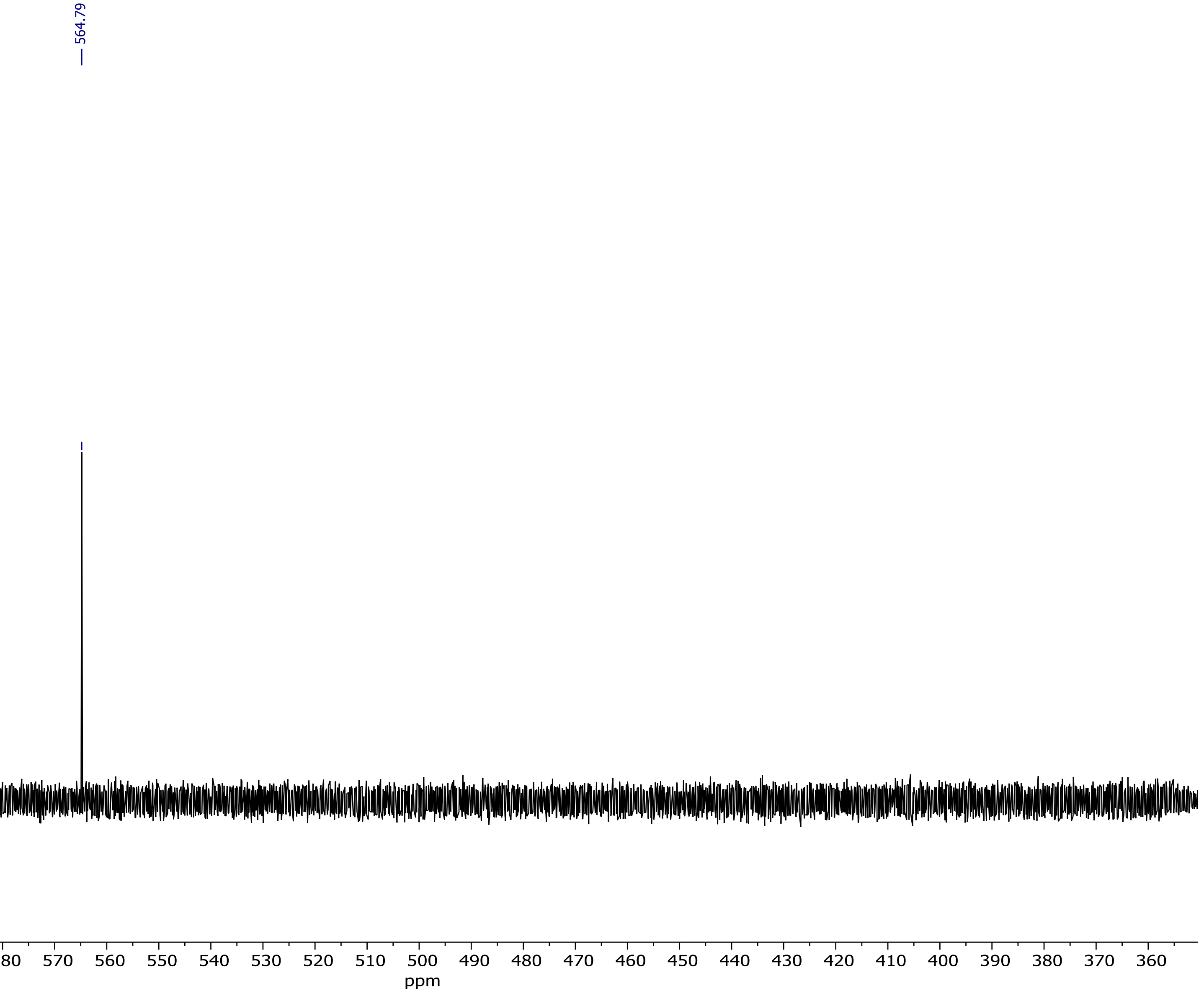

^{1}H NMR of **S68**
600 MHz, DMSO, 298 K

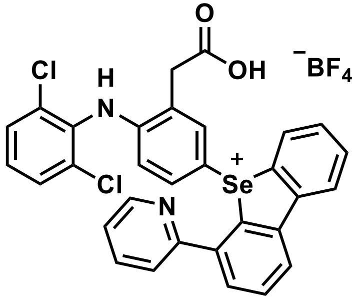

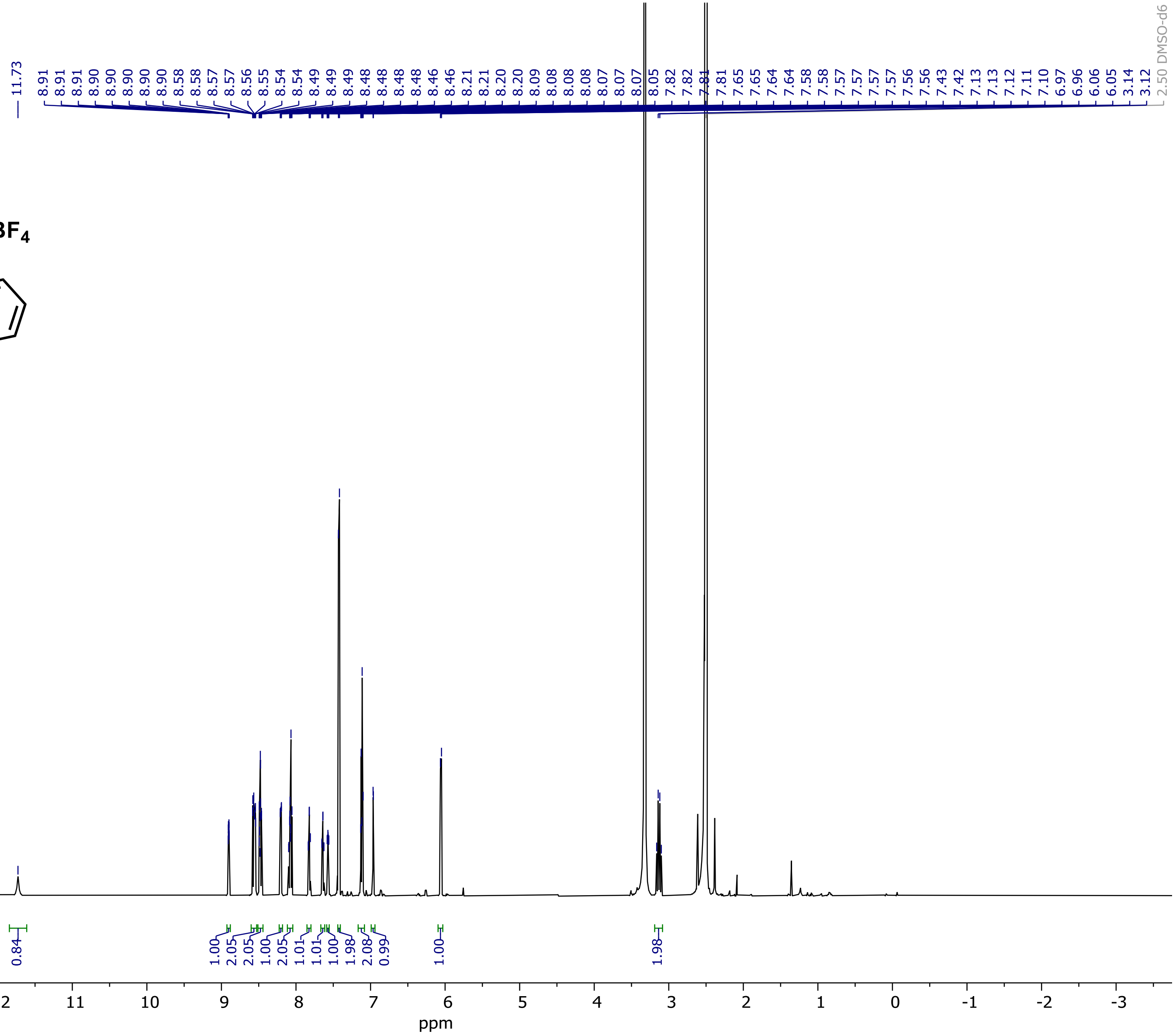

^{13}C NMR of **S68**
151 MHz, DMSO, 298 K

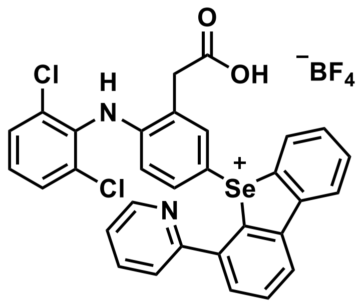

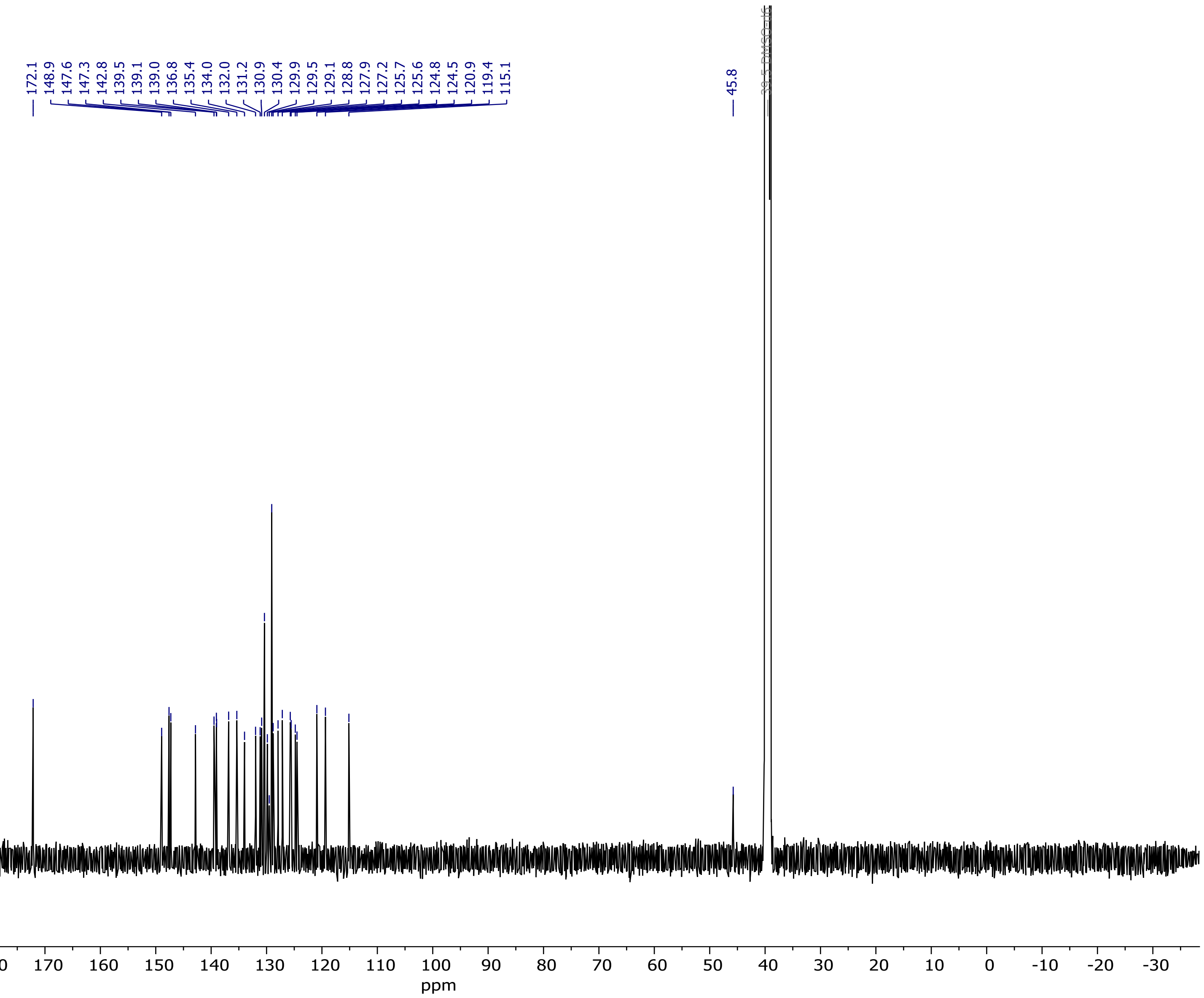

^{19}F NMR of **S68**
565 MHz, DMSO, 298 K

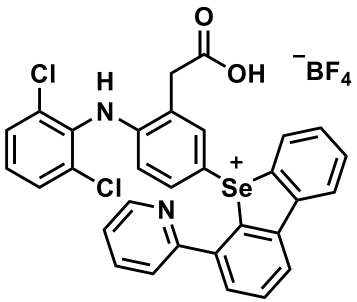

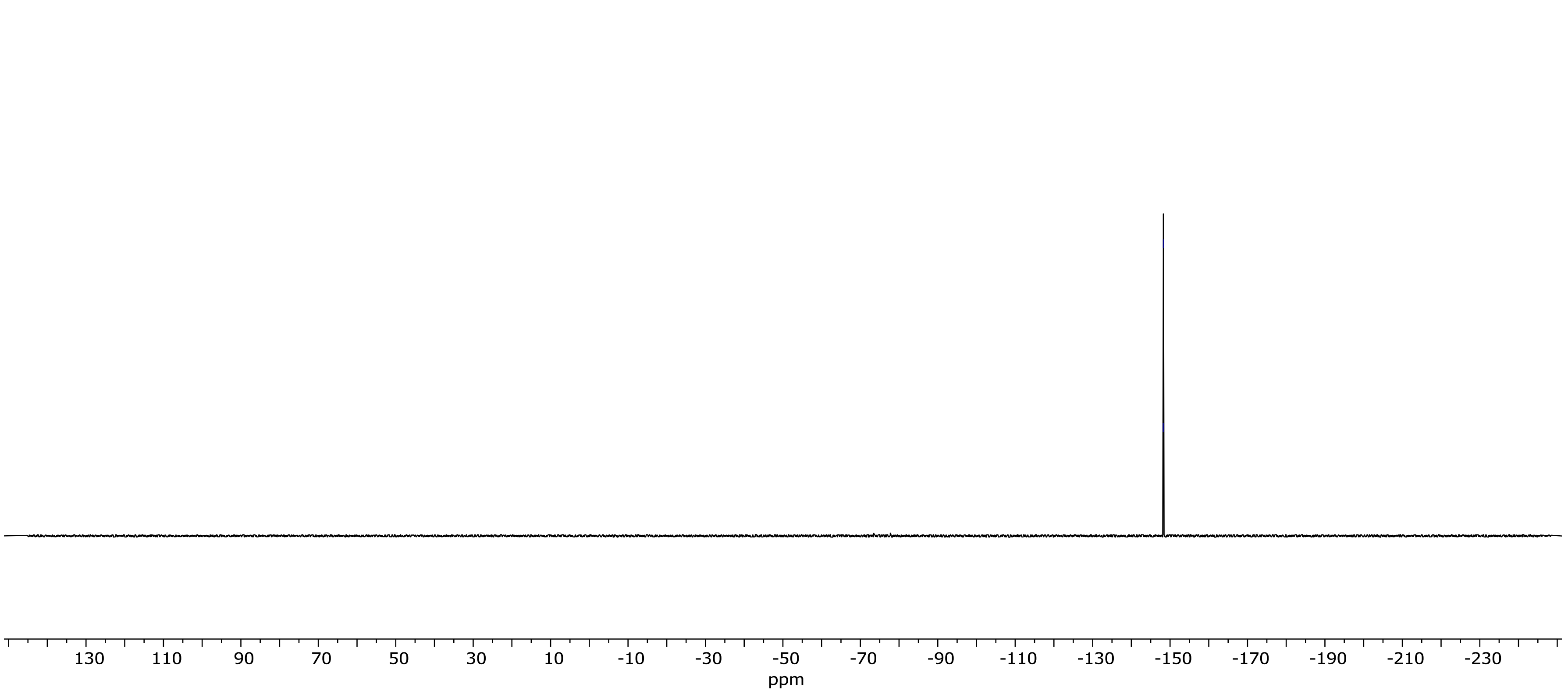

^{77}Se NMR of **S68**
115 MHz, DMSO, 298 K

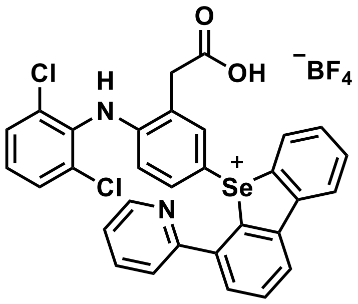

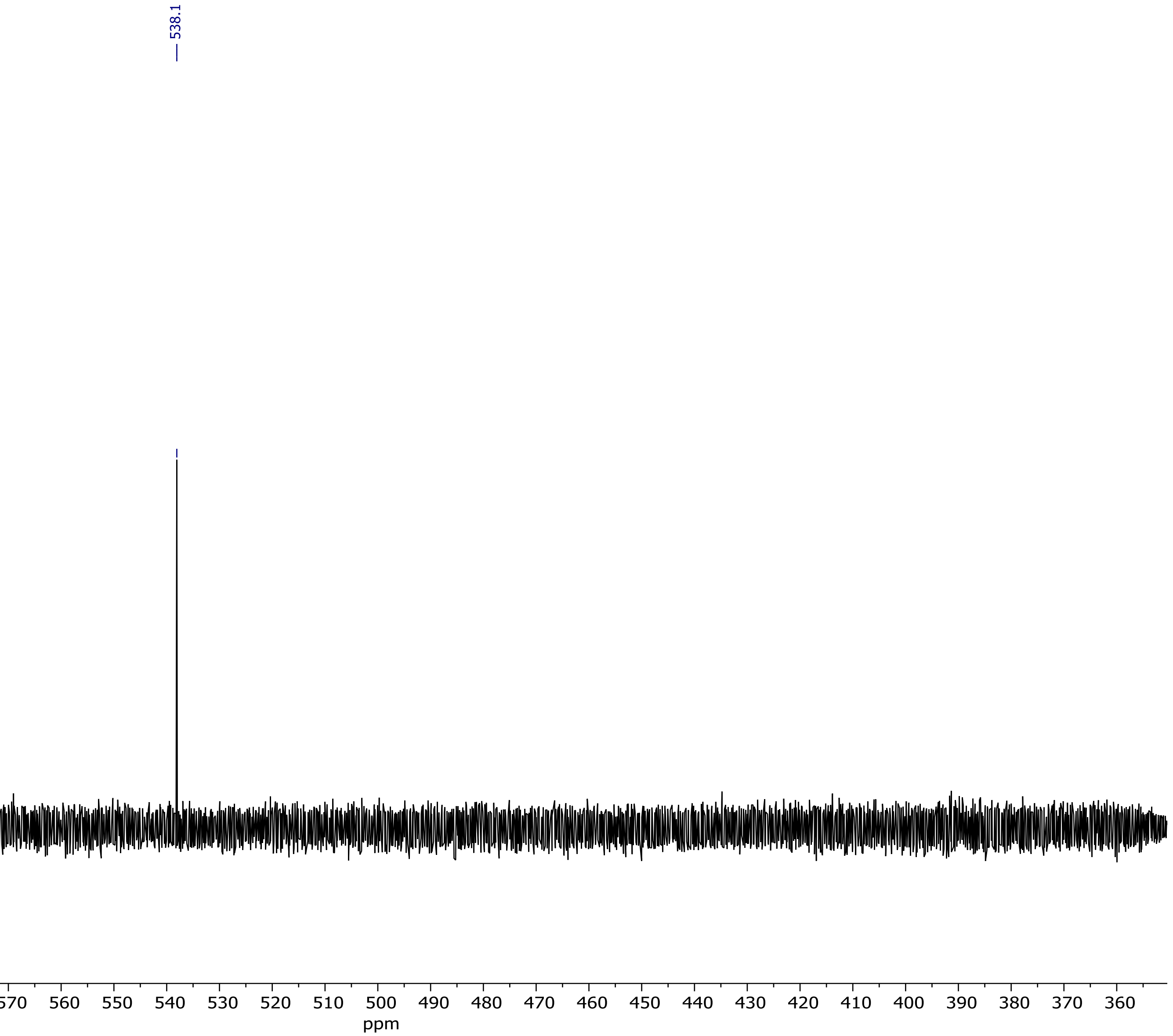

^{1}H NMR of **S69**

600 MHz, CD_3CN, CD_3OD, 298 K

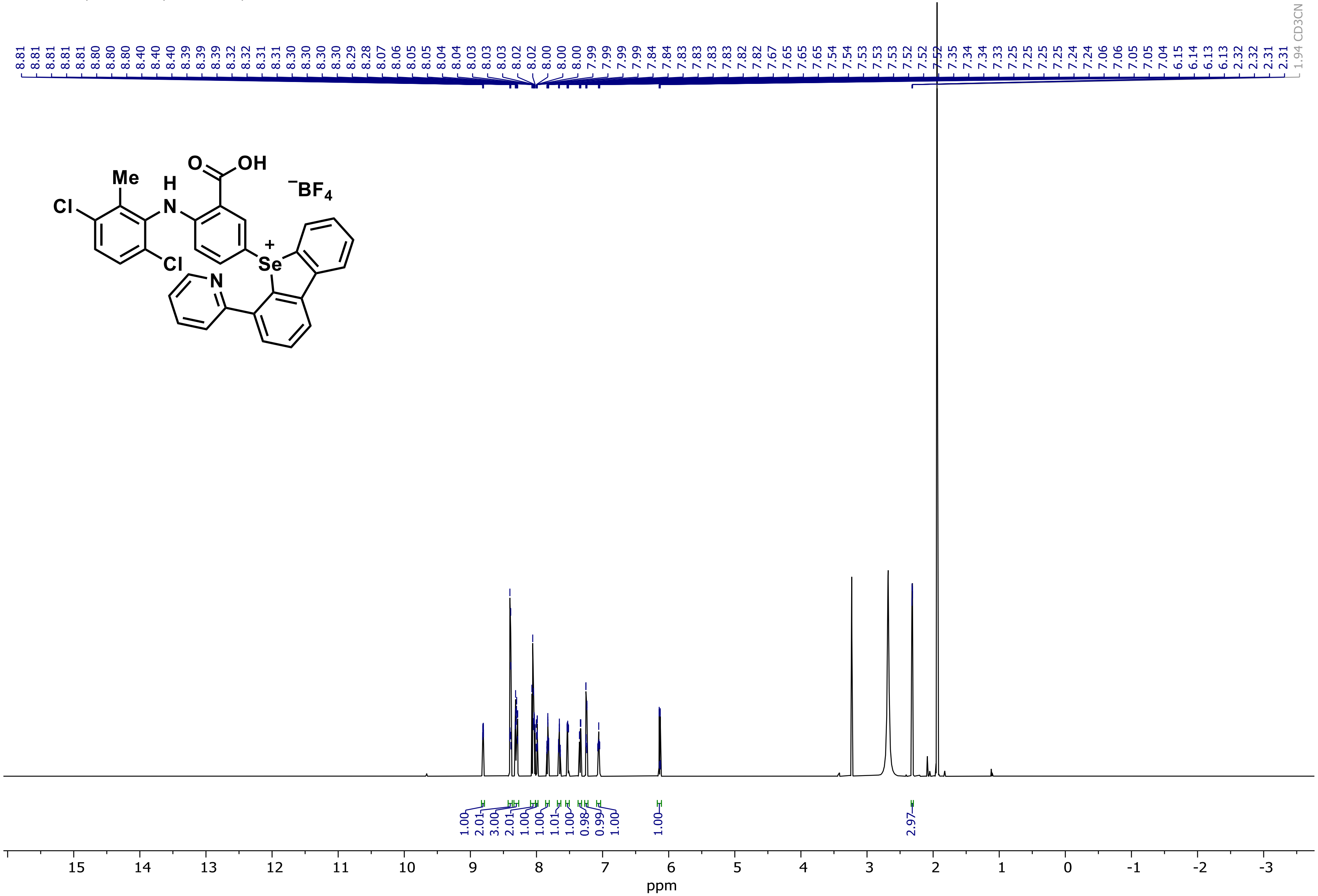

^{13}C NMR of **S69**
151 MHz, CD_3CN, CD_3OD, 298 K

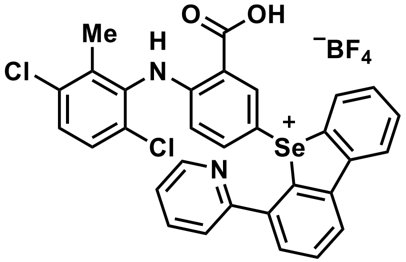

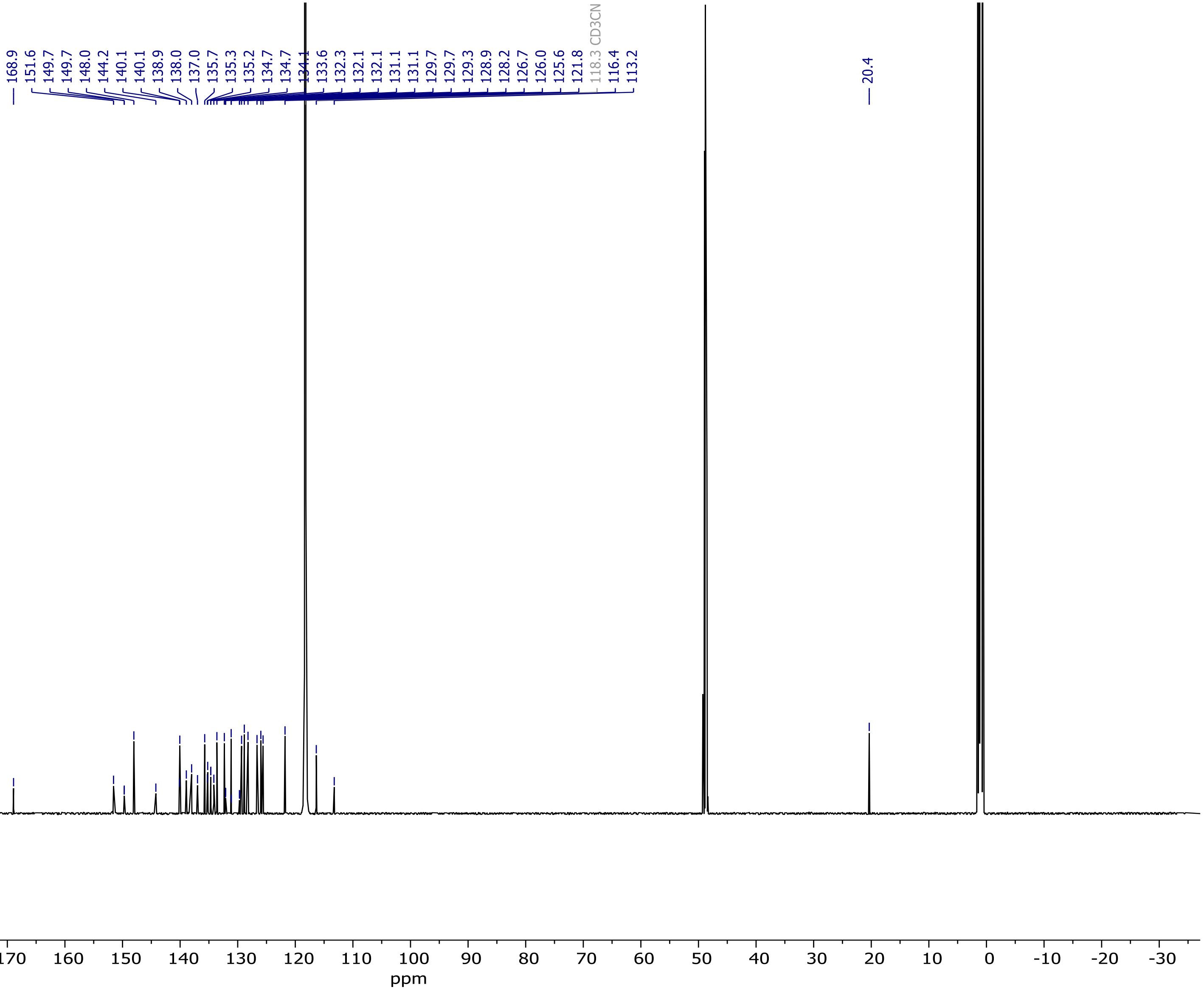

^{19}F NMR of **S69**
565 MHz, CD_3CN, CD_3OD, 298 K

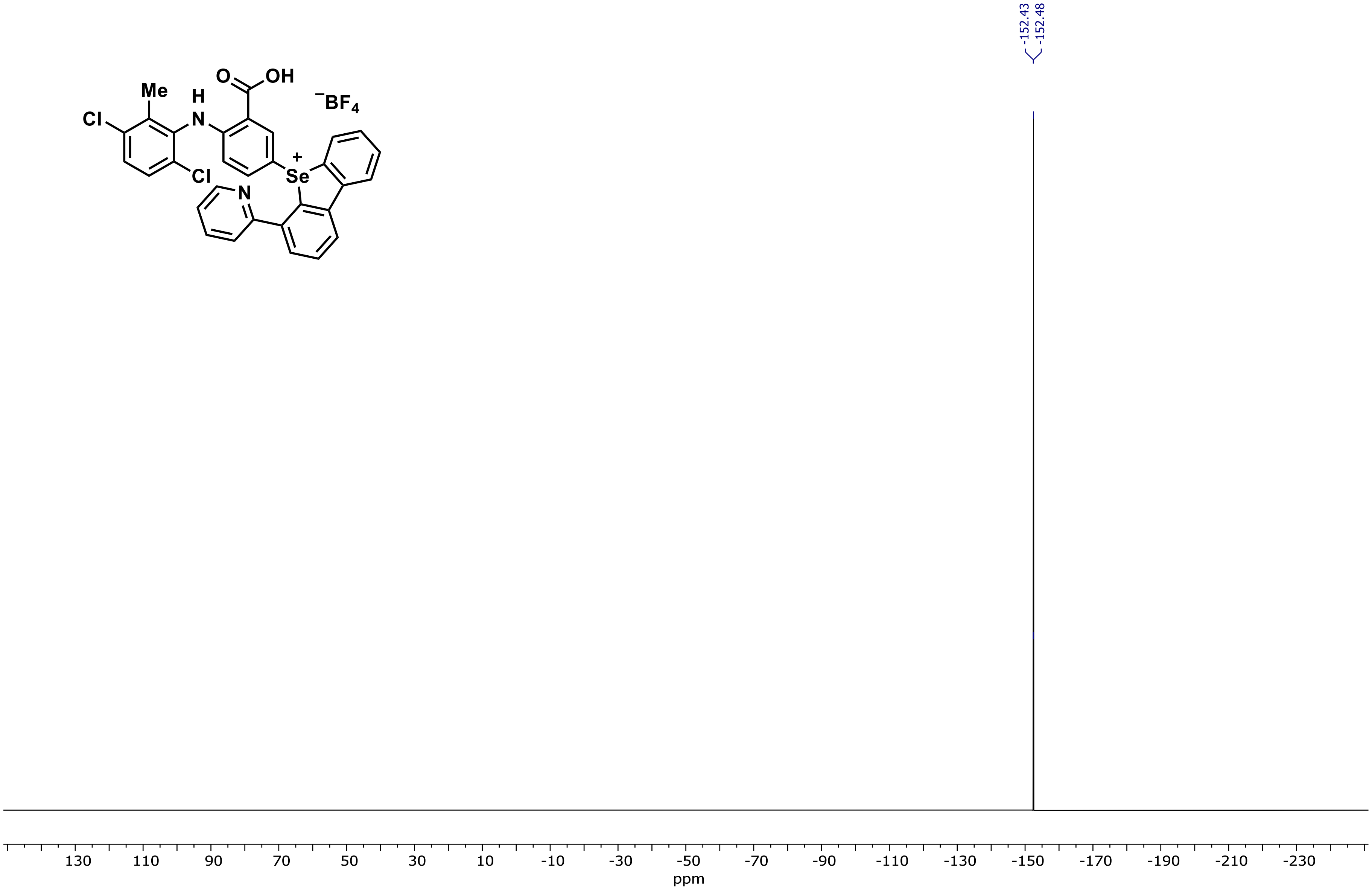

^{77}Se NMR of **S69**
115 MHz, CD_3CN, CD_3OD, 298 K

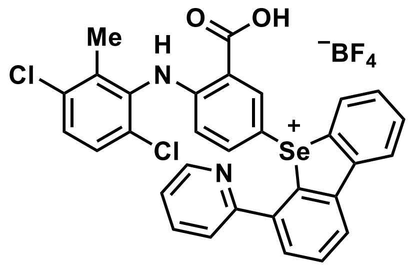

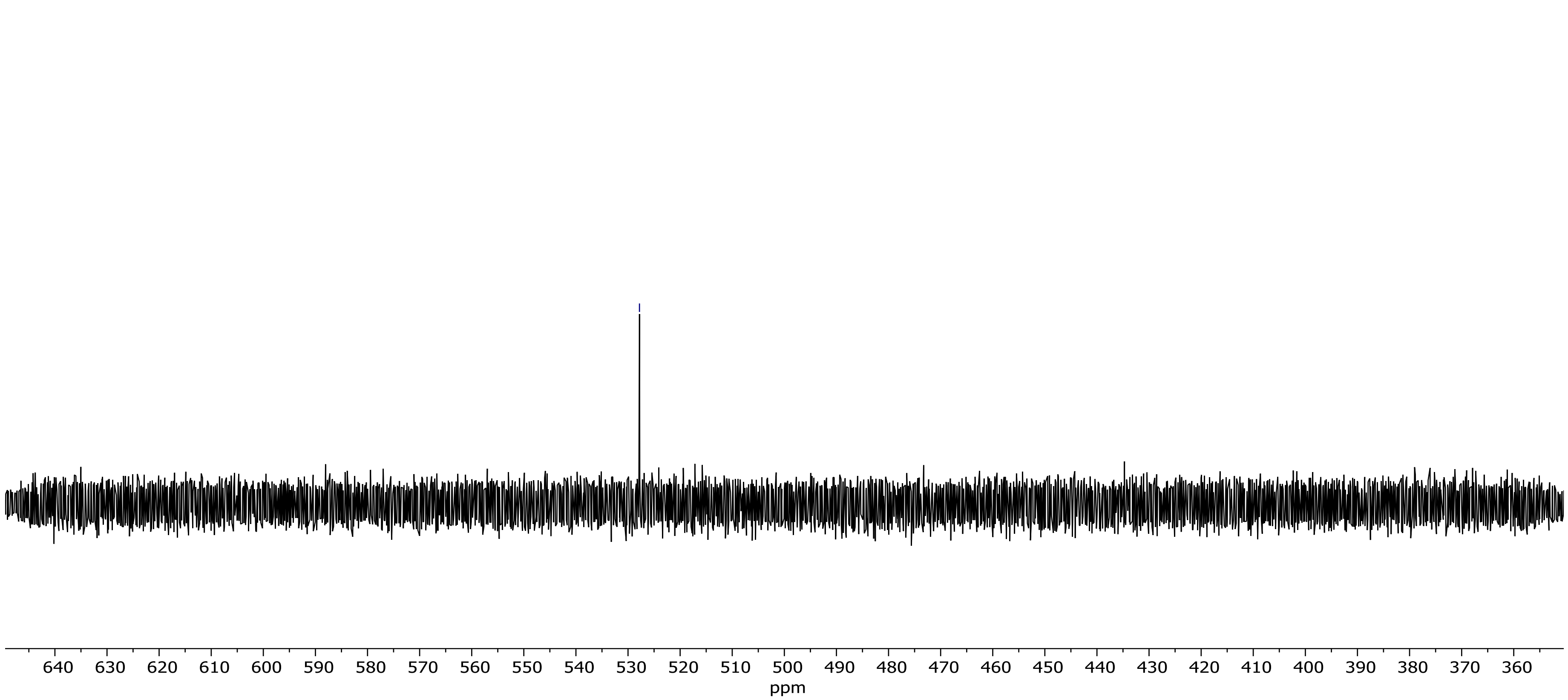

^{1}H NMR of **S70**
600 MHz, DMSO, 298 K

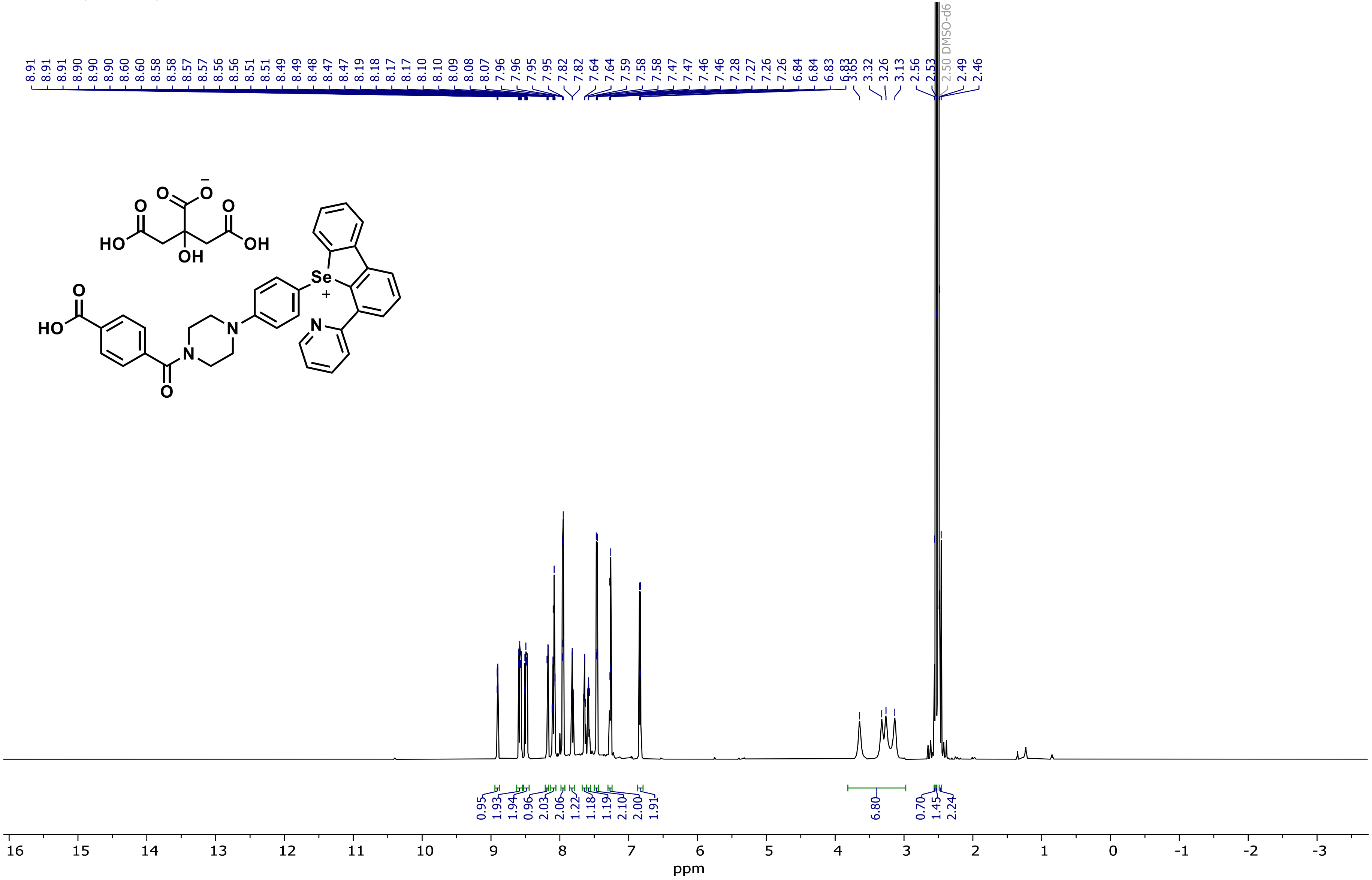

^{13}C NMR of **S70**
151 MHz, DMSO, 298 K

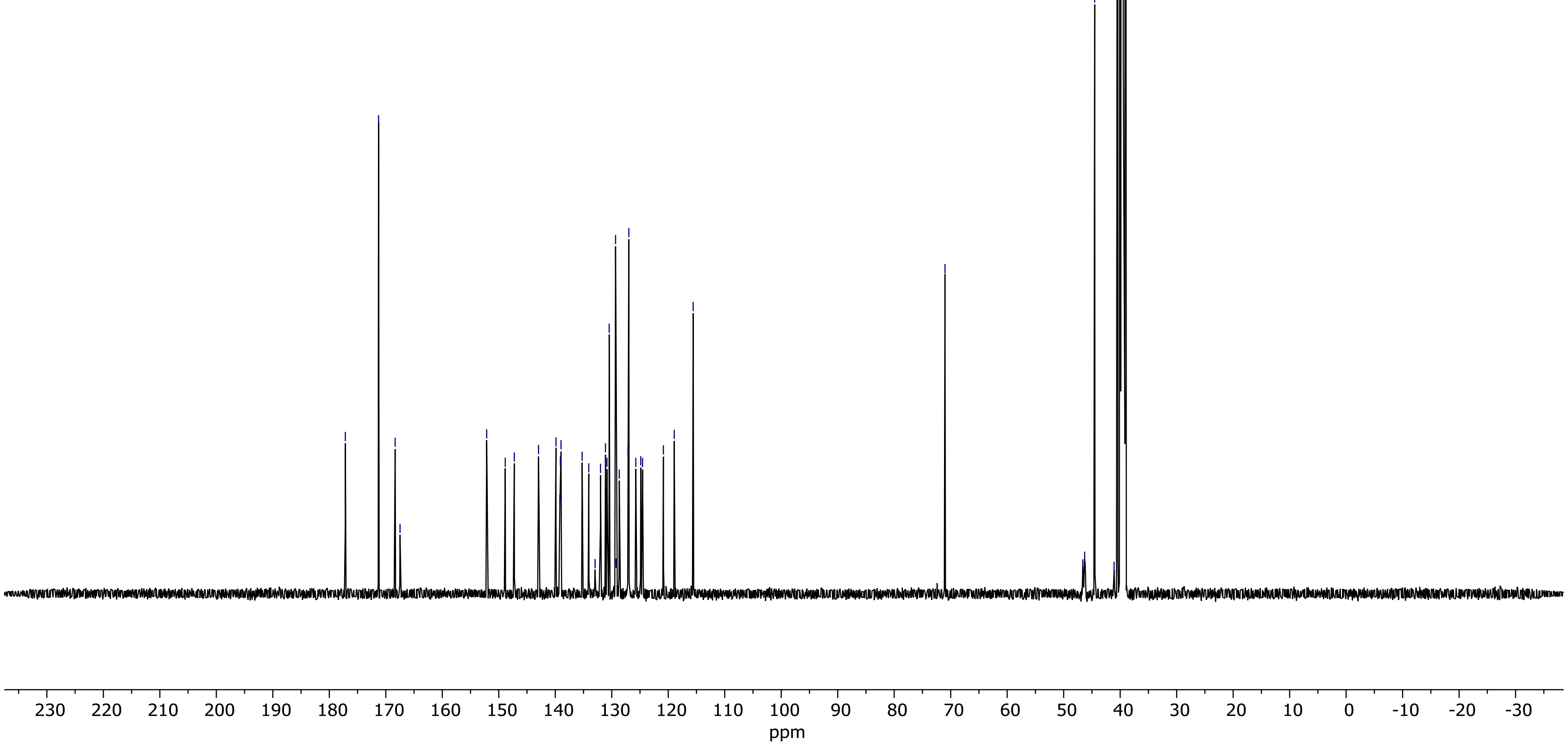

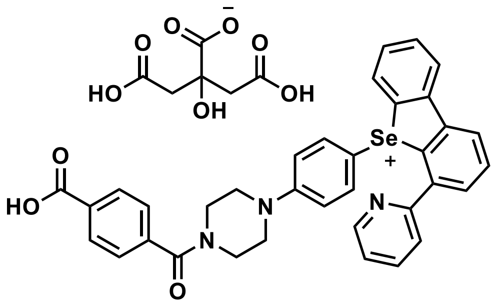

^{1}H NMR of **S71**
600 MHz, CD_3CN, 298 K

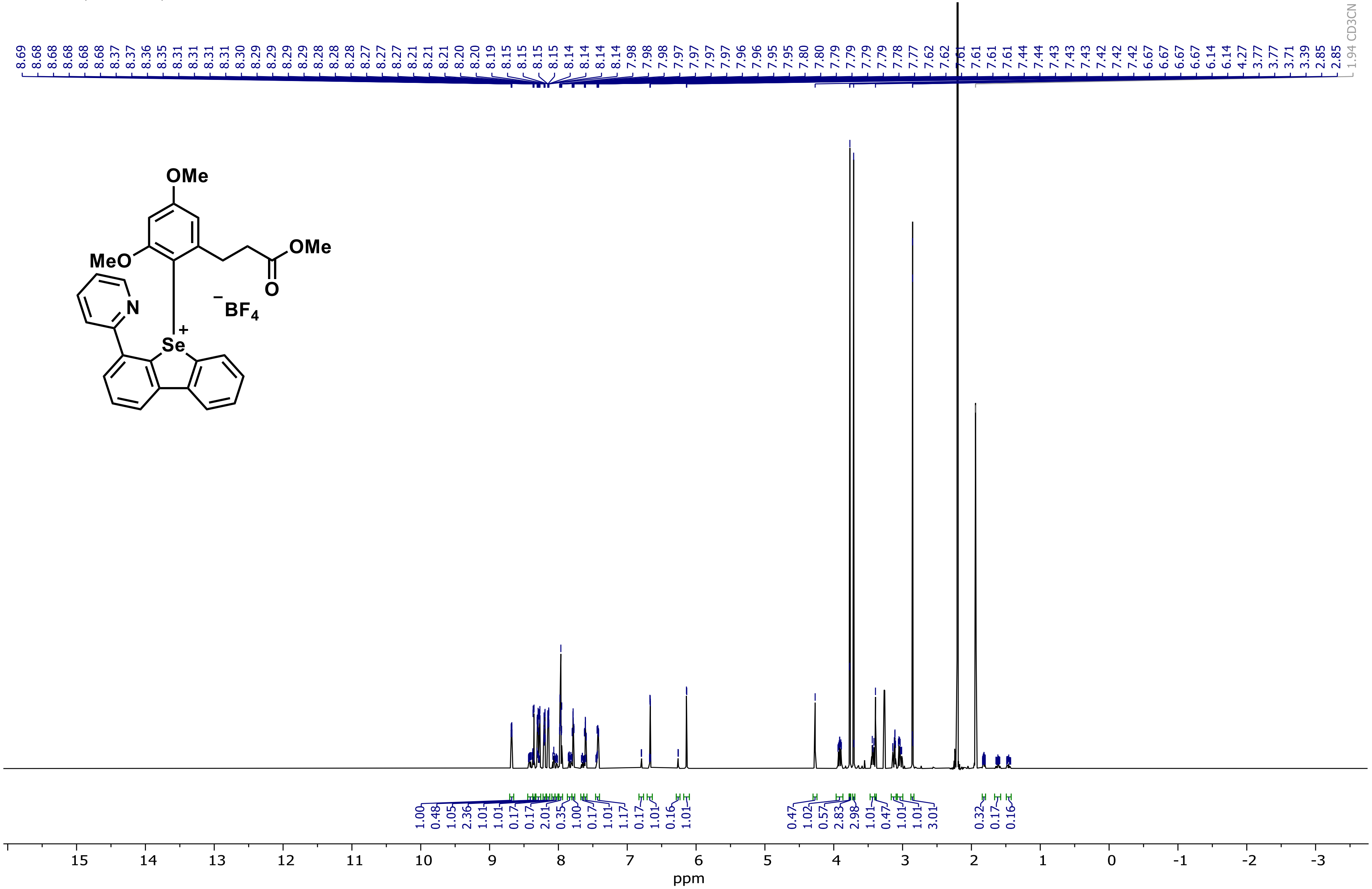

^{13}C NMR of **S71**
151 MHz, CD_3CN, 298 K

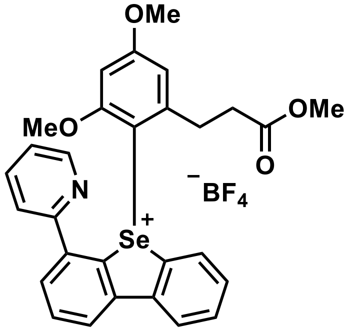

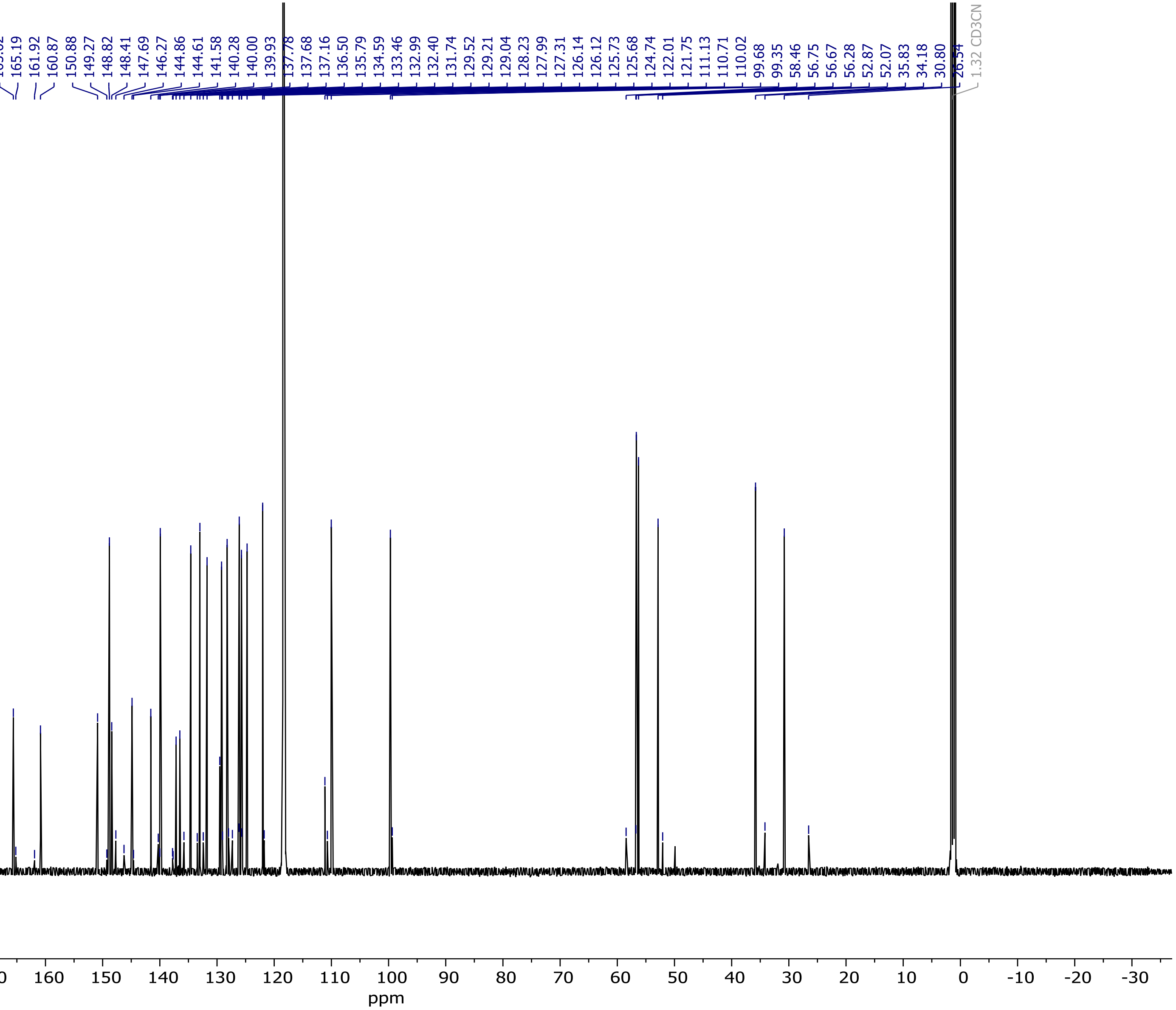

^{19}F NMR of **S71**
565 MHz, CD_3CN, 298 K

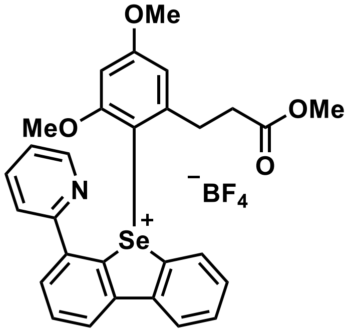

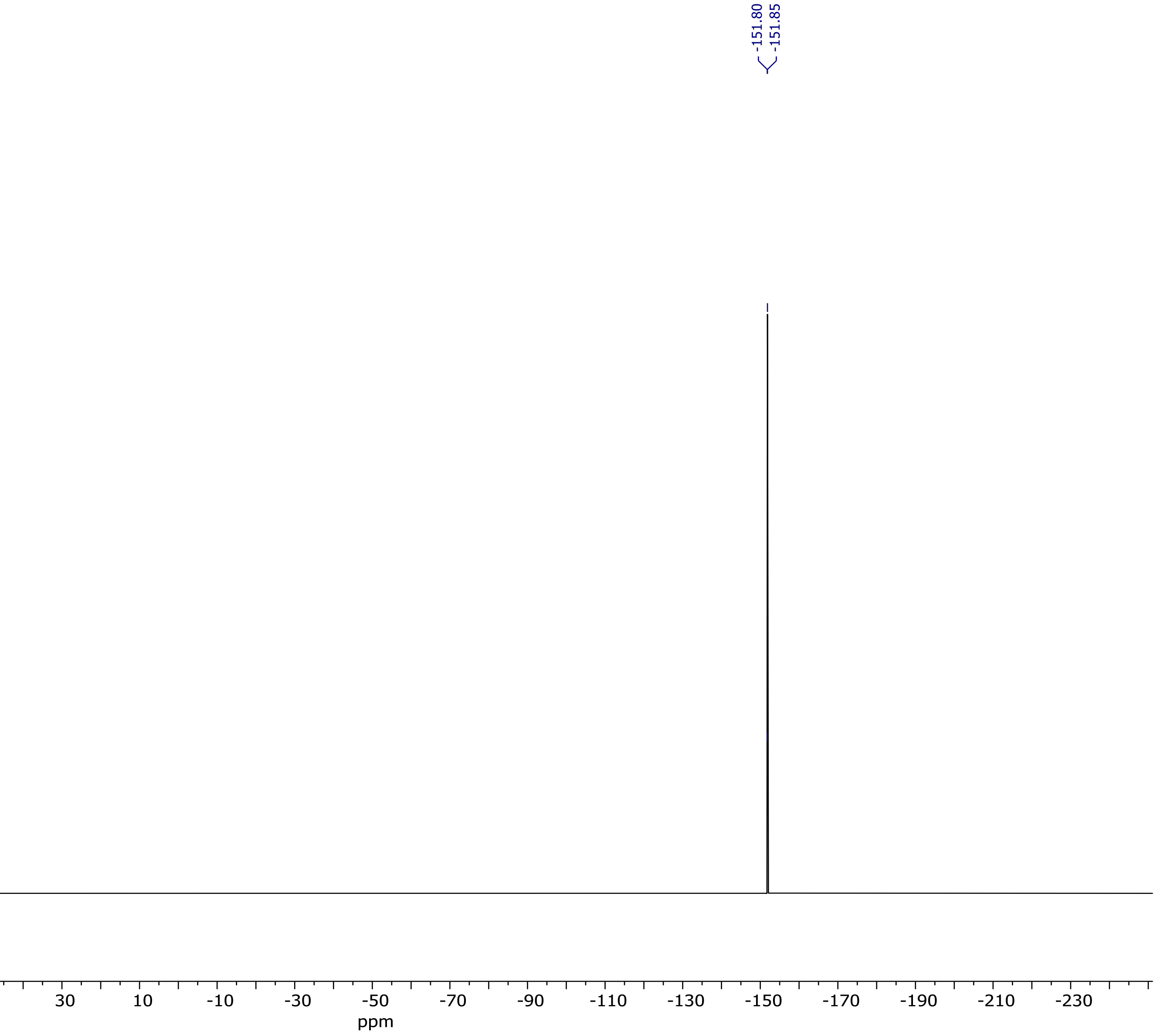

^{77}Se NMR of **S71**
115 MHz, CD_3CN, 298 K

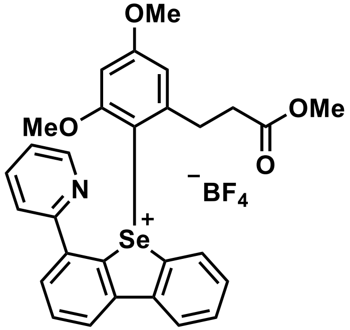

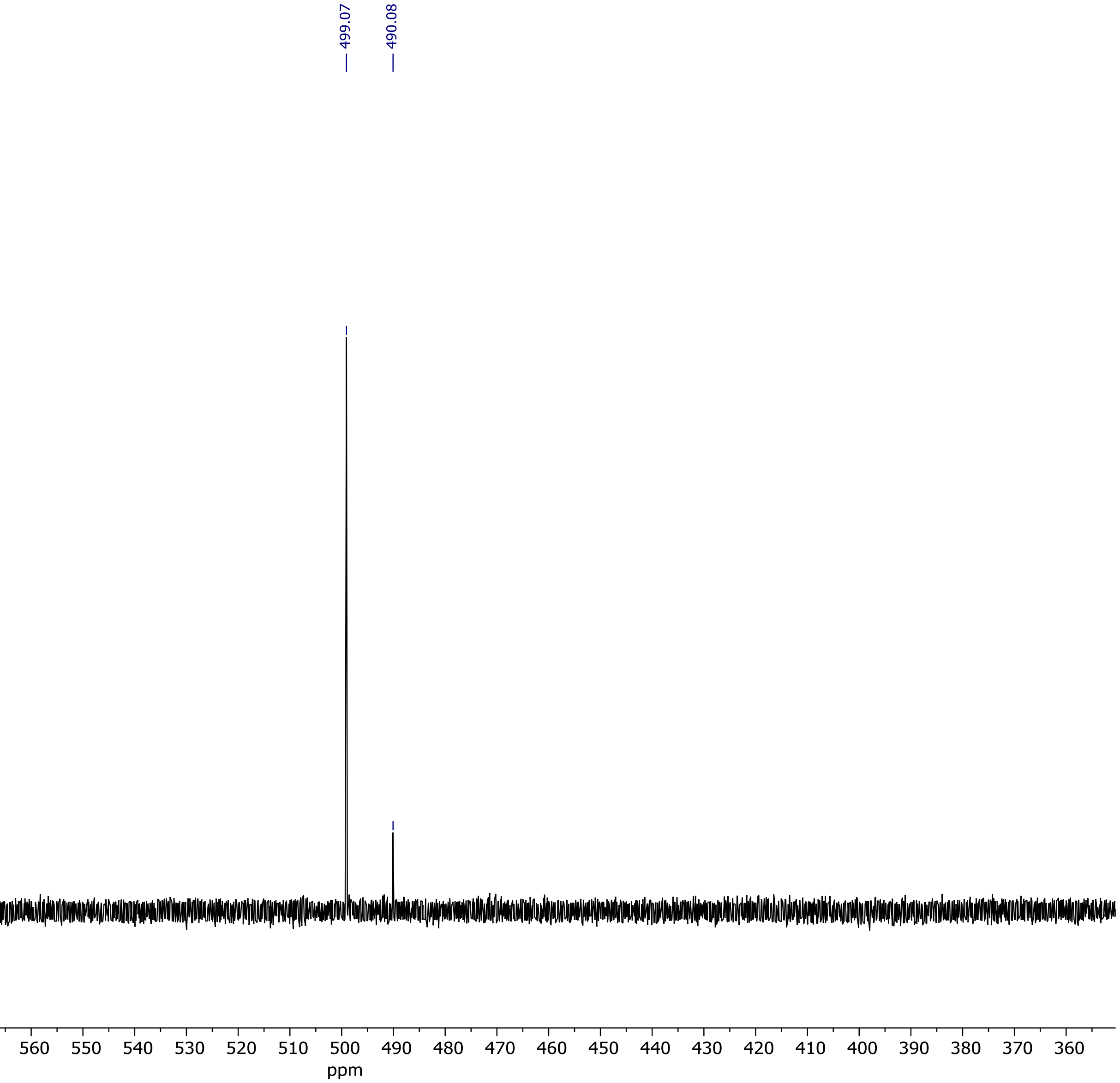

NOESY NMR of **S71**
600 MHz, CD_3CN, 298 K

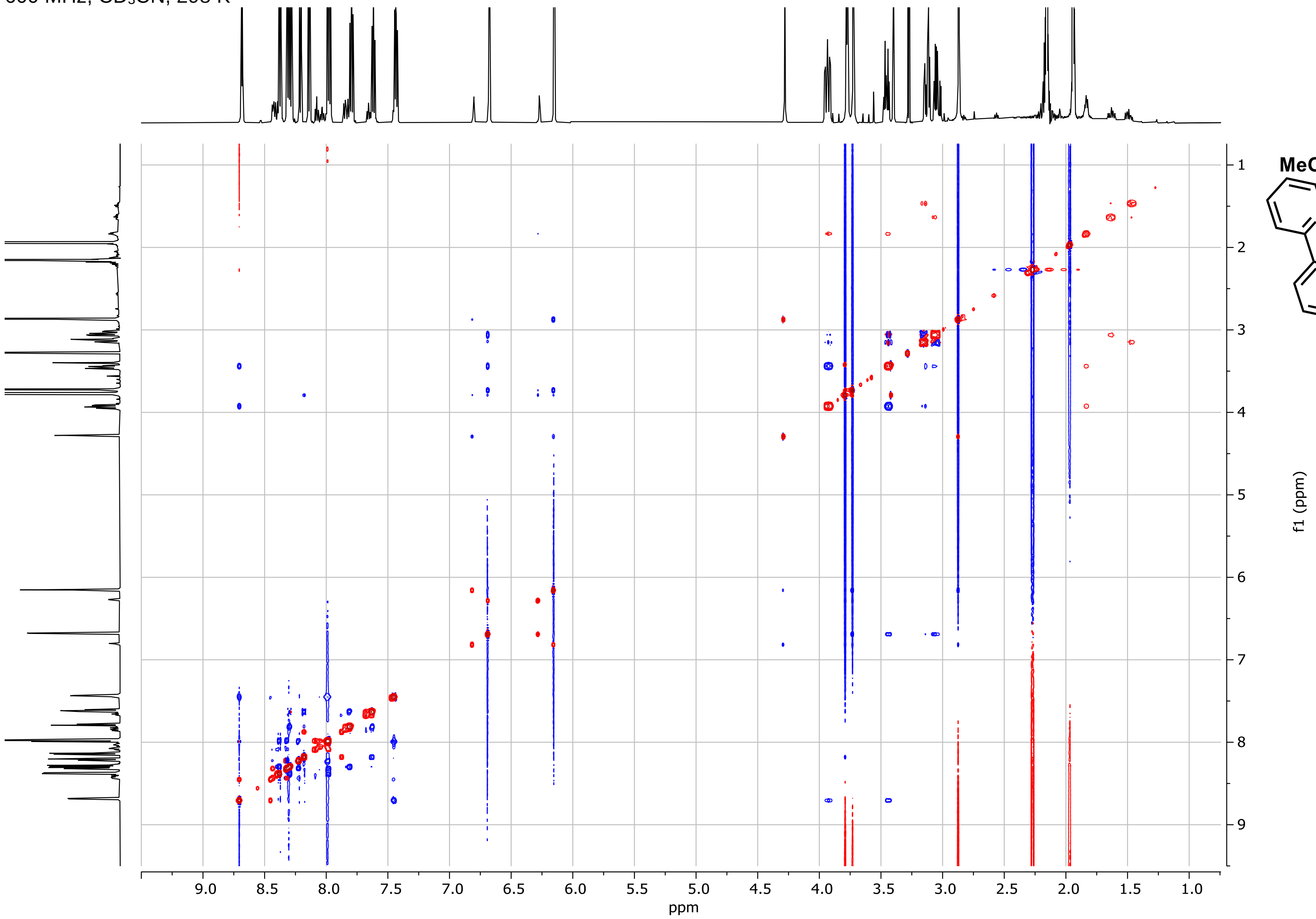

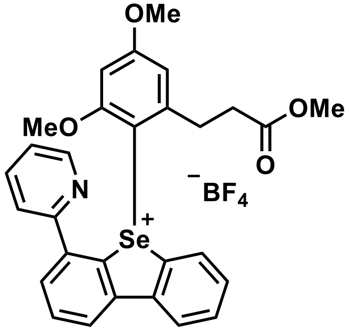

^{1}H NMR of **S72**
500 MHz, $CDCl_3$, 298 K

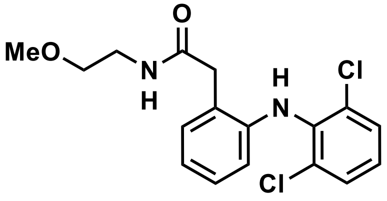

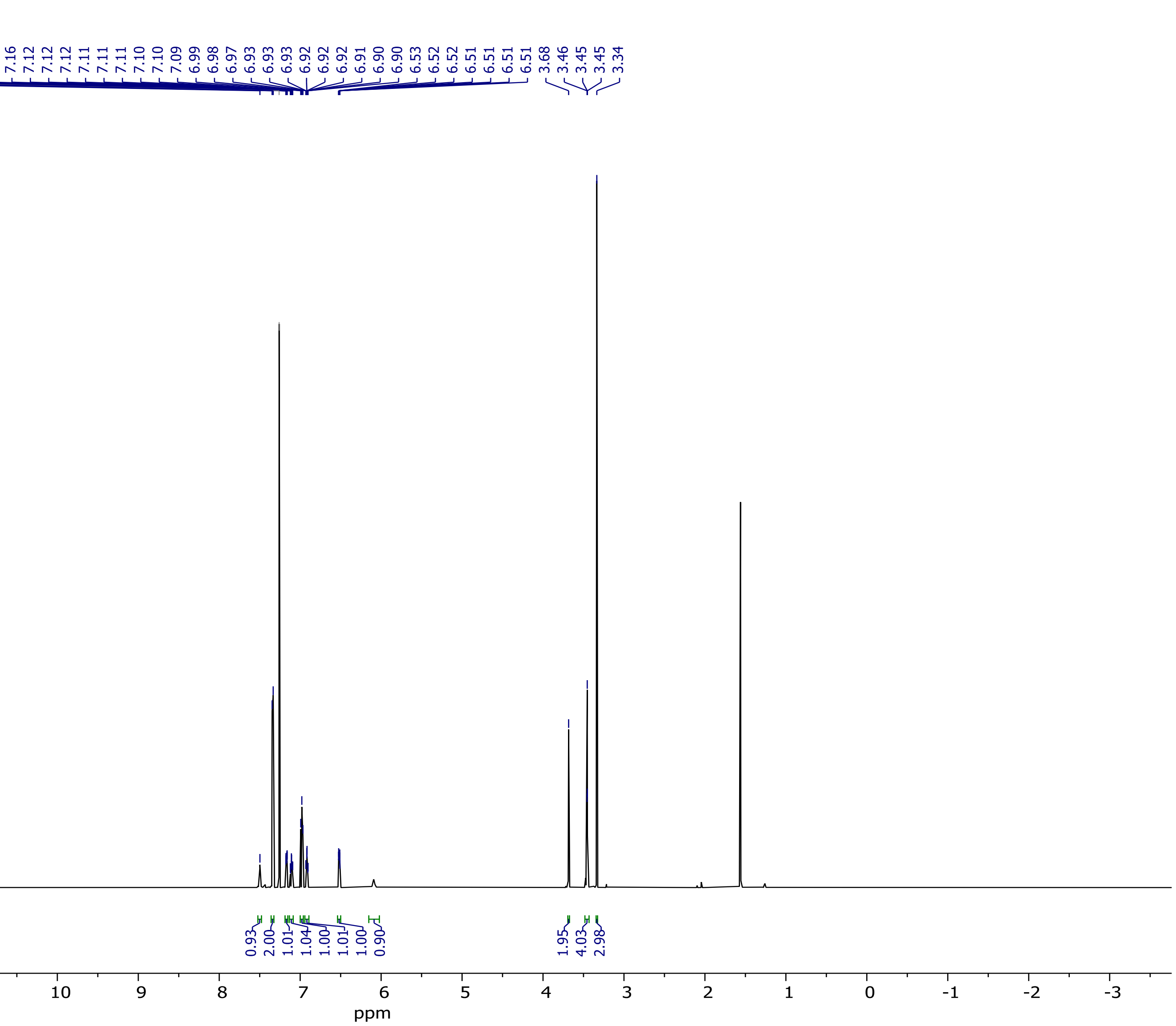

^{13}C NMR of **S72**
151 MHz, $CDCl_3$, 298 K

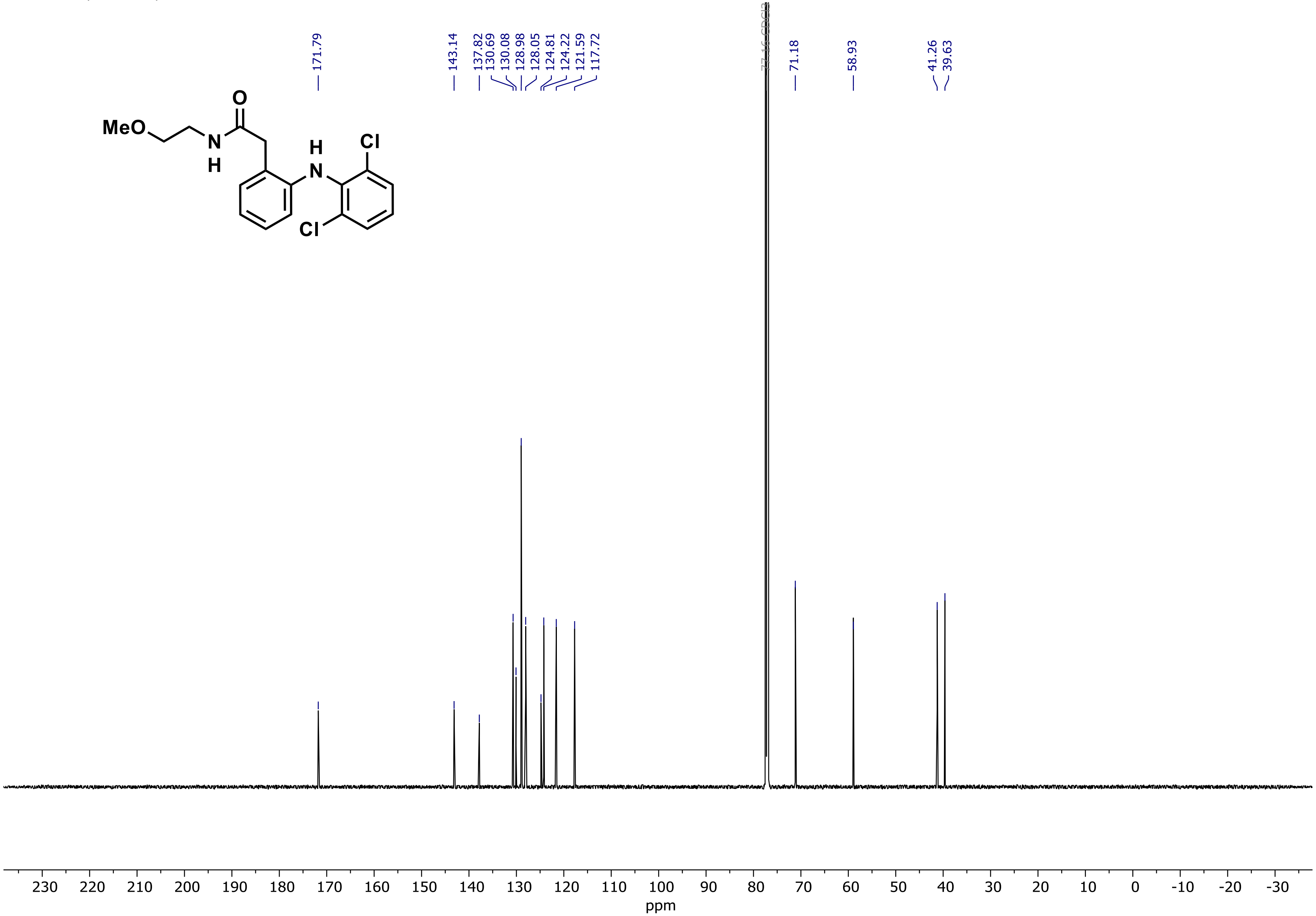

^{1}H NMR of **S73**
600 MHz, DMSO, 298 K

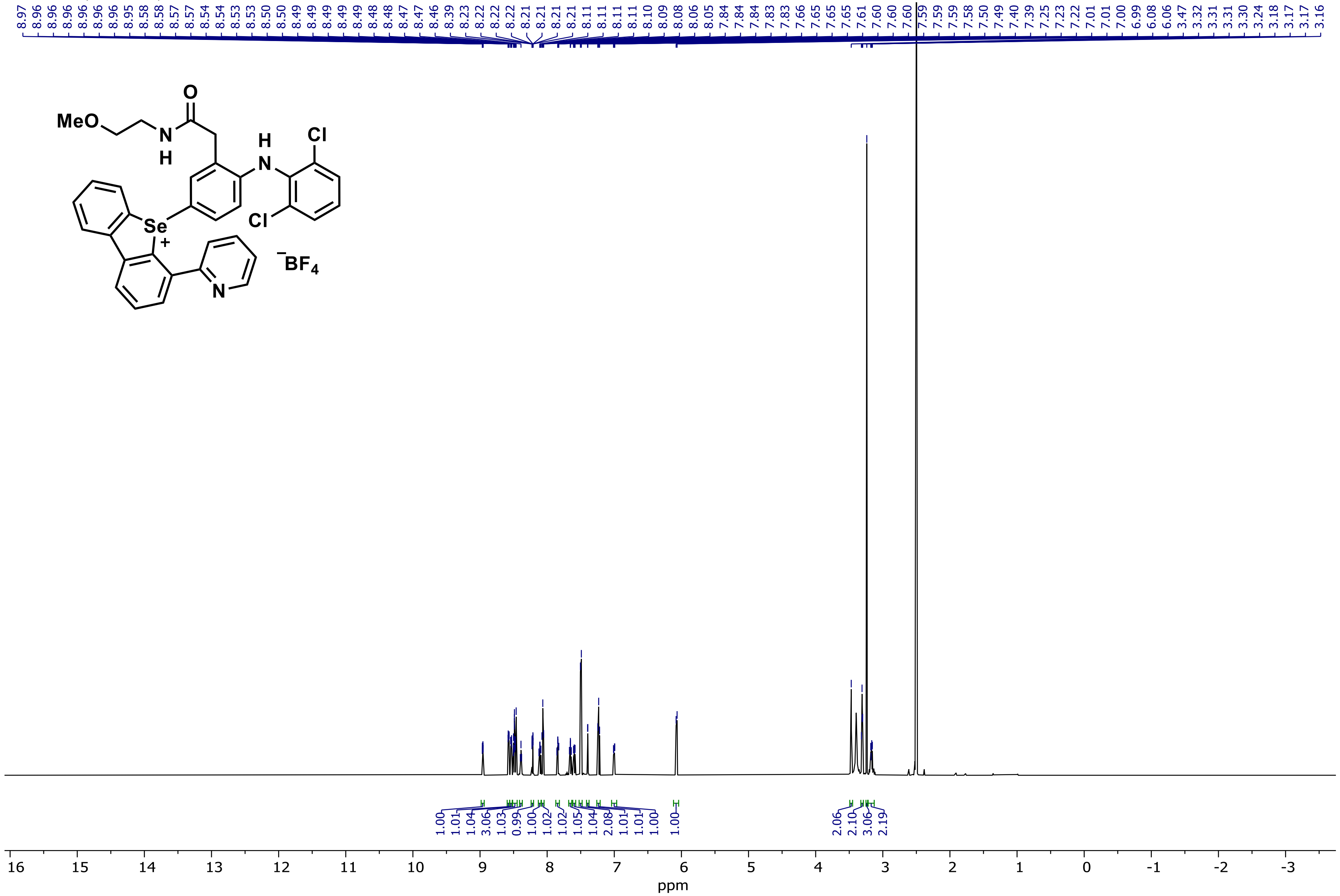

^{13}C NMR of **S73**
151 MHz, DMSO, 298 K

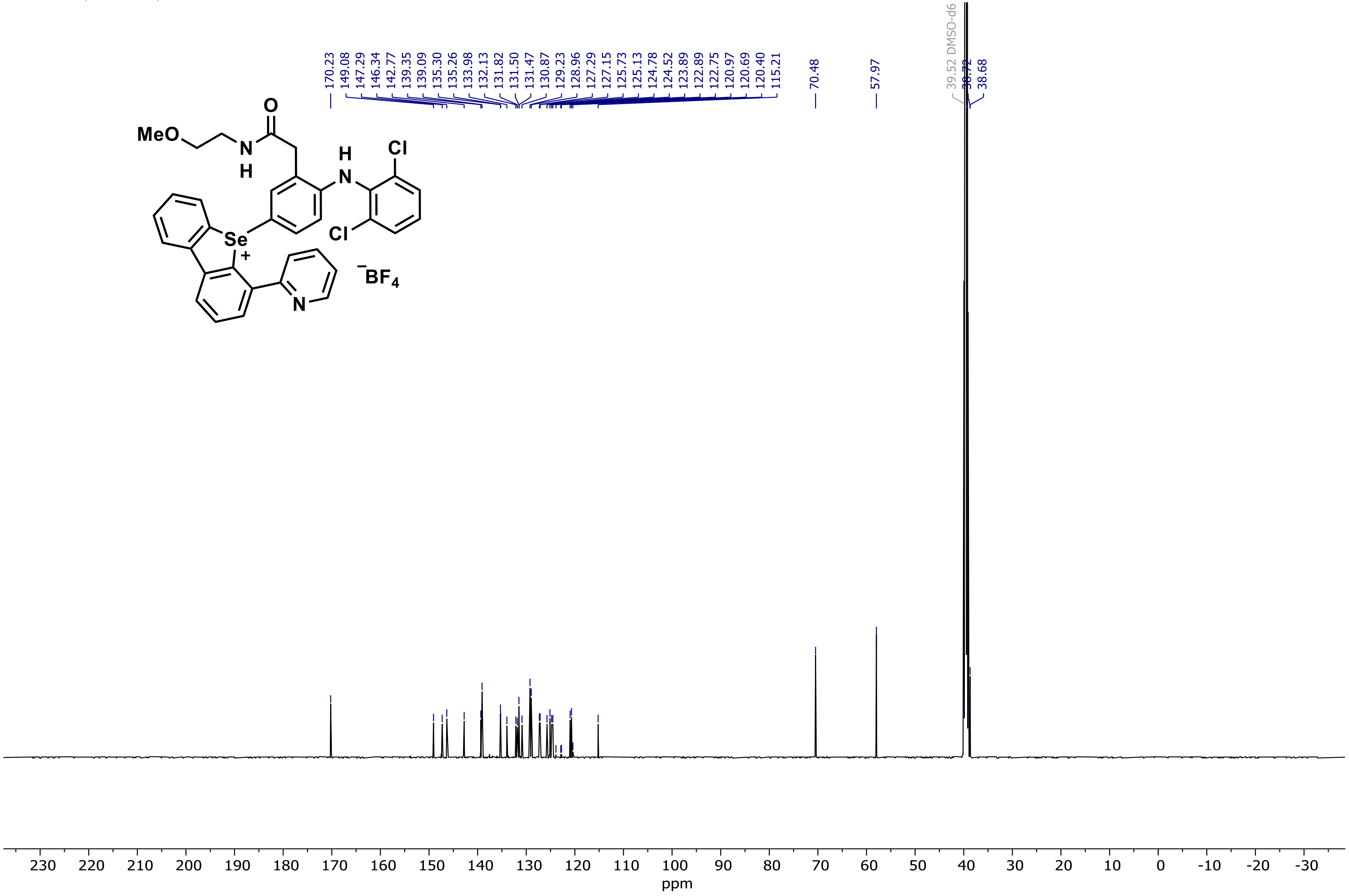

^{19}F NMR of **S73**
565 MHz, DMSO, 298 K

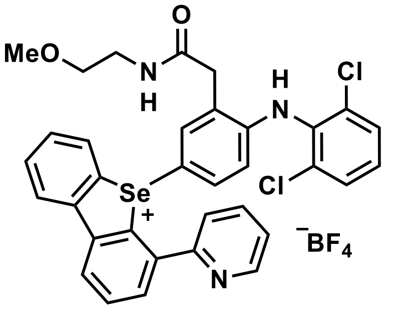

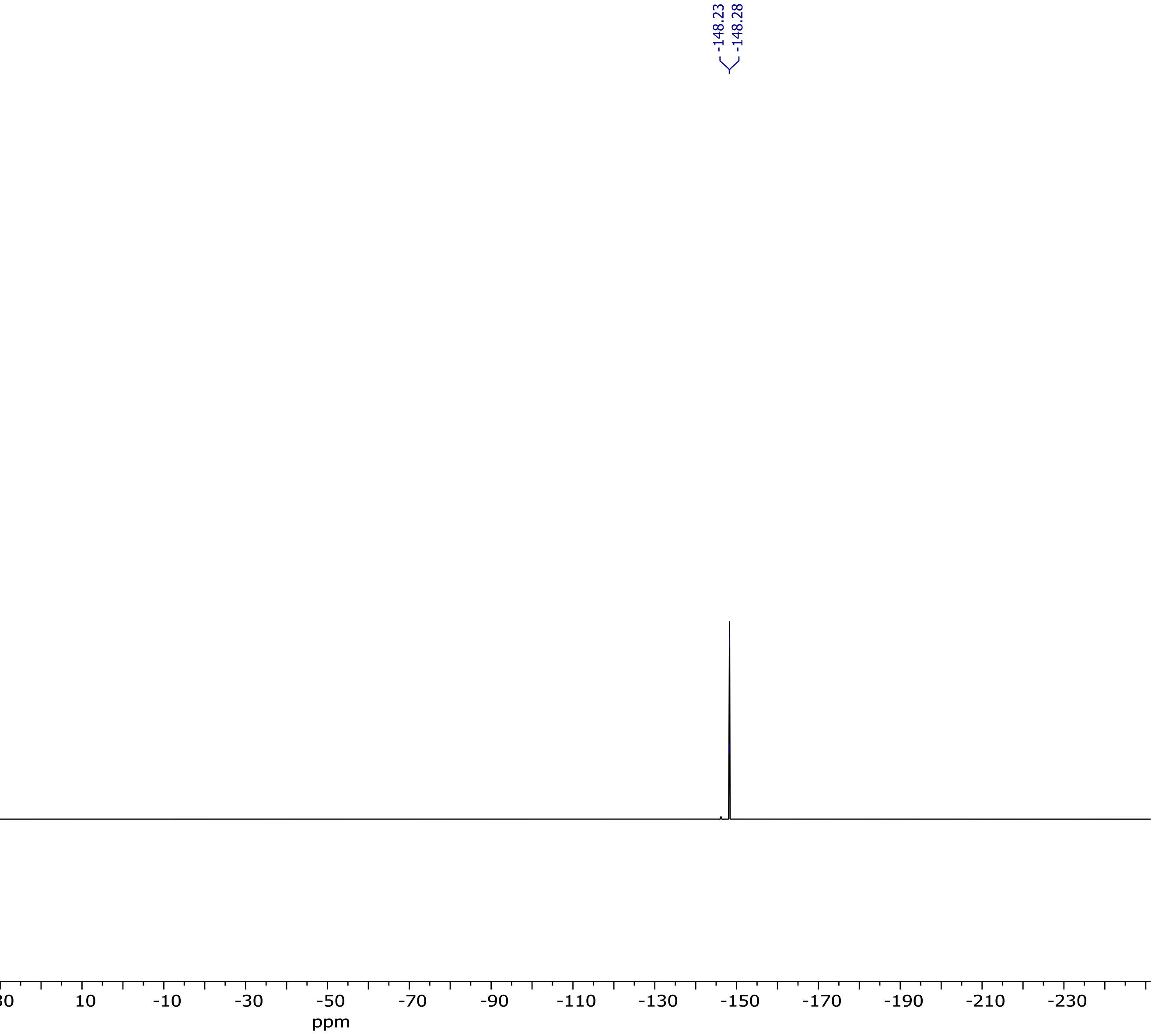

^{77}Se NMR of **S73**
115 MHz, DMSO, 298 K

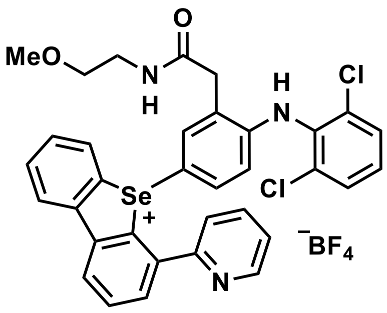

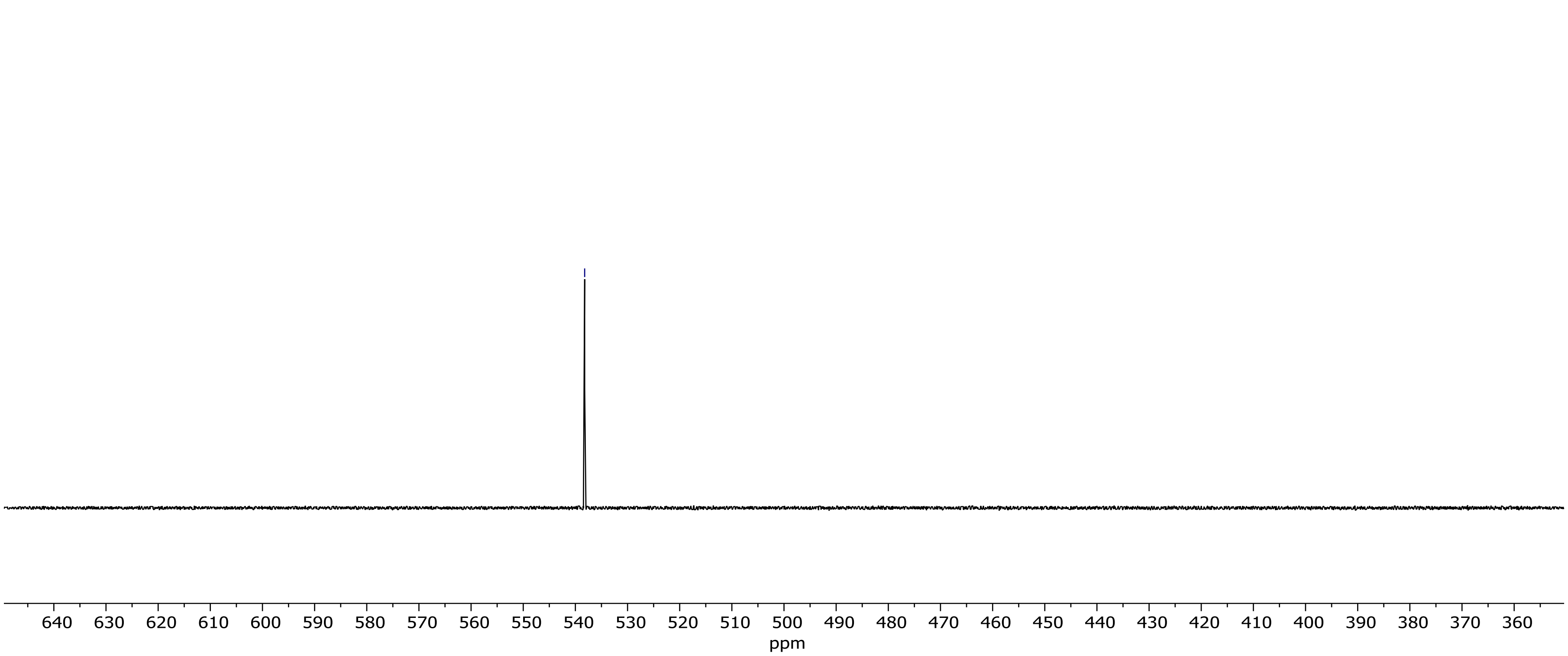

NMR-Characterization of “off DNA” following transformations

^{1}H NMR of **S74**
500 MHz, CD_2Cl_2, 298 K

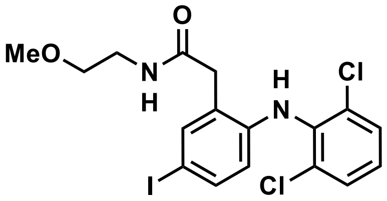

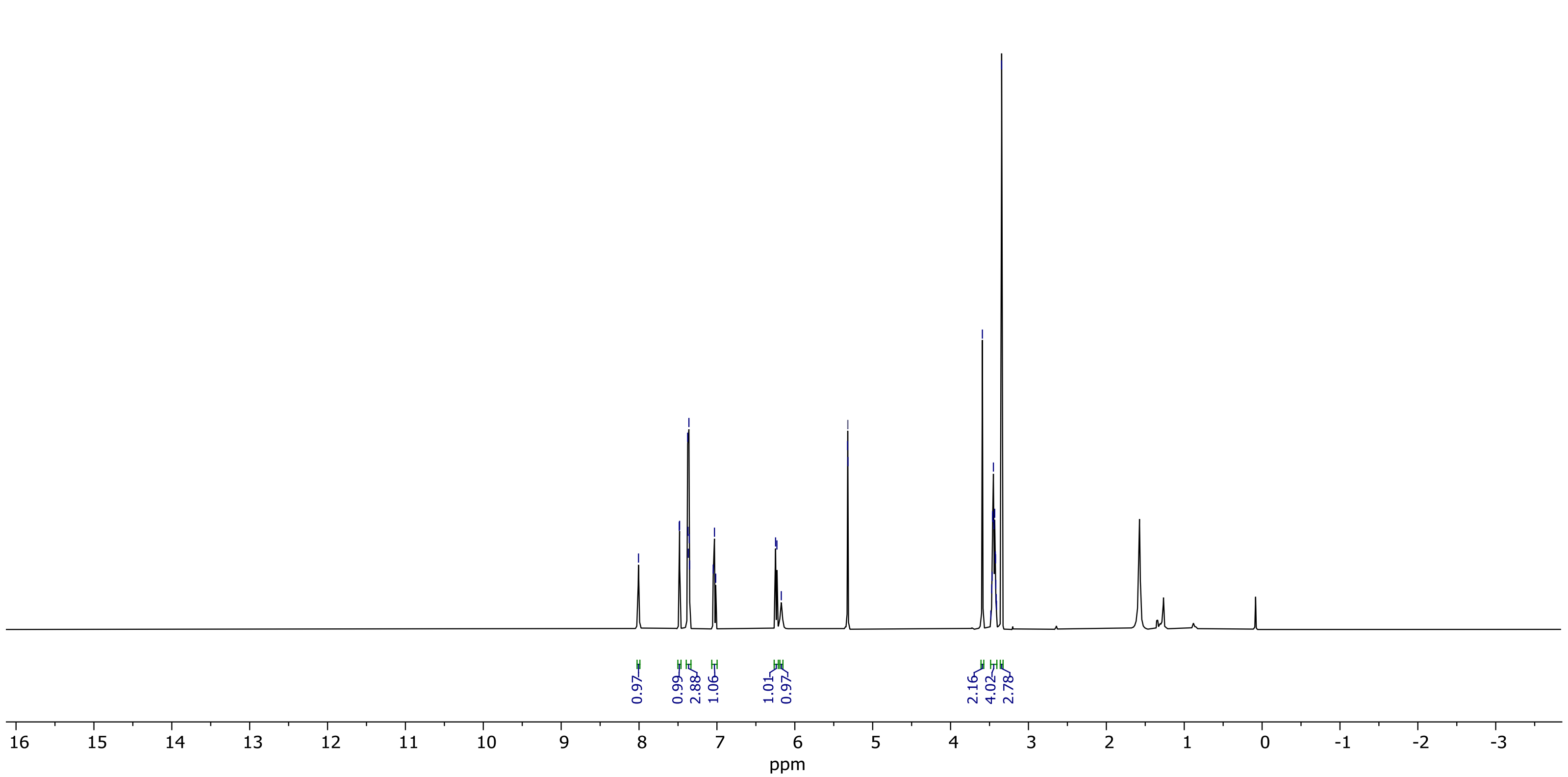

^{13}C NMR of **S74**
125 MHz, CD_2Cl_2, 298 K

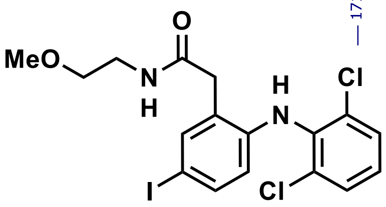

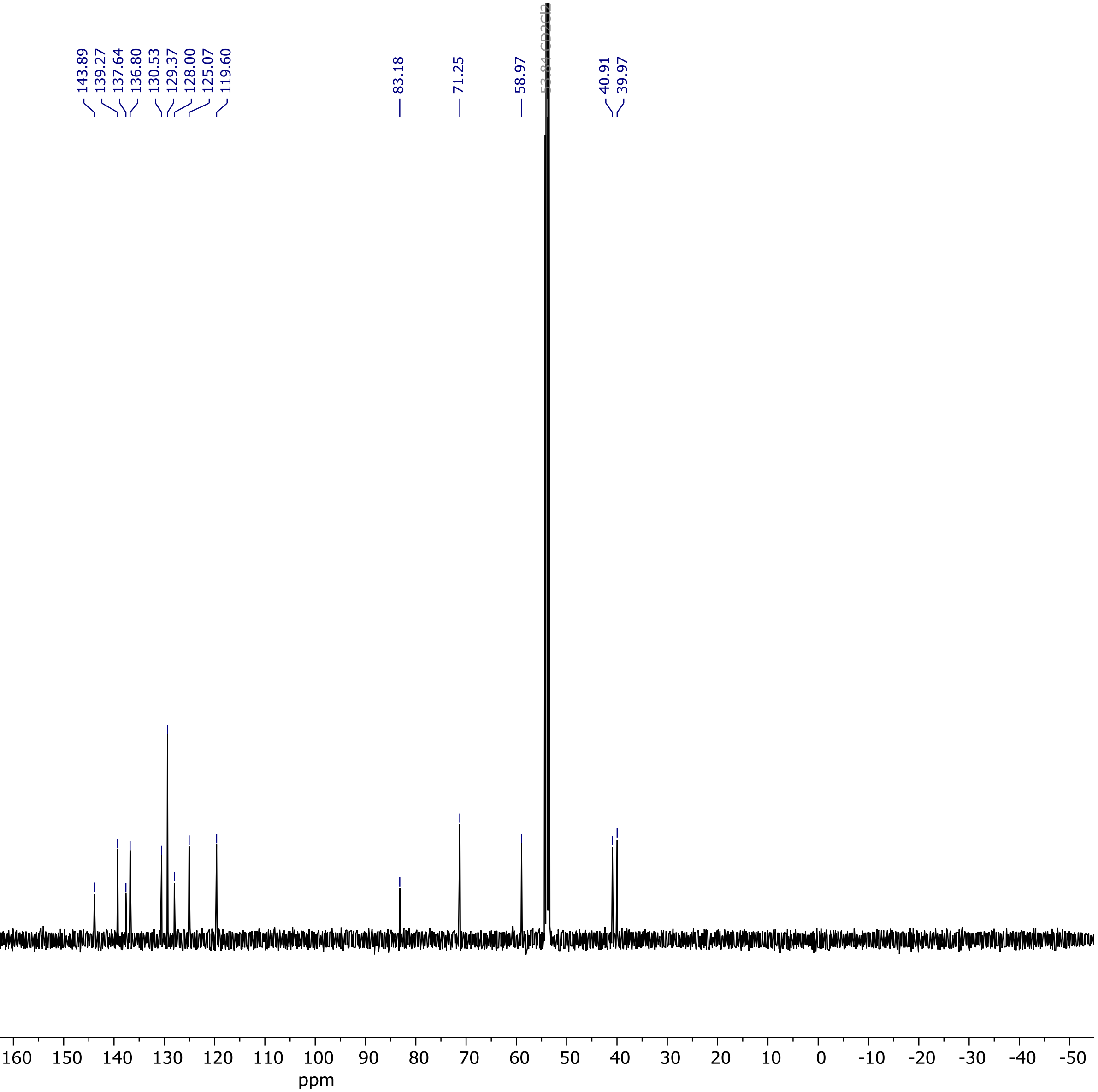

^{1}H NMR of **S75**
500 MHz, CD_2Cl_2, 298 K

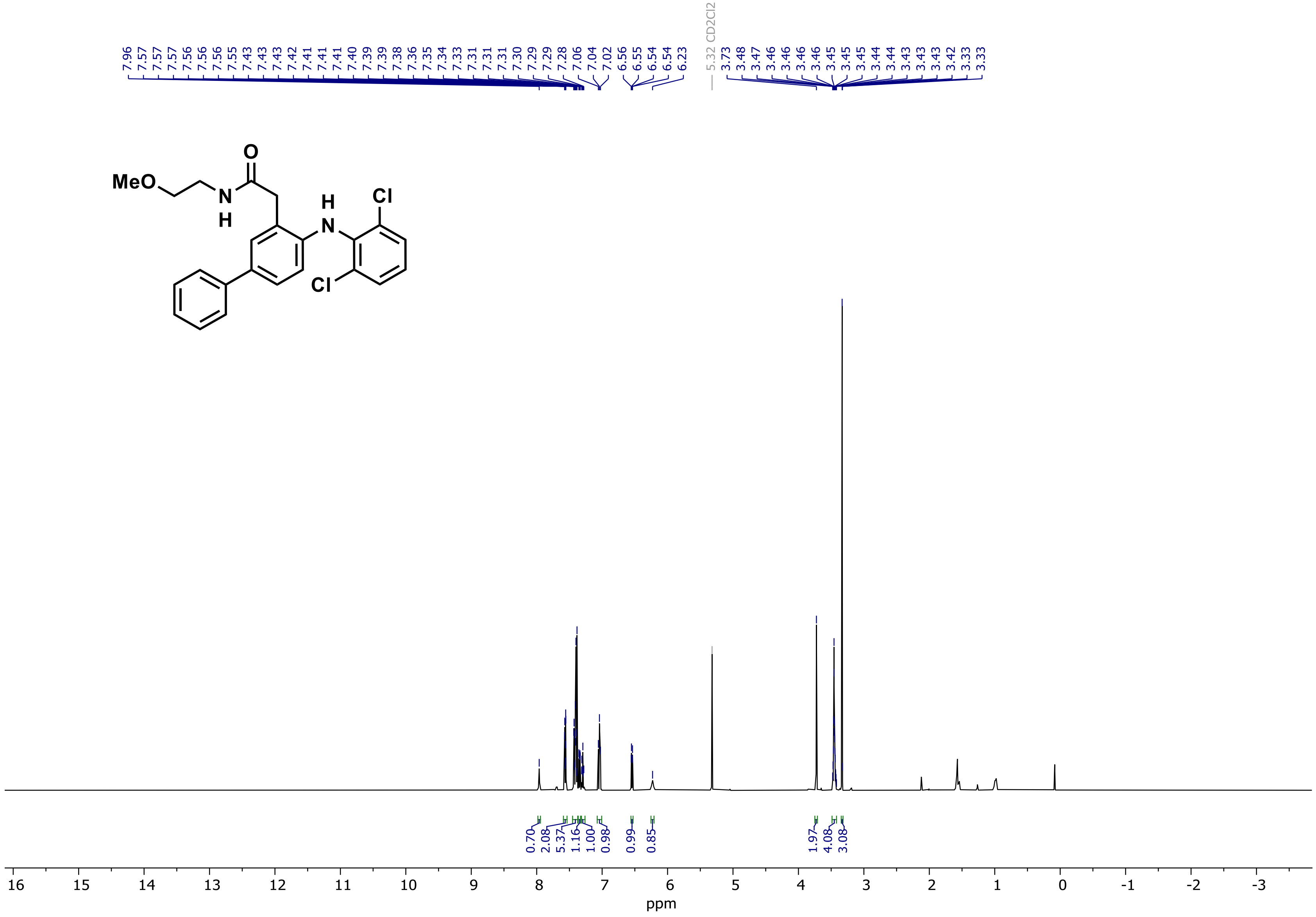

^{13}C NMR of **S75**
125 MHz, CD_2Cl_2, 298 K

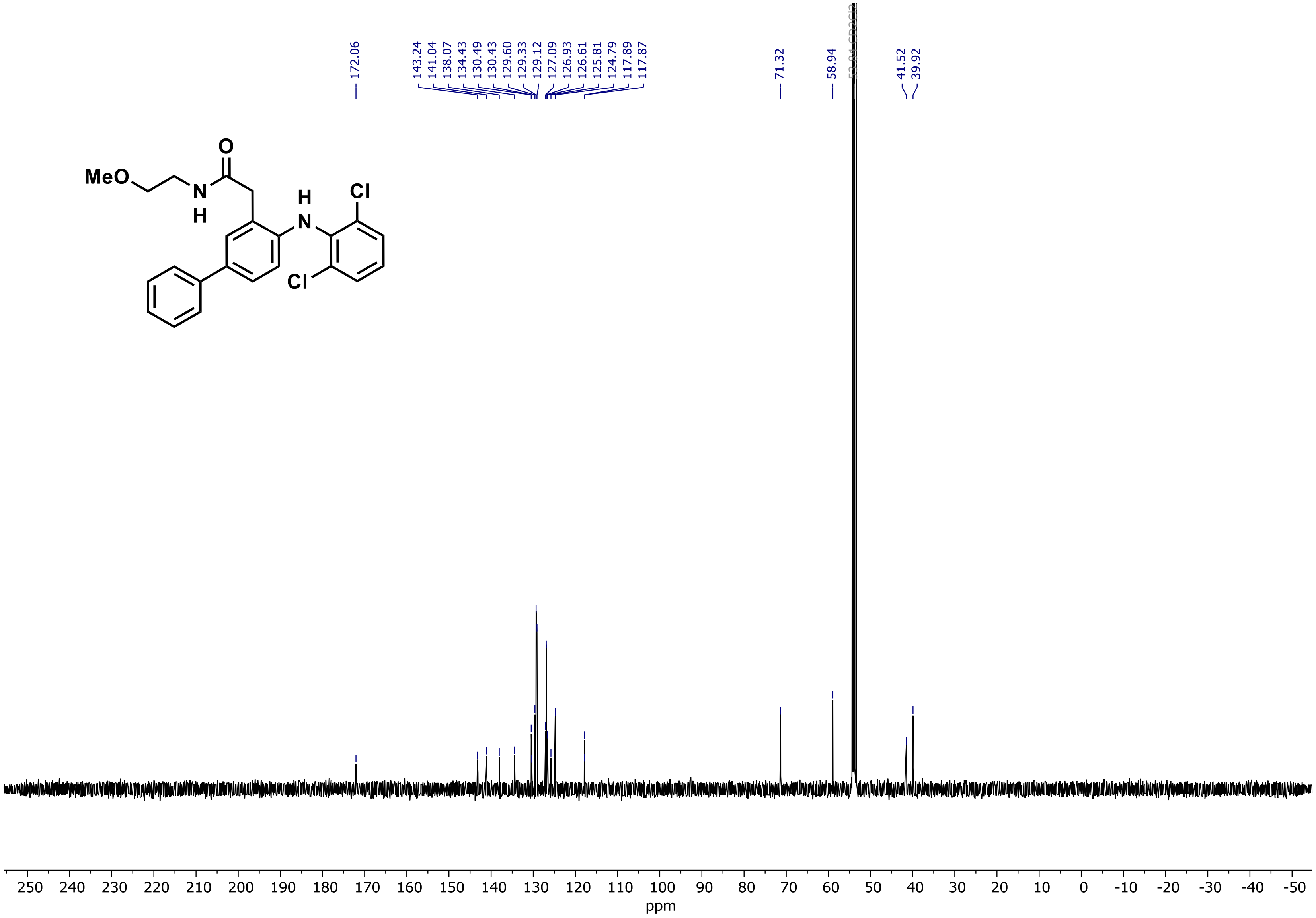

^{1}H NMR of **S76**
500 MHz, CD_2Cl_2, 298 K

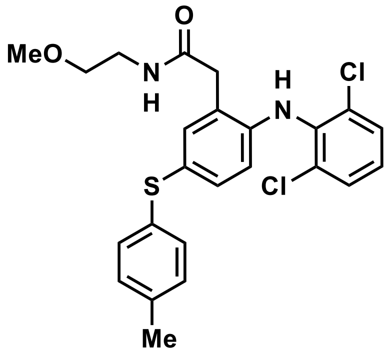

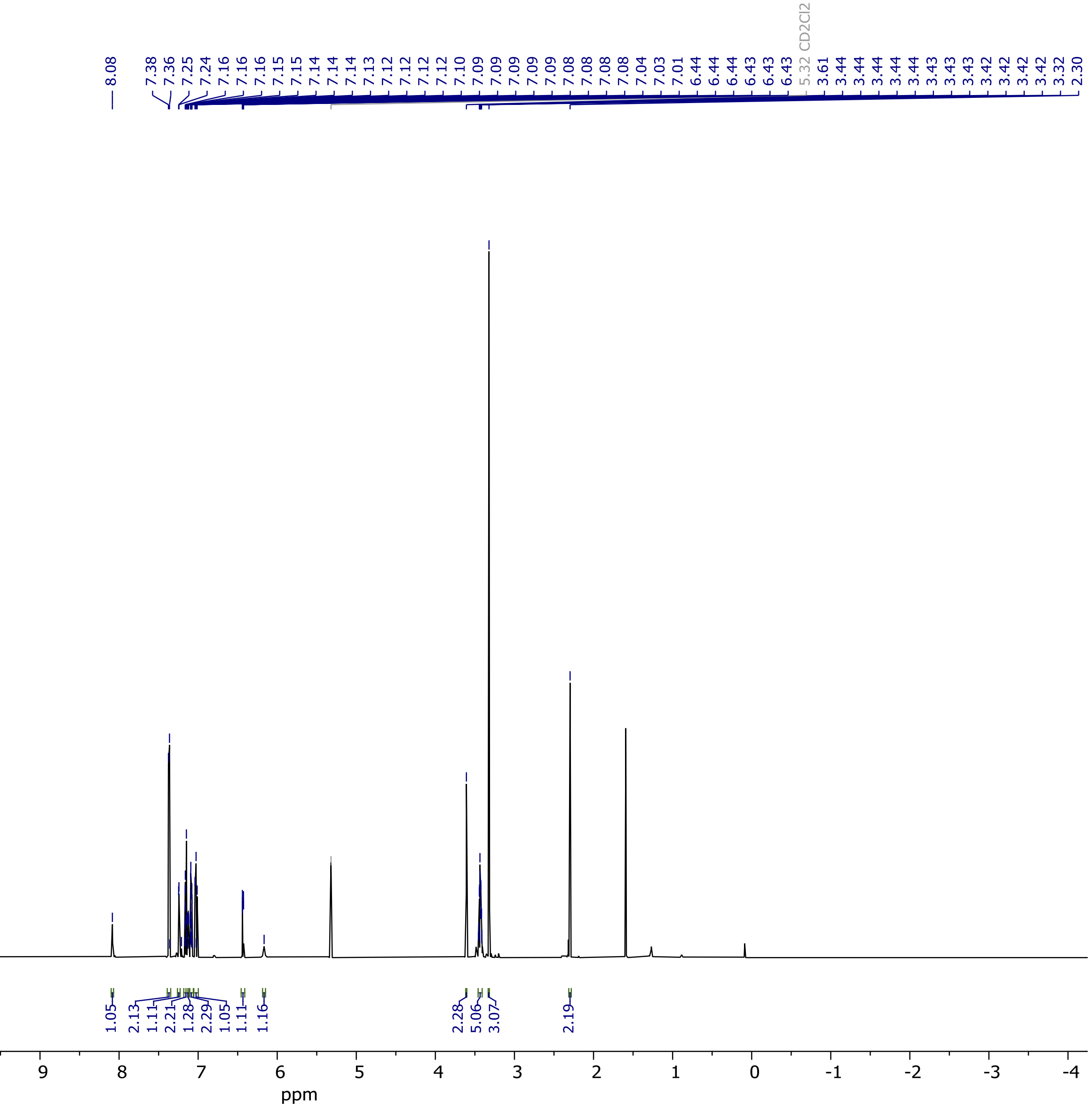

^{13}C NMR of **S76**
125 MHz, CD_2Cl_2, 298 K

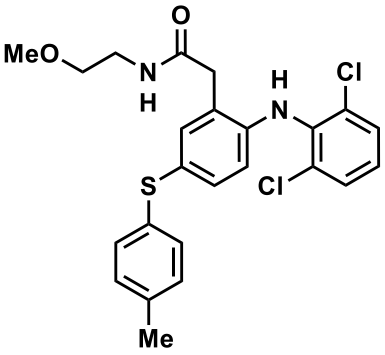

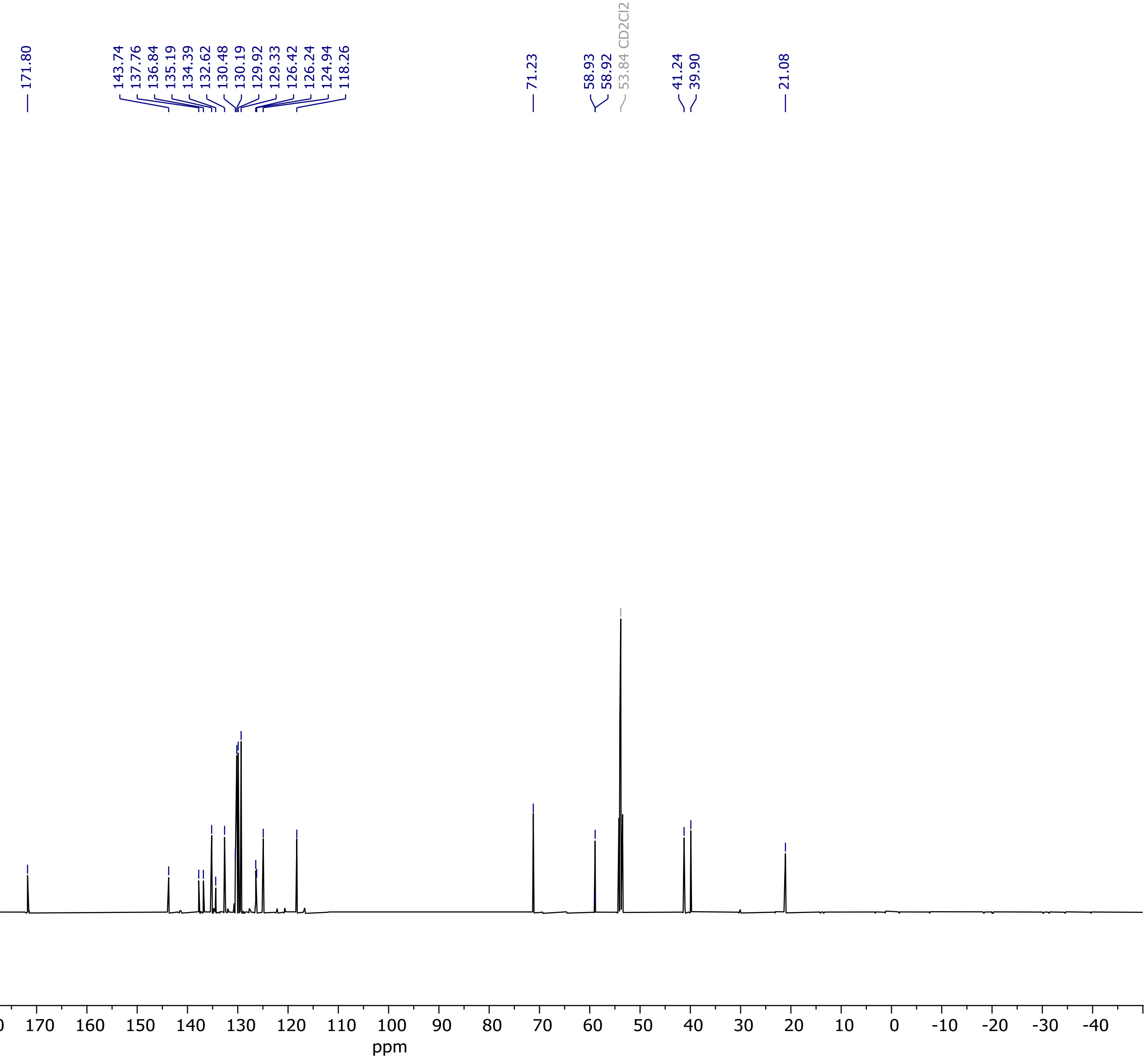

REFERENCES

1 Fulmer, G. R. *et al.* NMR Chemical Shifts of Trace Impurities: Common Laboratory Solvents, Organics, and Gases in Deuterated Solvents Relevant to the Organometallic Chemist. *Organometallics* **29**, 2176-2179, doi:10.1021/om100106e (2010).

2 Hale, P., A Handbook for DNA-Encoded Chemistry. John Wiley & Sons, Inc. (2014).

3 Sambrook, J., Molecular Cloning : a Laboratory Manual. Cold Spring Harbor, N.Y.: Cold Spring Harbor Laboratory Press (2001).

4 Li, Y. *et al.* Optimized Reaction Conditions for Amide Bond Formation in DNA-Encoded Combinatorial Libraries. *ACS Comb. Sci.* **18**, 438-443 (2016).

5 Sunkari, Y. K., Nguyen, T.-L., Siripuram, V. K. & Flajolet, M. Impact of organic chemistry conditions on DNA durability in the context of DNA-encoded library technology. *iScience* **26**, 107573 (2023).
